# Safety and efficacy of the ChAdOx1 nCoV-19 vaccine (AZD1222) against SARS-CoV-2: an interim analysis of four randomised controlled trials in Brazil, South Africa, and the UK

**DOI:** 10.1016/S0140-6736(20)32661-1

**Published:** 2021-01-09

**Authors:** Merryn Voysey, Sue Ann Costa Clemens, Shabir A Madhi, Lily Y Weckx, Pedro M Folegatti, Parvinder K Aley, Brian Angus, Vicky L Baillie, Shaun L Barnabas, Qasim E Bhorat, Sagida Bibi, Carmen Briner, Paola Cicconi, Andrea M Collins, Rachel Colin-Jones, Clare L Cutland, Thomas C Darton, Keertan Dheda, Christopher J A Duncan, Katherine R W Emary, Katie J Ewer, Lee Fairlie, Saul N Faust, Shuo Feng, Daniela M Ferreira, Adam Finn, Anna L Goodman, Catherine M Green, Christopher A Green, Paul T Heath, Catherine Hill, Helen Hill, Ian Hirsch, Susanne H C Hodgson, Alane Izu, Susan Jackson, Daniel Jenkin, Carina C D Joe, Simon Kerridge, Anthonet Koen, Gaurav Kwatra, Rajeka Lazarus, Alison M Lawrie, Alice Lelliott, Vincenzo Libri, Patrick J Lillie, Raburn Mallory, Ana V A Mendes, Eveline P Milan, Angela M Minassian, Alastair McGregor, Hazel Morrison, Yama F Mujadidi, Anusha Nana, Peter J O’Reilly, Sherman D Padayachee, Ana Pittella, Emma Plested, Katrina M Pollock, Maheshi N Ramasamy, Sarah Rhead, Alexandre V Schwarzbold, Nisha Singh, Andrew Smith, Rinn Song, Matthew D Snape, Eduardo Sprinz, Rebecca K Sutherland, Richard Tarrant, Emma C Thomson, M Estée Török, Mark Toshner, David P J Turner, Johan Vekemans, Tonya L Villafana, Marion E E Watson, Christopher J Williams, Alexander D Douglas, Adrian V S Hill, Teresa Lambe, Sarah C Gilbert, Andrew J Pollard, Oxford COVID Vaccine Trial Group, Marites Aban, Fatola Abayomi, Kushala Abeyskera, Jeremy Aboagye, Matthew Adam, Kirsty Adams, James Adamson, Yemi A. Adelaja, Gbadebo Adewetan, Syed Adlou, Khatija Ahmed, Yasmeen Akhalwaya, Saajida Akhalwaya, Andrew Alcock, Aabidah Ali, Elizabeth R. Allen, Lauren Allen, Thamires C. D. S. C Almeida, Mariana P.S. Alves, Fabio Amorim, Foteini Andritsou, Rachel Anslow, Matthew Appleby, Edward H. Arbe-Barnes, Mark P. Ariaans, Beatriz Arns, Laiana Arruda, Paula Azi, Lorena Azi, Gavin Babbage, Catherine Bailey, Kenneth F. Baker, Megan Baker, Natalie Baker, Philip Baker, Lisa Baldwin, Ioana Baleanu, Danieli Bandeira, Anna Bara, Marcella A.S. Barbosa, Debbie Barker, Gavin D. Barlow, Eleanor Barnes, Andrew S. Barr, Jordan R. Barrett, Jessica Barrett, Louise Bates, Alexander Batten, Kirsten Beadon, Emily Beales, Rebecca Beckley, Sandra Belij-Rammerstorfer, Jonathan Bell, Duncan Bellamy, Nancy Bellei, Sue Belton, Adam Berg, Laura Bermejo, Eleanor Berrie, Lisa Berry, Daniella Berzenyi, Amy Beveridge, Kevin R. Bewley, Helen Bexhell, Sutika Bhikha, Asad E. Bhorat, Zaheda E. Bhorat, Else Bijker, Geeta Birch, Sarah Birch, Adam Bird, Olivia Bird, Karen Bisnauthsing, Mustapha Bittaye, Katherine Blackstone, Luke Blackwell, Heather Bletchly, Caitlin L. Blundell, Susannah R. Blundell, Pritesh Bodalia, Bruno C. Boettger, Emma Bolam, Elena Boland, Daan Bormans, Nicola Borthwick, Francesca Bowring, Amy Boyd, Penny Bradley, Tanja Brenner, Phillip Brown, Claire Brown, Charlie Brown-O'Sullivan, Scott Bruce, Emily Brunt, Ruaridh Buchan, William Budd, Yusuf A. Bulbulia, Melanie Bull, Jamie Burbage, Hassan Burhan, Aileen Burn, Karen R. Buttigieg, Nicholas Byard, Ingrid Cabera Puig, Gloria Calderon, Anna Calvert, Susana Camara, Michelangelo Cao, Federica Cappuccini, João R. Cardoso, Melanie Carr, Miles W. Carroll, Andrew Carson-Stevens, Yasmin de M. Carvalho, José A.M. Carvalho, Helen R. Casey, Paul Cashen, Thais Castro, Lucia Carratala Castro, Katrina Cathie, Ana Cavey, José Cerbino-Neto, Jim Chadwick, David Chapman, Sue Charlton, Irina Chelysheva, Oliver Chester, Sunder Chita, Jee-Sun Cho, Liliana Cifuentes, Elizabeth Clark, Matthew Clark, Andrea Clarke, Elizabeth A. Clutterbuck, Sarah L.K. Collins, Christopher P. Conlon, Sean Connarty, Naomi Coombes, Cushla Cooper, Rachel Cooper, Lynne Cornelissen, Tumena Corrah, Catherine Cosgrove, Tony Cox, Wendy E.M. Crocker, Sarah Crosbie, Lorraine Cullen, Dan Cullen, Debora R.M.F. Cunha, Christina Cunningham, Fiona C. Cuthbertson, Suzete N. Farias Da Guarda, Larissa P. da Silva, Brad E. Damratoski, Zsofia Danos, Maria T.D.C. Dantas, Paula Darroch, Mehreen S. Datoo, Chandrabali Datta, Malika Davids, Sarah L. Davies, Hannah Davies, Elizabeth Davis, Judith Davis, John Davis, Maristela M.D. De Nobrega, Lis Moreno De Oliveira Kalid, David Dearlove, Tesfaye Demissie, Amisha Desai, Stefania Di Marco, Claudio Di Maso, Maria I.S. Dinelli, Tanya Dinesh, Claire Docksey, Christina Dold, Tao Dong, Francesca R. Donnellan, Tannyth Dos Santos, Thainá G. dos Santos, Erika Pachecho Dos Santos, Naomi Douglas, Charlotte Downing, Jonathan Drake, Rachael Drake-Brockman, Kimberley Driver, Ruth Drury, Susanna J. Dunachie, Benjamin S. Durham, Lidiana Dutra, Nicholas J.W. Easom, Samual van Eck, Mandy Edwards, Nick J. Edwards, Omar M. El Muhanna, Sean C. Elias, Mike Elmore, Marcus English, Alisgair Esmail, Yakub Moosa Essack, Eoghan Farmer, Mutjaba Farooq, Madi Farrar, Leonard Farrugia, Beverley Faulkner, Sofiya Fedosyuk, Sally Felle, Shuo Feng, Carla Ferreira Da Silva, Samantha Field, Richard Fisher, Amy Flaxman, James Fletcher, Hazel Fofie, Henry Fok, Karen J. Ford, Jamie Fowler, Pedro H.A. Fraiman, Emma Francis, Marilia M. Franco, John Frater, Marilúcia S.M. Freire, Samantha H. Fry, Sabrina Fudge, Julie Furze, Michelle Fuskova, Pablo Galian-Rubio, Eva Galiza, Harriet Garlant, Madita Gavrila, Ailsa Geddes, Karyna A. Gibbons, Ciaran Gilbride, Hardeep Gill, Sharon Glynn, Kerry Godwin, Karishma Gokani, Ursula Carvalho Goldoni, Maria Goncalves, Isabela G.S. Gonzalez, Jayne Goodwin, Amina Goondiwala, Katherine Gordon-Quayle, Giacomo Gorini, Janet Grab, Lara Gracie, Melanie Greenland, Nicola Greenwood, Johann Greffrath, Marisa M. Groenewald, Leonardo Grossi, Gaurav Gupta, Mark Hackett, Bassam Hallis, Mainga Hamaluba, Elizabeth Hamilton, Joseph Hamlyn, Daniel Hammersley, Aidan T. Hanrath, Brama Hanumunthadu, Stephanie A. Harris, Clair Harris, Tara Harris, Thomas D. Harrison, Daisy Harrison, Thomas C. Hart, Birgit Hartnell, Shadin Hassan, John Haughney, Sophia Hawkins, Jodie Hay, Ian Head, John Henry, Macarena Hermosin Herrera, David B. Hettle, Jennifer Hill, Gina Hodges, Elizea Horne, Mimi M. Hou, Catherine Houlihan, Elizabeth Howe, Nicola Howell, Jonathan Humphreys, Holly E. Humphries, Katrina Hurley, Claire Huson, Angela Hyder-Wright, Catherine Hyams, Sabina Ikram, Alka Ishwarbhai, Monica Ivan, Poppy Iveson, Vidyashankara Iyer, Frederic Jackson, Jeanne De Jager, Shameem Jaumdally, Helen Jeffers, Natasha Jesudason, Bryony Jones, Kathryn Jones, Elizabeth Jones, Christopher Jones, Marianna Rocha Jorge, Aylin Jose, Amar Joshi, Eduardo A.M.S. Júnior, Joanne Kadziola, Reshma Kailath, Faeeza Kana, Konstantinos Karampatsas, Mwila Kasanyinga, Jade Keen, Elizabeth J. Kelly, Dearbhla M. Kelly, Debbie Kelly, Sarah Kelly, David Kerr, Renato de Ávila Kfouri, Liaquat Khan, Baktash Khozoee, Sarah Kidd, Annabel Killen, Jasmin Kinch, Patrick Kinch, Lloyd D.W. King, Thomas B. King, Lucy Kingham, Paul Klenerman, Francesca Knapper, Julian C. Knight, Daniel Knott, Stanislava Koleva, Matilda Lang, Gail Lang, Colin W. Larkworthy, Jessica P.J. Larwood, Rebecca Law, Erica M. Lazarus, Amanda Leach, Emily A. Lees, Nana-Marie Lemm, Alvaro Lessa, Stephanie Leung, Yuanyuan Li, Amelia M. Lias, Kostas Liatsikos, Aline Linder, Samuel Lipworth, Shuchang Liu, Xinxue Liu, Adam Lloyd, Stephanie Lloyd, Lisa Loew, Raquel Lopez Ramon, Leandro Lora, Vicki Lowthorpe, Kleber Luz, Jonathan C. MacDonald, Gordon MacGregor, Meera Madhavan, David O. Mainwaring, Edson Makambwa, Rebecca Makinson, Mookho Malahleha, Ross Malamatsho, Garry Mallett, Kushal Mansatta, Takalani Maoko, Katlego Mapetla, Natalie G. Marchevsky, Spyridoula Marinou, Emma Marlow, Gabriela N. Marques, Paula Marriott, Richard P. Marshall, Julia L. Marshall, Flávia J. Martins, Masebole Masenya, Mduduzi Masilela, Shauna K. Masters, Moncy Mathew, Hosea Matlebjane, Kedidimetse Matshidiso, Olga Mazur, Andrea Mazzella, Hugh McCaughan, Joanne McEwan, Joanna McGlashan, Lorna McInroy, Zoe McIntyre, Daniela McLenaghan, Nicky McRobert, Steve McSwiggan, Clare Megson, Savviz Mehdipour, Wilma Meijs, Renata N.Á. Mendonça, Alexander J. Mentzer, Neginsadat Mirtorabi, Celia Mitton, Sibusiso Mnyakeni, Fiona Moghaddas, Kgaogelo Molapo, Mapule Moloi, Maria Moore, M. Isabel Moraes-Pinto, Marni Moran, Ella Morey, Róisín Morgans, Susan Morris, Sheila Morris, Helen C. Morris, Franca Morselli, Gertraud Morshead, Richard Morter, Lynelle Mottal, Andrew Moultrie, Nathifa Moya, Mushiya Mpelembue, Sibekezelo Msomi, Yvonne Mugodi, Ekta Mukhopadhyay, Jilly Muller, Alasdair Munro, Claire Munro, Sarah Murphy, Philomena Mweu, Celia Hatsuko Myasaki, Gurudutt Naik, Kush Naker, Eleni Nastouli, Abida Nazir, Bongani Ndlovu, Fabio Neffa, Cecilia Njenga, Helena Noal, Andrés Noé, Gabrielle Novaes, Fay L. Nugent, Géssika Nunes, Katie O'Brien, Daniel O'Connor, Miranda Odam, Suzette Oelofse, Blanche Oguti, Victoria Olchawski, Neil J. Oldfield, Marianne G. Oliveira, Catarina Oliveira, Angela Oosthuizen, Paula O'Reilly, Piper Osborne, David R.J. Owen, Lydia Owen, Daniel Owens, Nelly Owino, Mihaela Pacurar, Brenda V.B. Paiva, Edna M.F. Palhares, Susan Palmer, Sivapriyai Parkinson, Helena M.R.T. Parracho, Karen Parsons, Dipak Patel, Bhumika Patel, Faeezah Patel, Kelly Patel, Maia Patrick-Smith, Ruth O. Payne, Yanchun Peng, Elizabeth J. Penn, Anna Pennington, Marco Polo Peralta Alvarez, James Perring, Nicola Perry, Rubeshan Perumal, Sahir Petkar, Tricia Philip, Daniel J. Phillips, Jennifer Phillips, Mary Kgomotso Phohu, Lorinda Pickup, Sonja Pieterse, Jo Piper, Dimitra Pipini, Mary Plank, Joan Du Plessis, Samuel Pollard, Jennifer Pooley, Anil Pooran, Ian Poulton, Claire Powers, Fernando B. Presa, David A. Price, Vivien Price, Marcelo Primeira, Pamela C. Proud, Samuel Provstgaard-Morys, Sophie Pueschel, David Pulido, Sheena Quaid, Ria Rabara, Alexandra Radford, Kajal Radia, Durga Rajapaska, Thurkka Rajeswaran, Alberto San Francisco Ramos, Fernando Ramos Lopez, Tommy Rampling, Jade Rand, Helen Ratcliffe, Tom Rawlinson, David Rea, Byron Rees, Jesús Reiné, Mila Resuello-Dauti, Emilia Reyes Pabon, Carla M. Ribiero, Marivic Ricamara, Alex Richter, Neil Ritchie, Adam J. Ritchie, Alexander J. Robbins, Hannah Roberts, Ryan E. Robinson, Hannah Robinson, Talita T. Rocchetti, Beatriz Pinho Rocha, Sophie Roche, Christine Rollier, Louisa Rose, Amy L. Ross Russell, Lindie Rossouw, Simon Royal, Indra Rudiansyah, Sarah Ruiz, Stephen Saich, Claudia Sala, Jessica Sale, Ahmed M. Salman, Natalia Salvador, Stephannie Salvador, Milla Sampaio, Annette D. Samson, Amada Sanchez-Gonzalez, Helen Sanders, Katherine Sanders, Erika Santos, Mayara F.S. Santos Guerra, Iman Satti, Jack E. Saunders, Caroline Saunders, Aakifah Sayed, Ina Schim van der Loeff, Annina B. Schmid, Ella Schofield, Gavin Screaton, Samiullah Seddiqi, Rameswara R. Segireddy, Roberta Senger, Sonia Serrano, Rajiv Shah, Imam Shaik, Hannah E. Sharpe, Katherine Sharrocks, Robert Shaw, Adam Shea, Amy Shepherd, James G. Shepherd, Farah Shiham, Emad Sidhom, Sarah E. Silk, Antonio Carlos da Silva Moraes, Gilberto Silva-Junior, Laura Silva-Reyes, Anderson D. Silveira, Mariana B.V. Silveira, Jaisi Sinha, Donal T. Skelly, Daniel C. Smith, Nick Smith, Holly E. Smith, David J. Smith, Catherine C. Smith, Airanuédida Soares, Tiago Soares, Carla Solórzano, Guilherme L. Sorio, Kim Sorley, Tiffany Sosa-Rodriguez, Cinthia M.C.D.L. Souza, Bruno S.D.F. Souza, Alessandra R. Souza, Alexandra J. Spencer, Fernanda Spina, Louise Spoors, Lizzie Stafford, Imogen Stamford, Igor Starinskij, Ricardo Stein, Jill Steven, Lisa Stockdale, Lisa V. Stockwell, Louise H. Strickland, Arabella C. Stuart, Ann Sturdy, Natalina Sutton, Anna Szigeti, Abdessamad Tahiri-Alaoui, Rachel Tanner, Carol Taoushanis, Alexander W. Tarr, Keja Taylor, Ursula Taylor, Iona Jennifer Taylor, Justin Taylor, Rebecca te Water Naude, Yrene Themistocleous, Andreas Themistocleous, Merin Thomas, Kelly Thomas, Tonia M. Thomas, Asha Thombrayil, Fawziyah Thompson, Amber Thompson, Kevin Thompson, Ameeka Thompson, Julia Thomson, Viv Thornton-Jones, Patrick J. Tighe, Lygia Accioly Tinoco, Gerlynn Tiongson, Bonolo Tladinyane, Michele Tomasicchio, Adriana Tomic, Susan Tonks, James Towner, Nguyen Tran, Julia Tree, Gerry Trillana, Charlotte Trinham, Rose Trivett, Adam Truby, Betty Lebogang Tsheko, Aadil Turabi, Richard Turner, Cheryl Turner, Marta Ulaszewska, Benjamin R. Underwood, Rachel Varughese, Dennis Verbart, Marije Verheul, Iason Vichos, Taiane Vieira, Claire S. Waddington, Laura Walker, Erica Wallis, Matthew Wand, Deborah Warbick, Theresa Wardell, George Warimwe, Sarah C. Warren, Bridget Watkins, Ekaterina Watson, Stewart Webb, Alice Webb-Bridges, Angela Webster, Jessica Welch, Jeanette Wells, Alison West, Caroline White, Rachel White, Paul Williams, Rachel L. Williams, Rebecca Winslow, Mark Woodyer, Andrew T. Worth, Danny Wright, Marzena Wroblewska, Andy Yao, Rafael Zimmer, Dalila Zizi, Peter Zuidewind

**Affiliations:** a Oxford Vaccine Group, Department of Paediatrics, University of Oxford, Oxford, UK; b Jenner Institute, Nuffield Department of Medicine, University of Oxford, UK; c Institute of Global Health, University of Siena, Siena, Brazil; d Department of Paediatrics, University of Oxford, Oxford, UK; e Clinical BioManufacturing Facility, University of Oxford, Oxford, UK; f MRC Vaccines and Infectious Diseases Analytics Research Unit, Johannesburg, South Africa; g Department of Pediatrics, Universidade Federal de São Paulo, São Paulo, Brazil; g Respiratory and Meningeal Pathogens Research Unit, University of the Witwatersrand, Johannesburg, South Africa; i Family Centre for Research with Ubuntu, Department of Paediatrics, University of Stellenbosch, Cape Town, South Africa; j Soweto Clinical Trials Centre, Soweto, South Africa; k Perinatal HIV Research Unit, Faculty of Health Sciences, University of the Witwatersrand, Johannesburg, South Africa; l Wits Reproductive Health and HIV Institute, Faculty of Health Sciences, University of the Witwatersrand, Johannesburg, South Africa; m Department of Clinical Sciences, Liverpool School of Tropical Medicine and Liverpool University Hospitals NHS Foundation Trust, Liverpool, UK; n Department of Infection, Immunity and Cardiovascular Disease, University of Sheffield, Sheffield, UK; o Department of Infection and Tropical Medicine, Sheffield Teaching Hospitals NHS Foundation Trust, Sheffield, UK; p Division of Pulmonology, Groote Schuur Hospital and the University of Cape Town, South Africa; q Faculty of Infectious and Tropical Diseases, Department of Immunology and Infection, London School of Hygiene & Tropical Medicine, London, UK; r Department of Infection and Tropical Medicine, Newcastle upon Tyne Hospitals NHS Foundation Trust, Newcastle upon Tyne, UK; s Translational and Clinical Research Institute, Immunity and Inflammation Theme, Newcastle University, Newcastle upon Tyne, UK; t NIHR Southampton Clinical Research Facility and Biomedical Research Centre, University Hospital Southampton NHS Foundation Trust, Southampton, UK; u Faculty of Medicine and Institute for Life Sciences, University of Southampton, Southampton, UK; v School of Population Health Sciences, University of Bristol and University Hospitals Bristol and Weston NHS Foundation Trust, Bristol, UK; w Department of Infection, Guy's and St Thomas’ NHS Foundation Trust, St Thomas’ Hospital, London, UK; x MRC Clinical Trials Unit, University College London, London, UK; y NIHR/Wellcome Trust Clinical Research Facility, University Hospitals Birmingham NHS Foundation Trust, Birmingham, UK; z St George's Vaccine Institute, St George's, University of London, London, UK; aa AstraZeneca BioPharmaceuticals, Cambridge, UK; ab VIDA—Vaccines and Infectious Diseases Analytical Research Unit, Johannesburg, South Africa; ac Severn Pathology, North Bristol NHS Trust, Bristol, UK; ad NIHR UCLH Clinical Research Facility and NIHR UCLH Biomedical Research Centre, London, UK; af Department of Infection, Hull University Teaching Hospitals NHS Trust, UK; af Escola Bahiana de Medicina e Saúde Pública, Salvador, Braziland Hospital São Rafael, Salvador, Brazil; ag Instituto D’Or, Salvador, Brazil; ah Department of Infectious Diseases, Universidade Federal do Rio Grande do Norte, Natal, Brazil; ai London Northwest University Healthcare, Harrow, UK; aj Setshaba Research Centre, Pretoria, South Africa; ak Department of Internal Medicine, Hospital Quinta D’Or, Rio de Janeiro, Brazil; al Instituto D’Or de Pesquisa e Ensino (IDOR), Rio de Janeiro, Brazil; am Department of Internal Medicine, Universidade UNIGRANRIO, Rio de Janeiro, Brazil; an NIHR Imperial Clinical Research Facility and NIHR Imperial Biomedical Research Centre, London, UK; ao Clinical Research Unit, Department of Clinical Medicine, Universidade Federal de Santa Maria, Santa Maria, Brazil; ap College of Medical, Veterinary & Life Sciences, Glasgow Dental Hospital & School, University of Glasgow, Glasgow, UK; aq Division of Infectious Diseases, Boston Children's Hospital, Boston, MA, USA; ar Infectious Diseases Service, Hospital de Clinicas de Porto Alegre, Universidade Federal do Rio Grande do Sul, Porto Alegre, Brazil; as Clinical Infection Research Group, Regional Infectious Diseases Unit, Western General Hospital, Edinburgh, UK; at MRC-University of Glasgow Centre for Virus Research & Department of Infectious Diseases, Queen Elizabeth University Hospital, Glasgow, UK; au Department of Medicine, University of Cambridge, UK; av Cambridge University Hospitals NHS Foundation Trust, Cambridge, UK; aw Heart Lung Research Institute, Department of Medicine, University of Cambridge and Royal Papworth Hospital NHS Foundation Trust, Cambridge, UK; ax University of Nottingham and Nottingham University Hospitals NHS Trust, UK; ay Public Health Wales, Cardiff, Wales; az Aneurin Bevan University Health Board, Newport, UK

## Abstract

**Background:**

A safe and efficacious vaccine against severe acute respiratory syndrome coronavirus 2 (SARS-CoV-2), if deployed with high coverage, could contribute to the control of the COVID-19 pandemic. We evaluated the safety and efficacy of the ChAdOx1 nCoV-19 vaccine in a pooled interim analysis of four trials.

**Methods:**

This analysis includes data from four ongoing blinded, randomised, controlled trials done across the UK, Brazil, and South Africa. Participants aged 18 years and older were randomly assigned (1:1) to ChAdOx1 nCoV-19 vaccine or control (meningococcal group A, C, W, and Y conjugate vaccine or saline). Participants in the ChAdOx1 nCoV-19 group received two doses containing 5 × 10^10^ viral particles (standard dose; SD/SD cohort); a subset in the UK trial received a half dose as their first dose (low dose) and a standard dose as their second dose (LD/SD cohort). The primary efficacy analysis included symptomatic COVID-19 in seronegative participants with a nucleic acid amplification test-positive swab more than 14 days after a second dose of vaccine. Participants were analysed according to treatment received, with data cutoff on Nov 4, 2020. Vaccine efficacy was calculated as 1 - relative risk derived from a robust Poisson regression model adjusted for age. Studies are registered at ISRCTN89951424 and ClinicalTrials.gov, NCT04324606, NCT04400838, and NCT04444674.

**Findings:**

Between April 23 and Nov 4, 2020, 23 848 participants were enrolled and 11 636 participants (7548 in the UK, 4088 in Brazil) were included in the interim primary efficacy analysis. In participants who received two standard doses, vaccine efficacy was 62·1% (95% CI 41·0–75·7; 27 [0·6%] of 4440 in the ChAdOx1 nCoV-19 group *vs*71 [1·6%] of 4455 in the control group) and in participants who received a low dose followed by a standard dose, efficacy was 90·0% (67·4–97·0; three [0·2%] of 1367 *vs* 30 [2·2%] of 1374; p_*interaction*_=0·010). Overall vaccine efficacy across both groups was 70·4% (95·8% CI 54·8–80·6; 30 [0·5%] of 5807 *vs* 101 [1·7%] of 5829). From 21 days after the first dose, there were ten cases hospitalised for COVID-19, all in the control arm; two were classified as severe COVID-19, including one death. There were 74 341 person-months of safety follow-up (median 3·4 months, IQR 1·3–4·8): 175 severe adverse events occurred in 168 participants, 84 events in the ChAdOx1 nCoV-19 group and 91 in the control group. Three events were classified as possibly related to a vaccine: one in the ChAdOx1 nCoV-19 group, one in the control group, and one in a participant who remains masked to group allocation.

**Interpretation:**

ChAdOx1 nCoV-19 has an acceptable safety profile and has been found to be efficacious against symptomatic COVID-19 in this interim analysis of ongoing clinical trials.

**Funding:**

UK Research and Innovation, National Institutes for Health Research (NIHR), Coalition for Epidemic Preparedness Innovations, Bill & Melinda Gates Foundation, Lemann Foundation, Rede D’Or, Brava and Telles Foundation, NIHR Oxford Biomedical Research Centre, Thames Valley and South Midland's NIHR Clinical Research Network, and AstraZeneca.

**Research in context**

**Evidence before this study**

We searched PubMed for research articles published from database inception until Nov 23, 2020, with no language restrictions, using the terms “SARS-CoV-2”, “vaccine”, “clinical trial”, and “efficacy”. There were no peer-reviewed publications available on efficacy of any severe acute respiratory syndrome coronavirus 2 (SARS-CoV-2) vaccines in development and, at the time of the search, there were no licensed vaccines against SARS-CoV-2. Three vaccine developers recently reported initial efficacy results from phase 3 trials in the media (Pfizer/BioNTech, Moderna, and the Gamaleya National Research Center). Pfizer/BioNTech and Moderna, both developing mRNA vaccines, have reported initial efficacy results of 95% in their primary analysis (Pfizer/BioNTech) and 94·5% in an interim analysis (Moderna). We have previously published safety and immunogenicity results of ChAdOx1 nCoV-19 (AZD1222) for different age groups in phase 1/2 and 2/3 trials.

**Added value of this study**

We report on the first clinical efficacy results of ChAdOx1 nCoV-19 in a pooled analysis of phase 2/3 trials in the UK and Brazil, and safety data from more than 20 000 participants enrolled across four clinical trials in the UK, Brazil, and South Africa. ChAdOx1 nCoV-19 has an acceptable safety profile and is efficacious against symptomatic COVID-19, with no hospital admissions or severe cases reported in the ChAdOx1 nCoV-19 arm. The vaccine can be stored and distributed at 2–8°C, making it particularly suitable for global distribution.

**Implications of all the available evidence**

The development of safe, effective, affordable, and deployable vaccines against COVID-19 remains paramount in solving the pandemic crisis and re-establishing normality. The positive results presented here support regulatory submissions for conditional or emergency use of ChAdOx1 nCoV-19.

## Introduction

As the COVID-19 pandemic, caused by severe acute respiratory syndrome coronavirus 2 (SARS-CoV-2), continues to unfold, there has been widespread impact on health, including substantial mortality among older adults and those with pre-existing health conditions,^1^, ^2^ and repercussions for the global economy, caused by physical distancing measures, with the greatest consequences for the most vulnerable in society.

Despite global spread of the virus, a large proportion of the population in many countries is thought to have thus far escaped infection and remains non-immune to SARS-CoV-2.^3^ Vaccines could play an important role in increasing population immunity, preventing severe disease, and reducing the ongoing health crisis. In response, rapid global efforts to develop and test vaccines against SARS-CoV-2 have led to an unprecedented number of candidate vaccines starting clinical trials during 2020. Currently, 48 vaccines are under clinical evaluation.^4^ Several of these have shown good safety and immunogenicity, and 11 of these are currently being evaluated in phase 3 clinical efficacy studies.

The ChAdOx1 nCoV-19 vaccine (AZD1222) was developed at Oxford University and consists of a replication-deficient chimpanzee adenoviral vector ChAdOx1, containing the SARS-CoV-2 structural surface glycoprotein antigen (spike protein; nCoV-19) gene.

Following initiation of a phase 1 clinical trial in the UK (COV001) on April 23, 2020, three further randomised controlled trials of the candidate vaccine were initiated across the UK (COV002), Brazil (COV003), and South Africa (COV005). A further phase 1/2 trial has recently been initiated in Kenya and is not reported here. The immunogenicity results from the phase 1/2 UK study, COV001, in 1077 healthy adults aged 18–55 years,^5^ and a phase 2 cohort in COV002 in older adults (≥56 years)^6^ have been published and show an acceptable safety profile for the vaccine with induction of binding and neutralising antibodies as well as generation of interferon-γ enzyme-linked immunospot responses, with higher antibody titres after a second dose of vaccine.^5^, ^6^, ^7^

The phase 1 study (COV001) included an efficacy cohort and the phase 2 and 3 studies (COV002, COV003, and COV005) expanded enrolment to a wider population of participants with higher likelihood of exposure to the virus, such as health-care workers. Exclusion criteria were reduced for phase 3 trials, so that older adults and individuals with a range of comorbidities were also enrolled.

All studies have completed enrolment of their respective efficacy cohorts and are in the follow-up phase. Paediatric studies have not yet been initiated.

Here, we present the combined interim analysis of efficacy and safety from randomised controlled trials of ChAdOx1 nCoV-19.

## Methods

### Overview

This interim analysis of the efficacy and safety of the ChAdOx1 nCoV-19 vaccine includes data from four ongoing blinded, randomised, controlled trials done across three countries: COV001 (phase 1/2; UK), COV002 (phase 2/3; UK), COV003 (phase 3; Brazil), and COV005 (phase 1/2; South Africa). The interim efficacy is being assessed by a prespecified global pooled analysis combining data from COV002 and COV003. The safety of the vaccine is being assessed using data from all four studies (appendix 1 pp 3–4). Three of the studies are single blind and one is double blind (COV005). Primary efficacy was assessed in participants who received two doses of the vaccine. All four studies included participants who received two doses, with a booster dose incorporated into the three trials^6^ that were initially designed to assess a single-dose of ChAdOx1 nCoV-19 compared with control (COV001, COV002, and COV003) after review of the antibody response data from COV001.

Despite minor differences across the studies, there is sufficient consistency to justify the proposal for pooled analysis of data, which will provide greater precision for both efficacy and safety outcomes than can be achieved in individual studies and provides a broader understanding of the use of the vaccine in different populations. Once the studies were underway, a statistical analysis plan for the global pooled analysis of these studies was developed before data lock on Nov 4, 2020, and analysis, and was finalised with extensive feedback from national and international regulators (including the Medicines and Healthcare Products Regulatory Agency [UK] and the European Medicines Agency [EU]), including justification for including groups receiving different vaccine doses in the analysis (see statistical analysis plan for further details; appendix 2 pp 2–73). All participants in the four trials provided written informed consent.

Details of amendments to the four trial protocols and the statistical analysis plan are included in appendix 2 (pp 9, 178–182, 327–335, 438–441, 548–550).

### Study design and participants

#### COV001 (UK)

COV001 is a continuing single-blind phase 1/2 clinical trial in five sites in the UK, which began on April 23, 2020, and enrolled 1077 healthy volunteers aged 18–55 years, as previously described.^5^ Briefly, healthy adult participants were enrolled after screening to exclude those with pre-existing health conditions. Participants were randomly assigned 1:1 to receive ChAdOx1 nCoV-19 at a dose of 5 × 10^10^ viral particles (standard dose), measured using spectrophotometry, or meningococcal group A, C, W, and Y conjugate vaccine (MenACWY) as control. An open-label non-randomised subgroup of ten participants were given two doses of ChAdOx1 nCoV-19 28 days apart, as previously reported.^5^ This study was originally planned as a single-dose study and 88 participants in the phase 1 part of the study remain recipients of a single dose. However, the protocol was modified to a two-dose regime, following an amendment on July 30, 2020 (version 9.0; appendix 2 pp 180–181), for the remaining phase 2 cohorts as a result of robust booster responses identified in the evaluation of the early immunogenicity cohorts, with the booster dose given at the earliest possible time.^5^

#### COV002 (UK)

COV002 is a continuing single-blind phase 2/3 study in the UK that began on May 28, 2020, and enrolled participants in 19 study sites in England, Wales, and Scotland. Enrolment particularly targeted individuals working in professions with high possible exposure to SARS-CoV-2, such as health and social care settings.

Two dosage groups were included in COV002: participants who received a low dose of the vaccine (2·2 × 10^10^ viral particles) as their first dose and were boosted with a standard dose (in the LD/SD group), and subsequent cohorts who were vaccinated with two standard-dose vaccines (SD/SD group). Initial dosing in COV002 was with a batch manufactured at a contract manufacturing organisation using chromatographic purification. During quality control of this second batch, differences were observed between the quantification methods (spectrophotometry and quantitative PCR [qPCR]) prioritised by different manufacturing sites. In consultation with the national regulator (Medicines and Healthcare products Regulatory Agency), we selected a dose of 5 × 10^10^ viral particles by spectrophotometer (2·2 × 10^10^ viral particles by qPCR), in order to be consistent with the use of spectrophotometry in the phase 1 study (COV001),^5^ and to ensure the dose was within a safe and immunogenic range according to measurements by both methods. A lower-than-anticipated reactogenicity profile was noted in the trial, and unexpected interference of an excipient with the spectrophotometry assay was identified. After review and approval by the regulator, it was concluded that the qPCR (low-dose) reading was more accurate and further doses were adjusted to the standard dose (5 × 10^10^ viral particles) using a qPCR assay. The protocol was amended on June 5, 2020, resulting in enrolment of two distinct groups with different dosing regimens with no pause in enrolment (version 6.0; appendix 2 p 330). A suite of assays has now been developed for characterisation of concentration (which confirmed the low and standard dosing), and future batches are all released with a specification dose of 3·5–6·5 × 10^10^ viral particles, and this was used for the booster doses in the efficacy analysis presented here.

The LD/SD cohort (aged 18–55 years) was enrolled over 11 days between May 31 and June 10, 2020. The SD/SD cohort (aged 18–55 years) was enrolled from June 9 to July 20, 2020. Subsequently, enrolment of older age cohorts began (from Aug 8, 2020, for participants aged 56–69 years and from Aug 13, 2020, for participants aged ≥70 years), all of whom were assigned to two standard doses (SD/SD cohort). Each site implemented the protocol amendment before changing from low-dose administration to standard-dose administration, and therefore there was no overlap in enrolment of participants in these cohorts.

The 18–55-year-old cohorts were originally planned as single-dose efficacy cohorts. However, the protocol was modified on July 20, 2020, to offer a second dose to the participants in these cohorts as a result of robust booster responses identified in the evaluation of the early immunogenicity cohorts (version 9.0; appendix 2 pp 331–332).^5^ Boosting began on Aug 3, 2020, resulting in a longer gap between prime and booster vaccines in these cohorts than for those aged 55–69 years and those aged 70 years or older, as these participants were enrolled into two-dose groups from the start.

Results for participants enrolled into immunogenicity subgroups have been previously published, including a small subset who received a low-dose boost.^6^ Full details are available in the study protocol (appendix 2 pp 184–342) and the procedures have been previously described.^6^

#### COV003 (Brazil)

COV003 is a continuing single-blind phase 3 study in Brazil that began on June 23, 2020. The focus of recruitment was targeted at those at high risk of exposure to the virus, including health-care workers at six sites across Brazil. Participants were aged 18 years or older, and this trial included individuals with stable pre-existing health conditions. All participants were offered two doses of the vaccine at a dose of 3·5–6·5 × 10^10^ viral particles with administration up to 12 weeks apart (target 4 weeks), following a protocol amendment on July 28, 2020, to include booster groups (version 4.0; appendix 2 pp 438–439). Full details are available in the study protocol (appendix 2 pp 343–441).

#### COV005 (South Africa)

COV005 is a continuing double-blind phase 1/2 study in South Africa in healthy adults aged 18–65 years living without HIV that began on June 28, 2020. An additional immunogenicity cohort of those living with HIV was also enrolled but are not included in this interim analysis. All participants were offered two doses of the vaccine at a dose of 3·5–6·5 × 10^10^ viral particles, with doses administered 4 weeks apart. A small subgroup of 44 participants received a half-dose vaccine (21 as their first dose and 23 as their second dose) as a result of variability in the release assay, before the adoption of new methods for characterisation of concentration. Adjustment in dose was discussed with and approved by the national regulator. Full details are available in the study protocol (appendix 2 pp 442–559).

A combined independent data safety monitoring board reviews safety data from all four trials on a regular basis.

### Randomisation and masking

In efficacy cohorts for all studies, participants were randomised 1:1 to receive ChAdOx1 nCoV-19 or a control product. In COV002, MenACWY was chosen as the control group vaccine to minimise the chance of accidental participant unmasking due to local or systemic reactions to the vaccine. COV003 used MenACWY as the control for the first dose and saline for the second dose. In COV005, participants randomly assigned to the control group were administered saline solution. Randomisation lists were prepared by the study statistician (MV) using block randomisation, stratified by study site and study group, and uploaded into to the secure web platform used for the study electronic case report form (REDCap version 9.5.22) for COV001, COV002, and COV003. In COV005, the randomisation list was held by the unmasked study pharmacist who prepared the vaccines for administration, with all other trial staff masked to group allocation. The trial staff administering the vaccine prepared vaccines out of sight of the participants and syringes were covered with an opaque material until ready for administration to ensure masking of participants.

### Procedures

The recombinant adenovirus for ChAdOx1 nCoV-19 was manufactured and vialed by Advent (Pomezia, Italy), and additional batches produced by COBRA Biologics (Keele, UK) and vialed by Symbiosis (Sterling, UK). Both were manufactured according to Good Manufacturing Practice and approved by the regulatory agency in the UK, the Medicines and Healthcare products Regulatory Agency.

Baseline assessments included review of inclusion and exclusion criteria, medical history, vital signs measurement, history-directed clinical examination, and collection of serum for SARS-CoV-2 serology.

Participants across all four trials were asked to contact the study site if they experienced specific symptoms associated with COVID-19 and received regular reminders to do so. Those who met symptomatic criteria had a clinical assessment, a swab taken for a nucleic acid amplification test (NAAT), and blood samples taken for safety and immunogenicity. In the UK and Brazil, the list of qualifying symptoms for swabbing included any one of the following: fever of at least 37·8°C, cough, shortness of breath, and anosmia or ageusia. In South Africa, the list of qualifying symptoms for swabbing was broader, and additionally included myalgia, chills, sore throat, headache, nasal congestion, diarrhoea, runny nose, fatigue, nausea, vomiting, and loss of appetite.

In all studies, if participants were tested outside of the trial, either in their workplace if a health-care worker or by private providers, these results were recorded and assessed by a masked independent endpoint review committee. The source of each swab was recorded plus the details of the test kit where available.

To test for asymptomatic infections, participants in COV002 in the UK were asked to provide a weekly self-administered nose and throat swab for NAAT testing from 1 week after first vaccination using kits provided by the UK Department of Health and Social Care (DHSC). Participants were given home test kits provided by the DHSC that included step-by-step instructions on how to do a self-swab and a link to a demonstration video. The site trial team provided support with logistics of packaging and returning test kits and tracking swab results to participants if required. Swabs were taken by participants in their homes and posted to dedicated DHSC testing laboratories for processing. Participants were directly informed of their results by text or email from the National Health Service (NHS). Swab results from participants in England and Wales were provided to the trial statistician on a daily basis by the NHS and matched to individuals based on personal identification data (name, date of birth, NHS number, and postcode). Swab results from participants in Scotland were unavailable to the study team at the time of the data cutoff for this analysis, but will be included in future analyses. Any swab results that were not able to be matched to a study participant using at least two pieces of personal data were not added to the study database.

In Brazil, there was no testing plan for asymptomatic infections. In South Africa, asymptomatic infections were detected from swabs obtained at study visits attended, but are not summarised here as there were only a small number of timepoints for detection of these cases.

All cases of COVID-19 were reviewed by two members of a masked independent clinical review team who assessed clinical details, including medical history, symptoms, adverse events, and swab results, and assigned severity scores according to the WHO clinical progression scale.^8^

For symptomatic participants in COV002 in the UK, weekly swabbing continued both before and after participants reported symptoms to the study site. Thus, a participant who reported symptoms and was clinically assessed might also have had additional swabs return positive results through the asymptomatic testing process for several weeks. In addition, due to the large number of health-care workers enrolled in these studies, some participants were tested according to their workplace testing policies and these results were also entered into the database for review by the masked endpoint evaluation committee. Further exploratory assessment of the length of time participants remained NAAT-positive, and the sources of information used for case detection will be done in future analyses.

### Outcomes

The primary objective was to evaluate the efficacy of ChAdOx1 nCoV-19 vaccine against NAAT-confirmed COVID-19. The primary outcome was virologically confirmed, symptomatic COVID-19, defined as a NAAT-positive swab combined with at least one qualifying symptom (fever ≥37·8°C, cough, shortness of breath, or anosmia or ageusia).

All participants were given an emergency 24-h telephone number to contact the on-call study physician for the duration of the study to report any illnesses. Serious adverse events were recorded throughout the study and reviewed at each study visit, with causality assigned by the site investigator. Events were clinically coded according to the Medical Dictionary for Regulatory Activities.

### Statistical analysis

The plan for assessing efficacy and safety for the ChAdOx1 nCoV-19 vaccine is based on global analyses using all available data from four studies with analysis pooled across the studies. A global statistical analysis plan for pooling study data was developed, after extensive advice from regulators, to prespecify the analyses that would contribute to the assessment of efficacy and this was signed off before any data analysis was conducted.

Randomised participants who received at least one dose in all studies are included in the safety analysis. However, each study had to meet prespecified criteria of having at least five cases eligible for inclusion in the primary outcome before a study was included in efficacy analyses. Neither COV001 or COV005 met these criteria and so are not included in the efficacy assessment for this interim analysis. It is expected that they will be included in efficacy assessments in future analyses once more cases have accrued. Additionally, only efficacy groups for COV002 (ie, groups 4, 6, 9, and 10) were included.

Vaccine efficacy was calculated as 1 – adjusted relative risk (ChAdOx1 nCoV-19 *vs* control groups) computed using a Poisson regression model with robust variance.^9^ The model contained terms for study, treatment group, and age group (18–55, 56–69, and ≥70 years) at randomisation. A reduced model that did not contain a term for age was used for models affected by convergence issues due to having few cases in the older age groups. The logarithm of the period at risk for the primary endpoint for pooled analysis was used as an offset variable in the model to adjust for volunteers having different follow-up times during which the events occurred. Cumulative incidence is presented using the Kaplan-Meier method.

The global pooled analysis plan allowed for an interim and a final efficacy analysis with α adjusted between the two analyses using a flexible gamma α-spending function, with significance being declared if the lower bound of the (1 - α)% CI is greater than 20%. Evidence of efficacy at the time of the interim analysis was not considered reason to stop the trials and all trials are continuing to accrue further data that will be included in future analyses.

The first interim analysis was planned to be triggered when at least 53 cases in participants who had received two standard-dose vaccines (SD/SD) had accrued that met the primary outcome definition more than 14 days after the second dose. This analysis provides 77% power for the 20% threshold to assume a true vaccine efficacy of 70%. Although the number of cases in the SD/SD cohort was used as the trigger for the interim analysis, the prespecified primary analysis included both SD/SD and LD/SD recipients. Due to the rapid increase in incidence of COVID-19 in the UK in October, more than 53 cases had accrued by the time of data lock for this interim analysis. There were 98 cases available for inclusion in the SD/SD cohorts. Based on these numbers, the α level calculated using the gamma α-spending function for this analysis is 4·16%.

Participants were excluded from the primary efficacy analysis if they were seropositive at baseline or had no baseline result. Other exclusions included those with NAAT-positive swabs within 14 days after the second vaccination, or those who discontinued from the study before having met the primary efficacy endpoint with a follow-up time of less than 15 days after the second vaccination. All reasons for exclusion are shown in appendix 1 (pp 5–8).

An analysis of efficacy after the first standard-dose vaccine in those who only received standard-dose vaccines was undertaken as a secondary analysis. Individuals were excluded if they had a NAAT-positive swab within 21 days after their first standard-dose vaccine.

Participants were analysed according to the vaccines they received. Sensitivity analyses included those who were seropositive at baseline and an intention-to-treat analysis. Safety analyses include all randomised participants who received at least one dose of any vaccine in any study.

Prespecified subgroup analyses are not included in this report but will be presented in future analyses when a larger dataset is available. However, in response to reviewer and editorial comments, a small number of exploratory subgroup comparisons has been included to explore differences in efficacy in the LD/SD and SD/SD groups and potential confounder variables. The LD/SD cohort in the UK comprised participants aged 18–55 years who received their second dose after a substantial gap. Age and the time difference between vaccines were therefore potential confounders and were explored further in subgroup analyses, restricted to those aged 18–55 years, those with more than 8 weeks’ interval between vaccine doses, and a comparison of those in the SD/SD cohort receiving vaccines at short (<6 weeks) or long (≥6 weeks) intervals. Subgroup comparisons were done by incorporating the treatment-by-subgroup interaction term in the model and reporting the p value for the interaction term.

Data analysis was done using R (version 3.6.1 or later). Robust Poisson models were fitted using the PROC GENMOD function in SAS (version 9.4). The α level for the analysis was calculated using the gsDesign function in R. The cutoff date for inclusion in the analysis was Nov 4, 2020, and the data lock date was Nov 21, 2020.

The four trials are registered at ISRCTN89951424 (COV003) and ClinicalTrials.gov, NCT04324606 (COV001), NCT04400838 (COV002), and NCT04444674 (COV005).

### Role of the funding source

AstraZeneca reviewed the data from the study and the final manuscript before submission, but the academic authors retained editorial control. All other funders of the study had no role in the study design, data collection, data analysis, data interpretation, or writing of the report. All authors had full access to all the data in the study and had final responsibility for the decision to submit for publication.

## Results

Between April 23 and Nov 4, 2020, 23 848 participants were recruited and vaccinated across the four studies: 1077 in COV001 (UK), 10 673 in COV002 (UK), 10 002 in COV003 (Brazil), and 2096 in COV005 (South Africa). 11 636 participants in COV002 and COV003 met the inclusion criteria for the primary analysis, 5807 of whom received two doses of ChAdOx1 nCoV-19 and 5829 of whom received two doses of control product. A trial profile and reasons for exclusion from the primary analysis are shown in appendix 1 (pp 5–7). Here, we provide safety data on 74 341 person-months of follow-up after first dose (median 3·4 months, IQR 1·3–4·8) and 29 060 person-months of follow-up after two doses (median 2·0, 1·3–2·3).

Of the participants in COV002 and COV003 included in the primary efficacy analyses, the majority were aged 18–55 years (6542 [86·7%] of 7548 in the UK and 3676 [89·9%] of 4088 in Brazil; table 1). Those aged 56 years or older were recruited later and contributed 12·2% of the total cohort in the current analysis (1006 [13·3%] in the UK and 412 [10·1%] in Brazil). 7045 (60·5%) participants were female. 6902 (91·4%) participants in the UK and 2723 (66·6%) participants in Brazil were white (table 1). Baseline participants of the safety population are shown in appendix 1 (pp 9–10).

**Table 1:** Baseline characteristics of participants included in the primary efficacy population, by study and dosing strategy

		**COV002 (UK; LD/SD; N=2741)**		**COV002 (UK; SD/SD; N=4807)**		**COV003 (Brazil; all SD/SD; N=4088)**	
		ChAdOx1 nCoV-19 (n=1367)	MenACWY (n=1374)	ChAdOx1 nCoV-19 (n=2377)	MenACWY (n=2430)	ChAdOx1 nCoV-19 (n=2063)	MenACWY plus saline (n=2025)
Age, years							
	18–55	1367 (100·0%)	1374 (100·0%)	1879 (79·0%)	1922 (79·1%)	1843 (89·3%)	1833 (90·5%)
	56–69	0	0	285 (12·0%)	293 (12·1%)	209 (10·1%)	187 (9·2%)
	≥70	0	0	213 (9·0%)	215 (8·8%)	11 (0·5%)	5 (0·2%)
Sex							
	Female	886 (64·8%)	927 (67·5%)	1378 (58·0%)	1437 (59·1%)	1261 (61·1%)	1156 (57·1%)
	Male	481 (35·2%)	447 (32·5%)	999 (42·0%)	993 (40·9%)	802 (38·9%)	869 (42·9%)
BMI, kg/m^2^		25·2 (22·8–28·7)	25·3 (22·7–28·8)	25·4 (22·9–28·7)	25·5 (22·9–29·1)	25·6 (22·8–29·1)	25·6 (23·1–29·0)
Ethnicity							
	White	1257 (92·0%)	1278 (93·0%)	2153 (90·6%)	2214 (91·1%)	1357 (65·8%)	1366 (67·5%)
	Black	6 (0·4%)	2 (0·1%)	17 (0·7%)	14 (0·6%)	230 (11·1%)	210 (10·4%)
	Asian	76 (5·6%)	59 (4·3%)	137 (5·8%)	138 (5·7%)	54 (2·6%)	53 (2·6%)
	Mixed	19 (1·4%)	22 (1·6%)	48 (2·0%)	42 (1·7%)	410 (19·9%)	386 (19·1%)
	Other	9 (0·7%)	13 (0·9%)	22 (0·9%)	22 (0·9%)	12 (0·6%)	10 (0·5%)
Health and social care setting workers		1236 (90·4%)	1253 (91·2%)	1441 (60·6%)	1513 (62·3%)	1833 (88·9%)	1775 (87·7%)
Comorbidities							
	Cardiovascular disease	104 (7·6%)	92 (6·7%)	264 (11·1%)	266 (10·9%)	271 (13·1%)	244 (12·0%)
	Respiratory disease	158 (11·6%)	176 (12·8%)	285 (12·0%)	316 (13·0%)	215 (10·4%)	210 (10·4%)
	Diabetes	18 (1·3%)	15 (1·1%)	58 (2·4%)	60 (2·5%)	59 (2·9%)	60 (3·0%)

Data are n (%) or median (IQR). The primary efficacy population (LD/SD and SD/SD) includes randomly assigned participants who were seronegative at baseline and received LD/SD or SD/SD or were in the corresponding control group, and remained on study more than 14 days after their second dose without having had a previous virologically confirmed severe acute respiratory syndrome coronavirus 2 infection. In addition, for groups in COV002, only efficacy groups (ie, groups 4, 6, 9, and 10) are included. LD/SD=low-dose prime plus standard-dose boost. SD/SD=two standard-dose vaccines given. MenACWY=meningococcal group A, C, W, and Y conjugate vaccine. BMI=body-mass index.

The timing of priming and booster vaccine administration varied between studies. As protocol amendments to add a booster dose took place when the trials were underway, and owing to the time taken to manufacture and release a new batch of vaccine, doses could not be administered at a 4-week interval. 1459 (53·2%) of 2741 participants in COV002 in the LD/SD group received a second dose at least 12 weeks after the first (median 84 days, IQR 77—91) and only 22 (0·8%) received a second dose within 8 weeks of the first. The median interval between doses for the SD/SD group in COV002 was 69 days (50–86). Conversely, the majority of participants in COV003 in the SD/SD group (2493 [61·0%] of 4088) received a second dose within 6 weeks of the first (median 36 days, 32–58; appendix 1 p 11).

A small proportion of participants were seropositive at baseline (138 [1·3%] of 10 673 in the UK and 235 [2·3%] of 10 002 in Brazil). Three participants seropositive at baseline had subsequent NAAT-positive swabs. One participant had an asymptomatic infection 3 weeks after a first dose of ChAdOx1 nCoV-19. Two other participants in the control group had symptomatic infections 8 weeks and 21 weeks after their baseline sample was taken.

There were 131 cases of symptomatic COVID-19 in LD/SD or SD/SD recipients who were eligible for inclusion in the primary efficacy analysis more than 14 days after the second dose of vaccine (table 2). There were 30 (0·5%) cases among 5807 participants in the vaccine arm and 101 (1·7%) cases among 5829 participants in the control group, resulting in vaccine efficacy of 70·4% (95·8% CI 54·8–80·6; table 2; figure). In participants who received two standard-dose vaccines, vaccine efficacy was 62·1% (95% CI 41·0–75·7), whereas in those who received a low dose as their first dose of vaccine, efficacy was higher at 90·0% (67·4–97·0; p_interaction_=0·010; table 2; appendix 1 pp 12–13).

**Table 2:** Efficacy against SARS-CoV-2 more than 14 days after a second dose of ChAdOx1 nCoV-19 vaccine in the primary efficacy population

			**Total number of cases**	**ChAdOx1 nCoV-19**		**Control**		Vaccine efficacy (CI*)
				n/N (%)	Incidence rate per 1000 person-years (person-days of follow-up)	n/N (%)	Incidence rate per 1000 person-years (person-days of follow-up)	
All LD/SD and SD/SD recipients			131	30/5807 (0·5%)	44·1 (248 299)	101/5829 (1·7%)	149·2 (247 228)	70·4% (54·8 to 80·6)†
	COV002 (UK)		86	18/3744 (0·5%)	38·6 (170 369)	68/3804 (1·8%)	145·7 (170 448)	73·5% (55·5 to 84·2)
		LD/SD recipients	33	3/1367 (0·2%)	14·9 (73 313)	30/1374 (2·2%)	150·2 (72 949)	90·0% (67·4 to 97·0)§§
		SD/SD recipients	53	15/2377 (0·6%)	56·4 (97 056)	38/2430 (1·6%)	142·4 (97 499)	60·3% (28·0 to 78·2)
	COV003 (Brazil; all SD/SD)		45	12/2063 (0·6%)	56·2 (77 930)	33/2025 (1·6%)	157·0 (76 780)	64·2% (30·7 to 81·5)‡
	All SD/SD recipients		98	27/4440 (0·6%)	56·4 (174 986)	71/4455 (1·6%)	148·8 (174 279)	62·1% (41·0 to 75·7)
Other non-primary symptomatic COVID-19 disease¶			18	7/5807 (0·1%)	10·3 (248 299)	11/5829 (0·2%)	16·3 (247 228)	36·4% (−63·8 to 75·3)‡
Any symptomatic COVID-19 disease			149	37/5807 (0·6%)	54·4 (248 299)	112/5829 (1·9%)	165·5 (247 228)	67·1% (52·3 to 77·3)
Asymptomatic or symptoms unknown (COV002)			69	29/3288 (0·9%)	69·8 (151 673)	40/3350 (1·2%)	96·0 (152 138)	27·3% (−17·2 to 54·9)
	LD/SD recipients		24	7/1120 (0·6%)	41·4 (61 782)	17/1127 (1·5%)	100·6 (61 730)	58·9% (1·0 to 82·9)‡
	SD/SD recipients		45	22/2168 (1·0%)	89·4 (89 891)	23/2223 (1·0%)	92·9 (90 408)	3·8% (−72·4 to 46·3)
Any NAAT-positive swab			221	68/5807 (1·2%)	100·0 (248 299)	153/5829 (2·6%)	226·0 (247 228)	55·7% (41·1 to 66·7)

Vaccine efficacy was calculated from the robust Poisson model. The primary efficacy population (LD/SD and SD/SD) includes randomly assigned participants who were seronegative at baseline and received LD/SD or SD/SD or were in a corresponding control group, and remained on study more than 14 days after their second dose without having had a previous virologically confirmed SARS-CoV-2 infection. In addition, for groups in COV002, only efficacy groups (ie, groups 4, 6, 9, and 10) are included. SARS-CoV-2=severe acute respiratory syndrome coronavirus 2. LD/SD=low-dose prime plus standard-dose boost. SD/SD=two standard-dose vaccines given. NAAT=nucleic acid amplification test.

* CIs are 95% unless indicated otherwise.

† 95·8% CI used for primary analysis.

‡ Vaccine efficacy calculated from a reduced robust Poisson model that was not adjusted for age. All other models included an adjustment for age.

§ p value for interaction term comparing LD/SD with SD/SD is p=0·010.

¶ Other non-primary symptomatic COVID-19 disease includes cases who have symptoms other than the five main symptoms that are required for inclusion in the primary analysis (eg, a participant who has diarrhoea and malaise but no fever, cough, shortness of breath, anosmia, or ageusia).

**Figure:**
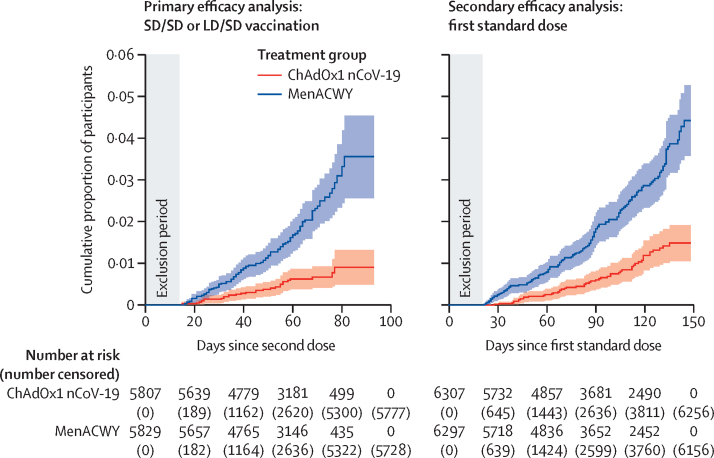
Kaplan-Meier cumulative incidence of primary symptomatic, NAAT-positive COVID-19 Cumulative incidence of symptomatic COVID-19 after two doses (left) or after first standard dose in participants receiving only standard-dose vaccines (right). Grey shaded areas show the exclusion period after each dose in which cases were excluded from the analysis. Blue and red shaded areas show 95% CIs. LD/SD=low-dose prime plus standard-dose boost. MenACWY=meningococcal group A, C, W, and Y conjugate vaccine. NAAT=nucleic acid amplification test. SD/SD=two standard-dose vaccines given.

In England and Wales, 129 529 weekly self-swabs were processed by the DHSC, of which 126 324 (97·5%) were matched to study participants. There were 435 positive swabs, of which 354 (81·4%) were matched. Symptoms in these participants were not routinely assessed as swabs were done at home and sent for testing through the post. Asymptomatic infections or those with unreported symptoms were detected in 69 participants (table 2). Vaccine efficacy in the 24 LD/SD recipients was 58·9% (95% CI 1·0 to 82·9), whereas it was 3·8% (−72·4 to 46·3) in the 45 participants receiving SD/SD (table 2).

Results from sensitivity analyses, including participants who were seropositive at baseline and by intention to treat, were very similar to main results (data not shown).

Results from the subgroup comparisons presented in this analysis were similar to overall results (table 3). In the SD/SD UK cohort who were aged 18–55 years, 49 cases were available for inclusion in the analysis and vaccine efficacy was 59·3% (95% CI 25·1 to 77·9; p_interaction_=0·019; table 3). When further restricted to those who received their vaccines more than 8 weeks apart, 33 cases were included in the SD/SD analysis and vaccine efficacy was 65·6% (24·5 to 84·4; p_interaction_=0·082; table 3; appendix 1 pp 12–13). In the SD/SD cohorts in the UK and Brazil, vaccine efficacy was similar when analysed in subgroups according to time between vaccines, at 53·4% (−2·5 to 78·8) in participants with less than 6 weeks’ interval between doses and 65·4% (41·1 to 79·6) in participants with at least 6 weeks’ interval (p_interaction_=0·56; table 3).

**Table 3:** Subgroup comparisons of efficacy against SARS-CoV-2 more than 14 days after a second dose of ChAdOx1 nCoV-19 vaccine in the primary efficacy population

		**Total number of cases**	**ChAdOx1 nCoV-19**	**Control**	**Vaccine efficacy (95% CI)**	**p value for interaction**
COV002 (UK), age 18–55 years*		..	..	..	..	0·019
	LD/SD recipients	33	3/1367 (0·2%)	30/1374 (2·2%)	90·0% (67·3 to 97·0)	..
	SD/SD recipients	49	14/1879 (0·7%)	35/1922 (1·8%)	59·3% (25·1 to 77·9)	..
COV002 (UK), age 18–55 years with >8 weeks' interval between vaccine doses*		..	..	..	..	0·082
	LD/SD recipients	33	3/1357 (0·2%)	30/1362 (2·2%)	90·0% (67·3 to 97·0)	..
	SD/SD recipients	34	8/1407 (0·6%)	26/1512 (1·7%)	65·6% (24·5 to 84·4)	..
All SD/SD (UK and Brazil)†		..	..	..	..	0·557
	<6 weeks' interval between vaccine doses	28	9/1702 (0·5%)	19/1698 (1·1%)	53·4% (−2·5 to 78·8)	..
	≥6 weeks' interval between vaccine doses	70	18/2738 (0·7%)	52/2757 (1·9%)	65·4% (41·1 to 79·6)	..

Cohorts are all subsets of the primary efficacy population. SARS-CoV-2=severe acute respiratory syndrome coronavirus 2. LD/SD=low-dose prime plus standard-dose boost. SD/SD=two standard-dose vaccines given. BMI=body-mass index.

* Models adjusted for BMI (<30 *vs* ≥30 kg/m^2^), health-care worker status (yes *vs* no), and ethnicity (white *vs* non-white).

† Model adjusted for BMI (<30 *vs* ≥30 kg/m^2^), health-care worker status (yes *vs* no), ethnicity (white *vs* non-white), age (<56 years *vs* ≥56 years), and study (COV002 *vs* COV003).

For our secondary analysis of cases occurring more than 21 days after the first standard dose in participants who received only standard doses, there were 192 included cases with a vaccine efficacy of 64·1% (95% CI 50·5–73·9; table 4; figure)

**Table 4:** Efficacy against SARS-CoV-2 more than 21 days after the first standard dose in seronegative participants who received only standard doses

	**Total number of cases**	**ChAdOx1 nCoV-19**		**Control**		**Vaccine efficacy (95% CI)**
		n/N (%)	Incidence per 1000 person-years (person-days of follow-up)	n/N (%)	Incidence per 1000 person-years (person-days of follow-up)	
COV002 (UK)	90	28/3060 (0·9%)	35·4 (288 955)	62/3064 (2·0%)	78·5 (288 395)	55·0% (29·7 to 71·1)
COV003 (Brazil)	102	23/3247 (0·7%)	46·7 (179 743)	79/3233 (2·4%)	162·4 (177 693)	71·2% (54·2 to 81·9)
Primary symptomatic COVID-19*	192	51/6307 (0·8%)	39·7 (468 698)	141/6297 (2·2%)	110·5 (466 088)	64·1% (50·5 to 73·9)
Other non-primary symptomatic COVID-19†	21	12/6307 (0·2%)	9·4 (468 698)	9/6297 (0·1%)	7·1 (466 088)	−32·8% (−214·8 to 44·0)‡
Any symptomatic COVID-19	213	63/6307 (1·0%)	49·1 (468 698)	150/6297 (2·4%)	117·5 (466 088)	58·3% (44·0 to 68·9)
Asymptomatic or symptoms unknown (COV002)	71	34/2751 (1·2%)	46·8 (265 142)	37/2760 (1·3%)	51·0 (264 994)	7·8% (−46·7 to 42·1)
Any NAAT-positive swab	291	102/6307 (1·6%)	79·5 (468 698)	189/6297 (3·0%)	148·1 (466 088)	46·3% (31·8 to 57·8)

Vaccine efficacy was calculated from the robust Poisson model. The first-standard-dose efficacy population includes participants seronegative at baseline who received only standard dose vaccines or were in the corresponding control group, and remained on study 22 days after their first dose without having had a previous virologically confirmed SARS-CoV-2 infection. In addition, for groups in COV002, only efficacy groups (ie, groups 4, 6, 9, and 10) are included. SARS-CoV-2=severe acute respiratory syndrome coronavirus 2. NAAT=nucleic acid amplification test.

* NAAT-positive swab plus at least one of cough, shortness of breath, fever higher than 37·8°C, anosmia, or ageusia.

† Other non-primary symptomatic COVID-19 disease includes cases that have symptoms other than the five main symptoms required for inclusion in the primary analysis (eg, a participant who has diarrhoea and malaise but no fever, cough, shortness of breath, anosmia, or ageusia).

‡ Vaccine efficacy was calculated from a reduced robust Poisson model (excluding the age group category due to the full model failing to converge). Participants with a low-dose prime were excluded.

More than 21 days after their first dose, ten participants were hospitalised due to COVID-19 (defined as WHO clinical progression score ≥4), two of whom were assessed as having severe COVID-19 (WHO score ≥6), including one fatal case. All ten cases were in the control group (table 5).

**Table 5:** Hospitalisation for COVID-19 and severe COVID-19 in the safety population

	**ChAdOx1 nCoV-19 (n=12 021)**	**MenACWY or saline control (n=11 724)**
**Hospitalisation (WHO clinical progression score ≥4)**		
≤21 days after the first dose	2*	6
>21 days after the first dose and ≤14 days after the second dose	0	5
>14 days after the second dose	0	5
**Severe COVID-19 (WHO clinical progression score ≥6)**		
≤21 days after the first dose	0	0
>21 days after the first dose and ≤14 days after the second dose	0	1
>14 days after the second dose	0	1

The safety population includes all randomisation participants who received at least one dose of vaccine. Severe COVID-19 (WHO score ≥6) is a subset of hospitalisations (WHO score ≥4). Cases were eligible for inclusion in efficacy if the first symptom or first NAAT-positive result was on or before the data cutoff date (Nov 4, 2020). Two cases appear in this table that do not appear in the table for serious adverse events in appendix 1 (pp 15–20) as the adverse event reporting date was after the data cutoff date. MenACWY=meningococcal group A, C, W, and Y conjugate vaccine. NAAT=nucleic acid amplification test.

* One case on the day of the first vaccination and one case 10 days after the first dose.

Five cases included in the primary analysis occurred in those participants older than 55 years of age. Vaccine efficacy in older age groups could not be assessed but will be determined, if sufficient data are available, in a future analysis after more cases have accrued.

Across all four studies, the vaccine had a good safety profile with serious adverse events and adverse events of special interest balanced across the study arms. Serious adverse events occurred in 168 participants, 79 of whom received ChAdOx1 nCoV-19 and 89 of whom received MenACWY or saline control (appendix 1 pp 15–18). There were 175 events (84 in the ChAdOx1 nCoV-19 group and 91 in the control group), three of which were considered possibly related to either the experimental or a control vaccine. A case of haemolytic anaemia in the control group in the UK phase 1/2 study occurring 10 days after MenACWY vaccine was considered possibly related to the intervention and has been previously described.^5^ A case of transverse myelitis was reported 14 days after ChAdOx1 nCoV-19 booster vaccination as being possibly related to vaccination, with the independent neurological committee considering the most likely diagnosis to be of an idiopathic, short segment, spinal cord demyelination. A potentially vaccine-related serious adverse event was reported 2 days after vaccination in South Africa in an individual who recorded fever higher than 40°C, but who recovered rapidly without an alternative diagnosis and was not admitted to hospital. The participant remains masked to group allocation, continues in the trial, and received a second dose of the allocated vaccine without a similar reaction.

There were two additional cases of transverse myelitis that were originally reported as potentially related but later determined to be unlikely to be related to vaccination by an independent committee of neurological experts. One case that occurred 10 days after a first vaccination with ChAdOx1 nCoV-19 was initially assessed as possibly related, but later considered unlikely to be related by the site investigator when further investigation revealed pre-existing, but previously unrecognised, multiple sclerosis. The second case was reported 68 days after MenACWY vaccination. While considered possibly related by the site investigator at the time of reporting, an independent panel of neurological experts considered this to be unlikely. All trial participants have recovered, or are in a stable or improving condition.

There were four non-COVID-19 deaths reported across the studies (three in the control arm and one in the ChAdOx1 nCoV-19 arm) that were all considered unrelated to the vaccine, with cause of death assessed as road traffic accident, blunt force trauma, homicide, and fungal pneumonia.

## Discussion

Here, we present the first interim safety and efficacy data for a viral vector coronavirus vaccine, ChAdOx1 nCoV-19, evaluated in four trials across three continents, showing significant vaccine efficacy of 70·4% after two doses and protection of 64·1% after at least one standard dose, against symptomatic disease, with no safety concerns.

The prespecified analysis population, which was determined following feedback from national and international regulators before unblinding of the study, included a pooled analysis from several countries to improve generalisability, and inclusion of two dose subgroups within the UK trial. This pooling strategy was authorised by the chief investigator (AJP) and study statistician (MV), with no concerns about pooling different control groups, and was accepted by regulators involved in the discussions. There had been initial concern that the LD/SD regimen might have lower efficacy than SD/SD, and the regulatory authority acceptance of the inclusion of the two trial regimens (LD/SD and SD/SD) in analysis was based on the observation that these regimens generated similar levels of binding antibody, and would therefore increase the sample size available for analysis without compromising efficacy. The discussion about pooling and inclusion of LD/SD was made at a time when disease rates were low in the UK and, in the face of the pandemic, it was agreed that pooling could provide the earliest possible read on efficacy that could contribute to public health.

No previous trials have been published on the efficacy of a viral-vectored coronavirus vaccine and so this study provides the first peer-reviewed evidence that induction of immune responses against spike protein using viral vectors provides protection against the disease in humans, as has been seen in animal models.

In participants who received two standard doses, efficacy against primary symptomatic COVID-19 was consistent in both the UK (60·3% efficacy) and Brazil (64·2% efficacy), indicating these results are generalisable across two diverse settings with different timings for the booster dose (with most participants in the UK receiving the booster dose more than 12 weeks after the first dose and most participants in Brazil receiving their second dose within 6 weeks of the first). Exploratory subgroup analyses included at the request of reviewers and editors also showed no significant difference in efficacy estimates when comparing those with a short time window between doses (<6 weeks) and those with longer (≥6 weeks), although further detailed exploration of the timing of doses might be warranted.

Efficacy of 90·0% seen in those who received a low dose as prime in the UK was intriguingly high compared with the other findings in the study. Although there is a possibility that chance might play a part in such divergent results, a similar contrast in efficacy between the LD/SD and SD/SD recipients with asymptomatic infections provides support for the observation (58·9% [95% CI 1·0 to 82·9] *vs* 3·8% [−72·4 to 46·3]). Exploratory subgroup analyses, included at the request of reviewers and editors, that were restricted to participants aged 18–55 years, or aligned (>8 weeks) intervals between doses, showed similar findings. Use of a low dose for priming could provide substantially more vaccine for distribution at a time of constrained supply, and these data imply that this would not compromise protection. While a vaccine that could prevent COVID-19 would have a substantial public health benefit, prevention of asymptomatic infection could reduce viral transmission and protect those with underlying health conditions who do not respond to vaccination, those who cannot be vaccinated for health reasons, and those who will not or cannot access a vaccine, providing wider benefit for society. However, the wide CIs around our estimates show that further data are needed to confirm these preliminary findings, which will be done in future analyses of the data accruing in these ongoing trials.

Similar results have been seen for other vaccines where a reduced number or type of priming dose in infancy can lead to higher responses to a booster vaccine.^10^ Further work is needed to determine the mechanism of the increased efficacy with a LD/SD regimen, which might be due to higher levels of neutralising antibody, lower levels of anti-vector immunity with lower vector-derived antigen content of the first dose, or differential antibody functionality or cellular immunity, including altered avidity or immunodominance.

Other coronavirus vaccine developers have released preliminary high-level results in public statements, including more than 90% efficacy reported for the lipid nanoparticle mRNA vaccine BNT162b2,^11^ 92% efficacy for the Sputnik V vaccine (developed at the National Research Centre for Epidemiology and Microbiology),^12^ and 94·5% for the Moderna lipid nanoparticle mRNA-1273 vaccine.^13^ The possibility that more than one efficacious vaccine against COVID-19 might be approved for use in the near future is encouraging. However, control of pandemic coronavirus will only be achieved if the licensure, manufacturing, and distribution of these vaccines can be achieved at an unprecedented scale and vaccination is rolled out to all those who are vulnerable.

The US Food and Drug Administration's guidelines indicate that they would license a vaccine against the pandemic virus that showed at least 50% efficacy^14^ and WHO have indicated a minimum efficacy of 50% in its target product profile.^15^ A modelling study found that a vaccine with efficacy of 60–80% could allow reduction in physical distancing measures, but this would still require high coverage.^16^ The findings here indicate that the efficacy of ChAdOx1 nCoV-19 exceeds these thresholds and has the potential to have a public health impact.

Much consideration has been given to the statistical confidence in vaccine efficacy estimates, given the size of the global population who might be vaccinated. To ensure that point estimates of efficacy in clinical trials are sufficiently robust, some regulatory authorities consider that the lower bound of the CI for efficacy should be higher than 20% (personal communication), with other authorities more stringent and anticipating a lower bound of 30% for licensure.^14^ Here, we present data that exceed both these thresholds in the pooled analysis, which we had agreed with regulators before unblinding of the study, and also meet the thresholds set in the individual analyses of trials by country and by study arm.

We designed our studies early in the pandemic and fixed our primary symptomatic disease endpoint on the basis of expert analysis and guidelines from Public Health England and WHO as the first wave of disease spread around the world, although these have now been substantially updated.^17^, ^18^ We have used a restricted definition of symptomatic disease, since many other symptoms that are associated with COVID-19 disease are non-specific. Since endpoints in protocols for different vaccines are not well aligned, we recognise that it will be difficult to compare efficacy across programmes. However, we have also included hospital admissions and severe disease as an endpoint in the current study, which might be easier to assess in comparison with other vaccines, and found that in the ten cases available for analysis more than 21 days after the first dose, there was complete protection against hospitalisation for COVID-19.

While the data presented here show that ChAdOx1 nCov-19 is efficacious against symptomatic disease, with most cases accruing in adults younger than 55 years of age so far, an important public health consideration is the morbidity and mortality of the disease in an older adult population and thus the potential efficacy in this age group. We have reported immunogenicity data showing similar immune responses following vaccination with two doses of ChAdOx1 nCov-19 in older adults, including those older than 70 years of age, when compared with those younger than 55 years.^6^ As older age groups were recruited later than younger age groups, there has been less time for cases to accrue and as a result, efficacy data in these cohorts are currently limited by the small number of cases, but additional data will be available in future analyses.

These trials, conducted on three different continents, enrolled geographically and ethnically diverse populations. Severe COVID-19 has been seen to disproportionately affect people of non-white ethnicity, as well as those who are male, overweight, and the elderly.^19^, ^20^

In our studies, the demographic characteristics of those enrolled varied between countries. In the UK, the enrolled population was predominantly white and, in younger age groups, included more female participants due to the focus on enrolment of health-care workers. This is a typically lower risk population for severe COVID-19. The demographic profile combined with the weekly self-swabbing for asymptomatic infection in the UK results in a milder case-severity profile. In Brazil, there was a larger proportion of non-white ethnicities, and again the majority of those enrolled were health-care workers.

We have previously reported on the local and systemic reactogenicity of ChAdOx1 nCoV-19 and shown that it is tolerated and that the side-effects are less both in intensity and number in older adults, with lower doses, and after the second dose. Although there were many serious adverse events reported in the study in view of the size and health status of the population included, there was no pattern of these events that provided a safety signal in the study. Three cases of transverse myelitis were initially reported as suspected unexpected serious adverse reactions, with two in the ChAdOx1 nCoV-19 vaccine study arm, triggering a study pause for careful review in each case. Independent clinical review of these cases has indicated that one in the experimental group and one in the control group are unlikely to be related to study interventions, but a relationship remained possible in the third case. Careful monitoring of safety, including neurological events, continues in the trials. All safety data will be provided to regulators for review.

In this interim analysis, we have not been able to assess duration of protection, since the first trials were initiated in April, 2020, such that all disease episodes have accrued within 6 months of the first dose being administered. Further evidence will be required to determine duration of protection and the need for additional booster doses of vaccine.

The results presented in this Article constitute the key findings from the first interim analysis, which are provided for rapid review by the public and policy makers. In future analyses with additional data included as they accrue, we will investigate differences in key subgroups such as older cohorts, ethnicity, dose regimen, and timing of booster vaccines, and we will search for correlates of protection.

Until widespread immunity halts the spread of SARS-CoV-2, physical distancing measures and novel therapies are needed to control COVID-19. In the meantime, an efficacious vaccine has the potential to have a major impact on the pandemic if used in populations at risk of severe disease. Here, we have shown for the first time that a viral vector vaccine, ChAdOx1 nCoV-19, is efficacious and could contribute to control of the disease in this pandemic.

**This online publication has been corrected. The corrected version first appeared at thelancet.com on January 7, 2021**

## Data sharing

Anonymised participant data will be made available when the trials are complete, upon requests directed to the corresponding author. Proposals will be reviewed and approved by the sponsor, investigator, and collaborators on the basis of scientific merit. After approval of a proposal, data can be shared through a secure online platform after signing a data access agreement. All data will be made available for a minimum of 5 years from the end of the trial.
