# Supplementary material for: Safety and efficacy of the ChAdOx1 nCoV-19 vaccine (AZD1222) against SARS-CoV-2: an interim analysis of four randomised controlled trials in Brazil, South Africa, and the UK
Source: Lancet. 2021 Jan 9;397(10269):99–111. doi: 10.1016/S0140-6736(20)32661-1 (PMC7723445; doi:10.1016/S0140-6736(20)32661-1)
Supplement: Supplementary appendix [file mmc1.pdf]

THE LANCET

Supplementary appendix

This appendix formed part of the original submission and has been peer reviewed. We post it as supplied by the authors.

This online publication has been corrected. The corrected version first appeared at thelancet.com on January 7, 2020.

Supplement to: Voysey M, Clemens SAC, Madhi SA, et al. Safety and efficacy of the ChAdOx1 nCoV-19 vaccine (AZD1222) against SARS-CoV-2: an interim analysis of four randomised controlled trials in Brazil, South Africa, and the UK. *Lancet* 2020; published online Dec 8. http://dx.doi.org/10.1016/S0140-6736(20)32661-1.

Supplementary Information

Supplementary Laboratory Methods 2

Table S1 Summary of trials included in the analysis 3

Figure S1 CONSORT participant flow diagram 5

Table S2 Participant disposition for primary analysis in COV002 and COV003 6

Table S3 Participant disposition for secondary analysis in COV002 and COV003 8

Table S4 Baseline characteristics of participants included in the any dose for safety population* 9

Table S5 Timing of vaccine administration in those included in the primary analysis* 11

Figure S2 Kaplan-Meier cumulative incidence of primary symptomatic COVID-19 in LD/SD recipients in the UK, and in SD/SD recipients in the UK and Brazil 12

Figure S3 Kaplan-Meier cumulative incidence of primary symptomatic COVID-19 in SD/SD recipients in the UK, and in SD/SD recipients in the Brazil 13

Table S6 Serious Adverse Events by MedDRA System Organ Class and Preferred Term at any time during the study, in randomised participants who received at least one dose of vaccine 15

Table S7 Adverse Events of Special Interest by Special Interest Category and Preferred Term in randomised participants who received at least one dose of vaccine (Any Dose for Safety Population) 19

Oxford Vaccine Trial Group Authorship 21

Acknowledgements 45

Supplementary Laboratory Methods

Serum samples were measured at baseline in a validated serological assay using the nucleocapsid antigen of SARS-COV-2 and run at PPD Central Labs (Zaventum, Belgium and Highland Heights, KY, USA). The Roche Elecsys Anti-SARS-CoV-2 serology test is an electroluminescence immunoassay-based modality that allows for the qualitative detection of IgG reactive to the SARS-CoV-2 nucleoprotein in human sera. Further details available at: https://www.accessdata.fda.gov/cdrh_docs/presentations/maf/maf3358-a001.pdf

Table S1 Summary of trials included in the analysis

Study	COV001	COV002	COV003	COV005
Country	United Kingdom	United Kingdom	Brazil	South Africa
Blinding	Single Blind	Single Blind	Single Blind	Double Blind
Control Group	MenACWY	MenACWY	MenACWY (first dose)<br>Saline (second dose)	Saline Placebo
Funding	UK Research and Innovation, Coalition for Epidemic Preparedness Innovations, National Institute for Health Research (NIHR), NIHR Oxford Biomedical Research Centre, Thames Valley and South Midland's NIHR Clinical Research Network	United Kingdom National Institute for Health Research (NIHR), Coalition for Epidemic Preparedness Innovations, NIHR Oxford Biomedical Research Centre, Thames Valley and South Midlands NIHR Clinical Research Network, and AstraZeneca.	University of Oxford, Fundação Lemann, Fundação Brava, Fundação Telles, Instituto D'or de Ensino e Pesquisa and AstraZeneca Brasil.	UK Research and Innovation (For Vaccine supply only), The Bill and Melinda Gates Foundation and South African Medical Research Council
Study Sites	1. Centre for Clinical Vaccinology and Tropical Medicine, University of Oxford;<br>2. NIHR Southampton Clinical Research Facility, University Hospital Southampton NHS Foundation Trust, Southampton;<br>3. Clinical Research Facility, Imperial College London;<br>4. St Georges University of London and	6. University Hospitals Birmingham NHS Foundation Trust<br>7. University Hospitals Bristol and Weston NHS Foundation Trust<br>8. NIHR Cambridge Clinical Research Facility<br>9. Aneurin Bevan Local Health Board Headquarters, Wales<br>10. Lothian NHS Board<br>11. Greater Glasgow and Clyde NHS Board<br>12. Guy's and St Thomas' NHS Foundation Trust<br>13. Hull University Teaching Hospitals NHS Trust	1. Centro de Referência para Imunobiológicos Especiais, Universidade Federal de São Paulo - São Paulo, SP<br>2. Instituto D'Or de Pesquisa e Ensino – Rio de Janeiro, RJ<br>3. Instituto D'Or de Pesquisa e Ensino – Salvador, BA .<br>4. Universidade Federal de Santa Maria - Santa Maria, RS<br>5. Hospital de Clínicas de Porto Alegre,	1. Respiratory and Meningeal Pathogens Research Unit - Johannesburg<br>2. Setshaba Research Centre (SRC) – Gauteng<br>3. Wits RHI Shandukani Research Centre – Johannesburg<br>4. Perinatal HIV Research Unit - Kliptown.<br>5. Family Centre for Research with Ubuntu

	5. University Hospital NHS Foundation Trust; and University Hospitals Bristol and Weston NHS Foundation Trust).	14. NIHR Imperial Clinical Research Facility Imperial College London<br>15. Liverpool School of Tropical Medicine<br>16. North Bristol NHS Trust<br>17. NIHR Newcastle Clinical Research Facility, The Newcastle upon Tyne Hospitals NHS Foundation Trust<br>18. Northwick Park Hospital<br>19. University of Nottingham Health Service<br>20. Oxford University Hospitals NHS Foundation Trust<br>21. Sheffield Teaching Hospitals NHS Foundation Trust University Hospital Southampton NHS Foundation Trust<br>22. St Georges University Hospital NHS Foundation Trust<br>23. University College London Hospitals NHS Foundation Trust	Universidade Federal do Rio Grande do Sul - Porto Alegre, RS<br>6. Centro de Pesquisas Clinicas – Natal, RN	(FAMCRU) – Cape Town<br>6. Soweto Clinical Trials Centre (SCTC)<br>7. University of Cape Town Lung Institute and Centre for Lung Infection and Immunity (CLII) – Cape Town<br>8. Soweto Clinical Trials Centre (SCTC) - Johannesburg

Figure S1 CONSORT participant flow diagram

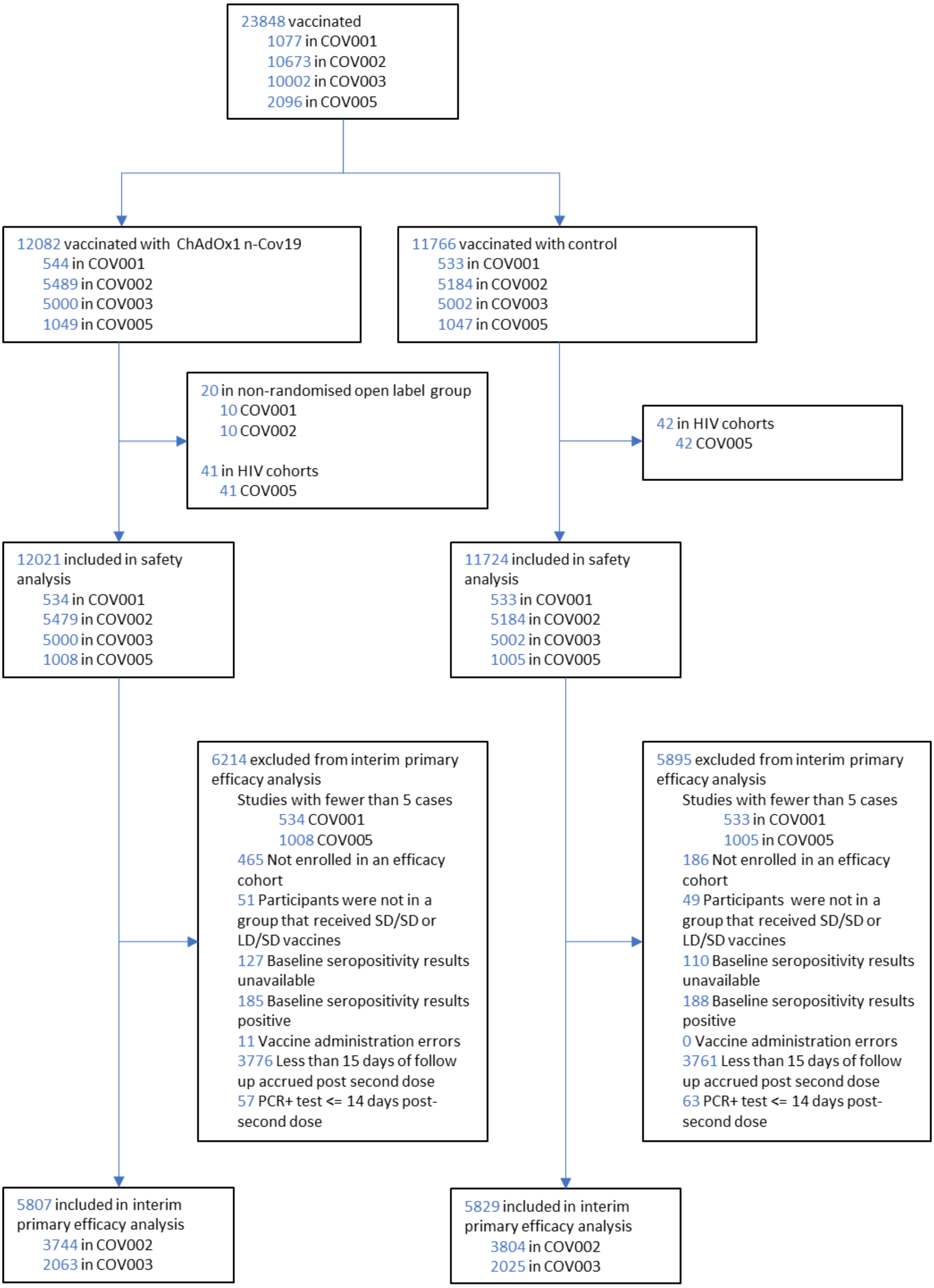

Table S2 Participant disposition for primary analysis in COV002 and COV003

**Exclusion from primary analysis**	**COV002**				**COV003**	
	**LD/SD**		**SD/SD**			
	**ChAdOx1 nCoV-19***	**Control***	**ChAdOx1 nCoV-19***	**Control***	**ChAdOx1 nCoV-19***	**Control***
**N Total number vaccinated with at least one dose**	**10673**				**10002**	
1. Non-randomised groups⁑	10				0	
2. Not enrolled in an efficacy cohort	465 (ChAdOx1 nCoV-19)<br>186 (Control)				0	
3. Participants were not in a group that received SD/SD, LD/SD vaccines‡	51 (ChAdOx1 nCoV-19)<br>49 (Control)				0	
4. Baseline seropositivity results unavailable ♯	4	4	27	23	96	83
5. Baseline seropositivity results positive	19	17	53	49	113	122
6. Vaccine administration errors	6 (LDSD:1, SDSD:5)†				5†	
7. Less than or equal to 14 days of follow up accrued post second dose	264	261	816	766	2696	2734
7.1 Chose not to receive a second dose	145	119	152	134	0	0
7.2 Yet to reach 28 days post first dose, so not yet eligible for second dose	0	0	248	247	1807	1823

7.3 All others yet to receive second dose	113	138	231	197	509	562
7.4 Received a second dose but did not reach the 14 days post boost time point	6	4	183	188	380	348
7.5 The participant withdrew early	0	0	2	0	0	1
8. PCR+ test <= 14 days post-second dose (by arm)	11	5	19	20	27	38
**N included in efficacy analysis**	**1367**	**1374**	**2377**	**2430**	**2063**	**2025**

† These participants received two different vaccines and were excluded. One SDSD participant in COV002 was randomised to control but received two doses of ChAdOx1 nCoV-19. Two participants in COV003 were randomised to control but received two doses of ChAdOx1 nCoV-19, and three participants were randomised to ChAdOx1 nCoV-19 but received two doses of control vaccine. These participants who, in error, received two doses of the same vaccine were included in the primary analysis under the vaccine they received. ⁑COV002 included a non-randomised open-label immunogenicity group of participants who had received a prior non-COVID ChAdOx1 vaccine and these participants are excluded.‡ This cohort received LD/LD vaccines.
♯Results were unavailable from some samples as the quantity of sample was not sufficient for the assay.

Table S3 Participant disposition for secondary analysis in COV002 and COV003

	COV002		COV003	
	**ChAdOx1 nCoV-19***	**Control***	**ChAdOx1 nCoV-19***	**Control***
**N Total number vaccinated with at least one dose**	**10673**		**10002**	
1. Non-randomised groups⁑	10		0	
2. Not enrolled in an efficacy cohort	465	186	0	0
3. Participants were not randomised to receive SD vaccines as the first dose	1715	1712	0	0
4. Baseline seropositivity results unavailable ♯	27	23	96	83
5. Baseline seropositivity results positive	53	49	113	122
6. Vaccine administration errors	1†		2†	
7. Less than or equal to 21 days of follow up accrued post first dose	150	149	1508	1526
7.1 Did not reach the 21 days post-prime time point	150	146	1508	1526
7.2 The participant withdrew early	0	3	0	0
8. PCR+ test <= 14 days post-second dose	6	3	34	38
**N included in efficacy analysis**	**3060**	**3064**	**3247**	**3233**

† These participants received two different vaccines with the 1st dose as MenACWY and 2nd dose as ChAdoOx1 nCoV-19 were excluded. ⁑COV002 included a non-randomised open-label immunogenicity group of participants who had received a prior non-COVID ChAdOx1 vaccine and these participants are excluded.
♯Results were unavailable from some samples as the quantity of sample was not sufficient for the assay.

Table S4 Baseline characteristics of participants included in the any dose for safety population*

	COV001 (UK) N=1067		COV002 (UK) N=10663		COV003 (Brazil) N=10002		COV005 (South Africa) N=2013	
**Study**	**ChAdOx1 nCoV-19 N=534 N (%)**	**MenACWY N=533 N (%)**	**ChAdOx1 nCoV-19 N=5479 N (%)**	**MenACWY N=5184 N (%)**	**ChAdOx1 nCoV-19 N=5000 N (%)**	**MenACWY N=5002 N (%)**	**ChAdOx1 nCoV-19 N=1008 N (%)**	**Saline N=1005 N (%)**
Age								
18-55 years	533 (99.8%)	532 (99.8%)	4160 (75.9%)	4110 (79.3%)	4146 (82.9%)	4192 (83.8%)	961 (95.3%)	954 (94.9%)
56-69 years	0 (0.0%)	0 (0.0%)	635 (11.6%)	553 (10.7%)	717 (14.3%)	692 (13.8%)	47 (4.7%)	51 (5.1%)
70+ years	0 (0.0%)	0 (0.0%)	684 (12.5%)	521 (10.1%)	137 (2.7%)	118 (2.4%)	0 (0.0%)£	0 (0.0%)£
missing	1 (0.2%)	1 (0.2%)	0 (0.0%)	0 (0.0%)	0 (0.0%)	0 (0.0%)	0 (0.0%)	0 (0.0%)
Sex (female) n%	259 (48.5%)	271 (50.8%)	3251 (59.3%)	3150 (60.8%)	2764 (55.3%)	2688 (53.7%)	437 (43.4%)	440 (43.8%)
BMI (median, IQR) kg/m^2	24.2 [22.2-26.6]	24.4 [22.1-26.9]	25.4 [22.9-28.8]	25.5 [22.9-28.9]	26.0 [23.2-29.4]	25.9 [23.3-29.4]	23.8 [20.6-28.3]	23.5 [20.8-28.4]
Ethnicity								
White	485 (90.8%)	485 (91.0%)	5023 (91.7%)	4784 (92.3%)	3395 (67.9%)	3434 (68.7%)	126 (12.5%)	132 (13.1%)
Black	4 (0.7%)	2 (0.4%)	28 (0.5%)	21 (0.4%)	452 (9.0%)	464 (9.3%)	714 (70.8%)	709 (70.5%)

Asian†	23 (4.3%)	28 (5.3%)	289 (5.3%)	258 (5.0%)	127 (2.5%)	100 (2.0%)		
Mixed	11 (2.1%)	9 (1.7%)	95 (1.7%)	80 (1.5%)	994 (19.9%)	972 (19.4%)	148 (14.7%)€	143 (14.2%)€
Other	11 (2.1%)	9 (1.7%)	44 (0.8%)	41 (0.8%)	32 (0.6%)	32 (0.6%)	20 (2.0%)	21 (2.1%)
missing	0 (0.0%)	0 (0.0%)	0 (0.0%)	0 (0.0%)	0 (0.0%)	0 (0.0%)	0 (0.0%)	0 (0.0%)
% Health and social care setting workers	105 (19.7%)	91 (17.1%)	3504 (64.0%)	3481 (67.1%)	3291 (65.8%)	3252 (65.0%)	79 (7.8%)	88 (8.8%)
Co-morbidities¥								
Cardiovascular disease	0 (0.0%)	0 (0.0%)	682 (12.4%)	598 (11.5%)	801 (16.0%)	782 (15.6%)	30 (3.0%)	23 (2.3%)
Respiratory disease	0 (0.0%)	0 (0.0%)	663 (12.1%)	655 (12.6%)	502 (10.0%)	494 (9.9%)	31 (3.1%)	20 (2.0%)
Diabetes	0 (0.0%)	0 (0.0%)	117 (2.1%)	99 (1.9%)	218 (4.4%)	186 (3.7%)	3 (0.3%)	5 (0.5%)

*Any dose for safety population as defined in the statistical analysis plan. † Asian not recorded as a category in South Africa. £ Maximum age at enrolment for eligibility was 65 years in South Africa. € This category recorded as 'Coloured' in original data. ¥ As an early-phase trial COV001 excluded participants with co-morbidities.

Table S5 Timing of vaccine administration in those included in the primary analysis*

	COV002 (UK)		COV003 (Brazil)	
**Study**	**ChAdOx1 nCoV-19 N (%)**	**MenACWY N (%)**	**ChAdOx1 nCoV-19 N (%)**	**MenACWY N (%)**
Time between first and second dose LD/SD				
< 6 weeks	0 (0.0%)	0 (0.0%)		
6-8 weeks	10/1367 (0.7%)	12/1374 (0.9%)		
9-11 weeks	624/1367 (45.6%)	636/1374 (46.3%)		
≥12 weeks	733/1367 (53.6%)	726/1374 (52.8%)		
Time between first and second dose SD/SD				
< 6 weeks	453/2377 (19.1%)	454/2430 (18.7%)	1249/2063 (60.5%)	1244/2025 (61.4%)
6-8 weeks	517/2377 (21.8%)	464/2430 (19.1%)	430/2063 (20.8%)	431/2025 (21.3%)
9-11 weeks	595/2377 (25.0%)	665/2430 (27.4%)	285/2063 (13.8%)	275/2025 (13.6%)
≥12 weeks	812/2377 (34.2%)	847/2430 (34.9%)	99/2063 (4.8%)	75/2025 (3.7%)

*The LDSD, SDSD efficacy population as defined in the statistical analysis plan.

Figure S2 Kaplan-Meier cumulative incidence of primary symptomatic COVID-19 in LD/SD recipients in the UK, and in SD/SD recipients in the UK and Brazil

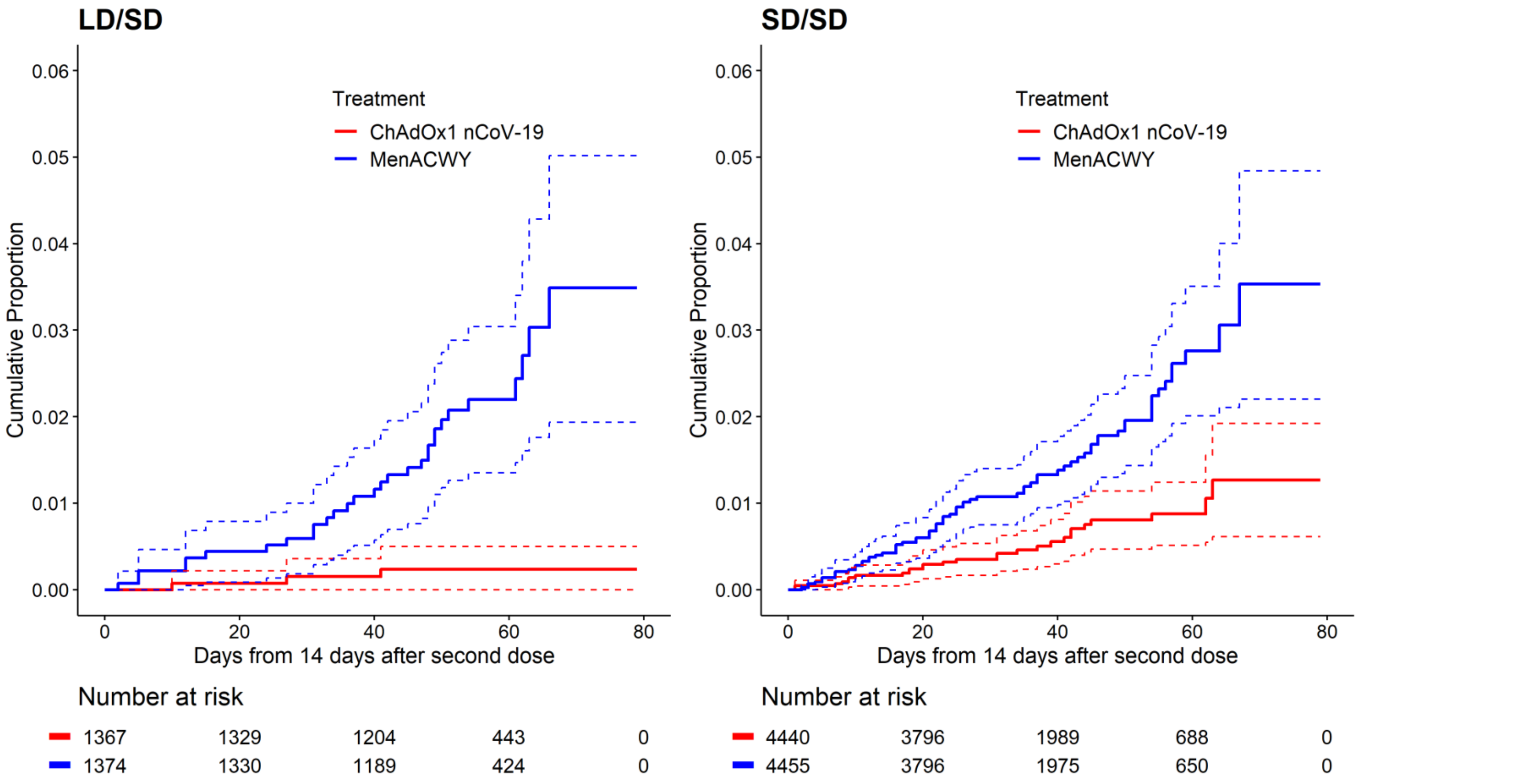

Cumulative incidence of symptomatic COVID-19 after low dose followed by standard dose (LD/SD) (left) or after two standard doses (right). Dotted lines show 95% confidence region.

Figure S3 Kaplan-Meier cumulative incidence of primary symptomatic COVID-19 in SD/SD recipients in the UK, and in SD/SD recipients in the Brazil

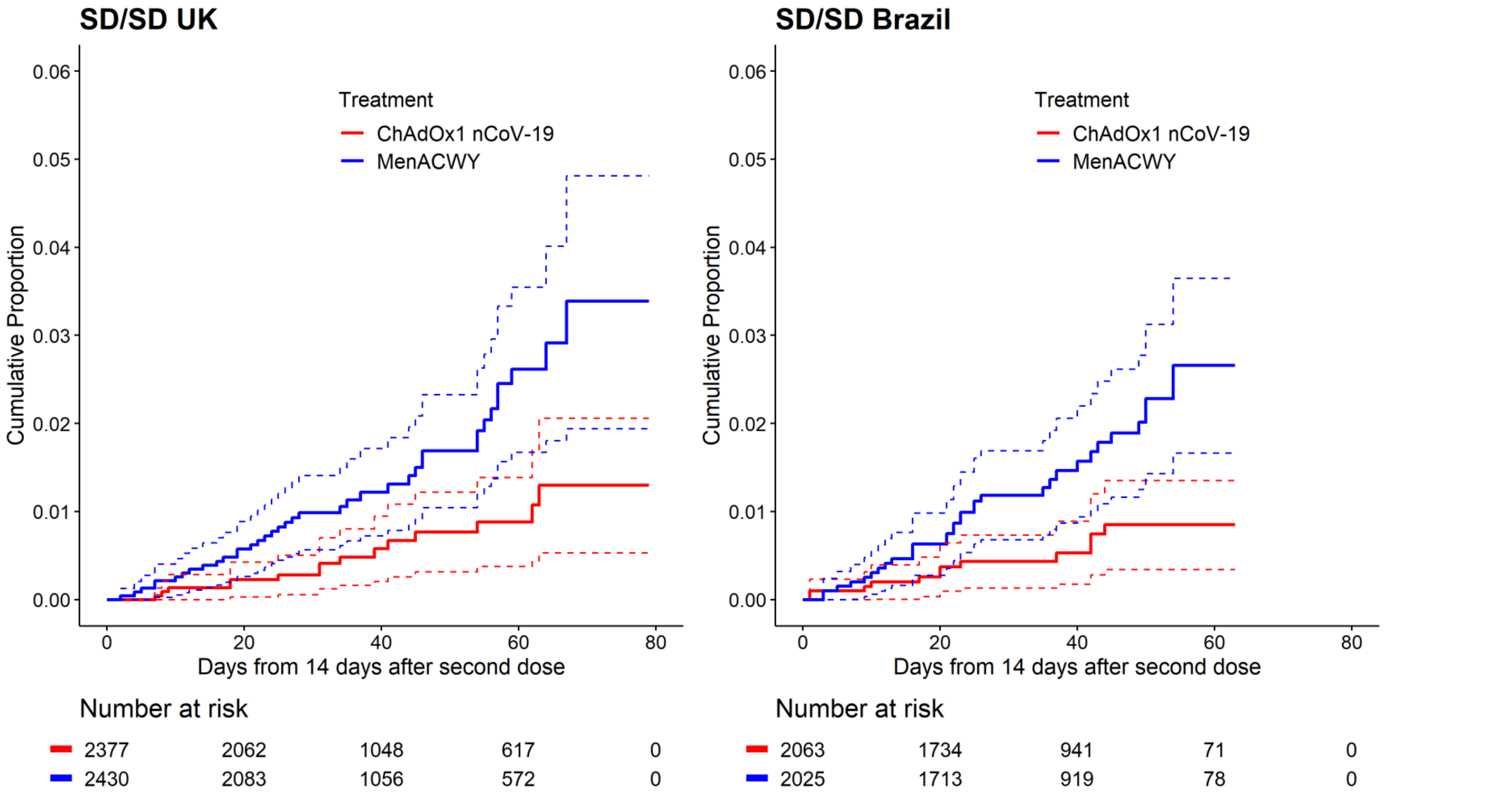

Cumulative incidence of primary symptomatic COVID-19 after two standard doses in the UK (left) and in Brazil (right). Dotted lines show 95% confidence region.

Table S6 Serious adverse events by MedDRA system organ class and preferred term at any time during the study, in randomised participants who received at least one dose of vaccine

	ChAdOx1 nCoV-19 (N = 12021)		Control (N = 11724)	
**System Organ Class** / **Preferred Term (MedDRA version 23.1)**	**Number (%) of Participants[a]**	**Number of Events**	**Number (%) of Participants[a]**	**Number of Events**
**Participants with any SAE**	**79 (0.7)**	**84**	**89 (0.8)**	**91**
**Blood and lymphatic system disorders**	**0**	**0**	**1 (<0.1)**	**1**
Autoimmune haemolytic anaemia	0	0	1 (<0.1)	1
**Cardiac disorders**	**5 (<0.1)**	**5**	**6 (0.1)**	**6**
Angina pectoris	3 (<0.1)	3	0	0
Angina unstable	0	0	1 (<0.1)	1
Atrial flutter	1 (<0.1)	1	0	0
Atrioventricular block complete	1 (<0.1)	1	0	0
Atrioventricular block second degree	0	0	1 (<0.1)	1
Palpitations	0	0	1 (<0.1)	1
Pericarditis	0	0	2 (<0.1)	2
Supraventricular tachycardia	0	0	1 (<0.1)	1
**Congenital, familial and genetic disorders**	**0**	**0**	**1 (<0.1)**	**1**
Syringomyelia	0	0	1 (<0.1)	1
**Ear and labyrinth disorders**	**1 (<0.1)**	**1**	**0**	**0**
Auricular chondritis	1 (<0.1)	1	0	0
**Eye disorders**	**2 (<0.1)**	**2**	**0**	**0**
Retinal detachment	1 (<0.1)	1	0	0
Retinal tear	1 (<0.1)	1	0	0
**Gastrointestinal disorders**	**8 (0.1)**	**9**	**11 (0.1)**	**11**
Abdominal pain lower	0	0	1 (<0.1)	1
Anal incontinence	0	0	1 (<0.1)	1
Diarrhoea	1 (<0.1)	1	0	0
Enteritis	0	0	1 (<0.1)	1
Epiploic appendagitis	0	0	1 (<0.1)	1
Gastritis	1 (<0.1)	1	0	0
Gastrointestinal haemorrhage	1 (<0.1)	1	0	0
Gastrooesophageal reflux disease	0	0	1 (<0.1)	1
Haematemesis	0	0	1 (<0.1)	1
Incarcerated inguinal hernia	1 (<0.1)	1	0	0
Pancreatitis	0	0	1 (<0.1)	1
Pancreatitis acute	1 (<0.1)	1	1 (<0.1)	1
Small intestinal obstruction	0	0	1 (<0.1)	1
Volvulus	2 (<0.1)	3	0	0
Vomiting	1 (<0.1)	1	2 (<0.1)	2
**General disorders and administration site conditions**	**3 (<0.1)**	**3**	**2 (<0.1)**	**2**
Chest pain	2 (<0.1)	2	0	0
Non-cardiac chest pain	0	0	1 (<0.1)	1

Pain	0	0	1 (<0.1)	1
Pyrexia	1 (<0.1)	1	0	0
**Immune system disorders**	**0**	**0**	**1 (<0.1)**	**1**
Allergy to arthropod sting	0	0	1 (<0.1)	1
**Injury, poisoning and procedural complications**	**10 (0.1)**	**10**	**14 (0.1)**	**14**
Animal bite	0	0	1 (<0.1)	1
Cervical vertebral fracture	1 (<0.1)	1	0	0
Craniocerebral injury	1 (<0.1)	1	1 (<0.1)	1
Fibula fracture	1 (<0.1)	1	0	0
Forearm fracture	0	0	1 (<0.1)	1
Gun shot wound	1 (<0.1)	1	0	0
Humerus fracture	0	0	1 (<0.1)	1
Injury	0	0	1 (<0.1)	1
Intentional overdose	0	0	2 (<0.1)	2
Joint dislocation	0	0	1 (<0.1)	1
Ligament rupture	1 (<0.1)	1	0	0
Limb injury	1 (<0.1)	1	0	0
Lower limb fracture	0	0	1 (<0.1)	1
Meniscus injury	1 (<0.1)	1	0	0
Post procedural complication	0	0	1 (<0.1)	1
Procedural nausea	1 (<0.1)	1	0	0
Road traffic accident	0	0	2 (<0.1)	2
Synovial rupture	1 (<0.1)	1	0	0
Thermal burn	1 (<0.1)	1	0	0
Upper limb fracture	0	0	1 (<0.1)	1
Wrist fracture	0	0	1 (<0.1)	1
**Infections and infestations**	**18 (0.1)**	**20**	**27 (0.2)**	**27**
Acute sinusitis	0	0	1 (<0.1)	1
Appendicitis	6 (<0.1)	6	5 (<0.1)	5
COVID-19	2 (<0.1)	2	11 (0.1)	11
COVID-19 pneumonia	0	0	2 (<0.1)	2
Campylobacter colitis	1 (<0.1)	1	0	0
Complicated appendicitis	0	0	1 (<0.1)	1
Diverticulitis	2 (<0.1)	2	0	0
Gastroenteritis	0	0	1 (<0.1)	1
Haematoma infection	0	0	1 (<0.1)	1
Hepatitis infectious mononucleosis	1 (<0.1)	1	0	0
Intervertebral discitis	1 (<0.1)	1	0	0
Large intestine infection	1 (<0.1)	1	0	0
Pilonidal cyst	0	0	2 (<0.1)	2
Pneumonia bacterial	1 (<0.1)	1	0	0
Pneumonia fungal	1 (<0.1)	1	0	0
Pulmonary tuberculosis	1 (<0.1)	1	0	0
Pyelonephritis	3 (<0.1)	3	0	0
Sepsis	0	0	1 (<0.1)	1
Subcutaneous abscess	0	0	1 (<0.1)	1
Tonsillitis	0	0	1 (<0.1)	1

**Investigations**	**2 (<0.1)**	**2**	**1 (<0.1)**	**1**
C-reactive protein increased	1 (<0.1)	1	1 (<0.1)	1
Liver function test abnormal	1 (<0.1)	1	0	0
**Metabolism and nutrition disorders**	**0**	**0**	**1 (<0.1)**	**1**
Diabetic ketoacidosis	0	0	1 (<0.1)	1
**Neoplasms benign, malignant and unspecified (incl cysts and polyps)**	**4 (<0.1)**	**4**	**5 (<0.1)**	**5**
Benign neoplasm	1 (<0.1)	1	0	0
Colorectal cancer metastatic	0	0	1 (<0.1)	1
Haemangioma	0	0	1 (<0.1)	1
Lip squamous cell carcinoma	0	0	1 (<0.1)	1
Malignant melanoma	1 (<0.1)	1	0	0
Neoplasm malignant	1 (<0.1)	1	0	0
Papillary thyroid cancer	0	0	1 (<0.1)	1
Renal cancer	1 (<0.1)	1	0	0
Uterine leiomyoma	0	0	1 (<0.1)	1
**Nervous system disorders**	**7 (0.1)**	**7**	**4 (<0.1)**	**4**
Facial spasm	1 (<0.1)	1	0	0
Ischaemic stroke	1 (<0.1)	1	0	0
Migraine	1 (<0.1)	1	0	0
Multiple sclerosis	1 (<0.1)	1	0	0
Myelitis	0	0	1 (<0.1)	1
Myelitis transverse	1 (<0.1)	1	0	0
Presyncope	1 (<0.1)	1	0	0
Serotonin syndrome	1 (<0.1)	1	0	0
Subarachnoid haemorrhage	0	0	1 (<0.1)	1
Syncope	0	0	1 (<0.1)	1
Transient ischaemic attack	0	0	1 (<0.1)	1
**Musculoskeletal and connective tissue disorders**	**5 (<0.1)**	**5**	**2 (<0.1)**	**2**
Arthritis reactive	0	0	1 (<0.1)	1
Costochondritis	0	0	1 (<0.1)	1
Intervertebral disc protrusion	4 (<0.1)	4	0	0
Myalgia	1 (<0.1)	1	0	0
**Pregnancy, puerperium and perinatal conditions**	**1 (<0.1)**	**1**	**2 (<0.1)**	**2**
Abortion incomplete	0	0	1 (<0.1)	1
Abortion spontaneous	1 (<0.1)	1	1 (<0.1)	1
**Psychiatric disorders**	**1 (<0.1)**	**1**	**1 (<0.1)**	**1**
Substance abuse	1 (<0.1)	1	0	0
Substance-induced psychotic disorder	0	0	1 (<0.1)	1
**Renal and urinary disorders**	**4 (<0.1)**	**4**	**6 (0.1)**	**6**
Acute kidney injury	1 (<0.1)	1	0	0
Calculus urethral	0	0	1 (<0.1)	1
Calculus urinary	1 (<0.1)	1	1 (<0.1)	1
Nephrolithiasis	0	0	2 (<0.1)	2
Renal colic	2 (<0.1)	2	2 (<0.1)	2
**Reproductive system and breast disorders**	**7 (0.1)**	**8**	**2 (<0.1)**	**2**
Adnexal torsion	1 (<0.1)	1	0	0

Dysmenorrhoea	1 (<0.1)	1	0	0
Endometriosis	2 (<0.1)	2	0	0
Haemorrhagic ovarian cyst	1 (<0.1)	1	0	0
Ovarian germ cell teratoma benign	0	0	1 (<0.1)	1
Ovulation pain	1 (<0.1)	1	0	0
Uterine haemorrhage	1 (<0.1)	1	1 (<0.1)	1
Vaginal haemorrhage	1 (<0.1)	1	0	0
**Respiratory, thoracic and mediastinal disorders**	**1 (<0.1)**	**1**	**1 (<0.1)**	**1**
Dyspnoea exertional	1 (<0.1)	1	0	0
Haemoptysis	0	0	1 (<0.1)	1
**Skin and subcutaneous tissue disorders**	**1 (<0.1)**	**1**	**1 (<0.1)**	**1**
Angioedema	0	0	1 (<0.1)	1
Cellulitis	1 (<0.1)	1	0	0
**Social circumstances**	**0**	**0**	**1 (<0.1)**	**1**
Homicide	0	0	1 (<0.1)	1
**Vascular disorders**	**0**	**0**	**1 (<0.1)**	**1**
Peripheral ischaemia	0	0	1 (<0.1)	1

[a] Participants with multiple events in the same preferred term are counted only once in each of those preferred term. Participants with events in more than 1 preferred term are counted once in each of those preferred term.

Table S7 Adverse events of special interest by special interest category and preferred term in randomised participants who received at least one dose of vaccine (Any Dose for Safety Population)

Special Interest Category<br>Preferred Term<br>(MedDRA version 23.1)	Number (%) of Participants [a]	
	ChAdOx1 nCoV-19 (N=12021)	Control (N=11724)
**Participants with any AESI**	**95 (0.8)**	**126 (1.1)**
**Anaphylaxis**	**1 (<0.1)**	**0**
Anaphylactic reaction	1 (<0.1)	0
**Generalized convulsion**	**1 (<0.1)**	**1 (<0.1)**
Seizure	0	1 (<0.1)
Tonic convulsion	1 (<0.1)	0
**Neurologic events-other**	**64 (0.5)**	**79 (0.7)**
Dysaesthesia	4 (<0.1)	1 (<0.1)
Gait disturbance	1 (<0.1)	1 (<0.1)
Hyperaesthesia	1 (<0.1)	1 (<0.1)
Hypoaesthesia	13 (0.1)	19 (0.2)
Muscular weakness	7 (0.1)	9 (0.1)
Neuralgia	2 (<0.1)	1 (<0.1)
Neuritis	1 (<0.1)	0
Neuropathy peripheral	1 (<0.1)	0
Paraesthesia	37 (0.3)	48 (0.4)
Sensory disturbance	2 (<0.1)	1 (<0.1)
Sensory loss	3 (<0.1)	3 (<0.1)
Visual impairment	3 (<0.1)	6 (0.1)
**Potential Immune Mediated Conditions - Gastrointestinal disorders**	**1 (<0.1)**	**3 (<0.1)**
Coeliac disease	1 (<0.1)	0
Colitis ulcerative	0	2 (<0.1)
Crohn's disease	0	1 (<0.1)
**Potential Immune Mediated Conditions- Musculoskeletal disorders**	**1 (<0.1)**	**1 (<0.1)**
Ankylosing spondylitis	1 (<0.1)	0
Arthritis reactive	0	1 (<0.1)
**Potential Immune Mediated Conditions- Neuroinflammatory disorders**	**5 (<0.1)**	**4 (<0.1)**
Facial paralysis	3 (<0.1)	3 (<0.1)
Multiple sclerosis	1 (<0.1)	0
Myelitis	0	1 (<0.1)
Myelitis transverse	1 (<0.1)	0
**Potential Immune Mediated Conditions- Skin disorders**	**3 (<0.1)**	**4 (<0.1)**
Alopecia areata	0	1 (<0.1)
Psoriasis	1 (<0.1)	2 (<0.1)
Rosacea	1 (<0.1)	1 (<0.1)
Vitiligo	1 (<0.1)	0
**Potential Immune Mediated Conditions- Vasculitides**	**0**	**1 (<0.1)**

Vasculitic rash	0	1 (<0.1)
**Potential Immune Mediated Conditions- other**	**3 (<0.1)**	**3 (<0.1)**
Autoimmune haemolytic anaemia	0	1 (<0.1)
Raynaud's phenomenon	1 (<0.1)	0
Uveitis	2 (<0.1)	2 (<0.1)
**Thrombotic, thromboembolic, and neurovascular events**	**4 (<0.1)**	**8 (0.1)**
Blindness transient	0	1 (<0.1)
Coronary artery occlusion	1 (<0.1)	1 (<0.1)
Deep vein thrombosis	0	1 (<0.1)
Hemiparesis	0	1 (<0.1)
Ischaemic stroke	1 (<0.1)	0
Monoparesis	0	1 (<0.1)
Pulmonary embolism	1 (<0.1)	0
Thrombophlebitis	0	1 (<0.1)
Thrombosis	1 (<0.1)	0
Transient ischaemic attack	0	2 (<0.1)
**VAERD**	**12 (0.1)**	**23 (0.2)**
COVID-19	10 (0.1)	21 (0.2)
COVID-19 pneumonia	0	2 (<0.1)
Suspected COVID-19	2 (<0.1)	0

AESI = Adverse events of special interest.
[a] Number (%) of participants with AEs, sorted in alphabetical order for special interest category and preferred term. Participants with multiple events in the same preferred term are counted only once in each of those preferred term. Participants with events in more than 1 preferred term are counted once in each of those preferred term.

Supplementary Authors List – Oxford Vaccine Group

Marites Aban	NIHR Imperial Clinical Research Facility, London, UK
Fatola Abayomi	NIHR BRC at Guy's and St Thomas' NHS Foundation Trust
Kushala Abeysekera	University Hospitals Bristol & Weston NHS Foundation Trust
Jeremy Aboagye	Jenner Institute, Nuffield Department of Medicine, University of Oxford, UK
Matthew Adam	Clinical Infection Research Group, Regional Infectious Diseases Unit, NHS Lothian, UK
Kirsty Adams	NIHR UCLH Clinical Research Facility, London, UK
James Adamson	Public Health Wales NHS Trust, Cardiff, UK
Victoria A. Adelaja	NIHR BRC at Guy's and St Thomas' NHS Foundation Trust, UK
Gbadebo Adewetan	London Northwest University Healthcare, Northwick Park Hospital, London, UK
Syed Adlou	Oxford Vaccine Group, Department of Paediatrics, University of Oxford, UK
Khatija Ahmed	Setshaba Research Centre, Pretoria, South Africa
Yasmeen Akhalwaya	Family Centre for Research with Ubuntu, Department of Paediatrics, University of Stellenbosch, Cape Town, South Africa
Saajida Akhalwaya	Wits Reproductive Health and HIV Institute, Faculty of Health Sciences, University of the Witwatersrand, Johanesburg, South Africa
Andrew Alcock	Oxford Biomedica PLC, Transport Way, Cowley, Oxford
Aabidah Ali	Jenner Institute, Nuffield Department of Medicine, University of Oxford, UK
Elizabeth R. Allen	Jenner Institute, Nuffield Department of Medicine, University of Oxford, UK
Lauren Allen	National Infection Service, Public Health England, UK
Thamires C. D. S. C. Almeida	Instituto D'Or de Ensino e Pesquisa, Rio De Janeiro, Brazil
Mariana P. S. Alves	Instituto D'Or de Ensino e Pesquisa, Rio De Janeiro, Brazil
Fábio Santos Amorim	Hospital Couto Maia / Hospital São Rafael / I'DOR, Brazil
Foteini Andritsou	Department of Paediatrics, University of Oxford, UK
Rachel Anslow	Oxford Vaccine Group, Department of Paediatrics, University of Oxford, UK
Matthew Appleby	Clinical Microbiology and Virology Department, University College London Hospitals NHS Trust, UK
Edward H. Arbe-Barnes	University of Oxford Medical School, Medical Sciences Division, University of Oxford, UK
Markus P. Ariaans	Department of Infection, Immunity and Cardiovascular Disease, University of Sheffield, UK
Beatriz Arns	Hospital de Clinicas de Porto Alegre, Universidade Federal do Rio Grande do Sul, Brazil
Laiana Arruda	Hospital São Rafael/ ID'OR
Paula De Almeida Azi	Hospital São Rafael / Hospital Aliança/ Rede D'OR, Brazil
Lorena De Almeida Azi	Hospital São Rafael / Hospital das Clínicas (Unidade de atenção psicossocial do HUPES), Brazil

Gavin Babbage	NIHR Southampton Clinical Research Facility, Southampton, UK
Catherine Bailey	Aneurin Bevan University Health Board, Newport, Wales, UK
Kenneth F. Baker	Department of Infection and Tropical Medicine, Newcastle upon Tyne Hospitals NHS Foundation Trust and Translational and Clinical Research Institute, Immunity and Inflammation Theme, Newcastle University
Megan Baker	Jenner Institute, Nuffield Department of Medicine, University of Oxford, UK
Natalie Baker	National Infection Service, Public Health England, UK
Philip Baker	University of Oxford Medical School, Medical Sciences Division, University of Oxford, UK
Lisa Baldwin	Hull University Teaching Hospitals NHS Trust, UK
Ioana Baleanu	Clinical BioManufacturing Facility, Jenner Institute, University of Oxford, UK
Danieli Bandeira	Postgraduate Programme in Nursing, Universidade Federal de Santa Maria, Santa Maria, Brazil
Anna Bara	NIHR Imperial Clinical Research Facility, London, UK
Marcella A. S. Barbosa	Centro de Estudos e Pesquisas em Moléstias Infecciosas, Rio Grande do Norte, Brazil
Deborah Barker	Oxford University Hospitals NHS Trust, Oxford, UK
Gavin D Barlow	Experimental Medicine & Biomedicine, Hull York Medical School, UK
Eleanor Barnes	Nuffield Department of Medicine, University of Oxford, UK
Andrew S. Barr	Department of Infection and Tropical Medicine, Newcastle upon Tyne Hospitals NHS Foundation Trust, UK
Jordan R. Barrett	Jenner Institute, Nuffield Department of Medicine, University of Oxford, UK
Jessica Barrett	London Northwest University Healthcare, Northwick Park Hospital, London, UK
Louise Bates	Oxford Vaccine Group, Department of Paediatrics, University of Oxford, UK
Alexander Batten	Clinical BioManufacturing Facility, Jenner Institute, University of Oxford, UK
Kirsten Beadon	Oxford Vaccine Group, Department of Paediatrics, University of Oxford, UK
Emily Beales	Vaccine Institute, Institute of Infection & Immunity, St. Georges, University of London and St Georges University Hospitals NHS Trust, London, UK
Rebecca Beckley	Oxford Vaccine Group, Department of Paediatrics, University of Oxford, UK
Sandra Belij-Rammerstorfer	Jenner Institute, Nuffield Department of Medicine, University of Oxford, UK
Jonathan Bell	Oxford Vaccine Group, Department of Paediatrics, University of Oxford, UK
Duncan Bellamy	Jenner Institute, Nuffield Department of Medicine, University of Oxford, UK
Nancy Bellei	Universidade Federal de SaoPaulo, Brazil
Sue Belton	The University of Nottingham Health Service, Cripps Health Centre, University Park, Nottingham, UK
Adam Berg	Jenner Institute, Nuffield Department of Medicine, University of Oxford, UK
Laura Bermejo	Oxford University Hospitals NHS Trust, Oxford, UK

Eleanor Berrie	Clinical BioManufacturing Facility, Jenner Institute, University of Oxford, UK
Lisa Berry	NIHR Southampton Clinical Research Facility, Southampton, UK
Daniella Berzsenyi	Oxford Vaccine Group, Department of Paediatrics, University of Oxford, UK
Amy Beveridge	Oxford Vaccine Group, Department of Paediatrics, University of Oxford, UK
Kevin R Bewley	National Infection Service, Public Health England, UK
Helen Bexhell	Hull University Teaching Hospitals NHS Trust, UK
Sutika Bhikha	VIDA - Vaccines and Infectious Diseases Analytical Research Unit, Diepkloof, Soweto, South Africa
Asad E. Bhorat	Soweto Clinical Trials Centre, Johannesburg, South Africa
Zahedah E. Bhorat	Soweto Clinical Trials Centre, Johannesburg, South Africa
Else Margreet Bijker	Oxford Vaccine Group, Department of Paediatrics, University of Oxford, UK
Gurpreet Birch	Cobra Biologics, Keele Science Park, UK
Sarah Birch	Department of Infection and Tropical Medicine, Sheffield Teaching Hospitals NHS Foundation Trust, UK
Adam Bird	Oxford Biomedica PLC, Transport Way, Cowley, Oxford
Olivia Bird	Vaccine Institute, Institute of Infection & Immunity, St. Georges, University of London and St Georges University Hospitals NHS Trust, London, UK
Karen Bisnauthsing	NIHR BRC at Guy's and St Thomas' NHS Foundation Trust
Mustapha Bittaye	Jenner Institute, Nuffield Department of Medicine, University of Oxford, UK
Katherine Blackstone	NIHR BRC at Guy's and St Thomas' NHS Foundation Trust, UK
Luke Blackwell	Oxford Vaccine Group, Department of Paediatrics, University of Oxford, UK
Heather Bletchly	Oxford Vaccine Group, Department of Paediatrics, University of Oxford, UK
Caitlin L Blundell	Department of Biochemistry, University of Oxford, UK
Susannah R Blundell	Department of Biochemistry, University of Oxford, UK
Pritesh Bodalia	Pharmacy, University College London Hospitals NHS Trust, UK
Bruno C. Boettger	Universidade Federal de SaoPaulo, Brazil
Emma Bolam	Clinical BioManufacturing Facility, Jenner Institute, University of Oxford, UK
Elena Boland	Clinical BioManufacturing Facility, Jenner Institute, University of Oxford, UK
Daan Bormans	Halix B.V., Tinbergenweg 1, 2333 BB Leiden, Netherlands
Nicola Borthwick	Jenner Institute, Nuffield Department of Medicine, University of Oxford, UK
Francesca Bowring	Oxford University Hospitals NHS Trust, Oxford, UK
Amy Boyd	Jenner Institute, Nuffield Department of Medicine, University of Oxford, UK
Penny Bradley	Department of Pharmacy, Newcastle upon Tyne Hospitals NHS Foundation Trust, UK
Tanja Brenner	Clinical BioManufacturing Facility, Jenner Institute, University of Oxford, UK

Phillip Brown	National Infection Service, Public Health England, UK
Claire Brown	NIHR/Wellcome Trust Birmingham Clinical Research Facility, Birmingham, UK
Charlie Brown-O'Sullivan	Jenner Institute, Nuffield Department of Medicine, University of Oxford, UK
Scott Bruce	Cobra Biologics, Keele Science Park, UK
Emily Brunt	National Infection Service, Public Health England, UK
Ruaridh Buchan	NHS Lothian, Edinburgh, UK
William Budd	NIHR Imperial Clinical Research Facility, London, UK
Yusuf A. Bulbulia	Soweto Clinical Trials Centre, Johannesburg, South Africa
Melanie Bull	Oxford Biomedica PLC, Transport Way, Cowley, Oxford
Jamie Burbage	Oxford Vaccine Group, Department of Paediatrics, University of Oxford, UK
Hassan Burhan	Department of Clinical Sciences, Liverpool School of Tropical Medicine and Liverpool University Hospitals NHS Foundation Trust, UK
Aileen Burn	Research Directorate, Newcastle upon Tyne Hospitals NHS Foundation Trust, UK
Karen R Buttigieg	National Infection Service, Public Health England, UK
Nicholas Byard	Jenner Institute, Nuffield Department of Medicine, University of Oxford, UK
Ingrid Cabera Puig	Jenner Institute, Nuffield Department of Medicine, University of Oxford, UK
Gloria Calderon	Windsor Research Unit, Cambridge and Peterborough NHS Foundation Trust, UK
Anna Calvert	Vaccine Institute, Institute of Infection & Immunity, St. Georges, University of London and St Georges University Hospitals NHS Trust, London, UK
Susana Camara	Oxford Vaccine Group, Department of Paediatrics, University of Oxford, UK
Michelangelo Cao	Nuffield Department of Clinical Neurosciences, University of Oxford, UK
Federica Cappuccini	Jenner Institute, Nuffield Department of Medicine, University of Oxford, UK
João R. Cardoso	Hospital Quinta D'OR, Rede D’OR, São Luiz, Rio De Janeiro, Brazil
Melanie Carr	Oxford Vaccine Group, Department of Paediatrics, University of Oxford, UK
Miles W Carroll	National Infection Service, Public Health England, UK
Andrew Carson-Stevens	Division of Population Medicine, School of Medicine, Cardiff University, UK
Yasmin de M. Carvalho	Centro de Estudos e Pesquisas em Moléstias Infecciosas, Rio Grande do Norte, Brazil
José A. M. Carvalho	Department of Clinical and Toxicological Analysis- Universidade Federal de Santa Maria, Santa Maria, Brazil
Helen R Casey	North Bristol NHS Trust, Bristol, UK
Paul Cashen	Pall Europe Ltd, Harbourgate Business Park, Portsmouth, UK
Lucia Carratala Castro	Vaccine Institute, Institute of Infection & Immunity, St. Georges, University of London and St Georges University Hospitals NHS Trust, London, UK

Thais R. Y Castro	Postgraduate Programme in Pharmaceutical Sciences, Universidade Federal de Santa Maria, Santa Maria, Brazil
Katrina Cathie	Paediatric Medicine, University of Cambridge, UK
Ana Cavey	Nuffield Department of Clinical Neurosciences, University of Oxford, UK
José Cerbino-Neto	Instituto D'Or de Ensino e Pesquisa, Rio De Janeiro, Brazil
Jim Chadwick	National Infection Service, Public Health England, UK
Sue Charlton	National Infection Service, Public Health England, UK
David Chapman	Oxfordshire Clinical Commissioning Group, Oxford, UK
Irina Chelysheva	Oxford Vaccine Group, Department of Paediatrics, University of Oxford, UK
Oliver Chester	Oxford Vaccine Group, Department of Paediatrics, University of Oxford, UK
Sunder Chita	London Northwest University Healthcare, Northwick Park Hospital, London, UK
Jee-Sun Cho	Jenner Institute, Nuffield Department of Medicine, University of Oxford, UK
Liliana Cifuentes	Kennedy Institute of Rheumatology, Nuffield Department of Orthopaedics, The University of Oxford, UK
Elizabeth Clark	Oxford Vaccine Group, Department of Paediatrics, University of Oxford, UK
Matthew Clark	Oxford Vaccine Group, Department of Paediatrics, University of Oxford, UK
Andrea Clarke	Clinical Infection Research Group, Regional Infectious Diseases Unit, NHS Lothian, UK
Elizabeth A Clutterbuck	Oxford Vaccine Group, Department of Paediatrics, University of Oxford, UK
Sarah L. K. Collins	Womens Reproductive Health, University of Oxford, UK
Christopher P. Conlon	Nuffield Department of Medicine, University of Oxford, UK
Sean Connarty	London Northwest University Healthcare, Northwick Park Hospital, London, UK
Naomi S. Coombes	National Infection Service, Public Health England, UK
Cushla Cooper	Nuffield Dept of Orthopaedics Rheumatology and Musculoskeletal Medicine, University of Oxford, UK
Rachel Cooper	Oxford Vaccine Group, Department of Paediatrics, University of Oxford, UK
Lynne Cornelissen	Family Centre for Research with Ubuntu, Department of Paediatrics, University of Stellenbosch, Cape Town, South Africa
Tumena Corrah	London Northwest University Healthcare, Northwick Park Hospital, London, UK
Catherine Cosgrove	Vaccine Institute, Institute of Infection & Immunity, St. Georges, University of London and St Georges University Hospitals NHS Trust, London, UK
Tony Cox OBE	NIHR National Biosample Centre, Milton Keynes, UK
Wendy E. M. Crocker	Jenner Institute, Nuffield Department of Medicine, University of Oxford, UK
Sarah Crosbie	Oxford University Hospitals NHS Trust, Oxford, UK
Lorraine Cullen	Hull University Teaching Hospitals NHS Trust, UK
Dan Cullen	Nuffield Department of Clinical Neurosciences, University of Oxford, UK

Debora R. M. F. Cunha	Hospital de Clinicas de Porto Alegre, Universidade Federal do Rio Grande do Sul, Brazil
Christina J Cunningham	Oxford Vaccine Group, Department of Paediatrics, University of Oxford, UK
Fiona C. Cuthbertson	Nuffield Department of Clinical Neurosciences, University of Oxford, UK
Suzete N. Farias Da Guarda	Universidade Federal da Bahia / Hospital São Rafael/ ID’OR, Brazil
Larissa P. da Silva	Hospital de Clinicas de Porto Alegre, Universidade Federal do Rio Grande do Sul, Brazil
Brad E Damratoski	Clinical BioManufacturing Facility, Jenner Institute, University of Oxford, UK
Zsofia Danos	Vaccine Institute, Institute of Infection & Immunity, St. Georges, University of London and St Georges University Hospitals NHS Trust, London, UK
Maria T. D. C. Dantas	Centro de Estudos e Pesquisas em Moléstias Infecciosas, Rio Grande do Norte, Brazil
Paula Darroch	NIHR BRC at Guy's and St Thomas' NHS Foundation Trust, UK
Mehreen S Datoo	Jenner Institute, Nuffield Department of Medicine, University of Oxford, UK
Chandrabali Datta	Clinical BioManufacturing Facility, Jenner Institute, University of Oxford, UK
Malika Davids	Centre for Lung Infection and Immunity, Division of Pulmonology, Department of Medicine and UCT Lung Institute & South African MRC/UCT Centre for the Study of Antimicrobial Resistance, University of Cape Town, Cape Town, South Africa
Sarah L Davies	Cobra Biologics, Keele Science Park, UK
Hannah Davies	Jenner Institute, Nuffield Department of Medicine, University of Oxford, UK
Elizabeth J. Davis	Oxford University Hospitals NHS Trust, Oxford, UK
Judith Davis	Oxford Vaccine Group, Department of Paediatrics, University of Oxford, UK
John Davis	Research Directorate, Newcastle upon Tyne Hospitals NHS Foundation Trust, UK
Maristela M. D. Nobrega	Universidade Federal de SaoPaulo, Brazil
Lis Moreno De Oliveira Kalid	Rede D’OR São Luiz, Brazil
David Dearlove	Department of Physiology Anatomy and Genetics, University of Oxford, UK
Tesfaye Demissie	Oxford Vaccine Group, Department of Paediatrics, University of Oxford, UK
Amisha Desai	NIHR/Wellcome Trust Birmingham Clinical Research Facility, Birmingham, UK
Stefania Di Marco	Advent SRL, Italy
Claudio Di Maso	Oxford Vaccine Group, Department of Paediatrics, University of Oxford, UK
Maria I. S. Dinelli	Universidade Federal de SaoPaulo, Brazil
Tanya Dinesh	Oxford Vaccine Group, Department of Paediatrics, University of Oxford, UK
Claire Docksey	Cobra Biologics, Keele Science Park, UK
Christina Dold	Oxford Vaccine Group, Department of Paediatrics, University of Oxford, UK

Tao Dong	Chinese Academy of Medical Sciences Oxford Institute, Nuffield Department of Medicine, Oxford University, UK
Francesca R Donnellan	Jenner Institute, Nuffield Department of Medicine, University of Oxford, UK
Tannyth Gomes Dos Santos	Centro de Estudos e Pesquisas em Moléstias Infecciosas, Rio Grande do Norte, Brazil
Thainá G. dos Santos	Hospital de Clinicas de Porto Alegre, Universidade Federal do Rio Grande do Sul, Brazil
Erika Pacheco Dos Santos	Postgraduate Programme in Nursing, Universidade Federal de Santa Maria, Santa Maria, Brazil
Naomi Douglas	Oxford Vaccine Group, Department of Paediatrics, University of Oxford, UK
Charlotte Downing	University of Oxford Medical School, Medical Sciences Division, University of Oxford, UK
Jonathan Drake	University of Oxford Medical School, Medical Sciences Division, University of Oxford, UK
Rachael Drake-Brockman	Oxford Vaccine Group, Department of Paediatrics, University of Oxford, UK
Kimberley Driver	NIHR UCLH Clinical Research Facility, London, UK
Ruth Elizabeth Drury	Oxford Vaccine Group, Department of Paediatrics, University of Oxford, UK
Susanna J. Dunachie	Centre for Tropical Medicine & Global Health, Nuffield Department of Medicine, Oxford, UK
Benjamin S. Durham	Department of Infection, Immunity and Cardiovascular Disease, University of Sheffield, UK
Lidiana Dutra	Postgraduate Programme in Nursing, Universidade Federal de Santa Maria, Santa Maria, Brazil
Nicholas J. W. Easom	Hull University Teaching Hospitals NHS Trust, UK
Samual van Eck	Wits Reproductive Health and HIV Institute, Faculty of Health Sciences, University of the Witwatersrand, Johanesburg, South Africa
Mandy Edwards	Aneurin Bevan University Health Board, Newport, Wales, UK
Nick J Edwards	Jenner Institute, Nuffield Department of Medicine, University of Oxford, UK
Omar M. El Muhanna	Clinical BioManufacturing Facility, Jenner Institute, University of Oxford, UK
Sean C Elias	Jenner Institute, Nuffield Department of Medicine, University of Oxford, UK
Michael J. Elmore	National Infection Service, Public Health England, UK
Marcus Rex English	University of Oxford Medical School, Medical Sciences Division, University of Oxford, UK
Aliasgar Esmail	Centre for Lung Infection and Immunity, Division of Pulmonology, Department of Medicine and UCT Lung Institute & South African MRC/UCT Centre for the Study of Antimicrobial Resistance, University of Cape Town, Cape Town, South Africa
Yakub Moosa Essack	Soweto Clinical Trials Centre, Johannesburg, South Africa
Eoghan Farmer	Department of Infectious Diseases, Queen Elizabeth University Hospital, Glasgow, UK

Mutjaba Ghulam Farooq	Oxford Vaccine Group, Department of Paediatrics, University of Oxford, UK
Madlen Farrar	Department of Clinical Sciences, Liverpool School of Tropical Medicine and Liverpool University Hospitals NHS Foundation Trust, UK
Leonard Farrugia	Department of Infectious Diseases, Queen Elizabeth University Hospital, Glasgow, UK
Beverley Faulkner	Research & Innovation, North Bristol NHS Trust, Bristol, UK
Sofiya Fedosyuk	Jenner Institute, Nuffield Department of Medicine, University of Oxford, UK
Sally Felle	Oxford Vaccine Group, Department of Paediatrics, University of Oxford, UK
Shuo Feng	Oxford Vaccine Group, Department of Paediatrics, University of Oxford, UK
Carla Ferreira Da Silva	Oxford Vaccine Group, Department of Paediatrics, University of Oxford, UK
Samantha Field	Nuffield Department of Population Health, University of Oxford, UK
Richard Fisher	Clinical BioManufacturing Facility, Jenner Institute, University of Oxford, UK
Amy Flaxman	Jenner Institute, Nuffield Department of Medicine, University of Oxford, UK
James Fletcher	NIHR Imperial Clinical Research Facility, London, UK
Hazel Fofie	Vaccine Institute, Institute of Infection & Immunity, St. Georges, University of London and St Georges University Hospitals NHS Trust, London, UK
Henry Fok	NIHR BRC at Guy's and St Thomas' NHS Foundation Trust and King's College London British Heart Foundation Centre, School of Cardiovascular Medicine and Sciences, UK
Karen J Ford	Oxford Vaccine Group, Department of Paediatrics, University of Oxford, UK
Jamie Fowler	Jenner Institute, Nuffield Department of Medicine, University of Oxford, UK
Pedro H. A. Fraiman	Centro de Estudos e Pesquisas em Moléstias Infecciosas, Rio Grande do Norte, Brazil
Emma Francis	Oxford Vaccine Group, Department of Paediatrics, University of Oxford, UK
Marília M. Franco	Rede D'OR São Luiz, Brazil
John Frater	Nuffield Department of Medicine, University of Oxford, UK
Marilúcia S. M. Freire	Centro de Estudos e Pesquisas em Moléstias Infecciosas, Rio Grande do Norte, Brazil
Samantha H Fry	Family Centre for Research with Ubuntu, Department of Paediatrics, University of Stellenbosch, Cape Town, South Africa
Sabrina Fudge	University Hospitals Bristol & Weston NHS Foundation Trust
Julie Furze	Jenner Institute, Nuffield Department of Medicine, University of Oxford, UK
Michelle Fuskova	Jenner Institute, Nuffield Department of Medicine, University of Oxford, UK
Pablo Galian-Rubio	Clinical BioManufacturing Facility, Jenner Institute, University of Oxford, UK
Eva Galiza	Vaccine Institute, Institute of Infection & Immunity, St. Georges, University of London and St Georges University Hospitals NHS Trust, London, UK
Harriet Garlant	National Infection Service, Public Health England, UK

Madita Gavrila	Oxford University Hospitals NHS Trust, Oxford, UK
Aisla Geddes	NHS Lothian, Edinburgh, UK
Karyna A. Gibbons	Oxford University Hospitals NHS Trust, Oxford, UK
Ciaran Gilbride	Jenner Institute, Nuffield Department of Medicine, University of Oxford, UK
Hardeep Gill	Oxford University Hospitals NHS Trust, Oxford, UK
Sharon Glynn	Department of Clinical Sciences, Liverpool School of Tropical Medicine and Liverpool University Hospitals NHS Foundation Trust, UK
Kerry Godwin	National Infection Service, Public Health England, UK
Karishma Gokani	NIHR/Wellcome Trust Birmingham Clinical Research Facility, Birmingham, UK
Ursula Carvalho Goldoni	Hospital Quinta D'OR, Rede D'OR, São Luiz, Rio De Janeiro, Brazil
Maria Luisa Freire Gonçalves	Hospital São Rafael / CEDAP - Centro Estadual Especializado em Diagnóstico, Assistência e Pesquisa, Brazil
Isabela G. S. Gonzalez	Universidade Federal de SaoPaulo, Brazil
Jayne Goodwin	Health and Care Research Wales, Cardiff, UK
Amina Goondiwala	Soweto Clinical Trials Centre, Johannesburg, South Africa
Katherine Gordon-Quayle	Nuffield Department of Surgical Sciences, University of Oxford, UK
Giacomo Gorini	Jenner Institute, Nuffield Department of Medicine, University of Oxford, UK
Janet Grab	Wits Reproductive Health and HIV Institute, Faculty of Health Sciences, University of the Witwatersrand, Johanesburg, South Africa
Lara Gracie	Oxford Vaccine Group, Department of Paediatrics, University of Oxford, UK
Melanie Greenland	Oxford Vaccine Group, Department of Paediatrics, University of Oxford, UK
Nicola Greenwood	Jenner Institute, Nuffield Department of Medicine, University of Oxford, UK
Johann Greffrath	VIDA - Vaccines and Infectious Diseases Analytical Research Unit, Diepkloof, Soweto, South Africa
Marisa M. Groenewald	Family Centre for Research with Ubuntu, Department of Paediatrics, University of Stellenbosch, Cape Town, South Africa
Leonardo Grossi	Hospital Quinta D'OR, Rede D'OR, São Luiz, Rio De Janeiro, Brazil
Gaurav Gupta	Jenner Institute, Nuffield Department of Medicine, University of Oxford, UK
Mark Hackett	University Hospitals Bristol & Weston NHS Foundation Trust
Bassam Hallis	National Infection Service, Public Health England, UK
Mainga Hamaluba	KEMRI-Wellcome Trust Research Programme and Centre for Tropical Medicine & Global Health, Nuffield Department of Medicine, Oxford, UK
Elizabeth Hamilton	Nuffield Department of Population Health, University of Oxford, UK
Joseph Hamlyn	Oxford Vaccine Group, Department of Paediatrics, University of Oxford, UK
Daniel Hammersley	The University of Nottingham Health Service, Cripps Health Centre, University Park, Nottingham, UK

Aidan T. Hanrath	Department of Infection and Tropical Medicine, Newcastle upon Tyne Hospitals NHS Foundation Trust and Translational and Clinical Research Institute, Immunity and Inflammation Theme, Newcastle University
Brama Hanumunthadu	Oxford Vaccine Group, Department of Paediatrics, University of Oxford, UK
Stephanie A Harris	Jenner Institute, Nuffield Department of Medicine, University of Oxford, UK
Clair Harris	NIHR BRC at Guy's and St Thomas' NHS Foundation Trust, UK
Tara Harris	Oxford University Hospitals NHS Trust, Oxford, UK
Thomas D. Harrison	Department of Infection and Tropical Medicine, Sheffield Teaching Hospitals NHS Foundation Trust, UK
Daisy Harrison	Oxford Vaccine Group, Department of Paediatrics, University of Oxford, UK
Thomas C Hart	Oxford Vaccine Group, Department of Paediatrics, University of Oxford, UK
Birgit Hartnell	Vaccines Manufacturing and Innovation Centre, Oxford Science Park, Oxford, UK
Shadin Hassan	Department of Infectious Diseases, Queen Elizabeth University Hospital, Glasgow, UK
John Haughney	Department of Infectious Diseases, Queen Elizabeth University Hospital, Glasgow, UK
Sophia Hawkins	Oxford Vaccine Group, Department of Paediatrics, University of Oxford, UK
Jodie Hay	Paul O'Gorman Leukaemia Research Centre, University of Glasgow, UK and Lighthouse Laboratory, Queen Elizabeth University Hospital, Glasgow, UK
Ian Head	University Hospitals Bristol & Weston NHS Foundation Trust
John Aaron Henry	University of Oxford Medical School, Medical Sciences Division, University of Oxford, UK
Macarena Hermosin Herrera	Oxford University Hospitals NHS Trust, Oxford, UK
David B Hettle	Infection Sciences, North Bristol NHS Trust, Bristol, UK
Jennifer Hill	Oxford Vaccine Group, Department of Paediatrics, University of Oxford, UK
Gina Hodges	Clinical BioManufacturing Facility, Jenner Institute, University of Oxford, UK
Elizea Horne	Wits Reproductive Health and HIV Institute, Faculty of Health Sciences, University of the Witwatersrand, Johanesburg, South Africa
Mimi M Hou	Jenner Institute, Nuffield Department of Medicine, University of Oxford, UK
Catherine Houlihan	Clinical Microbiology and Virology Department, University College London Hospitals NHS Trust, UK
Elizabeth Howe	Oxford Vaccine Group, Department of Paediatrics, University of Oxford, UK
Nicola Howell	Oxford Vaccine Group, Department of Paediatrics, University of Oxford, UK
Jonathan Humphreys	Vaccines Manufacturing and Innovation Centre, Oxford Science Park, Oxford, UK
Holly E. Humphries	National Infection Service, Public Health England, UK
Katrina Hurley	University Hospitals Bristol & Weston NHS Foundation Trust
Claire Huson	AstraZeneca BioPharmaceuticals PLC

Angela Hyder-Wright	Academic Respiratory Unit, University of Bristol, Southmead Hospital, Bristol, UK
Catherine Hyams	Department of Clinical Sciences, Liverpool School of Tropical Medicine and Liverpool University Hospitals NHS Foundation Trust, UK
Sabina Ikram	Vaccine Institute, Institute of Infection & Immunity, St. Georges, University of London and St Georges University Hospitals NHS Trust, London, UK
Alka Ishwarbhai	Jenner Institute, Nuffield Department of Medicine, University of Oxford, UK
Monica Ivan	Hull University Teaching Hospitals NHS Trust, UK
Poppy Iveson	University of Oxford Medical School, Medical Sciences Division, University of Oxford, UK
Vidyashankara Iyer	AstraZeneca BioPharmaceuticals PLC
Frederic Jackson	Clinical BioManufacturing Facility, Jenner Institute, University of Oxford, UK
Jeanne De Jager	Family Centre for Research with Ubuntu, Department of Paediatrics, University of Stellenbosch, Cape Town, South Africa
Shameem Jaumdally	Centre for Lung Infection and Immunity, Division of Pulmonology, Department of Medicine and UCT Lung Institute & South African MRC/UCT Centre for the Study of Antimicrobial Resistance, University of Cape Town, Cape Town, South Africa
Helen Jeffers	Nuffield Department of Medicine, University of Oxford, UK
Natasha Jesudason	MRC-University of Glasgow Centre for Virus Research & Department of Infectious Diseases, Queen Elizabeth University Hospital, UK
Bryony Jones	Department of Rheumatology, Queen Alexandra Hospital, Portsmouth Hospitals NHS Trust, UK
Christopher Jones	Infection Sciences, North Bristol NHS Trust, Bristol, UK
Kathryn Jones	Jenner Institute, Nuffield Department of Medicine, University of Oxford, UK
Elizabeth Jones	Oxford Vaccine Group, Department of Paediatrics, University of Oxford, UK
Marianna Rocha Jorge	Rede D'OR São Luiz, Brazil
Aylin Jose	VIDA - Vaccines and Infectious Diseases Analytical Research Unit, Diepkloof, Soweto, South Africa
Amar Joshi	Pall Europe Ltd, Harbourgate Business Park, Portsmouth, UK
Eduardo A. M. S. Júnior	Centro de Estudos e Pesquisas em Moléstias Infecciosas, Rio Grande do Norte, Brazil
Joanne Kadziola	Clinical Research Facility, Sheffield Teaching Hospitals NHS Foundation Trust, UK
Reshma Kailath	Jenner Institute, Nuffield Department of Medicine, University of Oxford, UK
Faeeza Kana	Soweto Clinical Trials Centre, Johannesburg, South Africa
Konstantinos Karampatsas	Vaccine Institute, Institute of Infection & Immunity, St. Georges, University of London and St Georges University Hospitals NHS Trust, London, UK
Mwila Kasanyinga	Oxford Vaccine Group, Department of Paediatrics, University of Oxford, UK
Jade Keen	Oxford Vaccine Group, Department of Paediatrics, University of Oxford, UK

Elizabeth J. Kelly	AstraZeneca BioPharmaceuticals PLC
Dearbhla M Kelly	Nuffield Department of Clinical Neurosciences, University of Oxford, UK
Debbie Kelly	Oxford University Hospitals NHS Trust, Oxford, UK
Sarah Kelly	Oxford Vaccine Group, Department of Paediatrics, University of Oxford, UK
David Kerr	Oxford Vaccine Group, Department of Paediatrics, University of Oxford, UK
Renato De Ávila Kfouri	Universidade Federal de SaoPaulo, Brazil
Liaquat Khan	Oxford Vaccine Group, Department of Paediatrics, University of Oxford, UK
Baktash Khozoee	Jenner Institute, Nuffield Department of Medicine, University of Oxford, UK
Sarah Kidd	University Hospitals Bristol & Weston NHS Foundation Trust
Annabel Killen	University of Oxford Medical School, Medical Sciences Division, University of Oxford, UK
Jasmin Kinch	Oxford Vaccine Group, Department of Paediatrics, University of Oxford, UK
Patrick Kinch	Oxford Vaccine Group, Department of Paediatrics, University of Oxford, UK
Lloyd D W King	Jenner Institute, Nuffield Department of Medicine, University of Oxford, UK
Thomas B King	University of Oxford Medical School, Medical Sciences Division, University of Oxford, UK
Lucy Kingham	Jenner Institute, Nuffield Department of Medicine, University of Oxford, UK
Paul Klenerman	Peter Medawar Building for Pathogen Research, NDM Experimental Medicine, University of Oxford, UK
Francesca Knapper	University Hospitals Bristol & Weston NHS Foundation Trust
Julian C. Knight	Wellcome Trust Centre for Human Genetics, University of Oxford, UK
Daniel Knott	National Infection Service, Public Health England, UK
Stanislava Koleva	Oxford Vaccine Group, Department of Paediatrics, University of Oxford, UK
Matilda Lang	London Northwest University Healthcare, Northwick Park Hospital, London, UK
Gail Lang	Nuffield Dept of Orthopaedics Rheumatology and Musculoskeletal Medicine, University of Oxford, UK
Colin W Larkworthy	Jenner Institute, Nuffield Department of Medicine, University of Oxford, UK
Jessica P J Larwood	University of Oxford Medical School, Medical Sciences Division, University of Oxford, UK
Rebecca Law	Oxford University Hospitals NHS Trust, Oxford, UK
Erica M. Lazarus	Perinatal HIV Research Unit, Faculty of Health Sciences, University of the Witwatersrand, Johannesburg, South Africa
Amanda Leach	AstraZeneca BioPharmaceuticals PLC
Emily A. Lees	Oxford Vaccine Group, Department of Paediatrics, University of Oxford, UK
Nana-Marie Lemm	NIHR Imperial Clinical Research Facility, London, UK
Álvaro Edson Ramos Lessa	Hospital São Rafael / ID’OR, Brazil
Stephanie Leung	National Infection Service, Public Health England, UK

Yuanyuan Li	Jenner Institute, Nuffield Department of Medicine, University of Oxford, UK
Amelia M Lias	Jenner Institute, Nuffield Department of Medicine, University of Oxford, UK
Kostas Liatsikos	Department of Clinical Sciences, Liverpool School of Tropical Medicine and Liverpool University Hospitals NHS Foundation Trust, UK
Aline Linder	Oxford Vaccine Group, Department of Paediatrics, University of Oxford, UK
Samuel Lipworth	Jenner Institute, Nuffield Department of Medicine, University of Oxford, UK
Shuchang Liu	Clinical BioManufacturing Facility, Jenner Institute, University of Oxford, UK
Xinxue Liu	Oxford Vaccine Group, Department of Paediatrics, University of Oxford, UK
Adam Lloyd	Clinical Research Facility Edinburgh, UK
Stephanie Lloyd	Oxford University Hospitals NHS Trust, Oxford, UK
Lisa Loew	Clinical BioManufacturing Facility, Jenner Institute, University of Oxford, UK
Raquel Lopez Ramon	Jenner Institute, Nuffield Department of Medicine, University of Oxford, UK
Leandro Lora	Hospital Quinta D'OR, Rede D’OR, São Luiz, Rio De Janeiro, Brazil
Vicki Lowthorpe	Hull University Teaching Hospitals NHS Trust, UK
Kleber Giovanni Luz	Universidade Federal do Rio Grande do Norte - UFRN, Brazil
Jonathan C. MacDonald	Department of Gastroenterology, Queen Elizabeth University Hospital, Glasgow, UK
Gordon MacGregor	Department of Respiratory Medicine, Queen Elizabeth University Hospital, Glasgow, UK
Meera Madhavan	Jenner Institute, Nuffield Department of Medicine, University of Oxford, UK
David O. Mainwaring	Pall Europe Ltd, Harbourgate Business Park, Portsmouth, UK
Edson Makambwa	Centre for Lung Infection and Immunity, Division of Pulmonology, Department of Medicine and UCT Lung Institute & South African MRC/UCT Centre for the Study of Antimicrobial Resistance, University of Cape Town, Cape Town, South Africa
Rebecca Makinson	Jenner Institute, Nuffield Department of Medicine, University of Oxford, UK
Mookho Malahleha	Setshaba Research Centre, Pretoria, South Africa
Ross Malamatsho	Setshaba Research Centre, Pretoria, South Africa
Garry Mallett	University of Oxford Medical School, Medical Sciences Division, University of Oxford, UK
Kushal Mansatta	University of Oxford Medical School, Medical Sciences Division, University of Oxford, UK
Takalani Maoko	Perinatal HIV Research Unit, Faculty of Health Sciences, University of the Witwatersrand, Johannesburg, South Africa
Katlego Mapetla	Setshaba Research Centre, Pretoria, South Africa
Natalie G. Marchevsky	Oxford Vaccine Group, Department of Paediatrics, University of Oxford, UK
Spyridoula Marinou	Oxford Vaccine Group, Department of Paediatrics, University of Oxford, UK
Emma Marlow	Jenner Institute, Nuffield Department of Medicine, University of Oxford, UK

Gabriela N. Marques	Rede D'OR São Luiz, Brazil
Paula Marriott	Kennedy Institute of Rheumatology, Nuffield Department of Orthopaedics, The University of Oxford, UK
Richard P. Marshall	AstraZeneca BioPharmaceuticals PLC
Julia L. Marshall	Jenner Institute, Nuffield Department of Medicine, University of Oxford, UK
Flávia J. Martins	Universidade Federal de SaoPaulo, Brazil
Masebole Masenya	Wits Reproductive Health and HIV Institute, Faculty of Health Sciences, University of the Witwatersrand, Johanesburg, South Africa
Mduduzi Masilela	Setshaba Research Centre, Pretoria, South Africa
Shauna K. Masters	Nuffield Dept of Orthopaedics Rheumatology and Musculoskeletal Medicine, University of Oxford, UK
Moncy Mathew	Pharmacy Clinical Trials (Adult), Guy's and St Thomas NHS Foundation Trust, UK
Hosea Matlebjane	Setshaba Research Centre, Pretoria, South Africa
Kedidimetse Matshidiso	Perinatal HIV Research Unit, Faculty of Health Sciences, University of the Witwatersrand, Johannesburg, South Africa
Olga Mazur	Oxford Vaccine Group, Department of Paediatrics, University of Oxford, UK
Andrea Mazzella	NIHR BRC at Guy's and St Thomas' NHS Foundation Trust
Hugh McCaughan	Laboratory For Bacterial Evolution and Pathogenisis (LBEP), The Roslin Institute, University of Edinburgh, UK
Joanne McEwan	Oxford Vaccine Group, Department of Paediatrics, University of Oxford, UK
Joanna McGlashan	National Infection Service, Public Health England, UK
Lorna McInroy	National Infection Service, Public Health England, UK
Zoe McIntyre	NIHR Clinical Research Facility, Cambridge University Hospitals NHS Foundation Trust, UK
Daniella McLenaghan	Department of Clinical Sciences, Liverpool School of Tropical Medicine and Liverpool University Hospitals NHS Foundation Trust, UK
Nicky McRobert	Oxford University Hospitals NHS Trust, Oxford, UK
Steve McSwiggan	Clinical Research Facility Edinburgh, UK
Clare Megson	Oxford University Hospitals NHS Trust, Oxford, UK
Savviz Mehdipour	NIHR Imperial Clinical Research Facility, London, UK
Wilma Meijs	Halix B.V., Tinbergenweg 1, 2333 BB Leiden, Netherlands
Renata N. Á. Mendonça	Rede D'OR São Luiz, Brazil
Alexander J Mentzer	Wellcome Centre for Human Genetics, Nuffield Department of Medicine, University of Oxford, UK
Neginsadat Mirtorabi	University of Oxford Medical School, Medical Sciences Division, University of Oxford, UK
Celia Mitton	Oxford Vaccine Group, Department of Paediatrics, University of Oxford, UK
Sibusiso Mnyakeni	Perinatal HIV Research Unit, Faculty of Health Sciences, University of the Witwatersrand, Johannesburg, South Africa

Fiona Moghaddas	Department of Clinical Immunology, North Bristol NHS Trust, Bristol, UK
Kgaogelo Molapo	Setshaba Research Centre, Pretoria, South Africa
Mapule Moloi	Wits Reproductive Health and HIV Institute, Faculty of Health Sciences, University of the Witwatersrand, Johanesburg, South Africa
Maria Moore	Oxford Vaccine Group, Department of Paediatrics, University of Oxford, UK
M. Isabel De Moraes-Pinto	Universidade Federal de SaoPaulo, Brazil
Marni Moran	Oxford University Hospitals NHS Trust, Oxford, UK
Ella Morey	Oxford Vaccine Group, Department of Paediatrics, University of Oxford, UK
Róisín Morgans	Clinical BioManufacturing Facility, Jenner Institute, University of Oxford, UK
Susan J. Morris	Clinical BioManufacturing Facility, Jenner Institute, University of Oxford, UK
Sheila Morris	Clinical Infection Research Group, Regional Infectious Diseases Unit, NHS Lothian, UK
Helen C. Morris	NIHR Clinical Research Facility, Cambridge University Hospitals NHS Foundation Trust, UK
Franca Morselli	NIHR BRC at Guy's and St Thomas' NHS Foundation Trust, UK
Gertraud Morshead	Oxford Vaccine Group, Department of Paediatrics, University of Oxford, UK
Richard Morter	Jenner Institute, Nuffield Department of Medicine, University of Oxford, UK
Lynelle Mottal	Centre for Lung Infection and Immunity, Division of Pulmonology, Department of Medicine and UCT Lung Institute & South African MRC/UCT Centre for the Study of Antimicrobial Resistance, University of Cape Town, Cape Town, South Africa
Andrew Moultrie	VIDA - Vaccines and Infectious Diseases Analytical Research Unit, Diepkloof, Soweto, South Africa
Nathifa A. Moya	Jenner Institute, Nuffield Department of Medicine, University of Oxford, UK
Mushiya Mpelembue	London Northwest University Healthcare, Northwick Park Hospital, London, UK
Sibekezelo Msomi	Perinatal HIV Research Unit, Faculty of Health Sciences, University of the Witwatersrand, Johannesburg, South Africa
Yvonne N. Mugodi	Perinatal HIV Research Unit, Faculty of Health Sciences, University of the Witwatersrand, Johannesburg, South Africa
Ekta Mukhopadhyay	Jenner Institute, Nuffield Department of Medicine, University of Oxford, UK
Jilly Muller	Oxford Vaccine Group, Department of Paediatrics, University of Oxford, UK
Alasdair P.S. Munro	NIHR Southampton Clinical Research Facility, Southampton, UK
Claire Munro	Oxford Vaccine Group, Department of Paediatrics, University of Oxford, UK
Sarah Murphy	Oxford Vaccine Group, Department of Paediatrics, University of Oxford, UK
Philomena Mweu	Oxford Vaccine Group, Department of Paediatrics, University of Oxford, UK
Celia Hatsuko Myasaki	Universidade Federal de SaoPaulo, Brazil
Gurudutt Naik	Aneurin Bevan University Health Board, Newport, Wales, UK

Kush Naker	NIHR/Wellcome Trust Birmingham Clinical Research Facility, Birmingham, UK
Eleni Nastouli	Clinical Microbiology and Virology Department, University College London Hospitals NHS Trust, UK
Abida Nazir	Clinical Research Facility, Sheffield Teaching Hospitals NHS Foundation Trust, UK
Bongani Ndlovu	Soweto Clinical Trials Centre, Johannesburg, South Africa
Fabio Neffa	Hospital Quinta D'OR, Rede D’OR, São Luiz, Rio De Janeiro, Brazil
Cecilia Njenga	NIHR Imperial Clinical Research Facility, London, UK
Helena C. Noal	Postgraduate Programme in Nursing, Universidade Federal de Santa Maria, Santa Maria, Brazil
Andrés Noé	Jenner Institute, Nuffield Department of Medicine, University of Oxford, UK
Gabrielle Novaes	Hospital Quinta D'OR, Rede D’OR, São Luiz, Rio De Janeiro, Brazil
Fay L Nugent	Jenner Institute, Nuffield Department of Medicine, University of Oxford, UK
Géssika Lanzillo A. Nunes	Centro de Estudos e Pesquisas em Moléstias Infecciosas, Rio Grande do Norte, Brazil
Katie O'Brien	Oxford Vaccine Group, Department of Paediatrics, University of Oxford, UK
Daniel O'Connor	Oxford Vaccine Group, Department of Paediatrics, University of Oxford, UK
Miranda Odam	Emergency Medicine Research Group of Edinburgh (EMERGE), UK
Suzette Oelofse	Centre for Lung Infection and Immunity, Division of Pulmonology, Department of Medicine and UCT Lung Institute & South African MRC/UCT Centre for the Study of Antimicrobial Resistance, University of Cape Town, Cape Town, South Africa
Blanché Oguti	Oxford Vaccine Group, Department of Paediatrics, University of Oxford, UK
Victoria Olchawski	Clinical BioManufacturing Facility, Jenner Institute, University of Oxford, UK
Neil J Oldfield	School of Life Sciences, University of Nottingham, Nottingham, UK
Marianne G. Oliveira	Centro de Estudos e Pesquisas em Moléstias Infecciosas, Rio Grande do Norte, Brazil
Catarina Oliveira	Clinical BioManufacturing Facility, Jenner Institute, University of Oxford, UK
Angela Oosthuizen	Wits Reproductive Health and HIV Institute, Faculty of Health Sciences, University of the Witwatersrand, Johanesburg, South Africa
Paula O'Reilly	Hull University Teaching Hospitals NHS Trust, UK
Piper Osborne	Oxford Vaccine Group, Department of Paediatrics, University of Oxford, UK
David R. J. Owen	NIHR Imperial Clinical Research Facility, London, UK
Lydia Owen	Oxford University Hospitals NHS Trust, Oxford, UK
Daniel R. Owens	NIHR Southampton Clinical Research Facility, Southampton, UK
Nelly Owino	Oxford Vaccine Group, Department of Paediatrics, University of Oxford, UK
Mihaela Pacurar	NIHR Southampton Clinical Research Facility, Southampton, UK

Brenda V. B. Paiva	Instituto D'Or de Ensino e Pesquisa, Rio De Janeiro, Brazil
Edna M. F. Palhares	Centro de Estudos e Pesquisas em Moléstias Infecciosas, Rio Grande do Norte, Brazil
Susan Palmer	Aneurin Bevan University Health Board, Newport, Wales, UK
Sivapriyai Parkinson	Hull University Teaching Hospitals NHS Trust, UK
Helena M. R. T. Parracho	Clinical BioManufacturing Facility, Jenner Institute, University of Oxford, UK
Karen Parsons	Oxford University Hospitals NHS Trust, Oxford, UK
Dipak Patel	Clinical Research and Innovation Office, Sheffield Teaching Hospitals NHS Foundation Trust, UK
Bhumika Patel	Nuffield Department of Surgical Sciences, University of Oxford, UK
Faeezah Patel	Wits Reproductive Health and HIV Institute, Faculty of Health Sciences, University of the Witwatersrand, Johanesburg, South Africa
Kelly Barrett	Women’s Reproductive Health, University of Oxford, UK
Maia Patrick-Smith	University of Oxford Medical School, Medical Sciences Division, University of Oxford, UK
Ruth O. Payne	Department of Infection and Tropical Medicine, Sheffield Teaching Hospitals NHS Foundation Trust and the Department of Infection, Immunity and Cardiovascular Disease, University of Sheffield, UK
Yanchun Peng	MRC Weatherall Institute of Molecular Medicine, NDM Experimental Medicine, University of Oxford, UK
Elizabeth J. Penn	National Infection Service, Public Health England, UK
Anna Pennington	Aneurin Bevan University Health Board, Newport, Wales, UK
Marco Polo Peralta Alvarez	Jenner Institute, Nuffield Department of Medicine, University of Oxford, UK
James Perring	University of Oxford Medical School, Medical Sciences Division, University of Oxford, UK
Nicola Perry	NIHR UCLH Clinical Research Facility, London, UK
Rubeshan Perumal	Centre for Lung Infection and Immunity, Division of Pulmonology, Department of Medicine and UCT Lung Institute & South African MRC/UCT Centre for the Study of Antimicrobial Resistance, University of Cape Town, Cape Town, South Africa
Sahir Yusuf Petkar	Soweto Clinical Trials Centre, Johannesburg, South Africa
Tricia Philip	Perinatal HIV Research Unit, Faculty of Health Sciences, University of the Witwatersrand, Johannesburg, South Africa
Daniel J Phillips	Oxford Vaccine Group, Department of Paediatrics, University of Oxford, UK
Jennifer Phillips	University Hospitals Bristol & Weston NHS Foundation Trust
Mary Kgomotso Phohu	Setshaba Research Centre, Pretoria, South Africa
Lorinda Pickup	NIHR Cambridge Clinical Research Facility, Cambridge, UK
Sonja Pieterse	Family Centre for Research with Ubuntu, Department of Paediatrics, University of Stellenbosch, Cape Town, South Africa
Jo Piper	NIHR Cambridge Clinical Research Facility, Cambridge, UK

Dimitra Pipini	Jenner Institute, Nuffield Department of Medicine, University of Oxford, UK
Mary Plank	AstraZeneca BioPharmaceuticals PLC
Joan Du Plessis	Family Centre for Research with Ubuntu, Department of Paediatrics, University of Stellenbosch, Cape Town, South Africa
Samuel Pollard	Oxford Vaccine Group, Department of Paediatrics, University of Oxford, UK
Jennifer Pooley	North Bristol NHS Trust, Bristol, UK
Anil Pooran	Centre for Lung Infection and Immunity, Division of Pulmonology, Department of Medicine and UCT Lung Institute & South African MRC/UCT Centre for the Study of Antimicrobial Resistance, University of Cape Town, Cape Town, South Africa
Ian Poulton	Jenner Institute, Nuffield Department of Medicine, University of Oxford, UK
Claire Powers	Jenner Institute, Nuffield Department of Medicine, University of Oxford, UK
Fernando B. Presa	Centro de Estudos e Pesquisas em Moléstias Infecciosas, Rio Grande do Norte, Brazil
David A. Price	Department of Infection and Tropical Medicine, Newcastle upon Tyne Hospitals NHS Foundation Trust, UK
Vivien Price	NIHR/Wellcome Trust Birmingham Clinical Research Facility, Birmingham, UK
Marcelo Ribeiro Primeira	Postgraduate Programme in Nursing, Universidade Federal de Santa Maria, Santa Maria, Brazil
Pamela C. Proud	National Infection Service, Public Health England, UK
Samuel Provstgaard-Morys	Oxford Vaccine Group, Department of Paediatrics, University of Oxford, UK
Sophie Pueschel	Oxford University Hospitals NHS Trust, Oxford, UK
David Pulido	Jenner Institute, Nuffield Department of Medicine, University of Oxford, UK
Sheena Quaid	London Northwest University Healthcare, Northwick Park Hospital, London, UK
Ria Rabara	Oxford University Hospitals NHS Trust, Oxford, UK
Alexandra Radford	Clinical Research Facility, Sheffield Teaching Hospitals NHS Foundation Trust, UK
Kajal Radia	University of Oxford Medical School, Medical Sciences Division, University of Oxford, UK
Durga Rajapaksa	National Infection Service, Public Health England, UK
Thurkka Rajeswaran	NIHR BRC at Guy's and St Thomas' NHS Foundation Trust
Alberto San Francisco Ramos	Vaccine Institute, Institute of Infection & Immunity, St. Georges, University of London and St Georges University Hospitals NHS Trust, London, UK
Fernando Ramos Lopez	Jenner Institute, Nuffield Department of Medicine, University of Oxford, UK
Tommy Rampling	Clinical Microbiology and Virology Department, University College London Hospitals NHS Trust, UK
Jade Rand	NIHR Southampton Clinical Research Facility, Southampton, UK
Helen Ratcliffe	Oxford Vaccine Group, Department of Paediatrics, University of Oxford, UK

Thomas Rawlinson	Jenner Institute, Nuffield Department of Medicine, University of Oxford, UK
David Rea	Clinical Research Network - West of England
Byron Rees	Pall Europe Ltd, Harbourgate Business Park, Portsmouth, UK
Jesús Reiné	Department of Clinical Sciences, Liverpool School of Tropical Medicine, UK
Mila Resuello-Dauti	NIHR UCLH Clinical Research Facility, London, UK
Emilia Reyes Pabon	Clinical BioManufacturing Facility, Jenner Institute, University of Oxford, UK
Carla M. Ribiero	NIHR Cambridge Clinical Research Facility, Cambridge, UK
Marivic Ricamara	NIHR UCLH Clinical Research Facility, London, UK
Alex Richter	NIHR/Wellcome Trust Birmingham Clinical Research Facility & Institute of Immunology and Immunotherapy, University of Birmingham, UK
Neil Ritchie	Department of Infectious Diseases, Queen Elizabeth University Hospital, Glasgow, UK
Adam J. Ritchie	Jenner Institute, Nuffield Department of Medicine, University of Oxford, UK
Alexander J. Robbins	NIHR Imperial Clinical Research Facility, London, UK
Hannah Roberts	Oxford Vaccine Group, Department of Paediatrics, University of Oxford, UK
Ryan E Robinson	Department of Clinical Sciences, Liverpool School of Tropical Medicine and Liverpool University Hospitals NHS Foundation Trust, UK
Hannah Robinson	Oxford Vaccine Group, Department of Paediatrics, University of Oxford, UK
Talita T. Rocchetti	Universidade Federal de SaoPaulo, Brazil
Beatriz Pinho Rocha	Universidade Federal de SaoPaulo, Brazil
Sophie Roche	University of Oxford Medical School, Medical Sciences Division, University of Oxford, UK
Christine S Rollier	Oxford Vaccine Group, Department of Paediatrics, University of Oxford, UK
Louisa Rose	Jenner Institute, Nuffield Department of Medicine, University of Oxford, UK
Amy L. Ross Russell	NIHR Southampton Clinical Research Facility, Southampton, UK
Lindie Rossouw	Family Centre for Research with Ubuntu, Department of Paediatrics, University of Stellenbosch, Cape Town, South Africa
Simon Royal	School of Medicine, Division of Primary Care, University of Nottingham, Nottingham, UK
Indra Rudiansyah	Jenner Institute, Nuffield Department of Medicine, University of Oxford, UK
Sarah Ruiz	NIHR BRC at Guy's and St Thomas' NHS Foundation Trust, UK
Stephen Saich	NIHR Southampton Clinical Research Facility, Southampton, UK
Claudia Sala	Hospital Universitário de Santa Maria, Santa Maria, Brazil
Jessica C Sale	NIHR/Wellcome Trust Birmingham Clinical Research Facility, Birmingham, UK
Ahmed M. Salman	Jenner Institute, Nuffield Department of Medicine, University of Oxford, UK
Natalia Salvador	Instituto D'Or de Ensino e Pesquisa, Rio De Janeiro, Brazil
Stephannie Salvador	Jenner Institute, Nuffield Department of Medicine, University of Oxford, UK

Milla Dias Sampaio	Rede D’OR São Luiz, Brazil
Annette D Samson	Hull University Teaching Hospitals NHS Trust, UK
Amada Sanchez-Gonzalez	Department of Infection and Tropical Medicine, Newcastle upon Tyne Hospitals NHS Foundation Trust, UK
Helen Sanders	Jenner Institute, Nuffield Department of Medicine, University of Oxford, UK
Katherine Sanders	Oxford Vaccine Group, Department of Paediatrics, University of Oxford, UK
Erika E. P. D. Santos	Postgraduate Programme in Nursing, Universidade Federal de Santa Maria, Santa Maria, Brazil
Mayara F. S. Guerra	Instituto D'Or de Ensino e Pesquisa, Rio De Janeiro, Brazil
Iman Satti	Jenner Institute, Nuffield Department of Medicine, University of Oxford, UK
Jack E. Saunders	Jenner Institute, Nuffield Department of Medicine, University of Oxford, UK
Caroline Saunders	NIHR Cambridge Clinical Research Facility, Cambridge, UK
Aakifah Bibi Arif Sayed	Perinatal HIV Research Unit, Faculty of Health Sciences, University of the Witwatersrand, Johannesburg, South Africa
Ina Schim van der Loeff	Department of Infection and Tropical Medicine, Newcastle upon Tyne Hospitals NHS Foundation Trust and Translational and Clinical Research Institute, Immunity and Inflammation Theme, Newcastle University
Annina B Schmid	Nuffield Department of Clinical Neurosciences, University of Oxford, UK
Ella Schofield	University of Oxford Medical School, Medical Sciences Division, University of Oxford, UK
Gavin Screaton	Medical Sciences, University of Oxford, UK
Samiullah Seddiqi	Oxford Vaccine Group, Department of Paediatrics, University of Oxford, UK
Rameswara R. Segireddy	Jenner Institute, Nuffield Department of Medicine, University of Oxford, UK
Roberta Senger	Hospital Universitário de Santa Maria, Santa Maria, Brazil
Sonia Serrano	NIHR BRC at Guy's and St Thomas' NHS Foundation Trust
Rajiv Shah	MRC-University of Glasgow Centre for Virus Research & Department of Infectious Diseases, Queen Elizabeth University Hospital, UK
Imam Shaik	National Infection Service, Public Health England, UK
Hannah R. Sharpe	Jenner Institute, Nuffield Department of Medicine, University of Oxford, UK
Katherine Sharrocks	Department of Medicine, University of Cambridge, UK
Robert Shaw	Oxford Vaccine Group, Department of Paediatrics, University of Oxford, UK
Adam Shea	Jenner Institute, Nuffield Department of Medicine, University of Oxford, UK
Amy Shepherd	Clinical Infection Research Group, Regional Infectious Diseases Unit, NHS Lothian, UK
James G. Shepherd	MRC-University of Glasgow Centre for Virus Research & Department of Infectious Diseases, Queen Elizabeth University Hospital, UK
Farah Shiham	Department of Clinical Sciences, Liverpool School of Tropical Medicine and Liverpool University Hospitals NHS Foundation Trust, UK
Emad Sidhom	Windsor Research Unit, Cambridge and Peterborough NHS Foundation Trust, UK

Sarah E. Silk	Jenner Institute, Nuffield Department of Medicine, University of Oxford, UK
Antonio Carlos Da Silva Moraes	Hospital Copa D'OR, Rede D'OR, Rio De Janeiro, Brazil
Gilberto D. A. Silva-Junior	Hospital Quinta D'OR, Rede D'OR, São Luiz, Rio De Janeiro, Brazil
Laura Silva-Reyes	Oxford Vaccine Group, Department of Paediatrics, University of Oxford, UK
Anderson D Silveira	Hospital de Clinicas de Porto Alegre, Universidade Federal do Rio Grande do Sul, Brazil
Mariana B. V. Silveira	Universidade Federal de SaoPaulo, Brazil
Jaisi Sinha	Public Health Wales NHS Trust, Cardiff, UK
Donal T Skelly	Nuffield Department of Clinical Neurosciences, University of Oxford, UK
Catherine C. Smith	Oxford Vaccine Group, Department of Paediatrics, University of Oxford, UK
Daniel C. Smith	Cobra Biologics, Keele Science Park, UK
Nicholas Smith	Cobra Biologics, Keele Science Park, UK
Holly E. Smith	Jenner Institute, Nuffield Department of Medicine, University of Oxford, UK
David J Smith	Oxford Vaccine Group, Department of Paediatrics, University of Oxford, UK
Airanuédida S. Soares	Centro de Estudos e Pesquisas em Moléstias Infecciosas, Rio Grande do Norte, Brazil
Tiago Soares	NIHR BRC at Guy's and St Thomas' NHS Foundation Trust, UK
Carla Solórzano	Department of Clinical Sciences, Liverpool School of Tropical Medicine, UK
Guilherme L. Sorio	Hospital de Clinicas de Porto Alegre, Universidade Federal do Rio Grande do Sul, Brazil
Kim Sorley	NIHR Imperial Clinical Research Facility, London, UK
Tiffany Sosa-Rodriguez	Halix B.V., Tinbergenweg 1, 2333 BB Leiden, Netherlands
Cinthia M. C. D. L. Souza	Centro de Estudos e Pesquisas em Moléstias Infecciosas, Rio Grande do Norte, Brazil
Bruno S. D. F. Souza	Monte Tabor Centro Ítalo Brasileiro de Promoção Sanitária, Fiocruz-BA e I'DOR, Brazil
Alessandra R. Souza	Universidade Federal de SaoPaulo, Brazil
Alexandra J Spencer	Jenner Institute, Nuffield Department of Medicine, University of Oxford, UK
Fernanda Garcia Spina	Universidade Federal de SaoPaulo, Brazil
Louise Spoors	Nuffield Dept of Orthopaedics Rheumatology and Musculoskeletal Medicine, University of Oxford, UK
Lizzie Stafford	Oxford University Hospitals NHS Trust, Oxford, UK
Imogen Stamford	Oxford University Hospitals NHS Trust, Oxford, UK
Igor Starinskij	Department of Infectious Diseases, Queen Elizabeth University Hospital, Glasgow, UK
Ricardo Stein	Hospital de Clinicas de Porto Alegre, Universidade Federal do Rio Grande do Sul, Brazil
Jill Steven	Clinical Research Facility Edinburgh, UK
Lisa Stockdale	Oxford Vaccine Group, Department of Paediatrics, University of Oxford, UK

Lisa V. Stockwell	Oxford Vaccine Group, Department of Paediatrics, University of Oxford, UK
Louise H. Strickland	Nuffield Dept of Orthopaedics Rheumatology and Musculoskeletal Medicine, University of Oxford, UK
Arabella Stuart	Oxford Vaccine Group, Department of Paediatrics, University of Oxford, UK
Ann Sturdy	London Northwest University Healthcare, Northwick Park Hospital, London, UK
Natalina Sutton	Vaccine Institute, Institute of Infection & Immunity, St. Georges, University of London and St Georges University Hospitals NHS Trust, London, UK
Anna Szigeti	Oxford Vaccine Group, Department of Paediatrics, University of Oxford, UK
Abdessamad Tahiri-Alaoui	Clinical BioManufacturing Facility, Jenner Institute, University of Oxford, UK
Rachel Tanner	Jenner Institute, Nuffield Department of Medicine, University of Oxford, UK
Carol Taoushanis	VIDA - Vaccines and Infectious Diseases Analytical Research Unit, Diepkloof, Soweto, South Africa
Alexander W Tarr	School of Life Sciences, University of Nottingham, Nottingham, UK & NIHR Nottingham Biomedical Research Centre, Nottingham University Hospitals NHS Trust, Nottingham, UK
Keja Taylor	Clinical BioManufacturing Facility, Jenner Institute, University of Oxford, UK
Ursula Taylor	Department of Paediatrics, University of Oxford, UK
Iona Jennifer Taylor	Jenner Institute, Nuffield Department of Medicine, University of Oxford, UK
Justin Taylor	Oxford Vaccine Group, Department of Paediatrics, University of Oxford, UK
Rebecca te Water Naude	University of Oxford Medical School, Medical Sciences Division, University of Oxford, UK
Yrene Themistocleous	Jenner Institute, Nuffield Department of Medicine, University of Oxford, UK
Andreas Themistocleous	Nuffield Department of Clinical Neurosciences, University of Oxford, UK
Merin Thomas	Jenner Institute, Nuffield Department of Medicine, University of Oxford, UK
Kelly M Thomas	National Infection Service, Public Health England, UK
Tonia M Thomas	Oxford Vaccine Group, Department of Paediatrics, University of Oxford, UK
Asha Thombrayil	VIDA - Vaccines and Infectious Diseases Analytical Research Unit, Diepkloof, Soweto, South Africa
Fawziyah Thompson	Centre for Lung Infection and Immunity, Division of Pulmonology, Department of Medicine and UCT Lung Institute & South African MRC/UCT Centre for the Study of Antimicrobial Resistance, University of Cape Town, Cape Town, South Africa
Amber Thompson	Oxford Vaccine Group, Department of Paediatrics, University of Oxford, UK
Ameeka Thompson	North Bristol NHS Trust, Bristol, UK
Kevin P Thompson	Pall Europe Ltd, Harbourgate Business Park, Portsmouth, UK
Julia Thomson	AstraZeneca BioPharmaceuticals PLC
Viv Thornton-Jones	Oxford University Hospitals NHS Trust, Oxford, UK

Patrick J Tighe	School of Life Sciences, University of Nottingham, Nottingham, UK
Lygia Accioly Tinoco	Rede D'OR São Luiz, Brazil
Gerlynn Ferreras Tiongson	London Northwest University Healthcare, Northwick Park Hospital, London, UK
Bonolo Tladinyane	Setshaba Research Centre, Pretoria, South Africa
Michele Tomasicchio	Centre for Lung Infection and Immunity, Division of Pulmonology, Department of Medicine and UCT Lung Institute & South African MRC/UCT Centre for the Study of Antimicrobial Resistance, University of Cape Town, Cape Town, South Africa
Adriana Tomic	Oxford Vaccine Group, Department of Paediatrics, University of Oxford, UK
Susan Tonks	Oxford Vaccine Group, Department of Paediatrics, University of Oxford, UK
James Towner	University of Oxford Medical School, Medical Sciences Division, University of Oxford, UK
Nguyen Tran	Jenner Institute, Nuffield Department of Medicine, University of Oxford, UK
Julia A. Tree	National Infection Service, Public Health England, UK
Gerardo Trillana	NIHR BRC at Guy's and St Thomas' NHS Foundation Trust, UK
Charlotte Trinham	NIHR/Wellcome Trust Birmingham Clinical Research Facility, Birmingham, UK
Rose Trivett	Oxford Vaccine Group, Department of Paediatrics, University of Oxford, UK
Adam Truby	Jenner Institute, Nuffield Department of Medicine, University of Oxford, UK
Betty Lebogang Tsheko	Soweto Clinical Trials Centre, Johannesburg, South Africa
Aadil El-Turabi	Jenner Institute, Nuffield Department of Medicine, University of Oxford, UK
Richard Turner	AstraZeneca BioPharmaceuticals PLC
Cheryl Turner	Jenner Institute, Nuffield Department of Medicine, University of Oxford, UK
Marta Ulaszewska	Jenner Institute, Nuffield Department of Medicine, University of Oxford, UK
Benjamin R. Underwood	Windsor Research Unit, Cambridge and Peterborough NHS Foundation Trust, UK
Rachel Varughese	Oxford Vaccine Group, Department of Paediatrics, University of Oxford, UK
Dennis Verbart	Halix B.V., Tinbergenweg 1, 2333 BB Leiden, Netherlands
Marije K Verheul	Oxford Vaccine Group, Department of Paediatrics, University of Oxford, UK
Iason Vichos	Oxford Vaccine Group, Department of Paediatrics, University of Oxford, UK
Taiane A Vieira	Hospital de Clinicas de Porto Alegre, Universidade Federal do Rio Grande do Sul, Brazil
Claire S Waddington	Department of Medicine, University of Cambridge, UK
Laura Walker	Oxford Vaccine Group, Department of Paediatrics, University of Oxford, UK
Erica Wallis	Clinical Research and Innovation Office, Sheffield Teaching Hospitals NHS Foundation Trust, UK
Matthew E Wand	National Infection Service, Public Health England, UK
Deborah Warbrick	Research & Innovation, North Bristol NHS Trust, Bristol, UK

Theresa Wardell	Oxford Biomedica PLC, Transport Way, Cowley, Oxford
George Warimwe	Centre for Tropical Medicine & Global Health, Nuffield Department of Medicine, Oxford, UK
Sarah C. Warren	NIHR Southampton Clinical Research Facility, Southampton, UK
Bridget Watkins	Nuffield Dept of Orthopaedics Rheumatology and Musculoskeletal Medicine, University of Oxford, UK
Ekaterina Watson	London Northwest University Healthcare, Northwick Park Hospital, London, UK
Stewart Webb	Department of Infectious Diseases, Queen Elizabeth University Hospital, Glasgow, UK
Alice Webb-Bridges	Oxford Vaccine Group, Department of Paediatrics, University of Oxford, UK
Andrea Webster	Research Directorate, Newcastle upon Tyne Hospitals NHS Foundation Trust, UK
Jessica Welch	Oxford University Hospitals NHS Trust, Oxford, UK
Jeanette Wells	Aneurin Bevan University Health Board, Newport, Wales, UK
Alison J. West	Oxford University Hospitals NHS Trust, Oxford, UK
Caroline White	Oxford Vaccine Group, Department of Paediatrics, University of Oxford, UK
Rachel White	Oxford Vaccine Group, Department of Paediatrics, University of Oxford, UK
Paul Williams	Clinical BioManufacturing Facility, Jenner Institute, University of Oxford, UK
Rachel Williams	Research & Innovation, North Bristol NHS Trust, Bristol, UK
Rebecca L. Winslow	NIHR/Wellcome Trust Birmingham Clinical Research Facility, Birmingham, UK
Mark Woodyer	Oxford Biomedica PLC, Transport Way, Cowley, Oxford, UK
Andrew T. Worth	Jenner Institute, Nuffield Department of Medicine, University of Oxford, UK
Daniel Wright	Jenner Institute, Nuffield Department of Medicine, University of Oxford, UK
Marzena Wroblewska	Jenner Institute, Nuffield Department of Medicine, University of Oxford, UK
Xin Li Yao	Oxford Vaccine Group, Department of Paediatrics, University of Oxford, UK
Rafael Leal Zimmer	Hospital de Clinicas de Porto Alegre, Universidade Federal do Rio Grande do Sul, Brazil
Dalila Zizi	Clinical BioManufacturing Facility, Jenner Institute, University of Oxford, UK
Peter Zuidewind	Family Centre for Research with Ubuntu, Department of Paediatrics, University of Stellenbosch, Cape Town, South Africa

Acknowledgements

**Advent, South Africa**
Michael Breese
**BioIndustry Association**
Annette England
Ian McCubbin
**Cell & Gene Therapy Catapult**
Stephen Ward
**CobraBio**
Mike Austin
Richard Condliffe
Peter Coleman
Steve Garland
Philip Ridley-Smith
**Clinical Trials Research Governance Office, University of Oxford**
Ronja Bahadori
Elaine Chick
Heather House
Claire Riddle
**Data and Safety Monitoring Board (DSMB)**
George Bouliotis
Steve Black
Elizabeth Bukusi
Cornelia Dekker
Robert Heyderman
Gregory Hussey
Paul Kaye
Bernhards Ogutu
Walter Orenstein
Sonia Ramos
Manish Sadarangani
**Deloitte UK**

Alex Hope
**Department of Health and Social Care, UK Government**
Harry Mayhew
Martin Shanahan
**Department of Paediatrics, University of Oxford**
Joanna Bagniewska
Elizabeth Derow
Georg A. Holländer
Samantha Vanderslott
**Endpoint Evaluation Committee**
Jeremy Carr
Stephen Chambers
Kim Davis
Simon Drysdale
Malick Gibani
Elizabeth Hammershaimb
Michael Harrington
Celina Jin
Seilesh Kadambari
Rama Kandasamy
Toby Maher
Jamilah Meghji
Claire Munro[1]
David Pace
Rekha R. Rapaka
Robindra Basu Roy
Daniel Silman
Gemma Sinclair
Jing Wang
**Halix**
Thijs Booiman
James Harris

[1] Claire Munro joined the Endpoint Evaluation Committee after having previously worked on the trial and having been removed from the delegation log.

Alex Huybens
**The Cambridge NIHR CRF COVID Vaccine Group**
**The GSTT NIHR CRF COVID Vaccine Group**
**The Imperial CRF COVID Vaccine Group**
**Jenner Institute, University of Oxford**
Iona Tarbet
**Nuffield Department of Medicine, University of Oxford**
Joshua Burgoyne
Richard Cornall
Richard Liwicki
Denis Murphy
Elizabeth Salter
Katherine Skinner
Philip Taylor
Oto Velicka
**Oxford Biomedica**
Andy Lewin
James Miskin
Pippa Radcliffe
Jason Slingsby
**Oxford Research Services (Contracts)**
Carly Banner
Sally Pelling-Deeves
Gary Priest
**Oxford University Hospitals NHS Trust**
Monique Andersson
Bruno Holthof
**Pall Europe**

Clive Glover
Peter Levison
**Public Affairs Directorate and Divisional Communication Team**
Alison Brindle
Alexander Buxton
James Colman
Chris McIntyre
Steve Pritchard
**Sartorius**
Zander Hack
**VMIC**
Simon Hoffman
Simon McEwen
Rachael Robbins
