# Supplementary material for: Safety and efficacy of the ChAdOx1 nCoV-19 vaccine (AZD1222) against SARS-CoV-2: an interim analysis of four randomised controlled trials in Brazil, South Africa, and the UK
Source: Lancet. 2021 Jan 9;397(10269):99–111. doi: 10.1016/S0140-6736(20)32661-1 (PMC7723445; doi:10.1016/S0140-6736(20)32661-1)
Supplement: Supplementary appendix [file mmc2.pdf]

THE LANCET

Supplementary appendix

This appendix formed part of the original submission and has been peer reviewed. We post it as supplied by the authors.

Supplement to: Voysey M, Clemens SAC, Madhi SA, et al. Safety and efficacy of the ChAdOx1 nCoV-19 vaccine (AZD1222) against SARS-CoV-2: an interim analysis of four randomised controlled trials in Brazil, South Africa, and the UK. *Lancet* 2020; published online Dec 8. http://dx.doi.org/10.1016/S0140-6736(20)32661-1.

Supplementary Information 2

Global Statistical Analysis Plan for the combined integrated analysis of studies of ChAdOx1 nCoV-19 (AZD1222) vaccine 2

COV001 Protocol 74

COV002 Protocol 184

COV003 Protocol 343

COV005 Protocol 442

Global Statistical Analysis Plan

for the combined integrated analysis of studies of ChAdOx1 nCoV-19 (AZD1222) vaccine

Study	Current Version of Protocol
COV001 (UK)	COV001_Protocol_v12.0_09 Nov_2020 clean
COV002 (UK)	COV002_Protocol_v14.0_09 Nov_2020 clean
COV003 (Brazil)	COV003 ChAdOx1 nCoV-19 Trial protocol V5_20200827_clean
COV005 (South Africa)	ChAdOx1-nCoV-19_ZA_Protocol_v4 1_30Sept2020_signed

	NAME	TITLE	SIGNATURE	DATE
Written by:	Merryn Voysey	Lead Statistician	Merryn Voysey	19 Nov 2020
Approved by:	Andrew Pollard	Chief Investigator	[signature]	19 Nov 2020

Version:	Version Date:	Changes:
1.0	06 Nov 2020	First Draft
1.1	12 Nov 2020	Updated to append MAA SAP – version 4
1.2	19 Nov 2020	Updated to append MAA SAP – version 5

Table of Contents

1 Introduction ii

2 Integrated Summary for Marketing Authorisation Application...1

1 Introduction

This document details the approach that will be used to assess the efficacy and safety of the University of Oxford sponsored studies of ChAdOx1 nCoV-19 vaccine (AZD1222).

The analysis detailed herein has been developed in collaboration with AstraZeneca for the purposes of the marketing authorisation application for the vaccine and does not preclude additional analyses being conducted that are beyond those required for regulatory submissions. Any additional analyses should follow the principles of the analysis detailed in the global SAP to the extent that is possible.

**Statistical Analysis Plan**	
Study Code	D8111
Edition Number	5.0
Date	17 November 2020

Integrated Summary for Marketing Authorisation Application

TABLE OF CONTENTS

TITLE PAGE ... 1

SIGNATURE PAGE ... 2

TABLE OF CONTENTS ... 3

LIST OF ABBREVIATIONS ... 7

AMENDMENT HISTORY ... 9

1 INTRODUCTION ... 11
1.1 Nonclinical Experience with AZD1222 ... 11
1.2 Clinical Experience with AZD1222 ... 11

2. INTEGRATED ANALYSIS OBJECTIVES ... 12
2.1 Primary Objective of the Pooled Analysis ... 12
2.2 Secondary Objectives of the Pooled Analysis ... 12

3. AZD1222 PROTOCOL SUMMARIES ... 13

4. POOLING OF DATA ... 20
4.1 Planned Analyses ... 21

5. ANALYSIS POPULATIONS ... 23

6. DOSING REGIMENS AND TREATMENT GROUPS ... 25

7. OVERVIEW OF ANALYSIS CONVENTIONS ... 26
7.1 Data Presentation ... 26
7.2 Definitions of Subgroups ... 27
7.3 Definition of Baseline ... 27
7.4 Handling of Missing Data ... 27
7.5 Reference Start Date and Study Day ... 28
7.6 Windowing Conventions ... 28

8. SUBJECT ENROLLMENT AND DISPOSITION ... 30
8.1 Subject Identification ... 30
8.2 Subject Disposition ... 30
8.3 Demographics and Baseline Characteristics ... 30
8.4 Exposure ... 31

9. EFFICACY EVALUATIONS ... 31
9.1 Efficacy Assessments ... 31
9.1.1 Primary Efficacy Endpoint ... 32
9.1.1.1 Time to Events Endpoints for Supplementary Analysis ... 33
9.1.2 Secondary Efficacy Endpoints ... 33

9.1.2.1 Incidence of Severe COVID-19 Occurring ≥ 15 Days Post Second Dose of Study Intervention 33
9.1.2.2 Incidence of Severe COVID-19 Occurring Post First Dose of Study Intervention 33
9.1.2.3 Incidence of Severe COVID-19 Occurring ≥ 22 Days Post First Dose of Study Intervention 34
9.1.2.4 Incidence of Asymptomatic SARS-CoV-2 Infection Occurring ≥ 15 Days Post Second Dose of Study Intervention for COV002 Only 34
9.1.2.5 Incidence of Asymptomatic SARS-CoV-2 Infection Occurring ≥ 22 Days Post First Dose of Study Intervention for COV002 Only 34
9.1.2.6 Incidence of SARS-CoV-2 Virologically-confirmed COVID-19 Occurring Post First Dose of Study Intervention 34
9.1.2.7 Incidence of SARS-CoV-2 Virologically-confirmed COVID-19 Occurring 22 Days Post First Dose of Study Intervention 35
9.1.2.8 Duration of Follow-up 35
9.1.2.9 Incidence of COVID-19 Hospital Admission Occurring ≥ 15 Days Post Second Dose of Study Intervention 35
9.1.2.10 Incidence of COVID-19 Hospital Admission Occurring Post First Dose of Study Intervention 35
9.1.2.11 Incidence of COVID-19 Hospital Admission Occurring 22 Days Post First Dose of Study Intervention 35
9.1.2.12 Incidence of COVID-19 Death Occurring ≥ 15 Days Post Second Dose of Study Intervention 36
9.1.2.13 Incidence of COVID-19 Death Associated with SARS-CoV-2 Virologically-confirmed COVID-19 Occurring Post First Dose of Study Intervention 36
9.1.2.14 Incidence of COVID-19 Death Associated with SARS-CoV-2 Virologically-confirmed COVID-19 Occurring 22 Days Post First Dose of Study Intervention 36
9.1.2.15 Incidence of COVID-19 Requiring ICU Occurring ≥ 15 Days Post Second Dose of Study Intervention 36
9.1.2.16 Incidence of COVID-19 Requiring ICU Occurring Post First Dose of Study Intervention 36
9.1.2.17 Incidence of COVID-19 Requiring ICU Occurring ≥ 22 Days Post First Dose of Study Intervention 36
9.1.2.18 Secondary Efficacy Endpoint Based on Nucleocapsid Antibody: Detection of SARS-CoV-2 Nucleocapsid Antibody Levels Over Time 36
9.2 Efficacy Analyses 36
9.2.1 Primary Efficacy Analyses 37
9.2.1.1 Pooled Analysis of Primary Efficacy Endpoint 37
9.2.1.2 Secondary Analysis of Primary Endpoint 38
9.2.1.3 Subgroup Analysis of Primary Endpoint 38
9.2.1.4 Supportive Analyses for Primary Efficacy Endpoint 38
9.2.1.5 Sensitivity Analysis of Primary Endpoint 38
9.2.2 Secondary Efficacy Analyses 38
9.2.2.1 Incidence of Asymptomatic SARS-CoV-2 Infection COV002 only 38
9.2.2.2 Duration of Follow-up since the Second Vaccination 38

9.2.2.3 Other Secondary Efficacy Endpoints 39
9.2.2.4 Secondary Efficacy Analyses for Endpoints Based on Nucleocapsid: Detection AntibodySARS-CoV-2 Nucleocapsid Related Endpoints 39
10. SAFETY ANALYSIS 39
10.1 Adverse Events 39
10.1.1 Reporting of Adverse events 39
10.1.2 Evaluation of Severity 39
10.1.3 Solicited AEs 43
10.1.4 Unsolicited AEs 43
10.1.5 Serious Adverse Events (SAEs) 44
10.1.6 Adverse Events of Special Interest 44
10.1.7 Death 48
10.2 Clinical Laboratory Results 48
10.3 Subgroup Analysis 49
11. IMMUNOGENICITY ANALYSIS 49
12. REFERENCES 51
13. APPENDICES 52

LIST OF APPENDICES

Appendix A Overview of Analyses 53
Appendix B Pooling and Severity Grading of Solicited Adverse Events: Studies COV001, COV002, COV003, and COV005 55
Appendix C Pooling and Severity Grading of Laboratory Assessed on Scheduled Visits 67

LIST OF TABLES

Table 1 Summary of Pivotal Studies to be Pooled 14
Table 2 WHO Clinical Progression Scale 21
Table 3 Populations for Analysis 23
Table 4 Actual AZD1222 Dose Levels Received by CTM and HD or LD Designation 25
Table 5 Visit Window for Immunogenicity 29
Table 6 Case Definitions for Evaluation of Efficacy 32
Table 7 Severity Rating Criteria for Physical Observations (Applies to Adults Only) for COV001, COV002 and COV003 40

Table 8 Severity Grading Criteria for Select Physical Observations (Based on DAIDS Grading Table; Version 2.1 – July 2017 for COV005)................ 40
Table 9 Adverse Events of Special Interest 45
Table A1 Overview of Evaluation of Efficacy 53
Table A2 Overview of Evaluation of Safety 54
Table A3 Overview of Evaluation of Immunogenicity... 54
Table B1 Schedule and Protocol Description of Solicited Adverse Event Reporting in Patient Diaries 56
Table B2 CSP-specified Solicited Adverse Events and Patient Diary Terms for these Events by Study 58
Table B3 Fever Severity Grades for the DMID and DAIDS Grading Scales 60
Table B4 CSP-specified Severity Grading and Patient Diary Measurements for Redness, Swelling, and Induration 61
Table B5 Patient Diary Severity Grades for Pain and Warmth at Injection Site, Malaise, Nausea, and Vomiting Events in Studies COV001, COV002, and COV003 62
Table B6 Patient Diary Severity Grades for Feverishness and Chills in Studies COV001, COV002, and COV003 63
Table B7 Patient Diary Severity Grading for Tenderness, Itching, Joint Pain, Muscle Pain, Fatigue, and Headache by Study 64
Table B8 Contribution of Individual Studies to the Overall Pool for Solicited Adverse Events 65
Table C1 Laboratory Abnormality Severity Grade Criteria to be Used for Pooled COV1, COV002, and COV005 Data... 68
Table C2 Laboratory Abnormality Severity Grade Criteria to be Used for Pooled COV001, COV002, and COV005 Data 70

LIST OF ABBREVIATIONS

Abbreviation or special term	Explanation
AE(s)	adverse event(s)
AESI	adverse event of special interest
BMI	body mass index
CBF	Clinical Biomanufacturing Facility
ChAdOx1 nCoV-19	name of AZD1222 by the University of Oxford
CI	confidence interval
COVID-19	coronavirus disease 2019
CSP	Clinical Study Protocol
CTM	clinical trial material
DAIDS	Division of AIDS
DSMB	Data and Safety Monitoring Board
ELISA	enzyme-linked immunosorbent assay
ELISpot	enzyme-linked immunospot
FDA	Food and Drug Administration
FIH	first-in-human
GMFR	geometric mean fold rise
GMT	geometric mean titre
HIV	human immunodeficiency viruses
IFN-γ	interferon-gamma
IgG	immunoglobulin G
IM	intramuscular
ITT	intent-to-treat
LD	low dose
LLOQ	lower limit of quantification
MAA	Marketing Authorisation Application
MedDRA	Medical Dictionary for Regulatory Activities
MenACWY	meningococcal Group A, C, W-135 and Y conjugate vaccine
MERS	Middle East Respiratory Syndrome
MHRA	Medicines and Healthcare Products Regulatory Agency
MNA	microneutralisation
NAb	neutralising antibody
NHP	non-human primate
NIH	national institutes of health
PRNT	plaque reduction neutralisation test

Abbreviation or special term	Explanation
RBD	receptor binding domain
RR	relative risk
RT-PCR	reverse transcriptase polymerase chain reaction
S	Spike
SAE	serious adverse event
SARS-CoV-2	severe acute respiratory syndrome-coronavirus 2
SD	standard dose
SDSD	standard dose standard dose
Std Dev	standard deviation
tPA	tissue plasminogen activator
UK	United Kingdom
VE	vaccine efficacy
vp	viral particles

AMENDMENT HISTORY

Version 5: Summary of Changes

Category*: Change refers to	Date	Description of change	Rationale
NA	04Sep2020	Not Applicable – First Version.	
Primary or secondary endpoints	02Nov2020	Primary endpoint for COV003 for IA1 was removed; endpoints related to first dose/first dose+22 days were added; the case definition for endpoints were added with WHO severity scoring.	Updated according to response from regulatory agency for planning of interim and primary analysis. Received additional information about adjudication.
Other	02Nov2020	Analysis sets were renamed/modified; overview of analysis was added in the appendix; redefined strategy for subgroups; AESI listing was replaced with updated definition in tables.	Clarify the analysis planned. Update according to new information.
Other	03Nov2020	Remove highlight in TOC; change version to 3.0 from 1.1 in title (skip version 2.0) to match with version in ANGEL. Correct the HIV group in COV002.	Clean the file.
Primary or secondary endpoints	03Nov2020	Update case definition of asymptomatic SARS-CoV-2 infection. Update the homogeneity test may be conducted and change the model of subgroup analysis.	Provide the source of information and reduce parameters in model.
Primary or secondary endpoints	11Nov2020	Added appendix for pooling of solicited events. Clarified COVID-19 infection will be virologically confirmed. Clarified subgroup analyses will be performed for all endpoints, unless specified otherwise.	Clarification of the analyses planned.
Primary or secondary endpoints	17Nov2020	Added appendix for severity grading and pooling of clinical laboratory results. Merged table 10 List of Potential Immune-mediated Medical Conditions into table 9 Adverse Events of special interest. Added clarification of control group for group 2d in COV002. Provided more information of efficacy endpoints in section 4.	Clarification of the analyses planned.

* Pre-specified categories are
Primary or secondary endpoints; Statistical analysis method for the primary or secondary endpoints; Derivation of primary or secondary endpoints; Multiple Testing Procedure; Data presentations; Other

1 INTRODUCTION

AZD1222 is a recombinant replication-defective chimpanzee adenovirus expressing the SARS-CoV-2 S surface glycoprotein driven by the human cytomegalovirus major immediate early promoter that includes intron A with a human tPA leader sequence at the N terminus.

1.1 Nonclinical Experience with AZD1222

AZD1222 has been shown to be immunogenic in BALB/c, CD-1 mice, porcine, and NHP models. Further, in a NHP SARS-CoV-2 challenge model, a single administration of AZD1222 significantly reduced viral load in bronchoalveolar lavage fluid and respiratory tract tissue of vaccinated animals as compared to vector controls (van Doremalen et al 2020). Efficacy studies in ferret and NHP models are in their final completion stage. Biodistribution studies with similar ChAd vaccines (AdCh63 ME-TRAP, AdCh63 MSP-1 and AdCh3NSmut) in mice have previously been performed and showed no evidence of replication of the virus or presence of disseminated infection after IM injections. Given these data, biodistribution studies were not performed based on advice from the MHRA. Toxicology studies have not been conducted on AZD1222. A toxicology study in mice for another ChAdOx1 vectored vaccine expressing a related betacoronavirus S surface antigen (ChAdOx1 MERS) is shown for reference. Further background information is presented in AZD1222 Investigator's Brochure, Section 4.

1.2 Clinical Experience with AZD1222

The University of Oxford is investigating the safety, immunogenicity, and efficacy of AZD1222 in 3 ongoing meningococcal vaccine-controlled clinical studies: a FIH Phase I/II Study COV001 in healthy adults 18 to 55 years of age in the UK; a Phase II/III Study COV002 in healthy adults ≥ 18 years of age (including the elderly) and children 5 to 12 years of age in the UK; and a Phase III Study COV003 in healthy adults ≥ 18 years of age in Brazil. An additional Phase I/II study using a saline placebo control, COV005, is ongoing in South Africa. For these studies, AZD1222 CTM from 3 different sources has been used: 1) The CBF at the University of Oxford; 2) Advent, Italy, and 3) Cobra Biologics. The analytical comparability assessment of AZD1222 Process 1 (CBF), Process 2 (Advent), and Process 3 (Symbiosis) for the clinical Drug Product, was conducted. Due to a potency miscalculation, participants in Groups 1, 2, 4 and 5a in Study COV002 received a lower dose of approximately 2×10^{10} vp instead of the planned dose of 5×10^{10} vp. Groups receiving this lower dose are listed as having received 5×10^{10} vp (Abs260) Advent in the study protocols.

The complete list and description of these ongoing University of Oxford-sponsored AZD1222 clinical studies is presented in Table 1.

Emerging safety and immunogenicity data from all the Oxford sponsored studies are reviewed approximately every 2 weeks by an independent DSMB. Preliminary safety and

immunogenicity data are available for Study COV001, which enrolled the first volunteer on 23 April 2020 and completed enrolment on 21 May 2020. A total of 1 077 volunteers have been enrolled, including 544 volunteers who received at least one dose of 5×10^{10} vp AZD1222 and 10 volunteers who received a second dose of 5×10^{10} vp AZD1222 (homologous prime boost) 4 weeks later. Safety data found the vaccine was generally well tolerated, with no treatmentrelated SAEs reported through 28 days post vaccination. The most common local solicited AEs were vaccination site pain and tenderness. The most common systemic solicited AEs were chills, feverishness, fever, headache, malaise, and myalgia. The majority of events were mild or moderate in severity and resolved within 1 to 7 days. Following the second dose, a general attenuation in the incidence and severity of local and systemic solicited AEs was observed, although this was based on only 10 participants.

Preliminary immunogenicity data from Study COV001 suggest that a single dose can elicit both humoral and cellular immunogenicity responses and that antibody responses are boosted after a second dose. Spike-specific T-cell responses peaked on Day 14. Anti-S IgG responses rose by Day 28 and were boosted 3-fold following a second dose.

Neutralising antibody responses against SARS-CoV-2 were detected in 32 of 35 (91%) participants after a single dose when measured in a microneutralisation assay (MNA_{80}) and in all 35 (100%) participants when measured in a plaque reduction neutralisation test ($PRNT_{50}$) by day 28. After a second dose, all participants had neutralising activity (9 of 9 in MNA_{80} at Day 42 and 10 of 10 in the Marburg virus neutralisation assay on Day 56). Neutralising antibody responses correlated strongly with antibody levels measured by ELISA (Folegatti et al 2020).

2. INTEGRATED ANALYSIS OBJECTIVES

This statistical analysis plan defines methodology and procedures in performing analyses of pooled efficacy and safety data from AZD1222 trials to determine the benefits and risks of AZD1222 as a non-replicating ChAdOx1 vector vaccine in healthy adults against COVID-19.

2.1 Primary Objective of the Pooled Analysis

To estimate the efficacy of 2 IM doses of AZD1222, with the second dose being SD, compared to control for the prevention of COVID-19 in adults ≥ 18 years of age.

2.2 Secondary Objectives of the Pooled Analysis

- To evaluate the efficacy of AZD1222 against severe COVID-19 disease.
- To assess the safety, tolerability and reactogenicity profile of AZD1222.
- To assess humoral immunogenicity of AZD1222 if data are available.
- To assess the cellular immunogenicity of AZD1222 if data are available.

3. AZD1222 PROTOCOL SUMMARIES

Table 1 Summary of Pivotal Studies to be Pooled

Element	COV001	COV002	COV003	COV005
Identifier	NCT04324606;<br>EudraCT 2020-001072-15	NCT04400838;<br>EudraCT 2020-001228-32	ISRCTN89951424	NCT04444674
Title	A phase I/II study to determine efficacy, safety and immunogenicity of the candidate Coronavirus Disease (COVID-19) vaccine ChAdOx1 nCoV-19 in UK healthy adult volunteers	A phase 2/3 study to determine the efficacy, safety and immunogenicity of the candidate Coronavirus Disease (COVID-19) vaccine ChAdOx1 nCoV-19	A Randomized, Controlled, Phase III Study to Determine the Safety, Efficacy, and Immunogenicity of the Non-Replicating ChAdOx1 nCoV-19 Vaccine.	An adaptive phase I/II randomized placebo-controlled trial to determine safety, immunogenicity and efficacy of non-replicating ChAdOx1 SARS-CoV-2 Vaccine in South African adults living without HIV; and safety and immunogenicity in adults living with HIV.
Region	United Kingdom	United Kingdom	Brazil	South Africa
Phase	I/II	II/III	III	I/II
Period	23Apr2020-ongoing	29 May2020-ongoing	Jun2020-ongoing	Jun2020-ongoing
Design	FIH, participant blind, randomised, controlled<br>Participant will be followed 364 days after the last dose	Participant blind, randomised, controlled<br>Participant will be followed 364 days after the last dose except groups 1b, 2b, 5d, 7b and 8b, which will be followed 364 days after the first dose	Participant blind, randomised, controlled<br>Participant will be followed 364 days after the last dose	Double blind, randomised, placebo-controlled, adaptive<br>Participant will be followed 364 days after the first dose
Primary study objective	To assess efficacy of AZD1222 against COVID-19;<br>To assess the safety of AZD1222	To assess efficacy of AZD1222 against COVID-19 in adults aged ≥18 years<br>Co-Primary: To assess the safety of AZD1222 in adults and children.	To evaluate the efficacy of AZD1222 against COVID-19 disease virologically-confirmed[c]	For group 1 and groups 2a and 2b:<br>To assess safety, tolerability and reactogenicity profile of AZD1222;<br>Co-primary objective for groups 2a and 2b:<br>To assess efficacy of AZD1222

Table 1 Summary of Pivotal Studies to be Pooled

Element	COV001	COV002	COV003	COV005
Study population	Healthy adults aged 18-55 years	Main efficacy study: Healthy adults aged ≥18 years<br>Priority given to health professionals and adults with high potential for exposure to SARS-CoV-2<br>Safety and immunogenicity substudies:<br>• Healthy children aged 5 to 12 years, inclusive[a]<br>• HIV+ adults aged 18 - 55 years	Health professionals and adults with high potential for exposure to SARS-CoV-2, aged ≥18 years	Adults aged 18-65 years, living with and without HIV
Actual treatment	AZD1222:<br>2.5×10^{10} vp<br>5×10^{10} vp<br>0.5 mL (3.5 – 6.5×10^{10} vp, Abs 260, corrected for PS80)<br>MenACWY: 0.5 mL	AZD1222:<br>2.2×10^{10} vp (qPCR)<br>2.5×10^{10} vp (qPCR)<br>5×10^{10} vp (Abs 260)<br>5×10^{10} vp (qPCR)<br>0.5 mL (3.5 – 6.5×10^{10} vp, Abs 260, corrected for PS80)<br>MenACWY: 0.5 mL	AZD1222:<br>5×10^{10} vp<br>0. 5mL (3.5 - 6.5×10^{10} vp)<br>MenACWY: 0.5 mL<br>0.9% saline solution: 0.5mL	AZD1222:<br>5×10^{10} vp;<br>Normal saline (0.9% NaCl)

Table 1 Summary of Pivotal Studies to be Pooled

Element	COV001	COV002	COV003	COV005
Efficacy endpoints	**Primary endpoint:** Virologically-confirmed[c] symptomatic cases of COVID-19	**Primary endpoint:** Virologically-confirmed[c] symptomatic cases of COVID-19	**Primary endpoint:** COVID-19 virologically-confirmed[c] symptomatic cases	**Primary endpoint:** Virologically-confirmed[c] COVID-19 cases occurring in participants that were COVID-19 naïve at the time of randomization and who received at least two doses of ChAdOx1 nCoV-19 or placebo. Events will be included if they occurred more than 14 days after the booster dose.

Table 1 Summary of Pivotal Studies to be Pooled

Element	COV001	COV002	COV003	COV005
Efficacy endpoints (continued)	**Secondary endpoints:**<br>a) Hospital admissions associated with COVID-19<br>b) Intensive care unit admissions associated with COVID-19<br>c) Deaths associated with COVID-19<br>d) Severe COVID-19 disease (defined according to clinical severity scales).<br>e) Seroconversion against non-Spike SARS- CoV-2 antigens	**Secondary endpoints:**<br>a) Hospital admissions associated with COVID-19<br>b) Intensive care unit admissions associated with COVID-19<br>c) Deaths associated with COVID-19<br>d) Seroconversion against non-Spike SARS-CoV-2 antigens<br>e) Severe COVID-19 disease (defined according to clinical severity scales)	**Secondary endpoints:**<br>a) Hospitalization for COVID-19 virologically-confirmed[c];<br>b) Severe COVID-19 virologically-confirmed[c];<br>c) Death associated with COVID-19;<br>d) Antibodies against SARS-CoV-2 non-Spike protein (efficacy against non-Spike seroconversion rates)	**Secondary endpoints:**<br>Endpoints in for the overall population and stratified by COVID-19 serological status at randomisation include:<br>a) VE in preventing virologically-confirmed[c] COVID-19; Per-protocol population analysis. Time frame: include all cases occurring onward from 21 days after a single dose or 7 days after a second dose (if a 2-dose schedule was adopted)<br>b) VE in preventing virologically-confirmed[c] COVID-19 cases<br>VE in preventing virologically-confirmed[c] moderate-severe COVID-19<br>c) VE in preventing hospitalization due to virologically-confirmed[c] COVID-19<br>VE in preventing death associated with virologically- confirmed[c] COVID-19<br>d) VE in preventing] all-cause LRTI (overall and stratified by hospitalization or not, irrespective of test result for SARS-COV-2)

Table 1 Summary of Pivotal Studies to be Pooled

Element	COV001	COV002	COV003	COV005
Efficacy endpoints (continued)	**Exploratory endpoints**:<br>N/A	**Exploratory endpoints**:<br>a) Virologically-confirmed[c] SARS-CoV-2 asymptomatic infection<br>b) Differences in viral loads between those with severe, mild, and asymptomatic virologically-confirmed[c] SARS-CoV-2 infections.<br><br>Where possible, sensitivity analyses will be conducted using common alternative definitions of virologically-confirmed COVID-19 disease, including those in use in other Phase 3 protocols (including but not limited to: USA AstraZeneca Phase 3 trial, South Africa COV005 trial, WHO solidarity trial, CEPI definition).	**Exploratory endpoints**:<br>Where possible, sensitivity analyses will be conducted using common alternative definitions of virologically-confirmed[c] COVID-19 disease, including those in use in other Phase 3 protocols (including but not limited to: USA AstraZeneca Phase 3 trial, South Africa COV005 trial, WHO solidarity trial, CEPI definition).	**Exploratory endpoints:**<br>a) VE in preventing death associated with virologically-confirmed[c] COVID-19<br>b) VE in preventing virologically-confirmed[c] COVID-19 or all-cause LRTI requiring supplemental oxygenation<br>c) VE in preventing virologically-confirmed[c] COVID-19 or all-cause LRTI mechanical ventilation<br>d) VE in preventing virologically-confirmed[c] COVID-19 or all-cause LRTI multi-organ dysfunction syndrome<br>e) VE in preventing virologically-confirmed[c] COVID-19 or all-cause LRTI all-cause mortality<br>f) VE in preventing asymptomatic SARS-CoV-2 infection (samples collected at scheduled study visits); ie, no presence of any of the symptoms contributing to COVID-19 outcome, but virologically-confirmed[c] infection<br>g) VE against sero-conversion suggestive of SARS-CoV-2 infection tested using a N-protein IgG assay

Table 1 Summary of Pivotal Studies to be Pooled

Element	COV001	COV002	COV003	COV005
Planned total enrolment	1122	12390	10000	2070
Control	MenACWY	MenACWY	MenACWY	Saline
Number of doses	One or two (based on study group)	One or two (based on study group)	Two	Two
AZD1222 dose levels[b]	Standard and Low	Standard and Low	Standard and standard	Standard and Low
Prophylactic treatment	Paracetamol for a portion of participants	Paracetamol for a portion of participants	Paracetamol systematically	As clinically needed

[a] This group will be enrolled later.
[b] AZD1222 dose levels are defined in Table 4.
[c] Virologically-confirmed from RT-PCR or other nucleic acid amplification test.
COVID = coronavirus disease 2019; FIH = first-in-human; HIV = human immunodeficiency virus; IgG = immunoglobulin G; LRTI = lower respiratory tract infection; MenACWY = meningococcal Group A, C, W-135 and Y conjugate vaccine; N/A = not applicable; PCR = polymerase chain reaction; PS80 = polysorbate 80; qPCR = quantitative polymerase chain reaction; RT-PCR = reverse transcriptase-polymerase chain reaction; SARS-CoV-2 = severe acute respiratory syndrome coronavirus-2; VE = vaccine efficacy; vp = viral particle.
Source: University of Oxford-sponsored study protocols for COV001 version 10.0, COV002 version 12.0, COV003 version 5.0, COV005 version 4.1.
AZD1222 is referred to as ChAdOx1 nCoV-19 in the University of Oxford study protocols.

4. POOLING OF DATA

Despite minor differences across the studies, there is sufficient consistency to justify the proposal for pooled analyses. Some of the differences were driven by input from investigators, funders, and local specificities. The design of studies to be pooled is summarised in Table 1.

The four Oxford-sponsored studies COV001, COV002, COV003, and COV005 are blinded, randomised, controlled studies that were designed to provide robust evidence of efficacy and safety of the AZD1222 candidate vaccine. The designs are compared in Table 1.

All studies enrolled adults 18 to 55. In addition, Studies COV002 (UK, Phase II/III) and COV003 (Brazil, Phase III), have enrolled older adults in age escalation groups of 56 to 69 years of age and ≥ 70 years of age. Enrolment in COV001 was restricted to healthy adults. The other studies allowed the inclusion of people with underlying health conditions with the exception of severe and/or uncontrolled underlying disease. All studies excluded pregnant and breastfeeding women. The safety and immunogenicity of AZD1222 in adults with known HIV infection was specifically investigated in a small subset of participants in Studies COV002 and COV005).

Collection and assessment of data for capture of COVID 19 variables is performed in a consistent manner across the studies. All participants have good access to health care and cases of COVID 19 are detected through passive surveillance systems. A central, blinded adjudication committee is being used by all 4 studies to assess COVID-19 cases from all participants with SARS-CoV-2 virologically positive results. Each case is assessed by the adjudication committee and classified according to the WHO severity grading scale reproduced in Table 2. The adjudicated results are used for the pooled analyses.

Table 2 WHO Clinical Progression Scale

Patient State	Descriptor	Score
Uninfected	Uninfected; no viral RNA detected	0
Ambulatory mild disease	Asymptomatic; viral RNA detected	1
	Symptomatic; independent	2
	Symptomatic; assistance needed	3
Hospitalised: moderate disease	Hospitalised; no oxygen therapy[a]	4
	Hospitalised; oxygen by mask or nasal prong	5
Hospitalised: severe disease	Hospitalised; oxygen by NIV or high flow	6
	Intubation and mechanical ventilation, pO2/FiO2 ≥ 150 or SpO2/FiO2 ≥ 200	7
	Mechanical ventilation pO2/FIO2 < 150 (SpO2/FiO2 <200) or vasopressors	8
	Mechanical ventilation pO2/FiO2 < 150 and vasopressors, dialysis, or ECMO	9
Dead	Dead	10

[a] If hospitalised for isolation only, record status as for ambulatory patient.
ECMO = extracorporeal membrane oxygenation; FiO2 = fraction of inspired oxygen; NIV = non-invasive ventilation; pO2 = partial pressure of oxygen; SpO2 = oxygen saturation.
Source: (WHO et al 2020).

Case definitions for the pooled analysis are given in Table 6. Per protocol in UK studies ICU admission was a protocol defined endpoint. In order to standardise "ICU admission" across trials for differences in local medical practice, this was redefined as "Requiring ICU admission" and corresponds to those WHO severity grades reflecting the need for mechanical ventilation.

Studies COV001, COV002, COV003, and COV005 are all randomised, controlled studies in healthy volunteers with similar efficacy endpoints. Case detection methods for efficacy assessments are also similar. Access to care for passive case detection is available in all studies; COV002 also includes weekly self-swabs for detection of infection and COV005 includes nasal swab and/or saliva on each scheduled visit. Key inclusion criteria are similar for all studies, except for age. Although there are some differences in the exclusion criteria across the studies, study populations are generally similar. All studies excluded patients with serious conditions or receiving medication that could interfere with the study conduct or the interpretation of study data.

4.1 Planned Analyses

The proposed primary and interim pooled analyses provide an opportunity to produce early and robust estimates of efficacy to support rapid decision making in the present conditions of

this public health emergency. One interim analysis and one primary analysis are planned as below:

The interim analysis will be triggered when 53 COVID-19 cases (SARS-CoV-2 virologically confirmed) that occurred ≥ 15 days post the second dose have been reported in participants who received SD/SD (as defined in Table 4) across the AZD1222 and control groups in pooled studies. This would provide 77% power for the 20% threshold to assume a true vaccine efficacy of 70%. The analysis will include participants who received two doses, with a SD as the second dose (ie, participants who received LD/SD or SD/SD). For an individual study to be included in the pooled analysis of efficacy, a minimum of 5 primary endpoint defined cases must be accrued. For COV002, only cases accruing in efficacy study groups will be included (groups 4, 6, 9, 10).

The primary analysis will be triggered when 105 COVID-19 cases (SARS-CoV-2 virologically confirmed) that occurred ≥ 15 days post the second dose have been reported in participants who received SD/SD (as defined in Table 4) across the AZD1222 and control groups. This would provide 90% power for the 20% threshold to assume a true vaccine efficacy of 60%. The analysis will include participants who received two doses, with the second dose being SD (ie, participants who received LD/SD or SD/SD). For an individual study to be included in the pooled analysis of efficacy, a minimum of 5 primary endpoint defined cases must be accrued. For COV002, only cases accruing in efficacy study groups will be included (groups 4, 6, 9, 10).

Gamma Alpha-Spending function is used to control the overall Type 1 Error at 5%. The planned alpha level is 1.13% for interim analysis and 4.44% for primary analysis. The minimum observed VE if an interim or primary analysis demonstrates evidence of efficacy is 64% and 48%, respectively.

At the time of the interim and primary analysis, the safety of AZD1222 as measured by defined safety endpoints or of potential risk in overall pooled data and in subgroups as appropriate will be evaluated. Note that whereas an individual study must accrue 5 cases to be included in the pooled efficacy analysis, all data from COV001, COV002, COV003 and COV005 available at the time of analysis will contribute to the safety analysis if meet the definitions for populations.

Analysis marked for primary manuscript only in this plan will be conducted by Oxford University.

5. ANALYSIS POPULATIONS

Analysis sets for the pooled and interim analyses are defined in Table 3. The groups/participants meeting any of the conditions below will be excluded from all analysis sets (unless otherwise indicated) regardless if they meet the definition in Table 3.

- Groups without randomization (eg, group 3 of COV001, group 11 of COV002);
- Participants previously vaccinated with a ChAdOx1 vectored vaccine (eg, group 11 of COV002);
- Participants with HIV diagnosed at study start (group 3 of COV005 and group 12 of COV002).
- Children (< 18 years old)

Furthermore, only groups evaluated for efficacy in COV002 (ie, groups 4, 6, 9,10) will be considered in populations for efficacy analysis.

The corresponding control group for group 2d in COV001 is group 2e.

All analyses will be performed by actual treatment received. For primary endpoint, a sensitivity analysis by randomized treatment assignment will be performed.

Table 3 Populations for Analysis

Population	Description
All participants analysis set	All participants screened for the studies, to be used for reporting disposition and screening failures.
Any Dose for Safety	All randomized adult participants who received at least 1 dose of study intervention (AZD1222 or control).<br>Participants who withdraw consent or assent to participate in the study will be included up to the date of their study termination.<br>Erroneously-treated participants (eg, those randomized to treatment A but are actually given treatment B) are accounted for in this analysis set by assigning them to the treatment they actually received. A participant who received at least one dose of AZD1222 is classified as AZD1222. This analysis set will be used for safety analysis.
Any Dose for Efficacy	All participants in Any Dose for Safety but for groups in COV002, only efficacy groups (ie, groups 4, 6, 9,10) will be considered.<br>This analysis set will be used for efficacy analysis.
Dose1 SD for Safety	Only participants who received SD as the first dose of AZD1222 or in corresponding control group in Any Dose for Safety. The treatment assignment will follow the same rule of Any Dose for Safety analysis set. This analysis set will be used for safety analysis.

Table 3 Populations for Analysis

Population	Description
Dose1 SD Seronegative for Efficacy	Only participants seronegative at baseline in Any Dose for Safety who received SD as the first dose of AZD1222 or in corresponding control group, and remain on-study 22 days after their first dose without having had a prior SARS-CoV-2 virologically-confirmed[a] COVID-19 infection. In addition, for groups in COV002, only efficacy groups (ie, groups 4, 6, 9,10) will be considered.<br>The treatment assignment will follow the same rule of Any Dose for Safety analysis set. This analysis set will be used for efficacy analysis.
SDSD + LDSD Seronegative for Efficacy	Only participants seronegative at baseline in Any Dose for Safety who received LD/SD or SD/SD or in the corresponding control group, and remain on-study 15 days after their second dose without having had a prior SARS-CoV-2 virologically-confirmed[a] COVID-19 infection. In addition, for groups in COV002, only efficacy groups (ie, groups 4, 6, 9,10) will be considered.<br>The treatment assignment will follow the same rule of Any Dose for Safety analysis set. This analysis set will be used for the efficacy analysis.
SDSD + LDSD Seronegative ITT for Efficacy	Only participants seronegative at baseline in Any Dose for Safety who received two doses, planned to receive LD/SD or SD/SD or in the corresponding control group, and remain on-study 15 days after their second dose without having had a prior SARS-CoV-2 virologically-confirmed[a] COVID-19 infection. In addition, for groups in COV002, only efficacy groups (ie, groups 4, 6, 9,10) will be considered.<br>Participants will be analysed according to their randomized treatment irrespective of whether they have prematurely discontinued, according to the intent-to-treat principle.<br>This analysis set will be used for the sensitivity analysis of primary endpoint.
SDSD Seronegative for Efficacy	Only participants seronegative at baseline in SDSD + LDSD Seronegative for Efficacy analysis set who received SD/SD or in the corresponding control group, and remain on-study 15 days after their second dose without having had a prior SARS-CoV-2 virologically-confirmed[a] COVID-19 infection.<br>The treatment assignment will follow the same rule of Any Dose for Safety analysis set. This analysis set will be used for the efficacy analysis.

Table 3 Populations for Analysis

Population	Description
SDSD + LDSD for Immunogenicity	Only participants in Any dose for Safety who received LD/SD or SD/SD of AZD1222 or in corresponding control group. Participants without at least one post baseline immunogenicity result will be excluded.<br>The treatment assignment will follow the same rule of Any dose for safety analysis set. This population will be used for the immunogenicity analysis.
SDSD for Immunogenicity	Only participants in Any Dose for Safety who received two SDs of AZD1222 or in corresponding control group. Participants without at least one post baseline immunogenicity result will be excluded.<br>The treatment assignment will follow the same rule of Any dose for safety analysis set. This analysis set will be used for immunogenicity analysis.

[a] Virologically-confirmed from RT-PCR or other nucleic acid amplification test.
ITT = intent-to-treat; LD = low dose; RT-PCR = reverse transcriptase-polymerase chain reaction; SD = standard dose.

6. DOSING REGIMENS AND TREATMENT GROUPS

Across the 4 University of Oxford-sponsored studies, participants were randomized to receive a single dose or two doses of either AZD1222, ranging from 2.2 to 5.0×10^{10} vp, or control (as described in Table 4). AZD1222 CTM was sourced from: 1) CBF at the University of Oxford; 2) Advent, Italy, and 3) Cobra Biologics. For control, the MenACWY vaccine was administrated in Studies COV001, COV002, and the first dose of COV003, and 0.9% normal saline (0.9% NaCl) was administered in Study COV005 and the second dose of Study COV003 for participants who received two doses.

Table 4 presents the actual AZD1222 dose levels participants received across the 4 studies by the CTM source and SD or LD designation. For the pooled analyses, a designation of SD or LD was given to each of the AZD1222 dose levels. Generally, 5×10^{10} vp or equivalent is designated as a SD, and 2.2×10^{10} vp or 2.5×10^{10} vp are designated as a LD. Due to a potency miscalculation, participants in Study COV002 groups 1, 2, 4 and 5a who were to receive 5×10^{10} vp (Abs260) Advent as the first dose per protocol, received a lower dose of approximately 2×10^{10} vp (qPCR). Study COV005 groups 1 and 2 also had the LD vaccine.

Table 4 Actual AZD1222 Dose Levels Received by CTM and HD or LD Designation

AZD1222 Dose Level	SD/LD
2.2×10^{10} vp (qPCR) Advent material	LD
2.5×10^{10} vp (qPCR) Advent material	LD

AZD1222 Dose Level	SD/LD
3.5 - 6.5 × 10^{10} vp Cobra material	SD
5 × 10^{10} vp (Abs260) Advent material	LD
5 × 10^{10} vp (qPCR) Advent material	SD
5 × 10^{10} vp Advent material	SD
5 × 10^{10} vp CBF material	SD

CTM = Clinical Trial Material; LD = low dose; SD = standard dose; vp = viral particles.

One source of heterogeneity relates to logistical constraints in the context of the rapid conditions in which this clinical program and scale up manufacturing were initiated in parallel, which led to delays in clinical study material availability for second dose vaccinations in studies to be included in the pooled analyses. This resulted in the fact that, for some participants, the interval between the first and second doses (ie, dose schedule) exceeded 1 month. Dose schedule of two doses will be summarized and homogeneity among different dose schedules will be investigated when needed.

7. OVERVIEW OF ANALYSIS CONVENTIONS

7.1 Data Presentation

The planned 5% alpha will be split across the interim and primary analyses as described in Section 4.1. All efficacy analyses will use a 2-sided alpha test unless otherwise stated. P values will be rounded to 4 decimal places. If a p value is less than 0.0001, it will be reported as "< 0.0001". If a p value is greater than 0.9999, it will be reported as "> 0.9999".

All continuous variables will be summarized using descriptive statistics, reporting N, mean, standard deviation, median, minimum, and maximum. As appropriate, minima and maxima will be reported with the same precision as the raw values; medians and means will have one additional decimal place; standard deviation will have 2 additional decimal places. Data will be displayed by treatment group in all listings, as needed. Participants will be uniquely identified in the listings by the combination of study number, study site number, and subject number.

For discrete, or categorical data, percentages will be suppressed when the count is zero. A row denoted as "Missing" will be included in the count tabulations where necessary to account for dropouts or missing values.

All regulatory analyses will be performed using SAS®, Version 9.4 or higher (SAS Institute Inc., Cary, NC).

7.2 Definitions of Subgroups

To explore the implications for efficacy, safety, and immunogenicity among different populations, the following subgroups will be used:

- Age at randomization;
  - 18-64, 65 years and above
  - 18-55, 56-69, 70 years and above (for efficacy analysis of the primary manuscript only)
- Country (UK, Brazil vs South Africa)
- Comorbidity at baseline (at least one comorbidity vs no comorbidity), where comorbidity is BMI ≥ 30 kg/m^2 at baseline, Cardiovascular Disorder, Respiratory disease or Diabetes.
- Baseline serostatus (seronegative vs seropositive)

The analyses by each subgroup will be performed for all endpoints (efficacy, safety, and immunogenicity) unless specified otherwise.

Additional subgroups which may be explored include but are not limited to:

- Gender (male, female)
- Race (Asian, Black, White, Mixed, Other, Unknown): only categories with at least 100 individuals exposed will be presented
- Use of prophylactic paracetamol (for analysis of reactogenicity)
- Dose level (LD/SD vs SD/SD)
- Dose schedule (< 6 weeks, 6-8 weeks, 9-11 weeks, ≥ 12 weeks)
- Control type (MenACWY, Saline) (for safety only)

7.3 Definition of Baseline

In general, the last non-missing measurement collected prior to the first dose is considered as baseline. If the change from the second dose of study intervention needs be calculated, the last non-missing assessment collected prior to the second dose of study intervention will be considered as baseline.

7.4 Handling of Missing Data

As a general rule, missing data values will not be imputed unless otherwise specified below.

In the summary of AEs by timing relative to each vaccination, if the AE onset date is completely missing, then the AE will be included in summary. If the AE onset date is partially missing, partial AE dates will be handled using the following imputation rules:

- Partial AE start dates where only the year is known:

- If the year is same as the year of dosing:
    - Assume (first dose date + 1 day) if when AE occurred relative to dosing is not available
    - Assume dose date if otherwise
- If the year is not same as the year of dosing, assume January 1 for start date

- Partial AE stop dates where only the year is known: assume December 31 for stop date. If imputed date is greater than the cut-off date of data, use cut-off date instead.
- Partial AE start dates where only the month and year are known:
    - If the year and the month are the same as the year and the month of dosing:
        - Assume (dose date + 1 day) if when AE occurred relative to dosing is not available
        - Assume dose date if otherwise
    - If the year and the month is not same as the year and the month of dosing: assume the first of the month for start date.
- Partial AE stop dates where only the month and year are known: assume the end of the month for stop date

7.5 Reference Start Date and Study Day

Study Day will be calculated from the reference start date and will be used to show start/stop day of assessments and events. Reference start date is defined as the day of the first dose of study drug intervention ie, Day 0.

Study Day will be computed as follows:

- Study Day = (Date of event –Date of first dose of study drug)

In addition, day relative to vaccination will be derived for each vaccination dose. For example, day relative to the first dose will be equal to the Study Day. Day relative to the second dose will start with a value of 0 on the day of the second dose.

7.6 Windowing Conventions

A windowing convention will be used to determine the analysis value for a given study visit for immunogenicity data analyses. The window definitions as following will be used for the immunogenicity.

- A window of ± 14 days from the target day is applied to the following visits: Study Days 28, 56, 90 and 182;
- A window of ± 30 days from the target day is applied to the following visits: Study Days 364;

- A window of ± 42 days from the target day is applied to the following visits: Overall post first dose + 28;

One or more results for a particular immunogenicity variable may be obtained in the same visit window. In such an event, the result with the date closest to the expected visit date will be used in the analysis. In the event that two observations are equidistant from the expected visit date, the later observation will be used in the analysis.

Table 5 Visit Window for Immunogenicity

Dosing Period	Visit	Day Relative to Dose within the Dosing Period[a]	Visit Window (Study Day) Relative to the Dosing Period
Period 1 (Relative to Dose 1)	Baseline[b]	≤ 0	≤ 0
	Day 28	28	14-42
	Day 56	56	42-70
	Day 90	90	76-104
	Day 182	182	168-196
	Day 364	364	334-394
	Overall post first dose + 28	56	14-104
Period 2 (Relative to Dose 2)	Day 28	28	14-42
	Day 56	56	42-70
	Day 90	90	76-104
	Day 182	182	168-196
	Day 364	364	334-394
		**Scheduled Illness Visit**	**Visit Window (Illness Day)**
Illness Visit	Baseline[a]	0	0
	Illness Day 7	7	5-9

[b] For each dosing period, the administration of the study intervention is designated as Study Day 0. For analyses within a period, the study day value is incremented by 1 for each date following the vaccine administration. Dates prior to the vaccine administration are decremented by 1, with the date preceding the vaccine administration designated as Study Day 0.

[c] Where time is available, the time of the collection must be prior to the first dose of study intervention. Day 0 observations taken after the first dose are considered post-baseline values.

[d] For each dosing period, the administration of the study intervention is designated as Study Day 0. For analyses within a period, the study day value is incremented by 1 for each date following the vaccine administration. Dates prior to the vaccine administration are decremented by 1, with the date preceding the vaccine administration designated as Study Day 0.

8. SUBJECT ENROLLMENT AND DISPOSITION

8.1 Subject Identification

Each subject in the pooled analysis will be uniquely identified by the combination of study number, study site number, and subject number.

8.2 Subject Disposition

Disposition will be summarized by treatment group, for overall and each subgroup.

This disposition table of participants will include the number of participants with informed consent, screen failure, in each exclusion criteria from pooled analysis. For each analysis set defined in Section 5, the number and percentage of participants ongoing in study, completed study, discontinued early from study and reasons of discontinuation, and reasons of exclusion in the analysis set will be provided. Only one reason for study discontinuation will be recorded for each discontinued subject.

8.3 Demographics and Baseline Characteristics

Pooled demography and baseline characteristics data will be summarized for Any dose for Safety analysis set, SDSD + LDSD Seronegative for Efficacy analysis set, SDSD Seronegative for Efficacy analysis set, SDSD + LDSD for Immunogenicity analysis set, and SDSD for Immunogenicity by actual treatment.

Demographics:

- Age (years) at randomization
- Age group (18-64 vs ≥ 65 years; 18-55, 56-69 vs ≥ 70 years)
- Gender
- Race (Asian, Black, White, Mixed, Other, Unknown)

Baseline characteristics:

- BMI at baseline (< 30 vs ≥ 30 kg/m^2)
- Serostatus at baseline (Negative, Indeterminate, Positive)
- Cardiovascular Disorder (Yes vs No)
  - Subcategories: chronic heart failure, ischaemic heart disease (including angina), atrial fibrillation, peripheral vascular disease, valvular heart disease, hypertension, myocardial infarction and other
- Respiratory disease (Yes vs No)
  - Subcategories: Chronic obstructive pulmonary disease, Bronchiectasis, Asthma, Other

- Diabetes (Yes, No, Not collected)
  - Subcategories: Type 1 diabetes, Type 2 diabetes not using insulin, Type 2 diabetes using insulin, Other
- Current smoker (Yes vs No)
- Former smoker (Yes, No, Not collected)
- Comorbidity at baseline (at least one comorbidity vs no comorbidity), where comorbidity includes BMI ≥30 kg/m^2 at baseline, Cardiovascular Disorder, Respiratory disease or Diabetes.

Missing due to participant not providing the answer will be presented under category "Missing" while missing due to information not collected in specific study will be presented under category "Not collected".

8.4 Exposure

The exposure information including dose level (ie, SD [only for analysis set based on Dose1 SD], LD/SD, SD/SD), number of dose(s), all available dose schedule for two doses and further categorized dose schedule (< 6 weeks, 6-8 weeks, 9-11 weeks, 12+ weeks) will be summarized by treatment for overall and each study. The summary will be performed for Any Dose for Safety analysis set, Any Dose for Efficacy analysis set, Dose1 SD for Safety analysis set, Dose 1 SD Seronegative for Efficacy analysis set, SDSD + LDSD Seronegative for Efficacy analysis set, SDSD + LDSD for Immunogenicity analysis set, and SDSD for Immunogenicity by actual treatment. The categories for dose schedule may be combined if number of participants are small in some of categories.

A listing of exposure will be provided at the time of the primary analysis.

9. EFFICACY EVALUATIONS

9.1 Efficacy Assessments

Efficacy analysis will be assessed at time planned in Section 4.1. The planned 5% alpha will be split across the interim and primary analyses as described in Section 4.1.

All data from participants with SARS-CoV-2 virologically positive results from RT-PCR or other nucleic acid amplification tests will be assessed by a blinded adjudication committee and the adjudicated endpoints used for all analyses. WHO clinical progression scale (WHO et al 2020) will be utilized to assess the severity of disease. The description of WHO clinical progression scale is in Table 2.

The case for evaluation of efficacy based on adjudicated results are defined as in Table 6.

The overview of the endpoints and corresponding analysis populations is shown in the Appendix A.

Table 6 Case Definitions for Evaluation of Efficacy

Case	Definition
COVID-19 (Primary)<br>Virologically-confirmed[a] symptomatic cases of COVID-19	PCR-confirmed SARS-CoV-2 and at least one of the following symptoms: objective fever (defined as ≥ 37.8 °C), cough, shortness of breath, anosmia, or ageusia. Confirmed by adjudication committee.
COVID-19 Severe Disease	WHO grade ≥ 6[b]
COVID-19 Hospital Admission	WHO grade ≥ 4[b]
COVID-19 Requiring ICU	WHO grade ≥ 7[b]
COVID-19 Death	WHO grade = 10[b]
Asymptomatic SARS-CoV-2 infection	PCR-confirmed SARS-CoV-2 infection and no symptom recorded in data. Confirmed by adjudication committee.

a Virologically-confirmed from RT-PCR or other nucleic acid amplification test.
b WHO clinical progression scale.

9.1.1 Primary Efficacy Endpoint

Incidence of SARS-CoV-2 Virologically-confirmed COVID-19 Occurring ≥ 15 Days Post Second Dose of Study Intervention

The primary efficacy endpoint is the first case of SARS-CoV-2 virologically-confirmed COVID-19 occurring ≥ 15 days post second dose of study intervention. The case is defined as COVID-19 (primary) in Table 6. Only cases with both the sampling date of positive PCR test and COVID-19 symptom(s) onset date ≥ 15 days post second dose will be counted as events. The event date is the date of the PCR test date or date of symptom onset, whichever comes first, and this date will be defined by the endpoint review committee for analysis. For participants with multiple events, only the first occurrence will be used for the primary efficacy endpoint analysis.

The primary efficacy analysis will be based on SDSD + LDSD Seronegative for Efficacy analysis set.

Period at risk for primary endpoint for pooled analysis will be calculated using the reference start as the second dose date + 15 days as Day 1. Calculations for Period at risk are provided below:

Period at risk for primary endpoint = [Last date at risk– (the second dose date + 15 days)] + 1

Where last date at risk will be:

- Date of first event for those diagnosed with SARS-CoV-2 virologically-confirmed COVID-19;
- Date of completion/early discontinuation for participants who complete or discontinue study without event;
- Data cut-off date for analysis for rest of participants who are ongoing without event at the time of analysis.

If the primary endpoint is missing, the period at risk for primary endpoint will be set to missing.

9.1.1.1 Time to Events Endpoints for Supplementary Analysis

Time to First SARS-CoV-2 Virologically-confirmed COVID-19 Occurring ≥ 15 Days Post Second Dose of Study Intervention

Time to First SARS-CoV-2 Virologically-confirmed COVID-19 is defined as the time from the second vaccination + 15 days until the date of the first SARS-CoV-2 virologically-confirmed COVID-19. The calculation is same as the period at risk for the primary endpoint for the pooled analysis. Participants with SARS-CoV-2 virologically-confirmed COVID-19 will be counted as having event while the participants with non-missing value for the period at risk for primary endpoint for pooled analysis will be counted as censored.

Participants with missing value for the period at risk for primary endpoint for pooled analysis will be excluded from analysis.

9.1.2 Secondary Efficacy Endpoints

9.1.2.1 Incidence of Severe COVID-19 Occurring ≥ 15 Days Post Second Dose of Study Intervention

An event is defined as COVID-19 Severe Disease in Table 6, and both the sampling date of positive PCR test and COVID-19 symptom(s) onset date ≥ 15 days post second dose. For participants with multiple events, only the first occurrence will be used for analysis.

9.1.2.2 Incidence of Severe COVID-19 Occurring Post First Dose of Study Intervention

An event is defined as COVID-19 Severe Disease in Table 6, and both the sampling date of positive PCR test and COVID-19 symptom(s) onset date post first dose. For participants with multiple events, only the first occurrence will be used for analysis.

9.1.2.3 Incidence of Severe COVID-19 Occurring ≥ 22 Days Post First Dose of Study Intervention

An event is defined as COVID-19 Severe Disease in Table 6, and both the sampling date of positive PCR test and COVID-19 symptom(s) onset date ≥ 22 days post first dose. For participants with multiple events, only the first occurrence will be used for analysis.

9.1.2.4 Incidence of Asymptomatic SARS-CoV-2 Infection Occurring ≥ 15 Days Post Second Dose of Study Intervention for COV002 Only

An event is defined as Asymptomatic SARS-CoV-2 infection in Table 6 and a PCR-positive sample collected ≥ 15 days post second dose. For participants with multiple events, only the first occurrence will be used for analysis.

The period at risk for SARS-CoV-2 Virologically-confirmed COVID-19 will be calculated similarly to period at risk for the primary endpoint for the pooled analysis replacing information related the primary endpoint for the pooled analysis with information of Asymptomatic SARS-CoV-2 Infection.

The corresponding time to event endpoint is below:

- Time to First Asymptomatic SARS-CoV-2 Infection Occurring ≥ 15 Days Post Second Dose of Study Intervention for COV002 only

9.1.2.5 Incidence of Asymptomatic SARS-CoV-2 Infection Occurring ≥ 22 Days Post First Dose of Study Intervention for COV002 Only

This endpoint will be calculated in the same manner as asymptomatic SARS-CoV-2 infection confirmed by RT-PCR COVID-19 occurring ≥ 15 days post second dose of study intervention for COV002 only (Section 9.1.2.4) by changing the reference date from date of 15 Days post second dose to date of 22 days post first dose and the analysis set will follow Appendix A.

The corresponding time to event endpoint is below:

- Time to Asymptomatic SARS-CoV-2 Infection Confirmed by RT-PCR COVID-19 Occurring ≥ 22 Days Post First Dose of Study Intervention for COV002 only

9.1.2.6 Incidence of SARS-CoV-2 Virologically-confirmed COVID-19 Occurring Post First Dose of Study Intervention

This endpoint will be calculated in the same manner as primary endpoint (Section 9.1.1) by changing the reference date from date of 15 Days post second dose to the first dose date and the analysis will be performed on Any dose for efficacy analysis set.

The corresponding time to event endpoint is below:

- Time to First SARS-CoV-2 Virologically-confirmed COVID-19 Occurring Post First Dose of Study Intervention

9.1.2.7 Incidence of SARS-CoV-2 Virologically-confirmed COVID-19 Occurring 22 Days Post First Dose of Study Intervention

This endpoint will be calculated in the same manner as primary endpoint (Section 9.1.2.1) by changing the reference date from date of 15 Days post second dose to date of 22 days post first dose and the analysis will be performed on Dose1 SD for efficacy analysis set.

The corresponding time to event endpoint is below:

- Time to First SARS-CoV-2 Virologically-confirmed COVID-19 Occurring 22 Days Post First Dose of Study Intervention

9.1.2.8 Duration of Follow-up

For participants in the pooled analysis, the duration of follow-up since the first dose, 22 days post the first dose and 15 days post the second dose will be calculated respectively as study completion/discontinuation date - reference date +1. If subject did not discontinue from the study at the time of the analysis, the data cut-off date will be used. The duration will be summarized by treatment group.

9.1.2.9 Incidence of COVID-19 Hospital Admission Occurring ≥ 15 Days Post Second Dose of Study Intervention

An event is defined as COVID-19 Hospital Admission in Table 6 and with both admission date of hospitalization and onset date of associated COVID-19 are ≥ 15 days post second dose of study intervention.

9.1.2.10 Incidence of COVID-19 Hospital Admission Occurring Post First Dose of Study Intervention

An event is defined as COVID-19 Hospital Admission in Table 6 and with both admission date of hospitalization and onset date of associated COVID-19 are post first dose of study intervention.

9.1.2.11 Incidence of COVID-19 Hospital Admission Occurring 22 Days Post First Dose of Study Intervention

An event is defined as COVID-19 Hospital Admission in Table 6 and with admission date of hospitalization and onset date of associated COVID-19 are ≥ 22 days post first dose of study intervention.

9.1.2.12 Incidence of COVID-19 Death Occurring ≥ 15 Days Post Second Dose of Study Intervention

An event is defined as COVID-19 Death in Table 6 and both death date and onset date of associated COVID-19 are ≥ 15 days post second dose of study intervention.

9.1.2.13 Incidence of COVID-19 Death Associated with SARS-CoV-2 Virologically-confirmed COVID-19 Occurring Post First Dose of Study Intervention

An event is defined as COVID-19 Death in Table 6 and with both death date and onset date of associated COVID-19 are post first dose of study intervention.

9.1.2.14 Incidence of COVID-19 Death Associated with SARS-CoV-2 Virologically-confirmed COVID-19 Occurring 22 Days Post First Dose of Study Intervention

An event is defined as COVID-19 Death in Table 6 and with both death date and onset date of associated COVID-19 are ≥ 22 days post first dose of study intervention.

9.1.2.15 Incidence of COVID-19 Requiring ICU Occurring ≥ 15 Days Post Second Dose of Study Intervention

An event is defined as COVID-19 Requiring ICU in Table 6 and with both admission date and onset date of associated COVID-19 are ≥ 15 days post second dose of study intervention.

9.1.2.16 Incidence of COVID-19 Requiring ICU Occurring Post First Dose of Study Intervention

An event is defined as COVID-19 Requiring ICU in Table 6 and with both admission date and onset date of associated COVID-19 are post first dose of study intervention.

9.1.2.17 Incidence of COVID-19 Requiring ICU Occurring ≥ 22 Days Post First Dose of Study Intervention

An event is defined as COVID-19 Requiring ICU in Table 6 and with both admission date and onset date of associated COVID-19 are ≥ 22 days post first dose of study intervention.

9.1.2.18 Secondary Efficacy Endpoint Based on Nucleocapsid Antibody: Detection of SARS-CoV-2 Nucleocapsid Antibody Levels Over Time

If data available, the detection of SARS-COV-2 nucleocapsid antibody will be summarised as proportion of participants who have a post-treatment response (negative at baseline to positive post treatment with study intervention) to SARS-CoV-2 Nucleocapsid antibodies. This will be summarized by study arm, by visit, overall and by PCR result based on analysis sets for immunogenicity. The window conventions defined in Table 5 will apply to this variable.

9.2 Efficacy Analyses

The efficacy analyses will be conducted in the analysis sets as planned in Table A1Table **3**, unless otherwise specified.

9.2.1 Primary Efficacy Analyses

9.2.1.1 Pooled Analysis of Primary Efficacy Endpoint

A Poisson regression model with robust variance (Zou 2004) will be used as the primary efficacy analysis model to estimate the relative risk (RR) of the incidence of SARS-CoV-2 virologically-confirmed primary symptomatic COVID-19 between the AZD1222 and control groups. The model contains the term of study code, treatment group, and age group at randomization (ie, 18-55 years, 56-69 years, and ≥ 70 years). The logarithm of the period at risk for primary endpoint for pooled analysis will be used as an offset variable in the model to adjust for volunteers having different follow up times during which the events occur.

Vaccine efficacy (VE), which is the incidence of infection in the vaccine group relative to the incidence of infection in the control group expressed as a percentage, will be calculated as VE = 1- relative risk. The VE, and its corresponding 2-sided (1-α) % confidence interval (CI), will be estimated from the model. In addition, the 2-sided p value testing null hypothesis that the incidence of SARS-CoV-2 virologically-confirmed primary symptomatic COVID-19 between AZD1222 and control groups are the same will be obtained from the model. Statistical significance will be achieved if the 2-sided p value is ≤ α, where α for interim and primary analysis as descripted in Section 4.1.

The Poisson regression with robust variance analysis will be implemented by using the SAS PROC GENMOD procedure for binary data with the REPEATED statement for subject ID and logarithm link. The estimated parameter $\hat{\beta}$ [ie, log ($\widehat{RR}$)], 2-sided (1-α) % CI for $\hat{\beta}$, and the 2-sided p value will be obtained from the SAS outputs. The estimated RR and corresponding CI for the RR is given by exponentiating $\hat{\beta}$ and its confidence limits. Therefore, the percent of VE is given by [(1 – exp ($\hat{\beta}$)) * 100%]. The CI for the percent of VE is given by ([1 – exp(upper confidence limit for $\hat{\beta}$) * 100%], [1 – exp(lower confidence limit for $\hat{\beta}$) * 100%]).

If the Poisson regression model with robust variance fails to converge, the exact conditional method for stratified Poisson regression using PROC GENMOD with the exact statement will be used.

To investigate if the pooled studies are homogeneous, a study-by-treatment interaction term may be included in the Poisson regression model as a factor and the type III p value for this term will be presented. If the p value is greater than or equal to 0.05, the study-by-treatment interaction term may be dropped from the model for presentation of summary analysis results. Same method may be performed for dose schedule (< 6 weeks, 6-8 weeks, 9-11 weeks, 12+ weeks), and dose pattern (LD/SD, SD/SD) if further exploration is warranted.

9.2.1.2 Secondary Analysis of Primary Endpoint

The analyses for the primary endpoint for the pooled analysis will be repeated for participants who received two SDs of vaccine (ie, SDSD Seronegative for Efficacy analysis set).

9.2.1.3 Subgroup Analysis of Primary Endpoint

Subgroup analysis will be performed for the primary efficacy endpoint, the incidence of SARS-CoV-2 virologically-confirmed COVID-19. Within each level of a subgroup, the VE and its corresponding (1-α) % CI will be estimated using a Poisson regression model with robust variance with the term of treatment. A forest plot of the VE and the (1-α) % CI will be presented. If the Poisson regression model does not converge for any stratum of a subgroup, the exact conditional method for stratified Poisson regression using PROC GENMOD with the exact statement will be used.

9.2.1.4 Supportive Analyses for Primary Efficacy Endpoint

To support the primary analysis, a Cox Proportional Hazards model using the same covariates as for the primary analyses as well as Kaplan-Meier curves will be presented for the active and control groups based on observed events, showing the cumulative incidence of the first case of SARS-CoV-2 RT-PCR-positive symptomatic illness occurring ≥ 15 days post second dose of study intervention. Time to event, i.e., the duration in days since 15 days post second study dose to event or censoring, will be fit using the PH model with treatment as a factor and age group, country as stratum. Hazards ratios for each study arm along with the two-sided (1-α) % CI will be obtained from the PH model. The number of participants with a primary endpoint and the number of censored participants will also be provided. The censoring timing at each month will be displayed.

9.2.1.5 Sensitivity Analysis of Primary Endpoint

The analyses for the primary endpoint for the pooled analysis will be repeated for participants who received two SDs of vaccine and the second dose is SD based on randomized treatment (ie, SDSD + LDSD Seronegative ITT for Efficacy analysis set).

9.2.2 Secondary Efficacy Analyses

9.2.2.1 Incidence of Asymptomatic SARS-CoV-2 Infection COV002 only

The analysis for those endpoints will be conducted in a similar manner as described in Section 9.2.1.1 (see Pooled Analysis of Primary Efficacy Endpoint) but remove Study code from model.

9.2.2.2 Duration of Follow-up since the Second Vaccination

Summary by treatment group will be calculated for this variable.

9.2.2.3 Other Secondary Efficacy Endpoints

The remaining endpoints will be analysed in the same manner as the pooled analysis of the primary efficacy endpoint as described in Section 9.2.1.1 (see Pooled Analysis of Primary Efficacy Endpoint). VE will not be computed unless 5 cases have accrued for the respective endpoint, but the distribution by groups will be presented.

9.2.2.4 Secondary Efficacy Analyses for Endpoints Based on Nucleocapsid: Detection AntibodySARS-CoV-2 Nucleocapsid Related Endpoints

Percentage of participants positive for SARS-CoV-2 nucleocapsid antibody levels over time will be summarized overall and by baseline seropositivity status. At each time point the % positive for nucleocapsid antibody will be summarized for those who were not previously positive at any time point and in those who were previously positive at least one time point.

10. SAFETY ANALYSIS

Safety summaries described below will be performed by treatment group (AZD1222 and Control) for analysis sets planned in Table A2.

10.1 Adverse Events

10.1.1 Reporting of Adverse events

All local and systemic AEs that occur within 28 days after each vaccination observed by the Investigator or reported by the participant, will be recorded by the participants in the Diary of Symptoms and by the Investigators in the study CRF. For COV001, diary cards for the second vaccines will not be filled out for participants in groups 2f, 2g, 4c and 4d. For COV002, solicited and unsolicited AEs will be reported for 7 days only for group 1-3, 5, 7, 8, 11 and 12 and a subset of up to 3000 participants for groups 4, 6, 9, and 10. In COV003, solicited AEs will be reported for a subset of 200 participants.

SAEs and Adverse Events of Special Interest will be collected throughout the study period.

Adverse events will be coded using the Medical Dictionary for Regulatory Activities (MedDRA) Version 23 or higher.

10.1.2 Evaluation of Severity

The severity of AEs will be assessed according to toxicity rating scales adapted from the FDA for healthy volunteers recruited in preventive vaccine clinical trials, listed in the specific study work instructions and Table 7. For COV005, DAIDS grading the severity of adult and paediatric adverse events is used. For solicited AE, refer to Appendix B.

Table 7 Severity Rating Criteria for Physical Observations (Applies to Adults Only) for COV001, COV002 and COV003

Vital Signs	Grade 1 (mild)	Grade 2 (moderate)	Grade 3 (serious)	Grade 4 Potentially fatal
Fever (oral)	38, 0°C- 38, 4°C	38.5°C - 38.9°C	39.0°C - 40°C	> 40°C
Tachycardia (bpm)[a]	101 – 115	116-130	> 130	A/E visit or hospitalization for arrhythmia
Bradycardia (bpm)[b]	50 – 54	45 – 49	< 45	A/E visit or hospitalization for arrhythmia
Systolic hypertension (mmHg)	141 -150	151 – 155	≥ 155	A/E visit or hospitalization for malignant hypertension
Diastolic hypertension (mmHg)	91 – 95	96 – 100	> 1100	A/E visit or hospitalization for malignant hypertension
Systolic hypotension (mmHg)[c]	85 – 89	80 – 84	< 80	A/E visit or hospitalization for hypotensive shock
Respiratory Rate - breaths per minute	17 – 20	21-25	> 25	Intubation

[a] Measured after ≥10 minutes at rest

[b] When the resting heart rate is between 60 to 100 beats per minute. Use the clinical criterion when characterizing bradycardia among some populations of healthy participants, for example, conditioned athletes.

[c] Only if symptomatic (for example, dizziness/vertigo)

A/E = accident & emergency department.

Table 8 Severity Grading Criteria for Select Physical Observations (Based on DAIDS Grading Table; Version 2.1 – July 2017 for COV005)

Vital signs	Grade 1 (mild)	Grade 2 (moderate)	Grade 3 (severe)	Grade 4 Potentially life threatening
	Mild symptoms causing no or minimal interference with usual social & functional activities with intervention not indicated	Moderate symptoms causing greater than minimal interference with usual social & functional activities with intervention indicated	Severe symptoms causing inability to perform usual social & functional activities with intervention or hospitalization indicated	Potentially life-threatening symptoms causing inability to perform basic self-care functions with intervention indicated to prevent permanent impairment, persistent disability, or death

Table 8 Severity Grading Criteria for Select Physical Observations (Based on DAIDS Grading Table; Version 2.1 – July 2017 for COV005)

Vital signs	Grade 1 (mild)	Grade 2 (moderate)	Grade 3 (severe)	Grade 4 Potentially life threatening
Arthralgia	Joint pain causing no or minimal interference with usual social & functional activities	Joint pain causing greater than minimal interference with usual social & functional activities	Joint pain causing inability to perform usual social & functional activities	Disabling joint pain causing inability to perform basic self-care functions
Arthritis	Stiffness or joint swelling causing no or minimal interference with usual social & functional activities	Stiffness or joint swelling causing greater than minimal interference with usual social & functional activities	Stiffness or joint swelling causing inability to perform usual social & functional activities	Disabling joint stiffness or swelling causing inability to perform basic self-care functions
Chills	Symptoms causing no or minimal interference with usual social & functional activities	Symptoms causing greater than minimal interference with usual social & functional activities	Symptoms causing inability to perform usual social & functional activities	NA
Fatigue or Malaise *Report only one*	Symptoms causing no or minimal interference with usual social & functional activities	Symptoms causing greater than minimal interference with usual social & functional activities	Symptoms causing inability to perform usual social & functional activities	Incapacitating symptoms of fatigue or malaise causing inability to perform basic self-care functions
Fever (non-axillary temperatures only)	38.0 to < 38.6°C	≥ 38.6 to < 39.3°C	≥ 39.3 to < 40.0°C	≥ 40.0°C or ≥ 104.0°F
Headache	Symptoms causing no or minimal interference with usual social & functional activities	Symptoms causing greater than minimal interference with usual social & functional activities	Symptoms causing inability to perform usual social & functional activities	Symptoms causing inability to perform basic self-care functions OR Hospitalization indicated OR Headache with significant impairment of alertness or other neurologic function

Table 8 Severity Grading Criteria for Select Physical Observations (Based on DAIDS Grading Table; Version 2.1 – July 2017 for COV005)

Vital signs	Grade 1 (mild)	Grade 2 (moderate)	Grade 3 (severe)	Grade 4 Potentially life threatening
Myalgia (generalized)	Muscle pain causing no or minimal interference with usual social & functional activities	Muscle pain causing greater than minimal interference with usual social & functional activities	Muscle pain causing inability to perform usual social & functional activities	Disabling muscle pain causing inability to perform basic self-care functions
Pain (not associated with study agent injections and not specified elsewhere) *Specify location*	Pain causing no or minimal interference with usual social & functional activities	Pain causing greater than minimal interference with usual social & functional activities	Pain causing inability to perform usual social & functional activities	Disabling pain causing inability to perform basic self-care functions OR Hospitalization indicated
Acute Allergic Reaction	Localized urticaria (wheals) with no medical intervention indicated	Localized urticaria with intervention indicated OR Mild angioedema with no intervention indicated	Generalized urticaria OR Angioedema with intervention indicated OR Symptoms of mild bronchospasm	Acute anaphylaxis OR Life-threatening bronchospasm OR Laryngeal edema
Blood Pressure Abnormalities Hypertension (with the lowest reading taken after repeat testing during a visit) ≥ 18 years of age	140 to < 160 mmHg systolic OR 90 to < 100 mmHg diastolic	≥ 160 to <180 mmHg systolic OR ≥ 100 to < 110 mmHg diastolic	≥ 180 mmHg systolic OR ≥ 110 mmHg diastolic	Life-threatening consequences in a participant not previously diagnosed with hypertension (eg, malignant hypertension) OR Hospitalization indicated
Hypotension	No symptoms	Symptoms corrected with oral fluid replacement	Symptoms AND IV fluids indicated	Shock requiring use of vasopressors or mechanical assistance to maintain blood pressure

Table 8 Severity Grading Criteria for Select Physical Observations (Based on DAIDS Grading Table; Version 2.1 – July 2017 for COV005)

Vital signs	Grade 1 (mild)	Grade 2 (moderate)	Grade 3 (severe)	Grade 4 Potentially life threatening
Dyspnea or Respiratory Distress *Report only one*	Dyspnea on exertion with no or minimal interference with usual social & functional activities OR Wheezing OR Minimal increase in respiratory rate for age	Dyspnea on exertion causing greater than minimal interference with usual social & functional activities OR Nasal flaring OR Intercostal retractions OR Pulse oximetry 90 to < 95%	Dyspnea at rest causing inability to perform usual social & functional activities OR Pulse oximetry < 90%	Respiratory failure with ventilator support indicated (eg, CPAP, BPAP, intubation)

10.1.3 Solicited AEs

Diary cards will collect the timing and severity of predefined Solicited AEs listed in Appendix B. Solicited local AEs and solicited systemic AEs collected for 7 days (Day 0 to Day 6 for COV005 while Day 0 to Day 7 for rest of studies) after vaccination for each dose will be summarized respectively. The strategy for pooling the data across different studies is provided in Appendix B.

Each solicited AE will be summarized by treatment group at the following time intervals: Days 0-7, and Days 0 to day 7 individually (for PA only). Each time interval will be repeated for after dose 1, after dose 2 and after any dose. For each time interval, the count and percentage of participants will be determined for each of the following categories: participants evaluated, participants with any event, mild events, moderate events, severe events, and potentially life-threatening events. Participants should not be double counted; therefore, the event of greatest severity will be used for participants with more than one episode of the same event. Similar counts and percentages will be presented for solicited local AEs "Overall" and solicited systemic AEs "Overall". For participants evaluated for diary cards, missing values will be treated as missing in the calculation.

A listing of all solicited AEs will be provided at the time of the primary analysis.

10.1.4 Unsolicited AEs

All AEs are unsolicited AEs unless categorized as solicited AEs. Unsolicited AEs from the start of each dose through 28 days (ie, day of vaccination and the following 27 days) will be summarized. Summaries will be produced of:

- Number of participants with unsolicited AEs by treatment group, system organ class and preferred term
- Number of participants with unsolicited AEs, assessed by investigator as possibly related to investigational product, by treatment, system organ class and preferred term
- Number of participants with unsolicited AEs ≥x% in either treatment group, by treatment and preferred term where x may be 1, 3 or 5

In the summary of number of participants, should a subject experience multiple events within a SOC or PT, the subject will be counted only once for that SOC or PT.

A listing of all unsolicited AEs will be provided at the primary analysis.

10.1.5 Serious Adverse Events (SAEs)

SAEs are those events recorded as "Serious" on the AE page of the eCRF. SAEs following the first vaccination to 364 days after the last vaccination will be summarized. Summaries will be produced of:

- Number of SAEs by treatment group, Medical Dictionary for Regulatory Activities (MedDRA) system organ class and preferred term
- Number of participants with SAEs by treatment group, system organ class and preferred term
- Number of participants with SAEs, assessed by investigator as possibly related to investigational product, by treatment group, system organ class and preferred term
- Number of participants with SAEs ≥ x% in either treatment group, by treatment and preferred term where x may be 1, 3 or 5

In the summary of number of participants, should a subject experience multiple events within a SOC or PT, the subject will be counted only once for that SOC or PT.

A listing of all SAEs including those prior to the first vaccination will be provided at the time of the primary analysis.

10.1.6 Adverse Events of Special Interest

AZD1222 AESIs are based on Brighton Collaboration case definitions (SPEAC 2020), clinical experience, and scientific interest. There is no current evidence to suggest that AZD1222 is associated with these AESIs.

Table 9 Adverse Events of Special Interest

AESI	Medical Concept
Neurologic	Generalized convulsion: Seizures are episodes of neuronal hyperactivity most commonly resulting in sudden, involuntary muscular contractions. They may also manifest as sensory disturbances, autonomic dysfunction and behavioral abnormalities, and impairment or loss of consciousness.
	Other neurologic events: These events would include new onset event (acute or subacute) motor and sensory disturbances (eg, weakness, numbness, paresthesias, hypoesthesia, hyperesthesia, dysesthesias), bowel/bladder dysfunction, gait impairment, or visual disturbance, or other sudden neurological deficit.
Vascular	Thrombotic, thromboembolic, and neurovascular events: These are events that can manifest as transient or permanent vision problems, dizziness, trouble understanding, facial droop, slurred speech, unilateral weakness, deep vein thrombosis with swollen, warm or painful leg, pulmonary embolism with shortness of breath, chest pain or irregular heart rate
Hematologic	Thrombocytopenia: Thrombocytopenia is a disorder in which there is an abnormally low platelet count; a normal platelet count ranges from 150 000 to 450 000 platelets per μL.
Immunologic	Vasculitides: Vasculitides are a group of related disorders characterized by inflammation of blood vessels (vasculitis) leading to tissue or end-organ injury.
	Anaphylaxis: Anaphylaxis an acute hypersensitivity reaction with multi-organ-system involvement that can present as, or rapidly progress to, a severe life-threatening reaction requiring immediate medical attention.
	Vaccine-associated enhanced respiratory disease: The pathogenicity of VAERD has been linked to a vaccine immune response characterized by induction of non-neutralizing antibodies, and a T-cell response of the Th2 type with hypereosinophilia (Lambert et al 2020). VAERD may manifest as a severe form of respiratory disease with prolonged fever, and diverse clinical manifestations of disease severity and pathological changes marked by increased areas of lung consolidation, broncho-interstitial pneumonia, and necrotizing bronchiolitis (Rajão et al 2016).
	Potential immune-mediated conditions: These conditions are a group of autoimmune inflammatory disorders characterized by an alteration in cellular homeostasis, which may or may not have an autoimmune aetiology. A list of events is provided below<br>• Gastrointestinal disorders<br>○ Celiac disease<br>○ Crohn's disease<br>○ Ulcerative colitis<br>○ Ulcerative proctitis

AESI	Medical Concept
	• Liver disorders<br>o Autoimmune cholangitis<br>o Autoimmune hepatitis<br>o Primary biliary cirrhosis<br>o Primary sclerosing cholangitis<br><br>• Metabolic diseases<br>o Addison's disease<br>o Autoimmune thyroiditis (including Hashimoto thyroiditis)<br>o Diabetes mellitus type I<br>o Grave's or Basedow's disease<br><br>• Musculoskeletal disorders<br>o Antisynthetase syndrome<br>o Dermatomyositis<br>o Juvenile chronic arthritis (including Still's disease)<br>o Mixed connective tissue disorder<br>o Polymyalgia rheumatic<br>o Polymyositis<br>o Psoriatic arthropathy<br>o Relapsing polychondritis<br>o Rheumatoid arthritis<br>o Scleroderma, including diffuse systemic form and CREST syndrome<br>o Spondyloarthritis, including ankylosing spondylitis, reactive arthritis<br>o (Reiter's Syndrome) and undifferentiated spondyloarthritis<br>o Systemic lupus erythematosus<br>o Systemic sclerosis

AESI	Medical Concept
	• Neuroinflammatory disorders<br>o Acute disseminated encephalomyelitis, including site specific variants (eg, non-infectious encephalitis, encephalomyelitis, myelitis, radiculomyelitis)<br>o Cranial nerve disorders, including paralyses/paresis (eg, Bell's palsy)<br>o Guillain-Barré syndrome, including Miller Fisher syndrome and other variants<br>o Immune-mediated peripheral neuropathies and plexopathies, including chronic inflammatory demyelinating polyneuropathy, multifocal motor neuropathy and polyneuropathies associated with monoclonal gammopathy<br>o Multiple sclerosis<br>o Neuromyelitis optica spectrum disorder<br>o Narcolepsy<br>o Optic neuritis<br>o Transverse myelitis<br>o Myasthenia gravis, including Eaton-Lambert syndrome<br><br>• Skin disorders<br>o Alopecia areata<br>o Autoimmune bullous skin diseases, including pemphigus, pemphigoid and dermatitis herpetiformis<br>o Cutaneous lupus erythematosus<br>o Erythema nodosum<br>o Morphoea<br>o Lichen planus<br>o Psoriasis<br>o Rosacea<br>o Sweet's syndrome<br>o Vitiligo<br>• Vasculitides<br>o Large vessels vasculitis including: giant cell arteritis such as Takayasu's arteritis and temporal arteritis<br>o Medium sized and/or small vessels vasculitis including: polyarteritis nodosa, Kawasaki's disease, microscopic polyangiitis, Wegener's granulomatosis, Churg– Strauss syndrome (allergic granulomatous angiitis), Buerger's disease, thromboangiitis obliterans, necrotizing vasculitis and anti-neutrophil cytoplasmic antibody (ANCA) positive vasculitis (type unspecified), Henoch-Schonlein purpura, Behcet's syndrome, leukocytoclastic vasculitis

AESI	Medical Concept
	• Other<br>○ Antiphospholipid syndrome<br>○ Autoimmune hemolytic anemia<br>○ Autoimmune glomerulonephritis (including IgA nephropathy, glomerulonephritis rapidly progressive, membranous glomerulonephritis, membranoproliferative glomerulonephritis, and mesangioproliferative glomerulonephritis)<br>○ Autoimmune myocarditis/cardiomyopathy<br>○ Autoimmune thrombocytopenia<br>○ Goodpasture syndrome<br>○ Idiopathic pulmonary fibrosis<br>○ Pernicious anemia<br>○ Raynaud's phenomenon<br>○ Sarcoidosis<br>○ Sjögren's syndrome<br>○ Stevens-Johnson syndrome<br>○ Uveitis

ADEM = acute disseminated encephalomyelitis; AESI = adverse event of special interest; GBS = Guillain-Barré syndrome; VAERD = vaccine-associated enhanced respiratory disease.

This list may be updated along with the studies.

AESIs following the first vaccination to 364 days after the last vaccination will be summarized. The number and percentage of participants with AESIs will be summarized by treatment group, and by treatment, AESI term, and PT. Should a participant experience multiple events within a PT, the participant will be counted only once for that PT according to the first onset date.

A listing of all AESIs will be provided at the primary analysis.

10.1.7 Death

Listings of all death due to causes other than COVID-19 will be provided at the time of the primary analysis.

10.2 Clinical Laboratory Results

Clinical laboratory results on scheduled visits for COV001, COV002 and COV005 (group 1 and 3 only) will be pooled to examine the most extreme value encoded in the 30 days post dose 1 and 30 days post dose 2 by treatment group and severity grade. The strategy for severity grading across different studies is provided in Appendix C.

The clinical laboratory tests include:

- Haematology; Full Blood Count
- Biochemistry; Sodium, Potassium, Urea, Creatinine, Albumin, Liver Function Tests (ALT, ALP, Bilirubin)

A listing for clinical laboratory results and a listing for laboratory results with toxicity grading scale ≥ 3 will be provided at the primary analysis.

10.3 Subgroup Analysis

Overview of AEs including factors below will be summarized by subgroup.

- Incidence of all AEs
- Incidence of solicited AEs
- AEs by severity
- Incidence of unsolicited AEs
- Incidence of SAEs
- Incidence of AESI
- Deaths other than due to COVID-19

11. IMMUNOGENICITY ANALYSIS

Immunogenicity analysis will be based on participants with data available. Summary by actual treatment for overall and by serostatus group (Seronegative, Seropositive) for analysis sets planned in Appendix A.

The immunogenicity endpoints are:

- SARS-CoV-2 S, RBD antibody quantification
- Virus NAb assays against SARS-CoV-2
- Antibody seroconversion rate (≥ 4-fold increase from baseline) against SARS-CoV-2 S protein, RBD and NAb

A multiplexed immunoassay that assesses SARS-CoV-2 S, RBD, and N antigens, will be utilised to determine the antibody responses to AZD1222 vaccination. GMTs and GMFRs of these antigens may be provided at baseline and 28 days after each dose in participants for whom data are available.

Data on neutralising antibodies will be provided in the form of a pseudoneutralisation assay, with GMTs and GMFRs reported at the same timepoints as above.

The proportion of participants who have a post-treatment seroresponse (≥ 4-fold rise in titers from the day of dosing baseline value to 28 days post each dose) to AZD1222 as measured by SARS-CoV-2 binding or neutralising antibodies will also be provided, as data are available. For neutralization assays the proportion with neutralizing titres (> LLOQ) will be generated for the primary manuscript.

As bandwidth of laboratories allows, live virus neutralisation and ELISpot data will complement the data provided. These data will be stratified by age (category 1: 18-55, 56-69, and ≥ 70 year-old participants for primary manuscript and category 2: 18-64 and ≥ 65 year-old participants), baseline serostatus and provided following validation of the assays and testing.

Descriptive statistics for GMTs and GMFRs will include number of participants, geometric mean, 95% CI, minimum and maximum. Medians, 25th quartiles and 75% quartiles may also be presented.

The GMT will be calculated as the antilogarithm of Σ (log 2 transformed titer/n), ie, as the antilogarithm transformation of the mean of the log-transformed titer, where n is the number of participants with titer information. The 95% CI will be calculated as the anti-logarithm transformation of the upper and lower limits for a two-sided CI for the mean of the log-transformed titers.

The fold rise is calculated as the ratio of the post-vaccination titer level to the pre-vaccination titer level, ie, the baseline level. GMFR will be calculated as anti-logarithm of Σ (log 2 transformed (post-vaccination titer/ pre-vaccination titer)/n). The 95% CIs for GMFR will be calculated similarly to those for GMT.

Results reported as lower than the LLOQ for SARS-CoV-2 S and RBD responses and virus neutralizing antibody responses will have a value equal to half of the LLOQ imputed in the calculation.

Immunogenicity data will be presented in a listing at the time of the primary analysis.

12. REFERENCES

FDA. (Food and Drug Administration). Guidance for Industry. Toxicity grading scale for healthy adult and adolescent volunteers enrolled in preventive vaccine clinical trials. . https://www.fda.gov/media/73679/download. Published 2007. Accessed 20 June 2020.

Folegatti PM, Ewer KJ, Aley PK, Angus B, Becker S, Belij-Rammerstorfer S et al. Safety and immunogenicity of the ChAdOx1 nCoV-19 vaccine against SARS-CoV-2: a preliminary report of a phase 1/2, single-blind, randomised controlled trial. Lancet. 2020;S0140-6736(20):31604-4.

Lambert P-H, Ambrosino DM, Andersen SR, Baric RS, Black SB, Chen RT et al. Consensus summary report for CEPI/BC March 12–13, 2020 meeting: Assessment of risk of disease enhancement with COVID-19 vaccines. Vaccine. 2020;38(31):4783-91.

NIH. (National Institutes of Health) National Institute of Allergy and Infectious Diseases, Division of AIDS. Division of AIDS (DAIDS) Table for Grading the Severity of Adult and Pediatric Adverse Events, Corrected Version 2.1 [July 2017]. https://rsc.niaid.nih.gov/sites/default/files/daidsgradingcorrectedv21.pdf. Published 2017. Accessed 13 November 2020.

Rajão DS, Chen H, Perez DR, Sandbulte MR, Gauger PC, Loving CL et al. Vaccine-associated enhanced respiratory disease is influenced by haemagglutinin and neuraminidase in whole inactivated influenza virus vaccines. The Journal of general virology. 2016;97(7):1489-99.

SPEAC. (Safety Platform for Emergency Vaccines) D2.3 Priority list of adverse events of special interest: COVID-19. Work Package: WP2 Standards and Tools. v1.1. 05 March 2020. https://media.tghn.org/articles/COVID-19_AESIs_SPEAC_V1.1_5Mar2020.pdf. Published 2020. Accessed 14 June 2020.

van Doremalen N, Lambe T, Spencer A, Belij-Rammerstorfer S, Purushotham JN, Port JR et al. ChAdOx1 nCoV-19 vaccination prevents SARS-CoV-2 pneumonia in rhesus macaques. bioRxiv. 2020;2020.05.13.093195.

WHO, Marshall JC, Murthy S, Diaz J, Adhikari NK, Angus DC et al. Working Group on the Clinical Characterisation and Management of COVID-19 Infection. A minimal common outcome measure set for COVID-19 clinical research. Lancet Infec Dis. 2020;20(8):e192-7.

Zou G. A modified poisson regression approach to prospective studies with binary data. Am J Epidemiol. 2004;159(7):702-6.

13. APPENDICES

Appendix A Overview of Analyses

Table A1 Overview of Evaluation of Efficacy

**Dose and regimen**[a]	**Population**	**N**[b]	**Time period of endpoints**	**Case definition**	**Other case definition**
SDSD + LDSD	18 years and above, Seronegative	~19000[c]	From 15 days post dose 2	Incidence of First SARS-CoV-2 virologically-confirmed[d] COVID-19	Severe COVID-19<br>Hospitalised COVID-19<br>ICU COVID-19<br>Death COVID-19<br>Infection SARS-CoV-2[e]
SDSD	18 years and above, Seronegative	~15600	From 15 days post dose 2	Incidence of First SARS-CoV-2 virologically-confirmed[d] COVID-19	Severe COVID-19<br>Hospitalised COVID-19<br>ICU COVID-19<br>Death COVID-19<br>Infection SARS-CoV-2[e]
First dose is SD	18 years and above, Seronegative	~15800	From 22 days post dose 1	Incidence of First SARS-CoV-2 virologically-confirmed[d] COVID-19	Severe COVID-19<br>Hospitalised COVID-19<br>ICU COVID-19<br>Death COVID-19<br>Infection SARS-CoV-2[e]
Any dose[e]	18 years and above	~19200	From dose 1	Incidence of First SARS-CoV-2 virologically-confirmed[d] COVID-19	Severe COVID-19<br>Hospitalised COVID-19<br>ICU COVID-19<br>Death COVID-19

a by treatment received
b Seronegative is not considered. The dose information is based on the spreadsheet from Oxford in Aug2020. Number is floored to 100.
c Sensitivity analysis of ITT by randomization to AZD1222 or control
d In a subset (COV002)
e Virologically-confirmed from RT-PCR or other nucleic acid amplification test.
f Required for safety evaluation

COVID-19 = coronavirus disease 2019; ICU = intensive care unit; LD = low dose; SARS-CoV-2 = severe acute respiratory syndrome-coronavirus 2; SD = standard dose.

Table A2 Overview of Evaluation of Safety

Dose and regimen[a]	Population	N[b]	Endpoints
First dose is SD	18+	~16000	Reactogenicity<br>Unsolicited AEs<br>SAEs<br>AESIs
Any dose	18+	~19800	Reactogenicity<br>Unsolicited AEs<br>SAEs<br>AESIs

a by treatment received
b Seronegative is not considered. The dose information is based on the spreadsheet from Oxford in Aug2020. Number is floored to 100.
AE = adverse event; AESI = adverse event of special interest; SAE = serious adverse event; SD = standard dose.

Table A3 Overview of Evaluation of Immunogenicity

Dose and regimen[a]	Population	N[b]	Endpoints	Note
SDSD + LDSD	18+	~15600	Spike<br>RBD<br>NAb	By serostatus
SDSD	18+	~19200	Spike<br>RBD<br>NAb	By serostatus

a by treatment received
b Seronegative and non-missing post baseline value is not considered. The dose information is based on the spreadsheet from Oxford in Aug2020. Number is floored to 100.
LD = low dose; SD = standard dose.

Appendix B Pooling and Severity Grading of Solicited Adverse Events: Studies COV001, COV002, COV003, and COV005

B 1 Introduction

In AZD1222 Studies COV001, COV002, COV003, and COV005, diary cards collect predefined local and systemic AEs that are commonly associated with vaccine administration, including the severity of these events. Although there are many similarities in the collection of solicited AEs in these studies, there are also differences. This document presents the similarities and differences in solicited event collection across these studies and a strategy for pooling events across studies.

B 2 Frequency of Solicited Adverse Event Entries

Table B1 presents the schedule of solicited AEs reporting by participants in the patient diaries and the protocol text describing the relevant outcome measures and timepoints, when available in Studies COV001, COV002, COV003, and COV005.

Table B1 Schedule and Protocol Description of Solicited Adverse Event Reporting in Patient Diaries

Study	1st vaccination									2nd vaccination							
COV001	Day 0	Day 1	Day 2	Day 3	Day 4	Day 5	Day 6	Day 7		Day 0	Day 1	Day 2	Day 3	Day 4	Day 5	Day 6	Day 7
- CSP text	Outcome measure: Occurrence of solicited local/systemic reactogenicity signs and symptoms for 7 days following vaccination: Day 0-7 Self-reported symptoms recorded using electronic diaries.																
COV002	Day 0	Day 1	Day 2	Day 3	Day 4	Day 5	Day 6	Day 7		Day 0	Day 1	Day 2	Day 3	Day 4	Day 5	Day 6	Day 7
- CSP text	Outcome measure: Occurrence of solicited systemic reactogenicity signs and symptoms for 7 days following vaccination/booster vaccination: Day 0-7 Self-reported symptoms recorded using electronic diaries																
COV003	Day 0	Day 1	Day 2	Day 3	Day 4	Day 5	Day 6	Day 7		Day 0	Day 1	Day 2	Day 3	Day 4	Day 5	Day 6	Day 7
- CSP text	Endpoint measure: Occurrence of signs and symptoms of local and systemic reactogenicity requested during 7 days after vaccination																
COV005	Day 0	Day 1	Day 2	Day 3	Day 4	Day 5	Day 6	-		Day 0	Day 1	Day 2	Day 3	Day 4	Day 5	Day 6	-
- CSP text	Endpoint measure: occurrence of solicited local/systemic reactogenicity signs and symptoms for 7 days following vaccination;																

CSP = clinical study protocol.

B 3 Solicited Adverse Events

Solicited AEs as defined in the CSP and in the patient diaries are presented in Table B2 below. Studies COV001 and COV002 have identical terms in the CSPs and patients diaries. Study COV003 terms are synonymous with the terms in Studies COV001 and COV002. Study COV005 has identical or synonymous terms for 12 of the solicited AEs, but some protocol-specified events are not collected in the patient diary (ie, pain, warmth, malaise, nausea, vomiting) and an additional local event, bruising, is collected in the patient diary.

**Table B2**	**CSP-specified Solicited Adverse Events and Patient Diary Terms for these Events by Study**					
**Adverse Event**	**CSP Terms**			**Patient Diary Terms**		
**Study**	**COV001, COV002**	**COV003**	**COV005**	**COV001, COV002**	**COV003**	**COV005**
Pain	Pain/<br>Pain at injection site	Pain/<br>Injection site pain	Pain/<br>Pain at injection site	Pain	Pain	-
Tenderness	Tenderness	Sensitivity	Tenderness	Tenderness	Sensitivity	Tenderness
Redness	Erythema at injection site	Redness/<br>Injection site erythema	Redness	Redness	Redness	Redness
Warmth	Warmth	Heat	Warmth	Warmth	Local heat	-
Itch	Itch	Itching	Itch	Itching	Itch	Itching
Swelling	Swelling	Swelling	Swelling	Swelling	Swelling	Swelling
Induration	Induration	Local hardening	Induration	Hardness	Local hardening	Hardness
Fever	Fever	Fever	Fever	Temperature	Temperature	Temperature
Feverishness	Feverishness	Feeling feverish	Feverishness	Feverishness	Feeling feverish	Feeling feverishness
Chills	Chills	Chills	Chills	Chills	Chills	Rigors
Joint pain	Joint pains	Joint pain	Joint pains	Joint pain	Joint pain	Joint pain
Fatigue	Fatigue	Fatigue	Fatigue	More tired than usual	Fatigue	Weakness/<br>Tiredness
Muscle pain	Muscle pains	Muscle ache	Muscle pain	Aching muscles	Muscle pain	Muscle pain
Headache	Headache	Headache	Headache	Headache	Headache	Headache
Malaise	Malaise	Malaise	Malaise	Generally unwell	Malaise	-
Nausea	Nausea	Nausea	Nausea	Nausea	Nausea	-

**Table B2**	**CSP-specified Solicited Adverse Events and Patient Diary Terms for these Events by Study**					
**Adverse Event**	**CSP Terms**			**Patient Diary Terms**		
**Study**	**COV001, COV002**	**COV003**	**COV005**	**COV001, COV002**	**COV003**	**COV005**
Vomiting	Vomiting	Vomiting	-	Vomiting	Vomiting	-
Bruising	-	-	Bruising	-	-	Bruising

CSP = clinical study protocol.

B 4 Pooling of Solicited Adverse Events with Objective Measurements

Four solicited AEs will be reported with objective measurements: fever (measured by body temperature in degrees Celsius), redness, swelling, and induration (reported in diameter in millimeters).

B 4.1 Fever

All 4 studies collected body temperature in degrees Celsius to the nearest tenth of a degree. In the individual CSPs, Studies COV001, COV002, and COV003 specify the use of the US FDA DMID scale for vaccine studies while Study COV005 specifies the use of the US NIH DAIDS grading scale. The scales are presented in Table B3 below. Note that the life-threatening criteria are identical, but the ranges of the other 3 severity grades overlap by 0.1- 0.3 °C.

Table B3 Fever Severity Grades for the DMID and DAIDS Grading Scales

Severity grade	DMID	DAIDS
Grade 1	38.0 - 38.4°C	38.0 to < 38.6°C
Grade 2	38.5 - 38.9°C	≥38.6 to < 39.3°C
Grade 3	39.0 - 40°C	≥39.3 to < 40.0°C
Grade 4	> 40°C	> 40°C

DAID = Division of AIDS; DMID = Division of Microbiology and Infectious Diseases

As the data collected are objective numerical measurements, the data can be easily pooled. The severity grades of fever from all 4 studies will be assessed based upon the DMID scale specified in Studies COV001, COV002, and COV003 prior to pooling.

B 4.2 Redness, Swelling, and Induration

Redness and swelling will be reported in millimeters in all 4 studies, and all 4 CSPs contain the same severity grading (Table B4). (Note: the source data are recorded in millimeters but the grading criteria use centimeter; unit conversion will be required).

Induration will also be reported in millimeters in Studies COV001, COV002, and COV003 with the same protocol-defined severity grades, in centimeters. However, in Study COV005, induration is reported in the patient diary at 3 severity grade levels (mild, moderate, severe) that are not compatible with the millimeter measurements used in the other 3 studies.

Based upon the above, redness and swelling can be pooled across all 4 studies, and induration can be pooled across Studies COV001, COV002, and COV003.

Table B4 CSP-specified Severity Grading and Patient Diary Measurements for Redness, Swelling, and Induration

Event	CSP-specified grading severity	Patient diary measurement	
Study	COV001, COV002, COV003, COV005	COV001, COV002, COV003	COV005
Redness	Grade 1: 2.5-5 cm	Millimeter	Millimeter
	Grade 2: 5.1-10 cm		
	Grade 3: > 10 cm		
	Grade 4: Necrosis or exfoliative dermatitis		
Swelling	Grade 1: 2.5-5 cm and does not interfere with activity	Millimeter	Millimeter
	Grade 2: 5.1 - 10 cm or interferes with activity		
	Grade 3: > 10 cm or prevents daily activity		
	Grade 4: Necrosis		
Induration	Grade 1: 2.5-5 cm and does not interfere with activity	Millimeter	Grade 1: small hard lumps/swelling felt, smaller than 25 mm
	Grade 2: 5.1 - 10 cm or interferes with activity		Grade 2: hard lump/swelling felt, larger than 25 mm
	Grade 3: > 10 cm or prevents daily activity		Grade 3: large lump/swelling felt, wider than half the arm width
	Grade 4: Necrosis		-

CSP = clinical study protocol.

B 4.3 Bruising

Only Study COV005 collected bruising as a solicited AE. Severity grading within the diary consisted of Grades 1 (< 10 mm), 2 (10-25 mm), and 3 (> 25 mm). For the sake of completeness, these results will be presented within the pooled data output.

B 5 Pooling of Solicited Adverse Events with Subjective Measurements

This section presents events that will be reported by participants using subjective severity grades.

B 5.1 Pain and Warmth at the Injection Site, Malaise, Nausea, and Vomiting

Pain and warmth at the injection site and malaise, nausea, and vomiting will be reported in Studies COV001, COV002, and COV003. (Study COV005 did not include vomiting in the protocol and did not collect pain and warmth at the injection site and malaise and nausea despite being specified in the protocol.) Common severity grades are used in Studies COV001 and COV002 (Table B5). The severity grades in Study COV003 are somewhat different but similar enough to justify pooling of these 4 solicited AE across all 3 studies.

Table B5 Patient Diary Severity Grades for Pain and Warmth at Injection Site, Malaise, Nausea, and Vomiting Events in Studies COV001, COV002, and COV003

Severity	COV001, COV002	COV003
Grade 1	Mild: easily tolerated with no limitation on normal activity	Mild: transient or mild discomfort (< 48 hours); no interference with routine activities; no medical intervention/therapy required
Grade 2	Moderate: some limitation of daily activity	Moderate: mild to moderate limitation in routine activities – some assistance may be needed; no or minimal medical intervention/therapy required
Grade 3	Severe: unable to perform normal daily activity	Severe: marked limitation in routine activities, some assistance usually required; medical intervention/therapy required
Grade 4	Emergency department or hospital admission required	Potentially life-threatening: requires assessment in accident & emergency department.or hospitalization

B 5.2 Feverishness and Chills

Feverishness and chills will be reported with common severity grades in Studies COV001 and COV002, slightly different severity grades in Study COV003, and a binary “yes” or “no” without severity grading in Study COV005 (Table B6). The severity grades used in Studies COV001, COV002, and COV003 are similar enough to justify pooling of these 2 solicited AEs across the 3 studies. The lack of severity grading of these events in Study COV005 precludes them being pooled with the other studies.

Table B6 Patient Diary Severity Grades for Feverishness and Chills in Studies COV001, COV002, and COV003

Severity	COV001, COV002	COV003	COV005
Grade 1	Mild: easily tolerated with no limitation on normal activity	Mild: transient or mild discomfort (< 48 hours); no interference with routine activities; no medical intervention/therapy required	Yes/No (without grading)
Grade 2	Moderate: some limitation of daily activity	Moderate: mild to moderate limitation in routine activities – some assistance may be needed; no or minimal medical intervention/therapy required	
Grade 3	Severe: unable to perform normal daily activity	Severe: marked limitation in routine activities, some assistance usually required; medical intervention/therapy required	
Grade 4	Emergency department or hospital admission required	Potentially life-threatening: requires assessment in accident & emergency department or hospitalization	

B 5.3 Tenderness, Itching, Joint pain, Muscle pain, Fatigue, and Headache

These solicited AEs have identical 4-level severity grading scales in Studies COV001 and COV002 and a slightly different 4-level grading scale in Study COV003 (Table B7). Study COV005 has event-specific criteria and differs further from the other studies in that a 3-point mild, moderate, and severe scale will be used for these events. The severity grades used in Studies COV001, COV002, and COV003, as discussed above, are similar enough to justify pooling of these 6 solicited AEs across all 3 studies. In addition, the 3-level event-specific severity grades used in Study COV005 do define mild, moderate, and severe events and are also judged to be similar enough to justify pooling across studies.

Table B7 Patient Diary Severity Grading for Tenderness, Itching, Joint Pain, Muscle Pain, Fatigue, and Headache by Study

**COV001, COV002**	**COV003**	**COV005**					
**All events**	**All events**	**Tenderness**	**Itching**	**Joint pain**	**Muscle pain**	**Fatigue**	**Headache**
Mild: easily tolerated with no limitation on normal activity	Mild: transient or mild discomfort (< 48 hours); no interference with routine activities; no medical intervention/therapy required	Mild: minor tenderness when injection site is touched	Mild: minor itching	Mild: mild aching	Mild aching	Minor weakness/ tiredness	Minor headache not requiring medication
Moderate: some limitation of daily activity	Moderate: mild to moderate limitation in routine activities – some assistance may be needed; no or minimal medical intervention/therapy required	Moderate: very tender when injection site is touched	Moderate: marked itching (like new mosquito bite)	Moderate: severe aching, but able to do most activities	Severe aching, but able to do most activities	Very weak/tired	Bad headache, but able to do most activities (with medication)
Severe: unable to perform normal daily activity	Severe: marked limitation in routine activities, some assistance usually required; medical intervention/therapy required	Severe: severe pain in the injected limb, increased when it is moved or movement of the limb is reduced	Severe: severe itching requiring soothing cream	Severe: very severe aching, requiring medication, limiting activities	Very severe aching, requiring medication, limiting activities	Unable to do normal activities during the day	Severe headache, requiring medication and unable to do normal activities during the day
Emergency department or hospital admission required	Potentially Life-threatening: requires assessment in accident & emergency department or hospitalization	-	-	-	-	-	-

B 6 Overall Solicited Adverse Event Pooling Strategy

Table B8 Contribution of Individual Studies to the Overall Pool for Solicited Adverse Events

	Data to be included in the pooling by study				Comments
	COV001	COV002	COV003	COV005	
Solicited local adverse events					
Pain	X	X	X	NA	Grade based upon Studies COV001/COV002 grading criteria. No data from Study COV005.
Tenderness	X	X	X	X	Grade based upon Studies COV001/COV002 grading criteria. Study COV005 scale lacks Grade 4
Redness	X	X	X	X	Grade based upon common objective grading criteria (diameter)
Warmth	X	X	X	NA	Grade based upon Studies COV001/COV002 grading criteria. No data from Study COV005.
Itch	X	X	X	X	Grade based upon Studies COV001/COV002 grading criteria. Study COV005 scale lacks grade 4
Swelling	X	X	X	X	Grade based upon common objective grading criteria (diameter)
Induration	X	X	X	-	Grade based upon the common objective grading criteria. Study COV005 grading scale not compatible.
Bruising	NA	NA	NA	X	Included despite recorded in only one study so that all available data are included.
Solicited systemic adverse events					
Fever	X	X	X	X	Grade based upon Studies COV001/COV002/COV003 grading criteria.
Feverishness	X	X	X	-	Grade based upon Studies COV001/COV002 grading criteria. Study COV005 has no severity grading
Chills	X	X	X	-	Grade based upon Studies COV001/COV002 grading criteria. Study COV005 has no severity grading.
Joint pain	X	X	X	X	Grade based upon Studies COV001/COV002 grading criteria. Study COV005 scale lacks Grade 4
Muscle pain	X	X	X	X	Grade based upon Studies COV001/COV002 grading criteria. Study COV005 scale lacks

Table B8 Contribution of Individual Studies to the Overall Pool for Solicited Adverse Events

	Data to be included in the pooling by study				Comments
	COV001	COV002	COV003	COV005	
					Grade 4
Fatigue	X	X	X	X	Grade based upon Studies COV001/COV002 grading criteria. Study COV005 scale lacks Grade 4
Headache	X	X	X	X	Grade based upon Studies COV001/COV002 grading criteria. Study COV005 scale lacks Grade 4
Malaise	X	X	X	NA	Grade based upon Studies COV001/COV002 grading criteria. No data from Study COV005.
Nausea	X	X	X	NA	Grade based upon Studies COV001/COV002 grading criteria. No data from Study COV005.
Vomiting	X	X	X	NA	Grade based upon Studies COV001/COV002 grading criteria. No data from Study COV005.

NA = not available.

Appendix C Pooling and Severity Grading of Laboratory Assessed on Scheduled Visits

In Studies COV001, COV002, and COV005, the severity of laboratory toxicity from scheduled assessments will be assessed on scales of Grades 1-4. In Studies COV001 and COV002, these laboratory abnormalities will be assessed according to grading scales that have been adapted from the US FDA Toxicity Grading Scale for Healthy and Adolescent Volunteers Enrolled in Preventive Vaccine Clinical Trials (FDA 2007), using identical grading scales. In Study COV005, laboratory toxicity will be assessed using grading scales adapted from the DAIDS Table for Grading the Severity of Adult and Pediatric Adverse Events, Corrected Version 2.1 (NIH 2017).

In order to pool laboratory abnormalities from these studies, all pooled data will be uniformly graded using the US FDA grading system as the common grading system. (Note: Study COV003 does not contribute data to this pooling as the study does not have scheduled laboratory assessments.) For the purposes of reporting, values which do not meet at least the criteria of Grade 1 will be reported as normal, to account for results falling between the upper limit of normal for individual laboratories and the lower limit for Grade 1 toxicity. For those variables in which US FDA severity gradings are based upon multiples of the upper limit of the normal reference range (ie, alanine transaminase, alkaline phosphate, and bilirubin), study specific reference ranges will be used.

Table C1 presents the severity gradings that will be used for pooled laboratory abnormalities from Studies COV001, COV002, and COV005. Table C2 presents a comparison of the US FDA severity grade criteria with the 3 studies' individual criteria.

Table C1 Laboratory Abnormality Severity Grade Criteria to be Used for Pooled COV1, COV002, and COV005 Data

Variable	Unit	Normal Range	Grade 1	Grade 2	Grade 3	Grade 4	Comments
Haemoglobin Absolute Decreased (male)	g/L	-	125-135	105-124	85-104	<85	FDA values.
Haemoglobin Absolute Decreased (female)	g/L	-	110-120	95-109	80-94	<80	FDA values.
Haemoglobin Decrease from Baseline	g/L	-	1-15	16-20	21-50	>50	FDA values, Consistent with COV001, COV002, except lower limit for Grade 1
White Blood Cells-Elevated	cells × 10^9/L	-	10.8-15.0	>15.0-20.0	>20.0-25.0	>25.0	FDA values. Consistent with COV001, COV002, except lower limit for Grade 1
White Blood Cells-Decreased	cells × 10^9/L	-	2.5-3.5	1.5-<2.5	1.0-<1.5	<1.0	FDA values. COV001, COV002
Platelets-Decreased	cells × 10^9/L	-	125-140	100-124	25-99	<25	FDA values. COV001, COV002
Neutrophils-Decreased	cells × 10^9/L	-	1.50-2.00	1.00-1.49	0.50-0.90	<0.50	FDA values. COV001, COV002
Lymphocytes-Decreased	cells × 10^9/L	-	0.750-1.000	0.500-0.749	0.250-0.499	<0.250	FDA values. COV001, COV002
Eosinophils-Elevated	cells × 10^9/L	-	0.650-1.500	1.501-5.000	>5.000	-	FDA values. COV001, COV002
Sodium-Elevated	mmol/L	-	144-145	146-147	148-150	>150	FDA values
Sodium-Decreased	mmol/L	-	132-134	130-131	125-129	>125	FDA values. COV001, COV002
Potassium-Elevated	mmol/L	-	5.1-5.2	5.3-5.4	5.5-5.6	>5.6	FDA values
Potassium-Decreased	mmol/L	-	3.5-3.6	3.3-3.4	3.1-3.2	<3.1	FDA values.

Table C1 Laboratory Abnormality Severity Grade Criteria to be Used for Pooled COV1, COV002, and COV005 Data

Variable	Unit	Normal Range	Grade 1	Grade 2	Grade 3	Grade 4	Comments
Urea-Elevated (*converted from BUN mg/dL)*	mmol/L	-	8.2-9.4	9.5-11.0	>11.0	-	FDA values. COV001, COV002
Creatinine-Elevated (*converted from mg/dL)*	µmol/L	-	133-154	155-181	182- 221	>221	FDA values
Bilirubin-Elevated with normal ALT/ALP	µmol/L	COV001/ COV002: 0-21 COV005: 5-21	1.1-1.5 × ULN	1.6-2.0 × ULN	2.0-3.0 × ULN	>3.0 × ULN	FDA values. COV001, COV002. Country specific normal ranges.
Bilirubin-Elevated with abnormal ALT/ALP	µmol/L		1.1-1.25 × ULN	1.26-1.5 × ULN	1.51-1.75 × ULN	>1.75 × ULN	FDA values. COV001, COV002. Country specific normal ranges.
Alanine Transaminase-Elevated	U/L	COV001/ COV002: 10-45 COV005: 15-40	1.1-2.5 × ULN	2.6-5.0 × ULN	5.1-10 × ULN	>10 × ULN	FDA values. COV001, COV002. Country specific normal ranges.
Alkaline Phosphate-Elevated	U/L	COV001, COV002: 30-130 COV005: 53-128	1.1-2.0 × ULN	2.1-3.0 × ULN	3.1-10 × ULN	>10 × ULN	FDA values. COV001, COV002. Country specific normal ranges.
Albumin-Decreased	g/L	-	28-31	25-27	<25	-	FDA values. COV001, COV002.

ALP = alkaline phosphate; ALT = alanine aminotransferase; BUN = blood urea nitrogen; FDA = (US) Food and Drug Administration; N.A. = not available; ULN = upper limit of normal.

Table C2 Laboratory Abnormality Severity Grade Criteria to be Used for Pooled COV001, COV002, and COV005 Data

Variable	Unit	Standard	Normal Range	Grade 1	Grade 2	Grade 3	Grade 4
Haemoglobin Absolute Decreased (male)	g/L	FDA	-	125-135	105-124	85-104	<85
		COV001 COV002	130-170	115-125	100-114	85-99	<85
		COV005 (DAIDS)	-	100-109	90-<100	70-<90	<70
Haemoglobin Absolute Decreased (female)	g/L	FDA	-	110-120	95-109	80-94	<80
		COV001 COV002	120-150	105-113	90-104	80-89	<80
		COV005 (DAIDS)	-	95-104	85-<95	65-<85	<65
Haemoglobin Decrease from Baseline	g/L	FDA	-	1-15	16-20	21-50	>50
		COV001 COV002	-	10-15	16-20	21-50	>50
		COV005 (DAIDS)	-	N.A.	N.A.	N.A.	N.A.
White Blood Cells-Elevated	cells × 10^9/L	FDA	-	10.8-15	>15-20	>20-25	>25
		COV001 COV002	4.0-11	11.5-15	>15-20	>20-25	>25
		Cov005 (DAIDS)	-	N.A.	N.A.	N.A.	N.A.
White Blood Cells-Decreased	cells × 10^9/L	FDA	-	2.5-3.5	1.5-2.49	1.0-1.49	<1.0
		COV001 COV002	4.0-11	2.5-3.5	1.5-2.49	1.0-1.49	<1.0
		Cov005 (DAIDS)	-	2.00-2.49	1.50-1.99	1.00-1.49	<1.0
Platelets-Decreased	cells × 10^9/L	FDA	-	125-140	100-124	25-99	<25
		COV001 COV002	150-400	125-140	100-124	25-99	<25
		COV005 (DAIDS)	-	100-< 125	50-<100	25-<50	< 25
Neutrophils-Decreased	cells × 10^9/L	FDA	-	1.5-2.00	1.0-1.49	0.5- 0.99	<0.50
		COV001 COV002	2.0-7.0	1.5-1.99	1.0-1.49	0.5-0.99	<0.50
		COV005 (DAIDS)	-	0.80-1.00	0.60-0.79	0.40-0.59	<0.40
Lymphocytes-Decreased	cells ×	FDA	-	0.750-1.000	0.500-0.749	0.250-0.499	<0.250

Table C2 Laboratory Abnormality Severity Grade Criteria to be Used for Pooled COV001, COV002, and COV005 Data

Variable	Unit	Standard	Normal Range	Grade 1	Grade 2	Grade 3	Grade 4
	10^9/L	COV001 COV002	1.0-4.0	0.75-0.99	0.5-0.74	0.25-0.49	<0.25
		COV005	-	0.60-0.65	0.500- 0.599	0.350-0.499	<0.350
Eosinophils-Elevated	cells × 10^9/L	FDA	-	0.650-1.500	1.501-5.000	>5.000	Hypereosinophilic
		COV001 COV002	0.02-0.5	0.65-1.5	1.51-5.00	>5.00	Hypereosinophilic
		COV005	-	N.A.	N.A.	N.A.	N.A.
Sodium-Elevated	mmol/L	FDA	-	144-145	146-147	148-150	>150
		COV001 COV002	134-145	146-147	148-149	150-155	>155
		COV005 (DAIDS)	-	146 to < 150	150 to < 154	154 to < 160	≥ 160
Sodium-Decreased	mmol/L	FDA	-	132-134	130-131	125-129	<125
		COV001 COV002	135-145	132-134	130-131	125-129	<125
		COV005 (DAIDS)	-	130-< 135	125-< 130	121-< 125	≤120
Potassium-Elevated	mmol/L	FDA	-	5.1-5.2	5.3-5.4	5.5-5.6	>5.6
		COV001 COV002	3.5-5	5.1-5.2	5.3-5.4	5.5-6.5	>6.5
		COV005 (DAIDS)	-	5.6-< 6.0	6.0-< 6.5	6.5-< 7.0	≥ 7.0
Potassium-Decreased	mmol/L	FDA	-	3.5-3.6	3.3-3.4	3.1-3.2	<3.1
		COV001 COV002	-	3.2-3.3	3.1	2.5-3.0	<2.5
		COV005 (DAIDS)	-	3.0-< 3.4	2.5-< 3.0	2.0-< 2.5	< 2.0
Urea-Elevated<br><br>(*converted from BUN mg/dL)*	mmol/L	FDA	-	8.2-9.3	9.4-11.0	>11.0	Requires dialysis
		COV001 COV002	2.5-7.4	8.2-9.3	9.4-11.0	>11.0	Requires dialysis
		COV005 (DAIDS)	-	N.A.	N.A.	N.A.	N.A.
Creatinine-Elevated (*converted from mg/dL)*	mg/dL (µmol/L)	FDA	-	1.5-1.7 (133-154)	1.8-2.0 (155-181)	2.1-2.5 (182-221)	>2.5 (>221) or requires dialysis

Table C2 Laboratory Abnormality Severity Grade Criteria to be Used for Pooled COV001, COV002, and COV005 Data

Variable	Unit	Standard	Normal Range	Grade 1	Grade 2	Grade 3	Grade 4
		COV001 COV002	49-104	1.1-1.5 × ULN<br>114-156	>1.5-2.0 × ULN<br>157-312	>2.0 × ULN<br>>312	Requires dialysis
		COV005	64-10	1.1-1.3 × ULN	> 1.3-1.8 × ULN	> 1.8-< 3.5 × ULN	≥ 3.5 × ULN
Bilirubin-Elevated	µmol/L	FDA with normal ALT/ALP	-	1.1-1.5 × ULN	1.6-2.0 x ULN	2.0-3.0 × ULN	>3.0 × ULN
		FDA with abnormal ALT/ALP		1.1-1.25 × ULN	1.26-1.5 × ULN	1.51-1.75 × ULN	>1.75 × ULN
		COV001<br>COV002<br>-Normal LFTs	0-21	1.1-1.5 × ULN<br>23-32	>1.5-2 × ULN<br>33-42	>2-3 × ULN<br>43-63	>3 × ULN<br>>64
		COV005<br>-Normal LFTs	5-21				
		COV001<br>COV002<br>-Abnormal LFTs	0-21	1.1-1.25 × ULN<br>23-26	>1.25-1.5 × ULN<br>27-32	>1.5-1.75 × ULN<br>33-37	>1.75 × ULN<br>>37
		COV005<br>-Abnormal LFTs	5-21				
		COV005 (DAIDS)	-	1.1-< 1.6 × ULN	1.6-< 2.6 × ULN	2.6-< 5.0 × ULN	≥ 5.0 × ULN
Alanine Transaminase-Elevated	U/L	FDA		1.1-2.5 ULN	2.6-5 × ULN	5.1-10 × ULN	>10 × ULN
		COV001 COV002	10-45	1.1-2.5 × ULN<br>49-112	>2.5-5 × ULN<br>113-225	>5-10 × ULN<br>226-450	>10 × ULN<br>>450
		COV005 (DAIDS)	15-40	1.25-< 2.5 × ULN	2.5-< 5.0 × ULN	5.0-< 10.0 × ULN	≥10.0 × ULN
Alkaline Phosphate-	µmol/L	FDA		1.1-2.0 × ULN	2.1-3.0 × ULN	3.1-10 × ULN	>10 × ULN

Table C2 Laboratory Abnormality Severity Grade Criteria to be Used for Pooled COV001, COV002, and COV005 Data

Variable	Unit	Standard	Normal Range	Grade 1	Grade 2	Grade 3	Grade 4
		COV001 COV002	30-130	1.1-2 × ULN<br>143-260	>2.-3 × ULN<br>261-390	>3-10 × ULN<br>391-1300	>10 × ULN<br>>1300
		COV005 (DAIDS)	53-128	1.25-< 2.5 × ULN	2.5-< 5.0 × ULN	5.0-< 10.0 × ULN	≥ 10.0 × ULN
Albumin-Decreased	μmol/L	FDA	-	28-31	25-27	<25	N.A.
		COV001 COV002	32-50	28-31	25-27	<25	N.A.
		COV005 (DAIDS)	-	30-<LLN	≥20-<30	<20	N.A.

ALP = alkaline phosphate; ALT = alanine aminotransferase; BUN = blood urea nitrogen; DAIDS = Division of AIDS; FDA = (US) Food and Drug Administration; N.A. = not available; LFT = liver function tests; LLN = lower limit of normal; ULN = upper limit of normal

Trial Title: A phase I/II study to determine efficacy, safety and immunogenicity of the candidate Coronavirus Disease (COVID-19) vaccine ChAdOx1 nCoV-19 in UK healthy adult volunteers

Short title A phase I/II study of a candidate COVID-19 vaccine (COV001)

Study Reference: COV001

Protocol Version: 12.0

Date: 09 Nov 2020

EudraCT number: 2020-001072-15

REC Reference: 20/SC/0145

IRAS Reference: 281259

Chief Investigator: Prof Andrew Pollard

Sponsor: University of Oxford

Funder: UK Research and Innovation

Key Trial Contacts

**Chief Investigator**	Prof Andrew Pollard
**Address**	Centre for Clinical Vaccinology and Tropical Medicine<br>University of Oxford,<br>Churchill Hospital, Old Road, Headington<br>Oxford, OX3 7LE<br>Email: andrew.pollard@paediatrics.ox.ac.uk
**Clinical Trials Unit Investigators**	Primary Care and Vaccines Collaborative CTU<br>Prof Brian Angus<br>Centre for Clinical Vaccinology and Tropical Medicine<br>Churchill Hospital, Old Road, Headington<br>Oxford, OX3 7LE<br>Email: brian.angus@ndm.ox.ac.uk<br><br>Prof Adrian Hill<br>The Jenner Institute, University of Oxford<br>Old Road Campus Research Building (ORCRB)<br>Roosevelt Drive Oxford OX3 7DQ<br>Email: adrian.hill@ndm.ox.ac.uk<br><br>Prof Sarah Gilbert<br>The Jenner Institute, University of Oxford<br>Old Road Campus Research Building (ORCRB)<br>Roosevelt Drive Oxford OX3 7DQ<br>Email: sarah.gilbert@ndm.ox.ac.uk<br><br>Dr Pedro Folegatti<br>Centre for Clinical Vaccinology and Tropical Medicine<br>Churchill Hospital, Old Road, Headington<br>Oxford, OX3 7LE<br>Email: pedro.folegatti@ndm.ox.ac.uk<br><br>Dr Alexander Douglas<br>The Jenner Institute<br>Wellcome Centre for Human Genetics<br>Roosevelt Drive<br>Oxford, OX3 7BN<br>Email: sandy.douglas@ndm.ox.ac.uk<br><br>Dr Maheshi Ramasamy<br>Oxford Vaccine Group<br>Centre for Clinical Vaccinology and Tropical Medicine<br>University of Oxford,<br>Churchill Hospital, Old Road, Headington<br>Oxford, OX3 7LE<br>Email : maheshi.ramasamy@paediatrics.ox.ac.uk

Prof Matthew Snape
Oxford Vaccine Group
Centre for Clinical Vaccinology and Tropical Medicine
University of Oxford,
Churchill Hospital, Old Road, Headington
Oxford, OX3 7LE
Email : matthew.snape@paediatrics.ox.ac.uk

Prof Teresa Lambe
The Jenner Institute, University of Oxford
Old Road Campus Research Building (ORCRB)
Roosevelt Drive Oxford OX3 7DQ
Email: teresa.lambe@ndm.ox.ac.uk

Prof Katie Ewer
The Jenner Institute, University of Oxford
Old Road Campus Research Building (ORCRB)
Roosevelt Drive Oxford OX3 7DQ
Email: katie.ewer@ndm.ox.ac.uk

Dr Angela Minassian
Centre for Clinical Vaccinology and Tropical Medicine
University of Oxford,
Churchill Hospital, Old Road, Headington
Oxford, OX3 7LE
Email : angela.minassian@ndm.ox.ac.uk

Trial Sites

Centre for Clinical Vaccinology & Tropical Medicine
University of Oxford
Churchill Hospital, Old Road, Headington, Oxford, OX3 7LE
PI at Site: Professor Adrian Hill
Email: adrian.hill@ndm.ox.ac.uk

Oxford University Hospital Foundation Trust
Prof Brian Angus
John Radcliffe Hospital
Headley Way, Headington
Oxford
OX3 9DU
Email: brian.angus@ndm.ox.ac.uk

NIHR WTCRF
University Hospital Southampton NHS Foundation Trust Southampton, SO16 6YD
PI at Site: Professor Saul Faust
Email: s.faust@soton.ac.uk

IHR Imperial CRF
NIHR Imperial Clinical Research Facility Imperial College
Hammersmith Hospital, Imperial College NHS Trust

	150 Du Cane Road, London, W12 0HS<br>PI at Site: Dr Katrina M. Pollock<br>Email: k.pollock@imperial.ac.uk
	**St Georges University Hospital NHS Foundation Trust**<br>Blackshaw Road, Tooting, London<br>SW17 0QT<br>PI at site: Professor Paul Heath<br>Email: pheath@sgul.ac.uk
	**St Georges University of London**<br>Paediatric Infectious Diseases Research Group<br>Jenner Wing, Level 2, Room 2.216F, Mail Point J2C<br>London<br>SW17 0RE<br>Email: pheath@sgul.ac.uk
	**University Hospitals Bristol and Weston NHS Foundation Trust**<br>Marlborough Street, Bristol, Avon<br>BS1 3NU<br>PI at site: Dr Rajeka Lazarus<br>Co Investigator: Professor Adam Finn<br>PI Email: rajeka.lazarus@uhbw.nhs.uk<br>Co- investigator email: Adam.Finn@bristol.ac.uk
**Sponsoring Institution**	**University of Oxford**<br>Clinical Trials and Research Governance<br>Joint Research Office,<br>Boundary Brook House<br>Churchill Drive, Headington<br>Oxford OX3 7GB<br>Email: ctrg@admin.ox.ac.uk
**Funder**	**UK Government**
**Monitor**	**Clinical Trials & Research Governance (Oxford Sites)**<br>Joint Research Office,<br>Boundary Brook House<br>Churchill Drive, Headington<br>Oxford OX3 7GB
	**Oxford Vaccine Centre (other sites)**<br>CCVTM<br>Churchill Hospital<br>Headington<br>OXON<br>OX3 7LE<br>Email: covid19@ndm.ox.ac.uk
**DSMB Chair**	Professor Christopher Chiu<br>Imperial College London

Professor Robert Heyderman (Co-chair)
University College London

Statistician

Merryn Voysey
Oxford Vaccine Centre
CCVTM, Churchill Hospital
Headington, OXON
OX3 7LE
Merryn.voysey@paediatrics.ox.ac.uk

Signature:

Confidentiality Statement

This document contains confidential information that must not be disclosed to anyone other than the Sponsor, the Investigator Team, HRA, host organisation, and members of the Research Ethics Committee and other regulatory bodies. This information cannot be used for any purpose other than the evaluation or conduct of the clinical investigation without the prior written consent of Prof Andrew Pollard.

Statement of Compliance
The trial will be conducted in compliance with the protocol, the principles of Good Clinical Practice, Medicines for Human Use (Clinical Trial) Regulations 2004 (as amended) and all other applicable regulatory requirements.

Investigator Agreement and Notification of Conflict of Interest I approve this protocol for use in the above named clinical trial and agree to abide by all provisions set forth therein.

According to the Declaration of Helsinki, 2008, I have read this protocol, and declare no/~~the following (delete as appropriate)~~ conflict of interest

Chief Investigator<br><br>Prof Andrew Pollard	Signature	Date:

Site: **Centre for Clinical Vaccinology and Tropical Medicine, University of Oxford**

I have read this protocol and agree to abide by all provisions set forth therein.

According to the Declaration of Helsinki, 2008, I have read this protocol, and declare the following conflict of interest. AH is a cofounder of and minor shareholder in an Oxford University spin-off company, Vaccitech Ltd, that has some non-exclusive rights to the vector, ChAdOx1, used in the vaccine to be tested, that may be of commercial value”

Principal Investigator<br><br>Adrian Hill	Signature	Date:

Site: **NIHR WTCRF**

I have read this protocol and agree to abide by all provisions set forth therein.

According to the Declaration of Helsinki, 2008, I have read this protocol, and declare no/~~the following (delete as appropriate)~~ conflict of interest

Principal Investigator<br><br>Saul Faust	Signature	Date:

Site: **NIHR Imperial CRF**

I have read this protocol and agree to abide by all provisions set forth therein.

According to the Declaration of Helsinki, 2008, I have read this protocol, and declare no/~~the following (delete as appropriate)~~ conflict of interest

Principal Investigator<br><br>Katrina M. Pollock	Signature	Date:

Site: **Oxford University Hospital Foundation Trust**

I have read this protocol and agree to abide by all provisions set forth therein.

According to the Declaration of Helsinki, 2008, I have read this protocol, and declare no/~~the following (delete as appropriate)~~ conflict of interest

Principal Investigator<br><br>Brian Angus	Signature	Date:

Site: **St Georges University Hospital NHS Foundation Trust**

I have read this protocol and agree to abide by all provisions set forth therein.

According to the Declaration of Helsinki, 2008, I have read this protocol, and declare no/~~the following (delete as appropriate)~~ conflict of interest

Principal Investigator<br><br>Paul Heath	Signature	Date:

Site: **St Georges University of London**

I have read this protocol and agree to abide by all provisions set forth therein.

According to the Declaration of Helsinki, 2008, I have read this protocol, and declare no/~~the following (delete as appropriate)~~ conflict of interest

Principal Investigator<br><br>Paul Heath	Signature	Date:

Site: **University Hospitals Bristol and Weston NHS Foundation Trust**

I have read this protocol and agree to abide by all provisions set forth therein.

According to the Declaration of Helsinki, 2008, I have read this protocol, and declare no/~~the following (delete as appropriate)~~ conflict of interest

Principal Investigator<br><br>Rajeka Lazarus	Signature	Date:

Table of Contents

1 SYNOPSIS ... 14
2 ABBREVIATIONS ... 19
3 BACKGROUND AND RATIONALE ... 21
3.1 Background ... 21
3.2 Pre-Clinical Studies ... 22
3.2.1 Immunogenicity (Jenner Institute, unpublished) ... 22
3.2.2 Efficacy ... 23
3.3 Disease Enhancement ... 23
3.4 Previous clinical experience ... 24
3.5 Rationale ... 29
4 OBJECTIVES AND ENDPOINTS ... 31
5 TRIAL DESIGN ... 33
5.1 Study groups ... 35
5.2 Trial volunteers ... 36
5.3 Definition of End of Trial ... 36
5.4 Duration of study ... 36
5.5 Potential Risks for volunteers ... 36
5.6 Known Potential Benefits ... 38
6 RECRUITMENT AND WITHDRAWAL OF TRIAL VOLUNTEERS ... 39
6.1 Identification of Trial Volunteers ... 39
6.2 Informed consent ... 40
6.3 Inclusion and exclusion criteria ... 41
6.3.1 Inclusion Criteria ... 41
6.3.2 Exclusion Criteria ... 41
6.3.3 Re-vaccination exclusion criteria ... 43
6.3.4 Effective contraception for female volunteers ... 44
6.3.5 Prevention of 'Over Volunteering' ... 44

6.3.6 Withdrawal of Volunteers 45
6.4 Pregnancy 45
7 TRIAL PROCEDURES 47
7.1 Schedule of Attendance 47
7.2 Observations 47
7.3 Blood tests, Nose/Throat Swab and urinalysis 47
7.4 Study visits 49
7.4.1 Screening visit 49
7.4.2 Day 0: Enrolment and vaccination visit 50
7.4.2.1 Vaccination 50
7.4.2.2 Sequence of Enrolment and Vaccination of Volunteers 50
7.4.3 Subsequent visits: 53
7.4.4 Participants under quarantine 53
7.4.5 Symptomatic volunteers 69
7.4.6 Household Weekly Questionnaire (optional) 69
7.4.7 Stool samples (optional) 70
7.4.8 Medical notes review 70
7.4.9 Randomisation, blinding and code-breaking 70
8 INVESTIGATIONAL PRODUCT AND TRIAL INTERVENTIONS 72
8.1 Manufacturing and presentation 72
8.1.1 Description of ChAdOx1 nCoV-19 72
8.2 Supply 72
8.3 Storage 72
8.4 Administration 72
8.5 Rationale for selected dose 73
8.6 Minimising environmental contamination with genetically modified organisms (GMO) 76
8.7 Control Vaccine 76
8.8 Compliance with Trial Treatment 77

8.9 Accountability of the Trial Treatment 77

8.10 Paracetamol (non-IMP) 77

8.11 Concomitant Medication 77

8.12 Provision of Treatment for Controls 77

9 ASSESSMENT OF SAFETY 78

9.1 Definitions 78

9.1.1 Adverse Event (AE) 78

9.1.2 Adverse Reaction (AR) 78

9.1.3 Serious Adverse Event (SAE) 78

9.1.4 Serious Adverse Reaction (SAR) 79

9.1.5 Suspected Unexpected Serious Adverse Reaction (SUSAR) 79

9.2 Expectedness 79

9.3 Foreseeable Adverse Reactions: 79

9.4 Adverse Events of Special Interest (AESI) 79

9.5 Causality 80

9.6 Reporting Procedures for All Adverse Events 81

9.7 Assessment of severity 82

9.8 Reporting Procedures for Serious AEs 84

9.9 Reporting Procedures for SUSARS 85

9.10 Development Safety Update Report 85

9.11 Procedures to be followed in the event of abnormal findings 85

9.12 Interim Reviews 85

9.13 Data Safety Monitoring Board 86

9.14 Safety Group Holding Rules 86

9.14.1 Group holding rules 87

9.14.2 Individual stopping rules (will apply to prime-boost group only) 88

10 STATISTICS 90

10.1 Description of Statistical Methods 90
10.2 Primary efficacy 90
10.3 Safety & Reactogenicity 91
10.4 Immunogenicity 91
10.5 Subgroup analyses 92
10.6 Interim and primary analyses of the primary outcome 92
10.7 Final Analysis 93
10.8 Procedure for Accounting for Missing, Unused, and Spurious Data. 93
10.9 Inclusion in Analysis 93
10.10 Interim analysis for the combined DSMB 93
11 DATA MANAGEMENT 94
11.1 Data Handling 94
11.2 Record Keeping 94
11.3 Source Data and Case Report Forms (CRFs) 95
11.4 Data Protection 95
11.5 Data Quality 96
11.6 Archiving 96
12 QUALITY CONTROL AND QUALITY ASSURANCE PROCEDURES 97
12.1 Investigator procedures 97
12.2 Monitoring 97
12.3 Protocol deviation 97
12.4 Audit & inspection 97
13 SERIOUS BREACHES 98
14 ETHICS AND REGULATORY CONSIDERATIONS 99
14.1 Declaration of Helsinki 99
14.2 Guidelines for Good Clinical Practice 99
14.3 Ethical and Regulatory Approvals 99

14.4 Volunteer Confidentiality 99

15 FINANCING AND INSURANCE 101

15.1 Financing 101

15.2 Insurance 101

15.3 Compensation 101

16 Publication Policy 102

17 DEVELOPMENT OF A NEW PRODUCT/PROCESS OR THE GENERATION OF INTELLECTUAL PROPERTY 103

1 SYNOPSIS

**Title**	A phase I/II study to determine efficacy, safety and immunogenicity of the candidate Coronavirus Disease (COVID-19) vaccine ChAdOx1 nCoV-19 in UK healthy adult volunteers.
**Trial Identifier**	COV001
**Trial Registration**	EudraCT number: 2020-001072-15<br>REC Reference: 20/SC/0145<br>IRAS Reference: 281259
**Chief Investigator**	Professor Andrew Pollard
**Clinical Phase**	I/II
**Design**	Single-blinded, randomised, controlled, multi-centre
**Population**	Healthy adults aged 18-55 years
**Planned Sample Size**	Up to 1090

Group	W0	W4	W8 (-7/+14 days)	Booster
1a (n=44) Intense Follow-up	ChAdOx1 nCoV-19 $5x10^{10}$vp	-		
1b (n=44) Intense Follow-up	MenACWY	-		
2a* (n= up to 206)	ChAdOx1 nCoV-19 $5x10^{10}$vp	-		
2b* (n= up to 206)	MenACWY	-		

2c* Prime-boost (up to 20 volunteers from 2a)	ChAdOx1 nCoV-19 $5x10^{10}$vp		ChAdOx1 nCoV-19 $5x10^{10}$vp	
2d* Prime-boost (up to 32 volunteers from 2a)	ChAdOx1 nCoV-19 $5x10^{10}$vp		ChAdOx1 nCoV-19 $2.5x10^{10}$vp	
2e* Prime-boost (up to 10 volunteers from 2b)	MenACWY		MenACWY	
2f* Prime-boost (remaining participants from 2a, n=up to 154)	ChAdOx1 nCoV-19 $5x10^{10}$vp			ChAdOx1 nCoV-19 0.5mL (3.5-$6.5x10^{10}$vp)
2g* Prime-boost (remaining participants from 2b, n= up to 196)	MenACWY			MenACWY
3 (n=10) Prime-boost	ChAdOx1 nCoV-19 $5x10^{10}$vp	ChAdOx1 nCoV-19 $5x10^{10}$vp		
4a** (n= up to 290)	ChAdOx1 nCoV-19 $5x10^{10}$ vp	-		
4b** (n= up to 290)	MenACWY	-		
4c Prime-boost (n= up to 290)	ChAdOx1 nCoV-19 $5x10^{10}$ vp			ChAdOx1 nCoV-19 0.5mL (3.5-$6.5x10^{10}$vp)
4d Prime-boost (n= up to 290)	MenACWY			MenACWY

	* Group 2 will consist of an overall sample size of up to 412 volunteers, of which up to 62 (52 IMP and 10 controls) will receive a booster dose at 8 weeks (-7/+14 days). The remaining participants in Group 2 will be invited to receive a booster dose at the earliest opportunity (minimum 4 weeks).<br>**Group 4 will consist of an overall sample size of up to 580 volunteers, of which up to 112 will be given Paracetamol at D0 visit. All volunteers in Group 4 will be invited to receive a booster dose at the earliest opportunity (minimum 4 weeks).

Visit Schedule: See schedule of attendances for different groups

**Planned Trial Duration**	12 months from last vaccination visit (approximately 15 months from enrolment for participants receiving 2 doses)

	Objective	Outcome Measure
**Primary**	To assess efficacy of the candidate ChAdOx1 nCoV-19 against COVID-19	a) Virologically confirmed (PCR* positive) symptomatic cases of COVID-19
**Co-Primary**	To assess the safety of the candidate vaccine ChAdOx1 nCoV	a) occurrence of serious adverse events (SAEs) throughout the study duration.
**Secondary**	To assess the safety, tolerability and reactogenicity profile of the candidate vaccine ChAdOx1 nCoV	a) occurrence of solicited local reactogenicity signs and symptoms for 7 days following vaccination;<br>b) occurrence of solicited systemic reactogenicity signs and symptoms for 7 days following vaccination;<br>c) occurrence of unsolicited adverse events (AEs) for 28 days following vaccination;

		d) change from baseline for safety laboratory measures and;<br>e) Occurrence of disease enhancement episodes
	To assess efficacy of the candidate ChAdOx1 nCoV-19 against severe and non-severe COVID-19	a) Hospital admissions associated with COVID-19<br>b) Intensive care unit (ICU) admissions associated with COVID-19<br>c) Deaths associated with COVID-19<br>d) Severe COVID-19 disease (defined according to clinical severity scales).<br>e) Seroconversion against non-Spike SARS-CoV-2 antigens
	To assess cellular and humoral immunogenicity of ChAdOx1 nCoV-19	a) Interferon-gamma (IFN-γ) enzyme-linked immunospot (ELISpot) responses to SARS-CoV-2 spike protein;<br>b) Quantify antibodies against SARS-CoV-2 spike protein (seroconversion rates)
**Exploratory**	Exploratory Immunology	a) virus neutralising antibody (NAb) assays against live and/or pseudotype SARS-CoV-2 virus<br>b)Cell analysis by flow cytometry assays<br>c) Functional antibody assays
	To assess safety, reactogenicity, immunogenicity and efficacy endpoints, for participants receiving prophylactic paracetamol	All safety, reactogenicity, immunogenicity and efficacy endpoints.
	To assess immunogenicity of ChAdOx1 nCoV-19 given as homologous prime-boost	Quantify antibodies against SARS-CoV-2 spike protein (seroconversion rates) post boost

	To compare viral shedding on stool samples of SARS-CoV-2 PCR* positive individuals	Differences in viral shedding on stool at 7 days and beyond post SARS-CoV-2 positivity.

* Or other nucleic acid amplification test (NAAT)

Sample analysis for the completion of exploratory endpoints may be performed under the OVC Biobank research tissue bank protocol (REC: 16/SC/0141).

**Investigational products**	a) ChAdOx1 nCoV-19, a replication-deficient simian adenoviral vector expressing the spike (S) protein of SARS-CoV-2<br>b) MenACWY, Meningococcal Group A, C, W-135 and Y conjugate vaccine
**Formulation**	ChAdOx1 nCoV-19: Liquid<br>MenACWY: powder and solvent for solution for injection
**Route of Administration**	IM<br>ChAdOx1 nCoV-19/MenACWY: Intramuscularly (IM) into the deltoid region of the arm
**Dose per Administration**	ChAdOx1 nCoV-19: 5×10^{10} vp<br>ChAdOx1 nCoV-19: 2.5×10^{10} vp<br>ChAdOx1 nCoV-19: 0.5mL ($3.5\text{-}6.5 \times 10^{10}$vp)<br>MenACWY: 0.5mL

2 ABBREVIATIONS

**AdHu**	Human adenovirus
**AdHu5**	Human adenovirus serotype 5
**AE**	Adverse event
**AID**	Autoimmune Disease
**CCVTM**	Centre for Clinical Vaccinology and Tropical Medicine, Oxford
**CBF**	Clinical BioManufacturing Facility
**CEF**	Chick embryo fibroblast
**ChAd63**	Chimpanzee adenovirus 63
**CI**	Confidence interval
**COP**	Code of Practice
**CRF**	Case Report Form or Clinical Research Facility
**CTRG**	Clinical Trials & Research Governance Office, Oxford University
**CTL**	Cytotoxic T Lymphocyte
**DSUR**	Development Safety Update Report
**ELISPOT**	Enzyme-linked immunospot
**GCP**	Good Clinical Practice
**GMO**	Genetically modified organism
**GMT**	Geometric Mean Titre
**GP**	General Practitioner
**HCG**	Human Chorionic Gonadotrophin
**HBV**	Hepatitis B virus
**HEK**	Human embryonic kidney
**HCV**	Hepatitis C virus
**HIV**	Human immunodeficiency virus
**HLA**	Human leukocyte antigen
**HRA**	Health Research Authority
**HTLV**	Human T-Lymphotrophic Virus
**IB**	Investigator Brochure
**ICH**	*International Conference on Harmonisation*
ICMJE	*International Committee of Medical Journal Editors*
ICS	*Intracellular Cytokine Staining*
**ID**	Intradermal
**IFNγ**	Interferon gamma
**IM**	Intramuscular
**IMP**	Investigational Medicinal Product
**IMP-D**	Investigational Medicinal Product Dossier
**IV**	Intravenous
**MenACWY**	Quadrivalent capsular group A, C, W and Y meningococcal protein-polysaccharide conjugate vaccine
**MHRA**	Medicines and Healthcare Products Regulatory Agency
**MVA**	Modified vaccinia virus Ankara
**NAAT**	Nucleic acid amplification test
**NHS**	National Health Service
**NIH**	National Institutes of Health
**NIHR**	National Institute for Health Research
**PBMC**	*Peripheral blood mononuclear cell*
**PCR**	Polymerase chain reaction

**PI**	Principal Investigator
**QP**	Qualified Person
**qPCR**	Quantitative polymerase chain reaction
**REC**	Research Ethics Committee
**SAE**	Serious adverse event
**SC**	Subcutaneous
**SmPc**	Summary of Product characteristics
**SOP**	Standard Operating Procedure
**SUSAR**	Suspected unexpected serious adverse reaction
**µg**	microgram
**vp**	viral particle
**VV**	viral vector
**WHO**	World Health Organisation

3 BACKGROUND AND RATIONALE

3.1 Background

In December 2019, a cluster of patients with pneumonia of unknown cause was linked to a seafood wholesale market in Wuhan, China and were later confirmed to be infected with a novel coronavirus, known as 2019-nCoV [1]. The virus was subsequently renamed to SARS-CoV-2 because it is similar to the coronavirus responsible for severe acute respiratory syndrome (SARS-CoV), a lineage B betacoronavirus. SARS-CoV-2 shares more than 79% of its sequence with SARS-CoV, and 50% with the coronavirus responsible for Middle East respiratory syndrome (MERS-CoV), a member of the lineage C betacoronavirus [2]. COVID-19 is the infectious disease caused by SARS-CoV-2. By January 2020 there was increasing evidence of human to human transmission as the number of cases rapidly began to increase in China. Despite unprecedented containment measures adopted by the Chinese government, SARS-CoV-2 rapidly spread across the world. The WHO declared the COVID-19 outbreak a public health emergency of international concern on 30th January 2020. As of 10th March 2020, over 118,000 cases have been reported with more than 4200 deaths and 115 countries affected.

Coronaviruses (CoVs) are spherical, enveloped, large positive-sense single-stranded RNA genomes. One-fourth of their genome is responsible for coding structural proteins, such as the spike (S) glycoprotein, envelope (E), membrane (M) and nucleocapsid (N) proteins. E, M, and N are mainly responsible for virion assembly whilst the S protein is involved in receptor binding, mediating virus entry into host cells during CoVs infection via different receptors.[3] SARS-CoV-2 belongs to the phylogenetic lineage B of the genus *Betacoronavirus* and it recognises the angiotensin-converting enzyme 2 (ACE2) as the entry receptor [4]. It is the seventh CoV known to cause human infections and the third known to cause severe disease after SARS-CoV and MERS-CoV.

The spike protein is a type I, trimeric, transmembrane glycoprotein located at the surface of the viral envelope of CoVs, which can be divided into two functional subunits: the N-terminal S1 and the C-terminal S2. S1 and S2 are responsible for cellular receptor binding via the receptor binding domain (RBD) and fusion of virus and cell membranes respectively, thereby mediating the entry of SARS-CoV-2 into target cells.[3] The roles of S in receptor binding and membrane fusion make it an ideal target for vaccine and antiviral development, as it is the main target for neutralising antibodies.

ChAdOx1 nCoV-19 vaccine consists of the replication-deficient simian adenovirus vector ChAdOx1, containing the structural surface glycoprotein (Spike protein) antigen of the SARS CoV-2 (nCoV-19), with a leading tissue plasminogen activator (tPA) signal sequence. ChAdOx1 nCoV-19 expresses a codon-optimised coding sequence for the Spike protein from genome sequence accession GenBank:

MN908947. The tPA leader sequence has been shown to be beneficial in enhancing immunogenicity of another ChAdOx1 vectored CoV vaccine (ChAdOx1 MERS) [5].

3.2 Pre-Clinical Studies

Refer to the Investigator Brochure for most recent pre-clinical data update

3.2.1 Immunogenicity (Jenner Institute, unpublished)

Mice (balb/c and CD-1) were immunised with ChAdOx1 expressing SARS-CoV-2 Spike protein or green fluorescent protein (GFP). Spleens were harvested for assessment of IFY ELISpot responses and serum samples were taken for assessments of S1 and S2 antibody responses on ELISA at 9 or 10 days post vaccination. The results of this study show that a single dose of ChAdOx1 nCoV was immunogenic in mice.

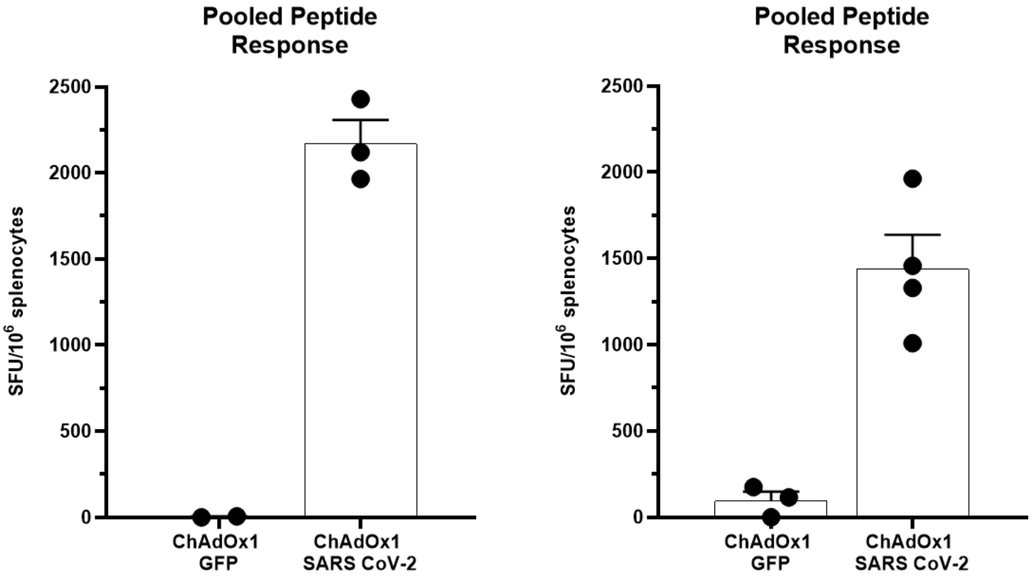

Figure 1. Summed splenic IFN-γ ELISpot responses of BALB/c (left panel) and CD-1 (right panel) mice, in response to peptides spanning the spike protein from SARS-CoV-2, nine or ten days post vaccination, with 1.7×10^{10} vp ChAdOx1 nCoV-19 or 8×10^{9} vp ChAdOx1 GFP. Mean with SEM are depicted

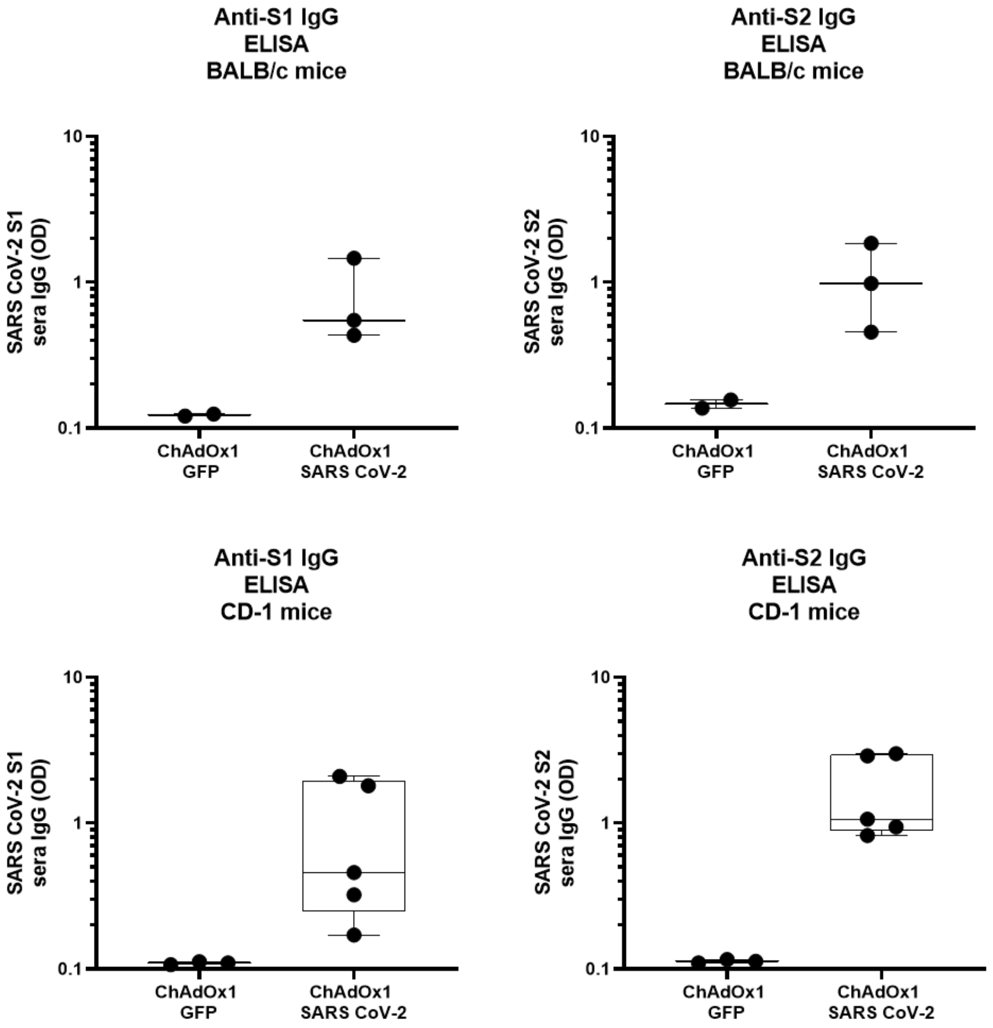

Figure 2. Box and whisker plot of the optical densities following ELISA analysis of BALB/C mouse sera (Top panel) incubated with purified protein spanning the S1 domain (left) or purified protein spanning the S2 domain (right) of the SARS-CoV-2 spike nine or ten days post vaccination, with 1.7×10^{10} vp ChAdOx1 nCoV-19 or 8×10^{9} vp ChAdOx1 GFP. Box and whisker plots of the optical densities following ELISA analysis of CD-1 mouse sera (Bottom panel) incubated with purified protein spanning the S1 domain (left) or purified protein spanning the S2 domain (right) of the SARS-CoV-2 spike.

3.2.2 Efficacy

Pre-clinical efficacy studies of ChAdOx1 nCoV-19 in ferrets and non-human primates are underway. Results will be included in the Investigator's Brochure when available.

3.3 Disease Enhancement

Safety concerns around the use of full length coronavirus Spike glycoproteins and other viral antigens (nucleoprotein) as a vaccine antigen have been raised following historical and limited reports of

immunopathology and antibody dependant enhancement (ADE) reported in vitro and post SARS-CoV challenge in mice, ferrets and non-human primates immunised with whole SARS-CoV inactivated or full-length S protein based vaccines, including a study using Modified Vaccinia Ankara as a vector.[6-8] To date, there has been one report of lung immunopathology following MERS-CoV challenge in mice immunised with an inactivated MERS-CoV candidate vaccine.[9] However, in preclinical studies of ChAdOx1 immunisation and MERS-CoV challenge, no ADE was observed in hDPP4 transgenic mice, dromedary camels or non-human primates (van Doremalen et al, manuscript submitted).[10, 11]

The risks of inducing lung immunopathology in the event of COVID-19 disease following ChAdOx1 nCoV-19 vaccination are unknown. Challenge studies on ferrets and NHPs are underway and these pre-clinical studies will report on presence or absence of lung pathology. Results will be reviewed as soon as they emerge and will inform discussions on risk/benefit to participants receiving the IMP. All pathology data arising from challenge studies of other SARS-CoV-2 vaccine candidates will also be taken into account.

3.4 Previous clinical experience

This will be the first-in-human study employing ChAdOx1 nCoV-19. However, ChAdOx1 vectored vaccines expressing different inserts have previously been used in over 320 healthy volunteers taking part in clinical trials conducted by or in partnership with the University of Oxford in the UK and overseas (table 1 and 2). Most importantly, a ChAdOx1 vectored vaccine expressing the full-length Spike protein from another Betacoronavirus, MERS-CoV, has been given to 31 participants to date as part of MERS001 and MERS002 trials. ChAdOx1 MERS was given at doses ranging from $5x10^9$ vp to $5x10^{10}$ vp (table 2) with no serious adverse reactions reported. Further safety and immunogenicity results on ChAdOx1 MERS can be found on the Investigator's Brochure for ChAdOx1 nCoV-19 for reference.

Clinical trials of ChAdOx1 vectored vaccines encoding antigens for Influenza (fusion protein NP+M1), Tuberculosis (85A), Prostate Cancer (5T4), Malaria (LS2), Chikungunya (structural polyprotein), Zika (prM and E), MERS-CoV (full-length Spike protein) and Meningitis B are listed below.

None of the below mentioned clinical trials reported serious adverse events associated with the administration of ChAdOx1, which was shown to have a good safety profile.

Table 1. Clinical experience with ChAdOx1 viral vector vaccines.

Country	Trial	Vaccine	Age	Route	Dose	Number of Volunteers (Received ChAdOx1)	Publication / Registration Number
UK	FLU004	ChAdOx1 NP+M1	18-50	IM	$5x10^8$ vp	3	Antrobus et al, 2014. Molecular Therapy.<br>DOI: 10.1038/mt.2013.284<br>[12]
					$5x10^9$ vp	3	
					$2.5x10^{10}$ vp	3	
					$5x10^{10}$ vp	6	
UK	FLU005	ChAdOx1 NP+M1<br>MVA NP+M1 (week 8)	18-50	IM	$2.5x10^{10}$ vp	12	Coughlan et al, 2018. EBioMedicine<br>DOI: 10.1016/j.ebiom.2018.02.011<br>DOI: 10.1016/j.ebiom.2018.05.001<br>[13]
		ChAdOx1 NP+M1<br>MVA NP+M1 (week 52)	18-50	IM	$2.5x10^{10}$ vp	12	
		MVA NP+M1<br>ChAdOx1 NP+M1 (week 8)	18-50	IM	$2.5x10^{10}$ vp	12	
		MVA NP+M1<br>ChAdOx1 NP+M1 (week 52)	18-50	IM	$2.5x10^{10}$ vp	9	
		ChAdOx1 NP+M1	>50	IM	$2.5x10^{10}$ vp	12	
		ChAdOx1 NP+M1<br>MVA NP+M1 (week 8)	>50	IM	$2.5x10^{10}$ vp	12	

Country	Trial	Vaccine	Age	Route	Dose	Number of Volunteers (Received ChAdOx1)	Publication / Registration Number
UK	TB034	ChAdOx1 85A	18-50	IM	$5x10^9$ vp	6	Wilkie et al, 2020 Vaccine<br>DOI: 10.1016/j.vaccine.2019.10.102<br>[14]
					$2.5x10^{10}$ vp	12	
		ChAdOx1 85A<br>MVA85A (week 8)	18-50	IM	$2.5x10^{10}$ vp	12	
		ChAdOx1 85A<br>(x2, 4weeks apart)<br>MVA85A (at 4 months)	18-50	IM	$2.5x10^{10}$ vp	12	
Switzerland	TB039<br>(ongoing)	ChAdOx1 85A	18-55	Aerosol	$1x10^9$ vp	3	Clinicaltrials.gov:<br>NCT04121494
				Aerosol	$5x10^9$ vp	3	
				Aerosol	$1x10^{10}$ vp	11	
				Aerosol/IM	$1x10^{10}$ vp	15	
Uganda	TB042<br>(ongoing)	ChAdOx1 85A	18-49	IM	$5x10^9$ vp	6	Clinicaltrials.gov:<br>NCT03681860
					$2.5 x10^{10}$	6	
UK	VANCE01	ChAdOx1.5T4<br>MVA.5T4	18 – 75	IM	$2.5x10^{10}$ vp	34	Clinicaltrials.gov:<br>NCT02390063

Country	Trial	Vaccine	Age	Route	Dose	Number of Volunteers (Received ChAdOx1)	Publication / Registration Number
UK	ADVANCE (ongoing)	ChAdOx1.5T4<br>MVA.5T4	≥18	IM	2.5×10^{10} vp	23 (as of Feb 20)	Clinicaltrials.gov:<br>NCT03815942
UK	VAC067	ChAdOx1 LS2	18-45	IM	5×10^{9} vp	3	Clinicaltrials.gov:<br>NCT03203421
					2.5×10^{10} vp	10	
UK	VAMBOX	ChAdOx1 MenB.1	18-50	IM	2.5×10^{10} vp	3	ISRCTN46336916
					5×10^{10} vp	26	
UK	CHIK001	ChAdOx1 Chik	18-50	IM	5×10^{9} vp	6	Clinicaltrials.gov:<br>NCT03590392<br>DOI: https://doi.org/10.4269/ajtmh.abstract2019<br>Abstract #59, page 19.
					2.5×10^{10} vp	9	
					5×10^{10} vp	9	
UK	ZIKA001 (ongoing)	ChAdOx1 Zika	18-50	IM	5×10^{9} vp	6	Clinicaltrials.gov:<br>NCT04015648
					2.5×10^{10} vp	3 (as of Feb 20)	
					5×10^{10} vp	-	

Table 2. Clinical experience with ChAdOx1 MERS

Country	Trial	Vaccine	Age	Route	Dose	Number of Volunteers (Received ChAdOx1)	Publication / Registration Number
UK	MERS001 (ongoing)	ChAdOx1 MERS	18-50	IM	$5x10^9$ vp	6	Clinicaltrials.gov: NCT03399578 DOI: https://doi.org/10.1016/S1473-3099(20)30160-2 Folegatti et.al. 2020, Lancet Infect.Dis. **[15]**
					$2.5x10^{10}$ vp	9	
					$5x10^{10}$ vp	9	
					$2.5x10^{10}$ vp (homologous prime-boost)	3	
Saudi Arabia	MERS002 (ongoing)	ChAdOx1 MERS	18-50	IM	$5x10^9$ vp	4	Clinicaltrials.gov: NCT04170829
					$2.5x10^{10}$ vp	3	
					$5x10^{10}$ vp	-	

3.5 Rationale

The COVID-19 epidemic has caused major disruption to healthcare systems with significant socioeconomic impacts. Containment measures have failed to stop the spread of virus, which is now at pandemic levels. There are currently no specific treatments available against COVID-19 and accelerated vaccine development is urgently needed.

Live attenuated viruses have historically been among the most immunogenic platforms available, as they have the capacity to present multiple antigens across the viral life cycle in their native conformations. However, manufacturing live-attenuated viruses requires complex containment and biosafety measures. Furthermore, live-attenuated viruses carry the risks of inadequate attenuation causing disseminated disease, particularly in immunocompromised hosts. Given that severe disease and fatal COVID-19 disproportionally affect older adults with co-morbidities, making a live-attenuated virus vaccine is a less viable option. Replication competent viral vectors could pose a similar threat for disseminated disease in the immuno-suppressed. Replication deficient vectors, however, avoid that risk while maintaining the advantages of native antigen presentation, elicitation of T cell immunity and the ability to express multiple antigens [16]. Subunit vaccines usually require the use of adjuvants and whilst DNA and RNA vaccines can offer manufacturing advantages, they are often poorly immunogenic requiring multiple doses, which is highly undesirable in the context of a pandemic.

Chimpanzee adenovirus vaccine vectors have been safely administered to thousands of people using a wide range of infectious disease targets. ChAdOx1 vectored vaccines have been given to over 320 volunteers with no safety concerns and have been shown to be highly immunogenic at single dose administration. Of relevance, a single dose of a ChAdOx1 vectored vaccine expressing full-length spike protein from another betacoronavirus (MERS-CoV) has shown to induce neutralising antibodies in recent clinical trials.

Data generated in this study will be used to support further larger phase II/III efficacy studies, which will include target groups at higher risk of severe disease.

The use of an active comparator (MenACWY) will minimise the chances of accidental participant unblinding, decreasing bias in reactogenicity or safety reporting and/or health seeking behaviours once symptomatic for COVID-19. The use of prophylactic paracetamol reduces the incidence and severity of fever and other adverse events following immunisation, and it has been previously recommended following Meningococcal B vaccine administration without negatively impacting its immunogenicity profile (reference: Bexsero SmPC). A prophylactic paracetamol dose arm has been introduced in order to assess safety, reactogenicity, immunogenicity and efficacy of the co-administration of paracetamol and ChAdOx1 nCoV-19 as an exploratory objective.

Whilst a single-dose regimen is the preferred option in the context of a pandemic, a two-dose schedule is likely to boost seroconversion rates and increase neutralising antibody levels, although correlates of protection for COVID-19 are still unknown. Groups 2c, d, and e have been added in order to gather additional evidence on immunogenicity of ChAdOx1 nCoV-19 given as part of the two-dose schedule.

A booster dose has been added to participants in groups 2 and 4 following interim immunogenicity results on homologous prime-boost groups, showing improved neutralising antibody titres after 2 doses when compared to a 1 dose regimen.

4 OBJECTIVES AND ENDPOINTS

Objectives	Outcome Measures	Time point(s) of evaluation of this outcome measure
**Primary Objective**<br>To assess efficacy of ChAdOx1 nCoV-19 against COVID-19	a) Virologically confirmed (PCR* positive) symptomatic cases of COVID-19	Throughout the study
**Co-primary Objective**<br>To assess the safety of the candidate vaccine ChAdOx1 nCoV	a) occurrence of serious adverse events (SAEs) throughout the study duration	Throughout the study
**Secondary Objectives**<br>To assess the safety, tolerability and reactogenicity profile of the candidate vaccine ChAdOx1 nCoV	a) occurrence of solicited local reactogenicity signs and symptoms for 7 days following vaccination<br><br>b) occurrence of solicited systemic reactogenicity signs and symptoms for 7 days following vaccination;<br><br>c) occurrence of unsolicited adverse events (AEs) for 28 days following vaccination;<br><br>d) change from baseline for safety laboratory measures and;<br><br>f) Occurrence of SAE of special interest: disease enhancement episodes	Day 0-7<br>Self-reported symptoms recorded using electronic diaries<br><br>Day 0-7<br>Self-reported symptoms recorded using electronic diaries<br><br>Day 0-28<br>Self-reported symptoms recorded using electronic diaries<br><br>Blood samples drawn at enrolment (before vaccination), day 3, 7 and 28<br><br>Throughout the study
To assess efficacy of ChAdOx1 nCoV-19 against COVID-19	a) Hospital admissions associated with COVID-19<br><br>b) Intensive care unit (ICU) admissions associated with COVID-19	Throughout the study<br><br>Throughout the study<br><br>Throughout the study

	c) Deaths associated with COVID-19<br>d) Severe COVID-19 disease (defined according to clinical severity scales)<br><br>e) Seroconversion against non-Spike antigens	Throughout the study<br><br><br><br>Blood samples drawn at D0, D28 , D182 and D364
To assess cellular and humoral immunogenicity of ChAdOx1 nCoV-19	a) Interferon-gamma (IFN-γ) enzyme-linked immunospot (ELISpot) responses to SARS-CoV-2 spike protein;<br><br>b) Quantify antibodies against SARS-CoV-2 spike protein (seroconversion rates)	See schedule of attendances
**Exploratory Objectives**<br>Exploratory Immunology	a) virus neutralising antibody (NAb) assays against live and/or pseudotype SARS-CoV-2 virus<br>b) Cell analysis by flow cytometry assays<br>c) Functional antibody assays	See schedule of attendances
To assess safety, reactogenicity, immunogenicity and efficacy endpoints, for participants receiving prophylactic paracetamol	All safety, reactogenicity, immunogenicity and efficacy endpoints.	Throughout the study
To assess immunogenicity of ChAdOx1 nCoV-19 given as homologous prime-boost	Quantify antibodies against SARS-CoV-2 spike protein (seroconversion rates) post boost	Blood samples drawn at D0, D28, D56, D70, D84, D182 and D364
To compare viral shedding on stool samples of SARS-CoV-2 PCR* positive individuals	Differences in viral shedding on stool between vaccine and comparator arms	At approximately 7 days and beyond post SARS-CoV-2 PCR* positivity.

* Or other nucleic acid amplification test

Sample analysis for the completion of exploratory endpoints may be performed under the ethically approved OVC Biobank protocol.

5 TRIAL DESIGN

This is a Phase I/II, single-blinded, -controlled, individually randomised study in healthy adults aged 18-55 years recruited in the UK. ChAdOx1 nCoV-19 or active control (licensed MenACWY) will be administered via an intramuscular injection into the deltoid. The study will assess efficacy, safety and immunogenicity of ChAdOx1 nCoV-19. Additional steps may be taken to keep clinical investigators assessing the primary efficacy endpoint blinded to group allocation with an aim to minimise unblinding of participants, where this is possible and practical to do so.

There will be 4 study groups with up to 540 volunteers in each of the single dose vaccine arms (ChAdOx1 nCoV-19 or licensed MenACWY) in groups 1, 2 & 4 combined and 10 participants in group 3 with an overall sample size of up to 1090 (Table 3). Randomisation will take place at an intervention to control ratio of 1:1. Only participants enrolled in groups 1, 2 and 4 will be randomised. Participants in group 3 will not be randomised or blinded. Up to 112 participants in group 4 will be requested to take prophylactic paracetamol 1000mg every 6 hours for 24 hours from the time of vaccination to reduce the chance of fever post immunisation. Participants receiving a booster dose in groups 2f, 2g, 4c and 4d will be advised to take prophylactic paracetamol for 24h post booster vaccine.

Staggered enrolment will apply to the first volunteers receiving the IMP as described in section 7.4.2.2. Participants will be first recruited in groups 1 and 3. Once groups 1 and 3 are fully recruited, subsequent volunteers will be enrolled in groups 2 and 4.

Safety will be assessed in real time and interim reviews are scheduled after 1, 4, and up to 54 participants received the IMP. Randomisation blocks will ensure there is at least 1 control for each participant receiving the IMP, so these safety reviews will take place after 2, 8, and up to 98 participants are enrolled in the study overall (groups 1 and 3).

Up to 62 participants enrolled in group 2 (a and b) will be invited to receive a booster vaccine. Participants in groups 2c and 2d will be randomised to receive either a standard booster dose ($5x10^{10}$vp), or a lower booster dose ($2.5x10^{10}$vp) at approximately 8 weeks post prime. Up to 10 volunteers from 2b will be receive a second dose of MenACWY at the same interval. The remaining participants in groups 2 and 4 (those who have not already been boosted) will be invited to receive a booster dose of either ChAdOx1 nCoV-19: 0.5mL (3.5-6.5x1010vp) or MenACWY. Volunteers in groups 2f, 2g, 4c and 4d will be advised to take prophylactic paracetamol for 24h post booster dose.

The DSMB will periodically assess safety and efficacy data every 4-8 weeks and/or as required.

Participants will be followed over the duration of the study to record adverse events and episodes of virologically confirmed symptomatic COVID-19 cases. Participants will be tested for COVID-19 if they present with a new onset of fever (≥37.8 C) OR cough OR shortness of breath OR anosmia/ageusia.

Moderate and Severe COVID-19 disease will be defined using clinical criteria. Detailed clinical parameters will be collected from medical records and aligned with agreed definitions as they emerge. These are likely to include, but are not limited to, oxygen saturation, need for oxygen therapy, respiratory rate and other vital signs, need for ventilatory support, Xray and CT scan imaging and blood test results, amongst other clinically relevant parameters.

5.1 Study groups

Group	Number of Volunteers	Dose	Route
1a	44	ChAdOx1 nCoV-19<br>$5x10^{10}$ vp	IM
1b	44	MenACWY	IM
2a*	Up to 206	ChAdOx1 nCoV-19<br>$5x10^{10}$ vp	IM
2b*	Up to 206	MenACWY	IM
2c*	Up to 20 volunteers from 2a	Homologous Prime-Boost<br>$5x10^{10}$ vp, 8 weeks apart (-7/+14 days)	IM
2d*	Up to 32 volunteers from 2a	Homologous Prime-Boost<br>$5x10^{10}$ vp (prime) and $2.5x10^{10}$ vp (boost) 8 weeks apart (-7/+14 days)	IM
2e*	Up to 10 volunteers from 2b	Two-dose MenACWY 8 weeks apart (-7/+14 days)	IM
2f*	Up to 154 volunteers from 2a	Homologous Prime-Boost<br>$5x10^{10}$ vp prime and 0.5mL ($3.5\text{-}6.5x10^{10}$vp) boost, at the earliest opportunity (minimum 4 weeks apart)	IM
2g*	Up to 196 volunteers from 2b	Two-dose MenACWY at the earliest opportunity (minimum 4 weeks apart)	IM
3	10	Homologous Prime-Boost	IM

		$5x10^{10}$ vp	
4a **	Up to 290	ChAdOx1 nCoV-19<br>$5x10^{10}$ vp	IM
4b**	Up to 290	MenACWY	IM
4c**	Up to 290 from 4a	Homologous Prime-Boost<br>$5x10^{10}$ vp prime and 0.5mL (3.5-6.5x10^{10}vp) boost, at the earliest opportunity (minimum 4 weeks apart)	IM
4d**	Up to 290 from 4b	Two-dose MenACWY at the earliest opportunity (minimum 4 weeks apart)	IM

* Group 2 will consist of an overall sample size of up to 412 volunteers, of which up to 62 (52 IMP and 10 controls) will receive a booster dose at 8 weeks (-7/+14 days), and the remainder will be invited to receive a booster dose at the earliest opportunity (minimum 4 weeks apart).

**Group 4 will consist of an overall sample size of up to 580 volunteers, of which up to 112 will be given Paracetamol at D0 visit. All volunteers in Group 4 will be invited to receive a booster dose at the earliest opportunity (minimum 4 weeks apart).

5.2 Trial volunteers

Healthy adult volunteers aged 18-55 will be recruited into the study. Volunteers will be considered enrolled immediately following administration of first vaccination.

5.3 Definition of End of Trial

The end of the trial is the date of the last assay conducted on the last sample collected.

5.4 Duration of study

The total duration of the study will be 12 months from the day of the last vaccination dose.

5.5 Potential Risks for volunteers

The potential risks are those associated with phlebotomy, vaccination and disease enhancement

Venepuncture

Localised bruising and discomfort can occur at the site of venepuncture. Infrequently fainting may occur. These will not be documented as AEs if they occur. The total volume of blood drawn over a six month period will be 177.5-621.5mL (blood volumes may vary slightly for volunteers at different investigator sites due to use of different volume vacutainers, following local Trust SOPs). This should not compromise these otherwise healthy volunteers, as they would donate 470mL during a single blood donation for the National Blood transfusion Service over a 3-4 month period. Volunteers will be asked to refrain from blood donation for the duration of their involvement in the trial.

Allergic reactions

Allergic reactions from mild to severe may occur in response to any constituent of a medicinal product's preparation. Anaphylaxis is extremely rare (about 1 in 1,000,000 vaccine doses) but can occur in response to any vaccine or medication.

Vaccination

Local reaction from IM vaccination

The typical local reaction as a result of IM injection is temporary pain, tenderness, redness, and swelling at the site of the injection.

Systemic reactions

Constitutional influenza-like symptoms such as fatigue, headache, malaise, feverishness, and muscle aches can occur with any vaccination and last for 2-3 days. Presyncopal and syncopal episodes may occur at the time of vaccination which rapidly resolve. For subset of participants in group 4, use of prophylactic paracetamol for 24 hours will be advised to alleviate potential fevers and flu-like symptoms. As with any other vaccine, temporary ascending paralysis (Guillain-Barré syndrome, GBS) or immune mediated reactions that can lead to organ damage may occur, but this should be extremely rare (1 in 100,000-1,000,000 vaccine doses).

Control participants will receive one or two doses of a licensed MenACWY vaccine, the risks of which are described in these vaccines SmPC.

Disease Enhancement

The risks of inducing disease enhancement and lung immunopathology in the event of COVID-19 disease following ChAdOx1 nCoV-19 vaccination are unknown as described above. Challenge studies on ferrets and NHPs are underway and results will be reviewed as they emerge. All pre-clinical data from challenge studies using ChAdOx1 nCoV-19 and other vaccine candidates (when available) will inform decisions on risk/benefit to participants receiving the IMP.

5.6 Known Potential Benefits

Volunteers enrolled into the control groups will receive 1-2 doses of MenACWY, a licensed vaccine that has been administered to teenagers in the UK routine schedule since 2015 and is used as a travel vaccine for high risk areas. The majority of participants in this study will not have had this vaccine previously, and therefore will gain the benefit of protection against group A, C, W and Y meningococcus. Those participants who have previously had MenACWY vaccines will have their immunity against these organisms boosted. Recipients of ChAdOx1 nCoV-19 do not have any guaranteed benefit. However, it is hoped that the information gained from this study will contribute to the development of a safe and effective vaccine against COVID-19. The only benefits for participants would be information about their general health status.

6 RECRUITMENT AND WITHDRAWAL OF TRIAL VOLUNTEERS

6.1 Identification of Trial Volunteers

Healthy adults in the UK will be recruited by use of an advertisement +/- registration form formally approved by the ethics committee(s) and distributed or posted in the following places:

- In public places, including buses and trains, with the agreement of the owner / proprietor.
- In newspapers or other literature for circulation.
- On radio via announcements.
- On a website or social media site operated by our group or with the agreement of the owner or operator (including on-line recruitment through our web-site).
- By e-mail distribution to a group or list only with the express agreement of the network administrator or with equivalent authorisation.
- By email distribution to individuals who have already expressed an interest in taking part in any clinical trial at the Oxford Vaccine Centre and other trial sites.
- On stalls or stands at exhibitions or fairs.
- Via presentations (e.g. presentations at lectures or invited seminars).
- Direct mail-out: This will involve obtaining names and addresses of adults via the most recent Electoral Roll. The contact details of individuals who have indicated that they do not wish to receive postal mail-shots would be removed prior to the investigators being given this information. The company providing this service is registered under the General Data Protection Regulation 2016/679. Investigators would not be given dates of birth or ages of individuals but the list supplied would only contain names of those aged between 18-55 years (as per the inclusion criteria).
- Direct mail-out using National Health Service databases: These include the National Health Applications and Infrastructure Services (NHAIS) via a NHAIS data extract or equivalent. Initial contact to potential participants will not be made by the study team. Instead study invitation material will be sent out on our behalf by an external company, CFH Docmail Ltd, in order to preserve the confidentiality of potential participants. CFH Docmail Ltd is accredited as having exceeded standards under the NHS Digital Data Security and Protection Toolkit (ODS ID – 8HN70).
- Oxford Vaccine Centre databases and other trial sites databases: We may contact individuals from databases of groups within the CCVTM (including the Oxford Vaccine Centre database) and other trial

sites of previous trial participants who have expressed an interest in receiving information about all future studies for which they may be eligible.

6.2 Informed consent

All volunteers will sign and date the informed consent form before any study specific procedures are performed. The information sheet will be made available to the volunteer at least 24 hours prior to the screening visit. At the screening visit, a video presentation of the aims of the study and all tests to be carried out may be screened to an audience, or made available for them to access it remotely. Individually each volunteer will have the opportunity to question an appropriately trained and delegated researcher before signing the consent. At the screening visit, the volunteer will be fully informed of all aspects of the trial, the potential risks and their obligations. The following general principles will be emphasised:

- Participation in the study is entirely voluntary
- Refusal to participate involves no penalty or loss of medical benefits
- The volunteer may withdraw from the study at any time.
- The volunteer is free to ask questions at any time to allow him or her to understand the purpose of the study and the procedures involved
- The study involves research of an investigational vaccine
- There is no direct benefit to the volunteer from participating
- The volunteer's GP will be contacted to corroborate their medical history. Written or verbal information regarding the volunteer's medical history will be sought from the GP or other sources. This can either be via the study team accessing patient's electronic care summaries, GP and other medical records from local systems, by contacting the GP practice, or volunteers bringing their medical care summaries from the GP to the study clinicians. However, volunteers may be enrolled based on medical information obtained during screening only, at the physician's discretion.
- Blood samples taken as part of the study may be sent outside of the UK and Europe to laboratories in collaboration with the University of Oxford. These will be anonymised. Volunteers will be asked if they consent to indefinite storage of any leftover samples for use in other ethically approved research, this will be optional.
- The volunteer will be registered on the TOPS database (The Over volunteering Prevention System; www.tops.org.uk).

The aims of the study and all tests to be carried out will be explained. The volunteer will be given the opportunity to ask about details of the trial, and will then have time to consider whether or not to participate. If they do decide to participate, they, and the investigator will sign and date the consent form. However, in the current crisis, there may be occasions when it is necessary for the consent form to be signed by an appropriately trained and delegated research nurse instead of the investigator. The participant would always have the opportunity to discuss the study with a medically qualified investigator if they wish. The volunteer will then be provided with a copy of the consent form to take away and keep, with the original being stored in the case report form (CRF). Reconsent will be taken by appropriately trained and delegated members of the team.

Participants are required to consent to receive an additional swab should they develop symptoms of COVID-19. Updated information will be sent to participants and written re-consent requested at the earliest scheduled visit. If the earliest visit to occur is in the symptomatic pathway, the participant may consent using an electronic signature on a tablet for infection control purposes.

6.3 Inclusion and exclusion criteria

This study will be conducted in healthy adults, who meet the following inclusion and exclusion criteria:

6.3.1 Inclusion Criteria

The volunteer must satisfy all the following criteria to be eligible for the study:

- Healthy adults aged 18-55 years.
- Able and willing (in the Investigator's opinion) to comply with all study requirements (participants must not rely on public transport or taxis).
- Willing to allow the investigators to discuss the volunteer's medical history with their General Practitioner and access all medical records when relevant to study procedures.
- For females only, willingness to practice continuous effective contraception (see below) during the study and a negative pregnancy test on the day(s) of screening and vaccination.
- Agreement to refrain from blood donation during the course of the study.
- Provide written informed consent.

6.3.2 Exclusion Criteria

The volunteer may not enter the study if any of the following apply:

- Prior receipt of any vaccines (licensed or investigational) ≤30 days before enrolment

- Planned receipt of any vaccine other than the study intervention within 30 days before and after each study vaccination with the exception of the licensed seasonal influenza vaccination and the licensed pneumococcal vaccine. Participants will be encouraged to receive these vaccinations at least 7 days before or after their study vaccine.
- Prior receipt of an investigational or licensed vaccine likely to impact on interpretation of the trial data (e.g. Adenovirus vectored vaccines, any coronavirus vaccines)
- Administration of immunoglobulins and/or any blood products within the three months preceding the planned administration of the vaccine candidate.
- Any confirmed or suspected immunosuppressive or immunodeficient state, including HIV infection; asplenia; recurrent severe infections and use of immunosuppressant medication within the past 6 months, except topical steroids or short-term oral steroids (course lasting <14 days) .
- Any autoimmune conditions, except mild psoriasis, well-controlled autoimmune thyroid disease, vitiligo or stable coeliac disease not requiring immunosuppressive or immunomodulatory therapy.
- History of allergic disease or reactions likely to be exacerbated by any component of the ChAdOx1 nCoV-19 or MenACWY vaccines.
- Any history of angioedema.
- Any history of anaphylaxis.
- Pregnancy, lactation or willingness/intention to become pregnant during the study.
- History of cancer (except basal cell carcinoma of the skin and cervical carcinoma in situ).
- History of serious psychiatric condition likely to affect participation in the study (e.g. ongoing severe depression, history of admission to an in-patient psychiatric facility, recent suicidal ideation, history of suicide attempt, bipolar disorder, personality disorder, alcohol and drug dependency, severe eating disorder, psychosis, use of mood stabilisers or antipsychotic medication).
- Bleeding disorder (e.g. factor deficiency, coagulopathy or platelet disorder), or prior history of significant bleeding or bruising following IM injections or venepuncture.
- Any other serious chronic illness requiring hospital specialist supervision.
- Chronic respiratory diseases, including mild asthma (resolved childhood asthma is allowed)
- Chronic cardiovascular disease (including hypertension), gastrointestinal disease, liver disease (except Gilberts Syndrome), renal disease, endocrine disorder (including diabetes) and neurological illness (excluding migraine)

- Seriously overweight (BMI≥40 Kg/m^2) or underweight (BMI≤18 Kg/m^2)
- Suspected or known current alcohol abuse as defined by an alcohol intake of greater than 42 units every week.
- Suspected or known injecting drug abuse in the 5 years preceding enrolment.
- Any clinically significant abnormal finding on screening biochemistry, haematology blood tests or urinalysis.
- Any other significant disease, disorder or finding which may significantly increase the risk to the volunteer because of participation in the study, affect the ability of the volunteer to participate in the study or impair interpretation of the study data.
- History of laboratory confirmed COVID-19.
- New onset of fever or a cough or shortness of breath or anosmia/ageusia since February 2020. Should a reliable test become available, this exclusion criteria will be replaced with seropositivity for SARS-CoV-2 before enrolment.
- Those who have been at high risk of exposure before enrolment, including but not limited to: close contacts of confirmed COVID-19 cases, anyone who had to self-isolate as a result of a symptomatic household member, frontline healthcare professionals working in A&E, ICU and other higher risk areas. Should a reliable test become available, this exclusion criteria will be replaced with seropositivity for SARS-CoV-2 before enrolment.
- Living in the same household as any vulnerable groups at risk of severe COVID-19 disease (as per PHE guidance)

Additional exclusion criteria (subset of participants receiving Paracetamol in group 4 only)

- History of allergic disease or reactions likely to be exacerbated by Paracetamol

6.3.3 Re-vaccination exclusion criteria

The following AEs associated with any vaccine, or identified on or before the day of vaccination constitute absolute contraindications to further administration of an IMP to the volunteer in question. If any of these events occur during the study, the subject will not be eligible to receive a booster dose and will be followed up by the clinical team or their GP until resolution or stabilisation of the event:

- Anaphylactic reaction following administration of vaccine
- Pregnancy
- Any AE that in the opinion of the Investigator may affect the safety of the participant or the interpretation of the study results

Participants who develop COVID-19 symptoms and have a positive NAAT test after the first vaccination can only receive a booster dose after a minimum 4 weeks interval from their first NAAT positive test, provided their symptoms have significantly improved. The decision to proceed with booster vaccinations in those cases will be at clinical discretion of the investigators. For participants who are asymptomatic and have a positive NAAT test, a minimum of 2 weeks from first NAAT positivity will be required before boosting.

6.3.4 Effective contraception for female volunteers

Female volunteers of childbearing potential are required to use an effective form of contraception at least during the first 3 months after their booster vaccination (groups 2-4) and the first 3 months after their single dose vaccine administration (group 1).

Acceptable forms of contraception for female volunteers include:

- Established use of oral, injected or implanted hormonal methods of contraception.
- Placement of an intrauterine device (IUD) or intrauterine system (IUS).
- Total abdominal hysterectomy.
- Bilateral tubal Occlusion
- Barrier methods of contraception (condom or occlusive cap with spermicide).
- Male sterilisation, if the vasectomised partner is the sole partner for the subject.
- True abstinence, when this is in line with the preferred and usual lifestyle of the subject. Periodic abstinence (e.g., calendar, ovulation, symptothermal, post-ovulation methods), declaration of abstinence for the duration of exposure to IMP, and withdrawal are not acceptable methods of contraception

6.3.5 Prevention of 'Over Volunteering'

Volunteers will be excluded from the study if they are concurrently involved in another trial where an IMP has been administered within 30 days prior to enrolment, or will be administered during the trial period. In order to ensure this, volunteers will be asked to provide their National Insurance or Passport number (if they are not entitled to a NI number) and will be registered on a national database of participants in clinical trials (*www.tops.org.uk*). They will not be enrolled if found to be actively registered on another trial until further information on IMP and bleeding schedule is obtained.

6.3.6 Withdrawal of Volunteers

In accordance with the principles of the current revision of the Declaration of Helsinki and any other applicable regulations, a volunteer has the right to withdraw from the study at any time and for any reason, and is not obliged to give his or her reasons for doing so. The Investigator may withdraw the volunteer at any time in the interests of the volunteer's health and well-being. In addition, the volunteer may withdraw/be withdrawn for any of the following reasons:

- Administrative decision by the Investigator.
- Ineligibility (either arising during the study or retrospectively, having been overlooked at screening).
- Significant protocol deviation.
- Volunteer non-compliance with study requirements.
- An AE, which requires discontinuation of the study involvement or results in inability to continue to comply with study procedures.

The reason for withdrawal will be recorded in the CRF. If withdrawal is due to an AE, appropriate follow-up visits or medical care will be arranged, with the agreement of the volunteer, until the AE has resolved, stabilised or a non-trial related causality has been assigned. The DSMB or DSMB chair may recommend withdrawal of volunteers.

Any volunteer who is withdrawn from the study may be replaced, if that is possible within the specified time frame.

If a volunteer withdraws from the study, data and blood samples collected before their withdrawal will still be used on the analysis. Storage of blood samples will continue unless the participant specifically requests otherwise.

In all cases of subject withdrawal, long-term safety data collection, including some procedures such as safety bloods, will continue as appropriate if subjects have received one or more vaccine doses, unless they decline any further follow-up.

6.4 Pregnancy

Should a volunteer become pregnant during the trial, no further study IMP will be administered. She will be followed up for clinical safety assessment with her ongoing consent and in addition will be followed until pregnancy outcome is determined. We would not routinely perform venepuncture in a pregnant volunteer unless there is clinical need.

7 TRIAL PROCEDURES

This section describes the trial procedures for evaluating study participants and follow-up after administration of study vaccine.

7.1 Schedule of Attendance

All volunteers in groups 1 will have the same schedule of clinic attendances and procedures as indicated in the schedules of attendance (Table 6). Group 2 will have clinic attendances and procedures as indicated in the schedules of attendances below (Table 7). Group 3 will have clinic attendances and procedures as indicated in the schedules of attendances below (Table 8). Group 4 will have clinic attendances and procedures as indicated in the schedules of attendances below (Table 9). Subjects will receive either the ChAdOx1 nCoV-19 vaccine or MenACWY control, and undergo follow-up for a total of 12 months from the last vaccination visit. The total volume of blood donated during the study will be 177.5 – 621.5mL depending on which group they are allocated to. Additional visits or procedures may be performed at the discretion of the investigators, e.g., further medical history and physical examination, urine microscopy in the event of positive urinalysis or additional blood tests if clinically relevant.

7.2 Observations

Pulse, blood pressure and temperature will be measured at the time-points indicated in the schedule of procedures and may also be measured as part of a physical examination if indicated at other time-points.

7.3 Blood tests, Nose/Throat Swab and urinalysis

Blood will be drawn for the following laboratory tests and processed at agreed NHS Trust laboratories using NHS standard procedures:

- **Haematology;** Full Blood Count
- **Biochemistry;** Sodium, Potassium, Urea, Creatinine, Albumin, Liver Function Tests (ALT, ALP, Bilirubin)
- **Diagnostic serology;** HBsAg, HCV antibodies, HIV antibodies (specific consent will be gained prior to testing blood for these blood-borne viruses)
- **Immunology;** Human Leukocyte Antigen (HLA) typing (groups 1 and 3 only)

A nose/throat swab will be conducted for COVID-19 NAAT

- **COVID-19** NAAT processing (nose/throat swabs)

Additional safety blood tests may be performed if clinically relevant at the discretion of the medically qualified investigators, including potential prognostic indicators or markers of severe COVID-19 disease.

At University of Oxford research laboratories:

- **Immunology**; Immunogenicity will be assessed by a variety of immunological assays. This may include antibodies to SARS-CoV-Spike and non-Spike antigens by ELISA, ex vivo ELISpot assays for interferon gamma and flow cytometry assays, neutralising and other functional antibody assays and B cell analyses. Other exploratory immunological assays including cytokine analysis and other antibody assays, DNA analysis of genetic polymorphisms potentially relevant to vaccine immunogenicity and gene expression studies amongst others may be performed at the discretion of the Investigators. SARS-CoV-2 serology to be conducted at screening on participants at high risk of COVID-19 exposure (healthcare workers will be prioritised), subject to test availability and lab capacity.
- **Urinalysis;** Urine will be tested for protein, blood and glucose at screening. For female volunteers only, urine will be tested for beta-human chorionic gonadotrophin (β-HCG) at screening and immediately prior to vaccination.
- **Stool samples**; SARS-CoV-2 NAAT, infectivity assays, calprotectin, and other exploratory immunology and microbiology assays may be conducted in a subset of participants, subject to site capacity, sample and test availability

Collaboration with other specialist laboratories in the UK, Europe and outside of Europe for further exploratory tests may occur. This would involve the transfer of serum, urine or plasma, PBMC and/or other study samples to these laboratories, but these would remain anonymised. Informed consent for this will be gained from volunteers. Samples collected for the purposes of COVID-19 diagnosis might be sent to reference labs in the UK alongside their personal data. This would be in line with the national guidance and policy for submitting samples for testing at reference labs.

Immunological assays will be conducted according to local SOPs.

Subjects will be informed that there may be leftover samples of their blood (after all testing for this study is completed), and that such samples may be stored indefinitely for possible future research (exploratory immunology), including genotypic testing of genetic polymorphisms potentially relevant to vaccine immunogenicity. Subjects will be able to decide if they will permit such future use of any leftover samples. With the volunteers' informed consent, any leftover cells, urine and serum/plasma will be frozen indefinitely for future analysis of COVID-19 and other coronaviruses related diseases or vaccine-related responses. If a subject elects not to permit this, all of that subject's leftover samples will be discarded after the required period of storage to meet Good Clinical Practice (GCP) and regulatory requirements.

Samples that are to be stored for future research will be transferred to the OVC Biobank (REC 16/SC/0141).

7.4 Study visits

The study visits and procedures will be undertaken by one of the clinical trials team. The procedures to be included in each visit are documented in the schedule of attendances (Tables6-9). Each visit is assigned a time-point and a window period, within which the visit will be conducted.

7.4.1 Screening visit

Participants will be required to complete an online questionnaire as an initial confirmation of eligibility. All potential volunteers will have a screening visit, which may take place up to 90 days prior to vaccination. At the screening visit, a video presentation of the aims of the study and all tests to be carried out may be screened to an audience. Individually each volunteer will have the opportunity to question an appropriately trained and delegated researcher before signing the consent. Informed consent will be taken before screening, as described in section 6.2. If consent is obtained, the procedures indicated in the schedule of attendances will be undertaken including a medical history, physical examination, blood tests and height and weight. To avoid unnecessary additional venepuncture, if the appropriate blood test results for screening are available for the same volunteer from a screening visit for another study, these results may be used for assessing eligibility (provided the results date is within the 6 months preceding enrolment in COV001).

We will aim to contact the subject's general practitioner with the written permission of the subject after screening to corroborate medical history when possible and practical to do so. GPs will be notified that the subject has volunteered for the study. During the screening, the volunteers will be asked to provide their National Insurance or passport number so that this can be entered on to a national database which helps prevent volunteers from participating in more than one clinical trial simultaneously or over-volunteering for clinical trials (www.tops.org.uk).

Abnormal clinical findings from the urinalysis or blood tests at screening will be assessed by a medically qualified study member. Abnormal blood tests following screening will be assessed according to specific laboratory adverse event grading tables. Any abnormal test result deemed clinically significant may be repeated to ensure it is not a single occurrence. If an abnormal finding is deemed to be clinically significant, the volunteer will be informed and appropriate medical care arranged with the permission of the volunteer.

The eligibility of the volunteer will be reviewed at the end of the screening visit and again when all results from the screening visit have been considered. Decisions to exclude the volunteer from enrolling in the trial or to withdraw a volunteer from the trial will be at the discretion of the Investigator. If eligible, a day 0 visit will be scheduled for the volunteer to receive the vaccine and subsequent follow-up.

7.4.2 Day 0: Enrolment and vaccination visit

Volunteers will be considered enrolled in to the trial at the point of vaccination. Before vaccination/trial intervention, the eligibility of the volunteer will be reviewed. Pulse, blood pressure and temperature will be observed and if necessary, a medical history and physical examination may be undertaken to determine need to withdraw the participant. Participants with symptoms meeting the case definition for COVID-19 or likely recent exposure to COVID-19 will be excluded. Vaccinations will be administered as described below.

7.4.2.1 Vaccination

All vaccines will be administered intramuscularly according to specific SOPs. The injection site will be covered with a sterile dressing and the volunteer will stay in the trial site for observation, in case of immediate adverse events. Observations will be taken 60 minutes after vaccination (+/- 30 minutes) for the prime dose and from 15 minutes after booster vaccination. The sterile dressing will be removed and injection site inspected.

In all groups, volunteers will be given an oral thermometer, tape measure and diary card (paper or electronic), with instructions on use, along with the emergency 24 hour telephone number to contact the on-call study physician if needed. Volunteers will be instructed on how to self-assess the severity of these AEs. There will also be space on the diary card to self-document unsolicited AEs, and whether medication was taken to relieve the symptoms. Volunteers in groups 2f, 2g, 4c and 4d will not be asked to fill-out diary cards for their booster vaccines. Diary cards will collect information on the timing and severity of the following solicited AEs:

Table 4. ***Solicited AEs as collected on post vaccination diary cards***

Local solicited AEs	Systemic solicited AEs
Pain	Fever
Tenderness	Feverishness
Redness	Chills
Warmth	Joint pains
Itch	Muscle pains
Swelling	Fatigue
Induration	Headache
	Malaise
	Nausea
	Vomiting

7.4.2.2 Sequence of Enrolment and Vaccination of Volunteers

Prior to initiation of the study, any newly available safety data will be reviewed from animal studies or clinical trials of coronavirus vaccines being tested elsewhere, and discussed with the DSMB and/or MHRA as necessary. For safety reasons, the first volunteer to receive the IMP will be vaccinated ahead of any other

participants and the profile of adverse events will be reviewed after 24 hours (+24h) post vaccination. Provided there are no safety concerns, as assessed by a medically qualified investigator and/or chair of DSMB, another 3 volunteers will be vaccinated with the IMP after at least 48 hours (±24h) has elapsed following first vaccination and at least 1 hour apart from each other. The profile of AEs will be assessed by a medically qualified investigator in real time and after 24 hours (+24h) of the first 4 participants receiving the IMP, further vaccinations will proceed provided there are no safety concerns. Relevant investigators and chair of DSMB will be asked to provide a decision on whether further vaccinations can go ahead after the first 4 participants received the IMP. A full DSMB may also be consulted should safety concerns arise at this point.

A review will be conducted based on accumulated safety data of the first up to 54 participants receiving the IMP. Enrolment of the remaining participants will only proceed if the CI, and/or other designated relevant investigators and the chair of DSMB assess the data as indicating that it is safe to do so. At this point, any new immunopathology data from pre-clinical challenge studies in ferrets and non-human primates will be assessed by the CI and/or other designated relevant investigators and the DSMB prior to enrolment of the remaining participants.

A second review will be conducted based on accumulated safety data once the trial is fully recruited. The table below provides an estimate of the sequence of recruitment

Table 5. Expected recruitment schedule

By Day	0	3	5	6 onwards
Single Dose IMP arms (up to)	1	3	40	496
Control arms (up to)	1	3	40	496
Prime-Boost Group (up to)			10	
Total per Day (up to)	2	6	90	Approximately 120 per day until trial fully recruited
Cumulative IMP	1	4	54	Up to 540
Cumulative Total	2	8	98	Up to 1090
Safety Review	Real time<br>Review of pre-clinical data	Real time	Real time<br>Review of first 54 participants receiving IMP before enrolling the remainder	Review of Immunopathology data (pre-clinical studies)<br>Review of accumulated safety data once trial fully recruited

7.4.3 Subsequent visits:

Follow-up visits will take place as per the schedule of attendances described in tables 6-9 with their respective windows. Volunteers will be assessed for local and systemic adverse events, interim history, physical examination, review of diary cards (paper or electronic) and blood tests at these time points as detailed in the schedule of attendances. Blood will also be taken for immunology purposes.

If volunteers experience adverse events (laboratory or clinical), which the investigator (physician), CI and/or DSMB chair determine necessary for further close observation, the volunteer may be admitted to an NHS hospital for observation and further medical management under the care of the Consultant on call.

7.4.4 Participants under quarantine

Given the evolving epidemiological situation both globally and in the UK, should a participant be under quarantine and unable to attend any of the scheduled visits, a telephone/video consultation will be arranged using smartphone or computer app if clinically appropriate in order to obtain core study data where possible.

Table 6 Schedule of attendances for participants in group 1

Attendance Number	1[S]	2	3	4	5	6	7	8	9	10	COVID-19 Testing	COVID-19 Testing +3-5 days	COVD-19 NAAT positive + 7 days	COVID-19 Follow-up
Timeline** (days)	≤ 90	0	1	3	7	14	28	56	182	364	As required	3-5 days post symptom onset	7 days post NAAT positive	As required
Time window (days)				±1	±2	±3	±7	±7	±14	±30	N/A		±2	N/A
Informed Consent	X													
Review contraindications, inclusion and exclusion criteria	X	X												
Vaccination		X												
Vital signs^	X	X		X	X	X	X	X	X	X	X	(X)	X	
Telephone/Video call			X											As required
Ascertainment of adverse events		X	X	X	X	X	X	X	X	X	X	(X)	X	X
Diary cards provided		X												X

Attendance Number	1[S]	2	3	4	5	6	7	8	9	10	COVID-19 Testing	COVID-19 Testing +3-5 days	COVD-19 NAAT positive + 7 days	COVID-19 Follow-up
Diary cards collected							X							X
Weekly household exposure questionnaire		ongoing												
Medical History, Physical Examination	X	(X)		(X)	(X)	(X)	(X)	(X)	(X)	(X)	(X)	(X)	(X)	
Biochemistry, Haematology (mL)	5	5		5	5		5				5	(X)	5	
Exploratory immunology (mL)	(5)*	50			50	50	50	50	50	50	up to 50		up to 50	
PAXgenes (mL)		2.5									2.5		2.5	
Nose/Throat Swab											X	(X)	(X)	
Stool sample [a,b]													(X)	(X)
Urinalysis	X													
Urinary bHCG (women only)	X	X												
HLA typing (mL)		4												

Attendance Number	1S	2	3	4	5	6	7	8	9	10	COVID-19 Testing	COVID-19 Testing +3-5 days	COVD-19 NAAT positive + 7 days	COVID-19 Follow-up
HBsAg, HCV Ab, HIV serology (mL)	5													
Blood volume per visit	15	61.5		5	55	50	55	50	50	50	up to 57.5	(X)5	up to 57.5	
Cumulative blood volume%*	15	76.5		81.5	136.5	186.5	241.5	291.5	341.5	391.5	449		506.5	

S = screening visit; (X) = if considered necessary ^ = Vital signs includes pulse, blood pressure and temperature; ** Timeline is approximate only. Exact timings of visits relate to the day on enrolment, ie, each visit must occur at indicated number of days after enrolment ± time window. % Cumulative blood volume for volunteers if blood taken as per schedule, and excluding any repeat safety blood test that may be necessary. Blood volumes may vary according to local site equipment and practices. *SARS-CoV-2 serology to be conducted at screening on participants at high risk of COVID-19 exposure (healthcare workers will be prioritised), subject to test availability and lab capacity. An extra 5mls should be added to cumulative blood volumes if extra COVID-19 serology is required at screening. [a] Subject to site capacity, sample and test availability. [b] Optional Stool sample at approximately 7 days after onset of symptoms for those who have a positive SARS-CoV-2 NAAT test result and possibly again at 14 days after symptom onset if necessary.

Table 7 Schedule of attendances for participants in group 2a and 2b

Attendance Number	1^{S}	2	3	4	5	COVID-19 Testing	COVID-19 Testing + 3-5	COVD-19 NAAT positive + 7 days	COVID-19 follow-up
Timeline** (days)	≤ 90	0	28	182	364	As required	3-5 days post symptom onset	7 days post NAAT positive	As required
Time window (days)			±7	±14	±30	N/A		±2	N/A
Informed Consent	X								
Review contraindications, inclusion and exclusion criteria	X	X							
Vaccination		X							
Vital signs^	X	X	X	X	X	X	(X)	X	
Telephone/Video call									As required
Ascertainment of adverse events		X	X	X	X	X	(X)	X	X
Diary cards provided		X							X
Diary cards collected			X						X
Weekly household exposure questionnaire		ongoing							
Medical History, Physical Examination	X	(X)	(X)	(X)	(X)	(X)	(X)	(X)	
Biochemistry, Haematology (mL)	5	5	5			5	(X)	5	
Exploratory immunology (mL)	(5)*	50	50	50	50	up to 50		up to 50	
PAXgenes (mL)		2.5				2.5		2.5	
Nose/Throat Swab						X	(X)	(X)	

Attendance Number	1[S]	2	3	4	5	COVID-19 Testing	COVID-19 Testing + 3-5	COVD-19 NAAT positive + 7 days	COVID-19 follow-up
Stool sample [a,b]								(X)	(X)
Urinalysis	X								
Urinary bHCG (women only)	X	X							
HBsAg, HCV Ab, HIV serology (mL)	5								
Blood volume per visit	15	57.5	55	50	50	up to 57.5	(X) 5	up to 57.5	
Cumulative blood volume[%]	15	72.5	127.5	177.5	227.5	285		342.5	

S = screening visit; (X) = if considered necessary ^ = Vital signs includes pulse, blood pressure and temperature; ** Timeline is approximate only. Exact timings of visits relate to the day on enrolment, ie, each visit must occur at indicated number of days after enrolment ± time window.% Cumulative blood volume for volunteers if blood taken as per schedule, and excluding any repeat safety blood test that may be necessary. Blood volumes may vary according to local site equipment and practices. *SARS-CoV-2 serology to be conducted at screening on participants at high risk of COVID-19 exposure (healthcare workers will be prioritised), subject to test availability and lab capacity. An extra 5mls should be added to cumulative blood volumes if extra COVID-19 serology is required at screening. [a] Subject to site capacity, sample and test availability. [b] Optional Stool sample at approximately 7 days after onset of symptoms for those who have a positive SARS-CoV-2 NAAT test result and possibly again at 14 days after symptom onset if necessary.

Table 8 Schedule of attendances for participants in group 2c, 2d and 2e

Attendance Number	1[S]	2	3	4	5	6	7	8	COVID-19 Testing	COVID-19 Testing +3-5 days	COVD-19 NAAT positive + 7 days	COVID-19 follow-up
Timeline** (days)	≤ 90	0	28	56	14 days post boost	28 days post boost	182 days post boost	364 days post boost	As required	3-5 days post symptom onset	7 days post NAAT positive	As required
Time window (days)			±7	-7/+14	±7	±7	±14	±30	N/A		±2	N/A
Informed Consent	X											
Review contraindications, inclusion and exclusion criteria	X	X										
Vaccination		X		X								
Vital signs^	X	X	X				X	X	X	(X)	X	
Telephone/Video call												As required
Ascertainment of adverse events		X	X				X	X	X	(X)	X	X
Diary cards provided		X		X								X
Diary cards collected			X			X						X
Weekly household exposure questionnaire		ongoing										

Attendance Number	1[S]	2	3	4	5	6	7	8	COVID-19 Testing	COVID-19 Testing +3-5 days	COVD-19 NAAT positive + 7 days	COVID-19 follow-up
Medical History, Physical Examination	X	(X)	(X)				(X)	(X)	(X)	(X)	(X)	
Biochemistry, Haematology (mL)	5	5	5	5	5	5			5	(X)	5	
Exploratory immunology (mL)	(5)*	50	50	Up to 50	Up to 50	Up to 50	Up to 50	Up to 50	up to 50		up to 50	
PAXgenes (mL)		2.5							2.5		2.5	
Nose/Throat Swab									X	(X)	(X)	
Stool sample [a,b]											(X)	(X)
Urinalysis	X											
Urinary bHCG (women only)	X	X		X								
HBsAg, HCV Ab, HIV serology (mL)	5											
Blood volume per visit	15	57.5	55	55	55	55	50	50	up to 57.5	(X) 5	up to 57.5	
Cumulative blood volume[%]	15	72.5	127.5	182.5	237.5	292.5	342.5	392.5	450		507.5	

S = screening visit; (X) = if considered necessary ^ = Vital signs includes pulse, blood pressure and temperature; ** Timeline is approximate only. Exact timings of visits relate to the day on enrolment, ie, each visit must occur at indicated number of days after enrolment ± time window.% Cumulative blood volume for volunteers if blood taken as per schedule, and excluding any repeat safety blood test that may be necessary. Blood volumes may vary according to local site equipment and practices. *SARS-CoV-2 serology to be conducted at screening on participants at high risk of COVID-19 exposure (healthcare workers will be prioritised), subject to test availability and lab capacity. An extra 5mls should be added to cumulative blood volumes if extra COVID-19 serology is required at

screening. [a] Subject to site capacity, sample and test availability. [b] Optional Stool sample at approximately 7 days after onset of symptoms for those who have a positive SARS-CoV-2 NAAT test result and possibly again at 14 days after symptom onset if necessary.

Table 9 Schedule of attendances for participants in group 3

Attendance Number	1[S]	2(V1)	3	4	5	6	7 (V2)	8	9	10	11	12	13	COVID-19 Testing	COVID-19 Testing +3-5 days	COVD-19 NAAT positive + 7 days	COVID-19 Follow-up
Timeline** (days)	≤ 90	0	1	3	7	14	28	31	35	42	56	182	364	As required	3-5 days post symptoms onset	7 days post NAAT positive	As required
Time window (days)			+1	±1	±3	±3	±7	±1	±2	±3	±3	±14	±30	N/A		±2	N/A
Informed Consent	X																
Review contraindications, inclusion and exclusion criteria	X	X					X										
Vaccination		X					X										
Vital signs^	X	X		X	X	X	X	X	X	X	X	X	X	X	(X)	X	
Telephone/Video call			X														As required
Ascertainment of adverse events		X	X	X	X	X	X	X	X	X	X	X	X	X	(x)	X	X
Diary cards provided		X					X										X
Diary cards collected							X				X						X
Weekly household exposure questionnaire		ongoing															

Attendance Number	1[S]	2(V1)	3	4	5	6	7 (V2)	8	9	10	11	12	13	COVID-19 Testing	COVID-19 Testing +3-5 days	COVD-19 NAAT positive + 7 days	COVID-19 Follow-up
Medical History, Physical Examination	X	(X)		(X)	(X)	(X)	(X)	(X)	(X)	(X)	(X)	(X)	(X)	(X)	(X)	(X)	
Biochemistry[$], Haematology (mL)	5	5		5	5		5	5	5		5			5	(X)	5	
Exploratory immunology[£] (mL)	(5)*	50			50	50	50		50	50	50	50	50	up to 50		up to 50	
PAXgenes (mL)		2.5												2.5		2.5	
Nasal/Throat Swab														X	(X)	(X)	
Stool sample [a,b]																(X)	(X)
Urinalysis	X																
Urinary bHCG (women only)	X	X					X										
HLA typing (mL)		4															
HBsAg, HCV Ab, HIV serology (mL)	5																
Blood volume per visit	15	61.5		5	55	50	55	5	55	50	55	50	50	up to 57.5	(X) 5	up to 57.5	
Cumulative blood volume[%]	15	76.5		81.5	136.5	186.5	241.5	246.5	301.5	351.5	406.5	456.5	506.5	564		621.5	

S = screening visit; (X) = if considered necessary ^ = Vital signs includes pulse, blood pressure and temperature; ** Timeline is approximate only. Exact timings of visits relate to the day on enrolment, ie, each visit must occur at indicated number of days after enrolment ± time window.% Cumulative blood volume for volunteers if blood taken as per schedule, and excluding any repeat safety blood test that may be necessary. Blood volumes may vary according to local site equipment and practices. *SARS-CoV-2 serology to be conducted at screening on participants at high risk of COVID-19 exposure (healthcare workers will be prioritised), subject to test availability and lab capacity. An extra 5mls should be added to cumulative blood volumes if extra COVID-19 serology is required at

screening. [a] Subject to site capacity, sample and test availability. [b] Optional Stool sample at approximately 7 days after onset of symptoms for those who have a positive SARS-CoV-2 NAAT test result and possibly again at 14 days after symptom onset if necessary.

Table 10 schedule of attendances for participants in group 4

Attendance Number	1[S]	2	3	4	5	COVID-19 Testing	COVID-19 Testing +3-5 days	COVD-19 Positive NAAT + 7 days	COVID-19 Follow-up
Timeline** (days)	≤ 90	0	28	182	364	As required	3-5 days post symptom onset	7 days post NAAT postive	As required
Time window (days)			±7	±14	±30	N/A	+2	±2	N/A
Informed Consent	X								
Review contraindications, inclusion and exclusion criteria	X	X							
Vaccination		X							
Prophylactic Paracetamol for 24h[p]		X							
Vital signs^	X	X	X	X	X	X	(X)	X	
Telephone/Video call									As required
Ascertainment of adverse events		X	X	X	X	X	(X)	X	X
Diary cards provided		X							X
Diary cards collected			X						X
Weekly household exposure questionnaire			ongoing						
Medical History, Physical Examination	X	(X)	(X)	(X)	(X)	(X)	(X)	(X)	
Biochemistry, Haematology (mL)	5	5	5			5	(X)	5	
Exploratory immunology (mL)	(5)*	10	10	10	10	up to 50		up to 50	

Attendance Number	1[S]	2	3	4	5	COVID-19 Testing	COVID-19 Testing +3-5 days	COVD-19 Positive NAAT + 7 days	COVID-19 Follow-up
PAXgenes (mL)		2.5				2.5		2.5	
Nose/Throat Swab						X	(X)	X	
Stool sample [a,b]								(X)	(X)
Urinalysis	X								
Urinary bHCG (women only)	X	X							
HBsAg, HCV Ab, HIV serology (mL)	5								
Blood volume per visit	15	17.5	15	10	10	up to 57.5	5	up to 57.5	
Cumulative blood volume[%]	15	32.5	47.5	57.5	67.5	125		182.5	

S = screening visit; (X) = if considered necessary ^ = Vital signs includes pulse, blood pressure and temperature; ** Timeline is approximate only. Exact timings of visits relate to the day on enrolment, ie, each visit must occur at indicated number of days after enrolment ± time window.% Cumulative blood volume for volunteers if blood taken as per schedule, and excluding any repeat safety blood test that may be necessary. Blood volumes may vary according to local site equipment and practices. *SARS-CoV-2 serology to be conducted at screening on participants at high risk of COVID-19 exposure (healthcare workers will be prioritised), subject to test availability and lab capacity. An extra 5mls should be added to cumulative blood volumes if extra COVID-19 serology is required at screening.P = prophylactic paracetamol over the first 24h post immunisation in a subset of participants in group 4 only. [a] Subject to site capacity, sample and test availability. [b] Optional Stool sample at approximately 7 days after onset of symptoms for those who have a positive SARS-CoV-2 NAAT test result and possibly again at 14 days after symptom onset if necessary.

Table 11 Schedule of attendances for participants in group 2f, 2g, 4c and 4d (booster)

Attendance Number (boost)	1 V2 (booster)	2	3	4	8	COVID-19 Testing	COVID-19 Testing +3-5 days	COVD-19 Positive NAAT + 7 days	COVID-19 Follow-up
Timeline** (days)	Minimum 4weeks post prime	28 days post boost	90 days post boost	182 days post boost	364 days post boost	As required	3-5 days post symptom onset	7 days post positive NAAT result	
Time window (days)	+14	±7	±14	±14	±30	N/A	+2	±2	
Informed Consent	X								
Review contraindications, inclusion and exclusion criteria	X								
Vaccination	X								
Prophylactic Paracetamol for 24h[p]	X								
Vital signs^	(X)	(X)	(X)	(X)	(X)	X	(X)	X	
Telephone/Video call									As required
Ascertainment of adverse events	X	X	X	X	X	X	(X)	X	X
Symptoms diary									X
Weekly household exposure questionnaire			ongoing						

Attendance Number (boost)	1<br>V2<br>(booster)	2	3	4	8	COVID-19 Testing	COVID-19 Testing +3-5 days	COVD-19 Positive NAAT + 7 days	COVID-19 Follow-up
Medical History	(X)	(X)	(X)	(X)	(X)	(X)	(X)	(X)	
Physical Examination (if necessary)	(X)	(X)	(X)	(X)	(X)	(X)	(X)	(X)	
Biochemistry, Haematology (mL)						5	(X)	5	
Exploratory immunology (mL)	up to 50	up to 50	up to 50	up to 50	up to 50	up to 50[d]		up to 50[d]	
Nose/Throat Swab and/or saliva sample						X	(X)	(X)	
Stool sample [a,b]								(X)	(X)
Urinary bHCG (women of childbearing potential only)	X								
Blood volume per visit	50	50	50	50	50	up to 55	(X)5	up to 55	
Cumulative blood volume[%]	50	100	150	200	250	305		360	

S = screening visit; (X) = if considered necessary; ** Timeline is approximate only. Exact timings of visits relate to the day on enrolment, ie, each visit must occur at indicated number of days after enrolment ± time window. ^Only temperature will be routinely measured at subsequent follow-up visits. At COVID-19 testing visits a full set observations will be taken, including respiratory rate and oxygen saturation. % Cumulative blood volume for volunteers if blood taken as per schedule, and excluding any repeat safety blood test that may be necessary. Blood volumes may vary according to local site equipment and practices. [a] Subject to site capacity, sample and test availability. [d] Optional and subject to site capacity. [b] Optional Stool sample at approximately 7 days after onset of symptoms for those who have a positive SARS-CoV-2 NAAT test result and possibly again at 14 days after symptom onset if necessary. [P] = prophylactic paracetamol over the first 24h post immunisation

7.4.5 Symptomatic volunteers

Participants who become symptomatic during follow-up will be instructed to call the study team who will then advise on how to proceed with clinical testing for COVID-19 if necessary, as per the trial working instructions. Participants will get weekly reminders (email or text messages) to get in touch with the study team if they present with a new onset of fever or cough or shortness of breath or anosmia/ageusia and if they are admitted to hospital for any reason. At the COVID-19 testing visit, a nose/throat swab, blood samples for safety (FBC, Biochemistry, CRP, others if deemed clinically relevant) and immunology (paxgenes, cytokine profile, PBMCs, serum and others), vital signs and other clinical data will be taken. Symptomatic volunteers may be regularly reviewed over the phone or via video call using a smartphone or computer app if clinically appropriate. Participants will be asked to attend a follow-up visit at 3-5 days post symptoms onset (+2 days) for clinical review and further testing or will be given a kit with instructions for a self-swab instead of a clinic visit. . Participants will be asked to record information on an electronic diary COVID-19 related symptoms for safety monitoring until symptom resolution or for at least 14 days if symptoms do not resolve before then. Participants who have a positive NAAT at S0, will not be required to attend a S3-5 visit (or provide a self-swab), but will be reviewed for safety at 7 days post positive swab. Clinical data, and additional blood samples for safety and immunology purposes will be taken at the S7 visit. Participants who have a positive swab at S3-5 will be reviewed for safety at 7 days post positive swab where clinical data, and additional blood samples for safety and immunology purposes will be taken. Participants who have 2 negative NAAT results from S0 and either a S3-5 visit or a self-swab will not be required to attend for an S7 visit. Closer follow-up and safety monitoring may be carried out by local trial teams if felt this is clinically indicated. If breathlessness is the only symptom that triggers a swab, further testing at S3-5 or S7 will be conducted at clinical discretion if there is no objective signs of respiratory distress (e.g. tachypnea,desaturation).

Participants who develop COVID-19 symptoms and have a positive NAAT test after the first vaccination can only receive a booster dose after a minimum 4 weeks interval from their first NAAT positive test, provided their symptoms have significantly improved. The decision to proceed with booster vaccinations in those cases will be at clinical discretion of the investigators. For participants who are asymptomatic and have a positive NAAT test (e.g. done outside the study), a minimum of 2 weeks from first NAAT positivity will be required before boosting.

7.4.6 Household Weekly Questionnaire (optional)

Participants will be asked to record information on a weekly basis about illnesses amongst household contacts and friends, their contact with the general public, and infection control procedures. This will be optional.

Volunteers will be asked to enter data in a diary from baseline to the end of the follow-up period. This will be recorded via a web-based electronic diary to which participants will be provided access at baseline.

7.4.7 Stool samples (optional)

Those participants who have a SARS-CoV-2 positive NAAT test result, may be asked to provide a stool sample at approximately 7 days after symptom onset and 14 days after the first sample if necessary, as per trial specific instructions. Samples will be processed to look at differences in viral shedding between the investigational vaccine and control arms, and to measure calprotectin levels as a marker of gastrointestinal inflammation. These samples will be collected and processed depending on test availability, laboratory capacity, and will not be compulsory to the volunteers. Further exploratory immunology and microbiology tests may be conducted at the investigators' discretion.

7.4.8 Medical notes review

With the participants consent, the study team will request access to medical notes or submit a data collection form for completion by attending clinical staff on any medically attended COVID-19 episodes. Any data which are relevant to ascertainment of efficacy endpoints and disease enhancement (AESI) will be collected. These are likely to include, but not limited to, information on ICU admissions, clinical parameters such as oxygen saturation, respiratory rates and vital signs, need for oxygen therapy, need for ventilatory support, imaging and blood tests results, amongst others.

7.4.9 Randomisation, blinding and code-breaking

Participants will be randomised to investigational vaccine or MenACWY in a 1:1 allocation, using block randomisation. Block sizes will reflect the numbers to be recruited at each stage of the study. The first block will be a block of 2 participants, followed by a block of 6, then blocks of 4 as required to meet the totals for randomisation for each day.

Participants enrolled in groups 1, 2 and 4 will be blinded to the arm they have been allocated to, whether investigational vaccine or control. The trial staff administering the vaccine will not be blinded. Vaccines will be prepared out of sight of the participant and syringes will be covered with an opaque object/material until ready for administration to ensure blinding.

Additional steps may be taken to keep clinical investigators assessing primary endpoints blinded to group allocation, where this is possible and practical to do so. A designated member of the clinical team may be unblinded for the purposes of safety reporting procedures.

If the clinical condition of a participant necessitates breaking the code, this will be undertaken according to a trial specific working instruction and group allocation sent to the attending physician, if unblinding is thought to be relevant and likely to change clinical management.

Participants enrolled in group 3 will not be randomised or blinded

8 INVESTIGATIONAL PRODUCT AND TRIAL INTERVENTIONS

8.1 Manufacturing and presentation

8.1.1 Description of ChAdOx1 nCoV-19

ChAdOx1 nCoV-19 vaccine consists of the replication-deficient simian adenovirus vector ChAdOx1, containing the structural surface glycoprotein (Spike protein) antigens of SARS-CoV-2.

8.2 Supply

ChAdOx1 nCoV-19 has been formulated and vialed at the Clinical BioManufacturing Facility (CBF), University of Oxford or Advent Srl, Italy. At the CBF the vaccine will be certified and labelled for the trial by a Qualified Person (QP) before transfer to the clinical site.

ChAdOx1 nCoV-19 (AZD1222) has been formulated at Cobra Biologics Ltd, vialed at Symbiosis Pharmaceutical Services, and labelled and packaged at Thermo Fisher Scientific (Hertfordshire, United Kingdom). It will be certified by a Qualified Person (QP) at the MedImmune Pharma, BV (Nijmegen, The Netherlands) or MedImmune Ltd (Cambridge, United Kingdom) before release and transfer to the clinical site.

8.3 Storage

The vaccine manufactured at CBF or Advent Srl is stored at nominal -80°C in a secure freezer, at the clinical site. The vaccine manufactured by Cobra Biologics Ltd is stored at 2-8°C in a secure fridge, at the clinical site. All movements of the study vaccines will be documented in accordance with existing standard operating procedure (SOP). Vaccine accountability, storage, shipment and handling will be in accordance with relevant SOPs and forms. To allow for large number of participants to receive the vaccine in a short time period additional clinic locations may be used. In this instance vaccines will be transported in accordance with local SOP's and approvals as required.

8.4 Administration

For Advent manufactured vaccine: On vaccination day, ChAdOx1 nCoV-19 will be allowed to thaw to room temperature and will be administered within 1 hour of removal from storage.

For Cobra manufactured vaccine: On vaccination day, the multi-dose vial will be removed from 2-8 storage as required. If the vaccine is stored outside of 2-8 it must be used within 6 hours.

The vaccine will be administered intramuscularly into the deltoid of the non-dominant arm (preferably). All volunteers will be observed in the unit for a minimum of 1 hour (±30 minutes) after vaccination for the prime dose and a minimum of 15 minutes after the booster vaccination. During administration of the investigational products, Advanced Life Support drugs and resuscitation equipment will be immediately available for the management of anaphylaxis. Vaccination will be performed and the IMPs handled according to the relevant SOPs.

8.5 Rationale for selected dose

The dose to be administered in this trial have been selected on the basis of clinical experience with the ChAdOx1 adenovirus vector expressing different inserts and other similar adenovirus vectored vaccines (eg. ChAd63).

A first-in-man dose escalation study using the ChAdOx1 vector encoding an influenza antigen (FLU004), safely administered ChAdOx1 NP+M1 at doses ranging from 5 x 10^8 to 5 x 10^{10} vp. Subsequent review of the data identified an optimal dose of 2.5 x 10^{10} vp balancing immunogenicity and reactogenicity. This dose has subsequently been given to over hundreds of volunteers in numerous larger phase 1 studies at the Jenner Institute. ChAdOx1 vectored vaccines have thus far demonstrated to be very well tolerated. The vast majority of AEs have been mild-moderate and there have been no SARs until this date.

Another simian adenovirus vector (ChAd63) has been safely administered at doses up to 2 x 10^{11} vp with an optimal dose of 5 x 10^{10} vp, balancing immunogenicity and reactogenicity.

MERS001 was the first clinical trial of a ChAdOx1 vectored expressing the full-length Spike protein from a separate, but related betacoronavirus. ChAdOx1 MERS has been given to 31 participants to date at doses ranging from 5x10^9 vp to 5x10^{10} vp. Despite higher reactogeniticy observed at the 5x10^{10} vp, this dose was safe, with self-limiting AEs and no SARs recorded. The 5x10^{10} vp was the most immunogenic, in terms of inducing neutralising antibodies against MERS-CoV using a live virus assay (Folegatti et al. Lancet Infect Dis, 2020, in press). Given the immunology findings and safety profile observed with a ChAdOx1 vectored vaccine against MERS-CoV, the 5x10^{10} vp dose was chosen for ChAdOx1 nCoV-19.

As this is a first-in-human assessment of the SARS-CoV-2 S antigenic insert, a staggered enrolment will apply for the first volunteers enrolled in the study. The same procedure will apply, should other batches of ChAdOx1 nCoV-19 become available. Safety of ChAdOx1 nCoV-19 will be monitored in real time. and should unacceptable adverse events or safety concerns arise, doses will be decreased via an amendment.

An analytical comparability assessment of ChAdOx1 nCoV-19 (AZD1222) manufactured by CBF, Advent and Cobra Biologics was conducted using a comprehensive set of physiochemical and biological release and

characterization tests. In order to support the analytical comparability assessment, A260 testing of Advent's process (K.0007, K.0008, and K.0009 lots) was performed, where corrections to the absorbance due to excess polysorbate 80 were made to compensate for polysorbate 80 concentrations above the formulation target of 0.1% (w/v).

Differences in strength related attributes (ie, virus particle concentration, virus genome concentration, and infectious virus concentration) are noted. These differences in strength is further examined for potential impact on clinical dosing. The target clinical dosage of CBF's product is 5×10^{10} viral particles per dose based on vp/mL concentration determined by UV spectroscopy (A260), whereas that of Advent's product is 5×10^{10} viral genome copies per dose based on vg/mL concentration determined by qPCR. The target clinical dosage of Symbiosis' product is $3.5 – 6.5 \times 10^{10}$ viral particles per dose based on the vp/mL concentration determined by A260, with a 0.5 mL dosing volume. This dosing range is based on a target 5×10^{10} viral particles per dose and a ± 30% range to take into account process and method variabilities. When the planned clinical dosage of Symbiosis' product is compared to that of CBF and Advent products, the resulting Symbiosis' product dosage at 0.5 mL for lot 20481A is somewhat lower in total viral particle per dose (20% from the lower range limit), slightly higher in total viral genome copies per dose (12% from the higher range limit), and slightly lower in total infectious particle per dose (8% from the lower range limit). These differences are considered to be comparable to or within the variabilities from the analytical methods used in concentration determination (A260, qPCR, and infectivity) and the dosing volumes during clinical administration. In summary, with a 0.5 mL dosing volume for Symbiosis' product, the strength difference from CBF and Advent products is not expected to have significant clinical impact in terms of reactogenicity and immunogenicity/efficacy.

Table 12 Clinical Strengths of ChAdOx1 nCoV-19 (AZD1222) Drug Product

Strength Attribute	CBF		Advent			Cobra
	Lot 02P20-01	Lot 02P20-02	Lot K.0007	Lot K.0008	Lot K.0009	Lot 20481A
**Concentration**						
Virus particle concentration (A_{260}) (vp/mL)	1.49×10^{11}	1.22×10^{11}	3.12×10^{11}	3.16×10^{11}	2.45×10^{11}	0.8×10^{11}
Virus genome concentration (qPCR) (vg/mL)	1.7×10^{11}	Not tested	1.7×10^{11}	2.1×10^{11}	1.4×10^{11}	1.3×10^{11}
Infectious particle concentration (ifu/mL) [a]	2.6×10^{9}	Not tested	2.9×10^{9}	3.0×10^{9}	2.4×10^{9}	1.3×10^{9}
**Target Clinical Dosage**						
Equivalent DP volume per dose (mL)	0.34	0.41	0.294	0.235	0.356	0.50
Dosing of virus particle (vp/dose)	5.1×10^{10}	5.0×10^{10}	9.2×10^{10}	7.4×10^{10}	8.7×10^{10}	4.0×10^{10}
Dosing of viral genome (vg/dose)	5.8×10^{10}	NA	5.0×10^{10}	4.9×10^{10}	5.0×10^{10}	6.5×10^{10}
Dosing of infectious particle (ifu/dose)	8.8×10^{8}	NA	8.5×10^{8}	7.1×10^{8}	8.5×10^{8}	6.5×10^{8}

ifu = infectious units; NA = not applicable; vp = virus particle; vg = virus genome

[a] Testing performed using the Advent infectivity assay.

8.6 Minimising environmental contamination with genetically modified organisms (GMO)

The study will be performed in accordance with the current version of the UK Genetically Modified Organisms (Contained Use) Regulations. Approved SOPs will be followed to minimise dissemination of the recombinant vectored vaccine virus into the environment. GMO waste will be inactivated according to approved SOPs.

8.7 Control Vaccine

Participants who are allocated to the control groups will receive one or two injections of MenACWY vaccine instead of ChAdOx1 nCoV-19. Either of the two licensed quadrivalent protein-polysaccharide conjugate vaccine MenACWY vaccines will be used, i.e.:

- Nimenrix (Pfizer). The licensed posology of this vaccine for those over 6 months of age is a single (0.5ml) intramuscular dose, containing 5mcg each of *Neisseria meningitidis* group A, C, W and Y polysaccharide, each conjugated to 44 mcg tetanus toxoid carrier protein.
- Menveo (Glaxosmithkline). The licensed posology of this vaccine for those 2 years of age and over is a single (0.5ml) intramuscular dose, containing
    - 10 mcg meningococcal group A polysaccharide, conjugated to 16.7 to 33.3 mcg *Corynebacterium diphtheriae* CRM_{197} protein
    - 5mcg meningococcal group C polysaccharide, conjugated to 7.1 to 12.5 mcg *C. diphtheriae* CRM_{197} protein
    - 5mcg meningococcal group W polysaccharide, conjugated to 3.3 to 8.3 mcg *C. diphtheriae* CRM_{197} protein
    - 5mcg meningococcal group Y polysaccharide, conjugated to 5.6 to 10.0 mcg *C. diphtheriae* CRM_{197} protein

The summary of product characteristics for both vaccines allows for administration of a booster dose if indicated by ongoing risk, therefore allows for the two doses administered to a subset of participants in this study. Similarly, previous receipt of either vaccine (or a plain polysaccharide quadrivalent meningococccal A, C, W and Y vaccine) will not be a contraindication to receiving a further vaccine in this study.

Participants will be blinded as to which intervention they are receiving. A vaccine accountability log of MenACWY will be maintained at each trial site. There will be no additional labelling of these vaccines beyond their licensed packaging.

MenACWY will be stored in a locked (or access controlled) refrigerator (2°C – 8°C) at the sites, as per SmPC.

8.8 Compliance with Trial Treatment

All vaccinations will be administered by the research team and recorded in the CRF. The study medication will be at no time in the possession of the participant and compliance will not, therefore, be an issue.

8.9 Accountability of the Trial Treatment

Accountability of the IMP and control vaccine will be conducted in accordance with the relevant SOPs.

8.10 Paracetamol (non-IMP)

Paracetamol will be provided to a subset of participants in group 4 to be taken at vaccination day for 24hours.

Participants in groups 2f, 2g, 4c, and 4d will be advised to take prophylactic paracetamol for 24h post booster vaccine.

8.11 Concomitant Medication

As set out by the exclusion criteria, volunteers may not enter the study if they have received: any vaccine in the 30 days prior to enrolment or there is planned receipt of any other vaccine within 30 days of each vaccination, any investigational product within 30 days prior to enrolment or if receipt is planned during the study period, or if there is any use of immunosuppressant medication within 6 months prior to enrolment or if receipt is planned at any time during the study period (except topical steroids and short course of low dose steroids < 14 day).

8.12 Provision of Treatment for Controls

If this vaccine is proven to be efficacious following analysis of the primary endpoint and if the DSMB agrees, participants allocated to MenACWY control group may be offered the IMP, should extra doses become available.

9 ASSESSMENT OF SAFETY

Safety will be assessed by the frequency, incidence and nature of AEs and SAEs arising during the study.

9.1 Definitions

9.1.1 Adverse Event (AE)

An AE is any untoward medical occurrence in a volunteer, which may occur during or after administration of an IMP and does not necessarily have a causal relationship with the intervention. An AE can therefore be any unfavourable and unintended sign (including any clinically significant abnormal laboratory finding or change from baseline), symptom or disease temporally associated with the study intervention, whether or not considered related to the study intervention.

9.1.2 Adverse Reaction (AR)

An AR is any untoward or unintended response to an IMP. This means that a causal relationship between the IMP and an AE is at least a reasonable possibility, i.e., the relationship cannot be ruled out. All cases judged by the reporting medical Investigator as having a reasonable suspected causal relationship to an IMP (i.e. possibly, probably or definitely related to an IMP) will qualify as AR.

Adverse events that may be related to the IMP are listed in the Investigator's Brochure for each product.

9.1.3 Serious Adverse Event (SAE)

An SAE is an AE that results in any of the following outcomes, whether or not considered related to the study intervention.

- Death
- Life-threatening event (i.e., the volunteer was, in the view of the Investigator, at immediate risk of death from the event that occurred). This does not include an AE that, if it occurred in a more severe form, might have caused death.
- Persistent or significant disability or incapacity (i.e., substantial disruption of one's ability to carry out normal life functions).
- Hospitalisation or prolongation of existing hospitalisation, regardless of length of stay, even if it is a precautionary measure for continued observation. Hospitalisation (including inpatient or outpatient hospitalisation for an elective procedure) for a pre-existing condition that has not worsened unexpectedly does not constitute a serious AE.
- An important medical event (that may not cause death, be life threatening, or require hospitalisation) that may, based upon appropriate medical judgment, jeopardise the volunteer and/or require medical or surgical intervention to prevent one of the outcomes listed above. Examples of such medical events

include allergic reaction requiring intensive treatment in an emergency room or clinic, blood dyscrasias, or convulsions that do not result in inpatient hospitalisation.

- Congenital anomaly or birth defect.

9.1.4 Serious Adverse Reaction (SAR)

An AE that is both serious and, in the opinion of the reporting Investigator or Sponsors, believed to be possibly, probably or definitely due to an IMP or any other study treatments, based on the information provided.

9.1.5 Suspected Unexpected Serious Adverse Reaction (SUSAR)

A SAR, the nature and severity of which is not consistent with the information about the medicinal product in question set out in the IB.

9.2 Expectedness

No IMP related SAEs are expected in this study. All SARs will therefore be reported as SUSARs.

9.3 Foreseeable Adverse Reactions:

The foreseeable ARs following vaccination with ChAdOx1 nCoV-19 include injection site pain, tenderness, erythema, warmth, swelling, induration, pruritus, myalgia, arthralgia, headache, fatigue, fever, feverishness, chills, malaise and nausea.

9.4 Adverse Events of Special Interest (AESI)

Disease enhancement following vaccination with ChAdOx1 nCoV-19 will be monitored. Severe COVID-19 disease will be defined using clinical criteria. Detailed clinical parameters will be collected from medical records and aligned with agreed definitions as they emerge. These are likely to include, but are not limited to, oxygen saturation, need for oxygen therapy, respiratory rate, need for ventilatory support, imaging and blood test results, amongst other clinically relevant parameters. Acute respiratory distress, pneumonitis, acute cardiac injury, arrhythmia, septic-shock like syndrome and acute kidney injury related with COVID-19 disease will be monitored from medical records review of hospitalised participants.

Eosinophilia as a marker skewed Th2 responses will be routinely monitored in participants attending their COVID-19 testing and follow-up visits. Marked eosinophilia of $\geq 1.5 \times 10^9$/L will be reported as SAEs.

AESI relevant to vaccination in general will also be monitored such as: generalised convulsion, Guillain-Barre Syndrome (GBS), Acute Disseminated Encephalomyelitis (ADEM), Thrombocytopenia, Anaphylaxis, Vasculitides in addition to serious solicited AEs will be monitored.

9.5 Causality

For every AE, an assessment of the relationship of the event to the administration of the vaccine will be undertaken by the CI-delegated clinician. An interpretation of the causal relationship of the intervention to the AE in question will be made, based on the type of event; the relationship of the event to the time of vaccine administration; and the known biology of the vaccine therapy (Table 11). Alternative causes of the AE, such as the natural history of pre-existing medical conditions, concomitant therapy, other risk factors and the temporal relationship of the event to vaccination will be considered and investigated. Causality assessment will take place during planned safety reviews, interim analyses (e.g. if a holding or stopping rule is activated) and at the final safety analysis, except for SAEs, which should be assigned by the reporting investigator, immediately, as described in SOP OVC005 Safety Reporting for CTIMPs.

0	**No Relationship**	No temporal relationship to study product ***and***<br>Alternate aetiology (clinical state, environmental or other interventions); ***and***<br>Does not follow known pattern of response to study product
1	**Unlikely**	Unlikely temporal relationship to study product ***and***<br>Alternate aetiology likely (clinical state, environmental or other interventions) ***and***<br>Does not follow known typical or plausible pattern of response to study product.
2	**Possible**	Reasonable temporal relationship to study product; ***or***<br>Event not readily produced by clinical state, environmental or other interventions; ***or***<br>Similar pattern of response to that seen with other vaccines
3	**Probable**	Reasonable temporal relationship to study product; ***and***<br>Event not readily produced by clinical state, environment, or other interventions ***or***<br>Known pattern of response seen with other vaccines
4	**Definite**	Reasonable temporal relationship to study product; ***and***<br>Event not readily produced by clinical state, environment, or other interventions; ***and***<br>Known pattern of response seen with other vaccines

Table 11. *Guidelines for assessing the relationship of vaccine administration to an AE.*

9.6 Reporting Procedures for All Adverse Events

All local and systemic AEs occurring in the 28 days following each vaccination observed by the Investigator or reported by the volunteer, whether or not attributed to study medication, will be recorded in electronic diaries or study database. All AEs that result in a volunteer's withdrawal from the study will be followed up until a satisfactory resolution occurs, or until a non-study related causality is assigned (if the volunteer consents to this). SAEs and Adverse Events of Special Interest will be collected throughout the entire trial period.

9.7 Assessment of severity

The severity of clinical and laboratory adverse events will be assessed according to scales based on FDA toxicity grading scales for healthy and adolescent volunteers enrolled in preventive vaccine clinical trials, listed in the study specific working instructions and tables 11-13 below,

Adverse Event	Grade	Intensity
Pain at injection site	1	Pain that is easily tolerated
	2	Pain that interferes with daily activity
	3	Pain that prevents daily activity
	4	A&E visit or hospitalization
Tenderness	1	Mild discomfort to touch
	2	Discomfort with movement
	3	Significant discomfort at rest
	4	A&E visit or hospitalization
Erythema at injection site*	1	2.5 - 5 cm
	2	5.1 - 10 cm
	3	>10 cm
	4	Necrosis or exfoliative dermatitis
Induration/Swelling at injection site	1	2.5 – 5 cm and does not interfere with activity
	2	5.1 - 10 cm or interferes with activity
	3	>10 cm or prevents daily activity
	4	Necrosis

Table 12. Severity grading criteria for local adverse events *erythema ≤2.5cm is an expected consequence of skin puncture and will therefore not be considered an adverse event

Vital Signs	Grade 1 (mild)	Grade 2 (moderate)	Grade 3 (severe)	Grade 4 Potentially Life threatening
Fever (oral)	38.0°C - 38.4°C	38.5°C – 38.9°C	39.0°C - 40°C	> 40°C
Tachycardia (bpm)*	101 - 115	116 – 130	>130	A&E visit or hospitalisation for arrhythmia
Bradycardia (bpm)**	50 – 54	45 – 49	<45	A&E visit or hospitalisation for arrhythmia
Systolic hypertension (mmHg)	141 - 150	151 – 155	≥155	A&E visit or hospitalization for malignant hypertension
Diastolic hypertension (mmHg)	91 - 95	96 – 100	>100	A&E visit or hospitalization for malignant hypertension
Systolic hypotension (mmHg)***	85 - 89	80 – 84	<80	A&E visit or hospitalization for hypotensive shock
Respiratory Rate –breaths per minute	17 - 20	21-25	>25	Intubation

Table 13. ***Severity grading criteria for physical observations.*** **Taken after ≥10 minutes at rest **When resting heart rate is between 60 – 100 beats per minute. Use clinical judgement when characterising bradycardia among some healthy subject populations, for example, conditioned athletes. ***Only if symptomatic (e.g. dizzy/ light-headed)*

**GRADE 0**	None
**GRADE 1**	Mild: Transient or mild discomfort (< 48 hours); No interference with activity; No medical intervention/therapy required
**GRADE 2**	Moderate: Mild to moderate limitation in activity – some assistance may be needed; no or minimal medical intervention/therapy required
**GRADE 3**	Severe: Marked limitation in activity, some assistance usually required; medical intervention/therapy required.
**GRADE 4**	Potentially Life-threatening: requires assessment in A&E or hospitalisation

Table 14. ***Severity grading criteria for local and systemic AEs.***

9.8 Reporting Procedures for Serious AEs

In order to comply with current regulations on SAE reporting to regulatory authorities, the event will be documented accurately and notification deadlines respected. SAEs will be reported on the SAE forms to members of the study team immediately after the Investigators become aware of their occurrence, as described in SOP OVC005 Safety Reporting for CTIMPs. Copies of all reports will be forwarded for review to the Chief Investigator (as the Sponsor's representative) within 24 hours of the Investigator being aware of the suspected SAE. The DSMB will be notified of SAEs that are deemed possibly, probably or definitely related to study interventions; the chair of DSMB will be notified immediately (within 24 hours) of the Sponsor being aware of their occurrence. SAEs will not normally be reported immediately to the ethical committee(s) unless there is a clinically important increase in occurrence rate, an unexpected outcome, or a new event that is likely to affect safety of trial volunteers, at the discretion of the Chief Investigator and/or DSMB. In addition to the expedited reporting above, the Investigator shall include all SAEs in the annual Development Safety Update Report (DSUR) report.

Cases falling under the Hy's Law should be reported as SAEs. A Hy's Law Case is defined by FDA Guidance for Industry "Drug-Induced Liver Injury: Premarketing Clinical Evaluation" (2009). Any study subject with an increase in Aspartate Aminotransferase (AST) or **Alanine Aminotransferase (ALT) ≥ 3x Upper Limit of Normal (ULN) together with Total Bilirubin ≥2xULN**, **where no other reason can be found to explain the combination of increases**, e.g., elevated serum alkaline phosphatase (ALP) indicating cholestasis, viral hepatitis A, B or C, or another drug capable of causing the observed injury.

9.9 Reporting Procedures for SUSARS

All SUSARs (including SUSARs related to the non-IMP where there is a possibility of an interaction between the non-IMP and IMP) will be reported by the sponsor delegate to the relevant Competent Authority and to the REC and other parties as applicable. For fatal and life-threatening SUSARS, this will be done no later than 7 calendar days after the Sponsor or delegate is first aware of the reaction. Any additional relevant information will be reported within 8 calendar days of the initial report. All other SUSARs will be reported within 15 calendar days.

Principal Investigators will be informed of all SUSARs for the relevant IMP for all studies with the same Sponsor, whether or not the event occurred in the current trial.

9.10 Development Safety Update Report

A Development Safety Update Report (DSUR) will be prepared annually, within 60 days of the anniversary of the first approval date from the regulatory authority for each IMP. The DSUR will be submitted by the CI to the Competent Authority, Ethics Committee, HRA (where required), Host NHS Trust and Sponsor.

9.11 Procedures to be followed in the event of abnormal findings

Eligibility for enrolment in the trial in terms of laboratory findings will be assessed by clinically qualified staff. Abnormal clinical findings from medical history, examination or blood tests will be assessed as to their clinical significance throughout the trial. Laboratory AEs will be assessed using specific toxicity grading scales adapted from the FDA Toxicity Grading Scale for Healthy Adult and Adolescent Volunteers Enrolled in Preventive Vaccine Clinical Trials. If a test is deemed clinically significant, it may be repeated, to ensure it is not a single occurrence. If a test remains clinically significant, the volunteer will be informed and appropriate medical care arranged as appropriate and with the permission of the volunteer. Decisions to exclude the volunteer from enrolling in the trial or to withdraw a volunteer from the trial will be at the discretion of the Investigator.

9.12 Interim Reviews

The safety profile will be assessed on an on-going basis by the Investigators. The CI and relevant Investigators (as per the trial delegation log) will also review safety issues and SAEs as they arise.

Interim safety reviews are planned after the first volunteer in the intervention arm and after the first 4 participants have been given a dose of the IMP.

Data from pre-clinical studies will be assessed by the CI, relevant investigators and the DSMB a soon as they are available and before up to 100 volunteers receive a dose of the IMP.

Safety data available from the first up to 54 volunteers receiving a dose of the IMP will be reviewed by the CI, relevant investigators and the chair of DSMB before proceeding with vaccination in the remaining volunteers.

The DSMB will review safety data accumulated when the study is fully recruited.

The DSMB will evaluate frequency of events, safety and efficacy data every 4-8 weeks and/or as required. The DSMB will make recommendations concerning the conduct, continuation or modification of the study.

9.13 Data Safety Monitoring Board

A Data Safety Monitoring Board will be appointed to

a) periodically review and evaluate the accumulated study data for participant safety, study conduct, progress, and efficacy.

b) make recommendations concerning the continuation, modification, or termination of the trial.

There will be a minimum of three appropriately qualified committee members of whom one will be the designated chair. The DSMB will operate in accordance with the trial specific charter, which will be established before recruitment starts.

The chair of the DSMB may be contacted for advice and independent review by the Investigator or trial Sponsor in the following situations:

- Following any SAE deemed to be possibly, probably or definitively related to a study intervention.
- Any other situation where the Investigator or trial Sponsor feels independent advice or review is important.

The DSMB will review SAEs deemed possibly, probably or definitively related to study interventions. The DSMB will be notified within 24 hours of the Investigators' being aware of their occurrence. The DSMB has the power to place the study on hold if deemed necessary following a study intervention-related SAE.

9.14 Safety Group Holding Rules

Safety holding rules have been developed considering the fact that this is a first-in-human study. Safety holding rules apply to participants receiving ChAdOx1 nCoV-19 only.

Solicited AEs are those listed as foreseeable ARs in section 9.3 of the protocol, occurring within the first 7 days after vaccination (day of vaccination and six subsequent days). ‘Unsolicited adverse events’ are adverse events other than the foreseeable ARs occurring within the first 7 days, or any AEs occurring after the first 7 days after vaccination

9.14.1 Group holding rules

For safety reasons, the first volunteer to receive a new vaccine will be vaccinated alone and the trial Investigators will wait 48 hours (±24 hours) before vaccinating subsequent volunteers. Three further volunteers may be vaccinated 48 hours (±24 hours) after the first and then at least another 48 hours (±24 hours) gap will be left before vaccinating the rest of the volunteers. Group holding rules mentioned below will apply to all study Groups

- **Solicited local adverse events:**
  - If more than 25% of doses of the vaccine at a given time point (e.g. Day 0, Day 28) in a study group are followed by the same Grade 3 solicited local adverse event beginning within 2 days after vaccination (day of vaccination and one subsequent day) and persisting at Grade 3 for >72 hrs
- **Solicited systemic adverse events:**
  - If more than 25% of doses of the vaccine at a given time point (e.g. Day 0, Day 28) in a study group are followed by the same Grade 3 solicited systemic adverse event beginning within 2 days after vaccination (day of vaccination and one subsequent day) and persisting at Grade 3 for >72 hrs
- **Unsolicited adverse events:**
  - If more than 25% of doses of the vaccine at a given time point (e.g. Day 0, Day 28) in a study group are followed by the same Grade 3 unsolicited adverse event beginning within 2 days after vaccination (day of vaccination and one subsequent day) and persisting at Grade 3 for >72 hrs
- **Laboratory adverse event:**
  - If more than 25% of doses of the vaccine at a given time point (e.g. Day 0, Day 28) in a study group are followed by the same Grade 3 laboratory adverse event beginning within 3 days after vaccination and persisting at Grade 3 for >72 hrs
- **A serious adverse event considered possibly, probably or definitely related to vaccination occurs**
  - If an SAE occurs in any one individual, which is possibly, probably or definitely related to vaccination this would trigger a holding rule. There are two exemptions from this rule, which would not activate a holding rule. These include:

- COVID-19 related hospital admissions considered to be at least possibly related to ChAdOx1 nCoV-19 (e.g. if considered to be a clinical presentation of a disease enhancement episode). COVID-19 related SAEs will be regularly reviewed by the DSMB, and a single event will not trigger a holding rule.
- SAEs reported under the Hy's Law requirement will not necessarily trigger a holding rule. These cases will also be reviewed by the DSMB

If any of the above holding rules are activated, then further vaccinations in any group will not occur until a safety review by the DSMB, study sponsor and the chief investigator has been conducted and it is deemed appropriate to restart dosing. The Regulatory Authority will be informed and a request to restart dosing with pertinent data will be submitted as a substantial amendment. The safety review will consider:

- The relationship of the AE or SAE to the vaccine.
- The relationship of the AE or SAE to the vaccine dose, or other possible causes of the event.
- If appropriate, additional screening or laboratory testing for other volunteers to identify those who may develop similar symptoms and alterations to the current Participant Information Sheet (PIS) are discussed.
- New, relevant safety information from ongoing research programs on the various components of the vaccine.

The local ethics committee and vaccine manufacturers will also be notified if a holding rule is activated or released.

All vaccinated volunteers will be followed for safety until resolution or stabilisation (if determined to be chronic sequelae) of their AEs.

9.14.2 Individual stopping rules (will apply to prime-boost group only)

In addition to the above stated group holding rules, stopping rules for individual volunteers will apply (i.e., indications to withdraw individuals from further vaccinations). Study participants who present with at least one of the following stopping rules will be withdrawn from further vaccination in the study:

- **Local reactions:** Injection site ulceration, abscess or necrosis

- **Laboratory AEs:**
  the volunteer develops a Grade 3 laboratory AE considered possibly, probably or definitely related within 7 days after vaccination and persisting continuously at Grade 3 for > 72hrs.

- **Systemic solicited adverse events:**

    - the volunteer develops a Grade 3 systemic solicited AE considered possibly, probably or definitely related within 2 days after vaccination (day of vaccination and one subsequent day) and persisting continuously at Grade 3 for > 72hrs.

- **Unsolicited adverse events:**
    - the volunteer has a Grade 3 adverse event, considered possibly, probably or definitely related to vaccination, persisting continuously at Grade 3 for >72hrs.
    - the volunteer has a SAE considered possibly, probably or definitely related to vaccination.
    - the volunteer has an acute allergic reaction or anaphylactic shock following the administration of vaccine investigational product.

If a volunteer has an acute respiratory illness (moderate or severe illness with or without fever) or a fever (oral temperature greater than 37.8°C) at the scheduled time of administration of investigational product/control, the volunteer will not be enrolled and will be withdrawn from the study.

All vaccinated volunteers will be followed for safety until the end of their planned participation in the study or until resolution or stabilisation (if determined to be chronic sequelae) of their AEs, providing they consent to this.

In addition to these pre-defined criteria, the study can be put on hold upon advice of the DSMB, Chief Investigator, Study Sponsor, regulatory authority, Ethical Committee(s), for any single event or combination of multiple events which, in their professional opinion, jeopardise the safety of the volunteers or the reliability of the data.

10 STATISTICS

10.1 Description of Statistical Methods

Both a fully detailed study level statistical analysis plan (SAP) as well as a separate Statistical Analysis Plan for the Marketing Authorisation Application (MAA SAP) will be written and signed off before any interim data analyses are conducted.

The data from this study will be included in prospective pooled analyses of studies for efficacy and safety of ChAdOx1 nCoV-19 to provide greater precision of both efficacy and safety outcomes.

10.1.1 Efficacy Outcomes

The primary efficacy endpoint is PCR* positive symptomatic COVID-19.

This is defined as a participant with a PCR+* swab and at least one of the following symptoms: cough, fever ≥ 37.8, shortness of breath, anosmia, or ageusia.

Where possible, sensitivity analyses will be conducted using common alternative definitions of virologically-confirmed COVID-19 disease, including those in use in other phase 3 protocols (including but not limited to: USA AstraZeneca phase 3 trial, South Africa COV005 trial, WHO solidarity trial, CEPI definition). This will aid in comparisons between various studies and meta-analyses. These alternative definitions will be detailed in the statistical analysis plan as exploratory analyses.

* Or other nucleic acid amplification test

10.2 Primary efficacy

The primary and secondary analyses will be conducted on participants who are seronegative at baseline. A sensitivity analysis will be conducted including all participants regardless of baseline serostatus.

Analysis of the primary endpoint will be computed as follows:

1. **Efficacy of two doses of vaccine.** Only cases occurring more than 14 days after the second vaccine will be included.

Secondary analysis

2. **Efficacy of at least one standard-dose** of any ChAdOx1 nCoV-19. Cases occurring more than 21 days after the first vaccination

Proportions will be compared between ChAdOx1 nCoV-19 and MenACWY groups using a Poisson regression model with robust variance (Zou 2004). The model will contain terms including treatment group, and age group at randomization if there is a sufficient sample size within each age category. The logarithm of the period at risk for primary endpoint will be used as an offset variable in the model to adjust for volunteers having different follow up times during which the events occur. Vaccine efficacy (VE) will be calculated as (1 – RR) x 100%, where RR is the relative risk of symptomatic infection (ChADOx1 nCoV-19: Control) and 95% confidence intervals will be presented.

If the Poisson regression model with robust variance fails to converge, the exact conditional method for stratified poisson regression will be used.

Cumulative incidence of symptomatic infections will be presented using the Kaplan-Meier method.

Secondary efficacy endpoints will be analysed in the same way as the primary efficacy endpoint.

Analyses will conducted for all adults combined as well as conducting analyses stratified by age cohorts.

All data from participants with PCR*-positive swabs will be assessed for inclusion in the efficacy analyses by two blinded assessors who will independently review each case according to pre-specified criteria as detailed in the statistical analysis plan, to classify each for inclusion in the primary and secondary outcomes. A separate CRF will be designed for this purpose.

All PCR*-positive results will be assessed for the primary outcome, including those with symptoms swabbed by trial staff and other potential sources of information such as health-care workers who are tested at their workplace as either a routine test procedure or due to developing symptoms.

PCR+* swabs from outside the trial (for example, a workplace routine swab result in a healthcare worker) will be reviewed by blinded staff and only included as a potential endpoint if the test was conducted in 1) a medical laboratory with ISO 15189 accreditation (provided by UKAS in UK) AND 2) an assay that is either CE marked or that has a derogation authorisation from the MHRA.

* Or other nucleic acid amplification test

10.3 Safety & Reactogenicity

Counts and percentages of each local and systemic solicited adverse reaction from diary cards, and all unsolicited AEs and SAEs will be presented for each group.

10.4 Immunogenicity

Highly skewed antibody data will be log-transformed prior to analysis. The geometric mean concentration and associated 95% confidence interval will be summarised for each group at each timepoint, by computing the anti-log of the mean difference of the log-transformed data.

The geometric mean concentration at day 28 and the proportion of participants seroconverting to the S-spike protein from day 0 to day 28 will be computed. Comparisons between ChAdOx1 nCoV-19 vaccine and MenACWY groups will be made using a Mann Whitney U test due to the low titres expected in the control group which will cause a non-normal distribution.

Spike-specific T cell responses (ELISpot) will be presented as means and confidence intervals, or medians and interquartile ranges if non-normally distributed at all post vaccination time points. Comparisons between ChAdOx1 nCoV-19 vaccine and MenACWY groups will be made using a Mann Whitney U test due to the low responses expected in the control group which will cause a non-normal distribution.

10.5 Subgroup analyses

Subgroup comparisons of efficacy, and safety will be conducted by incorporating vaccine-group by subgroup interaction terms into appropriate regression models. Subgroup comparisons will only be conducted if there are at least 5 cases in all subgroups.

Comparisons will include:

1. Males vs females

2. Age (18 to 55 years vs 56-<70 years vs 70+ years)

3. Seropositive to S-spike or non-spike proteins at baseline vs not seropositive

4. Health care workers and highly-exposed participants versus others

10.6 Interim and primary analyses of the primary outcome

It is planned that the primary evidence of efficacy and safety for the ChADOx1 nCoV-19 vaccine will be based on global analyses utilizing studies COV001 (the UK P1/2 study), COV002 (the UK P2/3 study), COV003 (the Brazil P3 study) and COV005 (the South Africa P1/2 study) including a pooled analysis across the studies. As such the interim and primary analyses for the primary outcome will be based on cases accumulated across multiple studies, details of which will be specified within the MAA SAP rather than for each individual study. Interim and primary data cuts from this study will therefore be carried out to support the pooled analysis.

The global MAA SAP allows for interim and primary analyses to be conducted once sufficient eligible cases have accumulated, where the overall type 1 error is controlled at the 5% level using a flexible alpha–spending approach that accounts for the incorporation of data from this study into pooled interim analyses under the global MAA SAP.

Evidence of efficacy will be determined if the lower bound of the multiplicity adjusted confidence interval is greater than a 20% threshold. The primary analysis will have approximately 90% power assuming a vaccine efficacy of 60%. A flexible alpha spending approach will be implemented to allow an earlier primary analysis in the situation where accumulation of eligible cases were lower than expected.

Evidence of efficacy at an interim or primary analysis of pooled data will not be considered a reason to stop the trial, but instead will be interpreted as early evidence of efficacy. However if an interim analysis demonstrates evidence of efficacy then a study level analysis according to the study SAP may be used to support study level evidence of efficacy.

10.7 Final Analysis

A final analysis will be conducted at the end of the study. The final study-specific analysis will incorporate all data from the study, including data that has previously contributed to global efficacy estimates under the pooled analysis strategy. The final analysis will be considered a supportive analysis to the global efficacy analysis. Alpha at the final study-specific analysis will be adjusted to incorporate the number of previous global analyses to which the study contributed data in order to control the overall study level type 1 error at 5%. Details will be specified in the study level SAP.

10.8 Procedure for Accounting for Missing, Unused, and Spurious Data.

All available data will be included in the analysis

10.9 Inclusion in Analysis

All vaccinated participants will be included in the analysis unless otherwise specified in the SAP.

10.10 Interim analysis for the combined DSMB

The independent DSMB will meet regularly to review safety data from all available studies of ChAdOx1 nCoV-19 and will assess whether the assumptions underlying the sample size calculation are in line with the observed cases. Additionally the independent DSMB will make recommendations based on the interim analyses to assess evidence of efficacy.

11 DATA MANAGEMENT

11.1 Data Handling

The Chief Investigator will be responsible for all data that accrues from the study.

All study data including participant diary will be recorded directly into an Electronic Data Capture (EDC) system (e.g. OpenClinica, REDCap, or similar) or onto a paper source document for later entry into EDC if direct entry is not available or is not practical at site. This includes safety data, laboratory data and outcome data. Any additional information that needs recording but is not relevant for the CRF (such as signed consent forms etc.) will be recorded on a separate paper source document. All documents will be stored safely and securely in confidential conditions.

All adverse event data (both solicited and unsolicited) reported by the volunteer will be entered onto a volunteer's electronic diary card (eDiary) for a maximum of 28 days following administration of the IMP. The eDiary provides a full audit trail of edits and will be reviewed at each review time-point indicated in the schedule of events. Any adverse event continuing beyond the period of the diary will be copied into the eCRF and followed to resolution, if there is a causal relationship to the IMP, or to the end of the study if there is no causal relationship.

The participants will be identified by a unique trial specific number and code in any database. The name and any other identifying detail will NOT be included in any trial data electronic file.

The EDC system (CRF data) uses a relational database (MySQL/ PostgreSQL) via a secure web interface with data checks applied during data entry to ensure data quality. The database includes a complete suite of features which are compliant with GCP, EU and UK regulations and Sponsor security policies, including a full audit trail, user-based privileges, and integration with the institutional LDAP server. The MySQL and PostgreSQL database and the webserver will both be housed on secure servers maintained by the University of Oxford IT personal. The servers are in a physically secure location in Europe. Backups will be stored in accordance with the IT department schedule daily, weekly, monthly, and are retained for one month, three months, and six months, respectively. The IT servers provide a stable, secure, well-maintained, and high capacity data storage environment. REDCap and OpenClinica are widely-used, powerful, reliable, well-supported systems. Access to the study's database will be restricted to the members of the study team by username and password.

11.2 Record Keeping

The Investigators will maintain appropriate medical and research records for this trial, in compliance with GCP and regulatory and institutional requirements for the protection of confidentiality of volunteers. The Chief

Investigator, co-Investigators and clinical research nurses will have access to records. The Investigators will permit authorised representatives of the Sponsor(s), as well as ethical and regulatory agencies to examine (and when required by applicable law, to copy) clinical records for the purposes of quality assurance reviews, audits and evaluation of the study safety and progress.

All trial records will be stored for a minimum of 5 years after the end of the trial at a secure archiving facility . If volunteers consent to be contacted for future research, information about their consent form will be recorded, retained and stored securely and separately from the research data. If volunteers consent to have their samples stored and used in future research, information about their consent form will be recorded, retained and stored securely as per Biobanking procedures and SOP.

11.3 Source Data and Case Report Forms (CRFs)

All protocol-required information will be collected in CRFs designed by the Investigator. All source documents will be filed in the CRF. Source documents are original documents, data, and records from which the volunteer's CRF data are obtained. For this study, these will include, but are not limited to, volunteer consent form, blood results, GP response letters, laboratory records, diaries, medical records and correspondence. In the majority of cases, CRF entries will be considered source data as the CRF is the site of the original recording (i.e. there is no other written or electronic record of data). In this study this will include, but is not limited to medical history, medication records, vital signs, physical examination records, urine assessments, blood results, adverse event data and details of vaccinations. All source data and volunteer CRFs will be stored securely.

Source data verification requirements will be defined in the trial risk assessment and monitoring plan.

To prevent withdrawal of a participant due to relocation, if there is a nearby participating site and with the consent of the participant, copies of relevant participant research records (such as ICF, paper source documents) will be transferred to the local site using secure email addresses such as nhs.net or by password protected sheets. The electronic research data stored on REDCap will also be transferred to the new site. The original records will be retained by the recruiting site. Consent to transfer information could be completed electronically.

11.4 Data Protection

The study protocol, documentation, data and all other information generated will be held in strict confidence. No information concerning the study or the data will be released to any unauthorised third party, without prior written approval of the sponsor.

11.5 Data Quality

Data collection tools will undergo appropriate validation to ensure that data are collected accurately and completely. Datasets provided for analysis will be subject to quality control processes to ensure analysed data is a true reflection of the source data.

Trial data will be managed in compliance with local data management SOPs. If additional, study specific processes are required, an approved Data Management Plan will be implemented

11.6 Archiving

Study data may be stored electronically on a secure server, and paper notes will be kept in a key-locked filing cabinet at the site. All essential documents will be retained for a minimum of 5 years after the study has finished. The need to store study data for longer in relation to licensing of the vaccine will be subject to ongoing review. For effective vaccines that may be licensed, we may store research data securely at the site at least 15 years after the end of the study, subject to adjustments in clinical trials regulations. Participants' bank details will be stored for 7 years in line with the site financial policy.

General archiving procedures will be conducted in compliance to SOP OVC020 Archiving.

12 QUALITY CONTROL AND QUALITY ASSURANCE PROCEDURES

12.1 Investigator procedures

Approved site-specific standard operating procedures (SOPs) will be used at all clinical and laboratory sites.

12.2 Monitoring

Regular monitoring will be performed according to GCP by the monitor. Following written SOPs and an approved, risk based monitoring plan, the monitor will verify that the clinical trial is conducted and data are generated, documented and reported in compliance with the protocol, GCP and the applicable regulatory requirements. The site will provide direct access to all trial related source data/documents and reports for the purpose of monitoring and auditing by the Sponsor and inspection by local and regulatory authorities.

12.3 Protocol deviation

Any deviations from the protocol will be documented in a protocol deviation form and filed in the trial master file. Each deviation will be assessed as to its impact on volunteer safety and study conduct. Significant protocol deviations will be listed in the end of study report.

12.4 Audit & inspection

The QA manager conducts systems based internal audits to check that trials are being conducted according to local procedures and in compliance with study protocols, departmental SOPs, GCP and applicable regulations.

The Sponsor, trial sites, and ethical committee(s) may carry out audit to ensure compliance with the protocol, GCP and appropriate regulations.

GCP inspections may also be undertaken by the MHRA to ensure compliance with protocol and the Medicines for Human Use (Clinical Trials) Regulations 2004, as amended. The Sponsor will assist in any inspections and will support the response to the MHRA as part of the inspection procedure.

13 SERIOUS BREACHES

The Medicines for Human Use (Clinical Trials) Regulations contain a requirement for the notification of "serious breaches" to the MHRA within 7 days of the Sponsor becoming aware of the breach.

A serious breach is defined as “A breach of GCP or the trial protocol which is likely to effect to a significant degree

(a) the safety or physical or mental integrity of the subjects of the trial; or

(b) the scientific value of the trial”.

In the event that a potential serious breach is suspected the Sponsor will be informed as soon as possible, to allow preliminary assessment of the breach and reporting to the MHRA within the required timelines.

14 ETHICS AND REGULATORY CONSIDERATIONS

14.1 Declaration of Helsinki

The Investigators will ensure that this study is conducted according to the principles of the current revision of the Declaration of Helsinki.

14.2 Guidelines for Good Clinical Practice

The Investigator will ensure that this trial is conducted in accordance with relevant regulations and with Good Clinical Practice.

14.3 Ethical and Regulatory Approvals

Following Sponsor approval the protocol, informed consent form, participant information sheet and any proposed advertising material will be submitted to an appropriate Research Ethics Committee (REC), HRA (where required), regulatory authorities (MHRA in the UK), and host institution(s) for written approval. No amendments to this protocol will be made without consultation with, and agreement of, the Sponsor.

The Investigator is responsible for ensuring that changes to an approved trial, during the period for which regulatory and ethical committee(s) approval has already been given, are not initiated without regulatory and ethical committee(s)' review and approval except to eliminate apparent immediate hazards to the subject (i.e as an Urgent Safety Measure).

14.4 Volunteer Confidentiality

The study will comply with the General Data Protection Regulation (GDPR) and Data Protection Act 2018, which require data to be de-identified as soon as it is practical to do so. The processing of personal data of participants will be minimised by making use of a unique participant study number only on all study documents and any electronic database(s), with the exception of informed consent forms and participant ID logs. All documents will be stored securely and only accessible by study staff and authorised personnel. The study staff will safeguard the privacy of participants' personal data. A separate confidential file containing identifiable information will be stored in a secured location in accordance with the current data protection legislation. Photographs taken of vaccination sites (if required, with the volunteer's written, informed consent) will not include the volunteer's face and will be identified by the date, trial code and subject's unique identifier. Once developed, photographs will be stored as confidential records, as above. This material may be shown to other professional staff, used for educational purposes, or included in a scientific publication.

If participants have a positive swab result for COVID-19 during the course of the study then the Public Health Authority will be notified as COVID-19 is a "notifiable disease" and this is legal requirement in the UK. This

may mean participants personal information from their health records will be shared with Public Health either by the processing lab or the study site. Participants may also be contacted by the NHS Test and Trace service.

Samples collected using home swab kits may be processed at laboratories within and outside the UK, as determined by the community testing programme. These laboratories provide a test result for the barcode to NPEx (National Pathology Exchange) and this result is then recombined with participant identifiable information by NHS Digital. NHS Digital provide lab results to the Sponsor (University of Oxford) who will match this with personal data including identifying contact information sent to them by the site in order to centralise the processing of results.

Participants will be required to separately consent to the terms and conditions of the national community swabbing programme, each time they perform a self-swab. This is available at:
https://www.gov.uk/government/publications/coronavirus-covid-19-testing-privacy-information/testing-for-coronavirus-privacy-information

15 FINANCING AND INSURANCE

15.1 Financing

The study is funded through UK Government

15.2 Insurance

The University has a specialist insurance policy in place which would operate in the event of any participant suffering harm as a result of their involvement in the research (Newline Underwriting Management Ltd, at Lloyd's of London). NHS indemnity operates in respect of the clinical treatment which is provided.

15.3 Compensation

Volunteers will be compensated for their time, the inconvenience of having blood tests and procedures, and their travel expenses. The total amount compensated will be approximately **£190-625** depending on the exact number of visits, and whether any repeat or additional visits are necessary. They will be compensated £25 for attending the screening visit. For all other trial visits as outlined in Tables 6-8, compensation will be calculated according to the following:

- Travel expenses: £15 per visit
- Inconvenience of blood tests: £10 per blood donation
- Time required for visit: £20 per hour

Should the volunteer decide to withdraw from the trial before it is completed, payment will be pro rata

16 Publication Policy

The Investigators will be involved in reviewing drafts of the manuscripts, abstracts, press releases and any other publications arising from the study. Data from the study may also be used as part of a thesis for a PhD or MD.

17 DEVELOPMENT OF A NEW PRODUCT/PROCESS OR THE GENERATION OF INTELLECTUAL PROPERTY

Ownership of IP generated by employees of the University vests in the University. The protection and exploitation of any new IP is managed by the University's technology transfer office, Oxford University Innovations. Investigators in this study may benefit from the royalty sharing policy of the University if new intellectual property is generated from the trial. Several investigators are applicants or co-inventors on previous patent filings or patents related to ChAdOx1 vaccines. The University of Oxford, which is partnered with the Oxford University Hospitals NHS Foundation Trust in the NIHR Oxford Biomedical Research Centre, is committed to the translational progress and commercial development of healthcare products potentially meeting medical and global health needs, and does and will work with commercial partners towards these goals.

18. References

1. Zhu, N., et al., *A Novel Coronavirus from Patients with Pneumonia in China, 2019.* New England Journal of Medicine, 2020. **382**(8): p. 727-733.
2. Lu, R., et al., *Genomic characterisation and epidemiology of 2019 novel coronavirus: implications for virus origins and receptor binding.* Lancet (London, England), 2020. **395**(10224): p. 565-574.
3. Li, F., *Structure, Function, and Evolution of Coronavirus Spike Proteins.* Annual review of virology, 2016. **3**(1): p. 237-261.
4. Zhou, P., et al., *A pneumonia outbreak associated with a new coronavirus of probable bat origin.* Nature, 2020.
5. Alharbi, N.K., et al., *ChAdOx1 and MVA based vaccine candidates against MERS-CoV elicit neutralising antibodies and cellular immune responses in mice.* Vaccine, 2017. **35**(30): p. 3780-3788.
6. Liu, L., et al., *Anti–spike IgG causes severe acute lung injury by skewing macrophage responses during acute SARS-CoV infection.* JCI insight, 2019. **4**(4).
7. Tseng, C.T., et al., *Immunization with SARS coronavirus vaccines leads to pulmonary immunopathology on challenge with the SARS virus.* PLoS One, 2012. **7**(4): p. e35421.
8. Weingartl, H., et al., *Immunization with modified vaccinia virus Ankara-based recombinant vaccine against severe acute respiratory syndrome is associated with enhanced hepatitis in ferrets.* J Virol, 2004. **78**(22): p. 12672-6.
9. Agrawal, A.S., et al., *Immunization with inactivated Middle East Respiratory Syndrome coronavirus vaccine leads to lung immunopathology on challenge with live virus.* Hum Vaccin Immunother, 2016. **12**(9): p. 2351-6.
10. Alharbi, N.K., et al., *Humoral Immunogenicity and Efficacy of a Single Dose of ChAdOx1 MERS Vaccine Candidate in Dromedary Camels.* Scientific reports, 2019. **9**(1): p. 1-11.
11. Munster, V.J., et al., *Protective efficacy of a novel simian adenovirus vaccine against lethal MERS-CoV challenge in a transgenic human DPP4 mouse model.* npj Vaccines, 2017. **2**(1): p. 1-4.
12. Antrobus, R.D., et al., *Clinical assessment of a novel recombinant simian adenovirus ChAdOx1 as a vectored vaccine expressing conserved Influenza A antigens.* Mol Ther, 2014. **22**(3): p. 668-74.
13. Coughlan, L., et al., *Heterologous Two-Dose Vaccination with Simian Adenovirus and Poxvirus Vectors Elicits Long-Lasting Cellular Immunity to Influenza Virus A in Healthy Adults.* EBioMedicine, 2018.
14. Wilkie, M., et al., *A phase I trial evaluating the safety and immunogenicity of a candidate tuberculosis vaccination regimen, ChAdOx1 85A prime - MVA85A boost in healthy UK adults.* Vaccine, 2020. **38**(4): p. 779-789.
15. Folegatti, P.M., et al., *Safety and immunogenicity of a candidate Middle East respiratory syndrome coronavirus viral-vectored vaccine: a dose-escalation, open-label, non-randomised, uncontrolled, phase 1 trial.* The Lancet Infectious Diseases, 2020.
16. Modjarrad, K., *MERS-CoV vaccine candidates in development: The current landscape.* Vaccine, 2016. **34**(26): p. 2982-7.

APPENDIX A: AMENDMENT HISTORY

Amendment No.	Protocol Version No.	Date issued	Author(s) of changes	Details of Changes made
N/A	1.0	13 Mar 2020	Pedro Folegatti, Daniel Jenkin, Sarah Gilbert, Andrew Pollard, Adrian Hill, Merryn Voysey	N/A
N/A	2.0	18 Mar 2020	Pedro Folegatti	Neutralising antibodies listed as exploratory endpoints instead of secondary ones; Added blood samples for safety and immunogenicity at COVID-19 testing visit, in addition to nose/throat swabs; Day 2 visit replaced with Day 3; Replaced references to Oxford site with 'the site'. COI statement updated for Prof Adrian Hill
SA01	3.0	23 Mar 2020	Pedro Folegatti	Replaced saline placebo with active comparator (MenACWY). Added Dr Maheshi Ramasamy and Prof Matthew Snape as investigators; Expanded list of exclusion criteria, adding all vulnerable groups considered at risk of severe COVID-19 disease; Changed procedures for swabbing volunteers when symptomatic to include ECDC case definition; Removed reference to separate consent procedure for Biobank (this is already covered in the consent form); Increased blood volumes for exploratory immunology at D3 from 10 to 50mL; Added COVID-19 Testing +7 visit; Added PAXgenes; Consent to be taken by

Amendment No.	Protocol Version No.	Date issued	Author(s) of changes	Details of Changes made
				both clinicians and appropriately trained and delegated research nurses when required.
SA02	4.0	20 Apr 2020	Pedro Folegatti, Merryn Voysey, Emma Plested	Increased sample size with added group 4; Added phone/video call review at D1 for groups 1 and 3; Added phone/video call review as required during COVID-19 follow-up; updated statistical analysis section; clarifications to exclusion criteria; expanded list of AESI and instruction to report eosinophilia greater than 1.5x10^9/L as SAE. Addition of sites. Addition of monitoring roles..; a reduced dose has been introduced for the booster doses in group 3 (2.5x10^10 vp) as this will allow for an assessment of dose sparing, optimising the available vials; re-consent to be taken by appropriately trained and delegated members of the team; added household weekly questionnaires as a measure of exposure to COVID-19; added lab AE grading scale as an appendix to the protocol.
SA03	5.0	21 Apr 2020	Pedro Folegatti	Clarification to screening procedures for high risk COVID-19 exposure participants.
SA05	6.0	06 May 2020	Pedro Folegatti, Merryn Voysey	Added Dr Angela Minassian as an investigator; Added prophylactic paracetamol use to a subset of participants in group 4; Added an exploratory objective to describe safety, reactogenicity, immunogenicity and efficacy amongst those receiving paracetamol for 24h; Clarifications to IMP

Amendment No.	Protocol Version No.	Date issued	Author(s) of changes	Details of Changes made
				storage locations; Updated sample size to reflect the actual number of vials available to run the study.; Corrected lab AE stopping rule to reflect timing of safety bloods taken.
SA06	7.0	19 May 2020	Pedro Folegatti	Booster dose changed back to $5x10^{10}$vp for consistency with COV002; clarification that holding rules apply to ChAdOx1 nCoV-19 only and not the licensed comparator; clarification to notification of DSMB chair on SAEs deemed at least possibly related which should happen within 24h of the Sponsor becoming aware of the event; additional steps taken to avoid unblinding of participants when attending for COVID-19 swabbing visits; Confirmation that additional steps may be taken to manage the blinding of staff when assessing endpoints or managing the unblinding of staff when managing safety reporting; correction of formatting and typographical errors
SA08	8.0	22 Jun 2020	Pedro Folegatti	Added booster vaccination on a subset of participants enrolled in Group 2.
SA09	9.0	30 Jul 2020	Pedro Folegatti, Maheshi Ramasamy, Merryn Voysey, Hannah Robinson	Added booster vaccination on all participants in Groups 2 and 4; Added Hy's law criteria to be reported as SAE; Clarifications to holding rules; Added information on Cobra material to be used in booster doses; added stool samples on PCR positive participants, added an observation time for post booster vaccinations; changed the D364 visit from

Amendment No.	Protocol Version No.	Date issued	Author(s) of changes	Details of Changes made
				optional.; changed swabbing pathway (S7 to be conducted only on positive cases, added S3-5 visit for second swab or home testing); updated statistical analysis section; Re-consent may be collected with electronic signatures if required for infection control purposes; an update to the 'Planned receipt of any vaccine other than the study intervention within 30 days before and after each study vaccination' exclusion criteria to allow an exception for the seasonal flu vaccine; clarifications made to visit time points.
SA12	10	16 Sep 2020	Pedro Folegatti, Merryn Voysey	Clarification to efficacy objectives to include efficacy against severe disease; Clarification to exclusion criteria where only licensed seasonal influenza vaccines will be allowed within 7 days of vaccine administration; Clarification to requirements for contraception (at least 3 months post last vaccination); Clarifications to the statistical analysis section on primary, secondary and exploratory analysis; Clarifications to symptomatic pathway; Correction of formatting and typographical errors; Addition of transfer possibility if participants are relocating to an area with a participating site; Update to the vaccine expiry time
SA14	11	21 Oct 2020	Hannah Robinson	Exclusion criteria updated to allow administration of licensed pneumococcal vaccines within 7 days of study vaccine administration; Clarification that home

Amendment No.	Protocol Version No.	Date issued	Author(s) of changes	Details of Changes made
				swabs may be processed outside of the UK; Clarification of the flow of information from home swabbing results to sponsor; Correction of blood sample volumes in table 11; statistician inserted, statistical 'section 10' updated.
SA15	12		Maheshi Ramasamy	Clarification that nucleic acid amplification assays (NAAT) will be used to confirm viral infection; Typographical correction to amendment reference for V11.0 protocol change summary

List details of all protocol amendments here whenever a new version of the protocol is produced.

Appendix. Toxicity grading scale for Lab AEs

**Haematology**			**Lab Range**	**Grade 1**	**Grade 2**	**Grade 3**	**Grade 4**
Haemoglobin Absolute	**Male**	g/l	130 - 170	115-125	100-114	85-99	<85
Haemoglobin Absolute	**Female**		120 - 150	105-113	90-104	80-89	<80
Haemoglobin Change from Baseline (Decrease)			n/a	10-15	16-20	21-50	>50
White Blood Cells	**Elevated**	x109/l	11	11.5-15.00	15.01-20	20.01-25	>25
White Blood Cells	**Low**		4.0	2.5-3.5	1.5-2.49	1.0-1.49	<1.0
Platelets	**Low**		150-400	125-140	100-124	25-99	<25
Neutrophils	**Low**		2.0-7.0	1.5-1.99	1.0-1.49	0.5-0.99	<0.50
Lymphocytes	**Low**		1.0-4.0	0.75-0.99	0.5-0.74	0.25-0.49	<0.25
Eosinophils	**Elevated**	x109/l	0.02 - 0.5	0.65-1.5	1.51-5.00	>5.00	Hypereosinophilia
**Biochemistry**							
Sodium	**Elevated**	mmol/l	145	146-147	148-149	150-155	>155
Sodium	**Low**		135	132-134	130-131	125-129	<125
Potassium	**Elevated**	mmol/l	5	5.1-5.2	5.3-5.4	5.5-6.5	>6.5
Potassium	**Low**		3.5	3.2-3.3	3.1	2.5-3.0	<2.5
Urea	**Elevated**	mmol/l	2.5 - 7.4	8.2-9.3	9.4-11.0	>11.0	Requires dialysis
Creatinine	**Elevated**	µmol/l	49 - 104	1.1-1.5xULN<br>114-156	>1.5-3.0xULN<br>157-312	>3.0xULN<br>>312	Requires dialysis
Bilirubin	**Normal LFTs**	µmol/l	0-21	1.1-1.5xULN<br>23-32	>1.5-2xULN<br>33-42	>2-3xULN<br>43-63	>3xULN<br>≥64
Bilirubin	**Abnormal LFTs**	µmol/l	0 - 21	1.1-1.25xULN<br>23-26	>1.25-1.5xULN<br>27-32	>1.5-1.75xULN<br>33-37	>1.75xULN<br>>37
ALT		IU/l	10 - 45	1.1-2.5xULN<br>49-112	>2.-5xULN<br>113-225	>5-10xULN<br>226-450	>10xUPN<br>>450
Alk Phosphatase	**Elevated**	IU/l	30 -130	1.1-2xULN<br>143-260	>2.-3xULN<br>261-390	>3-10xULN<br>391-1300	>10xULN<br>>1300
Albumin		g/l	32-50	28-31	25-27	<25	-

Normal lab ranges may vary between sites and should be adapted accordingly

Trial Title: A phase 2/3 study to determine the efficacy, safety and immunogenicity of the candidate Coronavirus Disease (COVID-19) vaccine ChAdOx1 nCoV-19

Short title Investigating a Vaccine Against COVID-19

Study Reference: (COV002)

Protocol Version: 14.0

Date: 09 November 2020

EudraCT number: 2020-001228-32

REC Reference: **20/SC/0179**

IRAS Reference: 281904

Chief Investigator: Prof Andrew Pollard

Sponsor: University of Oxford

Funder: **NIHR**

Key Trial Contacts

**Chief Investigator**	Prof Andrew Pollard
**Address**	Centre for Clinical Vaccinology and Tropical Medicine<br>University of Oxford,<br>Churchill Hospital, Old Road, Headington<br>Oxford, OX3 7LE<br>Email: andrew.pollard@paediatrics.ox.ac.uk
**Clinical Trials Unit**	Primary Care and Vaccines Collaborative CTU
**Investigators**	Prof Brian Angus<br>Centre for Clinical Vaccinology and Tropical Medicine<br>Churchill Hospital, Old Road, Headington<br>Oxford, OX3 7LE<br>Email: brian.angus@ndm.ox.ac.uk<br><br>Prof Adrian Hill<br>The Jenner Institute, University of Oxford<br>Old Road Campus Research Building (ORCRB)<br>Roosevelt Drive Oxford OX3 7DQ<br>Email: adrian.hill@ndm.ox.ac.uk<br><br>Prof Sarah Gilbert<br>The Jenner Institute, University of Oxford<br>Old Road Campus Research Building (ORCRB)<br>Roosevelt Drive Oxford OX3 7DQ<br>Email: sarah.gilbert@ndm.ox.ac.uk<br><br>Dr Pedro Folegatti<br>Centre for Clinical Vaccinology and Tropical Medicine<br>Churchill Hospital, Old Road, Headington<br>Oxford, OX3 7LE<br>Email: pedro.folegatti@ndm.ox.ac.uk<br><br>Dr Alexander Douglas<br>The Jenner Institute<br>Wellcome Centre for Human Genetics<br>Roosevelt Drive<br>Oxford, OX3 7BN<br>Email: sandy.douglas@ndm.ox.ac.uk<br><br>Prof Matthew Snape<br>Oxford Vaccine Group<br>Centre for Clinical Vaccinology and Tropical Medicine<br>University of Oxford,<br>Churchill Hospital, Old Road, Headington<br>Oxford, OX3 7LE<br>Email : matthew.snape@paediatrics.ox.ac.uk

Dr Maheshi Ramasamy
Oxford Vaccine Group
Centre for Clinical Vaccinology and Tropical Medicine
University of Oxford,
Churchill Hospital, Old Road, Headington
Oxford, OX3 7LE
Email : maheshi.ramasamy@paediatrics.ox.ac.uk

Prof Teresa Lambe
The Jenner Institute, University of Oxford
Old Road Campus Research Building (ORCRB)
Roosevelt Drive Oxford OX3 7DQ
Email: teresa.lambe@ndm.ox.ac.uk

Prof Katie Ewer
The Jenner Institute, University of Oxford
Old Road Campus Research Building (ORCRB)
Roosevelt Drive Oxford OX3 7DQ
Email: katie.ewer@ndm.ox.ac.uk

Dr Angela Minassian
Centre for Clinical Vaccinology and Tropical Medicine
University of Oxford,
Churchill Hospital, Old Road, Headington
Oxford, OX3 7LE
Email : angela.minassian@ndm.ox.ac.uk

Dr Paola Cicconi
Centre for Clinical Vaccinology and Tropical Medicine
University of Oxford,
Churchill Hospital, Old Road, Headington
Oxford, OX3 7LE
Email: paola.cicconi@ndm.ox.ac.uk

Trial Sites

Centre for Clinical Vaccinology & Tropical Medicine
University of Oxford
Churchill Hospital, Old Road, Headington, Oxford, OX3 7LE
PI at Site: Professor Adrian Hill
Email: adrian.hill@ndm.ox.ac.uk

NIHR WTCRF
University Hospital Southampton NHS Foundation Trust
Southampton, SO16 6YD
PI at Site: Professor Saul Faust
Email: s.faust@soton.ac.uk

IHR Imperial CRF
NIHR Imperial Clinical Research Facility Imperial College
Hammersmith Hospital, Imperial College NHS Trust
150 Du Cane Road, London, W12 0HS
PI at Site: Dr Katrina M. Pollock
Email: k.pollock@imperial.ac.uk

St Georges University Hospital NHS Foundation Trust
Blackshaw Road, Tooting, London
SW17 0QT
PI at site: Professor Paul Heath
Email: pheath@sgul.ac.uk

University Hospitals Bristol and Weston NHS Foundation Trust
Marlborough Street, Bristol, Avon
BS1 3NU
PI at site: Professor Adam Finn
Email: Adam.Finn@bristol.ac.uk

North Bristol NHS Trust
Southmead Hospital
Southmead Road
Westbury-on-Trym
Bristol, BS10 5NB
PI at site: Dr Rajeka Lazarus
Email: rajeka.lazarus@uhbw.nhs.uk

Oxford University Hospital Foundation Trust
John Radcliffe Hospital
Headley Way, Headington
Oxford
OX3 9DU
PI at site: Dr Maheshi Ramasamy
Email: maheshi.ramasamy@paediatrics.ox.ac.uk

University of Nottingham Health Service
Cripps Health Centre, University Park
Nottingham, NG7 2QW
PI at site: Dr David Turner
Email: david.turner@nottingham.ac.uk

Nottingham University Hospitals NHS Trust
C Floor, South Block
Queen's Medical Centre Campus
Derby Road Nottingham NG7 2UH
PI at site: Dr David Turner
Email: david.turner@nottingham.ac.uk

Sheffield Teaching Hospitals NHS Foundation Trust
Royal Hallamshire Hospital, Glossop Road
Sheffield S10 2RX
PI at site: Dr. Thomas Darton
Email: t.darton@sheffield.ac.uk

University Hospitals Birmingham NHS Foundation Trust (UHB)
Queen Elizabeth Hospital Birmingham
Mindelsohn Way
Birmingham B15 2TH
PI at site: Dr Christopher Green
Email: cgreen16@nhs.net

Wales (Public Health Wales)
Aneurin Bevan Local Health Board of Aneurin Bevan Local Health Board Headquarters,
St. Cadoc's Hospital,
Lodge Road,
Caerleon,
Newport,
NP18 3XQPI at site: Dr Chris J Williams
Email: Christopher.williams25@wales.nhs.uk

Hull University Teaching Hospitals NHS Trust (HUTH).
Hull Royal Infirmary
Anlaby Road, Hull
HU3 2JZ
PI at site: Dr Patrick Lillie
Email: patrick.lillie@hey.nhs.uk

Greater Glasgow and Clyde NHS Board
NHS Greater Glasgow and Clyde Corporate HQ
J B Russell House
Gartnavel Royal Hospital Campus
1055 Great Western Road
GLASGOW
G12 0XH
PI at site: Professor Emma Thomson (QEUH)
Co Investigator: Prof Andrew Smith (GRI)
PI Email: Emma.Thomson@glasgow.ac.uk
Co-Investigator Email: Andrew.Smith@glasgow.ac.uk

Guy's and St Thomas' NHS Foundation Trust
Department of Infection, St Thomas Hospital
Westminster Bridge Road, London SE1 7EH
PI at site: Dr Anna Goodman

Email: Anna.goodman@gstt.nhs.uk

Liverpool School of Tropical Medicine (LSTM)
Liverpool School of Tropical Medicine
Pembroke Place
Liverpool L3 5QA
PI at site: Dr Andrea Collins
Email: Andrea.collins@lstmed.ac.uk

The Newcastle upon Tyne Hospitals NHS Foundation Trust
Royal Victoria Infirmary
Newcastle upon Tyne
NE1 4LP
PI at site: Dr Christopher Duncan
Email: christopherduncan@nhs.net

UCLH
250 Euston Road, London
NW1 2PG
PI at site: Prof Vincenzo Libri
Email: vincenzo.libri@ucl.ac.uk

Lothian NHS Board
Waverleygate
2-4 Waterloo Place
Edinburgh
EH1 3EGPI at site: Dr Rebecca Sutherland
Email: Rebecca.sutherland@nhslothian.scot.nhs.uk

Cambridge
Cambridge Clinical Research Facility
Cambridge Biomedical Campus, Hills Road
Cambridge CB2 0QQ
PI at site: Dr Mark Toshner
Email: mrt34@medschl.cam.ac.uk

Northwick Park Hospital
London North West University Healthcare Trust, Northwick Park Hospital, Watford Road, Harrow, HA1 3UJ.
PI at site: Dr Alastair McGregor
Email: alastair.mcgregor1@nhs.net

Sponsoring Institution

University of Oxford
Clinical Trials and Research Governance
Joint Research Office,
Boundary Brook House
Churchill Drive, Headington

	Oxford OX3 7GB<br>Email: ctrg@admin.ox.ac.uk
**Funder**	**NIHR**
**Lead Monitor**	**Oxford Vaccine Centre**<br>CCVTM<br>Churchill Hospital<br>Headington<br>OXON<br>OX3 7LE<br>Email: covid19@ndm.ox.ac.uk
**DSMC Chair**	Professor Robert Heyderman (Chair)<br>University College London
**Statistician**	Merryn Voysey<br>**Oxford Vaccine Centre**<br>CCVTM, Churchill Hospital<br>Headington, OXON<br>OX3 7LE<br>merryn.voysey@paediatrics.ox.ac.uk<br><br>Signature:

Confidentiality Statement

This document contains confidential information that must not be disclosed to anyone other than the Sponsor, the Investigator Team, HRA, host organisation, and members of the Research Ethics Committee and other regulatory bodies. This information cannot be used for any purpose other than the evaluation or conduct of the clinical investigation without the prior written consent of Prof Andrew Pollard.

Statement of Compliance

The trial will be conducted in compliance with the protocol, the principles of Good Clinical Practice, Medicines for Human Use (Clinical Trial) Regulations 2004 (as amended) and all other applicable regulatory requirements.

Investigator Agreement and Notification of Conflict of Interest

Details can be found in Appendix 1

Table of Contents

1 SYNOPSIS 13
2 ABBREVIATIONS 26
3 BACKGROUND AND RATIONALE 28
3.1 Background 28
3.2 Preclinical studies 29
3.2.1 Immunogenicity (Jenner Institute, unpublished) 29
3.2.2 Non-human primate efficacy and immunogenicity – NIH (pre-print) 33
3.2.3 Antibody Dependant Enhancement and Immunopathology 38
3.3 Previous clinical experience 39
3.4 Rationale 45
3.4.1 Rationale for including older age groups 46
3.4.2 Rationale for including younger age groups 47
3.4.3 Phase I/II study - COV001 48
3.4.4 Rationale for including HIV Infected persons 48
4 OBJECTIVES AND ENDPOINTS 49
5 TRIAL DESIGN 57
5.1 Study groups 58
5.2 Trial volunteers 63
5.3 Definition of End of Trial 63
5.4 Potential Risks for volunteers 63
5.5 Known Potential Benefits 65
6 RECRUITMENT AND WITHDRAWAL OF TRIAL VOLUNTEERS 65
6.1 Identification of Trial Volunteers 65
6.2 Informed consent 67
6.3 Inclusion and exclusion criteria 69

6.3.1 Inclusion Criteria 69
6.3.2 Exclusion Criteria 70
6.3.3 Re-vaccination exclusion criteria (two-dose groups only) 72
6.3.4 Effective contraception for female volunteers 72
6.3.5 Withdrawal of Volunteers 73
6.4 Pregnancy 74
7 CLINICAL PROCEDURES 74
7.1 Schedule of Attendance 74
7.2 Observations, medical history and physical examination 74
7.3 Study visits 77
7.3.1 Screening visit 77
7.3.2 Day 0: Enrolment and vaccination visit 78
7.3.2.1 Vaccination 78
7.3.2.2 Sequence of Enrolment and Vaccination of Volunteers 80
7.3.3 Subsequent visits 80
7.3.4 Participants under quarantine 103
7.3.5 Symptomatic volunteers 103
7.3.6 Weekly PCR samples 104
7.3.7 Stool samples 104
7.4 Household Weekly Questionnaire (optional) 105
7.4.1 Medical notes review 105
7.4.2 Randomisation, blinding and code-breaking 105
8 INVESTIGATIONAL PRODUCT 107
8.1 Description of ChAdOx1 nCoV-19 107
8.2 Supply 107
8.3 Storage 107

8.4 Administration......107

8.5 Rationale for selected dose......108

8.6 Minimising environmental contamination with genetically modified organisms (GMO)......112

8.7 Control vaccine......112

8.8 Compliance with Trial Treatment......113

8.9 Accountability of the Trial Treatment......113

8.10 Concomitant Medication......113

8.11 Provision of Treatment for Controls......114

9 ASSESSMENT OF SAFETY......114

9.1 Definitions......114

9.1.1 Adverse Event (AE)......114

9.1.2 Adverse Reaction (AR)......114

9.1.3 Serious Adverse Event (SAE)......114

9.1.4 Serious Adverse Reaction (SAR)......115

9.1.5 Suspected Unexpected Serious Adverse Reaction (SUSAR)......115

9.2 Expectedness......115

9.3 Foreseeable Adverse Reactions:......116

9.4 Adverse Events of Special Interest......116

9.5 Causality......116

9.6 Reporting Procedures for All Adverse Events......117

9.7 Assessment of severity......119

9.8 Reporting Procedures for Serious AEs......121

9.9 Reporting Procedures for SUSARS......122

9.10 Development Safety Update Report......122

9.11 Procedures to be followed in the event of abnormal findings 122
9.12 Interim Safety Reviews 123
9.13 Data Safety Monitoring Board 123
9.14 Safety Group Holding Rules 124
9.14.1 Individual stopping rules (will apply to prime-boost groups only) 126
10 STATISTICS 128
10.1 Description of Statistical Methods 128
10.2 Primary efficacy 128
10.3 Safety & Reactogenicity 129
10.4 Immunogenicity 129
10.5 Subgroup analyses 130
10.6 Interim and primary analyses of the primary outcome 130
10.7 Final Analysis 131
10.8 Procedure for Accounting for Missing, Unused, and Spurious Data. 131
10.9 Inclusion in Analysis 131
10.10 Interim analysis for the combined DSMB 131
11 DATA MANAGEMENT 133
11.1 Data Handling 133
11.2 Record Keeping 134
11.3 Source Data and Case Report Forms (CRFs) 134
11.4 Data Protection 135
11.5 Data Quality 135
11.6 Archiving 135
12 QUALITY CONTROL AND QUALITY ASSURANCE PROCEDURES 136
12.1 Investigator procedures 136

12.2 Trial Steering Committee 136

The trial steering committee will meet regularly and as required. 136

12.3 Monitoring....... 136

12.4 Protocol deviation 137

12.5 Audit & inspection....... 137

13 SERIOUS BREACHES 137

14 ETHICS AND REGULATORY CONSIDERATIONS....... 138

14.1 Declaration of Helsinki 138

14.2 Guidelines for Good Clinical Practice 138

14.3 Ethical and Regulatory Approvals 138

14.4 Volunteer Confidentiality....... 138

15 FINANCING AND INSURANCE 139

15.1 Financing 139

15.2 Insurance....... 139

15.3 Compensation 140

15.4 Publication Policy 140

16 DEVELOPMENT OF A NEW PRODUCT/ PROCESS OR THE GENERATION OF INTELLECTUAL PROPERTY....... 140

17 Appendix....... 153

1 SYNOPSIS

**Title**	A phase 2/3 study to determine the efficacy, safety and immunogenicity of the candidate Coronavirus Disease (COVID-19) vaccine ChAdOx1 nCoV-19
**Trial Identifier**	COV002
**Trial Registration**	EudraCT number: 2020-001228-32<br>REC Reference: 20/SC/0179<br>IRAS: 281904
**Clinical Phase**	2/3
**Design**	A single-blind, randomised safety and efficacy study, with immunogenicity sub studies in older and younger age groups
**Population**	Main efficacy trial: Healthy adults aged ≥18 years.<br>Sequential age escalation/de-escalation immunogenicity sub studies:<br>1. Healthy adults aged between 56 – <70 years<br>2. Healthy adults aged 70 years or older<br>3. Healthy children aged 5 to 12 years, inclusive<br>4. Healthy adults aged 18 – 55 years.<br>5. HIV positive adults aged 18 – 55 years.
**Planned Sample Size**	Total number to enrol: up to 12,390 participants.<br>**Sequential age escalation/de-escalation groups:**<br>**Group 1: Adults aged between 56 – 69 years**<br>a1) Single dose ChAdOx1 nCoV-19 5x10^{10}vp (Abs 260)*, N=30, OR<br>a2) Single dose MenACWY N=10, OR<br>a3) Two-dose ChAdOx1 nCoV-19 5x10^{10}vp (Abs 260) prime and 0.5mL (3.5 – 6.5 × 10^{10} vp, Abs 260, corrected for PS80) boost*, N=up to 30 participants

recruited from group 1a1 will be invited to receive a booster dose at the earliest available opportunity (minimum 4 weeks from prime), OR

a4) Two-dose MenACWY, N=up to 10 participants recruited from group 1a2 will be invited to receive a booster dose at the earliest available opportunity (minimum 4 weeks from prime).

b1) Two-dose ChAdOx1 nCoV-19 $5x10^{10}$vp (Abs 260) prime and 2.2 $x10^{10}$vp (qPCR) boost * (4-6 weeks apart), N=30, OR

b2) Two-dose MenACWY(4-6 weeks apart) , N=10

Group 2: Adults aged 70 years and above

a1) Single dose ChAdOx1-nCoV-19 $5x10^{10}$vp (Abs 260)*, N=50, OR

a2) Single dose MenACWY N=10, OR

a3) Two-dose ChAdOx1 nCoV-19 $5x10^{10}$vp (Abs 260) prime and 0.5mL (3.5 – 6.5 × 10^{10} vp, Abs 260, corrected for PS80) boost*, N=up to 50 participants recruited from group 2a1 will be invited to receive a booster dose at the earliest available opportunity (minimum 4 weeks from prime), OR

a4) Two-dose MenACWY, N=up to 10 participants recruited from group 2a2 will be invited to receive a booster dose at the earliest available opportunity (minimum 4 weeks from prime).

b1) Two-dose ChAdOx1 nCoV-19 $5x10^{10}$vp (Abs 260) prime and 2.2 $x10^{10}$vp (qPCR) boost * (4-6 weeks apart), N=50, OR

b2) Two-dose MenACWY (4-6 weeks apart), N=10

Group 3: Children aged 5 – 12 years, inclusive

ChAdOx1 nCoV-19 $2.5x10^{10}$vp (qPCR)*, N=30 ,OR

Control vaccine: MenACWY, N=30

Group 4: Adults aged 18-55 (n=up to 3550)

a1) Single dose ChAdOx1 nCoV-19 $5x10^{10}$vp (Abs 260)*, N= up to 1775, OR

a2) MenACWY, N= up to 1775 OR

b1) Two-dose ChAdOx1 nCoV-19 $5x10^{10}$vp (Abs 260) prime and $2.2x10^{10}$vp (qPCR) boost*, (4-6 weeks apart), N= up to 50, OR

b2) Two-dose MenACWY, (4-6 weeks apart), N=up to 50

NB: A subset of up to 100 participants in group 4a will be invited to receive a booster dose in 4b, keeping the overall sample size in group 4 the same.

c1) Two-dose ChAdOx1 nCoV-19 $5x10^{10}$vp (Abs 260) prime and 0.5mL (3.5 – 6.5 × 10^{10} vp, Abs 260, corrected for PS80) boost* OR ChAdOx1 nCoV-19 $5x10^{10}$vp (qPCR) boost, (at least 4 weeks apart), N= up to 1725, OR

c2) Two-dose MenACWY, (at least 4 weeks apart), N=up to 1725

NB: Participants in group 4a, excluding those already in 4b, will be invited to receive a booster dose in 4c, keeping the overall sample size in group 4 the same.

Group 5: Adults aged 18-55 years

a1) ChAdOx1 nCoV-19 $5x10^{10}$vp (Abs 260)*, N= 50 OR

a2) MenACWY, N= 50, OR

a3) Two-dose ChAdOx1 nCoV-19 $5x10^{10}$vp (Abs 260) prime and 0.5mL (3.5 – 6.5 × 10^{10} vp, Abs 260, corrected for PS80) boost*, N=up to 50 participants recruited from group 5a1 will be invited to receive a booster dose at the earliest available opportunity (minimum 4 weeks from prime),

a4) Two-dose MenACWY, N=up to 50 participants recruited from group 5a2 will be invited to receive a booster dose at the earliest available opportunity (minimum 4 weeks from prime).

b1) ChAdOx1 nCoV-19 $5x10^{10}$vp (qPCR)*, N= 25 OR

b2) MenACWY, N= 25

(B cell immunology only)

c1) ChAdOx1 nCoV-19 $5x10^{10}$vp (qPCR)*, N= 25 OR

c2) MenACWY, N= 25

(B and T-cell immunology)

d1) Two dose ChAdOx1 nCoV-19 0.5mL (3.5 – 6.5 × 10^{10} vp Abs 260, corrected for PS80)*, (4-6 weeks apart) N=50, OR

d2) Two dose MenACWY, N= 10

(B cell immunology and T-cell in a subset)

Group 6: Adults aged 18-55 years (n= up to 6000)

a1) ChAdOx1 nCoV-19 $5x10^{10}$vp (qPCR), N= up to 3000

a2) MenACWY, N= up to 3000

b1) Two-dose ChAdOx1 nCoV-19 $5x10^{10}$vp (qPCR) prime and 0.5mL (3.5 – 6.5 × 10^{10} vp Abs 260, corrected for PS80) boost* OR ChAdOx1 nCoV-19 $5x10^{10}$vp (qPCR) boost, (at least 4 weeks apart), N= up to 3000, OR

b2) Two-dose MenACWY, (at least 4 apart), N=up to 3000

NB: Participants in group 6a, will be invited to receive a booster dose in 6b, keeping the overall sample size in group 6 the same.

Group 7: Adults aged between 56 – 69 years (n=80):

a1) Single dose ChAdOx1-nCoV-19 $5x10^{10}$vp (qPCR)*, N=30, OR

a2) Single dose MenACWY N=10, OR

b1) Two-dose ChAdOx1 nCoV-19 $5x10^{10}$vp (qPCR)* (4-6 weeks apart), N=30, OR

b2) Two-dose MenACWY(4-6 weeks apart) , N=10

Group 8: Adults aged 70 years and above (n=120):

a1) Single dose ChAdOx1-nCoV-19 $5x10^{10}$vp (qPCR)*, N=50, OR

a2) Single dose MenACWY N=10, OR

b1) Two-dose ChAdOx1 nCoV-19 $5x10^{10}$vp (qPCR) prime and 0.5mL (3.5 – 6.5 × 10^{10} vp, Abs 260, corrected for PS80) boost* OR ChAdOx1 nCoV-19 $5x10^{10}$vp (qPCR) boost (4-6 weeks apart), N=50, OR

b2) Two-dose MenACWY (4-6 weeks apart), N=10

Group 9: Adults aged 56-69 (n=1000, +/- 10%)

a1) Two dose ChAdOx1 nCoV-19 0.5mL (3.5 – 6.5 × 10^{10} vp, Abs 260, corrected for PS80)*, (4-6 weeks apart) N=500, OR

a2) Two dose MenACWY (4-6 weeks apart), N= 500

Group 10: Adults aged 70 years and above (n=1000, +/- 10%)

a1) Two dose ChAdOx1 nCoV-19 0.5mL (3.5 – 6.5 × 10^{10} vp, Abs 260, corrected for PS80)*, (4-6 weeks apart) N=500, OR

a2) Two dose MenACWY (4-6 weeks apart), N= 500

Group 11: Adults aged 18-55 who previously received a ChAdOx1 vectored vaccine (n=up to 60)

a1) Two dose ChAdOx1 nCoV-19 0.5mL (3.5 – 6.5 × 10^{10} vp, Abs 260, corrected for PS80)*, (4-6 weeks apart) N=up to 60

Group 12: HIV positive adults aged 18-55 (n=up to 60)

a1) Two dose ChAdOx1 nCoV-19 0.5mL (3.5 – 6.5 × 10^{10} vp, Abs 260, corrected for PS80)*, (4-6 weeks apart) N= up to 60

* See section 8.5 for further information on dosing

Visit Schedule : See schedule of attendances tables in section 7.3.3

Planned Trial Duration 12 months post last vaccination per participant

	Objective	Outcome Measure
**Primary**	To assess efficacy of the candidate ChAdOx1 nCoV-19 against COVID-19 in adults aged 18 years and older.	Virologically confirmed (PCR* positive) symptomatic cases of COVID-19
**Co-Primary**	To assess the safety of the candidate vaccine ChAdOx1 nCoV-19 in adults and children.	Occurrence of serious adverse events (SAEs) throughout the study duration.
**Secondary**	To assess the safety, tolerability and reactogenicity profile of the candidate vaccine ChAdOx1 nCoV-19	a) occurrence of solicited local reactogenicity signs and symptoms for 7 days following vaccination;<br>b) occurrence of solicited systemic reactogenicity signs and symptoms for 7 days following vaccination;<br>c) occurrence of unsolicited adverse events (AEs) for 28 days following vaccination ;<br>d) change from baseline for safety laboratory measures (except groups 4, 6, 9 and 10);<br>e) Occurrence of disease enhancement episodes

	To assess efficacy of the candidate ChAdOx1 nCoV-19 against severe and non-severe COVID-19	a) Hospital admissions associated with COVID-19<br>b) Intensive care unit (ICU) admissions associated with COVID-19<br>c) Deaths associated with COVID-19<br>d) Seroconversion against non-Spike SARS-CoV-2 antigens<br>e) Severe COVID-19 disease (defined according to clinical severity scales)
	To assess humoral immunogenicity of ChAdOx1 nCoV-19	a) Antibodies against SARS-CoV-2 spike protein (seroconversion rates) at Day 28 post-vaccination.<br>b) Proportion of seroconversion to antibodies against SARS-CoV-2 spike protein at Day 28 post-vaccination.
	To assess cellular immunity of ChAdOx1 nCoV-19 in older adults and in children (groups 1, 2, 3, 7 and 8 only)	a) Interferon-gamma (IFN-γ) enzyme-linked immunospot (ELISpot) responses to SARS-CoV-2 spike protein;
	To assess the safety and immunogenicity of a booster dose of ChAdOx1 nCoV-19 in older adults aged 56 years or older (two-dose	a) occurrence of solicited local reactogenicity signs and symptoms for 7 days following booster vaccination;

	schedules for groups 1, 2, 7 and 8 only)	b) occurrence of solicited systemic reactogenicity signs and symptoms for 7 days following booster vaccination;<br>c) occurrence of unsolicited adverse events (AEs) for 28 days following booster vaccination;<br>d) change from baseline and change from pre-booster for safety laboratory measures and;<br>e) Occurrence of disease enhancement episodes<br>f) Antibodies against SARS-CoV-2 spike protein at Day 56 post-vaccination.<br>g) Proportion of seroconversion to antibodies against SARS-CoV-2 spike protein at Day 56 post-vaccination
Tertiary	Exploratory Immunology	a) virus neutralising antibody (NAb) assays against live and/or pseudotype SARS-CoV-2 virus<br>b) Cell analysis by flow cytometry assays<br>c) Functional antibody assays<br>d) Anti-vector immunity induced by 1 or 2 doses of ChAdOx1 nCoV-19

	Measure exposure to COVID-19	Reported by weekly survey to collect information about cases amongst household contacts and friends, contact with the general public, infection control procedures
	Exploratory efficacy against infection<br>• To assess efficacy of the candidate ChAdOx1 nCoV-19 against SARS-CoV-2 infection	a) PCR* positive SARS-CoV-2 infection<br>b) Differences in viral loads between those with severe, mild, and asymptomatic PCR+* SARS-CoV-2 infections
	Compare safety, reactogenicity and immunogenicity between different manufacturing batches of ChAdOx1 nCoV-19 used in COV001 and COV002	a) Differences in safety, reactogenicity and immunogenicity profiles between Group 1 in COV001 and Group 5 in COV002 (proportion of Grade 3 solicited AEs, occurrence of fevers, seroconversion rates at D28, neutralising antibody titres and differences in T-cell responses at D14).
	Compare safety, reactogenicity and immunogenicity between different methods for measuring doses (Abs260, Abs 260 corrected for PS80 and qPCR) of ChAdOx1 nCoV-19	a) Differences in safety, reactogenicity and immunogenicity profiles between Groups 1, 2, and 5A compared with Groups, 7, 8, and 5B, C and D respectively (proportion of Grade 3 solicited AEs, occurrence of fevers, seroconversion rates at D28,

		neutralising antibody titres and differences in T-cell responses at D14).
	To assess vaccine induced mucosal immunity	Nasal mucosa IgA levels at D0 and D28 in a subset of individuals
	To compare viral shedding on stool samples of SARS-CoV-2 PCR* positive individuals	Differences in viral shedding on stool at 7 days and beyond post SARS-CoV-2 positivity.
	To compare immunogenicity of ChAdOx1 nCoV-19 in participants receiving 1 or 2 doses in groups 1, 2, 7 and 8	a) Differences in antibody titres (ELISA and Neutralising antibodies) in participants who received 1 or 2 doses of ChAdOx1 nCoV-19 (groups 1, 2, 7 and 8)<br>b) Longevity of immune responses in participants who received 1 or 2 doses of ChAdOx1 nCoV-19
	To describe the impact of previous vaccination with other ChAdOx1 vectored vaccines on safety and immune responses to ChAdOx1 nCoV-19	Differences reactogenicity profile, antibody titres and T-cell responses between groups 5d and 11 and their relationship with anti-vector neutralising antibody titres.
	To assess the cell-mediated and humoral immunogenicity profile of ChAdOx1 nCoV-19 vaccine in HIV infected adults	Cell-mediated and humoral responses against SARS-Cov-2 These will be measured by the following:

		a) Proportion of seroconversion to antibodies (Ab) against SARS-CoV-2 spike protein measured by ELISA.<br>b) Interferon-gamma enzyme linked immunospot (ELISpot) responses to SARS-CoV-2 spike protein<br>c) Intracellular Cytokine analyses of CD4 and CD8-specific SARS-CoV-2 spike protein responses<br>d) Further exploratory immunology
	To assess whether increasing age and or CD4 nadir are associated with a lack of immune response in HIV infected adults	a) relationship between nadir CD4 count and vaccine immune responses<br>b) relationship between age at enrolment and vaccine immune response<br>c) Immune responses to ChAdOx1 nCoV-19 (assessed as described above)
	To assess the safety of the candidate vaccine ChAdOx1 nCoV-19 in HIV infected adults	a) Occurrence of serious adverse events (SAEs) throughout the study duration<br>b) occurrence of solicited local reactogenicity signs and symptoms for 7 days following vaccination

		c) occurrence of solicited systemic signs and symptoms for 7 days following each vaccination<br>d) occurrence of unsolicited AEs for 28 days following each vaccination
	To assess Impact of vaccination on HIV reservoirs	Change in Total HIV DNA copies per million CD4 T cells
**Investigational products**	a) ChAdOx1 nCoV-19, a replication-deficient simian adenoviral vector expressing the spike (S) protein of SARS-CoV-2<br>b) MenACWY, Meningococcal Group A, C, W-135 and Y conjugate vaccine	

*or other nucleic acid amplification test (NAAT)

**Formulation**	ChAdOx1 nCoV-19: Aqueous solution for injection<br>MenACWY: powder and solvent for solution for injection
**Route of Administration**	Intramuscular (IM)
**Dose per Administration**	ChAdOx1 nCoV-19*:<br>- 2.2 x 10^{10} vp (qPCR)<br>- 2.5 x 10^{10} vp (qPCR)<br>- 5 x 10^{10} VP (Abs 260)<br>- 5 x 10^{10} VP (qPCR)<br>- 0.5mL (3.5 – 6.5 × 10^{10} vp, Abs 260, corrected for PS80)*

	Men ACWY: 0.5 mL

* See section 8.5 for further information on dosing

2 ABBREVIATIONS

**Abs 260**	Absorbance 260 nm
**AdHu**	Human adenovirus
**AdHu5**	Human adenovirus serotype 5
**AE**	Adverse event
**AID**	Autoimmune Disease
**CCVTM**	Centre for Clinical Vaccinology and Tropical Medicine, Oxford
**CBF**	Clinical BioManufacturing Facility
**CEF**	Chick embryo fibroblast
**ChAdOx**	Chimpanzee adenovirus 1
**CI**	Confidence interval
**COP**	Code of Practice
**CRF**	Case Report Form or Clinical Research Facility
**CTRG**	Clinical Trials & Research Governance Office, Oxford University
**CTL**	Cytotoxic T Lymphocyte
**DSUR**	Development Safety Update Report
**ELISPOT**	Enzyme-linked immunospot
**GCP**	Good Clinical Practice
**GMO**	Genetically modified organism
**GMT**	Geometric Mean Titre
**GP**	General Practitioner
**HCG**	Human Chorionic Gonadotrophin
**HEK**	Human embryonic kidney
**HIV**	Human Immunodeficiency virus
**HLA**	Human leukocyte antigen
**HRA**	Health Research Authority
**IB**	Investigator Brochure
**ICH**	*International Council for Harmonisation*
ICMJE	*International Committee of Medical Journal Editors*
ICS	*Intracellular Cytokine Staining*
**ID**	Intradermal
**IFNγ**	Interferon gamma
**IM**	Intramuscular
**IMP**	Investigational Medicinal Product
**IMP-D**	Investigational Medicinal Product Dossier
**IV**	Intravenous
**NAAT**	Nucleic acid amplification assay
**MenACWY**	Quadrivalent capsular group A, C, W and Y meningococcal protein-polysaccharide conjugate vaccine
**MHRA**	Medicines and Healthcare Products Regulatory Agency
**MVA**	Modified vaccinia virus Ankara
**NHS**	National Health Service
**NIH**	National Institutes of Health
**NIHR**	National Institute for Health Research
**PBMC**	*Peripheral blood mononuclear cell*

**PCR**	Polymerase chain reaction
**PI**	Principal Investigator
**PS80**	Polysorbate 80
**QP**	Qualified Person
**qPCR**	Quantitative polymerase chain reaction
**REC**	Research Ethics Committee
**SAE**	Serious adverse event
**SC**	Subcutaneous
**SmPc**	Summary of Product characteristics
**SOP**	Standard Operating Procedure
**SUSAR**	Suspected unexpected serious adverse reaction
**µg**	microgram
**Vp**	viral particle
**VV**	viral vector
**WHO**	World Health Organisation

3 BACKGROUND AND RATIONALE

3.1 Background

In December 2019, a cluster of patients with pneumonia of unknown cause was linked to a seafood wholesale market in Wuhan, China and were later confirmed to be infected with a novel coronavirus, known as 2019-nCoV [1]. The virus was subsequently renamed to SARS-CoV-2 because it is similar to the coronavirus responsible for severe acute respiratory syndrome (SARS-CoV), a lineage B betacoronavirus. SARS-CoV-2 shares more than 79% of its sequence with SARS-CoV, and 50% with the coronavirus responsible for Middle East respiratory syndrome (MERS-CoV), a member of the lineage C betacoronavirus[2]. COVID-19 is the infectious disease caused by SARS-CoV-2. By January 2020 there was increasing evidence of human to human transmission as the number of cases rapidly began to increase in China. Despite unprecedented containment measures adopted by the Chinese government, SARS-CoV-2 rapidly spread across the world. The WHO declared the COVID-19 outbreak a public health emergency of international concern on 30th January 2020. As of 26th May 2020, over 5, 584,091 cases have been reported with more than 349,894 deaths and 188 countries affected.

Coronaviruses (CoVs) are spherical, enveloped, large positive-sense single-stranded RNA genomes. One-fourth of their genome is responsible for coding structural proteins, such as the spike (S) glycoprotein, envelope (E), membrane (M) and nucleocapsid (N) proteins. E, M, and N are mainly responsible for virion assembly whilst the S protein is involved in receptor binding, mediating virus entry into host cells during CoVs infection via different receptors.[3] SARS-CoV-2 belongs to the phylogenetic lineage B of the genus *Betacoronavirus* and it recognises the angiotensin-converting enzyme 2 (ACE2) as the entry receptor [4]. It is the seventh CoV known to cause human infections and the third known to cause severe disease after SARS-CoV and MERS-CoV.

The spike protein is a type I, trimeric, transmembrane glycoprotein located at the surface of the viral envelope of CoVs, which can be divided into two functional subunits: the N-terminal S1 and the C-terminal S2. S1 and S2 are responsible for cellular receptor binding via the receptor binding domain (RBD) and fusion of virus and cell membranes respectively, thereby mediating the entry of SARS-CoV-2 into target cells.[3] The roles of S in receptor binding and

membrane fusion make it an ideal target for vaccine and antiviral development, as it is the main target for neutralising antibodies.

ChAdOx1 nCoV-19 vaccine consists of the replication-deficient simian adenovirus vector ChAdOx1, containing the structural surface glycoprotein (Spike protein) antigen of the SARS CoV-2 (nCoV-19), with a leading tissue plasminogen activator (tPA) signal sequence. ChAdOx1 nCoV-19 expresses a codon-optimised coding sequence for the Spike protein from genome sequence accession GenBank: MN908947. The tPA leader sequence has been shown to be beneficial in enhancing immunogenicity of another ChAdOx1 vectored CoV vaccine (ChAdOx1 MERS) [5].

3.2 Preclinical studies

Refer to the Investigator Brochure for most recent pre-clinical data update

3.2.1 Immunogenicity (Jenner Institute, unpublished)

Mice (balb/c and CD-1) were immunised with ChAdOx1 expressing SARS-CoV-2 Spike protein or green fluorescent protein (GFP). Spleens were harvested for assessment of IFY ELISpot responses and serum samples were taken for assessments of S1 and S2 antibody responses on ELISA at 9 or 10 days post vaccination. The results of this study show that a single dose of ChAdOx1 nCoV was immunogenic in mice.

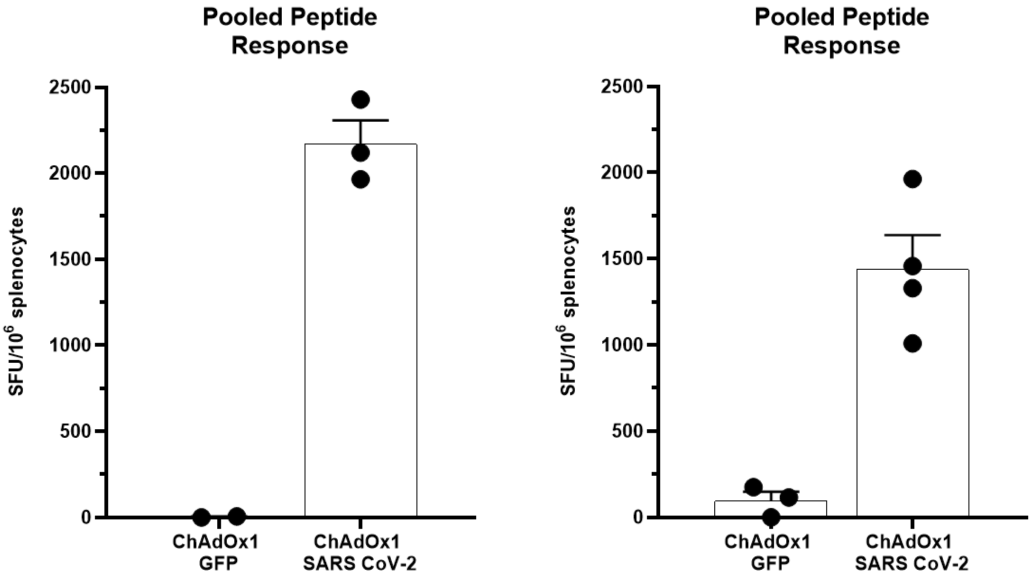

Figure 1. Summed splenic IFN-γ ELISpot responses of BALB/c (left panel) and CD-1 (right panel) mice, in response to peptides spanning the spike protein from SARS-CoV-2, nine or ten days post vaccination, with 1.7×10^{10} vp ChAdOx1 nCoV-19 or 8×10^{9} vp ChAdOx1 GFP. Mean with SEM are depicted

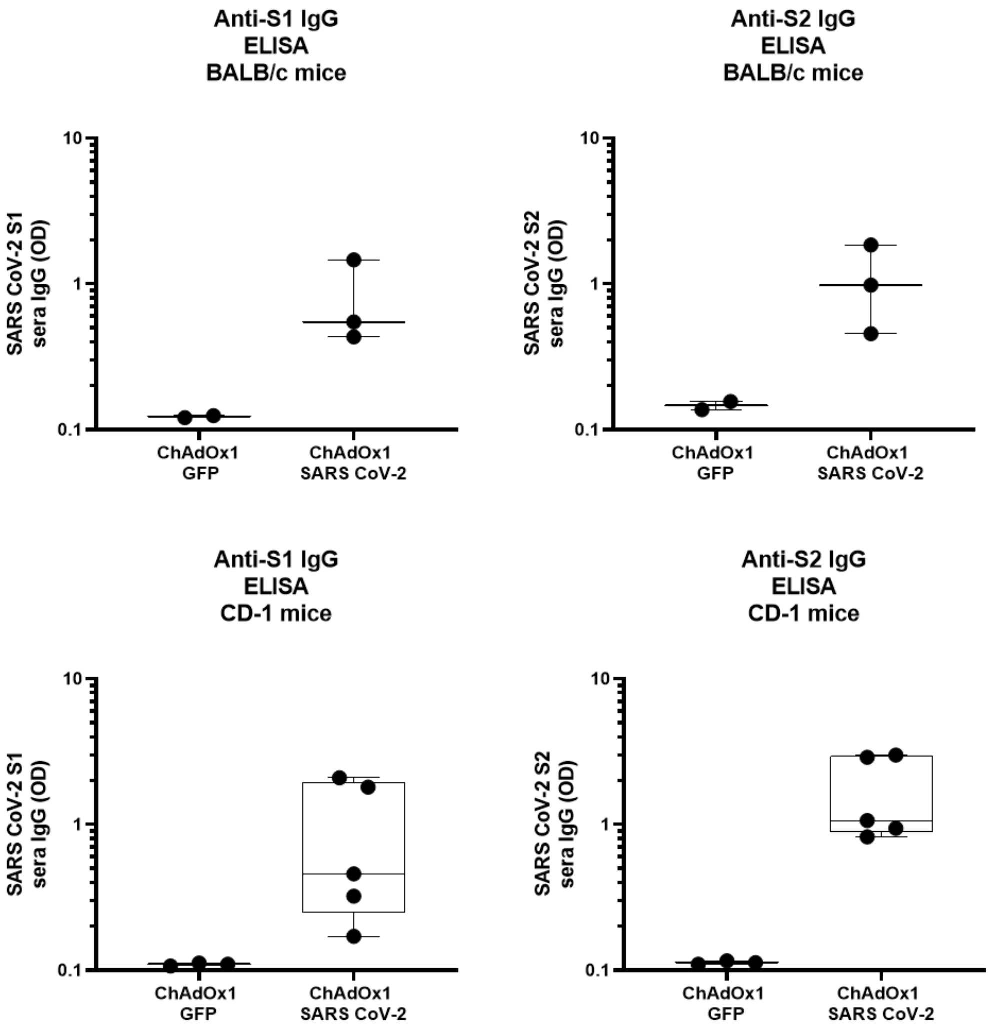

Figure 2. Box and whisker plot of the optical densities following ELISA analysis of BALB/C mouse sera (Top panel) incubated with purified protein spanning the S1 domain (left) or purified protein spanning the S2 domain (right) of the SARS-CoV-2 spike nine or ten days post vaccination, with 1.7×10^{10} vp ChAdOx1 nCoV-19 or 8×10^{9} vp ChAdOx1 GFP. Box and whisker plots of the optical densities following ELISA analysis of CD-1 mouse sera (Bottom panel) incubated with purified protein spanning the S1 domain (left) or purified protein spanning the S2 domain (right) of the SARS-CoV-2 spike.

Two mouse strains (BALB/c, N=5 and outbred CD1, N=8) were vaccinated intramuscularly (IM) with ChAdOx1 nCoV-19 or ChAdOx1 GFP, a control vaccine expressing green fluorescent protein. Humoral and cellular immunity were studied 9-14 days later. Total IgG titers were detected against spike protein subunits S1 and S2 in all vaccinated mice (Figure 3a). Profiling of the IgG subclasses showed a predominantly Th1 response post vaccination (Figure 4a). Virus-specific neutralising antibodies were detected in all mice vaccinated with ChAdOx1 nCoV-19, whereas no neutralisation was detected in serum from mice vaccinated with ChAdOx1 GFP (Figure 5b). Splenic T-cell responses measured by IFN-γ ELISpot and intracellular cytokine staining (ICS) were detected against peptides spanning the full length of the spike construct (Figure 3c). Again, a strong Th1-type response was detected post vaccination as supported by high levels of IFN-γ and TNF-α, and low levels of IL-4 and IL-10 (Figure 3d & Figure 4b-c).

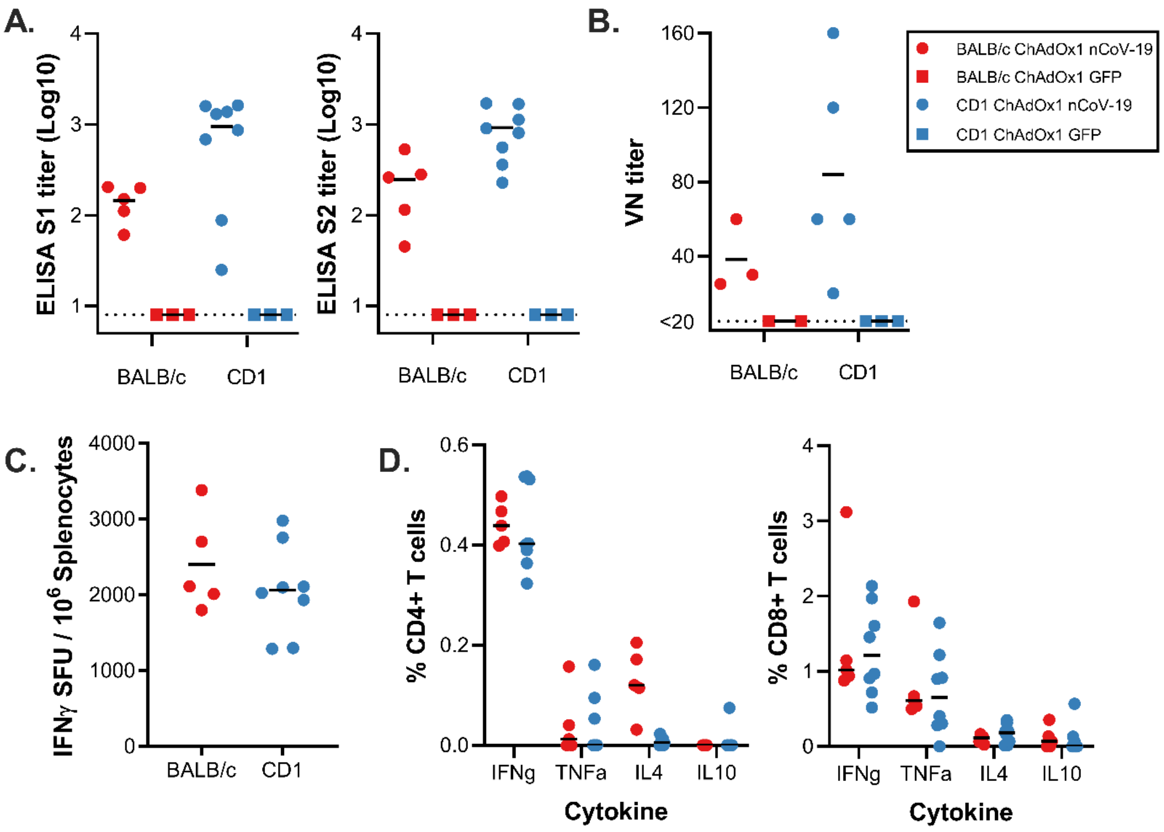

Figure 3: Humoral and cellular immune responses to ChAdOx1 58 nCoV-19 vaccination in mice. A). End point titer of serum IgG detected against S1 or S2 protein. Control mice were below the limit of detection. B). Virus neutralizing titer in serum. C). Summed IFN-γ ELISpot responses in splenocytes toward peptides spanning the spike protein. Control mice had low

(<100 SFU) or no detectable response. D). Summed frequency of spike-specific cytokine positive CD4+ or CD8+ T cells. BALB/c = red; CD1 = blue; vaccinated = circle; control = square; dotted line = limit of detection; line = mean; SFU = spot-forming units.

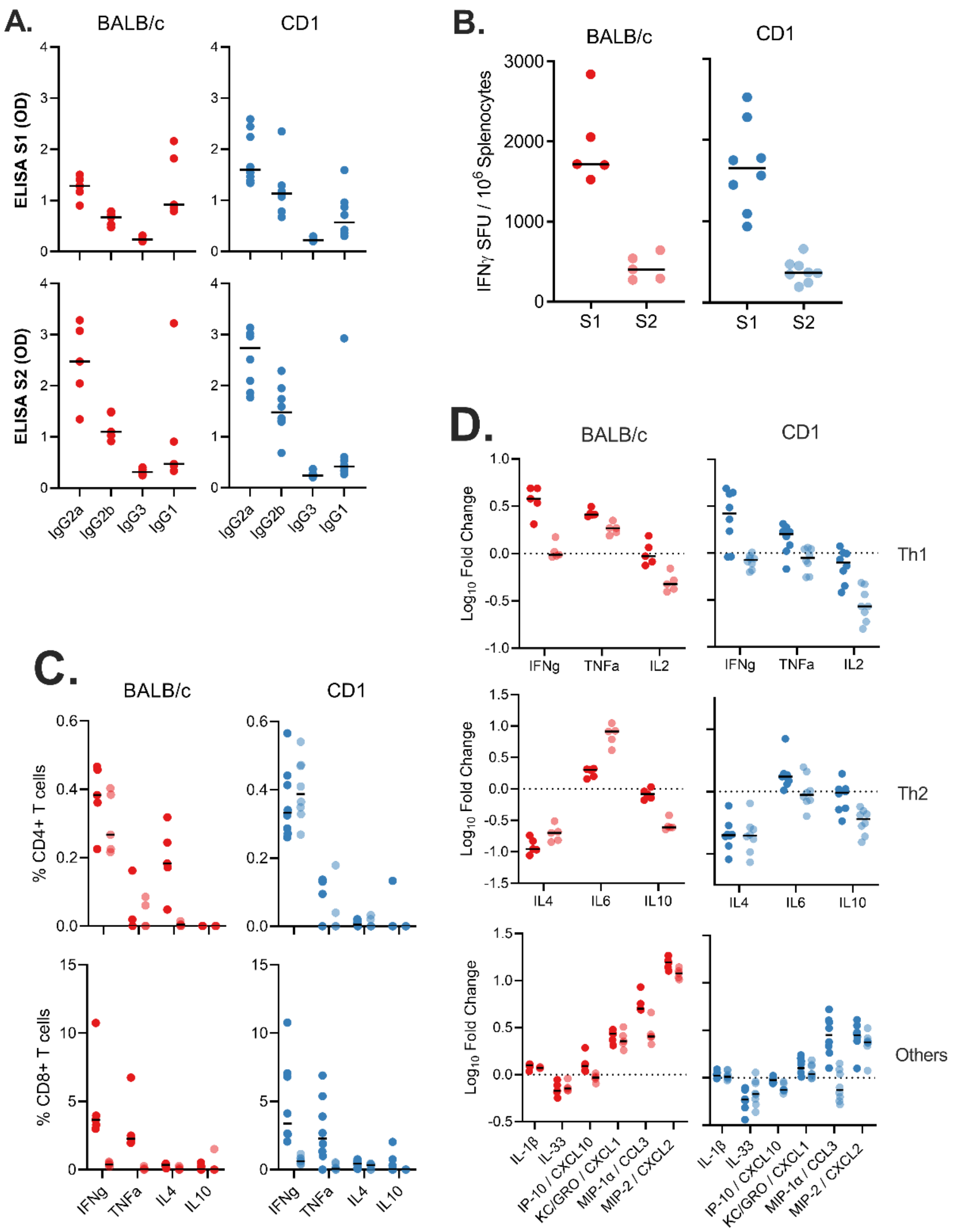

Figure 4. Antigen specific responses following ChAdOx1 nCov19 vaccination. A). IgG subclass antibodies detected against S1 or S2 protein in sera of BALB/c or CD1 mice. B).Frequency of cytokine positive CD4+ or CD8+ T cells following stimulation of splenocytes with S1 pool (dark) or S2 pool (transparent) peptides in BALB/c (red) and CD1 (blue) mice. C) Percentage of CD4+

or CD8+ T cells in BALB/c or CD1. D) Log10 fold change in cytokine levels in supernatant from S1 (dark) and S2 (transparent) stimulated splenocytes when compared to corresponding unstimulated 407 splenocyte sample for BALB/c and CD1 mice.

3.2.2 Non-human primate efficacy and immunogenicity – NIH (pre-print)

Details of this experiment are available at: https://www.biorxiv.org/content/10.1101/2020.05.13.093195v1 doi: https://doi.org/10.1101/2020.05.13.093195. In this study, two groups of rhesus macaques were utilized. Animals were adults, vaccinated group contained six animals, control group contained three animals. Group 1 was vaccinated with ChAdOx1 nCoV-19 at a dose of 2.5 x 10^{10} vp/animal at 28 days before challenge. Group 2 (control) was vaccinated with ChAdOx1 GFP at a dose of 2.5 x 10^{10} vp/animal at 28 days before challenge. The dose is half that which is planned for humans.

Animals were challenged with 2.6 x 10^{6} TCID¬50/animal of SARS-CoV-2 using 4 routes: intranasal (0.5ml per nostril), intratracheal (4ml), oral (1ml), and ocular (0.25ml per eye) of a 4x10^{5} TCID50/ml virus dilution in sterile DMEM.

Animals were examined on 1, 3, 5, and 7 days post challenge and will be euthanized at 7 days post challenge.

<u>Humoral response</u>

Antibodies in serum against SARS-CoV-2 spike protein were measured by ELISA. An increase in ELISA titer (Figure 5B) and neutralizing antibodies (Figure 5C) was found when comparing serum obtained before initial vaccination (-28), and at day of challenge (0).

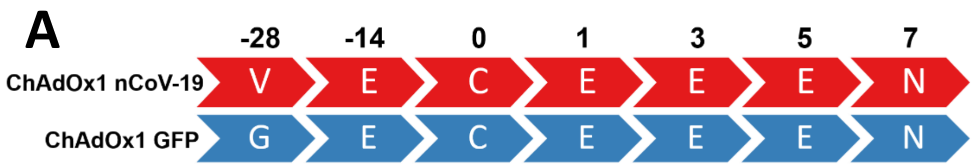

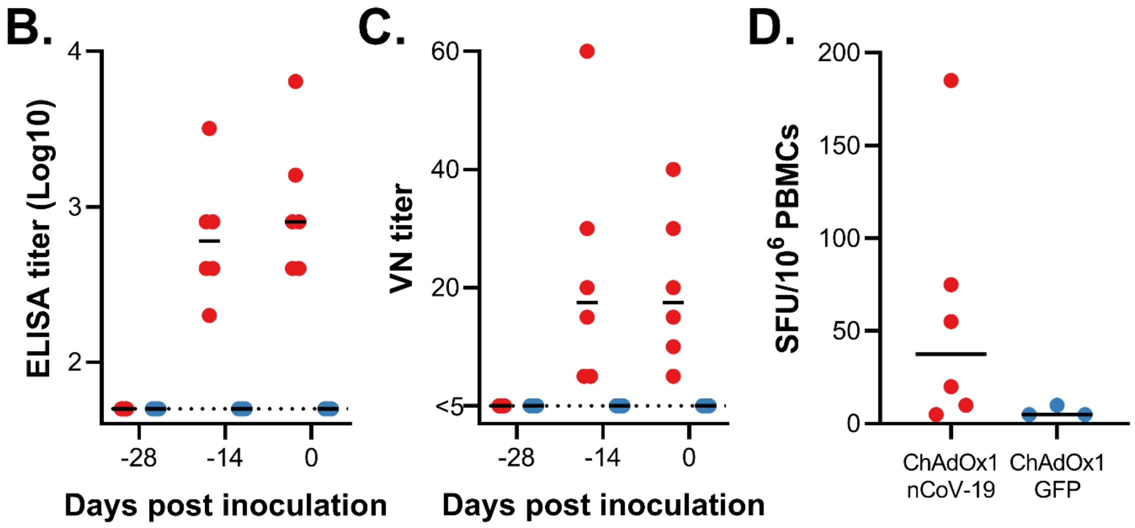

Figure 5. Humoral and cellular response to ChAdOx1 nCoV-19 vaccination in rhesus macaques. A. Study schedule for NHPs. V = vaccination with ChAdOx1 nCoV-19; G = vaccination with ChAdOx1 GFP; E = exam; N = necropsy. B. End point titre of serum IgG detected against S protein via ELISA at -28, -14 and 0 DPI. C. Two-fold serial-diluted serum samples were tested for neutralizing antibodies against SARS CoV-2 in VeroE6 cells at -28, -14 and 0 DPI. D. Summed S protein specific IFN-γ ELISpot responses. Vaccinated animals = red; control animals = blue; dotted line = limit of detection.

Cytokine response

Cytokines in serum were analysed after challenge to monitor immune responses. We observed an upregulation in IFN-γ at 1 DPI in ChAdOx1 nCoV-19 vaccinated animals, but not in control animals. No significant differences were observed between ChAdOx1 nCoV-19 and control animals for TNF-α, IL-2, IL-4, IL-6, and IL-10 (Figure 6).

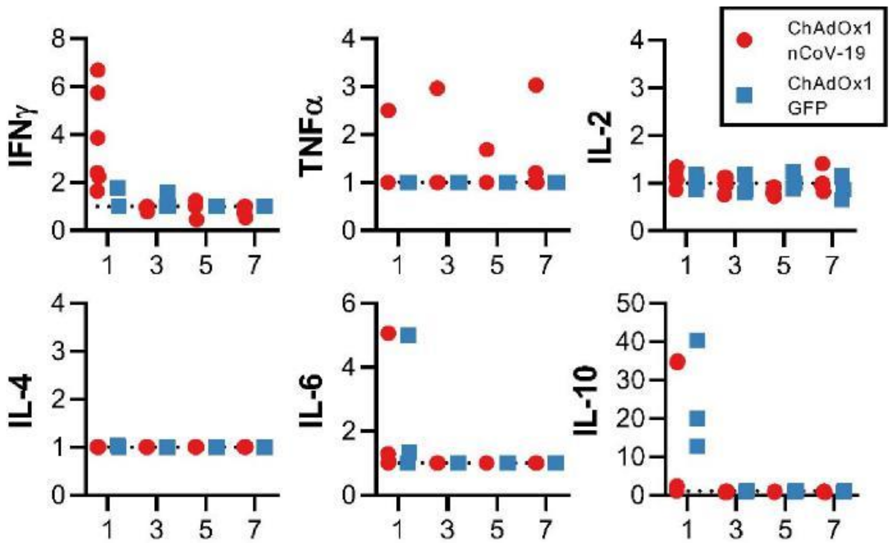

Figure 6. Serum cytokines in rhesus macaques challenged with SARS-CoV-2. Fold increase in cytokines in serum compared to 0 DPI values.

Shedding of virus

Viral gRNA load was high in lung tissue of control animals and viral sgRNA was detected in 2 out of 3 control animals (Figure 7d). In contrast, the viral gRNA load was significantly lower in lung tissue obtained from vaccinated animals as determined via Mann-Whitney's rank test and below limits of detection in two vaccinated animals. Viral sgRNA was detected in lung tissue obtained from 1 out of 6 vaccinated animals ($p<0.0001$, Figure 11d). Viral gRNA could be detected in other tissues but was low in both groups (Figure 8).

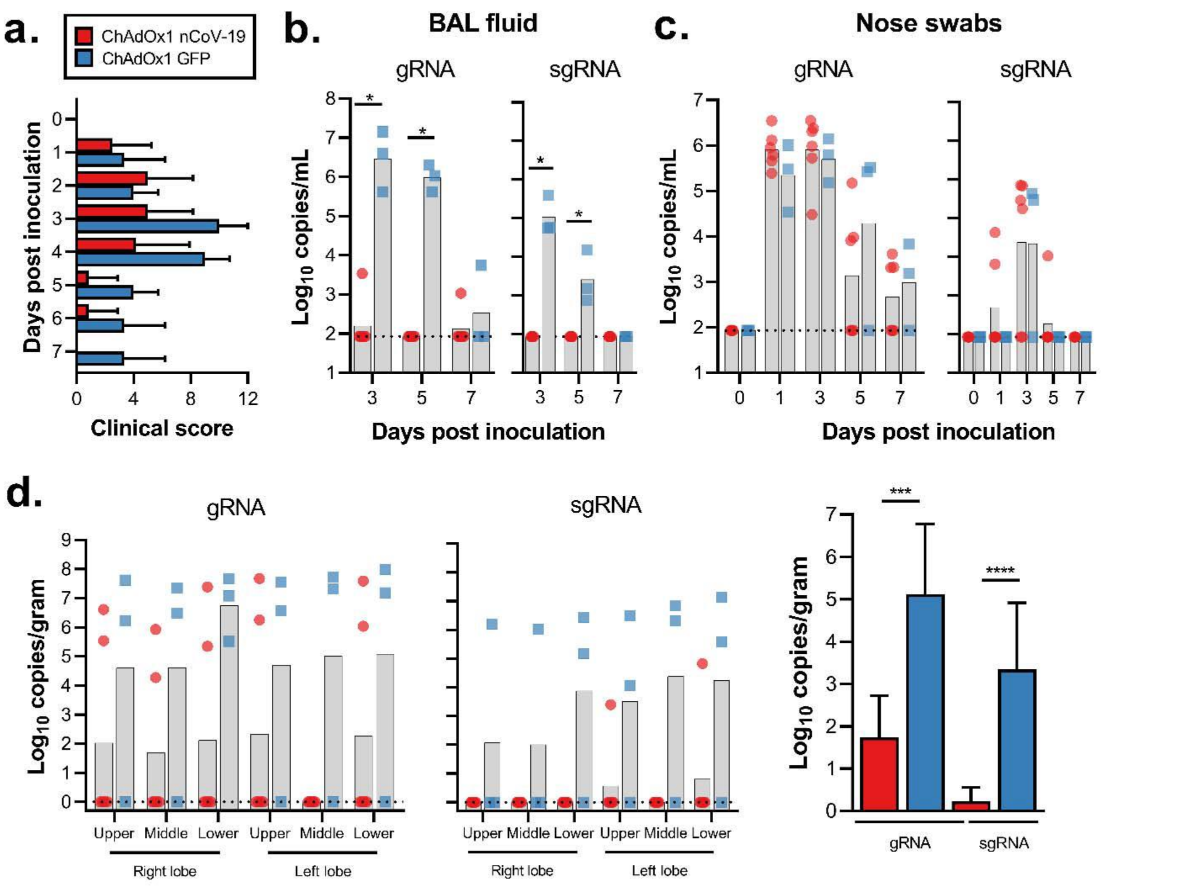

Figure 7. Clinical signs and viral load in rhesus macaques inoculated with SARS-CoV-2 after vaccination with ChAdOx1 nCoV-19. a. Mean clinical score with standard deviation in NHPs. Any scoring associated with food was removed from final score. b. Viral load in BAL fluid obtained from rhesus macaques, bar at geometric mean. *=p-value<0.0166. c. Viral load in nose swabs obtained from rhesus macaques, bar at geometric mean. d. Viral load in tissues at 7 DPI. Pictured are individual values with geometric mean bars (left panels) and geometric mean of all lung lobes per group (right panel). ***=p-value<0.001; ****=p-value<0.0001. Vaccinated animals = red circles; control animals = blue squares; dotted line = limit of detection.

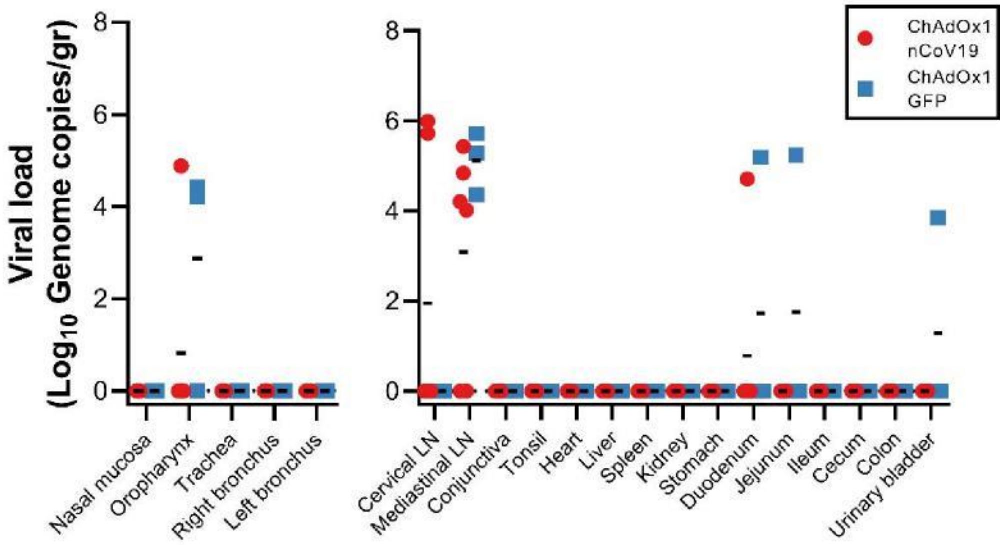

Figure 8. Viral load in rhesus macaques challenged with SARS-CoV-2. Viral genomic RNA in respiratory tissues excluding lung tissue (left panel) and other tissues (right panel). A two-tailed Mann-Whitney's rank test was performed to investigate statistical significance. Bonferroni correction was applied, and thus statistical significance was reached at p>0.0125.

Pulmonary pathology

At 7 days post inoculation, all animals were euthanized, and tissues were collected. None of the vaccinated monkeys developed pulmonary pathology after inoculation with SARS-CoV-2. All lungs were histologically normal and no evidence of viral pneumonia nor immune-enhanced inflammatory disease was observed. In addition, no SARS-CoV-2 antigen was detected by immunohistochemistry in the lungs of any of the vaccinated animals. Two out of 3 control animals developed some degree of viral interstitial pneumonia. Lesions were widely separated and characterized by thickening of alveolar septae by small amounts of edema fluid and few macrophages and lymphocytes. Alveoli contained small numbers of pulmonary macrophages and, rarely, edema. Type II pneumocyte hyperplasia was observed. Multifocally, perivascular infiltrates of small numbers of lymphocytes forming perivascular cuffs were observed. Immunohistochemistry demonstrated viral antigen in type I and II pneumocytes, as well as in alveolar macrophages (Figure 9.

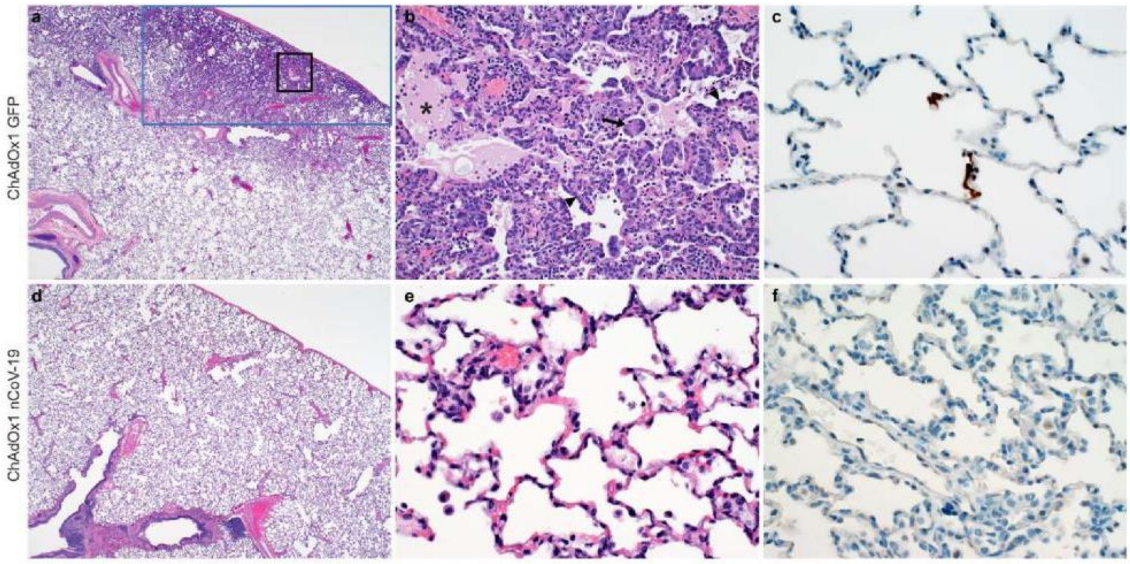

Figure 9 Histological changes in lungs of rhesus macaques on 7 dpi. a) Focal interstitial pneumonia in lungs of a control animal (blue box). The area in the black box is magnified in panel b. b) Interstitial pneumonia with edema (asterisk), type II pneumocyte hyperplasia (arrowhead) and syncytial cells (arrow) in control animals. c) SARS-CoV-2 antigen (visible as red-brown staining) was detected by immunohistochemistry in type I and type II pneumocytes in the lungs of control animals. d) No histological changes were observed in the lungs of ChadOx1 nCoV-19-vaccinated animals. e) Higher magnification of lung tissue in panel d. No evidence of pneumonia or immune-enhanced inflammation is observed. f) No SARS-CoV-2 antigen was detected by immunohistochemistry in the lungs of vaccinated animals. Magnification: panels a, d 40x; panels b, c, e, f 400x.

Further pre-clinical efficacy studies of ChAdOx1 nCoV-19 in ferrets and non-human primates are in progress. Results will be included in the Investigator's Brochure when available

3.2.3 Antibody Dependant Enhancement and Immunopathology

Safety concerns around the use of full length coronavirus Spike glycoproteins and other viral antigens (nucleoprotein) as a vaccine antigen have been raised following historical and limited reports of immunopathology and antibody dependant enhancement (ADE) reported *in vitro* and post SARS-CoV challenge in mice, ferrets and non-human primates immunised with whole SARS-CoV inactivated or full-length S protein based vaccines, including a study using Modified Vaccinia Ankara as a vector.[6-8] To date, there has been one report of lung immunopathology

following MERS-CoV challenge in mice immunised with an inactivated MERS-CoV candidate vaccine.[9] However, in preclinical studies of ChAdOx1 immunisation and MERS-CoV challenge, no ADE was observed in hDPP4 transgenic mice, dromedary camels or non-human primates (van Doremalen et al, manuscript submitted).[10,11]

The risks of inducing lung immunopathology in the event of COVID-19 disease following ChAdOx1 nCoV-19 vaccination are unknown. The NHP study conducted by NIH described above showed no evidence of immune-enhanced inflammation in ChAdOx1 nCoV-19 vaccinated animals who underwent SARS-CoV-2 challenge 4 weeks post immunisation, at 7 days post challenge. Results from a separate challenge study conducted on a purified inactivated SARS-CoV-2 vaccine also corroborate with NIH findings where no ADE has been detected in vaccinated animals [12]. However, the negative findings on ADE and lung immunopathology from both reports should be interpreted with caution, as challenged animals were sacrificed and examined shortly after challenge (7 days post inoculation). Further challenge studies on ChAdOx1 nCoV-19 vaccinated ferrets and NHPs with observation periods greater than 7 days after challenge are underway. These pre-clinical studies will report on presence or absence of lung pathology. Results will be reviewed as soon as they emerge and will inform discussions on risk/benefit to participants receiving the IMP. All pathology data arising from challenge studies of other SARS-CoV-2 vaccine candidates will also be taken into account.

3.3 Previous clinical experience

ChAdOx1 vectored vaccines expressing different inserts have previously been used in over 320 healthy volunteers taking part in clinical trials conducted by or in partnership with the University of Oxford in the UK and overseas (table 1 and 2). Most importantly, a ChAdOx1 vectored vaccine expressing the full-length Spike protein from another Betacoronavirus, MERS-CoV, has been given to 31 participants to date as part of MERS001 and MERS002 trials. ChAdOx1 MERS was given at doses ranging from $5x10^9$ vp to $5x10^{10}$ vp (table 2) with no serious adverse reactions reported. Further safety and immunogenicity results on ChAdOx1 MERS can be found on the Investigator's Brochure for ChAdOx1 nCoV-19 for reference.

Clinical trials of ChAdOx1 vectored vaccines encoding antigens for Influenza (fusion protein NP+M1), Tuberculosis (85A), Prostate Cancer (5T4), Malaria (LS2), Chikungunya (structural

polyprotein), Zika (prM and E), MERS-CoV (full-length Spike protein) and Meningitis B are listed below.

None of the below mentioned clinical trials reported serious adverse events associated with the administration of ChAdOx1, which was shown to have a good safety profile.

Table 1. Clinical experience with ChAdOx1 viral vector vaccines.

Country	Trial	Vaccine	Age	Route	Dose	Number of Volunteers (Received ChAdOx1)	Publication / Registration Number
UK	FLU004	ChAdOx1 NP+M1	18-50	IM	$5x10^{8}$ vp	3	Antrobus et al, 2014. Molecular Therapy. DOI: 10.1038/mt.2013.284 [13]
					$5x10^{9}$ vp	3	
					$2.5x10^{10}$ vp	3	
					$5x10^{10}$ vp	6	
UK	FLU005	ChAdOx1 NP+M1<br>MVA NP+M1 (week 8)	18-50	IM	$2.5x10^{10}$ vp	12	Coughlan et al, 2018. EBioMedicine DOI: 10.1016/j.ebiom.2018.02.011 DOI: 10.1016/j.ebiom.2018.05.001 [14]
		ChAdOx1 NP+M1<br>MVA NP+M1 (week 52)	18-50	IM	$2.5x10^{10}$ vp	12	
		MVA NP+M1<br>ChAdOx1 NP+M1 (week 8)	18-50	IM	$2.5x10^{10}$ vp	12	
		MVA NP+M1<br>ChAdOx1 NP+M1 (week 52)	18-50	IM	$2.5x10^{10}$ vp	9	
		ChAdOx1 NP+M1	>50	IM	$2.5x10^{10}$ vp	12	
		ChAdOx1 NP+M1<br>MVA NP+M1 (week 8)	>50	IM	$2.5x10^{10}$ vp	12	
UK	TB034	ChAdOx1 85A	18-50	IM	$5x10^{9}$ vp	6	Wilkie et al, 2020 Vaccine

Country	Trial	Vaccine	Age	Route	Dose	Number of Volunteers (Received ChAdOx1)	Publication / Registration Number
					2.5x10^{10} vp	12	DOI: 10.1016/j.vaccine.2019.10.102 [15]
		ChAdOx1 85A<br>MVA85A (week 8)	18-50	IM	2.5x10^{10} vp	12	
		ChAdOx1 85A<br>(x2, 4weeks apart)<br>MVA85A (at 4 months)	18-50	IM	2.5x10^{10} vp	12	
Switzerland	TB039<br>(ongoing)	ChAdOx1 85A	18-55	Aerosol	1x10^{9} vp	3	Clinicaltrials.gov:<br>NCT04121494
				Aerosol	5x10^{9} vp	3	
				Aerosol	1x10^{10} vp	11	
				Aerosol/IM	1x10^{10} vp	15	
Uganda	TB042<br>(ongoing)	ChAdOx1 85A	18-49	IM	5x10^{9} vp	6	Clinicaltrials.gov:<br>NCT03681860
					2.5 x10^{10}	6	
UK	VANCE01	ChAdOx1.5T4<br>MVA.5T4	18 – 75	IM	2.5x10^{10} vp	34	Clinicaltrials.gov:<br>NCT02390063
UK	ADVANCE	ChAdOx1.5T4	≥18	IM	2.5x10^{10} vp	23 (as of Feb 20)	Clinicaltrials.gov:

Country	Trial	Vaccine	Age	Route	Dose	Number of Volunteers (Received ChAdOx1)	Publication / Registration Number
	(ongoing)	MVA.5T4					NCT03815942
UK	VAC067	ChAdOx1 LS2	18-45	IM	$5x10^{9}$ vp<br>$2.5x10^{10}$ vp	3<br>10	Clinicaltrials.gov:<br>NCT03203421
UK	VAMBOX	ChAdOx1 MenB.1	18-50	IM	$2.5x10^{10}$ vp<br>$5x10^{10}$ vp	3<br>26	ISRCTN46336916
UK	CHIK001	ChAdOx1 Chik	18-50	IM	$5x10^{9}$ vp<br>$2.5x10^{10}$ vp<br>$5x10^{10}$ vp	6<br>9<br>9	Clinicaltrials.gov:<br>NCT03590392<br>DOI: https://doi.org/10.4269/ajtmh.abstract2019<br>Abstract #59, page 19.
UK	ZIKA001<br>(ongoing)	ChAdOx1 Zika	18-50	IM	$5x10^{9}$ vp<br>$2.5x10^{10}$ vp<br>$5x10^{10}$ vp	6<br>3 (as of Feb 20)<br>-	Clinicaltrials.gov:<br>NCT04015648

Table 2. Clinical experience with ChAdOx1 MERS

Country	Trial	Vaccine	Age	Route	Dose	Number of Volunteers (Received ChAdOx1)	Publication / Registration Number
UK	MERS001 (ongoing)	ChAdOx1 MERS	18-50	IM	$5x10^9$ vp<br>$2.5x10^{10}$ vp<br>$5x10^{10}$ vp<br>$2.5x10^{10}$ vp (homologous prime-boost)	6<br>9<br>9<br>3	Clinicaltrials.gov: NCT03399578<br>Folegatti et.al. 2020, Lancet Infect.Dis<br>DOI: https://doi.org/10.1016/S1473-3099(20)30160-2 [16]
Saudi Arabia	MERS002 (ongoing)	ChAdOx1 MERS	18-50	IM	$5x10^9$ vp<br>$2.5x10^{10}$ vp<br>$5x10^{10}$ vp	4<br>3<br>-	Clinicaltrials.gov: NCT04170829

3.4 Rationale

The COVID-19 epidemic has caused major disruption to healthcare systems with significant socioeconomic impacts. Containment measures have failed to stop the spread of virus, which has reached pandemic levels. There are currently no specific treatments available against COVID-19 and accelerated vaccine development is urgently needed.

Live attenuated viruses have historically been among the most immunogenic platforms available, as they have the capacity to present multiple antigens across the viral life cycle in their native conformations. However, manufacturing live-attenuated viruses requires complex containment and biosafety measures. Furthermore, live-attenuated viruses carry the risks of inadequate attenuation causing disseminated disease, particularly in immunocompromised hosts. Given that severe disease and fatal COVID-19 disproportionally affect older adults with co-morbidities, making a live-attenuated virus vaccine is a less viable option. Replication competent viral vectors could pose a similar threat for disseminated disease in the immuno-suppressed. Replication deficient vectors, however, avoid that risk while maintaining the advantages of native antigen presentation, elicitation of T cell immunity and the ability to express multiple antigens [17]. Subunit vaccines usually require the use of adjuvants and whilst DNA and RNA vaccines can offer manufacturing advantages, they are often poorly immunogenic requiring multiple doses, which is highly undesirable in the context of a pandemic.

Chimpanzee adenovirus vaccine vectors have been safely administered to thousands of people using a wide range of infectious disease targets. ChAdOx1 vectored vaccines have been given to over 320 volunteers with no safety concerns and have been shown to be highly immunogenic at single dose administration. Of relevance, a single dose of a ChAdOx1 vectored vaccine expressing full-length spike protein from another betacoronavirus (MERS-CoV) has shown to induce neutralising antibodies in recent clinical trials.

The use of an active comparator (MenACWY) will minimise the chances of accidental participant unblinding, decreasing bias in reactogenicity or safety reporting and/or health seeking behaviours once symptomatic for COVID-19.

The use of prophylactic paracetamol reduces the incidence and severity of fever and other adverse events following immunisation (AEFI). It has been previously recommended following Meningococcal B vaccine administration without negatively impacting its immunogenicity profile (reference: Bexsero

SmPC). Given the potential higher reactogenicity profile of ChAdOx1 nCoV-19 at $5x10^{10}$vp doses, a prophylactic paracetamol dose has been introduced in order to minimise severity of commonly observed local and systemic AEFI.

A batch comparison group (Group 5) has been included to assess potential differences in safety, reactogenicity and immunogenicity profiles across different ChAdOx1 nCoV-19 vaccine manufacturers.

Group 6 has been added to provide a comparison between efficacy at $5x10^{10}$vp dose on Abs260 and $5x10^{10}$ vp qPCR methods from different vaccine manufacturers.

Groups 7 and 8 have been added to provide safety, reactogenicity and immunogenicity data in older age groups receiving a $5x10^{10}$vp dose on qPCR, and replicate the study design in groups 1 and 2.

Group 4b has been added to provide immunogenicity data on homologous prime-boost at $5x10^{10}$vp (Abs260) prime and $2.2x10^{10}$vp (qPCR) boost, where up to 100 volunteers aged 18-55 initially recruited into group 4a will receive a booster dose of the vaccine 4-6 weeks apart.

Groups 4c and 6b have been added following interim immunogenicity results on homologous prime-boost groups showing improved neutralising antibody titres after 2 doses when compared to 1 dose regimen.

Groups 9 and 10 have been added as part of main safety and efficacy assessments in older age groups (56 – 69 years and 70 years and over) and removed from groups 4 and 6, as no vaccinations have been given to these age groups at the time of these group additions.

Group 11 has been added as an open-label and not randomised group to investigate the impact of previous ChAdOx1 vectored vaccines in immune responses elicited by ChAdOx1 nCoV-19.

Group 12 has been added as an open-label and not randomised group to investigate the safety and immunogenicity of ChAdOx1 nCoV-19 in people living with HIV.

3.4.1 Rationale for including older age groups

Deaths from COVID-19 infections are more common in adults aged 70 or older, and in those with pre-existing co-morbidities such as cardiovascular disease, diabetes, chronic respiratory disease, hypertension and cancer. SARS-CoV-2 infects children as well as adults and the elderly. However, COVID-19 infections in children are less severe and rarely result in death. It is the oldest age group

that is most at risk of death following natural infection, and in whom the vaccine would most likely be used first if deployed in a future public health campaign.

This study will recruit volunteers aged 56 to 70 years and those aged over 70 years. Simultaneously we will proceed to enrol a further up to 10,000 participants aged 18+ for a wide assessment of efficacy, with those over 55 years included in this larger cohort only as safety data become available from Group 1 and 2 cohorts.

3.4.2 Rationale for including younger age groups

ChAdOx1 vectored vaccines have not been administered to children before. However, ChAd63 – a closely related simian adenovirus vector- has been given to over 450 children and infants from 10 weeks old at doses ranging from 1 to 5 x 10^{10} vp, as part of malaria vaccine trials in Burkina Faso and West Africa [18,19]. Other adenoviral vectored vaccine (human adenovirus vector) expressing the RSV pre-fusion antigen (Ad26.RSV.pref) and the Ebola glycoprotein (Ad26.ZEBOV) has been previously administered to over 650 toddlers, children and adolescents at doses up to 5x10^{10}vp. None of these trials reported safety concerns with the use of adenoviral vectored vaccines in children. It is expected that ChAdOx1 nCoV-19 will have will have similar safety profile in paediatric groups.

Reports of COVID-19 amongst children are increasing and the majority of cases are considered to be milder or even asymptomatic [20-22]. Despite significantly lower case-fatality rates, severe presentations have been reported and at least 11 deaths recorded in England in paediatric groups (as of 30th April 2020, NHS England). Preliminary evidence suggests that children are just as likely to become infected with SARS-CoV-2 as adults but the importance of children in virus transmission remains uncertain [23]. Nonetheless, the WHO preferred target product profile for COVID-19 vaccines has all ages as target population, recognizing that herd immunity (and transmission blocking) will depend on broad immunization, likely including children (WHO, COVID-19 Vaccine TPP). In influenza, which has a similar age-dependent severity profile, universal vaccination of primary-school age children is an important strategy in UK immunisation policy to control disease through herd immunity.

We will, therefore, seek to enrol children aged 5-12 years old in order to obtain safety and immunogenicity data of ChAdOx1 nCoV-19 in paediatric groups. Given that a) immune responses in children aged 12-18 are unlikely to differ from those in adults; b) the comparator chosen in this study is routinely administered to children aged 13-15 in the UK; and c) the complexities around consent

procedures and contraception requirements, we have taken a pragmatic approach to limit the upper age to 12 years old. The lower age limit has been chosen based on safety concerns of febrile induced seizures following immunisation in younger children up to 5 years of age and potential for highest impact in curbing disease transmission in the community by prioritising school aged children.

[18,19]Since the benefit for children is lower than for adults in the population, we will accrue safety data from the phase I-III adult studies before embarking on the paediatric trial.

Recent reports of increased incidence in Kawasaki-like disease in children during the pandemic have raised concerns over the potential role for SARS-CoV-2 infection or its immune mediated response as a potential triggers [24]. The role of vaccine induced immune response against the SARS-CoV-2 in Kawasaki-like disease and other hyperinflammation syndromes is currently unknown. Kawasaki-like disease will be monitored and recorded as an adverse event of special interest.

3.4.3 Phase I/II study - COV001

The phase I/II study of efficacy, safety and immunogenicity of the ChAdOx1 nCoV19 vaccine (COV001, EudraCT 2020-001072-15) is the first evaluation of the vaccine in healthy adults aged 18-55 years in the UK started in April 2020. Over 1000 participants were enrolled and received either the investigational vaccine or a licensed MenACWY vaccine.

The two clinical studies are aligned in terms of study procedures and endpoints to allow data to be compared and combined across the two studies. The safety data from animal studies and from COV001 will be reviewed prior to vaccinating the first participant in COV002, and at each time point prior to expansion into additional age groups. See section 5 for further details.

3.4.4 Rationale for including HIV Infected persons

People living with HIV may have less functional immunity and have more associated co-morbidities than the general population. Indeed the chronic immune activation and inflammation observed in HIV-infected patients has been associated with poor antibody (Ab) responses to vaccines against influenza and HAV/HBV [25,26]. Evaluating immunological outcomes to the ChAdOx1 nCoV-19 vaccine allows us to assess whether responses are the same as in a matched HIV negative cohort, facilitating global policy on vaccine implementation in areas of high HIV prevalence

4 OBJECTIVES AND ENDPOINTS

	Objective	Outcome Measure	Timepoint of evaluation
**Primary**	To assess efficacy of the candidate ChAdOx1 nCoV-19 against COVID-19 in adults aged 18 years and older.	Virologically confirmed (PCR* positive) symptomatic cases of COVID-19	Throughout the study
**Co-Primary**	To assess the safety of the candidate vaccine ChAdOx1 nCoV-19 in adults and children.	Occurrence of serious adverse events (SAEs) throughout the study duration.	Throughout the study
**Secondary**	To assess the safety, tolerability and reactogenicity profile of the candidate vaccine ChAdOx1 nCoV-19	a) occurrence of solicited local reactogenicity signs and symptoms for 7 days following vaccination;<br>b) occurrence of solicited systemic reactogenicity signs and symptoms for 7 days following vaccination;<br>c) occurrence of unsolicited adverse events (AEs) for 28 days following vaccination ;<br>d) change from baseline for safety laboratory measures and (except groups 4, 6, 9 and 10);<br>e) Occurrence of disease enhancement episodes	a) Day 0-7 Self-reported symptoms recorded using electronic diaries<br>b) Day 0-7 Self-reported symptoms recorded using electronic diaries<br>c) Day 0-28 Self-reported symptoms recorded using electronic diaries<br>d) See schedule of attendances<br>e) Throughout the study

	To assess efficacy of the candidate ChAdOx1 nCoV-19 against severe and non-severe COVID-19	a) Hospital admissions associated with COVID-19<br>b) Intensive care unit (ICU) admissions associated with COVID-19<br>c) Deaths associated with COVID-19<br>d) Seroconversion against non-Spike SARS-CoV-2 antigens<br>e) Severe COVID-19 disease (defined according to clinical severity scales)	Throughout the study<br><br>See schedule of attendances<br><br>Throughout the study
	To assess humoral immunogenicity of ChAdOx1 nCoV-19	a) Antibodies against SARS-CoV-2 spike protein at Day 28 post-vaccination.<br>b) Proportion of seroconversion to antibodies against SARS-CoV-2 spike protein measured by ELISA at Day 28 post-vaccination.	Blood samples drawn at Day 0 and Day 28 post-vaccination
	To assess cellular immunity of ChAdOx1 nCoV-19 in older adults and in children (groups 1, 2, 3, 7 and 8 only)	a) Interferon-gamma (IFN-γ) enzyme-linked immunospot (ELISpot) responses to SARS-CoV-2 spike protein;	See schedule of attendances
	To assess the safety and immunogenicity of a booster dose of ChAdOx1 nCoV-19 in older adults aged 56 years or older (two-dose	a) occurrence of solicited local reactogenicity signs and symptoms for 7 days following booster vaccination;	a) Day 28-35 Self-reported symptoms recorded using electronic diaries

	schedules for groups 1, 2, 7 and 8 only)	b) occurrence of solicited systemic reactogenicity signs and symptoms for 7 days following booster vaccination;<br>c) occurrence of unsolicited adverse events (AEs) for 28 days following booster vaccination;<br>d) change from pre-booster for safety laboratory measures and;<br>e) Occurrence of disease enhancement episodes<br>f) Antibodies against SARS-CoV-2 spike protein at Day 56 post-vaccination.<br>g) Proportion of seroconversion to antibodies against SARS-CoV-2 spike protein from baseline at Day 56 post-vaccination.	b) Day 28-35 Self-reported symptoms recorded using electronic diaries<br>c) Day 28-56 Self-reported symptoms recorded using electronic diaries<br>d) See schedule of attendances<br>e) Throughout the study<br>f) Blood samples drawn at day 0, 28 and at day 56.<br>g) Blood samples drawn at day 0, 28 and at day 56.
Tertiary	Exploratory Immunology	a) virus neutralising antibody (NAb) assays against live and/or pseudotype SARS-CoV-2 virus<br>b) Cell analysis by flow cytometry assays<br>c) Functional antibody assays<br>d) Anti-vector immunity induced by 1 or 2 doses of ChAdOx1 nCoV-19	See schedule of attendances

	Exploratory efficacy against infection<br>• To assess efficacy of the candidate ChAdOx1 nCoV-19 against SARS-CoV-2 infection	a) PCR* positive SARS-CoV-2 asymptomatic infection<br>b) Differences in viral loads between those with severe, mild, and asymptomatic PCR+* SARS-CoV-2 infections.	Throughout the study
	Measure exposure to COVID-19	Reported by weekly survey to collect information about cases amongst household contacts and friends, contact with the general public, infection control procedures	Weekly throughout the study
	Compare safety, reactogenicity and immunogenicity between different manufacturing batches of ChAdOx1 nCoV-19 used in COV001 and COV002	Differences in safety, reactogenicity and immunogenicity profiles between Group 1 in COV001 and Group 5 in COV002 (proportion of Grade 3 solicited AEs, occurrence of fevers, seroconversion rates, neutralising antibody titres and differences in T-cell responses.	Day 0-7 for solicited AEs<br><br>D28 for seroconversion rates and neutralising antibodies<br><br>D14 for T-cell immunology readouts
	Compare safety, reactogenicity and immunogenicity between different methods for measuring doses (Abs260, Abs 260 corrected for PS80, and qPCR) of ChAdOx1 nCoV-19	Differences in safety, reactogenicity and immunogenicity profiles between Groups 1, 2, and 5A compared with and Groups, 7, 8 and 5B, C and D respectively (proportion of Grade 3 solicited AEs, occurrence of fevers, seroconversion	Day 0-7 for solicited AEs<br><br>D28 for seroconversion rates and neutralising antibodies

		rates at D28, neutralising antibody titres and differences in T-cell responses at D14).	D14 for T-cell immunology readouts
	To assess vaccine induced mucosal immunity	Differences in IgA levels in nasal mucosa in a subset of individuals	at D0 and D28 post vaccination
	To compare viral shedding on stool samples of SARS-CoV-2 PCR* positive individuals	Differences in viral shedding on stool between vaccine and comparator arms	At approximately 7 days and beyond post SARS-CoV-2 PCR* positivity.
	To compare immunogenicity of ChAdOx1 nCoV-19 in participants receiving 1 or 2 doses (groups 1, 2, 7 and 8)	a) Differences in antibody titres (ELISA and Neutralising antibodies) in participants who received 1 or 2 doses of ChAdOx1 nCoV-19 (groups 1, 2, 7 and 8)<br>b) Longevity of immune responses in participants who received 1 or 2 doses of ChAdOx1 nCoV-19 (groups 1, 2, 7 and 8)	a) At 28 days post prime and 28 days post boost<br>b) At 6 and 12 months post prime (prime only) and 6 and 12 months post boost
	To describe the impact of previous vaccination with other ChAdOx1 vectored vaccines in immune responses to ChAdOx1 nCoV-19	Differences in antibody titres and T-cell responses between groups 5d and 11 and their relationship with anti-vector neutralising antibody titres.	At day 14 post ChAdOx1 nCoV-19 prime (T-cell responses), day 28 post prime and day 28 post boost.

	To assess the cell-mediated and humoral immunogenicity profile of ChAdOx1 nCoV-19 vaccine in HIV infected adults	Cell-mediated and humoral responses against SARS-Cov-2 These will be measured by the following:<br>a) Proportion of seroconversion to antibodies (Ab) against SARS-CoV-2 spike protein measured by ELISA.<br>b) Interferon-gamma enzyme linked immunospot (ELISpot) responses to SARS-CoV-2 spike protein<br>c) Intracellular Cytokine analyses of CD4 and CD8-specific SARS-CoV-2 spike protein responses<br>d) Further exploratory immunology	a) at all exploratory immunology timepoints described in the schedule of attendances<br>b) at all exploratory immunology timepoints described in the schedule of attendances<br>c) at all exploratory immunology timepoints described in the schedule of attendances<br>d) at all exploratory immunology timepoints described in the schedule of attendances

	To assess whether increasing age and or CD4 nadir are associated with a lack of immune response in HIV infected adults	a) Nadir CD4 count<br>b) Age at enrolment<br>c) Immune responses to ChAdOx1 nCoV-19 (assessed as described above)	a) at all exploratory immunology timepoints described in the schedule of attendances
	To assess the safety of the candidate vaccine ChAdOx1 nCoV-19 in HIV infected adults	a) Occurrence of serious adverse events (SAEs) throughout the study duration<br>b) occurrence of solicited local reactogenicity signs and symptoms for 7 days following vaccination<br>c) occurrence of solicited systemic signs and symptoms for 7 days following each vaccination<br>d) occurrence of unsolicited AEs for 28 days following each vaccination	a) Throughout the study<br>b) Day 0-7 post prime and boost<br>c) Day 0-7 post prime and boost<br>d) Day 0-28 post prime and boost
	To assess Impact of vaccination on HIV reservoirs	Change in Total HIV DNA copies per million CD4 T cells	a) Throughout the study

*or other nucleic acid amplification test

Sample analysis for the completion of exploratory endpoints may be performed under the ethically approved OVC Biobank protocol.

5 TRIAL DESIGN

This is a Phase 2/3, participant-blinded individually randomised controlled trial in adults and healthy children in the UK, administering either a single dose or two-doses of ChAdOx1 nCoV-19 or licensed MenACWY vaccine via IM injection. Additional steps may be taken to keep clinical investigators assessing the primary efficacy endpoint blinded to group allocation, where this is possible and practical to do so. All data from participants with PCR (or other nucleic acid amplification test) - positive swabs will be assessed for inclusion in the primary efficacy analysis by two blinded assessors who will independently review each case according to pre-specified criteria as detailed in the statistical analysis plan, to classify each for inclusion in the primary and secondary outcomes.

After review of all available data from animal studies and at least 4 weeks safety and immunogenicity data from the first 54 participants receiving ChAdOx1 nCoV-19 in COV001, following DSMB review, enrolment into Groups 1, 4, 5 & 6 will commence. A minimum of 7 days safety data from group 1 will be reviewed by the DSMB prior to enrolment of participants into group 2. Participants will be randomised to ChAdOx1 nCoV-19/MenACWY on a 3:1:3:1 ratio in groups 1 and 7, and 5:1:5:1 ratio in groups 2 and 8, 1:1 in groups 3, 4, 5a, 5b, 5c, 6, 9 and 10, and 5:1 in group 5d. Participants in groups 4, 6, 9 and 10 will be advised to take prophylactic paracetamol for 24 hours (1000 mg every 4-6 hours) from the time of vaccination to reduce the likelihood of fever. The sequence of enrolment of participants over the age of 56 years is outlined in section 7.3.2.2. Recruitment into group 3 will not start before a minimum of 4 weeks safety and immunogenicity data from all healthy adult volunteers recruited into COV001 and all participants in groups 1 and 2 and 5, from COV002 are reviewed. Staggered enrolment will apply to group 3 with an interim review after 15 participants have received the IMP (half of the total number of participants expected to receive the IMP in this group). Up to 100 volunteers in group 4 will be invited to receive a booster dose of $2.2x10^{10}$vp (qPCR) 4-6 weeks after prime. All remaining volunteers in group 4 and all participants in group 6 will be invited to receive a booster dose of 0.5mL ($3.5 – 6.5 \times 10^{10}$ vp, Abs 260, corrected for PS80) at least 4 weeks after prime. Participants who were originally randomised to receive a single dose in groups 1 (a1 and a2), 2 (a1 and a2) and 5 (a1 and a2) will be invited to receive a booster dose of 0.5mL ($3.5 – 6.5 \times 10^{10}$ vp, Abs 260, corrected for PS80) at the earliest available opportunity, with a minimum 4 weeks interval from prime.

Safety will be assessed in real time. The DSMB will periodically assess safety and efficacy data every 4-8 weeks and/or as required.

Participants will be followed over the duration of the study to record adverse events and episodes of virologically confirmed symptomatic COVID-19 cases. Participants will be tested for COVID-19 if they present with a new onset of fever (≥37.8 C) OR cough OR shortness of breath OR anosmia/ageusia.

Weekly testing for PCR+ infection with SARS-CoV-2 using home test kits will also be undertaken in partnership with the Department of Health and Social care national community testing programme, subject to testing resource availability.

Moderate and Severe COVID-19 disease will be defined using clinical criteria. Detailed clinical parameters will be collected from medical records and aligned with agreed definitions as they emerge. These are likely to include, but are not limited to, oxygen saturation, need for oxygen therapy, respiratory rate and other vital signs, need for ventilatory support, Xray and CT scan imaging and blood test results, amongst other clinically relevant parameters.

Accumulated safety data from COV001 will be reviewed before commencing enrolment.

To account for the multisite recruitment activity, it is recognised that the number of volunteers enrolled into each group 9 and 10 will be 1000 +/-10%.

HIV -Group 12: open-label

This is a single arm group whereby up to 60 HIV infected individuals who are stable on antiretroviral therapy (ARV) will be recruited and receive ChAdOx1 nCoV-19 vaccination according to the schedule of attendance described in table 14.

5.1 Study groups

**Group**	**Vaccine**	**Number of Volunteers**	**Age group of volunteers**
**Randomised groups**			
Group 1 ****	a1) Single dose ChAdOx1 nCOV19 vaccine, 5x1010vp (Abs 260)*, OR<br>a2) Single dose MenACWY<br>a3) Two-dose ChAdOx1 nCoV-19 5x1010vp (Abs 260) prime and 0.5mL	N=30<br><br>N=10	Adults aged 56 – 69 years

	(3.5 – 6.5 × 1010 vp, Abs 260, corrected for PS80) boost*, OR<br>a4) Two-dose MenACWY,<br>b1) Two dose ChAdOx1 nCOV19 vaccine, 5x1010vp (Abs 260) prime and 2.2x1010vp (qPCR) boost* (4-6 weeks apart), OR<br>b2) Two-dose MenACWY (4-6 weeks apart)	N= up to 30 from 1a1<br>N= up to 10 from 1a2<br>N=30<br>N=10	
Group 2****	a1) Single dose ChAdOx1 nCOV19 vaccine, 5x1010vp (Abs 260)*, OR<br>a2) Single dose MenACWY (4-6 weeks apart)<br>a3) Two-dose ChAdOx1 nCoV-19 5x1010vp (Abs 260) prime and 0.5mL (3.5 – 6.5 × 1010 vp, Abs 260, corrected for PS80) boost*, OR<br>a4) Two-dose MenACWY<br>b1) Two dose ChAdOx1 nCOV19 vaccine, 5x1010vp (Abs 260) prime and 2.2x1010vp (qPCR) boost * (4-6 weeks apart), OR<br>b2) Two-dose MenACWY	N=50<br>N=10<br>N= up to 50 from 2a1<br>N= up to 10 from 2a2<br>N=50<br>N=10	Adults aged 70 years or older
Group 3	Single low-dose ChAdOx1 nCoV19 vaccine, 2.5×10^{10}vp (qPCR)* , OR<br>MenACWY	N=30<br>N=30	Children aged 5 to 12 years (inclusive)
Group 4**<br>(n= up to 3550)	a1) Single dose ChAdOx1 nCoV19 vaccine, 5×10^{10}vp (Abs 260)*OR<br>a2) MenACWY	N=up to 1775<br>N=up to 1775	Adults aged 18 – 55 years

	b1) Two dose ChAdOx1 nCOV19 vaccine, $5x10^{10}$vp (Abs260) prime and $2.2x10^{10}$vp (qPCR) boost* (4-6 weeks apart) OR<br><br>b2) Two dose MenACWY<br><br>c1) Two dose ChAdOx1 nCOV19 vaccine, $5x10^{10}$vp (Abs260) prime and 0.5mL (3.5 – 6.5 × 10^{10} vp, Abs 260, corrected for PS80) boost* OR $5x10^{10}$vp (qPCR) boost (at least 4 weeks apart) OR<br><br>c2) Two dose MenACWY	N= up to 50 (from 4a1)<br><br>N= up to 50 (from 4a2)<br><br>N= up to 1725 (from 4a1)<br><br>N= up to 1725 (from 4a2)	
Group 5****	a1) Single dose ChAdOx1 nCoV19 vaccine, $5x10^{10}$vp, (Abs 260)* OR<br>a2) MenACWY<br><br>a3) Two-dose ChAdOx1 nCoV-19 5x1010vp (Abs 260) prime and 0.5mL (3.5 – 6.5 × 1010 vp, Abs 260, corrected for PS80) boost*<br>a4) Two-dose MenACWY<br><br>b1) Single dose ChAdOx1 nCoV19 vaccine, $5x10^{10}$vp, (qPCR)* OR<br><br>b2) Men ACWY MenACWY<br>(B-cell immunology only)<br><br>c1) Single dose ChAdOx1 nCoV19 vaccine, $5x10^{10}$vp, (qPCR)* OR<br><br>c2) MenACWY<br>(B and T-cell immunology)	N= 50<br><br>N=50<br><br>N = up to 50 from 5a1<br><br>N = up to 50 from 5a2<br><br>N= up to 25<br><br>N= up to 25<br><br>N= up to 25<br><br>N= up to 25	Adults aged 18-55 years

	d1) Two-dose ChAdOx1 nCoV19 vaccine, 0.5mL (3.5 – 6.5 × 10^{10} vp, Abs 260, corrected for PS80)*, (4-6 weeks apart) OR<br>d2) Men ACWY	N= up to 50<br><br>N= up to 10	
Group 6***<br><br>(n= up to 6000)	a1) ChAdOx1 nCoV19 vaccine, 5x10^{10}vp (qPCR)* OR<br><br>a2) MenACWY<br><br>b1) Two dose ChAdOx1 nCoV-19 vaccine, 5x10^{10}vp (qPCR) prime and 0.5mL (3.5 – 6.5 × 10^{10} vp, Abs 260, corrected for PS80) boost* OR 5x10^{10}vp (qPCR) boost* (at least 4 weeks apart) OR<br><br>b2) Two dose MenACWY	N = up to 3000<br><br>N = up to 3000<br><br>N = up to 3000 (from 6a1)<br><br>N = up to 3000 (from 6a2)	Adults aged 18 – 55 years
Group 7	a1) Single dose ChAdOx1nCOV19 vaccine, 5x10^{10}vp (qPCR)*, OR<br><br>a2) Single dose MenACWY<br><br>b1) Two dose ChAdOx1nCOV19 vaccine, 5x10^{10}vp (qPCR)* (4-6 weeks apart), OR<br><br>b2) Two-dose MenACWY (4-6 weeks apart)	N=30<br><br>N=10<br><br>N=30<br><br>N=10	Adults aged 56 – 69 years
Group 8	a1) Single dose ChAdOx1nCOV19 vaccine, 5x10^{10}vp (qPCR)*, OR<br><br>a2) Single dose MenACWY<br><br>b1) Two dose ChAdOx1nCOV19 vaccine, 5x10^{10}vp (qPCR) prime and 0.5mL (3.5 –	N=50<br><br>N=10<br><br>N=50	Adults aged 70 years or older

	6.5 × 10^{10} vp, Abs 260, corrected for PS80) boost* OR 5x10^{10}vp (qPCR) boost (4-6 weeks apart), OR<br>b2) Two-dose MenACWY (4-6 weeks apart)	N=10	
Group 9	a1)Two dose ChAdOx1 nCOV19 vaccine, 0.5mL (3.5 – 6.5 × 10^{10} vp, Abs 260, corrected for PS80)* (4-6 weeks apart) OR<br>a2) Two dose MenACWY	N= approx. 500<br><br>N= approx. 500	Adults aged 56 – 69 years
Group 10	a1)Two dose ChAdOx1 nCOV19 vaccine, 0.5mL (3.5 – 6.5 × 10^{10} vp, Abs 260, corrected for PS80)* (4-6 weeks apart) OR<br>a2) Two dose MenACWY	N= approx. 500<br><br>N= approx. 500	Adults aged 70 years or older
Group 11	Two dose ChAdOx1 nCOV19 vaccine, 0.5mL (3.5 – 6.5 × 10^{10} vp, Abs 260, corrected for PS80)* (4-6 weeks apart)	N=up to 60	Adults aged 18-55 who previously received a ChAdOx1 vectored vaccine.
Group 12	Two dose ChAdOx1 nCOV19 vaccine, 0.5mL (3.5 – 6.5 × 10^{10} vp, Abs 260, corrected for PS80)* (4-6 weeks apart)	N=up to 60	HIV positive adults aged 18-55

* See section 8.5 for further information on dosing

** A subset of up to 100 participants in group 4a will be invited to receive a booster dose in 4b, keeping the overall sample size in group 4 the same. All remaining participants in group 4a will be invited to receive a booster dose in 4c, keeping the overall sample size in group 4 the same.

*** Participants in group 6a will be invited to receive a booster dose in 6b, keeping the overall sample size in group 6 the same

**** Participants in groups 1a (a1 and a2), 2a (a1 and a2) and 5a (a1 and a2) will be invited to receive a booster dose in the respective a3 and a4 groups, keeping the overall sample size in group 1a, 2a and 5a the same.

5.2 Trial volunteers

Adult volunteers aged at least 18 years, and healthy children aged 5 – 12 years (inclusive) will be recruited into the study. Volunteers will be considered enrolled immediately following administration of the vaccine.

5.3 Definition of End of Trial

The end of the trial is the date of the last assay conducted on the last sample collected.

5.4 Potential Risks for volunteers

The potential risks are those associated with phlebotomy, vaccination and disease enhancement

Venepuncture

Adult Groups

Localised bruising and discomfort can occur at the site of venepuncture. Infrequently fainting may occur. These will not be documented as AEs if they occur. The total volume of blood drawn over a 12 month period will be 105-621.5mL in the adult groups (blood volumes may vary slightly for volunteers at different investigator sites due to use of different volume vacutainers, following local Trust SOPs). The total volume of blood drawn over a 12 month period in the HIV group will be 1077.5mL. This should not compromise these otherwise healthy volunteers, as they would donate 470mL during a single blood donation for the National Blood transfusion Service over a 3-4 month period. Volunteers will be asked to refrain from blood donation for the duration of their involvement in the trial.

Group 3

In the paediatric group maximum blood volumes per visit are based on 0.8ml/kg. This is in line with guidance given by the European Commission of Public Health which are tabled below in table 3. The weights for each age group are based on the 0.4th centile on the female UK-WHO growth chart. These volumes should not compromise these otherwise healthy paediatric participants to ensure < 3% total blood volume sampling over a 3 month period.

Table 3 Paediatric maximum blood volumes per visit based on guidance by European Commission of public health

Age	Maximum (target) volume of blood (ml)
5-7 years	10ml
8-11 years	15ml
12 years	20ml

Allergic reactions

Allergic reactions from mild to severe may occur in response to any constituent of a medicinal product's preparation. Anaphylaxis is extremely rare (about 1 in 1,000,000 vaccine doses) but can occur in response to any vaccine or medication.

Vaccination

Local reaction from IM vaccination

The typical local reaction as a result of IM injection is temporary pain, tenderness, redness, and swelling at the site of the injection.

Systemic reactions

Constitutional influenza-like symptoms such as fatigue, headache, malaise, feverishness, and muscle aches can occur with any vaccination and last for approximately 2-3 days. In the phase 1 COV001 study, approximately 30-40% of participants not taking prophylactic paracetamol felt feverishness, or had chills, muscle ache, malaise, fatigue, or headache which they rated as moderate to severe. (See the investigator brochure for further details). Presyncopal and syncopal episodes may occur at the time of vaccination which rapidly resolve. As with many vaccines, temporary ascending paralysis (Guillain-Barré syndrome, GBS) or immune mediated reactions that can lead to organ damage may occur, but this should be extremely rare (1 in 100,000-1,000,000 vaccine doses).

Transient neutropenia, lymphopenia and thrombocytopenia has been described following immunization with other adenoviral-vectored vaccines, and is not perceived to be of clinical significance.

Control participants will receive one or two doses of a licensed MenACWY vaccine, the risks of which are described in these vaccines SmPC.

Disease Enhancement

The risks of inducing disease enhancement and lung immunopathology in the event of COVID-19 disease following ChAdOx1 nCoV-19 vaccination are unknown as described above. Two NHP challenge studies have shown no evidence of disease enhancement from immunisation with ChAdOx1 nCoV-19 or inactivated SARS-CoV-2 virus, but caution should be taken when interpreting these negative findings. . All pre-clinical data from challenge studies using ChAdOx1 nCoV-19 and other vaccine candidates (when available) will inform decisions on risk/benefit to participants receiving the IMP. Any safety signals associated with disease enhancement potentially observed in COV001 will also inform these decisions.

5.5 Known Potential Benefits

Volunteers enrolled into the control groups will receive 1 or 2 doses of MenACWY, a licensed vaccine that has been administered to teenagers in the UK routine schedule since 2015 and is used as a travel vaccine for high risk areas. The majority of participants in this study will not have had this vaccine previously, and therefore will gain the benefit of protection against group A, C, W and Y meningococcus. Those participants who have previously had MenACWY vaccines will have their immunity against these organisms boosted. Recipients of ChAdOx1 nCoV-19 do not have any guaranteed benefit, however it is hoped that the information gained from this study will contribute to the development of a safe and effective vaccine against COVID-19.

6 RECRUITMENT AND WITHDRAWAL OF TRIAL VOLUNTEERS

6.1 Identification of Trial Volunteers

Volunteers will be recruited by use of an advertisement +/- registration form formally approved by the ethics committee(s) and distributed or posted in the following places:

- In public places, including buses and trains, with the agreement of the owner / proprietor.
- In newspapers or other literature for circulation.
- On radio via announcements.

- On a website or social media site operated by our group or with the agreement of the owner or operator (including on-line recruitment through our website).
- By e-mail distribution to a group or list only with the express agreement of the network administrator or with equivalent authorisation.
- By email distribution to individuals who have already expressed an interest in taking part in any clinical trial at the Oxford Vaccine Centre and at trial sites.
- On stalls or stands at exhibitions or fairs.
- Via presentations (e.g. presentations at lectures or invited seminars).
- Direct mail-out: This will involve obtaining names and addresses of adults via the most recent Electoral Roll. The contact details of individuals who have indicated that they do not wish to receive postal mail-shots would be removed prior to the investigators being given this information. The company providing this service is registered under the General Data Protection Regulation 2016/679. Investigators would not be given dates of birth or ages of individuals but the list supplied would only contain names of those aged ≥18 years (as per the inclusion criteria).
- Direct mail-out using National Health Service databases: These include the National Health Applications and Infrastructure Services (NHAIS) via a NHAIS data extract or equivalent. Initial contact to potential participants will not be made by the study team. Instead study invitation material will be sent out on our behalf by an external company, CFH Docmail Ltd, in order to preserve the confidentiality of potential participants. CFH Docmail Ltd is accredited as having exceeded standards under the NHS Digital Data Security and Protection Toolkit (ODS ID – 8HN70).
- Oxford Vaccine Centre databases and study site databases: We may contact individuals from databases of groups within the CCVTM (including the Oxford Vaccine Centre database) and other study sites of previous trial participants who have expressed an interest in receiving information about all future studies for which they may be eligible.
- Using local GP practices or Trusts as Participant Identification Centres (PICs)

Recruitment of those with likely higher exposure to SARS-CoV-2 will be prioritised, in order to increase the likelihood of obtaining efficacy endpoints in the context of a waning epidemic. These

priority groups will mainly consist of, but are not limited to, COVID-19 patient facing frontline healthcare workers (e.g. those working in ICU, A&E, COVID-19 wards, Paramedics, Care Homes, GP COVID-19 hubs, dentists, COVID-19 testing centres), non-healthcare staff working in COVID-19 clinical areas (e.g. hospital porters, receptionists, cleaners, other hospital workers), and other public facing keyworkers with no access to personal protective equipment, amongst others.

6.2 Informed consent

The parent/legal guardian of the participant or the participant themselves (when aged 18 or over) will personally sign and date the latest approved version of the Informed Consent form. A written version and verbal explanation of the Study Information leaflet and Informed Consent will be presented to the participant/parent/legal guardian of the participant detailing:

• the exact nature of the study

• what it will involve for the participant

• the implications and constraints of the protocol

• the known side effects and any risks involved in taking part

• sample handling – participants will be informed that anonymised samples taken during the course of the study may be shared with study collaborators.

• Individual results will not be shared with participants

The Study Information leaflet will be made available to the participant and/or parent/legal guardian for an appropriate amount of time (where possible this will a minimum of 24 hours) prior to consent being obtained. A video presentation of the Study Information leaflet may be screened to an audience, or made available for them to access it remotely. However, participants will have the opportunity to individually question an appropriately trained and delegated researcher before signing consent. Assent will also be sought from children 7 years of age or older, to participate in the trial.

The following general principles will be emphasised:

- Participation in the study is entirely voluntary
- Refusal to participate involves no penalty or loss of medical benefits
- The volunteer may withdraw from the study at any time.

- The volunteer is free to ask questions at any time to allow him or her to understand the purpose of the study and the procedures involved
- The study involves research of an investigational vaccine
- There is no direct benefit to the volunteer from participating
- The volunteer's GP will be contacted to corroborate their medical history (Groups 1, 2 , 7 and 8 only, except group 12 where GPs can be replaced by their HIV consultant). Written or verbal information regarding the volunteer's medical history will be sought from the GP or other sources. This can either be via the study team accessing patient's electronic care summaries from local systems, by contacting the GP practice, or volunteers bringing their medical care summaries from the GP to the study clinicians. However, volunteers in all remaining groups may be enrolled based on medical information obtained during screening and/or enrolment visit, at the physician's discretion..
- Blood samples taken as part of the study may be sent outside of the UK and Europe to laboratories in collaboration with the University of Oxford. These will be de-identified. Volunteers will be asked if they consent to indefinite storage of any leftover samples for use in other ethically approved research, this will be optional.

The parent/legal guardian of the participant or adult participant will be allowed as much time as wish to consider the information, and the opportunity to question the Investigator, their GP or other independent parties to decide whether they will participate in the study. Written informed consent will then be obtained by means of the adult participant or the parent/legal guardian of the participant dated signature, and dated signature of the person who presented and obtained the Informed consent. The person who obtained the consent must be suitably qualified and experienced, and have been authorised to do so by the Chief/Principal Investigator and listed on the delegation log. A copy of the signed informed consent will be given to the participant or parent/legal guardian of the participant. The original signed form will be retained at the research study site, in the case report form (CRF).

Updated information that require volunteers to be re-consented will be sent to participants and written re-consent requested at the earliest scheduled visit. If the earliest visit to occur is in the symptomatic pathway, the participant may consent using an electronic signature for infection control purposes. Where appropriate, and when re-consenting in person is not possible (e.g. participants in self-isolation), volunteers may be contacted over the phone and an appropriately trained and

delegated researcher will obtain re-consent. In this instance the re-consent discussion will be documented by the researcher, the participant will sign the form (electronic or paper) and a copy will be signed by the researcher. The dates of signature may be different and a fully signed copy will be provided to the participant at the next scheduled visit. The participant may re-consent using an electronic signature.

6.3 Inclusion and exclusion criteria

This study will be conducted in adults and children, who meet the following inclusion and exclusion criteria:

6.3.1 Inclusion Criteria

The volunteer must satisfy all the following criteria to be eligible for the study:

- Adults aged 18 - 55 years (groups 4, 5, 6 and 11)
- Adults aged 56-69 years (groups 1, 7, and 9)
- Adults aged 70 years and older (groups 2, 8, and 10)
- Children aged 5-12 years inclusive (group 3)
- Able and willing (in the Investigator's opinion) to comply with all study requirements.
- Willing to allow the investigators to discuss the volunteer's medical history with their General Practitioner and access all medical records when relevant to study procedures.
- For females of childbearing potential only, willingness to practice continuous effective contraception (see below) during the study and a negative pregnancy test on the day(s) of screening and vaccination.
- Agreement to refrain from blood donation during the course of the study.
- Provide written informed consent.
- Parent/Guardian provides informed consent

Additional Inclusion criteria to Group 12 (HIV sub-study):

- HIV positive
- Receiving antiretroviral therapy

- Undetectable HIV viral load
- CD4>350 cells/mL

6.3.2 Exclusion Criteria

The volunteer may not enter the study if any of the following apply:

- Participation in COVID-19 prophylactic drug trials for the duration of the study.

  Note: Participation in COVID-19 treatment trials is allowed in the event of hospitalisation due to COVID-19. The COV002 study team should be informed as soon as possible.

- Participation in SARS-CoV-2 serological surveys where participants are informed of their serostatus for the duration of the study.

  Note: Disclosure of serostatus post enrolment may accidently unblind participants to group allocation. Participation in COV002 can only be allowed if volunteers are kept blinded to their serology results from local/national serological surveys

- Receipt of any vaccine (licensed or investigational) other than the study intervention within 30 days before and after each study vaccination, with the exception of the licensed seasonal influenza vaccination and the licensed pneumococcal vaccination. Participants will be encouraged to receive these vaccinations at least 7 days before or after their study vaccine.
- Prior or planned receipt of an investigational or licensed vaccine or product likely to impact on interpretation of the trial data (e.g. Adenovirus vectored vaccines, any coronavirus vaccines). This exclusion criteria will not apply to group 11, as recruitment will be targeted at those volunteers who previously received a ChAdOx1 vectored vaccine.
- Administration of immunoglobulins and/or any blood products within the three months preceding the planned administration of the vaccine candidate.
- Any confirmed or suspected immunosuppressive or immunodeficient state (except group 12, where HIV infected participants are allowed); asplenia; recurrent severe infections and use of immunosuppressant medication within the past 6 months, except topical steroids or short-term oral steroids (course lasting ≤14 days)
- History of allergic disease or reactions likely to be exacerbated by any component of ChAdOx1 nCoV-19 or MenACWY
- Any history of angioedema.

- Any history of anaphylaxis.
- Pregnancy, lactation or willingness/intention to become pregnant during the study.
- Current diagnosis of or treatment for cancer (except basal cell carcinoma of the skin and cervical carcinoma in situ).
- History of serious psychiatric condition likely to affect participation in the study.
- Bleeding disorder (e.g. factor deficiency, coagulopathy or platelet disorder), or prior history of significant bleeding or bruising following IM injections or venepuncture.
- Continuous use of anticoagulants, such as coumarins and related anticoagulants (i.e. warfarin) or novel oral anticoagulants (i.e. apixaban, rivaroxaban, dabigatran and edoxaban)
- Suspected or known current alcohol or drug dependency.
- Any other significant disease, disorder or finding which may significantly increase the risk to the volunteer because of participation in the study, affect the ability of the volunteer to participate in the study or impair interpretation of the study data.
- Severe and/or uncontrolled cardiovascular disease, respiratory disease, gastrointestinal disease, liver disease, renal disease, endocrine disorder and neurological illness (mild/moderate well controlled comorbidities are allowed)
- History of laboratory confirmed COVID-19 (except groups 5d, 9, 10 and 11).

o Seropositivity to SARS-CoV-2 before enrolment (except groups 5d, 9, 10 and 11)

o NB: volunteers with previous NAAT positive results are also allowed in groups 9, 10 and 11

Additional Exclusion criteria to Groups 4, 6, 9 and 10

- History of allergic disease or reactions likely to be exacerbated by Paracetamol

o Note: Caution should be taken when recommending paracetamol to adults who already take paracetamol chronically

Additional Exclusion Criteria to Group 3

- Chronic medical conditions such as chronic lung disease, chronic liver disease, chronic renal failure, chronic heart disease, congenital genetic syndromes (e.g. Trisomy 21)
- Fulfil any of the contraindications to vaccination as specified in The Green Book

6.3.3 Re-vaccination exclusion criteria (two-dose groups only)

The following AEs associated with any vaccine, or identified on or before the day of vaccination constitute absolute contraindications to further administration of an IMP to the volunteer in question. If any of these events occur during the study, the subject will not be eligible to receive a booster dose and will be followed up by the clinical team or their GP until resolution or stabilisation of the event:

- Anaphylactic reaction following administration of vaccine
- Pregnancy
- Any AE that in the opinion of the Investigator may affect the safety of the participant or the interpretation of the study results

Participants who develop COVID-19 symptoms and have a positive NAAT test after the first vaccination can only receive a booster dose after a minimum 4 weeks interval from their first NAAT positive test, provided their symptoms have significantly improved. The decision to proceed with booster vaccinations in those cases will be at clinical discretion of the investigators. For participants who are asymptomatic and have a positive NAAT test, a minimum of 2 weeks from first NAAT positivity will be required before boosting.

6.3.4 Effective contraception for female volunteers

Female volunteers of childbearing potential are required to use an effective form of contraception during the course of the study.

Acceptable forms of contraception for female volunteers include:

- Established use of oral, injected or implanted hormonal methods of contraception.
- Placement of an intrauterine device (IUD) or intrauterine system (IUS).
- Total hysterectomy.
- Bilateral Tubal Occlusion
- Barrier methods of contraception (condom or occlusive cap with spermicide).
- Male sterilisation, if the vasectomised partner is the sole partner for the subject.

- True abstinence, when this is in line with the preferred and usual lifestyle of the subject (Periodic abstinence and withdrawal are not acceptable methods of contraception).

6.3.5 Withdrawal of Volunteers

In accordance with the principles of the current revision of the Declaration of Helsinki and any other applicable regulations, a volunteer has the right to withdraw from the study at any time and for any reason, and is not obliged to give his or her reasons for doing so. The Investigator may withdraw the volunteer at any time in the interests of the volunteer's health and well-being. In addition, the volunteer may withdraw/be withdrawn for any of the following reasons:

- Administrative decision by the Investigator.
- Ineligibility (either arising during the study or retrospectively, having been overlooked at screening).
- Significant protocol deviation.
- Volunteer non-compliance with study requirements.
- An AE, which requires discontinuation of the study involvement or results in inability to continue to comply with study procedures.

The reason for withdrawal will be recorded in the CRF. If withdrawal is due to an AE, appropriate follow-up visits or medical care will be arranged, with the agreement of the volunteer, until the AE has resolved, stabilised or a non-trial related causality has been assigned. Any volunteer who is withdrawn from the study may be replaced, if that is possible within the specified time frame. The DSMB or DSMB chair may recommend withdrawal of volunteers.

If a volunteer withdraws from the study, data and blood samples collected before their withdrawal will still be used on the analysis. Storage of blood samples will continue unless the participant specifically requests otherwise.

In all cases of subject withdrawal, long-term safety data collection, including some procedures such as safety bloods, will continue as appropriate if subjects have received one or more vaccine doses, unless they decline any further follow-up.

6.4 Pregnancy

Should a volunteer become pregnant during the trial, no further study IMP will be administered. She will be followed up for clinical safety assessment with her ongoing consent and in addition will be followed until pregnancy outcome is determined. We would not routinely perform venepuncture in a pregnant volunteer unless there is clinical need.

7 CLINICAL PROCEDURES

This section describes the clinical procedures for evaluating study participants and follow-up after administration of study vaccine.

7.1 Schedule of Attendance

All volunteers will have clinic attendances and procedures as indicated in the schedule of attendances below (tables 5-12). Subjects will receive either the ChAdOx1 nCoV-19 vaccine or MenACWY, and undergo follow-up for a total of 12 months from the last vaccination visit. Additional visits or procedures may be performed at the discretion of the investigators, e.g., further medical history and physical examination, or additional blood tests and other investigations if clinically relevant.

7.2 Observations, medical history and physical examination

Temperature will be routinely measured at the time-points indicated in the schedule of procedures. Respiratory rate, oxygen saturation, pulse, blood pressure and temperature will be measured at the COVID-19 testing visits and if clinically required. All subjects will undergo medical history and a targeted physical examination if considered necessary at screening or pre-enrolment on D0. The purpose of this examination is to assess and document the subject's baseline health status so that any later change can be determined. Vital signs (temperature, heart rate, respiratory rate, blood pressure +/- oxygen saturation) will be measured at screening or pre-enrolment on D0 as part of baseline assessments. Further medical history, physical examination and observations may be done throughout the study based on clinical discretion. A targeted physical examination, including neurological assessment, must be conducted, when appropriate, in the event of a SAE.

Blood tests, Nose/Throat Swabs, Saliva samples and urinalysis

Blood will be drawn for the following laboratory tests and processed at contractually agreed NHS Trust laboratories using NHS standard procedures:

- **Haematology;** Full Blood Count
- **Biochemistry;** Sodium, Potassium, Urea, Creatinine, Albumin, Liver Function Tests (ALT, ALP, Bilirubin)
- **Diagnostic serology;** HBsAg, HCV antibodies, HIV antibodies in groups 1, 2, 5a, 5b, 5c and 5d, 7 and 8 only (specific consent will be gained prior to testing blood for these blood-borne viruses). HBsAg and HCV antibodies will be in group 12 with HIV antibodies only done at the investigators discretion.
- **Immunology;** Human Leukocyte Antigen (HLA) typing (groups 5a, 5b, 5c only)
- **COVID-19;** A nose/throat swab and/or saliva sample will be taken for COVID-19 NAAT testing
- **CD4 count and HIV viral load;** volunteers in group 12 only, before enrolment.

Additional safety blood tests may be performed if clinically relevant at the discretion of the medically qualified investigators, including potential prognostic indicators or markers of severe COVID-19 disease

At University of Oxford research laboratories or at designated specialist laboratories:

- **Immunology**; Immunogenicity will be assessed by a variety of immunological assays. This may include antibodies to SARS-CoV-Spike and non-Spike antigens by ELISA, ex vivo ELISpot assays for interferon gamma and flow cytometry assays, neutralising and other functional antibody assays and B cell analyses, virus neutralising Ab (NAb) assays against live and/or pseudotype SARS-CoV-2 virus. Other exploratory immunological assays including cytokine analysis and other antibody assays, DNA analysis of genetic polymorphisms potentially relevant to vaccine immunogenicity and gene expression studies amongst others may be performed at the discretion of the Investigators. Further exploratory immunology assays may be performed at the discretion of the Investigators on HIV and non-HIV cohorts, including, but not limited to: T cell Proliferative responses to SARS-CoV-2 antigen; T cell cross-reactivity to circulating common cold coronaviruses; Multiparameter immunophenotyping by CyTOF; BCR and TCR repertoire analysis; Serum analysis by Luminex (including inflammatory, anti-inflammatory and adaptive cytokines, chemokines, growth factors and antimicrobial proteins); HIV viral reservoir; amongst others.
- **Stool samples**; SARS-CoV-2 NAAT, infectivity assays, calprotectin, and other exploratory immunology and microbiology assays may be conducted in a subset of participants, subject to site capacity, sample and test availability

- **Mucosal Immunity Swabs (Synthetic Absorptive Matrix [SAM])**; an assessment of mucosal immunity will be conducted in a subset of participants, subject to site capacity, sample and test availability.
- **SARS-CoV-2 weekly PCR sample**; weekly nose/throat swabs will be processed via the Department of Health and Social Care's community testing programme.

At each site:

- **Urinalysis;** For female volunteers of child bearing potential only, urine will be tested for beta-human chorionic gonadotrophin (β-HCG) at screening (when applicable) and immediately prior to vaccination. Where local policies require, a serum β-HCG may replace urinary test.
- **Serum;** Samples may be centrifuged at local sites and shipped to University of Oxford laboratories or elsewhere for analysis.

SARS-CoV-2 serology will be conducted at screening (except in groups 5d, 9, 10 and 11). These may be conducted at appropriate university research or NHS trust laboratories facilities at sites. SARS-CoV-2 screening serology samples and or COVID-19 related immunology samples taken, may also be shipped from sites to a central laboratory facility at the University of Oxford or elsewhere.

Collaboration with other specialist laboratories in the UK, Europe and outside of Europe for further exploratory tests may occur. This would involve the transfer of serum, urine, stool or plasma, PBMC and/or other study samples to these laboratories, but these would remain anonymised. Informed consent for this will be gained from volunteers. Samples collected for the purposes of COVID-19 diagnosis might be sent to reference labs in the UK alongside their personal data. This would be in line with the national guidance and policy for submitting samples for testing at reference labs.

Immunological assays will be conducted according to local SOPs.

Subjects will be informed that there may be leftover samples of their blood (after all testing for this study is completed), and that such samples may be stored indefinitely for possible future research (exploratory immunology), including genotypic testing of genetic polymorphisms potentially relevant to vaccine immunogenicity. Subjects will be able to decide if they will permit such future use of any leftover samples. With the volunteers' informed consent, any leftover cells, urine, stool and serum/plasma will be frozen indefinitely for future analysis of COVID-19 and other coronaviruses related diseases or vaccine-related responses. If a subject elects not to permit this, all of that subject's

leftover samples will be discarded after the required period of storage to meet Good Clinical Practice (GCP) and regulatory requirements.

Samples that are to be stored for future research will be transferred to the OVC Biobank (REC 16/SC/0141).

7.3 Study visits

The study visits and procedures will be undertaken by one of the clinical trials team. The procedures to be included in each visit are documented in the schedule of attendances (tables 5-12). Each visit is assigned a time-point and a window period, within which the visit will be conducted.

7.3.1 Screening visit

Participants will be required to complete an online questionnaire as an initial confirmation of eligibility.

In order to minimise the risks of COVID-19 exposure in clinic, participants may be asked to provide verbal permission or electronic consent to collect and record details of their medical history over the phone, ahead of their screening visit (for the purpose of the eligibility assessment and if enrolled the recording of baseline health records). This will be recorded on their Pre-screening questionnaire (either directly completed by the volunteer or on behalf of the volunteer by a member of the study team with the volunteers verbal consent) or on the reply slip. This will reduce the amount of time participants have with the clinical team during their screening procedures.

All potential volunteers will have a screening visit, which may take place up to 90 days prior to vaccination. At the screening visit, a video presentation of the aims of the study and all tests to be carried out may be screened to an audience or accessed remotely. Individually each volunteer will have the opportunity to question an appropriately trained and delegated researcher before signing the consent. Informed consent will be taken before screening/enrolment, as described in section 6.2.

If written consent is obtained, the procedures indicated in the schedule of attendances will be undertaken including a medical history (if not already collected by phone), physical examination (if required), height and weight and blood tests including a SARS-CoV-2 screening test (all groups except 4c, 6b, 5d, 9, 10 and 11) and safety bloods (groups 1, 2, 5, 7 and 8) will be done. To avoid unnecessary additional venepuncture, if the appropriate blood test results for screening are available for the same

volunteer from a screening visit for another study, these results may be used for assessing eligibility (provided the results date is within the 6 months preceding enrolment in COV002).

We will aim to contact the subject's general practitioner with the permission of the subject after screening to corroborate medical history when possible and practical to do so (Groups 1, 2, 7 and 8 only, except group 12 where GPs can be replaced by their HIV consultant). GPs will be notified that the subject has volunteered for the study (all study groups,).

Abnormal clinical findings from blood tests at screening (Groups 1, 2, 5, 7 and 8 only) will be assessed by a medically qualified study member. Abnormal blood tests following screening will be assessed according to site-specific laboratory adverse event grading tables. Any abnormal test result deemed clinically significant may be repeated to ensure it is not a single occurrence. If an abnormal finding is deemed to be clinically significant, the volunteer will be informed and appropriate medical care arranged with the permission of the volunteer.

The eligibility of the volunteer will be reviewed at the end of the screening visit and again when all results from the screening visit have been considered. Decisions to exclude the volunteer from enrolling in the trial or to withdraw a volunteer from the trial will be at the discretion of the Investigator. If eligible, a day 0 visit will be scheduled for the volunteer to receive the vaccine and subsequent follow-up.

7.3.2 Day 0: Enrolment and vaccination visit

The parent(s)/legal guardian(s) of participants in group 3 and participants in all remaining groups will have informed consent taken as per section 6.2. Volunteers will be considered enrolled in to the trial at the point of vaccination. Before vaccination/trial intervention, the eligibility of the volunteer will be reviewed. Temperature will be observed and if necessary, a medical history and physical examination maybe undertaken to determine need to postpone vaccination or withdraw the participant. Vaccinations/trial intervention will be administered as described below.

7.3.2.1 Vaccination

All vaccines will be administered intramuscularly according to specific SOPs. The injection site will be covered with a sterile dressing and the volunteer will stay in the trial site for observation for a minimum of 15 minutes (+15 minutes), in case of immediate adverse events. The sterile dressing will be removed and injection site inspected.

In groups 1-3, 5, 7, 8, 11 and 12 and in a subset of volunteers in groups 4, 6, 9 and 10 (n=up to 1000, in each groups 4 and 6 and approximately 500 in each of groups 9 and 10), participants will be given an oral thermometer, tape measure and diary card (paper or electronic), with instructions on use. The approximate 3000 participants in groups 4, 6, 9 and 10 that are required to complete diaries will be allocated according to site. The allocation will ensure distribution of ages. All participants will be given the emergency 24 hour telephone number to contact the on-call study physician if needed. Volunteers will be instructed on how to self-assess the severity of these AEs. There will also be space on the diary card to self-document unsolicited AEs, and whether medication was taken to relieve the symptoms. Participants in groups 4, 6, 9 and 10 will be advised to take prophylactic paracetamol for 24 hours after vaccination and will record this in the e-diary (up to 1,000 participants in each of groups 4 and 6, and approximately 500 in each of groups 9 and 10 only). Participants in groups 1-3 and 5, 7, 8 and 11 will be asked to report on solicited AEs for 7 days and unsolicited AEs for 28 days. The subset of approximately 3000 participants in groups 4, 6, 9, and 10 will be asked to report solicited and unsolicited AEs for 7 days only. Participants in group 12 will be asked to report on solicited local and systemic AEs for 7 days and unsolicited AEs for 28 days.

Diary cards will collect information on the timing and severity of the following solicited AEs:

Table 4**. Solicited AEs as collected on post vaccination diary cards**

Local solicited AEs	Systemic solicited AEs
Pain	Fever
Tenderness	Feverishness
Redness	Chills
Warmth	Joint pains
Itch	Muscle pains
Swelling	Fatigue
Induration	Headache
	Malaise
	Nausea
	Vomiting

7.3.2.2 Sequence of Enrolment and Vaccination of Volunteers

Prior to initiation of the study, any newly available safety data will be reviewed from animal studies or clinical trials of coronavirus vaccines being tested in the UK (COV001) or elsewhere, and discussed with the DSMB and/or MHRA as necessary. Recruitment of groups 1, 4, 5, 6, 11 and 12 may occur simultaneously. However, older adults aged 56 and above will only be recruited into groups 4, 6, 7 and 8 following a safety review of participants enrolled in groups 1 and 2. This review will include the profile of AEs observed following a single dose of ChAdOx1 nCoV-19. Adults aged 56 and above will only be recruited into group 9 following safety review of groups 1 and 7, and into group 10 following safety review of groups 2 and 8.

7.3.3 Subsequent visits

Follow-up visits will take place as per the schedule of attendances described in tables 5-12 with their respective windows. Volunteers in groups 1-3 and 5, 7 and 8 will be assessed for local and systemic adverse events, interim history, physical examination, review of diary cards (paper or electronic) and blood tests at these time points as detailed in the schedule of attendances. Blood will also be taken for immunology purposes.

If volunteers experience adverse events (laboratory or clinical), which the investigator (physician), CI and/or DSMB chair determine necessary for further close observation, the volunteer may be admitted to an NHS hospital for observation and further medical management under the care of the Consultant on call.

Table 5 Schedule of attendances for participants in groups 1a, 2a, 7a and 8a (single dose)

Attendance Number	1^{S}	2	3	4	5	6	7	8	9	COVID-19 Testing	COVID-19 Testing +3-5 days	COVD-19 NAAT positive + 7 days	COVID-19 Follow-up
Timeline**(days)	≤ 90	0	3	7	14	28	56	182	364	As required	3-5 days post symptom onset	7 days post NAAT positive	
Time window (days)			±1	±2	±3	±7	±7	±14	±30	N/A	+2	±2	
Verbal Consent to discuss medical history over the phone	(X)												
Informed Consent	X												
Review contraindications, inclusion and exclusion criteria	X	X											
Vaccination		X											
Vital signs^	X	X	X	X	X	X	X	X	X	X	(X)	X	
Telephone/Video call													As required
Ascertainment of adverse events		X	X	X	X	X	X	X	X	X	(X)	X	X
Diary cards provided		X											X
Diary cards collected						X							X
Weekly household exposure questionnaire	ongoing												
Medical History, Physical Examination	X	(X)	(X)	(X)	(X)	(X)	(X)	(X)	(X)	(X)	(X)	(X)	
Biochemistry, Haematology (mL)	5	(5)*	5	5		5				5	(5)	5	

Attendance Number	1S	2	3	4	5	6	7	8	9	COVID-19 Testing	COVID-19 Testing +3-5 days	COVD-19 NAAT positive + 7 days	COVID-19 Follow-up
SARS-CoV-2 Serology	5												
Exploratory immunology (mL)		up to 55		up to 50	up to 50	up to 50	up to 50	up to 50	up to 50	up to 50		up to 50	
PAXgenes		2.5								2.5		2.5	
Nose/Throat Swab and/or Saliva Sample										X	(X)	(X)	
Stool sample[a,b]												(X)	(X)
Weekly PCR sample[a]	ongoing												
Urinary bHCG (women of childbearing potential only)	X	X											
HBsAg, HCV Ab, HIV serology (mL)	5												
Blood volume per visit	Up to 15	Up to 57.5	Up to 5	Up to 55	Up to 50	Up to 55	Up to 50	Up to 50	Up to 50	up to 57.5		up to 57.5	
Cumulative blood volume[%]	15	72.5	77.5	132.5	182.5	237.5	287.5	337.5	387.5	445		502.5	

S = screening visit; (X) = if considered necessary; ** Timeline is approximate only. Exact timings of visits relate to the day on enrolment, ie, each visit must occur at indicated number of days after enrolment ± time window. ^Vital signs at screening or pre-enrolment assessment on D0 include pulse, blood pressure, temperature, respiratory rate +/- oxygen saturation. Only temperature will be routinely measured at subsequent follow-up visits. At COVID-19 testing visits a full set of observations will be taken, including respiratory rate and oxygen saturation. % Cumulative blood volume for volunteers if blood taken as per schedule, and excluding any repeat safety blood test that may be necessary. Blood volumes may vary according to local site equipment and practices. *Safety bloods should only be repeated at vaccination day if there is a period greater than 2 weeks between screening and vaccination visit; an extra 5mL of blood should be added to the overall cumulative blood volume. [a] Subject to site capacity, sample and test availability. [b] Optional Stool sample at approximately 7 days after onset of symptoms for those who have a positive SARS-CoV-2 NAAT test result and possibly again at 14 days after symptom onset if necessary.

Table 6 Schedule of attendances for participants in groups 1b, 2b,5d, 7b and 8b (two dose)

Attendance Number	1[$]	2 (V1)	3	4	5	6 (V2)	7	8	9	10	11	12	COVID-19 Testing	COVID-19 Testing +3-5 days	COVD-19 NAAT positive +7 days	COVID-19 Follow-up
Timeline** (days)	≤ 90	0	3	7	14	28	31 (3 days post boost)	35 (7 days post boost)	42 (14 days post boost)	56 (28 days post boost	182	364	As required	3-5 days post symptom onset	7 days post NAAT positive	
Time window (days)			±1	±3	±3	+14	±1	±2	±3	±7	±14	±30	N/A	+2	±2	
Verbal Consent to discuss medical history over the phone	(X)															
Informed Consent	X															
Review contraindications, inclusion and exclusion criteria	X	X				X										
Vaccination		X				X										
Vital signs^	X	X	X	X	X	X	X	X	X	X	X	X	X	(X)	X	
Telephone/Video call																As required
Ascertainment of adverse events		X	X	X	X	X	X	X	X	X	X	X	X	(X)	X	X
Diary cards provided		X				X										X
Diary cards collected						X				X						X
Weekly household exposure questionnaire			ongoing													
Medical History, Physical Examination	X[c]	(X)	(X)	(X)	(X)	(X)	(X)	(X)	(X)	(X)	(X)	(X)	(X)	(X)	(X)	
Biochemistry[$], Haematology (mL)	5	(5)*	5	5		5	5	5		5			5	(5)	5	
SARS-CoV-2 Serology (mL)	(5)															
Exploratory immunology[£] (mL)		up to 55		up to 50	up to 50	up to 50		up to 50	up to 50	up to 50	up to 50	up to 50	up to 50		up to 50	
PAXgenes		2.5											2.5		2.5	

Attendance Number	1[S]	2 (V1)	3	4	5	6 (V2)	7	8	9	10	11	12	COVID-19 Testing	COVID-19 Testing +3-5 days	COVD-19 NAAT positive +7 days	COVID-19 Follow-up
Nasal/Throat Swab, and/or Saliva sample													X	(X)	(X)	
Stool sample [a,b]															(X)	(X)
Weekly PCR sample			ongoing													
Urinary bHCG (women of childbearing potential only)	X	X				X										
HBsAg, HCV Ab, HIV serology (mL)	5															
Blood volume per visit	Up to 15	Up to 57.5	Up to 5	Up to 55	Up to 50	Up to 55	Up to 5	Up to 55	Up to 50	Up to 55	Up to 50	Up to 50	up to 57.5		up to 57.5	
Cumulative blood volume[%]	15	72.5	77.5	132.5	182.5	237.5	242.5	297.5	347.5	402.5	452.5	502.5	560		617.5	

S = screening visit; (X) = if considered necessary; ** Timeline is approximate only. Exact timings of visits relate to the day on enrolment, ie, each visit must occur at indicated number of days after enrolment ± time window. Where a second dose is administered, the window will apply to the time their last vaccination took place ^Vital signs at screening or pre-enrolment assessment on D0 include pulse, blood pressure, temperature, respiratory rate +/- oxygen saturation. Only temperature will be routinely measured at subsequent follow-up visits. At COVID-19 testing visits a full set observations will be taken, including respiratory rate and oxygen saturation. % Cumulative blood volume for volunteers if blood taken as per schedule, and excluding any repeat safety blood test that may be necessary. Blood volumes may vary according to local site equipment and practices. *Safety bloods should only be repeated at vaccination day if there is a period greater than 2 weeks between screening and vaccination visit; an extra 5mL of blood should be added to the overall cumulative blood volume. [a] Subject to site capacity, sample and test availability. [b] Optional Stool sample at approximately 7 days after onset of symptoms for those who have a positive SARS-CoV-2 NAAT test result and possibly again at 14 days after symptom onset if necessary. [c] Targeted physical examination if considered necessary for group 5d.

Table 7: Schedule of attendances for participants in group 3

Attendance Number	1S	1	2	3	4	5	6	COVID-19 Testing	COVID-19 Testing +3-5 days	COVD-19 NAAT positive +7 days	COVID-19 Follow-up
Timeline**(days)		0	3	7	28	182	364	As required	3-5 days post symptom onset	7 days post COVID-19 Testing	
Time window (days)			±1	±2	±7	±14	±30	N/A	+2	±2	
Informed Consent	X										
Review contraindications, inclusion and exclusion criteria	X	X									
Vaccination		X									
Vital Signs ^	X	X	(X)	(X)	(X)	(X)	(X)	X	(X)	X	
Telephone/Video call											As required
Ascertainment of adverse events		X	X	X	X	X	X	X	(X)	X	X
Diary cards provided		X									X
Diary cards collected					X						X
Weekly household exposure questionnaire			ongoing								
Medical History, Physical Examination	(X)	(X)	(X)	(X)	(X)	(X)	(X)	(X)	(X)	(X)	
Biochemistry, Haematology (mL)		4	4[a]	4[b]	4			4	(5)	4	
SARS-CoV-2 Serology (mL)	3.5										
Exploratory immunology (mL)		up to 6 -16			up to 6 - 16	up to 10 -20	up to 10 - 20	up to 6 - 16		up to 6-16	
Nasal/Throat Swab and/or Saliva sample								X	(X)	(X)	
Weekly PCR sample[c]			ongoing								

Blood volume per visit (mL)	3.5	10-20	4	4	10 - 20	10 -20	10 - 20	10 – 20		10-20	
Cumulative blood volume[%]	3.5	13.5-23.5	17.5 - 27.5	17.5 - 27.5	27.5 – 47.5	37.5 – 67.5	47.5 – 87.5	57.5 – 107.5		67.5-127.5	

S = screening visit (X) = if considered necessary ^ = Vital signs includes temperature, a full set of observations will be done if clinically required; ** Timeline is approximate only. Exact timings of visits relate to the day on enrolment, ie, each visit must occur at indicated number of days after enrolment ± time window. [a,b,] Participants will have blood taken for safety and immunogenicity at one of 2 time points as outlined. Half of the participants will be bleed at D3 and half a t D7. [c] Subject to site capacity and test availability % Cumulative blood volume for volunteers if blood taken as per schedule and excluding any repeat safety blood test that may be necessary.

Table 8 Schedule of attendances for participants in group 4a and 6a

Attendance Number	1	2	3	4	5	6	COVID-19 Testing	COVID-19 Testing +3-5 days	COVD-19 NAAT positive +7 days	COVID-19 Follow-up
Timeline** (days)		0	28	90	182	364	As required	3-5 days post symptom onset	7 days post NAAT positive	
Time window (days)			-7/+14	±14	±14	±30	N/A	+2	±2	
Informed Consent	X	X								
Review contraindications, inclusion and exclusion criteria	X	X								
Vaccination		X								
Vital signs^	X	X	(X)	(X)	(X)	(X)	X	(X)	X	
Telephone/Video call										As required
Ascertainment of adverse events		X	X	X	X	X	X	(X)	X	X
Diary Cards[$]		X								
Symptoms diary										X
Weekly household exposure questionnaire			ongoing							
Medical History (required at 1 timepoint prior to enrolment),	X	X	(X)	(X)	(X)	(X)	(X)	(X)	(X)	
Physical Examination (if necessary)	(X)	(X)	(X)	(X)	(X)	(X)	(X)	(X)	(X)	
Biochemistry, Haematology (mL)							5	(5)	5	
Exploratory immunology (mL)		up to 15	up to10	up to 10	up to 10	up to 10	up to 50[d]		up to 50[d]	
Mucosal Immunity[a,c]		(X)	(X)							
PAXgenes						(2.5)[P]	2.5		2.5	
SARS-CoV-2 Serology	5									

Attendance Number	1	2	3	4	5	6	COVID-19 Testing	COVID-19 Testing +3-5 days	COVD-19 NAAT positive +7 days	COVID-19 Follow-up
Nose/Throat Swab and/or saliva sample							X	(X)	(X)	
Stool sample [a,b]									(X)	(X)
Weekly PCR sample[a]			ongoing							
Urinary bHCG (women of childbearing potential only)	X	X								
Blood volume per visit	5	15	10	10	10	10	up to 57.5		up to 57.5	
Cumulative blood volume[%]	5	20	30	40	50	60	117.5		175	

S = screening visit; (X) = if considered necessary; ** Timeline is approximate only. Exact timings of visits relate to the day on enrolment, ie, each visit must occur at indicated number of days after enrolment ± time window. ^Vital signs at screening or pre-enrolment assessment on D0 include pulse, blood pressure, temperature, respiratory rate +/- oxygen saturation. Only temperature will be routinely measured at subsequent follow-up visits. At COVID-19 testing visits a full set observations will be taken, including respiratory rate and oxygen saturation. % Cumulative blood volume for volunteers if blood taken as per schedule, and excluding any repeat safety blood test that may be necessary. Blood volumes may vary according to local site equipment and practices. [a] Subject to site capacity, sample and test availability. [d] Optional and subject to site capacity. [$] A subset of up to 1000 volunteers will be asked to fill an e-diary with reactogenicity symptoms for 7 days only in groups 4, 6, 9 and 10. [P] Pax genes sample at D364 to be done only on participants with positive SARS-CoV-2 NAAT at COVID-19 testing visits, an extra 2.5mL should be added to the cumulative blood volume when this applies. [b] Optional Stool sample at approximately 7 days after onset of symptoms for those who have a positive SARS-CoV-2 NAAT test result and possibly again at 14 days after symptom onset if necessary. [c] Mucosal immunity assessments to be done in a subset of group 6 individuals only.

Table 9: Schedule of attendances for participants in group 4b

Attendance Number	1	2	3	4	5	6	7	8	COVID-19 Testing	COVID-19 Testing +3-5 days	COVD-19 NAAT positive +7 days	COVID-19 Follow-up
Timeline** (days)		0	28	42 (14 days post boost)	56 (28 days post boost)	118 (90 days post boost)	210 (182 days post boost)	392 (364 days post boost)	As required	3-5 days post symptom onset	7 days post NAAT positive	
Time window (days)			+14	±7	±7	±14	±14	±30	N/A	+2	±2	
Informed Consent	X	X										
Review contraindications, inclusion and exclusion criteria	X	X										
Vaccination		X	X									
Vital signs^	X	X	(X)	(X)	(X)	(X)	(X)	(X)	X	(X)	X	
Telephone/Video call												As required
Ascertainment of adverse events		X	X	X	X	X	X	X	X	(X)	X	X
Diary Cards$		X	X									
Symptoms diary												X
Weekly household exposure questionnaire				ongoing								
Medical History (required at 1 timepoint prior to enrolment),	X	X	(X)	(X)		(X)	(X)	(X)	(X)	(X)	(X)	
Physical Examination (if necessary)	(X)	(X)	(X)	(X)		(X)	(X)	(X)	(X)	(X)	(X)	
Biochemistry, Haematology (mL)									5	(5)	5	
Exploratory immunology (mL)		up to 15	up to 20	up to 20	up to 20	up to 20	up to 20	up to 20	up to 50[d]		up to 50[d]	

Attendance Number	1	2	3	4	5	6	7	8	COVID-19 Testing	COVID-19 Testing +3-5 days	COVD-19 NAAT positive +7 days	COVID-19 Follow-up
PAXgenes								(2.5)[P]	2.5		2.5	
SARS-CoV-2 Serology	5											
Nose/Throat Swab and/or saliva sample									X	(X)	(X)	
Stool sample [a,b]											(X)	(X)
Weekly PCR sample[a]			ongoing									
Urinary bHCG (women of childbearing potential only)	X	X										
Blood volume per visit	5	15	20	20	20	20	20	20	up to 57.5		up to 57.5	
Cumulative blood volume[%]	5	20	40	60	80	100	120	140	197.5		255	

S = screening visit; (X) = if considered necessary; ** Timeline is approximate only. Exact timings of visits relate to the day. Where a second dose is administered, the window will apply to the time their last vaccination took place . ^Vital signs at screening or pre-enrolment assessment on D0 include pulse, blood pressure, temperature, respiratory rate +/- oxygen saturation. Only temperature will be routinely measured at subsequent follow-up visits. At COVID-19 testing visits a full set observations will be taken, including respiratory rate and oxygen saturation. % Cumulative blood volume for volunteers if blood taken as per schedule, and excluding any repeat safety blood test that may be necessary. Blood volumes may vary according to local site equipment and practices. [a] Subject to site capacity, sample and test availability. [d] Optional and subject to site capacity. [$] A subset of up to 1000 volunteers will be asked to fill an e-diary with reactogenicity symptoms for 7 days only in groups 4, 6, 9 and 10. [P] Pax genes sample at D364 to be done only on participants with positive SARS-CoV-2 NAAT at COVID-19 testing visits, an extra 2.5mL should be added to the cumulative blood volume when this applies. [b] Optional Stool sample at approximately 7 days after onset of symptoms for those who have a positive SARS-CoV-2 NAAT test result and possibly again at 14 days after symptom onset if necessary.

Table 10 Schedule of attendances for participants in group 5a1,5a2, 5b and 5c

Attendance Number	1[S]	2	3	4	5	6	7	8	9	COVID-19 Testing	COVID-19 Testing +3-5 days	COVD-19 NAAT positive + 7 days	COVID-19 Follow-up
Timeline**(days)	≤ 90	0	3	7	14	28	56	182	364	As required	3-5 days post symptom onset	7 days post NAAT positive	
Time window (days)			±1	±2	±3	±7	±7	±14	±30	N/A	+2	±2	
Informed Consent	X												
Review contraindications, inclusion and exclusion criteria	X	X											
Vaccination		X											
Vital signs	X	X	(X)	(X)	(X)	(X)	(X)	(X)	(X)	X	(X)	X	
Telephone/Video call													As required
Ascertainment of adverse events		X	X	X	X	X	X	X	X	X	(X)	X	X
Diary cards provided		X											X
Diary cards collected						X							X
Weekly household exposure questionnaire			ongoing										
Medical History, Physical Examination	(X)	(X)	(X)	(X)	(X)	(X)	(X)	(X)	(X)	(X)	(X)	(X)	
Biochemistry, Haematology (mL)	5	(5)*	5	5		5				5	(5)	5	
Hep B, C and HIV serology	5												
Exploratory immunology (mL)[e]		up to 55		up to 50	up to 50	up to 50	up to 50	up to 50	up to 50	up to 50		up to 50	
SARS-CoV-2 Serology	5												
PAXgenes		2.5								2.5		2.5	
Nose/Throat Swab and/or Saliva Sample										X	(X)	(X)	

Attendance Number	1[S]	2	3	4	5	6	7	8	9	COVID-19 Testing	COVID-19 Testing +3-5 days	COVD-19 NAAT positive + 7 days	COVID-19 Follow-up
Stool sample [a,b]												(X)	(X)
Weekly PCR sample			ongoing										
Urinary bHCG (women of childbearing potential only)	X	X											
HLA typing (mL)		4											
Blood volume per visit	15	61.5	5	55	50	55	50	50	50	up to 57.5		up to 57.5	
Cumulative blood volume[%]	15	76.5	81.5	136.5	186.5	241.5	291.5	341.5	391.5	449		506.5	

S = screening visit; (X) = if considered necessary; ** Timeline is approximate only. Exact timings of visits relate to the day on enrolment, ie, each visit must occur at indicated number of days after enrolment ± time window. ^Vital signs at screening or pre-enrolment assessment on D0 include pulse, blood pressure, temperature, respiratory rate +/- oxygen saturation. Only temperature will be routinely measured at subsequent follow-up visits. At COVID-19 testing visits a full set observations will be taken, including respiratory rate and oxygen saturation. % Cumulative blood volume for volunteers if blood taken as per schedule, and excluding any repeat safety blood test that may be necessary. Blood volumes may vary according to local site equipment and practices. *Safety bloods should only be repeated at vaccination day if there is a period greater than 2 weeks between screening and vaccination visit; an extra 5mL of blood should be added to the overall cumulative blood volume. [a] Subject to site capacity, sample and test availability. [b] Optional Stool sample at approximately 7 days after onset of symptoms for those who have a positive SARS-CoV-2 NAAT test result and possibly again at 14 days after symptom onset if necessary. [e] Participants enrolled in group 5b will only have B-cell immunology assessments whereas those in group 5c will have both B and T-cell immunology assessments.

Table 11 Schedule of attendances for participants in group 4c, 1a3, 1a4, 2a3, 2a4, 5a3, 5a4 and 6b (booster)

Attendance Number (boost)	1 V2 (booster)	2	3	4	5	COVID-19 Testing	COVID-19 Testing +3-5 days	COVD-19 NAAT positive + 7 days	COVID-19 Follow-up
Timeline** (days)	> 4 weeks post prime	28 post boost	90 post boost	182 post boost	364 post boost	As required	3-5 days post symptom onset	7 days post NAAT positive	
Time window (days)	+14	±7	±14	±14	±30	N/A	+2	±2	
Informed Consent	X								
Review contraindications, inclusion and exclusion criteria	X								
Vaccination	X								
Vital signs^	(X)	(X)	(X)	(X)	(X)	X	(X)	X	
Telephone/Video call									As required
Ascertainment of adverse events	X	X	X	X	X	X	(X)	X	X
Diary Cards$	X								
Symptoms diary									X
Weekly household exposure questionnaire		ongoing							
Medical History	(X)	(X)	(X)	(X)	(X)	(X)	(X)	(X)	
Physical Examination (if necessary)	(X)	(X)	(X)	(X)	(X)	(X)	(X)	(X)	
Biochemistry, Haematology (mL)						5	(5)	5	

Attendance Number (boost)	1 V2 (booster)	2	3	4	5	COVID-19 Testing	COVID-19 Testing +3-5 days	COVD-19 NAAT positive + 7 days	COVID-19 Follow-up
Exploratory immunology (mL)	up to 20	up to 20	up to 20	up to 20	up to 20	up to 50[d]		up to 50[d]	
PAXgenes					(2.5)[P]	2.5		2.5	
Nose/Throat Swab and/or saliva sample						X	(X)	(X)	
Stool sample [a,b]								(X)	(X)
Weekly PCR sample[a]		ongoing							
Urinary bHCG (women of childbearing potential only)	X								
Blood volume per visit	20	20	20	20	20	up to 57.5		up to 57.5	
Cumulative blood volume[%]	20	40	60	80	100	157.5		215	

S = screening visit; (X) = if considered necessary; ** Timeline is approximate only. Exact timings of visits relate to the day on enrolment, ie, each visit must occur at indicated number of days after enrolment ± time window. Where a second dose is administered, the window will apply to the time their last vaccination took place . ^Only temperature will be routinely measured at subsequent follow-up visits. At COVID-19 testing visits a full set observations will be taken, including respiratory rate and oxygen saturation. % Cumulative blood volume for volunteers if blood taken as per schedule, and excluding any repeat safety blood test that may be necessary. Blood volumes may vary according to local site equipment and practices. [a] Subject to site capacity, sample and test availability. [d] Optional and subject to site capacity. [$] A subset of up to 1000 volunteers will be asked to fill an e-diary with reactogenicity symptoms for 7 days only in groups 4, 6, 9 and 10. [P] Pax genes sample at D364 to be done only on participants with positive SARS-CoV-2 NAAT at COVID-19 testing visits, an extra 2.5mL should be added to the cumulative blood volume when this applies. [b] Optional Stool sample at approximately 7 days after onset of symptoms for those who have a positive SARS-CoV-2 NAAT test result and possibly again at 14 days after symptom onset if necessary.

Table 12. Schedule of attendances for participants in groups 9 and 10.

Attendance Number	1	2 V1	3 V2	4	5	6	7	COVID-19 Testing	COVID-19 Testing +3-5 days	COVD-19 NAAT positive+ 7 days	COVID-19 Follow-up
Timeline** (days)		0	28	56 (28 post boost)	118 (90 post boost	210 (182 post boost)	392 (364 post boost)	As required	3-5 days post symptom onset	7 days post NAAT positive	
Time window (days)			+14	±7	±14	±14	±30	N/A	+2	±2	
Informed Consent	X	X									
Review contraindications, inclusion and exclusion criteria	X	X									
Vaccination		X	X								
Vital signs^	X	X	(X)	(X)	(X)	(X)	(X)	X	(X)	X	
Telephone/Video call											As required
Ascertainment of adverse events		X	X	X	X	X	X	X	(X)	X	X
Diary Cards$		X	X								
Symptoms diary											X
Weekly household exposure questionnaire				ongoing							
Medical History (required at 1 timepoint prior to enrolment),	X	X	(X)		(X)	(X)	(X)	(X)	(X)	(X)	
Physical Examination (if necessary)	(X)	(X)	(X)		(X)	(X)	(X)	(X)	(X)	(X)	
Biochemistry, Haematology (mL)								5	(5)	5	
Exploratory immunology (mL)		up to 50	up to 50	up to 50	up to 50	up to 50	up to 50	up to 50[d]		up to 50[d]	

Attendance Number	1	2 V1	3 V2	4	5	6	7	COVID-19 Testing	COVID-19 Testing +3-5 days	COVD-19 NAAT positive+ 7 days	COVID-19 Follow-up
PAXgenes							(2.5)[P]	2.5		2.5	
Nose/Throat Swab and/or saliva sample								X	(X)	(X)	
Stool sample [a,b]										(X)	(X)
Weekly PCR sample[a]				ongoing							
Urinary bHCG (women of childbearing potential only)	X	X									
Blood volume per visit		50	50	50	50	50	50	up to 57.5		up to 57.5	
Cumulative blood volume[%]		50	100	150	200	250	300	357.5		415	

S = screening visit; (X) = if considered necessary; ** Timeline is approximate only. Exact timings of visits relate to the day on enrolment, ie, each visit must occur at indicated number of days after enrolment ± time window. Where a second dose is administered, the window will apply to the time their last vaccination took place. ^Only temperature will be routinely measured at subsequent follow-up visits. At COVID-19 testing visits a full set observations will be taken, including respiratory rate and oxygen saturation. % Cumulative blood volume for volunteers if blood taken as per schedule, and excluding any repeat safety blood test that may be necessary. Blood volumes may vary according to local site equipment and practices. [a] Subject to site capacity, sample and test availability. [d] Optional and subject to site capacity. [$] A subset of up to 1000 volunteers will be asked to fill an e-diary with reactogenicity symptoms for 7 days only in groups 4, 6, 9 and 10. [P] Pax genes sample at D364 to be done only on participants with positive SARS-CoV-2 NAAT at COVID-19 testing visits, an extra 2.5mL should be added to the cumulative blood volume when this applies. [b] Optional Stool sample at approximately 7 days after onset of symptoms for those who have a positive SARS-CoV-2 NAAT test result and possibly again at 14 days after symptom onset if necessary.

Table 13 Schedule of attendances for participants in group 11.

Attendance Number	1	2 V1	3	4 V2	5	6	7	8	COVID-19 Testing	COVID-19 Testing +3-5 days	COVD-19 NAAT positive +7 days	COVID-19 Follow-up
Timeline** (days)		0	14	28	56 (28 days post boost)	118 (90 days post boost	210 (182 days post boost)	392 (364 days post boost)	As required	3-5 days post symptom onset	7 days post NAAT positive	
Time window (days)			±3	+14	±7	±14	±14	±30	N/A	+2	±2	
Informed Consent	X	X										
Review contraindications, inclusion and exclusion criteria	X	X										
Vaccination		X		X								
Vital signs^	X	X		(X)	(X)	(X)	(X)	(X)	X	(X)	X	
Telephone/Video call												As required
Ascertainment of adverse events		X		X	X	X	X	X	X	(X)	X	X
Diary Cards		(X)		(X)								
Symptoms diary												X
Weekly household exposure questionnaire		ongoing										

Attendance Number	1	2 V1	3	4 V2	5	6	7	8	COVID-19 Testing	COVID-19 Testing +3-5 days	COVD-19 NAAT positive +7 days	COVID-19 Follow-up
Medical History (required at 1 timepoint prior to enrolment),	X	X		(X)		(X)	(X)	(X)	(X)	(X)	(X)	
Physical Examination (if necessary)	(X)	(X)		(X)		(X)	(X)	(X)	(X)	(X)	(X)	
Biochemistry, Haematology (mL)									5	(5)	5	
Exploratory immunology (mL)		up to 50	up to 50	up to 50	up to 50	up to 50	up to 50	up to 50	up to 50[d]		up to 50[d]	
PAXgenes								(2.5)[P]	2.5		2.5	
Nose/Throat Swab and/or saliva sample									X	(X)	(X)	
Stool sample [a,b]											(X)	(X)
Weekly PCR sample[a]			Ongoing									
Urinary bHCG (women of childbearing potential only)	X	X										
Blood volume per visit		50	50	50	50	50	50	52.5	up to 57.5	5	up to 57.5	
Cumulative blood volume[%]	0	50	100	150	200	250	300	352.5	410	415	472.5	472.5

S = screening visit; (X) = if considered necessary; ** Timeline is approximate only. Exact timings of visits relate to the day on enrolment, ie, each visit must occur at indicated number of days after enrolment ± time window. Where a second dose is administered, the window will apply to the time their last vaccination took place. ^Only temperature will be routinely measured at subsequent follow-up visits. At COVID-19 testing visits a full set observations will be taken, including respiratory rate and oxygen saturation. % Cumulative blood volume for volunteers if blood taken as per schedule, and excluding any repeat safety blood test that may be necessary. Blood volumes may vary according to local site equipment and practices. [a] Subject to site capacity,

sample and test availability. [d] Optional and subject to site capacity. [P] Pax genes sample at D364 to be done only on participants with positive SARS-CoV-2 NAAT at COVID-19 testing visits, an extra 2.5mL should be added to the cumulative blood volume when this applies. [b] Optional Stool sample at approximately 7 days after onset of symptoms for those who have a positive SARS-CoV-2 NAAT test result and possibly again at 14 days after symptom onset if necessary.

Table 14 Schedule of attendances for participants in group 12 (two dose)

Attendance Number	1^{S}	2 (V1)	3	4	5	6 (V2)	7	8	9	10	11	12	COVID-19 Testing	COVID-19 Testing +3-5 days	COVD-19 NAAT positive +7 days	COVID-19 Follow-up
Timeline** (days)	≤ 90	0	3	7	14	28	31 (3 days post boost)	35 (7 days post boost)	42 (14 days post boost)	56 (28 days post boost)	182	364	As required	3-5 days post symptom onset	7 days post NAAT positive	
Time window (days)			±1	±3	±3	+14	±1	±2	±3	±7	±14	±30	N/A	+2	±2	
Verbal Consent to discuss medical history over the phone	(X)															
Informed Consent	X															
Review contraindications, inclusion and exclusion criteria	X	X				X										
Vaccination		X				X										
Vital signs^	X	X	X	X	X	X	X	X	X	X	X	X	X	(X)	X	
Telephone/Video call																As required
Ascertainment of adverse events		X	X	X	X	X	X	X	X	X	X	X	X	(X)	X	X
Diary cards provided		X				X										X
Diary cards collected						X				X						X
Weekly household exposure questionnaire			ongoing													

Attendance Number	1[S]	2 (V1)	3	4	5	6 (V2)	7	8	9	10	11	12	COVID-19 Testing	COVID-19 Testing +3-5 days	COVD-19 NAAT positive +7 days	COVID-19 Follow-up
Medical History, Physical Examination	X	(X)	(X)	(X)	(X)	(X)	(X)	(X)	(X)	(X)	(X)	(X)	(X)	(X)	(X)	
SARS-CoV-2 Ab	5															
Biochemistry[$], Haematology (mL)	5	(5)*	5	5		5	5	5		5			5	(5)	5	
Exploratory immunology[£] (mL)		up to 95		up to 95	up to 95	up to 95		up to 95	up to 95	up to 95	up to 95	up to 95	up to 95		up to 95	
PAXgenes		2.5											2.5		2.5	
Nasal/Throat Swab, and/or Saliva sample													X	(X)	(X)	
Stool sample [a,b]															(X)	(X)
Weekly PCR sample			ongoing													
Urinary bHCG (women of childbearing potential only)	X	X				X										
HBsAg, HCV Ab, (mL)	5															
HIV serology (at investigators discretion)	(X)															
CD4 count and Viral Load	Up to 20mL															
Blood volume per visit	Up to 35	Up to 102.5	Up to 5	Up to 100	Up to 95	Up to 100	Up to 5	Up to 100	Up to 95	Up to 100	Up to 95	Up to 95	up to 102.5		up to 102.5	
Cumulative blood volume[%]	35	137.5	142.5	242.5	337.5	437.5	442.5	542.5	637.5	737.5	832.5	927.5	1030		1132.5	

S = screening visit; (X) = if considered necessary; ** Timeline is approximate only. Exact timings of visits relate to the day on enrolment, ie, each visit must occur at indicated number of days after enrolment ± time window. ^Vital signs at screening or pre-enrolment assessment on D0 include pulse, blood

pressure, temperature, respiratory rate +/- oxygen saturation. Only temperature will be routinely measured at subsequent follow-up visits. At COVID-19 testing visits a full set observations will be taken, including respiratory rate and oxygen saturation. % Cumulative blood volume for volunteers if blood taken as per schedule, and excluding any repeat safety blood test that may be necessary. Blood volumes may vary according to local site equipment and practices. *Safety bloods should only be repeated at vaccination day if there is a period greater than 2 weeks between screening and vaccination visit; an extra 5mL of blood should be added to the overall cumulative blood volume. [a] Subject to site capacity, sample and test availability. [b] Optional Stool sample at approximately 7 days after onset of symptoms for those who have a positive SARS-CoV-2 NAAT test result and possibly again at 14 days after symptom onset if necessary.

7.3.4 Participants under quarantine

Given the evolving epidemiological situation both globally and in the UK, should a participant be under quarantine and unable to attend any of the scheduled visits, a telephone/video consultation will be arranged in order to obtain core study data where possible.

7.3.5 Symptomatic volunteers

Participants who become symptomatic during follow-up will be instructed to call the study team who will then advise on how to proceed with clinical testing for COVID-19 if necessary, as per the trial working instructions. Participants will get weekly reminders (email or text messages) to get in touch with the study team if they present with a fever or cough or shortness of breath or anosmia/ageusia, experience any new event requiring medical attendance, or if they are admitted to hospital for any reason. At the COVID-19 testing visit, a nose/throat swab and/or saliva sample, blood samples for safety (FBC, Biochemistry, CRP and others if deemed clinically relevant) and immunology (paxgenes, cytokine profile, PBMCs, serum and others), vital signs and other clinical data will be taken. Symptomatic volunteers may be regularly reviewed over the phone or via video call using a smartphone or computer app if clinically appropriate.

Participants will be asked to attend a follow-up visit at 3-5 days post symptoms onset (+2 days) for clinical review and further testing or will be given a kit with instructions for a self-swab instead of a clinic visit. Participants will be asked to record information on COVID-19 related symptoms in an electronic diary for safety monitoring until symptom resolution or for at least 14 days. Participants who have a positive NAAT at S0, will not be required to attend a S3-5 visit (or provide a self-swab), but will be reviewed for safety at 7 days post positive swab. Clinical data, and additional blood samples for safety and immunology purposes will be taken at the S7 visit. Participants who have a positive swab at S3-5 will be reviewed for safety at 7 days post positive swab where clinical data, and additional blood samples for safety and immunology purposes will be taken. Participants who have 2 negative NAAT results from S0 and either a S3-5 visit or a self-swab will not be required to attend for an S7 visit. Closer follow-up and safety monitoring may be carried out by local trial teams if felt this is clinically indicated. If breathlessness is the only symptom that triggers a swab, further testing at S3-5 or S7 will be conducted at clinical discretion if there is no objective signs of respiratory distress

(e.g. tachypnea, desaturation). Immunology samples from symptomatic volunteers will be optional at their COVID-19 testing visits and will be subject to local site capacity.

Participants who develop COVID-19 symptoms and have a positive NAAT test after the first vaccination can only receive a booster dose after a minimum 4 weeks interval from their first NAAT positive test, provided their symptoms have significantly improved. The decision to proceed with booster vaccinations in those cases will be at clinical discretion of the investigators. For participants who are asymptomatic and have a positive NAAT test, a minimum of 2 weeks from first NAAT positivity will be required before boosting.

7.3.6 Weekly PCR samples

Participants may be asked provide a saliva and/or a nose/throat self-swab sample every week from the date of enrolment, which will be posted and processed in the Department of Health and Social Care's community testing programme. . This process will be detailed in trial specific instructions. Weekly PCR samples will be collected and processed depending on test availability, laboratory capacity, and other local screening programmes, which will determine the number of participants asked to provide weekly samples.

Participants with a positive test result from home testing (self-swabbing) will be notified of their test results by the Department of Health and Social Care community testing programme and advised to self-isolate as per current government guidance. No additional trial follow up of these participants will occur at this time unless they become symptomatic. Symptomatic volunteers will then be reviewed follow the procedures outlined in section 7.3.5 above.

7.3.7 Stool samples

Those participants who have a SARS-CoV-2 positive NAAT test result, may be asked to provide a stool sample at approximately 7 days after symptom onset or positive NAAT result if asymptomatic and 14 days after the first sample if necessary, as per trial specific instructions. Samples will be processed to look at differences in viral shedding between the investigational vaccine and control arms, and to measure calprotectin levels as a marker of gastrointestinal inflammation. These samples will be collected and processed depending on test availability, laboratory capacity, and will not be compulsory to the volunteers. Further exploratory immunology and microbiology tests may be conducted at the investigators' discretion.

7.4 Household Weekly Questionnaire (optional)

Participants will be asked to record information on a weekly basis about illnesses amongst household contacts and friends, their contact with the general public, and infection control procedures. This will be optional.

Volunteers will be asked to enter data in a diary from baseline to the end of the follow-up period. This will be recorded via a web-based electronic diary to which participants will be provided access at baseline. Participants working in clinical areas will be exempt from this questionnaire to reduce the study load on these participants whose exposure to COVID-19 is likely to be in the workplace rather than the home or community.

7.4.1 Medical notes review

With the participant's consent, the study team will request access to medical notes or submit a data collection form for completion by attending clinical staff on any medically attended COVID-19 episodes. Any data which are relevant to ascertainment of efficacy endpoints and disease enhancement (AESI) will be collected. These are likely to include, but not limited to, information on ICU admissions, clinical parameters such as oxygen saturation, respiratory rates and vital signs, need for oxygen therapy, need for ventilatory support, imaging and blood tests results, amongst others.

7.4.2 Randomisation, blinding and code-breaking

Participants will be randomised to investigational vaccine or MenACWY in a 3:1:3:1 (Groups 1 and 7), 5:1:5:1 (Groups 2 and 8) and 1:1 (groups 3, 4, 5a, 5b, 5c, 6, 9 and 10) and 5:1 (group 5d) allocation, using block randomisation. Group 11 will be open-label and randomisation to investigational vaccine or comparator will not apply.

Participants will be blinded to the arm they have been allocated to, whether investigational vaccine or MenACWY. The trial staff administering the vaccine will not be blinded. Vaccines will be prepared out of sight of the participant and syringes will be covered with an opaque object/material until ready for administration to ensure blinding.

If the clinical condition of a participant necessitates breaking the code, this will be undertaken according to a trial specific working instruction and group allocation sent to the attending physician, if unblinding is thought to be relevant and likely to change clinical management.

Additional steps may be taken to keep clinical investigators assessing primary endpoints blinded to group allocation, where this is possible and practical to do so. A designated member of the clinical team may be unblinded for the purposes of safety reporting procedures. All data from participants with NAAT-positive swabs will be assessed for inclusion in the primary efficacy analysis by two blinded assessors who will independently review each case according to pre-specified criteria as detailed in the statistical analysis plan, to classify each for inclusion in the primary and secondary outcomes.

8 INVESTIGATIONAL PRODUCT

8.1 Description of ChAdOx1 nCoV-19

ChAdOx1 nCoV-19 vaccine consists of the replication-deficient simian adenovirus vector ChAdOx1, containing the structural surface glycoprotein (Spike protein) antigens of SARS-CoV-2.

8.2 Supply

ChAdOx1 nCoV-19 has been formulated and vialed at Advent S.r.I. (Pomezia, Italy). Labelling has been done at the Clinical BioManufacturing Facility (University of Oxford) or Advent S.r.I. (Pomezia, Italy). It will be certified by a Qualified Person (QP) at the Clinical BioManufacturing Facility (University of Oxford) before release and transfer to the clinical site.

ChAdOx1 nCoV-19 (AZD1222) has been formulated at Cobra Biologics Ltd, vialed at Symbiosis Pharmaceutical Services, and labelled and packaged at Thermo Fisher Scientific (Hertfordshire, United Kingdom). It will be certified by a Qualified Person (QP) at the MedImmune Pharma, BV (Nijmegen, The Netherlands) or MedImmune Ltd (Cambridge, United Kingdom) before release and transfer to the clinical site.

8.3 Storage

The vaccine manufactured by Advent is stored at nominal -80°C (+/- 20 ^{o}C) in a secure freezer, at the clinical site. The vaccine manufactured by Cobra Biologics Ltd is stored at 2-8°C in a secure fridge, at the clinical site. All movements of the study vaccines will be documented in accordance with existing standard operating procedure (SOP). Vaccine accountability, storage, shipment and handling will be in accordance with relevant SOPs and forms. To allow for large number of participants to receive the vaccine in a short time period, additional clinic locations may be used. In this instance vaccines will be transported in accordance with local SOP's and approvals as required

8.4 Administration

On vaccination day, ChAdOx1 nCoV-19 will be allowed to thaw to room temperature and will be administered in accordance with trial specific instructions or stored at 2-8 for a maximum of 6 hours, where multiple doses are required from a single vial The vaccine manufactured by Cobra Biologics is a multi-dose vial which is stored at 2-8 degrees and does not require thawing. If the vaccine is stored

outside of 2-8 it must be used within 6 hours. The vaccine will be administered intramuscularly into the deltoid of the non-dominant arm (preferably). All volunteers will be observed in the unit for a minimum of 15 minutes (+15 minutes) after vaccination. During administration of the investigational products, Advanced Life Support drugs and resuscitation equipment will be immediately available for the management of anaphylaxis. Vaccination will be performed and the IMPs handled according to the relevant SOPs.

8.5 Rationale for selected dose

The dose to be administered in this trial have been selected on the basis of clinical experience with the ChAdOx1 adenovirus vector expressing different inserts and other similar adenovirus vectored vaccines (eg. ChAd63).

A first-in-man dose escalation study using the ChAdOx1 vector encoding an influenza antigen (FLU004), safely administered ChAdOx1 NP+M1 at doses ranging from 5 x 10^8 to 5 x 10^{10} vp. Subsequent review of the data identified an optimal dose of 2.5 x 10^{10} vp balancing immunogenicity and reactogenicity. This dose has subsequently been given to over hundreds of volunteers in numerous larger phase 1 studies at the Jenner Institute. ChAdOx1 vectored vaccines have thus far demonstrated to be very well tolerated. The vast majority of AEs have been mild-moderate and there have been no SARs until this date.

Another simian adenovirus vector (ChAd63) has been safely administered at doses up to 2 x 10^{11} vp with an optimal dose of 5 x 10^{10} vp, balancing immunogenicity and reactogenicity.

MERS001 was the first clinical trial of a ChAdOx1 vectored expressing the full-length Spike protein from a separate, but related betacoronavirus. ChAdOx1 MERS has been given to 31 participants to date at doses ranging from 5x10^9 vp to 5x10^{10} vp. Despite higher reactogeniticy observed at the 5x10^{10} vp, this dose was safe, with self-limiting AEs and no SARs recorded. The 5x10^{10} vp was the most immunogenic, in terms of inducing neutralising antibodies against MERS-CoV using a live virus assay [16]. Given the immunology findings and safety profile observed with a ChAdOx1 vectored vaccine against MERS-CoV, the 5x10^{10} vp dose was chosen for ChAdOx1 nCoV-19.

For children, a lower-dose of 2.5x10^{10} VP will be administered as the lower dose is likely to be less reactogenic and therefore more acceptable given the lower risk of severe illness in this age group.

The Clinical BioManufacturing Facility (CBF), who manufactured and tested batches 02P20-01 and 02P20-02 for the COV001 trial, determined vp/mL for Advent manufactured batch(es). This was done using a spectrophotometry-based methodology documented in their internal SOP (SOP A104). The doses to be administered in COV002, on Advent manufactured batches, will be determined by both methods (Abs260 and qPCR). Overseas studies conducted on Advent batch(es) will be dosed based on Advent's qPCR method. The University of Oxford is also performing a number of for information only tests for ChAdOx1 nCoV-19 batch(es) manufactured by Advent to demonstrate comparability between the different manufacturing processes and suitability of a reconstitution process involving dilution of the product with 0.9 % (w/v) saline that will be managed using local clinical trial SOPs available at each site/location.

For Advent Lot Number K.0007 the concentration is $1.7x10^{11}$ vp/mL (qPCR) which has been assessed by the CBF as equivalent to $3.89x10^{11}$ vp (Abs 260).

An analytical comparability assessment of ChAdOx1 nCoV-19 (AZD1222) manufactured by CBF, Advent and Cobra Biologics was conducted using a comprehensive set of physiochemical and biological release and characterization tests. In order to support the analytical comparability assessment, A260 testing of Advent's process (K.0007, K.0008, and K.0009 lots) was performed, where corrections to the absorbance due to excess polysorbate 80 were made to compensate for polysorbate 80 concentrations above the formulation target of 0.1% (w/v).

Differences in strength related attributes (ie, virus particle concentration, virus genome concentration, and infectious virus concentration) are noted. These differences in strength is further examined for potential impact on clinical dosing. The target clinical dosage of CBF's product is 5×10^{10} viral particles per dose based on vp/mL concentration determined by UV spectroscopy (A260), whereas that of Advent's product is 5×10^{10} viral genome copies per dose based on vg/mL concentration determined by qPCR. The target clinical dosage of Symbiosis' product is $3.5 – 6.5 \times 10^{10}$ viral particles per dose based on the vp/mL concentration determined by A260, with a 0.5 mL dosing volume. This dosing range is based on a target 5×10^{10} viral particles per dose and a ± 30% range to take into account process and method variabilities. The planned clinical dosage of Symbiosis' product is compared to that of CBF and Advent products, the resulting Symbiosis' product dosage at 0.5 mL for lot 20481A is somewhat lower in total viral particle per dose (20% from the lower range limit), slightly higher in total viral genome copies per dose (12% from the higher range limit), and slightly

lower in total infectious particle per dose (8% from the lower range limit). These differences are considered to be comparable to or within the variabilities from the analytical methods used in concentration determination (A260, qPCR, and infectivity) and the dosing volumes during clinical administration. In summary, with a 0.5 mL dosing volume for Symbiosis' product, strength difference from CBF and Advent products is not expected to have significant clinical impact in terms of reactogenicity and immunogenicity/efficacy.

Table 15 Clinical Strengths of ChAdOx1 nCoV-19 (AZD1222) Drug Product

Strength Attribute	CBF		Advent			Cobra
	Lot 02P20-01	Lot 02P20-02	Lot K.0007	Lot K.0008	Lot K.0009	Lot 20481A
**Concentration**						
Virus particle concentration (A_{260}) (vp/mL)	1.49×10^{11}	1.22×10^{11}	3.12×10^{11}	3.16×10^{11}	2.45×10^{11}	0.8×10^{11}
Virus genome concentration (qPCR) (vg/mL)	1.7×10^{11}	Not tested	1.7×10^{11}	2.1×10^{11}	1.4×10^{11}	1.3×10^{11}
Infectious particle concentration (ifu/mL) [a]	2.6×10^{9}	Not tested	2.9×10^{9}	3.0×10^{9}	2.4×10^{9}	1.3×10^{9}
**Target Clinical Dosage**						
Equivalent DP volume per dose (mL)	0.34	0.41	0.294	0.235	0.356	0.50
Dosing of virus particle (vp/dose)	5.1×10^{10}	5.0×10^{10}	9.2×10^{10}	7.4×10^{10}	8.7×10^{10}	4.0×10^{10}
Dosing of viral genome (vg/dose)	5.8×10^{10}	NA	5.0×10^{10}	4.9×10^{10}	5.0×10^{10}	6.5×10^{10}
Dosing of infectious particle (ifu/dose)	8.8×10^{8}	NA	8.5×10^{8}	7.1×10^{8}	8.5×10^{8}	6.5×10^{8}

[a] ifu = infectious units; NA = not applicable; vp = virus particle; vg = virus genome

[a] **Testing performed using the Advent infectivity assay.**

8.6 Minimising environmental contamination with genetically modified organisms (GMO)

The study will be performed in accordance with the current version of the UK Genetically Modified Organisms (Contained Use) Regulations. Approved SOPs will be followed to minimise dissemination of the recombinant vectored vaccine virus into the environment. GMO waste will be inactivated according to approved SOPs.

8.7 Control vaccine

Participants who are allocated to the control groups will receive one or two injections of MenACWY vaccine instead of ChAdOx1 nCoV-19. Either of the two licensed quadrivalent protein -polysaccharide conjugate vaccine MenACWY vaccines will be used, i.e.:

- Nimenrix (Pfizer). The licensed posology of this vaccine for those over 6 months of age is a single (0.5ml) intramuscular dose, containing 5mcg each of *Neisseria meningitidis* group A, C, W and Y polysaccharide, each conjugated to 44 mcg tetanus toxoid carrier protein.
- Menveo (Glaxosmithkline). The licensed posology of this vaccine for those 2 years of age and over is a single (0.5ml) intramuscular dose, containing
    - 10 mcg meningococcal group A polysaccharide, conjugated to 16.7 to 33.3 mcg *Corynebacterium diphtheriae* CRM_{197} protein
    - 5mcg meningococcal group C polysaccharide, conjugated to 7.1 to 12.5 mcg *C. diphtheriae* CRM_{197} protein
    - 5mcg meningococcal group W polysaccharide, conjugated to 3.3 to 8.3 mcg *C. diphtheriae* CRM_{197} protein
    - 5mcg meningococcal group Y polysaccharide, conjugated to 5.6 to 10.0 mcg *C. diphtheriae* CRM_{197} protein

The summary of product characteristics for both vaccines allows for administration of a booster dose if indicated by ongoing risk, therefore allows for the two doses administered to a subset of participants in this study. Similarly, previous receipt of either vaccine (or a plain polysaccharide quadrivalent meningococccal A, C, W and Y vaccine) will not be a contraindication to receiving a further vaccine in this study.

Participants will be blinded as to which injection they are receiving. A vaccine accountability log of MenACWY will be maintained at each trial site. There will be no additional labelling of these vaccines beyond their licensed packaging.

MenACWY will be stored in a locked (or access controlled) refrigerator (2°C – 8°C) at the sites, as per SmPC.

8.8 Compliance with Trial Treatment

All vaccinations will be administered by the research team and recorded in the CRF. The study medication will be at no time in the possession of the participant and compliance will not, therefore, be an issue.

8.9 Accountability of the Trial Treatment

Accountability of the IMP and MenACWY will be conducted in accordance with the relevant SOPs.

8.10 Concomitant Medication

As set out by the exclusion criteria, volunteers may not enter the study if they have received: any vaccine in the 30 days prior to enrolment, any investigational product within 30 days prior to enrolment or if receipt is planned during the study period, or if there is any chronic use (>14 days) of any immunosuppressant medication within 6 months prior to enrolment or if receipt is planned at any time during the study period (topical steroids are permitted).

Participants on continuous use of oral anticoagulants, such as coumarins and related anticoagulants (i.e. warfarin) or novel oral anticoagulants (i.e. apixaban, rivaroxaban, dabigatran and edoxaban) will be excluded from this trial, as per the exclusion criteria.

Participants in groups 4 and 6 will be advised to take Paracetamol after vaccination at 1g every 4-6 hours for the first 24 hours (maximum dose 4g within 24 hours). This will not be a requirement for study participation, and participants will have the option to not follow the advice.

8.11 Provision of Treatment for Controls

If this vaccine is proven to be efficacious following analysis of the primary endpoint and if the DSMB agrees, participants allocated to MenACWY group may be offered the IMP, should extra doses become available.

9 ASSESSMENT OF SAFETY

Safety will be assessed by the frequency, incidence and nature of AEs and SAEs arising during the study.

9.1 Definitions

9.1.1 Adverse Event (AE)

An AE is any untoward medical occurrence in a volunteer, which may occur during or after administration of an IMP and does not necessarily have a causal relationship with the intervention. An AE can therefore be any unfavourable and unintended sign (including any clinically significant abnormal laboratory finding or change from baseline), symptom or disease temporally associated with the study intervention, whether or not considered related to the study intervention.

9.1.2 Adverse Reaction (AR)

An AR is any untoward or unintended response to an IMP. This means that a causal relationship between the IMP and an AE is at least a reasonable possibility, i.e., the relationship cannot be ruled out. All cases judged by the reporting medical Investigator as having a reasonable suspected causal relationship to an IMP (i.e. possibly, probably or definitely related to an IMP) will qualify as AR.

Adverse events that may be related to the IMP are listed in the Investigator's Brochure for each product.

9.1.3 Serious Adverse Event (SAE)

An SAE is an AE that results in any of the following outcomes, whether or not considered related to the study intervention.

- Death

- Life-threatening event (i.e., the volunteer was, in the view of the Investigator, at immediate risk of death from the event that occurred). This does not include an AE that, if it occurred in a more severe form, might have caused death.
- Persistent or significant disability or incapacity (i.e., substantial disruption of one's ability to carry out normal life functions).
- Hospitalisation or prolongation of existing hospitalisation, regardless of length of stay, even if it is a precautionary measure for continued observation. Hospitalisation (including inpatient or outpatient hospitalisation for an elective procedure) for a pre-existing condition that has not worsened unexpectedly does not constitute a serious AE.
- An important medical event (that may not cause death, be life threatening, or require hospitalisation) that may, based upon appropriate medical judgment, jeopardise the volunteer and/or require medical or surgical intervention to prevent one of the outcomes listed above. Examples of such medical events include allergic reaction requiring intensive treatment in an emergency room or clinic, blood dyscrasias, or convulsions that do not result in inpatient hospitalisation.
- Congenital anomaly or birth defect.

9.1.4 Serious Adverse Reaction (SAR)

An AE that is both serious and, in the opinion of the reporting Investigator or Sponsors, believed to be possibly, probably or definitely due to an IMP or any other study treatments, based on the information provided.

9.1.5 Suspected Unexpected Serious Adverse Reaction (SUSAR)

A SAR, the nature and severity of which is not consistent with the information about the medicinal product in question set out in the IB.

9.2 Expectedness

No IMP related SAEs are expected in this study. All SARs will therefore be reported as SUSARs.

9.3 Foreseeable Adverse Reactions:

The foreseeable ARs following vaccination with ChAdOx1 nCoV-19 include injection site pain, tenderness, erythema, warmth, swelling, induration, pruritus, myalgia, arthralgia, headache, fatigue, fever, feverishness, chills, malaise, nausea and vomiting.

9.4 Adverse Events of Special Interest

Disease enhancement following vaccination with ChAdOx1 nCoV-19 will be monitored. Severe COVID-19 disease will be defined using clinical criteria. Detailed clinical parameters will be collected from medical records and aligned with agreed definitions as they emerge. These are likely to include, but are not limited to, oxygen saturation, need for oxygen therapy, respiratory rate, need for ventilatory support, imaging and blood test results, amongst other clinically relevant parameters.

Acute respiratory distress, pneumonitis, acute cardiac injury, arrhythmia, septic-shock like syndrome and acute kidney injury related with COVID-19 disease will be monitored from medical records review of hospitalised participants.

Kawasaki-like disease and other hyperinflammatory syndromes will be monitored and recorded as AESI in the paediatric group.

Eosinophilia as a marker skewed Th2 responses will be routinely monitored in participants attending their COVID-19 testing and follow-up visits. Marked eosinophilia of ≥ 1.5 x109/L will be reported as an SAE.

AESI relevant to vaccination in general will also be monitored such as: generalised convulsion, Guillain-Barre Syndrome (GBS), Acute Disseminated Encephalomyelitis (ADEM), Thrombocytopenia, Anaphylaxis, Vasculitides in addition to serious solicited AEs will be monitored.

9.5 Causality

For every AE, an assessment of the relationship of the event to the administration of the vaccine will be undertaken by the CI-delegated clinician. An interpretation of the causal relationship of the intervention to the AE in question will be made, based on the type of event; the relationship of the event to the time of vaccine administration; and the known biology of the vaccine therapy (Table 13). Alternative causes of the AE, such as the natural history of pre-existing medical conditions, concomitant therapy, other risk factors and the temporal relationship of the event to vaccination will

be considered and investigated. Causality assessment will take place during planned safety reviews, interim analyses (e.g. if a holding or stopping rule is activated) and at the final safety analysis, except for SAEs, which should be assigned by the reporting investigator, immediately, as described in SOP OVC005 Safety Reporting for CTIMPs.

0	**No Relationship**	No temporal relationship to study product ***and***<br>Alternate aetiology (clinical state, environmental or other interventions); ***and***<br>Does not follow known pattern of response to study product
1	**Unlikely**	Unlikely temporal relationship to study product ***and***<br>Alternate aetiology likely (clinical state, environmental or other interventions) ***and***<br>Does not follow known typical or plausible pattern of response to study product.
2	**Possible**	Reasonable temporal relationship to study product; ***or***<br>Event not readily produced by clinical state, environmental or other interventions; ***or***<br>Similar pattern of response to that seen with other vaccines
3	**Probable**	Reasonable temporal relationship to study product; ***and***<br>Event not readily produced by clinical state, environment, or other interventions ***or***<br>Known pattern of response seen with other vaccines
4	**Definite**	Reasonable temporal relationship to study product; ***and***<br>Event not readily produced by clinical state, environment, or other interventions; ***and***<br>Known pattern of response seen with other vaccines

Table 16. Guidelines for assessing the relationship of vaccine administration to an AE.

9.6 Reporting Procedures for All Adverse Events

All local and systemic AEs occurring in the 28 days following each vaccination observed by the Investigator or reported by the volunteer, whether or not attributed to study medication, will be recorded in electronic diaries or study database. Participants in a subset of groups 4 and 6 will be

asked to record local and systemic AE's for 7 days following vaccination in the electronic diary. Any unsolicited AEs reported by participants in any group at subsequent routine visits will be documented on the eCRF until at least 6 months of safety data has accrued for ChAdOx1 nCOV19. All AEs that result in a volunteer's withdrawal from the study will be followed up until a satisfactory resolution occurs, or until a non-study related causality is assigned (if the volunteer consents to this). SAEs and Adverse Events of Special Interest will be collected throughout the entire trial period.

9.7 Assessment of severity

The severity of clinical and laboratory adverse events will be assessed according to scales based on FDA toxicity grading scales for healthy and adolescent volunteers enrolled in preventive vaccine clinical trials, listed in the study specific working instructions and tables 14-16 below.

Adverse Event	Grade	Intensity
Pain at injection site	1	Pain that is easily tolerated
	2	Pain that interferes with daily activity
	3	Pain that prevents daily activity
	4	A&E visit or hospitalization
Tenderness	1	Mild discomfort to touch
	2	Discomfort with movement
	3	Significant discomfort at rest
	4	A&E visit or hospitalization
Erythema at injection site*	1	2.5 - 5 cm
	2	5.1 - 10 cm
	3	>10 cm
	4	Necrosis or exfoliative dermatitis
Induration/Swelling at injection site	1	2.5 – 5 cm and does not interfere with activity
	2	5.1 - 10 cm or interferes with activity
	3	>10 cm or prevents daily activity
	4	Necrosis

Table 17**. Severity grading criteria for local adverse events** *erythema ≤2.5cm is an expected consequence of skin puncture and will therefore not be considered an adverse event

**Vital Signs**	**Grade 1 (mild)**	**Grade 2 (moderate)**	**Grade 3 (severe)**	**Grade 4 Potentially Life threatening**
Fever (oral)	38.0°C - 38.4°C	38.5°C – 38.9°C	39.0°C - 40°C	> 40°C
Tachycardia (bpm)*	101 - 115	116 – 130	>130	A&E visit or hospitalisation for arrhythmia
Bradycardia (bpm)**	50 – 54	45 – 49	<45	A&E visit or hospitalisation for arrhythmia
Systolic hypertension (mmHg)	141 - 150	151 – 155	≥155	A&E visit or hospitalization for malignant hypertension
Diastolic hypertension (mmHg)	91 - 95	96 – 100	>100	A&E visit or hospitalization for malignant hypertension
Systolic hypotension (mmHg)***	85 - 89	80 – 84	<80	A&E visit or hospitalization for hypotensive shock
Respiratory Rate –breaths per minute	17 - 20	21-25	>25	Intubation

Table 18**. *Severity grading criteria for physical observations (applies to adults only).*** **Taken after ≥10 minutes at rest **When resting heart rate is between 60 – 100 beats per minute. Use clinical judgement when characterising bradycardia among some healthy subject populations, for example, conditioned athletes. ***Only if symptomatic (e.g. dizzy/ light-headed)*

**GRADE 0**	None
**GRADE 1**	Mild: Transient or mild discomfort (< 48 hours); No interference with activity; No medical intervention/therapy required
**GRADE 2**	Moderate: Mild to moderate limitation in activity – some assistance may be needed; no or minimal medical intervention/therapy required
**GRADE 3**	Severe: Marked limitation in activity, some assistance usually required; medical intervention/therapy required.
**GRADE 4**	Potentially Life-threatening: requires assessment in A&E or hospitalisation

Table 19. *Severity grading criteria for local and systemic AEs.* NB: A&E assessment in itself does not constitute a SAE. Refer to 9.1.3 for SAE definition

9.8 Reporting Procedures for Serious AEs

In order to comply with current regulations on SAE reporting to regulatory authorities, the event will be documented accurately and notification deadlines respected. SAEs will be reported on the SAE forms to members of the study team immediately the Investigators become aware of their occurrence, as described in SOP OVC005 Safety Reporting for CTIMPs. Copies of all reports will be forwarded for review to the Chief Investigator (as the Sponsor's representative) within 24 hours of the Investigator being aware of the suspected SAE. The DSMB will be notified of SAEs that are deemed possibly, probably or definitely related to study interventions; the chair of DSMB will be notified immediately (within 24 hours) of the sponsor being aware of their occurrence. SAEs will not normally be reported immediately to the ethical committee(s) unless there is a clinically important increase in occurrence rate, an unexpected outcome, or a new event that is likely to affect safety of trial volunteers, at the discretion of the Chief Investigator and/or DSMB. In addition to the expedited reporting above, the Investigator shall include all SAEs in the annual Development Safety Update Report (DSUR) report.

Grade 4 laboratory AEs should be reported as SAEs and under the category of outcome of an important medical event. A&E attendances should not routinely be reported as SAEs unless they meet the SAE definition described above.

Cases falling under the Hy's Law should be reported as SAEs. A Hy's Law Case is defined by FDA Guidance for Industry "Drug-Induced Liver Injury: Premarketing Clinical Evaluation" (2009). Any study participant with an increase in Aspartate Aminotransferase (AST) or **Alanine Aminotransferase (ALT) ≥ 3x Upper Limit of Normal (ULN) together with Total Bilirubin ≥2xULN**, **where no other reason can be found to explain the combination of these abnormal results**, e.g., elevated serum alkaline phosphatase (ALP) indicating cholestasis, viral hepatitis A, B or C, another drug capable of causing the observed injury, amongst others.

9.9 Reporting Procedures for SUSARS

All SUSARs will be reported by the sponsor delegate to the relevant Competent Authority and to the REC and other parties as applicable. For fatal and life-threatening SUSARS, this will be done no later than 7 calendar days after the Sponsor or delegate is first aware of the reaction. Any additional relevant information will be reported within 8 calendar days of the initial report. All other SUSARs will be reported within 15 calendar days.

Principal Investigators will be informed of all SUSARs for the relevant IMP for all studies with the same Sponsor, whether or not the event occurred in the current trial.

9.10 Development Safety Update Report

A Development Safety Update Report (DSUR) will be prepared annually, within 60 days of the anniversary of the first approval date from the regulatory authority for each IMP. The DSUR will be submitted by the CI to the Competent Authority, Ethics Committee, HRA (where required), Host NHS Trust and Sponsor.

9.11 Procedures to be followed in the event of abnormal findings

Eligibility for enrolment in the trial in terms of laboratory findings will be assessed by clinically qualified staff. Abnormal clinical findings from medical history, examination or blood tests will be assessed as to their clinical significance throughout the trial. Laboratory AEs will be assessed using specific toxicity grading scales adapted from the FDA Toxicity Grading Scale for Healthy Adult and Adolescent Volunteers Enrolled in Preventive Vaccine Clinical Trials. Paediatric laboratory AEs will be assessed using site specific paediatric laboratory reference ranges, values outside of these age

specific ranges will be reviewed by a study clinician to determine clinical significance. If a test is deemed clinically significant, it may be repeated, to ensure it is not a single occurrence. If a test remains clinically significant, the volunteer will be informed and appropriate medical care arranged as appropriate and with the permission of the volunteer. Decisions to exclude the volunteer from enrolling in the trial or to withdraw a volunteer from the trial will be at the discretion of the Investigator

9.12 Interim Safety Reviews

The safety profile will be assessed on an on-going basis by the Investigators. The CI and relevant Investigators (as per the trial delegation log) will also review safety issues and SAEs as they arise.

Immunopathology data from pre-clinical and phase 1 studies will be assessed by the CI, relevant investigators and the DSMB as soon as they are available and before any volunteers receive a dose of the IMP.

The DSMB will evaluate frequency of events, safety and efficacy data every 4-8 weeks and/or as required. The DSMB will make recommendations concerning the conduct, continuation or modification of the study.

In particular, the DSMB will review the data at the following key timepoints:

- Before vaccination of the first participant (all accumulated data available will be reviewed from COV001, with a minimum 4 weeks safety and immunogenicity data from the first 54 participants receiving the IMP and all accumulated data from the animal studies)
- Prior to expansion of the recruitment of groups 4 and 6 in those aged older than 55 years (data will be reviewed from groups 1 and 2)
- Prior to enrolment of the first child (all data available will be reviewed from both studies, with a minimum 4 weeks safety data from groups 1 and 2 in COV002 and 4 weeks safety data from all volunteers recruited into COV001)

9.13 Data Safety Monitoring Board

The Data Safety Monitoring Board that is in place for COV001 will also oversee COV002 and review data from both studies combined

The chair of the DSMB may be contacted for advice and independent review by the Investigator or trial Sponsor in the following situations:

- Following any SAE deemed to be possibly, probably or definitively related to a study intervention.
- Any other situation where the Investigator or trial Sponsor feels independent advice or review is important.

The DSMB will review SAEs deemed possibly, probably or definitively related to study interventions. The DSMB will be notified within 24 hours of the Investigators' being aware of their occurrence. The DSMB can recommend placing the study on hold if deemed necessary following a study intervention-related SAE.

The DSMB will only be able to judge the short-term safety of the ChAdOx1 nCoV-19 vaccine. Given the complexity of the underlying immunology and the minimal immunological data that will be available for review early in the study, the DSMB will not be in a position to comment on the effects of a later wave of SARS-CoV-2 as vaccine-induced immunity wanes and the theoretical risk of immune enhancement increases.

9.14 Safety Group Holding Rules

These safety holding rules apply to ChAdOx1 nCoV-19 vaccine only. Staggered enrolment will apply to group 3 where half of the overall number of participants allocated to the IMP arm will be vaccinated first and their safety data reviewed after 7 days before enrolment of the remainder. Solicited, unsolicited and laboratories adverse events will be systematically collected in groups 1, 2, 3, 5, 7 and 8. Only a sub-set of up to 1000 participants in each of Groups 4 and 6 will be asked to record solicited and unsolicited AEs for 7 days.

- **Solicited local adverse events:**
  - If more than 25% of doses of the vaccine at a given time point (e.g. Day 0, Day 28) in a study group are followed by the same Grade 3 solicited local adverse event beginning within 2 days after vaccination (day of vaccination and one subsequent day) and persisting at Grade 3 for >72 hrs
- **Solicited systemic adverse events:**

  - If more than 25% of doses of the vaccine at a given time point (e.g. Day 0, Day 28) in a study group are followed by the same Grade 3 solicited systemic adverse event beginning within 2 days after vaccination (day of vaccination and one subsequent day) and persisting at Grade 3 for >72 hrs

- **Unsolicited adverse events:**
  - If more than 25% of doses of the vaccine at a given time point (e.g. Day 0, Day 28) in a study group are followed by the same Grade 3 unsolicited adverse event beginning within 2 days after vaccination (day of vaccination and one subsequent day) and persisting at Grade 3 for >72 hrs

- **Laboratory adverse event:**
  - If more than 25% of doses of the vaccine at a given time point (e.g. Day 0, Day 28) in a study group are followed by the same Grade 3 laboratory adverse event beginning within 3 days after vaccination and persisting at Grade 3 for >72 hrs

- **A serious adverse event considered possibly, probably or definitely related to vaccination occurs**
  - If an SAE occurs in any one individual, which is possibly, probably or definitely related to vaccination this would trigger a holding rule. There are two exemptions from this rule, which would not activate a holding rule. These include:
    - COVID-19 related hospital admissions considered to be at least possibly related to ChAdOx1 nCoV-19 (e.g. if considered to be a clinical presentation of a disease enhancement episode). COVID-19 related SAEs will be regularly reviewed by the DSMB, and a single event will not trigger a holding rule.
    - SAEs reported under the Hy's Law requirement will not necessarily trigger a holding rule. These cases will also be reviewed by the DSMB

If any of the above holding rules are activated, then further vaccinations in any of the groups will not occur until a safety review by the DSMB, study sponsor and the chief investigator has been conducted and it is deemed appropriate to restart dosing. The Regulatory Authority will be informed and a

request to restart dosing with pertinent data will be submitted as a substantial amendment. The safety review will consider:

- The relationship of the AE or SAE to the vaccine.
- The relationship of the AE or SAE to the vaccine dose, or other possible causes of the event.
- If appropriate, additional screening or laboratory testing for other volunteers to identify those who may develop similar symptoms and alterations to the current Participant Information Sheet (PIS) are discussed.
- New, relevant safety information from ongoing research programs on the various components of the vaccine.

The local ethics committee and vaccine manufacturers will also be notified if a holding rule is activated or released.

All vaccinated volunteers will be followed for safety until resolution or stabilisation (if determined to be chronic sequelae) of their AEs.

9.14.1 Individual stopping rules (will apply to prime-boost groups only)

In addition to the above stated group holding rules, stopping rules for individual volunteers will apply (i.e., indications to withdraw individuals from further vaccinations). Study participants who present with at least one of the following stopping rules will be withdrawn from further vaccination in the study:

- **Local reactions:** Injection site ulceration, abscess or necrosis

- **Laboratory AEs:**
  the volunteer develops a Grade 3 laboratory AE considered possibly, probably or definitely related within 7 days after vaccination and persisting continuously at Grade 3 for > 72hrs.

- **Systemic solicited adverse events:**
  - the volunteer develops a Grade 3 systemic solicited AE considered possibly, probably or definitely related within 2 days after vaccination (day of vaccination and one subsequent day) and persisting continuously at Grade 3 for > 72hrs.

- **Unsolicited adverse events:**

- o the volunteer has a Grade 3 adverse event, considered possibly, probably or definitely related to vaccination, persisting continuously at Grade 3 for >72hrs.
- o the volunteer has a SAE considered possibly, probably or definitely related to vaccination.
- o the volunteer has an acute allergic reaction or anaphylactic shock following the administration of vaccine investigational product.

If a volunteer has an acute respiratory illness (moderate or severe illness with or without fever) or a fever (oral temperature greater than 37.8°C) at the scheduled time of administration of investigational product/control, the volunteer will not be enrolled and will be withdrawn from the study.

All vaccinated volunteers will be followed for safety until the end of their planned participation in the study or until resolution or stabilisation (if determined to be chronic sequelae) of their AEs, providing they consent to this.

In addition to these pre-defined criteria, the study can be put on hold upon advice of the DSMB, Chief Investigator, Study Sponsor, regulatory authority, Ethical Committee(s), for any single event or combination of multiple events which, in their professional opinion, jeopardise the safety of the volunteers or the reliability of the data.

10 STATISTICS

10.1 Description of Statistical Methods

Both a fully detailed study level statistical analysis plan (SAP) as well as a separate Statistical Analysis Plan for the Marketing Authorisation Application (MAA SAP) will be written and signed off before any interim data analyses are conducted.

The data from this study will be included in prospective pooled analyses of studies for efficacy and safety of ChAdOx1 nCoV-19 to provide greater precision of both efficacy and safety outcomes.

10.1.1 Efficacy Outcomes

The primary efficacy endpoint is PCR* positive symptomatic COVID-19.

This is defined as a participant with a PCR+* swab and at least one of the following symptoms: cough, fever ≥ 37.8, shortness of breath, anosmia, or ageusia.

Where possible, sensitivity analyses will be conducted using common alternative definitions of virologically-confirmed COVID-19 disease, including those in use in other phase 3 protocols (including but not limited to: USA AstraZeneca phase 3 trial, South Africa COV005 trial, WHO solidarity trial, CEPI definition). This will aid in comparisons between various studies and meta-analyses. These alternative definitions will be detailed in the statistical analysis plan as exploratory analyses.

*or other nucleic acid amplification test

10.2 Primary efficacy

The primary and secondary analyses will be conducted on participants who are seronegative at baseline. A sensitivity analysis will be conducted including all participants regardless of baseline serostatus.

Analysis of the primary endpoint will be computed as follows:

1. **Efficacy of two doses of vaccine** where the booster vaccine was a high-dose ChAdOx1 nCoV-19. Only participants who received two doses will be included (LD/SD or SD/SD) and only cases occurring more than 14 days after the second vaccine will be included.

Secondary analysis
2. **Efficacy of at least one standard-dose** of any ChAdOx1 nCoV-19. Cases occurring more than 21 days after the first vaccination will be included if the first vaccine was a high-dose vaccine. For participants who received a low-dose as their first vaccine, only cases occurring more than 14 days after a standard-dose booster will be included. Participants receiving only low-dose vaccines will be excluded.

3. **Efficacy of two standard-doses of vaccine**. Only participants who received two standard-dose vaccines will be included and only cases occurring more than 14 days after the second vaccine will be included.

Proportions will be compared between ChAdOx1 nCoV-19 and MenACWY groups using a Poisson regression model with robust variance (Zou 2004). The model will contain terms including treatment group, and age group at randomization if there is a sufficient sample size within each age category. The logarithm of the period at risk for primary endpoint will be used as an offset variable in the model to adjust for volunteers having different follow up times during which the events occur. Vaccine efficacy (VE) will be calculated as (1 – RR) x 100%, where RR is the relative risk of symptomatic infection (ChADOx1 nCoV-19: Control) and 95% confidence intervals will be presented.

If the Poisson regression model with robust variance fails to converge, the exact conditional method for stratified poisson regression will be used.

Cumulative incidence of symptomatic infections will be presented using the Kaplan-Meier method.

Secondary efficacy endpoints will be analysed in the same way as the primary efficacy endpoint.

Analyses will conducted for all adults combined as well as conducting analyses stratified by age cohorts.

All data from participants with PCR-positive swabs will be assessed for inclusion in the efficacy analyses by two blinded assessors who will independently review each case according to pre-specified criteria as detailed in the statistical analysis plan, to classify each for inclusion in the primary and secondary outcomes. A separate CRF will be designed for this purpose.

All PCR-positive results will be assessed for the primary outcome, including those with symptoms swabbed by trial staff, those with positive throat swabs from weekly home-testing, and other potential sources of information such as health-care workers who are tested at their workplace as either a routine test procedure or due to developing symptoms.

PCR+ swabs from outside the trial (for example, a workplace routine swab result in a healthcare worker) will be reviewed by blinded staff and only included as a potential endpoint if the test was conducted in 1) a medical laboratory with ISO 15189 accreditation (provided by UKAS in UK) AND 2) an assay that is either CE marked or that has a derogation authorisation from the MHRA.

*or other nucleic acid amplification test

10.3 Safety & Reactogenicity

Counts and percentages of each local and systemic solicited adverse reaction from diary cards, and all unsolicited AEs and SAEs will be presented for each group.

10.4 Immunogenicity

Highly skewed antibody data will be log-transformed prior to analysis. The geometric mean concentration and associated 95% confidence interval will be summarised for each group at each timepoint, by computing the anti-log of the mean difference of the log-transformed data.

The geometric mean concentration at day 28 and the proportion of participants seroconverting to the S-spike protein from day 0 to day 28 will be computed. Comparisons between ChAdOx1 nCoV-19 vaccine and MenACWY groups will be made using a Mann Whitney U test due to the low titres expected in the control group which will cause a non-normal distribution.

In addition, those aged 56 years or older receiving either a single-dose or two-doses of ChAdOx1 nCoV-19 vaccine will be compared with those in phase 1 (COV001) aged 18-55 years who received single-dose ChAdOx1 nCoV-19.

Spike-specific T cell responses (ELISpot) will be presented as means and confidence intervals, or medians and interquartile ranges if non-normally distributed at all post vaccination time points. Comparisons between ChAdOx1 nCoV-19 vaccine and MenACWY groups will be made using a Mann Whitney U test due to the low responses expected in the control group which will cause a non-normal distribution. Comparison between two different dose levels of ChAdOx1 nCoV-19 will be made using t-tests. In addition, those aged 56 years or older receiving either a single-dose or two-doses of ChAdOx1 nCoV-19 vaccine will be compared with those in phase 1 (COV001) aged 18-55 years who received single-dose ChAdOx1 nCoV-19.

10.5 Subgroup analyses

Subgroup comparisons of efficacy, and safety will be conducted by incorporating vaccine-group by subgroup interaction terms into appropriate regression models. Subgroup comparisons will only be conducted if there are at least 5 cases in all subgroups.

Comparisons will include:

1. Males vs females
2. Age (18 to 55 years vs 56-<70 years vs 70+ years)
3. Seropositive to S-spike or non-spike proteins at baseline vs not seropositive
4. Health care workers and highly-exposed participants versus others
5. Standard dose versus low dose

10.6 Interim and primary analyses of the primary outcome

It is planned that the primary evidence of efficacy and safety for the ChADOx1 nCoV-19 vaccine will be based on global analyses utilizing studies COV001 (the UK P1/2 study), COV002 (the UK P2/3 study), COV003 (the Brazil P3 study) and COV005 (the South Africa P1/2 study) including a pooled analysis across the studies. As such the interim and primary analyses for the primary outcome will be based on cases accumulated across multiple studies, details of which will be specified within the MAA SAP rather than for each individual study. Interim and primary data cuts from this study will therefore be carried out to support the pooled analysis.

The global MAA SAP allows for interim and primary analyses to be conducted once sufficient eligible cases have accumulated, where the overall type 1 error is controlled at the 5% level using a flexible alpha–spending approach that accounts for the incorporation of data from this study into pooled interim analyses under the global MAA SAP.

Evidence of efficacy will be determined if the lower bound of the multiplicity adjusted confidence interval is greater than a 20% threshold. The primary analysis will have approximately 90% power assuming a vaccine efficacy of 60%. A flexible alpha spending approach will be implemented to allow an earlier primary analysis in the situation where accumulation of eligible cases were lower than expected.

Evidence of efficacy at an interim or primary analysis of pooled data will not be considered a reason to stop the trial, but instead will be interpreted as early evidence of efficacy. However if an interim analysis demonstrates evidence of efficacy then a study level analysis according to the study SAP may be used to support study level evidence of efficacy.

10.7 Final Analysis

A final analysis will be conducted at the end of the study. The final study-specific analysis will incorporate all data from the study, including data that has previously contributed to global efficacy estimates under the pooled analysis strategy. The final analysis will be considered a supportive analysis to the global efficacy analysis. Alpha at the final study-specific analysis will be adjusted to incorporate the number of previous global analyses to which the study contributed data in order to control the overall study level type 1 error at 5%. Details will be specified in the study level SAP.

10.8 Procedure for Accounting for Missing, Unused, and Spurious Data.

All available data will be included in the analysis

10.9 Inclusion in Analysis

All vaccinated participants will be included in the analysis unless otherwise specified in the SAP.

10.10 Interim analysis for the combined DSMB

The independent DSMB will meet regularly to review safety data from all available studies of ChAdOx1 nCoV-19 and will assess whether the assumptions underlying the sample size calculation are in line with

the observed cases. Additionally the independent DSMB will make recommendations based on the interim analyses to assess evidence of efficacy.

11 DATA MANAGEMENT

11.1 Data Handling

The Chief Investigator will be responsible for all data that accrues from the study.

All study data including participant diary will be recorded directly into an Electronic Data Capture (EDC) system (e.g. OpenClinica, REDCap, or similar) or onto a paper source document for later entry into EDC if direct entry is not available. This includes safety data, laboratory data and outcome data. Any additional information that needs recording but is not relevant for the CRF (such as signed consent forms etc.) will be recorded on a separate paper source document. All documents will be stored safely and securely in confidential conditions.

All adverse event data (both solicited and unsolicited) reported by the volunteer will be entered onto a volunteer's electronic diary card (eDiary) for a maximum of 28 days following administration of the IMP. The eDiary provides a full audit trial of edits and will be reviewed at time-points as indicated in the schedule of events. Any adverse event continuing beyond the period of the diary will be copied into the eCRF and followed to resolution, if there is a causal relationship to the IMP, or to the end of the study if there is no causal relationship.

The participants will be identified by a unique trial specific number and code in any database. The name and any other identifying detail will NOT be included in any trial data electronic file, with the exception of the electronic diaries and household questionnaire, for which consent will be obtained to store the participant email address for quality control purposes. Only site research staff and sponsor data managers have access to view the email address.

The EDC system (CRF data) uses a relational database (MySQL/ PostgreSQL) via a secure web interface with data checks applied during data entry to ensure data quality. The database includes a complete suite of features which are compliant with GCP, EU and UK regulations and Sponsor security policies, including a full audit trail, user-based privileges, and integration with the institutional LDAP server. The MySQL and PostgreSQL database and the webserver will both be housed on secure servers maintained by the University of Oxford IT personnel. The servers are in a physically secure location in Europe. Backups will be stored in accordance with the IT department schedule of daily, weekly, and monthly retained for one month, three months, and six months, respectively. The IT servers provide a stable, secure, well-maintained, and high capacity data storage environment. REDCap and

OpenClinica are widely-used, powerful, reliable, well-supported systems. Access to the study's database will be restricted to the members of the study team by username and password.

If participants consent to provide stool samples; the stool sample (in an anonymised form) will be collected from them by a courier and processed in a laboratory by International Health Management Associates (IHMA), an accredited central laboratory. The sample will then be shipped for analysis by Astra Zeneca in a laboratory in the US. The participant would need to provide their name and address to the courier company.

11.2 Record Keeping

The Investigators will maintain appropriate medical and research records for this trial, in compliance with GCP and regulatory and institutional requirements for the protection of confidentiality of volunteers. The Chief Investigator, co-Investigators and clinical research nurses will have access to records. The Investigators will permit authorised representatives of the Sponsor(s), as well as ethical and regulatory agencies to examine (and when required by applicable law, to copy) clinical records for the purposes of quality assurance reviews, audits and evaluation of the study safety and progress.

Identifiable information such as contact details will be stored for a minimum of 5 years and until the youngest participant turns 21 years. De-identified research data maybe be stored indefinitely. If volunteers consent to be contacted for future research, information about their consent form will be recorded, retained and stored securely and separately from the research data. If volunteers consent to have their samples stored and used in future research, information about their consent form will be recorded, retained and stored securely as per Biobanking procedures and SOP.

11.3 Source Data and Case Report Forms (CRFs)

All protocol-required information will be collected in CRFs designed by the Investigator. All source documents will be filed in the CRF. Source documents are original documents, data, and records from which the volunteer's CRF data are obtained. For this study, these will include, but are not limited to, volunteer consent form, blood results, GP response letters, laboratory records, diaries, medical records and correspondence. In the majority of cases, CRF entries will be considered source data as the CRF is the site of the original recording (i.e. there is no other written or electronic record of data).

In this study this will include, but is not limited to medical history, medication records, vital signs, physical examination records, urine assessments, blood results, adverse event data and details of vaccinations. All source data and volunteer CRFs will be stored securely.

To prevent withdrawal of a participant due to relocation, if there is a nearby participating site and with the consent of the participant, copies of relevant participant research records (such as ICF, paper source documents) will be transferred to the local site using secure email addresses such as nhs.net or by password protected sheets. The electronic research data stored on REDCap will also be transferred to the new site. The original records will be retained by the recruiting site.

11.4 Data Protection

The study protocol, documentation, data and all other information generated will be held in strict confidence. No information concerning the study or the data will be released to any unauthorised third party, without prior written approval of the sponsor.

11.5 Data Quality

Data collection tools will undergo appropriate validation to ensure that data are collected accurately and completely. Datasets provided for analysis will be subject to quality control processes to ensure analysed data is a true reflection of the source data.

Trial data will be managed in compliance with local data management SOPs. If additional, study specific processes are required, an approved Data Management Plan will be implemented

11.6 Archiving

Study data may be stored electronically on a secure server, and paper notes will be kept in a key-locked filing cabinet at the site. All essential documents will be retained for a minimum of 5 years after the study has finished. The need to store study data for longer in relation to licensing of the vaccine will be subject to ongoing review. For effective vaccines that may be licensed, we may store research data securely at the site at least 15 years after the end of the study, subject to adjustments

in clinical trials regulations. Where relevant participants' bank details will be stored for 7 years in line with the site financial policy. De-identified research data maybe be stored indefinitely

General archiving procedures will be conducted in compliance to SOP OVC020 Archiving.

12 QUALITY CONTROL AND QUALITY ASSURANCE PROCEDURES

12.1 Investigator procedures

Approved site-specific standard operating procedures (SOPs) will be used at all clinical and laboratory sites.

12.2 Trial Steering Committee

A Trial Steering Committee will be appointed and will consist of an independent Chairman, not less than two other independent members and the Chief Investigator. All significant operational matters relating to the research will be decided upon by the trial steering committee that would have as main objectives:

- provide advice, through its chair, to the investigators, the trial sponsor, the collaborators on all appropriate aspects of the trial
- concentrate on progress of the trial, adherence to the protocol, patient safety and the consideration of new information of relevance to the research question
- agree proposals for substantial protocol amendments and provide advice to the sponsor and funder regarding approvals of such amendments

The trial steering committee will meet regularly and as required.

12.3 Monitoring

Regular monitoring will be performed according to GCP by the monitor. Following written SOPs, the monitor will verify that the clinical trial is conducted and data are generated, documented and reported in compliance with the protocol, GCP and the applicable regulatory requirements. The site will provide direct access to all trial related source data/documents and reports for the purpose of monitoring and auditing by the Sponsor and inspection by local and regulatory authorities.

12.4 Protocol deviation

Any deviations from the protocol will be documented in a protocol deviation form and filed in the trial master file. Each deviation will be assessed as to its impact on volunteer safety and study conduct. Significant protocol deviations will be listed in the end of study report.

12.5 Audit & inspection

The QA manager conducts systems based internal audits to check that trials are being conducted according to local procedures and in compliance with study protocols, departmental SOPs, GCP and applicable regulations.

The Sponsor, trial sites, and ethical committee(s) may carry out audit to ensure compliance with the protocol, GCP and appropriate regulations.

GCP inspections may also be undertaken by the MHRA to ensure compliance with protocol and the Medicines for Human Use (Clinical Trials) Regulations 2004, as amended. The Sponsor will assist in any inspections and will support the response to the MHRA as part of the inspection procedure.

13 SERIOUS BREACHES

The Medicines for Human Use (Clinical Trials) Regulations contain a requirement for the notification of "serious breaches" to the MHRA within 7 days of the Sponsor becoming aware of the breach.

A serious breach is defined as “A breach of GCP or the trial protocol which is likely to effect to a significant degree

(a) the safety or physical or mental integrity of the subjects of the trial; or

(b) the scientific value of the trial”.

In the event that a potential serious breach is suspected the Sponsor will be informed as soon as possible, to allow preliminary assessment of the breach and reporting to the MHRA within the required timelines.

14 ETHICS AND REGULATORY CONSIDERATIONS

14.1 Declaration of Helsinki

The Investigators will ensure that this study is conducted according to the principles of the current revision of the Declaration of Helsinki.

14.2 Guidelines for Good Clinical Practice

The Investigator will ensure that this trial is conducted in accordance with relevant regulations and with Good Clinical Practice.

14.3 Ethical and Regulatory Approvals

Following Sponsor approval the protocol, informed consent form, participant information sheet and any proposed advertising material will be submitted to an appropriate Research Ethics Committee (REC), HRA (where required), regulatory authorities (MHRA in the UK), and host institution(s) for written approval. No amendments to this protocol will be made without consultation with, and agreement of, the Sponsor.

The Investigator is responsible for ensuring that changes to an approved trial, during the period for which regulatory and ethical committee(s) approval has already been given, are not initiated without regulatory and ethical committee(s)' review and approval except to eliminate apparent immediate hazards to the subject (i.e. as an Urgent Safety Measure).

14.4 Volunteer Confidentiality

The study will comply with the General Data Protection Regulation (GDPR) and Data Protection Act 2018, which require data to be de-identified as soon as it is practical to do so. The processing of personal data of participants will be minimised by making use of a unique participant study number only on all study documents and any electronic database(s), with the exception of informed consent forms, participant ID logs, electronic diaries and the Household Questionnaire. All documents will be stored securely and only accessible by study staff and authorised personnel. The study staff will safeguard the privacy of participants' personal data. A separate confidential file containing identifiable information will be stored in a secured location in accordance with the current data

protection legislation. Photographs taken of vaccination sites (if required, with the volunteer's written, informed consent) will not include the volunteer's face and will be identified by the date, trial code and subject's unique identifier. Once developed, photographs will be stored as confidential records, as above. This material may be shown to other professional staff, used for educational purposes, or included in a scientific publication.

If participants have a positive swab result for COVID-19 during the course of the study then the Public Health Authority will be notified as COVID-19 is a "notifiable disease" and this is a legal requirement in the UK. This may mean participants personal information from their health records will be shared with Public Health either by the processing lab or the study site. Participants may also be contacted by the NHS Test and Trace service. Samples collected using home swab kits may be processed at laboratories within and outside the UK, as determined by the community testing programme. These laboratories provide a test result for the barcode to NPEx (National Pathology Exchange) and this result is then recombined with participant identifiable information by NHS Digital. NHS Digital provide lab results to the Sponsor (University of Oxford) who will match this with personal data including identifying contact information sent to them by the site in order to centralise the processing of weekly surveillance results. Participants will be required to separately consent to the terms and conditions of the national community swabbing programme, each time they perform a self-swab. This is available at: https://www.gov.uk/government/publications/coronavirus-covid-19-testing-privacy-information/testing-for-coronavirus-privacy-information

15 FINANCING AND INSURANCE

15.1 Financing

The study is funded by the UK Government through the National Institute for Health Research (NIHR). AstraZeneca have provided funding for some exploratory objectives.

15.2 Insurance

The University has a specialist insurance policy in place which would operate in the event of any participant suffering harm as a result of their involvement in the research (Newline Underwriting Management Ltd, at Lloyd's of London). NHS indemnity operates in respect of the clinical treatment which is provided.

15.3 Compensation

Volunteers in groups 1, 2, 5, 7, 8 and 12 will be compensated for their time, the inconvenience of having blood tests and procedures, and their travel expenses. The total amount compensated will be approximately £390-555 depending on the exact number of visits, and whether any repeat or additional visits are necessary. They will be compensated £25 for attending the screening visit. For all other trial visits as outlined in Tables 5-14, compensation will be calculated according to the following:

- Travel expenses: £15 per visit
- Inconvenience of blood tests: £10 per blood donation
- Time required for visit: £20 per hour

Paediatric volunteers in group 3 will be compensated approximately £50 depending on the exact number of visits, and whether any repeat or additional visits are necessary. They will receive a £10 voucher for each study visit which will be given on completion of the trial. Should a volunteer from any group decide to withdraw from the trial before it is completed, payment will be pro rata.

Participants in Groups 4, 6, 9, 10 and 11 will not be compensated.

15.4 Publication Policy

The Investigators will be involved in reviewing drafts of the manuscripts, abstracts, press releases and any other publications arising from the study. Data from the study may also be used as part of a thesis for a PhD or MD.

16 DEVELOPMENT OF A NEW PRODUCT/ PROCESS OR THE GENERATION OF INTELLECTUAL PROPERTY

Ownership of IP generated by employees of the University rests in the University. The protection and exploitation of any new IP is managed by the University's technology transfer office, Oxford University Innovations. Investigators in this study may benefit from the royalty sharing policy of the University if new intellectual property is generated from the trial. Several investigators are applicants

or co-inventors on previous patent filings or patents related to ChAdOx1 vaccines. The University of Oxford, which is partnered with the Oxford University Hospitals NHS Foundation Trust in the NIHR Oxford Biomedical Research Centre, is committed to the translational progress and commercial development of healthcare products potentially meeting medical and global health needs, and does and will work with commercial partners towards these goals.

References

1 Zhu, N. *et al.* A Novel Coronavirus from Patients with Pneumonia in China, 2019. *New England Journal of Medicine* **382**, 727-733, doi:10.1056/NEJMoa2001017 (2020).

2 Lu, R. *et al.* Genomic characterisation and epidemiology of 2019 novel coronavirus: implications for virus origins and receptor binding. *Lancet* **395**, 565-574, doi:10.1016/S0140-6736(20)30251-8 (2020).

3 Li, F. Structure, Function, and Evolution of Coronavirus Spike Proteins. *Annual review of virology* **3**, 237-261, doi:10.1146/annurev-virology-110615-042301 (2016).

4 Zhou, P. *et al.* A pneumonia outbreak associated with a new coronavirus of probable bat origin. *Nature*, doi:10.1038/s41586-020-2012-7 (2020).

5 Alharbi, N. K. *et al.* ChAdOx1 and MVA based vaccine candidates against MERS-CoV elicit neutralising antibodies and cellular immune responses in mice. *Vaccine* **35**, 3780-3788, doi:10.1016/j.vaccine.2017.05.032 (2017).

6 Tseng, C. T. *et al.* Immunization with SARS coronavirus vaccines leads to pulmonary immunopathology on challenge with the SARS virus. *PLoS One* **7**, e35421, doi:10.1371/journal.pone.0035421 (2012).

7 Weingartl, H. *et al.* Immunization with modified vaccinia virus Ankara-based recombinant vaccine against severe acute respiratory syndrome is associated with enhanced hepatitis in ferrets. *J Virol* **78**, 12672-12676, doi:10.1128/JVI.78.22.12672-12676.2004 (2004).

8 Liu, L. *et al.* Anti-spike IgG causes severe acute lung injury by skewing macrophage responses during acute SARS-CoV infection. *JCI insight* **4**, e123158, doi:10.1172/jci.insight.123158 (2019).

9 Agrawal, A. S. *et al.* Immunization with inactivated Middle East Respiratory Syndrome coronavirus vaccine leads to lung immunopathology on challenge with live virus. *Hum Vaccin Immunother* **12**, 2351-2356, doi:10.1080/21645515.2016.1177688 (2016).

10 Munster, V. J. *et al.* Protective efficacy of a novel simian adenovirus vaccine against lethal MERS-CoV challenge in a transgenic human DPP4 mouse model. *NPJ vaccines* **2**, 28, doi:10.1038/s41541-017-0029-1 (2017).

11 Alharbi, N. K. *et al.* Humoral Immunogenicity and Efficacy of a Single Dose of ChAdOx1 MERS Vaccine Candidate in Dromedary Camels. *Scientific reports* **9**, 16292, doi:10.1038/s41598-019-52730-4 (2019).

12 Gao, Q. *et al.* Development of an inactivated vaccine candidate for SARS-CoV-2. *Science*, eabc1932, doi:10.1126/science.abc1932 (2020).

13 Antrobus, R. D. *et al.* Clinical assessment of a novel recombinant simian adenovirus ChAdOx1 as a vectored vaccine expressing conserved Influenza A antigens. *Molecular therapy : the journal of the American Society of Gene Therapy* **22**, 668-674, doi:10.1038/mt.2013.284 (2014).

14 Coughlan, L. *et al.* Heterologous Two-Dose Vaccination with Simian Adenovirus and Poxvirus Vectors Elicits Long-Lasting Cellular Immunity to Influenza Virus A in Healthy Adults. *EBioMedicine*, doi:https://doi.org/10.1016/j.ebiom.2018.02.011 (2018).

15 Wilkie, M. *et al.* A phase I trial evaluating the safety and immunogenicity of a candidate tuberculosis vaccination regimen, ChAdOx1 85A prime - MVA85A boost in healthy UK adults. *Vaccine* **38**, 779-789, doi:10.1016/j.vaccine.2019.10.102 (2020).

16 Folegatti, P. M. *et al.* Safety and immunogenicity of a candidate Middle East respiratory syndrome coronavirus viral-vectored vaccine: a dose-escalation, open-label, non-randomised,

uncontrolled, phase 1 trial. *The Lancet Infectious Diseases*, doi:https://doi.org/10.1016/S1473-3099(20)30160-2 (2020).

17 Modjarrad, K. MERS-CoV vaccine candidates in development: The current landscape. *Vaccine* **34**, 2982-2987, doi:10.1016/j.vaccine.2016.03.104 (2016).

18 Afolabi, M. O. *et al.* Safety and Immunogenicity of ChAd63 and MVA ME-TRAP in West African Children and Infants. *Molecular therapy : the journal of the American Society of Gene Therapy* **24**, 1470-1477, doi:10.1038/mt.2016.83 (2016).

19 Tiono, A. B. *et al.* First field efficacy trial of the ChAd63 MVA ME-TRAP vectored malaria vaccine candidate in 5-17 months old infants and children. *PloS one* **13**, e0208328, doi:10.1371/journal.pone.0208328 (2018).

20 She, J., Liu, L. & Liu, W. COVID-19 epidemic: Disease characteristics in children. *J Med Virol*, doi:10.1002/jmv.25807 (2020).

21 Dong, Y. *et al.* Epidemiology of COVID-19 Among Children in China. *Pediatrics*, doi:10.1542/peds.2020-0702 (2020).

22 Ludvigsson, J. F. Systematic review of COVID-19 in children shows milder cases and a better prognosis than adults. *Acta Paediatr*, doi:10.1111/apa.15270 (2020).

23 Zimmermann, P. & Curtis, N. Coronavirus Infections in Children Including COVID-19: An Overview of the Epidemiology, Clinical Features, Diagnosis, Treatment and Prevention Options in Children. *The Pediatric Infectious Disease Journal* **39**, 355-368, doi:10.1097/inf.0000000000002660 (2020).

24 Verdoni, L. *et al.* An outbreak of severe Kawasaki-like disease at the Italian epicentre of the SARS-CoV-2 epidemic: an observational cohort study. *The Lancet*, doi:https://doi.org/10.1016/S0140-6736(20)31103-X (2020).

25 Parmigiani, A. *et al.* Impaired antibody response to influenza vaccine in HIV-infected and uninfected aging women is associated with immune activation and inflammation. *PLoS One* **8**, e79816, doi:10.1371/journal.pone.0079816 (2013).

26 Shive, C. L. *et al.* Pre-vaccine plasma levels of soluble inflammatory indices negatively predict responses to HAV, HBV, and tetanus vaccines in HCV and HIV infection. *Vaccine* **36**, 453-460, doi:10.1016/j.vaccine.2017.12.018 (2018).

APPENDIX A: AMENDMENT HISTORY

Amendment No.	Protocol Version No.	Date issued	Author(s) of changes	Details of Changes made
N/A	1.0	03 APR 2020	Pedro Folegatti, Andrew Pollard, Merryn Voysey, Sarah Gilbert	N/A
1	2.0	14 APR 2020	Emma Plested	Addition of North Bristol NHS Trust as a site.
N/A	3.0	30 APR 2020	Pedro Folegatti	Added rationale for recruiting paediatric groups; Added stopping/holding rules to groups 1, 2 and 3; Specified minimum safety and immunogenicity data required from COV001 prior to start of COV002; Specified minimum safety data required prior to enrolment into older participants in group 4 and enrolment of children into group 3; Added staggered enrolment with interim reviews for groups 1, 2 and 3.
2	4.0	14 May 2020	Pedro Folegatti	Added Dr Angela Minassian as an Investigator; 1 year follow-up as standard trial procedures; added group 5 for batch safety and immunogenicity comparison with COV001; increased sample size to up to 10,260 and adjusted statistical analysis section to reflect this; changes and clarifications to exclusion criteria; added priority groups for recruitment; added anosmia/ageusia as part of the trigger for swabbing criteria; added efficacy against infection as tertiary/exploratory

Amendment No.	Protocol Version No.	Date issued	Author(s) of changes	Details of Changes made
				endpoint; weekly PCR samples subject to test availability and site capacity; HCW exemption from filling-out weekly COVID-19 exposure diaries; adjusted blood volumes; added a section on changes to group numbers in the event of further different batches being required in order to complete dosing; clarifications to storage conditions of the IMP; harmonised AESI section with COV001 and as per Brighton Collaboration suggestions; introduced prophylactic paracetamol in group 4; Changes to funding arrangements; added multiple sites.
3	5.0	26 May 2020	Merryn Voysey, Pedro Folegatti, Maheshi Ramasamy	Addition of age stratification in randomisation to group 4 (<55years and ≥56years); Corrections to site addresses; updated information on pre-clinical data and disease enhancement/immunopathology; expanded on rationale for recruiting children; addition of exploratory endpoint for batch comparison between COV001 and COV002; Groups 1 and 2 to be recruited sequentially instead of in parallel following request from the DSMB with a minimum of 7 days interval; updated section on potential risks to volunteers following preliminary pre-clinical and clinical data on ChAdOx1 nCoV-19; removed potential benefit from taking part in the study as participants in

Amendment No.	Protocol Version No.	Date issued	Author(s) of changes	Details of Changes made
				group 4 won't necessarily undergo physical examination; clarification to recruitment strategy on priority groups; clarification to additional exclusion criteria in group 4 where caution to be taken when advising Paracetamol to participants on chronic use; Added PAXgene sample to last follow-up visit in participants who had a positive COVID-19 PCR sample at diagnosis visit; removed baseline PCR swab; additional text to encourage participants to contact study team for any medical attended event; clarification to weekly swab procedures; Swab testing to be undertaken by DHSC and data from this and any COVID-19 testing will be shared with lead site for central analysis; Nasopharyngeal swab to be conducted at 7 days post COVID-19 diagnosis visit only if considered necessary or if first sample is negative; added Kawasaki-like disease and other hyperinflammatory syndromes as AESI in the paediatric group; clarification to holding rule procedures, so it applies to all groups if a holding rule is met; clarifications to statistical analysis section; added information on volunteer confidentiality regarding weekly swabs data processing; clarification that participants in Group 4 will not be compensated; correction of formatting and typographical errors throughout the document; added information blinding

Amendment No.	Protocol Version No.	Date issued	Author(s) of changes	Details of Changes made
				for efficacy endpoints; clarification of the groups required to report AEs; Option for participants to transfer to existing sites if relocating
4	6.0	05-JUN-2020	Pedro Folegatti	Reduced number of participants recruited into group 4; Added group 6 for comparison between dosing on Abs260 and qPCR methods.
5	7.0	18 Jun 2020	Pedro Folegatti, Merryn Voysey, Hannah Robinson	Addition of groups 5 A, B/C, 7 A and B & 8 A and B (reactogeniticy and immunogenicity comparison between different doses given with different methods for measuring doseS); Increase in sample size to up to 10,560;Group 4b has been added to provide immunogenicity data on homologous prime-boost at $5x10^{10}$vp (Abs260) prime and 2.2x1010vp (qPCR) boost, where up to 100 volunteers aged 18-55 initially recruited into group 4a will receive a booster dose of the vaccine 4-6 weeks apart<br><br>Addition of process should a participant who wishes to continue in the trial, relocate and to an area with a study site; Inclusion of a mucosal immunity swabs in a subset of participants<br><br>Addition of optional stool samples; Clarification to AE grading table where not all A&E assessments should be

Amendment No.	Protocol Version No.	Date issued	Author(s) of changes	Details of Changes made
				recorded as SAEs; clarification on which PCR positive tests conducted outside the study procedures would be acceptable and included in primary endpoint analysis.<br>Change of PI at Cambridge site.
6	8.0	22 Jun 2020	Pedro Folegatti	Added day 42 visit in group 4b and increased the volume of serum taken. Clarification that the mucosal immunity assessments is to be done in a subset of group 6 individuals only.
9	9.0	20 Jul 2020	Pedro Folegatti, Merryn Voysey	Increased overall sample size; Added groups 5d (batch comparison group on Cobra material) and 9 and 10 (efficacy groups in participants aged 56 and above); Addition of diary completion for 7 days for groups 9 and 10 and 28 days for group 11; Groups 9 and 10 will be recommended to take paracetamol post vaccination; Removed participants aged 56 and above from groups 4 and 6; Added booster doses to groups 4 and 6; updated study endpoints to reflect comparisons of 1 vs 2 doses (groups 1, 2, 7 and 8); added information on Cobra material and product comparability and administration; updated primary efficacy analysis to reflect changes above; expanded the number of volunteers filling out diaries.; clarifications to holding/stopping rules; added Hy's law

Amendment No.	Protocol Version No.	Date issued	Author(s) of changes	Details of Changes made
				cases to be reported as SAEs; removed the requirement for SARS-CoV-2 serology prior to enrolment in groups 5d, 9 , 10 and 11.; added group 11 to recruit participants who previously received a ChAdOx1 vectored vaccine,; Paola Cicconi added as Investigator
Minor Amendment	9.1	31 Jul 2020	Pedro Folegatti,	Correction of errors with blood volumes for participants in groups 9, 10 and 11; Clarification of dose batches; clarification that the physical examination for group 5d is only if required.
10	10.0	06 Aug 2020	Pedro Folegatti, Merryn Voysey, Emma Plested, Hannah Robinson, Maheshi Ramasamy	Inclusion of D14 visit for group 11, changed swabbing pathway (S7 to be conducted only on positive cases, added S3-5 visit for second swab or home testing); an update to the 'Planned receipt of any vaccine other than the study intervention within 30 days before and after each study vaccination' exclusion criteria to allow an exception for the seasonal flu vaccine; clarifications made to visit time points; Re-consent may be collected with electronic signatures if required for infection control purposes; Confirmation that 15 minutes is a minimum time for post vaccine observations; clarification on the collection of AEs. ; addition of word 'boost' in group description; Clarification of the criteria for exclusion or delay of booster vaccination; Correction of length

Amendment No.	Protocol Version No.	Date issued	Author(s) of changes	Details of Changes made
				of study missed in previous amendment; Clarification of the process for information sharing following diagnosis with COVID-19; additional exclusion criteria for boosting doses to include AEs post prime that may affect the safety of the participant or the interpretation of the study results and SARS-CoV-2 PCR positivity within 4 weeks if symptomatic, or 2 weeks if asymptomatic; clarification to length of follow-up (i.e. 12 months from last vaccination); clarification to the process for sharing of information with public health authorities on COVID-19 positive cases; changes to the analysis procedures on the primary endpoint to reflect the new 2 dose schedule proposed
11	11.0	15 SEP 2020	Pedro Folegatti, Merryn Voysey, Andrew Pollard, Julie Fox	Clarifications to which groups are entitled to receive financial compensation; Clarification to efficacy objectives to include efficacy against severe disease; Clarification to exclusion criteria where only licensed seasonal influenza vaccines will be allowed within 30 days of vaccine administration, inclusion of HIV volunteers into sub-study, clarification to inclusion of participants with previous laboratory confirmed SARS-CoV-2 infection ; Clarifications to the statistical analysis section on primary, secondary and exploratory analysis; Clarifications to symptomatic pathway; Addition of boosting doses to groups 1a, 2a and 5a;

Amendment No.	Protocol Version No.	Date issued	Author(s) of changes	Details of Changes made
				Addition of HIV cohort sub-study; Correction of formatting and typographical errors; Changes to re-consent process to allow re-consent over the phone and electronic signatures when is not possible to have a physical visit;<br>.Updated exploratory immunology assays to reflect inclusion of HIV group (Group 12); Increase in time to vaccine administration from 4 to 6 hours; clarification to window for booster vaccinations.
12	12.0	20 – OCT -2020	K Emary	Clarification that home swabs may be processed outside UK; Clarification of the flow of information from home swabbing results to sponsor Clarification that stool may be collected at approximately 7 days post PCR positive result in those who are asymptomatic; Inclusion of CD4 count for screening of group 12; HIV serology is at investigators discretion for group 12; AstraZeneca have provided funding for some exploratory objectives; Clarification to exclusion criteria where licensed pneumococcal vaccines will be allowed within 7 days of study vaccine administration; increase in G12 sample volume to allow sufficient volume for exploratory objectives.

Amendment No.	Protocol Version No.	Date issued	Author(s) of changes	Details of Changes made
13 (SA16)	V13.0		Merryn Voysey<br>Pedro Folegatti	Updated statistical analysis section; statistician signature space;<br>Number of participants recruited in groups 9 and 10 will be 1000 +/- 10% each, to account for the multiple site recruitment activity and recognizing the potential for over recruitment. The overall sample size is unchanged.
14 (SA17)	V14.0		Maheshi Ramasamy, Emma Plested	Updated diagnostic PCR to NAAT (nucleic acid amplification assay) for purposes of endpoint definition; The number of diaries for Group 9 and 10 have been listed as approximately 500 in each group given the concurrent recruitment across multiple sites.

List details of all protocol amendments here whenever a new version of the protocol is produced.

17 Appendix

Investigator Agreement and Notification of Conflict of Interest

I approve this protocol for use in the above named clinical trial and agree to abide by all provisions set forth therein.

According to the Declaration of Helsinki, 2008, I have read this protocol, and declare no/~~the following (delete as appropriate)~~ conflict of interest

---

Chief Investigator Signature Date

Site: **Centre for Clinical Vaccinology and Tropical Medicine, University of Oxford**

I have read this protocol and agree to abide by all provisions set forth therein.

According to the Declaration of Helsinki, 2008, I have read this protocol, and declare the following conflict of interest. AH is a cofounder of and minor shareholder in an Oxford University spin-off company, Vaccitech Ltd, that has some non-exclusive rights to the vector, ChAdOx1, used in the vaccine to be tested, that may be of commercial value"

Principal Investigator<br><br>Prof Adrian Hill	Signature	Date:

Site: **NIHR WTCRF**

I have read this protocol and agree to abide by all provisions set forth therein.

According to the Declaration of Helsinki, 2008, I have read this protocol, and declare no/~~the following (delete as appropriate)~~ conflict of interest

Principal Investigator<br><br>Prof Saul Faust	Signature	Date:

Site: **NIHR Imperial CRF**

I have read this protocol and agree to abide by all provisions set forth therein.

According to the Declaration of Helsinki, 2008, I have read this protocol, and declare no/~~the following (delete as appropriate)~~ conflict of interest

Principal Investigator<br><br>Dr Katrina M. Pollock	Signature	Date:

Site: **Oxford University Hospital Foundation Trust**

I have read this protocol and agree to abide by all provisions set forth therein.

According to the Declaration of Helsinki, 2008, I have read this protocol, and declare no/~~the following (delete as appropriate)~~ conflict of interest

Principal Investigator<br><br>Dr Maheshi Ramasamy	Signature	Date:

Site: **St Georges University Hospital NHS Foundation Trust**

I have read this protocol and agree to abide by all provisions set forth therein.

According to the Declaration of Helsinki, 2008, I have read this protocol, and declare no/~~the following (delete as appropriate)~~ conflict of interest

Principal Investigator<br><br>Prof Paul Heath	Signature	Date:

Site: **University Hospitals Bristol and Weston NHS Foundation Trust**

I have read this protocol and agree to abide by all provisions set forth therein.

According to the Declaration of Helsinki, 2008, I have read this protocol, and declare no/~~the following (delete as appropriate)~~ conflict of interest

Principal Investigator<br><br>Prof Adam Finn	Signature	Date:

Site: **North Bristol NHS Trust**

I have read this protocol and agree to abide by all provisions set forth therein.

According to the Declaration of Helsinki, 2008, I have read this protocol, and declare no/~~the following (delete as appropriate)~~ conflict of interest

Principal Investigator<br><br>Dr Rajeka Lazarus	Signature	Date:

Site:
University of Nottingham Health Service and Nottingham University Hospitals NHS Trust

I have read this protocol and agree to abide by all provisions set forth therein.

According to the Declaration of Helsinki, 2008, I have read this protocol, and declare no/~~the following (delete as appropriate)~~ conflict of interest

Principal Investigator<br><br>Dr David Turner	Signature	Date:

Site: **Sheffield Teaching Hospitals**

I have read this protocol and agree to abide by all provisions set forth therein.

According to the Declaration of Helsinki, 2008, I have read this protocol, and declare no/~~the following (delete as appropriate)~~ conflict of interest

Principal Investigator<br><br>Dr. Thomas Darton	Signature	Date:

Site: **University Hospitals Birmingham NHS Foundation Trust (UHB)**

I have read this protocol and agree to abide by all provisions set forth therein.

According to the Declaration of Helsinki, 2008, I have read this protocol, and declare no/~~the following (delete as appropriate)~~ conflict of interest

Principal Investigator<br><br>Dr Christopher Green	Signature	Date:

Site: **Wales (Public Health Wales)**

I have read this protocol and agree to abide by all provisions set forth therein.

According to the Declaration of Helsinki, 2008, I have read this protocol, and declare no/~~the following (delete as appropriate)~~ conflict of interest

Principal Investigator<br><br>Dr Chris J Williams	Signature	Date:

Site: **Castle Hill Hospital**

I have read this protocol and agree to abide by all provisions set forth therein.

According to the Declaration of Helsinki, 2008, I have read this protocol, and declare no/~~the following (delete as appropriate)~~ conflict of interest

Principal Investigator<br><br>Dr Patrick Lillie	Signature	Date:

Site: **NHS Greater Glasgow & Clyde Hospitals**

I have read this protocol and agree to abide by all provisions set forth therein.

According to the Declaration of Helsinki, 2008, I have read this protocol, and declare no/~~the following (delete as appropriate)~~ conflict of interest

Principal Investigator<br><br>Professor Emma Thomson	Signature	Date:

Site: **Guy's and St Thomas' NHS Foundation Trust**

I have read this protocol and agree to abide by all provisions set forth therein.

According to the Declaration of Helsinki, 2008, I have read this protocol, and declare no/~~the following (delete as appropriate)~~ conflict of interest

Principal Investigator<br><br>Dr Anna Goodman	Signature	Date:

Site: **Liverpool School of Tropical Medicine (LSTM)**

I have read this protocol and agree to abide by all provisions set forth therein.

According to the Declaration of Helsinki, 2008, I have read this protocol, and declare no/~~the following (delete as appropriate)~~ conflict of interest

Principal Investigator<br><br>Dr Andrea Collins	Signature	Date:

Site: **The Newcastle upon Tyne Hospitals NHS Foundation Trust**

I have read this protocol and agree to abide by all provisions set forth therein.

According to the Declaration of Helsinki, 2008, I have read this protocol, and declare no/~~the following (delete as appropriate)~~ conflict of interest

Principal Investigator<br><br>Dr Christopher Duncan	Signature	Date:

Site: **UCLH**

I have read this protocol and agree to abide by all provisions set forth therein.

According to the Declaration of Helsinki, 2008, I have read this protocol, and declare no/~~the following (delete as appropriate)~~ conflict of interest

Principal Investigator<br><br>Prof Vincenzo Libri	Signature	Date:

Site: **NHS Lothian**

I have read this protocol and agree to abide by all provisions set forth therein.

According to the Declaration of Helsinki, 2008, I have read this protocol, and declare no/~~the following (delete as appropriate)~~ conflict of interest

Principal Investigator<br><br>Dr Rebecca Sutherland	Signature	Date:

Site: **Cambridge**

I have read this protocol and agree to abide by all provisions set forth therein.

According to the Declaration of Helsinki, 2008, I have read this protocol, and declare no/~~the following (delete as appropriate)~~ conflict of interest

Principal Investigator<br><br>Dr Mark Toshner	Signature	Date:

Site: **Northwick Park**

I have read this protocol and agree to abide by all provisions set forth therein.

According to the Declaration of Helsinki, 2008, I have read this protocol, and declare no/~~the following (delete as appropriate)~~ conflict of interest

Principal Investigator<br><br>Dr Alastair McGregor	Signature	Date:

**Study Title:**	A Randomized, Controlled, Phase III Study to Determine the Safety, Efficacy, and Immunogenicity of the Non-Replicating ChAdOx1 nCoV-19 Vaccine.

Study Reference: COV003

Protocol Version:	8.0
**Date:**	November 12th, 2020
**Chief Investigator:**	Prof. Andrew J Pollard
**Sponsor:**	University of Oxford
**Funder:**	OXFORD University and external donors (Fundação Lemann, Fundacao Brava, Fundacao Telles, Instituto D'or de Ensino e Pesquisa and AstraZeneca Brasil)

Main contacts of the study

Chief Investigator	Professor Andrew J Pollard
Address	Center for Clinical Vaccinology and Tropical Medicine<br>University of Oxford,<br>Churchill Hospital, Old Road, Headington<br>Oxford, OX3 7LE<br>Email: andrew.pollard@paediatrics.ox.ac.uk
Local Principal Investigator	Professor Lily Weckx<br>Centro de Referência para Imunobiológicos Especiais - CRIE<br>Universidade Federal de São Paulo - UNIFESP<br>Rua Borges Lagoa, 770 - Vila Clementino<br>São Paulo - SP, 04038-001, Brazil<br>Email: lily.crie@huhsp.org.br
Co-Principal Investigator – National Coordination	Professor Sue Ann Costa Clemens<br>Director of Oxford Vaccine Study – Brazil<br>Visiting Professor of Global Health – University of Oxford<br>Institute for Global Health - Siena University<br>Via Val Di Montone, 1<br>Siena, 53-100, Italy<br><br>Centro de Referência para Imunobiológicos Especiais - CRIE<br>Universidade Federal de São Paulo - UNIFESP<br>Rua Borges Lagoa, 770 - Vila Clementino<br>São Paulo - SP, 04038-001, Brazil<br>Email: sue.costa@unisi.it
Co-Investigators	Professor Sarah Gilbert<br>The Jenner Institute, University of Oxford

Old Road Campus Research Building (ORCRB)
Roosevelt Drive Oxford OX3 7DQ
Email: sarah.gilbert@ndm.ox.ac.uk

Professor Teresa Lambe
The Jenner Institute, University of Oxford
Old Road Campus Research Building (ORCRB)
Roosevelt Drive Oxford OX3 7DQ
Email: teresa.lambe@ndm.ox.ac.uk

Professor Adrian Hill
The Jenner Institute, University of Oxford
Old Road Campus Research Building (ORCRB)
Roosevelt Drive Oxford OX3 7DQ
Email: adrian.hill@ndm.ox.ac.uk

Dr. Alexander Douglas
The Jenner Institute, University of Oxford
Welcome Center for Human Genetics
Roosevelt Drive Oxford, OX3 7BN
Email: sandy.douglas@ndm.ox.ac.uk

Dr. Pedro Folegatti
Center for Clinical Vaccinology and Tropical Medicine
Churchill Hospital, Old Road, Headington
Oxford, OX3 7LE
Email: pedro.folegatti@ndm.ox.ac.uk

Study Sites

Centro de Referência para Imunobiológicos Especiais - CRIE
Universidade Federal de São Paulo - UNIFESP
Rua Borges Lagoa, 770 - Vila Clementino

São Paulo - SP, 04038-001, Brazil

Instituto D'Or de Pesquisa e Ensino - I'Dor

a. Rua Diniz Cordeiro, 30 - Botafogo
   Rio de Janeiro - RJ, 22281-100, Brazil
b. Avenida São Rafael, 2152 - São Marcos
   Salvador - BA, 41253-190, Brazil

Centro de Pesquisas Clinicas de Natal (CPCLIN)

Rua Dr Ponciano Barbosa, 282 – Cidade Alta

Natal – RN, 59025-050, Brazil

Universidade Federal de Santa Maria (UFSM)

Av. Roraima, 1000 Cidade Universitária Bairro Camobi

Santa Maria - RS, 97105-900, Brazil

Hospital das Clinicas de Porto Alegre

Universidade Federal do Rio Grande do Sul (UFRGS).

Rua Ramiro Barcelos, 2.350 Bairro Santa Cecília

Porto Alegre – RS, 90035-903, Brazil

Sponsor Institution	University of Oxford
Funder	OXFORD University and external donors (Fundação Lemann, Instituto D'or de Ensino e Pesquisa and AstraZeneca Brasil)

Confidentiality Statement

This document contains confidential information that should not be disclosed to anyone other than the Sponsor, the Research Team, the host organization, members of the Research Ethics Committee, and other regulatory bodies. This information may not be used for any purpose other than evaluating or conducting clinical research without the prior written consent of the Chief Investigator.

Compliance Statement

The study will be conducted in compliance with the Protocol, the principles of Good Clinical Practices, Standards for Medicines for Human Use (Clinical Trial) 2004 (as amended), and all other applicable regulatory requirements.

Investigator's Agreement and Conflict of Interest Notification

I approve this Protocol for use in the abovementioned clinical trial and agree to comply with all provisions established therein.

In accordance with Declaration of Helsinki, 2008, I have read this protocol, and declare that I have no conflict of interest.

Lead Investigator	Signature	Date

Research Site

I have read this protocol and agree to comply with all the provisions established therein.

In accordance with Declaration of Helsinki, 2008, I have read this protocol, and declare that I have no conflict of interest.

Principal Investigator Signature Date

TABLE OF CONTENTS

1 SYNOPSIS *12*

2 ABBREVIATIONS *16*

3 BACKGROUND AND RATIONALE *19*

3.1 Background **19**

3.2 Pre-clinical Studies **20**

3.2.1 Immunogenicity (Jenner Institute) 20

3.2.2 Efficacy 24

3.2.3 Immunopathology and antibody-dependent potentiation 24

3.3 Previous clinical experience **26**

3.4 Rationale **34**

4 OBJECTIVES AND ENDPOINTS *35*

5 STUDY DESIGN *36*

5.1 Study groups **40**

5.2 Study participants **41**

5.3 Definition of the End of Study **42**

5.4 Potential risks for participants **42**

5.5 Known potential benefits **44**

6 RECRUITMENT AND WITHDRAWAL OF STUDY PARTICIPANTS *44*

6.1 Identification of Participants **44**

6.2 Informed Consent Form **44**

6.3 Inclusion and exclusion criteria **46**

6.3.1 Inclusion Criteria 46

6.3.2 Exclusion Criteria 48

6.3.3 Re-vaccination exclusion criteria (two-dose groups only) 49
6.3.4 Effective birth control for volunteers 50
6.3.5 Withdrawal of participants 50
6.4 Pregnancy 51
7 CLINICAL PROCEDURES 52
7.1 Visit Schedule 52
7.2 Observations, medical history and physical examination 52
7.3 Blood samples, nose/throat swabs and urine analysis 52
7.4 Study visits 54
7.4.1 Screening visit and recruitment 54
7.4.2 Day 0: Recruitment and vaccination visit 55
7.4.3 Later visits 56
7.4.4 Symptomatic participants 64
7.4.5 Review of the medical record 65
7.4.6 Randomization, blinding, and unblinding 66
7.5 Description of ChAdOx1 nCoV-19 66
7.6 Storage 66
7.7 Administration 66
7.8 Rationale for selected dose 67
7.9 Environmental contamination control (GMO) 69
7.10 Control vaccine 69
7.11 Placebo 70
7.12 Compliance with the investigational treatment 71
7.13 Investigational treatment accounting 71

7.14 Concomitant medication 71

7.15 Provision of treatment for controls 72

8 SAFETY ASSESSMENT 72

8.1 Definitions 72

8.1.1 Adverse Event (AE) 72

8.1.2 Adverse Reaction (AR) 72

8.1.3 Serious Adverse Event (SAE) 73

8.1.4 Serious Adverse Reaction (SAR) 73

8.1.5 Suspected Unexpected Serious Adverse Reaction (SUSAR) 73

8.2 Expectation 74

8.3 Predicted/expected adverse reactions: 74

8.4 Adverse Events of Special Interest 74

8.5 Causality 75

8.6 Reporting procedures for all adverse events 76

8.7 Evaluation of severity 76

8.8 Serious AE reporting procedures 79

8.9 Procedures for reporting SUSARs 80

8.10 Safety assessments 80

8.11 Data Safety Monitoring Committee 81

9 STATISTICS 81

9.1 Description of statistical methods 81

9.2 Efficacy Outcomes 82

Regarding the differentiation of ADE and the lack of effectiveness of the vaccine: there is no internationally accepted definition of ADE. Differences in disease severity between groups will be

described. If the proportion of serious illness is similar between the two groups, this would support the lack of effectiveness and not the improvement of the illness. 82

9.2.1 Efficacy 82

9.2.2 Safety and Reactogenicity 84

9.2.3 Immunogenicity 84

The geometric means of concentration on day 28 and the proportion of participants with serum conversion to S-spike protein from day 0 to day 28 will be computed. Comparisons between the ChAdOx1 nCoV-19 vaccine and control groups will be made using a Mann Whitney U test due to the low titers expected in the control group that will cause non-normal distribution. 85

9.3 Subgroup analyses 85

9.4 Number of Participants 85

9.5 Interim and primary analyses of the primary outcome 85

9.6 Final Analysis 86

9.7 Inclusion in the analysis 86

9.8 Data and Safety Monitoring Committee 86

10 DATA MANAGEMENT 87

10.1 Data processing 87

10.2 Record keeping 87

10.3 Source data and technical data sheets (CRFs) 88

10.4 Data protection 88

10.5 Data quality 88

10.6 Archiving 89

11 QUALITY CONTROL PROCEDURES AND QUALITY ASSURANCE 89

11.1 Investigator's Procedures 89

11.2 Monitoring **89**

11.3 Deviation from the protocol **89**

11.4 Audit and inspection **90**

12 ETHICAL AND REGULATORY CONSIDERATIONS *90*

12.1 Declaration of Helsinki **90**

12.2 Guidelines for good clinical practices **90**

12.3 Ethical and regulatory approvals **90**

12.4 Volunteers confidentiality **91**

13 FUNDING AND INSURANCE *92*

13.1 Funding **92**

13.2 Insurance **92**

13.3 Publication Policy **92**

14 DEVELOPMENT OF A NEW PRODUCT/PROCESS OR INTELLECTUAL PROPERTY GENERATION *92*

1 SYNOPSIS

Title	A Randomized, Controlled, Phase III Study to Determine the Safety, Efficacy, and Immunogenicity of the Non-Replicating ChAdOx1 nCoV-19 Vaccine.
Study Identifier	COV003
Trial Record	https://www.isrctn.com/ (registration number: ISRCTN89951424)
Study Sites	Centro de Referência para Imunobiológicos Especiais (CRIE) - UNIFESP, Universidade Federal de São Paulo, Rua Borges Lagoa, 770, Vila Clementino, Zip Code 04038-001, São Paulo/SP, Brazil<br><br>Instituto D'Or de Pesquisa e Ensino<br>Rua Diniz Cordeiro, 30, Botafogo, Zip Code 22281-100, Rio de Janeiro - RJ, Brazil<br><br>Instituto D'Or de Pesquisa e Ensino<br>Avenida São Rafael, 2152, São Marcos, Zip Code 41253-190, Salvador - BA, Brazil<br><br>Centro de Pesquisas Clinicas de Natal (CPCLIN)<br>Rua Dr Ponciano Barbosa, 282 – Cidade Alta<br>Natal – RN, 59025-050, Brazil<br><br>Universidade Federal de Santa Maria (UFSM)<br>Av. Roraima, 1000 Cidade Universitária Bairro Camobi

	Santa Maria - RS, 97105-900, Brazil<br><br>Hospital das Clinicas de Porto Alegre<br>Universidade Federal do Rio Grande do Sul (UFRGS).<br>Rua Ramiro Barcelos, 2.350 Bairro Santa Cecília<br>Porto Alegre – RS, 90035-903, Brazil
Clinical Phase	3
Design	A Single-Blind, Randomized Study of Safety, Efficacy, and Immunogenicity.
Population	Health professionals and adults with high potential for exposure to SARS-CoV-2, aged ≥18 years.
Planned Sample Size	The total sample size will be up to 10,300 participants (with a margin of 1%).
Planned Duration	12 months post final vaccine, per participant

	Objective	Endpoint Measure
Primary Objective	To evaluate the efficacy of ChAdOx1 nCoV-19 vaccine against COVID-19 disease confirmed with PCR.	a) COVID-19 virologically confirmed symptomatic cases (PCR positive).

Secondary Objectives	To evaluate the safety, tolerability, and reactogenicity profile of ChAdOx1 nCoV-19 candidate vaccine.	a) Occurrence of signs and symptoms of local and systemic reactogenicity requested during 7 days after vaccination (in a subset of 200 participants*);<br>b) Occurrence of serious adverse events;<br>c) Occurrence of disease enhancement episodes .
	To evaluate the efficacy of ChAdOx1 nCoV-19 candidate vaccine against severe and non-severe COVID-19 disease.	a) Hospitalization for COVID-19 disease confirmed by PCR;<br>b) COVID-19 severe disease confirmed by PCR;<br>c) Death associated with COVID-19 disease;<br>d) Antibodies against SARS-CoV-2 non-Spike protein (efficacy against non-spike seroconversion rates).
	To assess the humoral immunogenicity of ChAdOx1 nCoV-19 candidate vaccine.	a) Antibodies against SARS-CoV-2 spike protein (sero-conversion rates).<br>b) Virus neutralizing antibodies (NAb) against live and/or pseudotyped SARS-CoV-2 virus.

	To assess the cellular immunogenicity of ChAdOx1 nCoV-19 candidate vaccine.	a) Interferon-gamma (IFN-γ) enzyme-linked immunospot (ELISpot) responses to SARS-CoV-2 spike protein;
Investigational Products	a) ChAdOx1 nCoV-19, an adenoviral vector of replication-defective monkey expressing the SARS-CoV-2 spike (S) protein;<br>b) MenACWY vaccine;<br>c) Saline Placebo (for the control arm boosting dose)	

*Detailed assessments of local and systemic reactogenicity for 7 days after vaccination with ChAdOx1 nCoV-19 compared to MenACWY as a control have been documented in a sufficient number of participants in previous studies. In study COV003, detailed local and systemic reactogenicity will be evaluated in 200 randomized participants, a quantity determined to ensure proportionality and comparative representativeness compared to studies COV001 and COV002.

Formulation	ChAdOx1 nCoV-19: Liquid
	MenACWY: powder and solvent for solution for injection
Route of Administration	Intramuscular (IM)
Doses per Administration	ChAdOx1 nCoV-19: 5 x 10^{10} vp
	ChAdOx1 nCoV-19: 0.5mL (3.5 x 10^{10} to 6.5 x 10^{10})
	MenACWY: 0.5 mL
	0.9% saline solution: 0.5mL
	Both groups will receive prophylactic paracetamol: 500mg - 1 g q6h/24 hours.

2 ABBREVIATIONS

AdHu	Human adenovirus
AdHu5	Human adenovirus serotype 5
ADE	Antibody-Dependent Enhancement
AE	Adverse Event
AESI	Adverse Event of Special Interest
AID	Autoimmune Disease
CCVTM	Centre for Clinical Vaccinology and Tropical Medicine, Oxford
CBF	Clinical Bio manufacturing Facility
ChAdOx	Chimpanzee adenovirus 1
CI	Confidence interval
COP	Code of Practice
CRF	Clinical Record or Clinical Research Facility
CTRG	Clinical Trials & Research Governance Office, University of Oxford
CTL	Cytotoxic T Lymphocyte
DSMB	Data and Safety Monitoring Board
DSUR	Development Safety Update Report
ELISPOT	Enzyme-linked immunospot
GCP	Good Clinical Practices
GMO	Genetically modified organism
GMT	Geometric mean titer
GP	General Practitioner
HCG	Human Chorionic Gonadotrophin
HEK	Human embryonic kidney
HLA	Human leukocyte antigen
HRA	Health Research Authority
IB	Investigator's Brochure
ICH	International Conference on Harmonization

ICMJE	International Committee of Medical Journal Editors
ICS	Intracellular Cytokine Staining
ID	Intradermal
IFNγ	Gamma interferon
IM	Intramuscular
IMP	Investigational medicinal product
IMP-D	Investigational Medicinal Product Dossier
IV	Intravenous
MenACWY	Quadrivalent meningococcal conjugate vaccine (protein-polysaccharide) against group A, C, W, and Y capsular serotype
MHRA	Medicines and Healthcare Products Regulatory Agency
MVA	Modified Vaccinia Ankara virus
NHS	National Health Service
NIH	National Institutes of Health
NIHR	National Institute for Health Research
PBMC	Peripheral blood mononuclear cell
PCR	Polymerase chain reaction
PI	Principal Investigator
QP	Qualified Person
qPCR	Quantitative polymerase chain reaction
REC	Research Ethics Committee
SAE	Serious adverse event
SC	Subcutaneous
SOP	Standard Operating Procedure
SUSAR	Suspected unexpected serious adverse reaction
µg	Microgram
VP	viral particle
VV	viral vector

WHO	World Health Organization

3 BACKGROUND AND RATIONALE

3.1 Background

In December 2019, a group of pneumonia patients of unknown cause was linked to a wholesale seafood market in Wuhan, China, and it was later confirmed that they were infected with a new coronavirus, known as 2019-nCoV [1]. The virus was later renamed SARS-CoV-2 because it is similar to the coronavirus responsible for severe acute respiratory syndrome (SARS-CoV), a betacoronavirus lineage B. SARS-CoV-2 shares more than 79% of its sequence with SARS-CoV, and 50% with the coronavirus responsible for Middle East respiratory syndrome (MERS-CoV), a member of betacoronavirus lineage C[2]. Covid-19 is the infectious disease caused by SARS-CoV-2. In January 2020, there was an increase in evidence of human-to-human transmission as the number of cases began to increase rapidly in China. Despite the unprecedented containment measures adopted by the Chinese government, SARS-CoV-2 has spread rapidly across the world. WHO declared the COVID-19 outbreak as an international public health emergency on January 30, 2020.

Coronaviruses (CoVs) are large, spherical, and enveloped single-stranded RNA genomes. A quarter of its genome is responsible for encoding structural proteins, such as glycoprotein spike (S), envelope (E), membrane (M) and nucleocapsid proteins (N). E, M, and N are mainly responsible for virion assembly, while protein S is involved in binding to the receptor, mediating the entry of the virus into host cells during CoVs infection through different receptors. [3] SARS-CoV-2 belongs to the phylogenetic lineage B of the genus *Betacoronavirus* and recognizes the angiotensin-converting enzyme 2 (ACE2) as an input receptor[4]. This is the seventh CoV that has been proven to cause infections in humans and the third that has been proven to cause serious illness after SARS-CoV and MERS-CoV.

The spike protein is a type I transmembrane, trimeric, type I glycoprotein located on the surface of the viral envelope of CoVs, which can be divided into two functional subunits: the N-terminal S1 and the C-terminal S2. S1 and S2 are responsible for the binding to the cellular receptor through the receptor binding domain (RBD, for its acronym in-English) and fusion of virus and cell membranes, respectively, thereby mediating the entry of SARS-CoV-2 into

target cells. [3] The functions of S in receptor binding and membrane fusion make it an ideal target for vaccine and antiviral development, as it is the main target for neutralizing antibodies.

ChAdOx1 nCoV-19 vaccine consists of a replication-deficient monkey adenoviral vector ChAdOx1, containing the SARS CoV-2 structural surface glycoprotein antigen (spike protein) (nCoV-19), with a signal sequence from the leading tissue plasminogen activator (tPA). ChAdOx1 nCoV-19 expresses a codon-optimized coding sequence for the Spike protein from GenBank genomic sequence access: MN908947. The leader tPA sequence was shown to be beneficial in increasing the immunogenicity of another CoV vaccine vectorized by ChAdOx1 (ChAdOx1 MERS)[5].

3.2 Pre-clinical Studies

Refer the Investigator's Brochure for the most recent update of preclinical data.

3.2.1 Immunogenicity (Jenner Institute)

The mice (balb/c and CD-1) were immunized with ChAdOx1 which expresses the SARS-CoV-2 Spike protein or green fluorescent protein (GFP). From 9 to 10 days after vaccination, spleen samples were used to assess IFN-γ ELISpot responses and serum samples for assessments of S1 and S2 antibody responses with ELISA. The results of these studies show that a single dose of ChAdOx1 nCoV was immunogenic in mice.

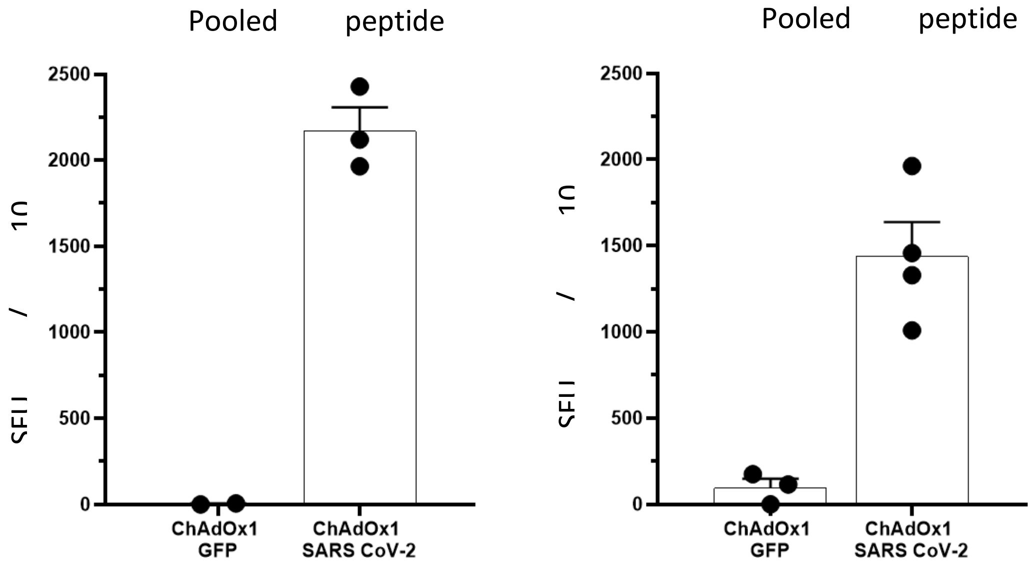

Figure 1. Splenic responses combined with IFN-γ ELISpot from BALB/c (left panel) and CD-1 (right panel) mice, in response to peptides that comprise the SARS-CoV-2 spike protein, nine or ten days after vaccination, with 1.7×10^{10} pv ChAdOx1 nCoV-19 or 8×10^{9} pv ChAdOx1 GFP. The means with SEM are described.

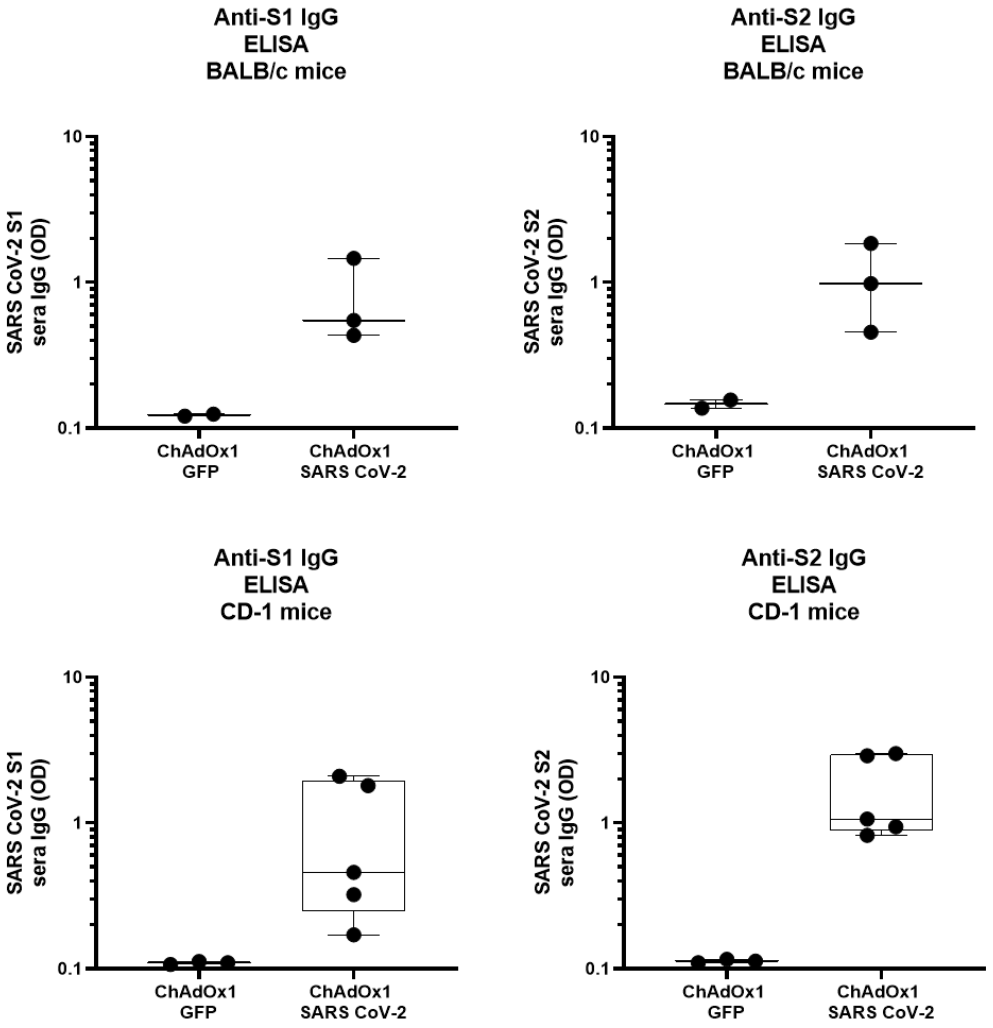

Figure 2. Box and mustache plot of optical densities after ELISA analysis of BALB/C mouse serum (top panel) incubated with purified protein spanning the S1 domain (left) or purified protein spanning the S2 domain (right) of the SARS-CoV spike -2 nine or ten days after vaccination, with 1.7 × 10^{10} pv ChAdOx1 nCoV-19 or 8 × 10^{9} pv ChAdOx1 GFP. Box and mustache plots of optical densities after ELISA analysis of CD-1 mouse serum (bottom panel) incubated with purified protein spanning the S1 domain (left) or purified protein spanning the S2 domain (right) of the SARS-CoV spike -2.

A second experiment was performed with a different dose. The results are summarized in the figure below. Intracellular cytokine staining shows a pattern consistent with predominantly Th1 responses.

A. ELISpot Responses

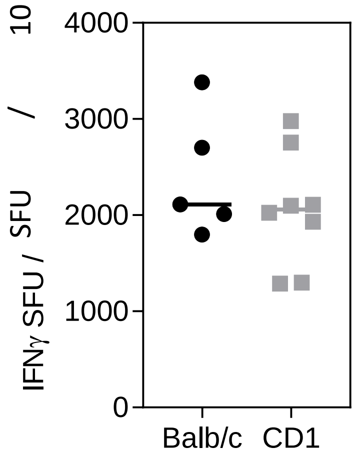

B. Intracellular cytokine staining

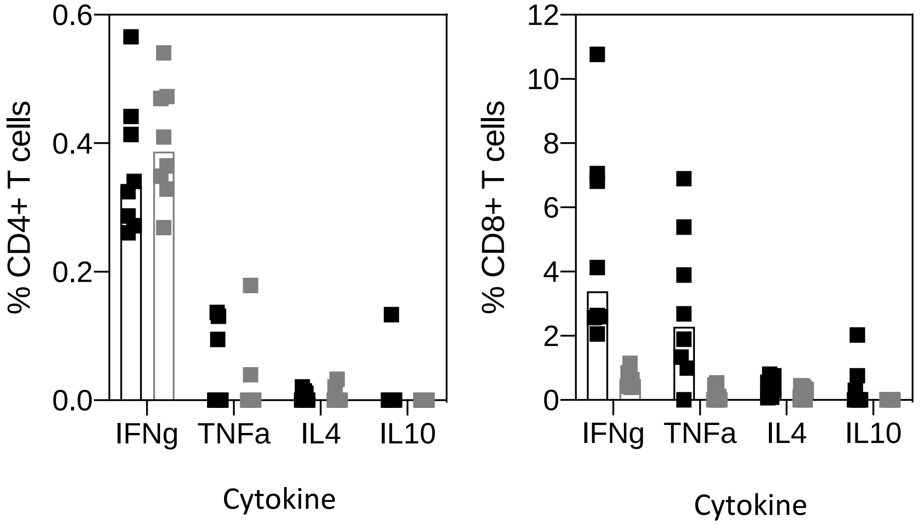

Figure 3. Specific antigen responses after vaccination with ChAdOx nCov19. 10^8 UI ChAdOx nCoV-19 was administered intramuscularly to heterogeneous BALB/c mice (CD1). Fourteen days later, the harvested spleens and cells stimulated peptides that span the extension of S1 and S2.

A. The graph shows the IFN-γ ELIspot responses summed up in BALB/c (black circles) and heterogeneous cd1 (gray squares) mice.

B. The graphs show the frequency of cytokine positive CD4 (left) or CD8 (right) cells, measured by intracellular cytokine staining after splenocyte stimulation with clustered S1 (black) or clustered S2 (gray) peptides in CD1 mice.

3.2.2 Efficacy

Pre-clinical efficacy studies of ChAdOx1 nCoV-19 in ferrets and non-human primates are ongoing. Once available, the results will be included in the Investigator's Brochure.

3.2.3 Immunopathology and antibody-dependent potentiation

Safety concerns have arisen around the use of coronavirus Spike glycoproteins to their full extent and other viral antigens (nucleoprotein) as vaccine antigens after historical and limited reports of immunopathology and antibody dependent potentiation (ADE) reported *in vitro* and post challenge with SARS-CoV in mice, ferrets and non-human primates immunized with vaccines based on inactivated complete SARS-CoV or protein S in its full extension, including a study that used modified Vaccinia Ankara as a vector. [6-8] So far, there has been a report of pulmonary immunopathology after the challenge with MERS-CoV in mice immunized with a candidate vaccine against inactivated MERS-CoV. [9] However, in preclinical trials of immunization with ChAdOx1 and challenge with MERS-CoV, ADE was not observed in hDPP4 transgenic mice, dromedary camels or non-human primates (van Doremalen et al, submitted manuscript).[10.11]

The risks of inducing pulmonary immunopathology in the case of COVID-19 after vaccination with ChAdOx1 nCoV-19 are unknown. The NHP study conducted by NIH described in the investigator's brochure showed no evidence of immune-enhanced inflammation in ChAdOx1 nCoV-19 vaccinated animals who underwent SARS-CoV-2 challenge 4 weeks post immunization, at 7 days post challenge. Results from a separate challenge study conducted on a purified inactivated SARS-CoV-2 vaccine also corroborate with NIH findings where no ADE has been detected in vaccinated animals [1]. However, the negative findings on ADE and lung immunopathology from both reports should be interpreted with caution, as challenged animals were sacrificed and examined shortly after challenge (7 days post inoculation).

Challenge studies in ferrets and non-human primates (PNH) are ongoing and these pre-clinical trials will report on the presence or absence of pulmonary pathology. The results will be reviewed as soon as they are available and will be part of the risk/benefit discussions for participants who receive the investigational product (IMP). All pathological data from challenge studies of other SARS-CoV-2 candidate vaccines will also be taken into account.

3.3 Previous clinical experience

Prior to current studies, vaccines vectorized by ChAdOx1 that express different inserts have been used previously in more than 320 healthy volunteers participating in clinical trials conducted by University of Oxford in the United Kingdom and abroad (tables 1 and 2). Most importantly, a ChAdOx1 vector vaccine expressing the total Spike protein from another Betacoronavirus, MERS-CoVCoV, has been administered to 31 participants, so far, as part of the MERS001 and MERS002 studies. ChAdOx1 MERS was administered in doses ranging from $5x10^9$ pv $5x10^{10}$ pv (table 2) with no serious adverse reactions reported. Further references and results on safety and immunogenicity about ChAdOx1 MERS can be found in the ChAdOx1 Investigator's Brochure nCoV-19.

Clinical trials of ChAdOx1 vectorized vaccines encoding antigens for Influenza (NP + M1 fusion protein), Tuberculosis (85A), Prostate Cancer (5T4), Malaria (LS2), Chikungunya (structural polyprotein), Zika (prM and E) , MERS-CoV (total spike protein) and Meningitis B are listed below.

None of the clinical trials mentioned below reported serious adverse events associated with the administration of ChAdOx1, which had a good safety profile.

Phase I/II testing of ChAdOx1 nCov19 vaccine is currently ongoing in the United Kingdom. Since 4/May/2020, 600 volunteers have been vaccinated.

Table 1. Clinical experience with ChAdOx1 viral vector vaccines.

Country	Study	Vaccine	Age	Route	Dose	Number of volunteers (received ChAdOx1)	Publication/Registration Number
UK	FLU004	ChAdOx1 NP + M1	18-50	IM	$5x10^8$ pv	3	Antrobus et al, 2014. Molecular Therapy. DOI: 10.1038/mt. 2013.284 [2]
					$5x10^9$ pv	3	
					$2.5x10^{10}$ pv	3	
					$5x10^{10}$ pv	6	
UK	FLU005	ChAdOx1 NP + M1 MVA NP + M1 (week 8)	18-50	IM	$2.5x10^{10}$ pv	12	Coughlan et al, 2018. EBioMedicine DOI: 10.1016/j.ebiom.2018.02.011 DOI: 10.1016/j.ebiom.2018.05.001 [3]
		ChAdOx1 NP + M1 MVA NP + M1 (week 52)	18-50	IM	$2.5x10^{10}$ pv	12	
		MVA NP+M1 ChAdOx1 NP + M1 (week 8)	18-50	IM	$2.5x10^{10}$ pv	12	

Country	Study	Vaccine	Age	Route	Dose	Number of volunteers (received ChAdOx1)	Publication/Registration Number
		MVA NP+M1<br>ChAdOx1 NP + M1 (week 52)	18-50	IM	$2.5x10^{10}$ pv	9	
		ChAdOx1 NP + M1	>50	IM	$2.5x10^{10}$ pv	12	
		ChAdOx1 NP + M1<br>MVA NP + M1 (week 8)	>50	IM	$2.5x10^{10}$ pv	12	
		ChAdOx1 85A	18-50	IM	$5x10^{9}$ pv	6	Wilkie et al, 2020 Vaccine
					$2.5x10^{10}$ pv	12	
UK	TB034	ChAdOx1 85A<br>MVA85A (week 8)	18-50	IM	$2.5x10^{10}$ pv	12	
		ChAdOx1 85A<br>(x2, 4 weeks apart)	18-50	IM	$2.5x10^{10}$ pv	12	

Country	Study	Vaccine	Age	Route	Dose	Number of volunteers (received ChAdOx1)	Publication/Registration Number
		MVA85A (in 4 months)					
Switzerland	TB039 (ongoing)	ChAdOx1 85A	18-55	Aerosol	$1x10^9$ pv	3	Clinicaltrials.gov: NCT04121494
				Aerosol	$5x10^9$ pv	3	
				Aerosol	$1x10^{10}$ pv	11	
				Aerosol/IM	$1x10^{10}$ pv	15	
Uganda	TB042 (ongoing)	ChAdOx1 85A	18-49	IM	$5x10^9$ pv	6	Clinicaltrials.gov: NCT03681860
					$2.5 x10^{10}$	6	
UK	VANCE01	ChAdOx1.5T4 MVA.5T4	18 - 75	IM	$2.5x10^{10}$ pv	34	Clinicaltrials.gov: NCT02390063
UK	ADVANCE (ongoing)	ChAdOx1.5T4 MVA.5T4	≥18	IM	$2.5x10^{10}$ pv	23 (on Feb 20)	Clinicaltrials.gov: NCT03815942

Country	Study	Vaccine	Age	Route	Dose	Number of volunteers (received ChAdOx1)	Publication/Registration Number
UK	VAC067	ChAdOx1 LS2	18-45	IM	$5x10^9$ pv	3	Clinicaltrials.gov: NCT03203421
					$2.5x10^{10}$ pv	10	
UK	VAMBOX	ChAdOx1 MenB.1	18-50	IM	$2.5x10^{10}$ pv	3	ISRCTN46336916
					$5x10^{10}$ pv	26	
UK	CHIK001	ChAdOx1 Chik	18-50	IM	$5x10^9$ pv	6	Clinicaltrials.gov: NCT03590392 DOI: https://doi.org/10.4269/ajtmh.abstract2019 Abstract # 59, page 19.
					$2.5x10^{10}$ pv	9	
					$5x10^{10}$ pv	9	
UK	ZIKA001 (ongoing)	ChAdOx1 Zika	18-50	IM	$5x10^9$ pv	6	Clinicaltrials.gov: NCT04015648
					$2.5x10^{10}$ pv	3 (on Feb 20)	
					$5x10^{10}$ pv	-	

Table 2. Clinical experience with ChAdOx1 MERS

Country	Study	Vaccine	Age	Route	Dose	Number of volunteers (received ChAdOx1)	Publication/Registration Number
UK	MERS001 (ongoing)	ChAdOx1 MERS	18-50	IM	$5x10^9$ pv<br>$2.5x10^{10}$ pv<br>$5x10^{10}$ pv<br>$2.5x10^{10}$ pv Initiation - homologous reinforcement	6<br>9<br>9<br>3	Clinicaltrials.gov: NCT03399578<br>Folegatti et.al. 2020, Lancet Infect.Dis<br>DOI: https://doi.org/10.1016/S1473-3099(20)30160-2
Saudi Arabia	MERS002 (ongoing)	ChAdOx1 MERS	18-50	IM	$5x10^9$ pv<br>$2.5x10^{10}$ pv<br>$5x10^{10}$ pv	4<br>3<br>-	Clinicaltrials.gov: NCT04170829

The first clinical trial of the ChAdOx1 nCoV-19 candidate vaccine started on April 23, after approval by the IRB and CTA by the MHRA. The study included healthy adults between the ages of 18 and 55 at various research sites in the UK. The objectives of the study are to assess vaccine

safety, reactogenicity and immunogenicity, as well as the collection and analysis of any confirmation of COVID-19 by PCR. These cases will be analyzed in a meta-analysis, together with case collections from other studies (methodology still under review). The study involved approximately 1070 individuals who are in the follow-up period. The Independent Safety Data Monitoring Committee (DSMB), which continuously monitors the study, has so far not reported any concerns to the MHRA or the study sponsor (secure follow-up of one to four weeks per participant). Safety follow-up for the full four weeks will be available to a subset of participants prior to the application of the first vaccine in Brazil.

In addition, before the start of the COV003 study in Brazil, another phase II study, COV002, will be initiated at various locations in the United Kingdom. In a first stage, COV002 will include 80 healthy adults from 56 to 69 years old, 120 elderly people over 70 with no upper age limit and 60 children from 5 to 12 years old. The assessed endpoints will be safety and immunogenicity, including T cell immunity. This study will be expanded in stage 2 to a phase III study of safety, immunogenicity and efficacy, including 10,000 adults over 18 years of age, with an increased risk of infection by COVID-19 at various research sites in the United Kingdom. The safety and efficacy assessments of the phase III part of the British study COV002 are the same as those of the COV003 protocol in Brazil. This will allow for the eventual grouping of effectiveness data between studies.

3.4 Rationale

The epidemic of COVID-19 has caused a major disruption in health systems with significant socioeconomic impacts. Containment measures have failed to stop the spread of the virus, which has reached pandemic levels. Currently, there are no specific treatments available against COVID-19 and accelerated vaccine development is sorely needed.

Live attenuated viruses have historically been one of the most immunogenic platforms available, as they have the ability to present multiple antigens throughout the viral life cycle in their native conformations. However, the manufacture of live attenuated viruses requires complex measures of containment and biosafety. In addition, live attenuated viruses carry the risk of inadequate attenuation, causing widespread disease, particularly in immunocompromised hosts. Given that COVID-19 is a serious and potentially fatal disease, which disproportionately affects elderly people with comorbidities, producing a vaccine with live attenuated viruses is the least viable option. The replication of competent viral vectors may pose a similar threat in relation to the disease spread in immunosuppressed individuals. Vectors with poor replication, however, avoid this risk by maintaining the advantages of the presentation of native antigens, increased immunity of T cells and the ability to express multiple antigens[15]. Subunit vaccines generally require the use of adjuvants and, although DNA and RNA vaccines can offer manufacturing advantages, they are often precariously immunogenic and require multiple doses, which is highly undesirable in the context of a pandemic.

Chimpanzee adenovirus vaccine vectors have been safely administered to thousands of people targeting a wide range of infectious diseases. ChAdOx1 vectorized vaccines were administered to more than 320 volunteers without safety concerns and were shown to be highly immunogenic with the administration of single doses. Relevant information refers to recent clinical trials where a single dose of a vectorized ChAdOx1 vaccine expressing the total spike protein of another beta-choronavirus (MERS-CoV) has been shown to induce neutralizing antibodies.

The use of an active vaccine as a comparator for the control group will minimize the chances of accidentally unblinding the participant, reducing the bias in the analysis of reactogenicity,

in the safety report and/or changes in the search for health services once that were symptomatic for COVID-19.

Groups 1c and 1d have been added following interim immunogenicity results on homologous prime-boost groups (as part of the COV001 UK study – see investigator's brochure for further details) showing improved neutralising antibody titres after 2 doses when compared to 1 dose regimen. A saline placebo will be used in place of an active comparator in group 1d. As we have seen less reactogenicity when giving the booster dose in the UK studies, there is less risk of unblinding the participant by using placebo as a comparator for the booster dose.

4 OBJECTIVES AND ENDPOINTS

	Objective	Endpoint Measure
Primary Objective	To evaluate the efficacy of ChAdOx1 nCoV-19 vaccine against COVID-19 disease confirmed with PCR.	a) COVID-19 virologically confirmed symptomatic cases (PCR positive).
Secondary Objectives	To evaluate the safety, tolerability and reactogenicity profile of ChAdOx1 nCoV-19 candidate vaccine.	a) Occurrence of signs and symptoms of local and systemic reactogenicity requested during 7 days after vaccination (in a subset of 200 participants*);<br>b) Occurrence of serious adverse events;<br>c) Occurrence of disease enhancement episodes
	To evaluate the efficacy of ChAdOx1 nCoV-19 candidate vaccine against severe and non-severe COVID-19 disease	a) Hospitalization for COVID-19 disease confirmed by PCR;<br>b) COVID-19 severe disease confirmed by PCR;

		c) Death associated with COVID-19 disease;<br>d) Antibodies against SARS-CoV-2 non-Spike protein (efficacy against non-spike seroconversion rates).
	To evaluate the humoral immunogenicity of ChAdOx1 nCoV-19.	a) Antibodies against the SARS-CoV-2 spike protein (sero-conversion rates);<br>b) Virus neutralizing antibodies (NAb) against live and/or pseudotyped SARS-CoV-2 virus.
	To assess the cellular immunogenicity of ChAdOx1 nCoV-19 candidate vaccine**.	a) Interferon-gamma (IFN-γ) enzyme-linked immunospot (ELISpot) responses to SARS-CoV-2 spike protein;

*Detailed assessments of local and systemic reactogenicity for 7 days after vaccination with ChAdOx1 nCoV-19 compared to MenACWY as a control have been documented in a sufficient number of participants in previous studies. In study COV003, detailed local and systemic reactogenicity will be evaluated in 200 randomized participants, a quantity determined to ensure proportionality and comparative representativeness compared to studies COV001 and COV002.

** Cellular immune responses will be measured in a subset of individuals only (up to 60 volunteers who will be recruited from the CRIE-UNIFESP site, sequentially)

5 STUDY DESIGN

This is a phase III, controlled, randomized, single-blind study to be conducted in adults with high exposure to COVID-19, who are administered two-doses of ChAdOx1 nCoV-19 or MenACWY and saline placebo by means of an IM injection with co-administered paracetamol.

After reviewing all available data from animal studies and UK studies (COV001 and COV002), participants will be randomized to ChAdOx1 nCoV-19 or MenACWY vaccine in a 1:1 ratio in blocks of 4, and all participants will be blinded to the allocation of the vaccine groups. The DSMB will periodically evaluate safety and efficacy data, every 4-8 weeks and/or as needed. The DSMB will consist of the members of the DSMB currently convened who oversee trials in the UK.

Following the immunogenicity results of the UK phase I/II study which showed higher levels of neutralizing antibodies with a prime-boost schedule (https://doi.org/10.1016/S0140-6736(20)31604-4), a booster dose of vaccine will be offered to all participants in the study. Participants enrolled on version 4.0 of the protocol onwards will only be allowed to take part in the study if they agree to receive 2 doses of either ChAdOx1 nCoV-19 or MenACWY/saline placebo. Participants who already received a dose of either ChAdOx1 nCoV-19 or MenACWY (before approvals for the second dose were in place) will be offered a booster dose 4-12 weeks after the prime dose of either ChAdOx1 nCoV-19 or saline placebo, depending on which arm they were originally allocated to. Any volunteers enrolled prior to the booster dose protocol amendment will be able to refuse a second dose and will continue their follow-up as per their previously agreed schedule of attendances.

Participants will be followed for the duration of the study to record adverse events and episodes of symptomatic COVID-19 confirmed by PCR. Participants will be assessed for COVID-19 if they have a new fever (≥ 37.8 °C) OR cough OR shortness of breath OR anosmia/ageusia.

Moderate and severe COVID-19 disease will be defined by clinical criteria. Detailed clinical parameters will be collected from medical records and aligned with the definitions of moderate and severe disease agreed by the international scientific community, such as a score greater than 6 on the NEWS-2 scale, or a score of 4 and above on ISARIC/WHO (International Severe Acute Respiratory and Emerging Infection Consortium; WHO Working Group on Clinical Characterisation and Management of COVID-19). Such parameters include, but are not limited to oxygen saturation, need for oxygen therapy, respiratory rate, and other vital signs, need for ventilatory support, radiographic and computed tomography, and blood test results, among other clinically relevant parameters. Considering that the NEWS-2 scale is

not used in the clinical routine of the research site, all staff involved in conducting the project will receive specific training.

NEWS-2 scoring system for serious COVID-19 assessment:

				Score			
Physiological parameter	3	2	1	0	1	2	3
Breathing rate (per minute)	≤8		9–11	12–20		21–24	≥25
SpO2 Scale 1 (%)	≤91	92–93	94–95	≥96			
SpO2 Scale 2 (%) - use in hypercapnic respiratory failure	≤83	84–85	86–87	88–92 ≥93 in the air	93–94 in oxygen	95–96 in oxygen	≥97 in oxygen
Air or oxygen?		Oxygen		Air			
Systolic blood pressure (mmHg)	≤90	91–100	101–110	111–219			≥220
Pulse (per minute)	≤40		41–50	51–90	91–110	111–130	≥131
Consciousness				Alert			CVPU
Temperature (°C)	≤35.0		35.1–36.0	36.1–38.0	38.1–39.0	≥39.1	

Patient State	Descriptor	Score
Uninfected	Uninfected; no viral RNA detected	0
Ambulatory mild disease	Asymptomatic; viral RNA detected	1
	Symptomatic; independent	2
	Symptomatic; assistance needed	3
Hospitalised: moderate disease	Hospitalised; no oxygen therapy*	4
	Hospitalised; oxygen by mask or nasal prongs	5
Hospitalised: severe diseases	Hospitalised; oxygen by NIV or high flow	6
	Intubation and mechanical ventilation, $pO_2/FiO_2 \geq 150$ or $SpO_2/FiO_2 \geq 200$	7
	Mechanical ventilation $pO_2/FIO_2 < 150$ ($SpO_2/FiO_2 < 200$) or vasopressors	8
	Mechanical ventilation $pO_2/FiO_2 < 150$ and vasopressors, dialysis, or ECMO	9
Dead	Dead	10

WHO clinical progression scale

ECMO=extracorporeal membrane oxygenation. FiO2=fraction of inspired oxygen. NIV=non-invasive ventilation. pO2=partial pressure of oxygen. SpO2=oxygen saturation. *If hospitalised for isolation only, record status as for ambulatory patient.

5.1 Study groups

Vaccine	Number of Participants	Participants
1a) Single dose of ChAdOx1nCOV19 vaccine, $5x10^{10}$ vp + paracetamol	N = up to 1600 participants	Health professionals and adults with high known likely exposure to COVID-19.

1b) Single dose of MenACWY + paracetamol	N = up to 1600 participants	Health professionals and adults with high known likely exposure to COVID-19.
1c) Two doses of ChAdOx1 nCoV-19 vaccine, $5x10^{10}$vp (prime) and 0.5mL boost (3.5 – 6.5 × 10^{10} vp), 4-12 weeks apart + paracetamol	N= up to 5150 (up to 1600 invited from 1a to receive a booster dose and new volunteers recruited)	Participants recruited in group 1a will be invited to receive a booster dose, 4-12 weeks apart) and new participants recruited on version 4.0 of the protocol onwards will consent to receive a 2-dose schedule.
1d) MenACWY prime, and Saline Placebo boost (0.5mL)), 4-12 weeks apart + paracetamol.	N= up to 5150 (up to 1600 invited from 1b to receive a booster dose and new volunteers recruited)	Participants recruited in group 1b will be invited to receive a booster dose, 4-12 weeks apart) and new participants recruited on version 4.0 of the protocol onwards will consent to receive a 2-dose schedule.

The overall sample size will be up to 10,300 (with a margin of 1%) participants. All volunteers previously enrolled in groups 1a and 1b will be offered a booster dose. Any new participants recruited into the study on version 4.0 of the protocol onwards will necessarily have to consent to a 2-dose schedule.

5.2 Study participants

Adult participants over the age of 18 will be enrolled in the study. Participants will be considered enrolled immediately after the vaccine is administered. Recruitment will focus on healthcare professionals and those with likely high known exposure to COVID-19. For example, they are health professionals: students, residents and professionals who perform health care activities such as nurses and nursing technicians, pharmacists, doctors, physiotherapists, speech therapists and radiology technicians. High exposure adults will be

considered: cleaning and hygiene personnel; safety; reception and concierge; volunteers; drivers, among others. Participants in older age groups (56-69 years and 70 years and above) will be recruited at the investigators' discretion. Their likelihood of COVID-19 exposure will be judged on a case-by-case basis, regardless of their previous occupation.

5.3 Definition of the End of Study

The end of study is the date of the last test performed on the last sample collected.

5.4 Potential risks for participants

Potential risks are those associated with phlebotomy, vaccination, and disease potentiation.

Venipuncture

Hematomas and discomfort located at the venipuncture site may occur. More rarely. Fainting may occur. These will not be documented as AE if they occur. The total volume of blood drawn during the study period will be approximately 330 mL, excluding any necessary repeated extraction (blood volumes may vary slightly between participants due to the use of different volume vacutainers, the operational procedures of the research sites and the number of symptomatic visits). Participants will be asked to refrain from donating blood during the period of their involvement in the study.

Allergic reactions

Mild to severe allergic reactions can occur in response to any component of a drug preparation. Anaphylaxis is extremely rare (about 1 in 1,000,000 doses of vaccine), but it can occur in response to any vaccine or medication.

Vaccination

Local reaction due to IM vaccination

The typical local reaction as a result of the IM injection is temporary pain, tenderness, redness and swelling at the injection site.

Systemic reactions

Constitutional symptoms similar to the flu, such as fatigue, headache, malaise, feverish sensation and muscle pain can occur with any vaccination and last from 2 to 3 days. Pre-syncopal and syncopal episodes may occur at the time of vaccination. Participants will be asked to administer prophylactic paracetamol for 24 hours to minimize possible fever and flu-like symptoms. As with many vaccines, temporary ascending paralysis (Guillain-Barré syndrome, GBS) or immune-mediated reactions can occur that can lead to organ damage, but this should be extremely rare (1 in 100,000-1,000,000 doses vaccine).

Transient neutropenia and thrombocytopenia have been described after immunization with other adenoviral vectorized vaccines, but it is not perceived as clinically significant.

Control group participants will receive a single dose of an authorized MenACWY vaccine, the risks of which are described in the vaccine package insert.

Disease Enhancement

The risks of inducing disease enhancement and pulmonary immunopathology in case of COVID-19 disease after vaccination with ChAdOx1 nCoV-19 are unknown. Challenge studies on ferrets and PNH are ongoing and the results will be reviewed as they arise. Two studies on PNH so far have shown no evidence of disease potentiation until day 7 after the challenge. All preclinical data from challenge studies using ChAdOx1 nCoV-19 and other candidate vaccines (where available) will guide risk/benefit decisions for participants who receive the IMP. Any

safety signs associated with the enhancement of the disease potentially observed in COV001/COV002 will also guide these decisions.

5.5 Known potential benefits

Participants enrolled in the control groups will receive MenACWY, a licensed vaccine that has been administered to adolescents on routine vaccination schedules in several countries, including the United Kingdom, and is used as a travel vaccine for high-risk areas. Most of the participants in this study will not have received this vaccine before and therefore will gain the benefit of protection against meningococci in groups A, C, W and Y. Participants who had previously been vaccinated with MenACWY will have their immunity enhanced against these organisms and are not exposed to additional risks when receiving the additional dose of the comparator vaccine. Participants receiving ChAdOx1 nCoV-19 will have no guaranteed benefit, however, it is expected that the information obtained in this study will contribute to the development of a safe and effective vaccine against COVID-19. All participants will obtain information about their general health.

If the effectiveness of the vaccine against COVID-19 is proven, after the analysis of the effectiveness results and approval by the Data and Safety Monitoring Committee, the sponsor will make this vaccine available to the research participants who received the control vaccine, MenACWY.

6 RECRUITMENT AND WITHDRAWAL OF STUDY PARTICIPANTS

6.1 Identification of Participants

Participants can be recruited through advertisements approved by the local Ethics Committee. The leaflets will be distributed, including the name of the study, information from the centers, age group, disease, and vaccine.

6.2 Informed Consent Form

The participant will sign and date personally or electronically the last approved version of the Informed Consent Form. When the process is carried out in person, it will be presented to the participants, individually, a written version and verbal explanation of the Informed Consent

Form. When the process is carried out electronically, participants will individually receive a link to access the electronic version of the Informed Consent Form. In both cases, it will be detailed:

• the exact nature of the study;

• what it will imply for the participant;

• the implications and restrictions of the protocol;

• the known side effects and any risks involved in participating;

• sample manipulation - participants will be informed about the samples that will be collected anonymously during the course of the study and that can be shared with the study collaborators;

• that individual results will not be shared with participants.

The Informed Consent Form will be made available to the participant before obtaining consent. However, participants will have the opportunity to individually question a properly trained and delegated researcher before signing the consent.

The following general principles will be emphasized:

- Participation in the study is completely voluntary;
- Refusal to participate does not involve a penalty or loss of medical benefits;
- The participant can withdraw from the study at any time;
- The participant is free to ask questions at any time to understand the purpose of the study and the procedures involved;
- The study involves researching an investigational vaccine;
- There is no direct benefit expected for the participant;
- The general practitioner/personal physician of the participant can be contacted to corroborate his/her medical history. Written or verbal information about the participant's medical history can be requested from the general practitioner/personal physician or other sources;
- Blood samples taken as part of the study can be sent abroad, to the United Kingdom, to laboratories at the University of Oxford. These will not be identified. Participants

will be asked whether they agree to biorepository storage for future use, but this will be optional.

The participant will have as much time as he/she wishes to consider the information and the opportunity to question the Investigator, his/her clinical physician or other independent parties to decide whether to participate in the study. The electronic Informed Consent Form must be signed and dated electronically by the adult participant, and the printed Informed Consent Form must be signed and dated by the adult participant and by the person responsible for obtaining the informed consent. This person must be suitably qualified and experienced, have been authorized to do so by the Lead/Principal Investigator and be listed on the delegation´s record. In the case of the printed Informed Consent Form, a copy of the signed document will be given to the participant. The signed original document will be retained at the research study sites.

6.3 Inclusion and exclusion criteria

This study will be performed in healthy adults, who meet the following inclusion and exclusion criteria:

6.3.1 Inclusion Criteria

The participant must meet all the following criteria to be eligible for the study:

- Adults from 18 to 55 years of age.
- Adults aged 56-69 years old (after review of safety data by DSMB in this age group in the UK trial)
- Adults aged 70 and above years old (after review of safety data by DSMB in this age group in the UK trial)
- Able and willing (in the Investigator's opinion) to fulfill all study requirements;
- Health professionals and/or adults at high risk of exposure to SARS-CoV-2, as defined in section 5.2 of this protocol;
- Serology with SARS-CoV-2 negative IgG antibodies; This inclusion criteria does not apply to participants enrolled from version 4.0 of the protocol onwards.

- Willing to allow investigators to discuss the participant's clinical history with their GP/personal physician and access medical records relevant to the study procedures;
- Only for women of childbearing age willing to practice continuous effective birth control (see below) during the study, and a negative pregnancy test on the screening and vaccination day(s);
- Consent to abstain from blood donation during the course of the study;
- Provide informed consent in writing.

The DSMB will review safety data from volunteers aged 56 and above recruited as part of the COV002 UK study. Recruitment of older adults will only be allowed following advice from the DSMB.

6.3.2 Exclusion Criteria

The participant will not be eligible for the study if any of the following criteria apply:

- Participation in trials of prophylactic drugs for COVID-19 during the course of the study;

  Note: Participation in COVID-19 treatment trials is permitted in case of hospitalization due to COVID-19, after confirmation of positive PCR. The study team should be informed as soon as possible. Participants with COVID-19 not hospitalized with positive PCR results for COVID-19 may be medicated according to standard clinical practice, however, participation in treatment trials will not be allowed.

- Planned receipt of any vaccine (authorized or investigational), within 30 days before and after vaccination;
- Prior receipt of an investigational or licensed vaccine with the possibility of impacting the interpretation of the study data (for example, Adenovirus vector vaccines , any vaccines against coronavirus);
- Administration of immunoglobulins and/or any blood products in the three months prior to the planned administration of the candidate vaccine;
- Any confirmed or suspected immunosuppressive or immunodeficiency state, including HIV (regardless of treatment, CD4 count or viral load status); asplenia; severe recurrent infections and chronic use (more than 14 days) of immunosuppressive medication in the last 6 months, except for topical steroids or short-term oral steroids (cycle lasting ≤14 days);
- History of allergic disease or reactions possibly exacerbated by any component of ChAdOx1 nCoV-19 or MenACWY or paracetamol;
- Any history of angioedema;
- Any history of anaphylaxis;
- Pregnancy, lactation or willingness/intention to become pregnant during the study;
- Current diagnosis or treatment for cancer (except basal cell carcinoma of the skin and cervical carcinoma in situ);
- History of severe psychiatric illness that possibly affects your participation in the study;

- Hemorrhagic disorder (for example, factor deficiency, coagulopathy or platelet disorder), or a previous history of significant bleeding or bruising after IM injections or venipuncture;
- Current suspected or known dependence on alcohol or drugs;
- Severe and/or uncontrolled cardiovascular diseases, respiratory diseases, gastrointestinal diseases, liver disease, kidney disease, endocrine disorder, and neurological disease (mild/moderate well-controlled comorbidities are allowed);
- History of COVID-19 confirmed by laboratory (serology, rapid tests based on antigen or antibody or PCR);
- Seropositive for antibodies to SARS-CoV-2 before recruitment; This exclusion criteria does not apply to participants enrolled from version 4.0 of the protocol onwards
- Continued use of anticoagulants, such as coumarins and related anticoagulants (for example, warfarin) or new oral anticoagulants (for example, apixaban, rivaroxaban, dabigatran and edoxaban);
- Any other significant illness, disorder or finding that may significantly increase the risk for the participant, affect his/her ability to participate in the study or impair the interpretation of the study data.

6.3.3 Re-vaccination exclusion criteria (two-dose groups only)

The following AEs associated with any vaccine, or identified on or before the day of vaccination constitute absolute contraindications to further administration of an IMP to the volunteer in question. If any of these events occur during the study, the subject will not be eligible to receive a booster dose and will be followed up by the clinical team or their regular doctor until resolution or stabilisation of the event:

- Anaphylactic reaction following administration of vaccine
- Pregnancy – if the outcome of pregnancy is termination or miscarriage, volunteers can be boosted if appropriate to do so and given they have a negative pregnancy test at the time of boosting.
- Any AE that in the opinion of the Investigator may affect the safety of the participant or the interpretation of the study results

- Participants who develop COVID-19 symptoms and have a positive PCR test after the first vaccination can only receive a booster dose after a minimum 4 weeks interval from their first PCR positive test, provided their symptoms have significantly improved. The decision to proceed with booster vaccinations in those cases will be at clinical discretion of the investigators. For participants who are asymptomatic and have a positive PCR test, a minimum of 2 weeks from first PCR positivity will be required before boosting

6.3.4 Effective birth control for volunteers

Participants of childbearing potential must use an effective form of birth control during the 12 months of study.

Acceptable forms of birth control for participants include:

- Established use of oral, injected or implanted hormonal of birth control methods;
- Placement of an intrauterine device (IUD) or intrauterine system (IUS);
- Complete hysterectomy;
- Bilateral occlusion of the tubes;
- Contraceptive barrier methods (condom or occlusive tampon with spermicide);
- Male sterilization, if the vasectomized partner is the participant's only sexual partner;
- True abstinence, when it is in line with the subject's preferred and usual lifestyle (periodic abstinence and withdrawal are not acceptable of birth control methods).

6.3.5 Withdrawal of participants

In accordance with the principles of the current version of the Declaration of Helsinki and any other applicable regulations, the participant has the right to withdraw from the study at any time and for any reason, and is not required to give his/her reasons for doing so. The Investigator may withdraw the participant at any time for the sake of his/her health and well-being. In addition, the participant can withdraw/be withdrawn for any of the following reasons:

- Investigator's Decision;
- Ineligibility (both during the study and retrospectively, having been omitted in the screening);
- Significant deviation from the protocol;
- Non-adherence of the participant to the study requirements;
- An AE, which requires discontinuing participation in the study or resulting in an inability to continue to comply with the study procedures.

The reason for the withdrawal will be registered with the CRF. If the withdrawal is a consequence of an AE, appropriate follow-up visits and/or medical care will be scheduled, with the consent of the participant, until the AE is resolved, stabilized or a causality unrelated to his/her participation in the study has been attributed. Any participant who is withdrawn from the study may be replaced, if this is possible within the specified period. The DSMB or DSMB president may recommend withdrawing participants.

If a participant withdraws from the study, the data collected before their withdrawal will still be used in the analysis. The storage of blood samples will continue unless the participant specifically requests otherwise.

In all cases of withdrawal from the subject, the collection of long-term safety data, including some procedures such as a safety blood test, will continue as appropriate if individuals have received one or more doses of the vaccine, unless they refuse any additional follow-up.

6.4 Pregnancy

If a participant becomes pregnant during the trial, she will be followed up for clinical safety assessment with her continuous consent and, in addition, she will be followed up until the outcome of the pregnancy is determined. We will not routinely perform venipuncture on a pregnant participant unless there is a clinical need. In addition, full and free follow-up and assistance will be ensured, for as long as necessary for: (a) the participants who become pregnant, and (b) the fetus, if applicable.

7 CLINICAL PROCEDURES

This section describes the clinical procedures for evaluating study participants and following up after administering the study vaccine.

7.1 Visit Schedule

All participants will have visits and clinical procedures as indicated in the visit schedule below table 4. The subjects will receive the ChAdOx1 nCoV-19 or MenACWY vaccine/saline solution placebo, and will be followed up for a total of 12 months post final vaccination procedure. Additional visits or procedures may be performed at the investigators' discretion, for example, medical history and additional physical examination, or additional blood tests, if they are clinically relevant.

All participants in groups 1a and 1b will be offered a booster dose of vaccine, however if a participant declines the booster dose they will continue in group 1a or 1b and follow the planned visit schedule, as per table 4.

7.2 Observations, medical history and physical examination

Temperature will be routinely measured at the time-points indicated in the schedule of procedures. Respiratory rate, oxygen saturation, pulse, blood pressure and temperature will be measured at the COVID-19 testing visits and if clinically required. All subjects will undergo medical history and a targeted physical examination if considered necessary at screening or pre-enrolment on D0. The purpose of this examination is to assess and document the subject’s baseline health status so that any later change can be determined. Vital signs (temperature, heart rate, respiratory rate, blood pressure +/- oxygen saturation), height and weight will be measured at screening or pre-enrolment on D0 as part of baseline assessments. Further medical history, physical examination and observations may be done throughout the study based on clinical discretion.

7.3 Blood samples, nose/throat swabs and urine analysis

- **PCR process for COVID-19.** A nose andthroat swab will be collected for COVID-19 PCR.

- **Immunology.** Immunogenicity will be assessed using a variety of immunological assays. This may include antibodies to SARS-CoV-Spike and non-Spike antigens by ELISA, ex vivo ELISpot assays for interferon gamma and flow cytometry assays, neutralization and other functional antibody assays and B cell analyses. Other exploratory immunological assays, including cytokine analysis and other antibody assays, among others, may be performed at the Investigators' discretion. Immunology samples for the assays described above will be drawn as per the schedule of attendances below (table 4)
- **Urine analysis.** In the case of participants of childbearing age, urine will be tested for beta-human chorionic gonadotropin (β-HCG) at screening (when applicable) and immediately before vaccination.

Collaboration with other specialized laboratories in the UK, Europe and outside of Europe may take place for new exploratory tests and for some of the immunology testing described above. This would involve transferring serum, or plasma, PBMC and/or other study samples to these laboratories, but these would remain anonymous. For this, after evaluation and prior approval of the REC/CONEP system, the participant will be presented with a new Informed Consent Form. Only after obtaining this new consent form can the samples be used for purposes other than those specified in this protocol.

Immunological tests will be performed according to the standard operating procedures of the research sites, the University of Oxford, and collaborating international laboratories.

The subjects will be informed which blood sample (after all tests for this study are completed), will be stored in a biorepository for future use. The subjects will be able to decide whether to allow such future use of any sample. With the informed consent of the participants, blood serum and/or PBMCs will be frozen for future analysis of COVID-19 and responses related to the vaccine. If a subject chooses not to allow this, no sample will be stored beyond the storage period required to meet Good Laboratory Practices (GLP) and regulatory requirements.

7.4 Study visits

Study visits and procedures will be performed by the research sites staff. The procedures to be included in each visit are documented in the visit schedule (table 4). Each visit is assigned a time and window period, within which the visit will take place.

7.4.1 Screening visit and recruitment

All potential participants will have a screening visit, up to 7 days before vaccination for a baseline assessment. For participants who are recruited in the study before version 4.0 of the study protocol, a serological evaluation of baseline antibodies against SARS-CoV-2 is performed. The results of the serology should be available within this period, no later than 7 days after collection. Volunteers with negative serology for IgG antibodies to SARS-CoV-2 may participate in the study (applicable to previous protocol versions only and not from version 4.0 onwards).

Having established that there is a low baseline seropositivity in the study population, the remaining participants can be included without baseline SARS-CoV-2 antibodies. This allows the screening visit to take place at the same day as the vaccination visit, and will precede vaccination procedure.

At the screening visit, the objectives of the study and all tests to be performed will be described to the participants. Individually, each participant will have the opportunity to question a duly trained and delegated researcher before signing the consent. The informed consent procedure will be performed before screening/recruitment procedures, as described in section 6.2. A medical history, including previous vaccinations, and targeted physical examination (when necessary) will be conducted at the screening visit. Findings will be recorded as part of baseline and eligibility assessment. A screening visit can be repeated if time from screening to vaccination is greater than the pre-specified window in the study protocol.

The research site staff may contact the subject's general practitioner/personal physician with written permission after screening to corroborate the clinical history when possible and practical to do so. The participant's doctor may be notified that the subject has been enrolled voluntarily in the study.

7.4.2 Day 0: Recruitment and vaccination visit

Participants will be considered enrolled in the trial at the time of vaccination. Before the investigational vaccination/treatment, the eligibility of the participant will be reviewed. The temperature and, if necessary, a medical history and physical examination will be performed to determine the need to postpone vaccination. The study vaccines/treatment will be administered as described below.

7.4.2.1 Vaccination

All vaccines will be administered intramuscularly according to specific standard operating procedures. The injection site will be covered with a sterile dressing and the participant will remain at the study site for observation, in case of immediate adverse events. Observations will be made at a minimum of 15 minutes after vaccination, the sterile dressing will be removed and the injection site inspected.

A sub sample of 200 participants will receive a thermometer, a metric ruler, access to the electronic diary via web, a printed diary (for use in case the electronic diary presents problems), guidelines and instructions for use, together with a contact card containing the number of 24-hour emergency telephone number to contact the research site if necessary. Participants will be instructed on how to self-assess the intensity and severity of requested adverse events (Table 3 - Requested and unsolicited AEs). There will also be space in the electronic (or paper) Symptom Diary for the participant to document unsolicited AEs for 28 days, and if a medication has been taken to relieve the symptoms. The diaries will collect information about the timing and severity of the following solicited AEs. Participants who were asked to fill out a diary post prime vaccination will be asked to fill out the same diary post booster vaccination.

Table 3. Spontaneous, requested AEs collected in the post-vaccination electronic diary (or daily card) for reactogenicity

AE spontaneous locations	AE systemic spontaneous
Pain	Fever
Sensitivity	Feeling feverish

Redness	Chills
Heat	Joint pain
Itching	Muscle ache
Swelling	Fatigue
Local hardening	Headache
	Malaise
	Nausea
	Vomiting

The use of electronic diaries allows the real-time monitoring of the safety of the study vaccines.

NB All volunteers who develop COVID-19 symptoms triggering the symptomatic pathway will be asked to fill out a symptoms diary (electronic or paper), which is separate to the vaccine reactogenity diaries described above.

7.4.3 Later visits

Follow-up visits will take place according to the visit schedule described in table 4, with their respective windows.

If the participants experience adverse events (laboratory or clinical), for which the investigator (doctor) and/or DSMB president requires more rigorous observation, the participant may be admitted to the hospital for observation and subsequent medical treatment under the care of the hospitalization team.

Table 4 Visiting schedule for participants

Groups 1a and 1b

Visit number	Screening	1	2	3	4	5	Test for COVID-19 (S0)	COVID-19 Testing +3-5 days (S3-5) - Only if PCR at S0 is negative	COVID-19 PCR positive + 7 days (S7)
Chronology** (days)	-7	0	28	90	182	364	As required	3-5 days post S0,	7 days after positive PCR for COVID-19
Time window (days)	±7		-7/+14	±7	±14	±30	N/A	±2	±2
Informed consent	X								
Review of contraindications, inclusion and exclusion criteria		X							
Vaccination		X							
Vital signs	X	X	X	X	X	X	X	(X)	X
Ascertainment of adverse events		X	X	X	X	X	X	X	X
COVID Hospital Admission Medical Records Review and Data Collection							If COVID-19 case results in Hospital Admission		

Electronic Diaries of Vaccine Symptoms $ (subset of volunteers only)		X	X					
Electronic Diaries of COVID-19 Symptoms (all participants in the study that enter the symptomatic pathway)							X		
Clinical history, physical examination		(X)	(X)	(X)	(X)	(X)	(X)	(X)	(X)
Biochemistry, hematology (mL)							5	(X) Only if clinically necessary	5
Exploratory immunology (mL)		Up to 50mL	Up to 50mL	Up to 50mL	Up to 50mL	Up to 50mL	10		10
Nose andThroat Swab							X	X [a]	
urinary bHCG (women only)	X	X							
Blood volume per visit		50	50	50	50	10	15		15
Accumulated blood volume %		50	100	150	200	250	265		280

(X) = if deemed necessary ^ = Vital signs at screening or pre-enrolment assessment on D0 include pulse, blood pressure, temperature, respiratory rate +/- oxygen saturation. Only temperature will be routinely measured at subsequent follow-up visits. At COVID-19 testing visits a full set of observations will be taken, including respiratory rate and oxygen saturation; ** Chronology is approximate only. The exact

times of the visits refer to the day of recruitment, that is, each visit must take place within the indicated interval of days after recruitment ± time window. % Accumulated blood volume for participants if blood is drawn according to schedule, and excluding any repeat safety blood tests that may be required. [a] A second PCR at 3-5 days post symptoms onset will be done if the first sample is negative. [$] Vaccine reactogeniticy diaries are applicable to a subset of participants only, all volunteers in the trial who present COVID-19 symptoms will be asked to fill out a COVID-19 symptoms diary.

NB Participants who refuse a booster vaccination should be followed-up as per the schedule of attendances above.

Group 1c and 1d

Attendance Number (boost)	Screening	1 (prime)	2 (booster)	3	4	5	6	COVID-19 Testing (S0)	COVID-19 Testing +3-5 days (S3-5) – Only if PCR at S0 is negative	COVD-19 Positive PCR + 7 days (S7)
Timeline** (days)	-7	0	4-12 weeks post prime	28 post booster	90 post booster	182 post booster	364 post booster	As required	3-5 days post S0	7 days post COVID-19 PCR positive

Attendance Number (boost)	Screenin g	1 (prime)	2 (booster)	3	4	5	6	COVID-19 Testing (S0)	COVID-19 Testing +3-5 days (S3-5) – Only if PCR at S0 is negative	COVD-19 Positive PCR + 7 days (S7)
Time window (days)	±7		+14	+7	±14	±14	±30	N/A	±2	±2
Informed Consent	X		X[b]							
Review contraindications, inclusion and exclusion criteria		X	X							
Vaccination		X	X							
Vital signs^	X	X	(X)	(X)	(X)	(X)	(X)	X	(X)	X
Ascertainment of adverse events		X	X	X	X	X	X	X	X	X
COVID Hospital Admission Medical Records Review and Data Collection								If COVID-19 case results in Hospital Admission		
Electronic Diaries of Vaccine Symptoms $		X	X							

Attendance Number (boost)	Screenin g	1 (prime)	2 (booster)	3	4	5	6	COVID-19 Testing (S0)	COVID-19 Testing +3-5 days (S3-5) – Only if PCR at S0 is negative	COVD-19 Positive PCR + 7 days (S7)
(subset of volunteers only)										
Electronic Diaries of COVID-19 Symptoms (all participants in the study that enter the symptomatic pathway)								X		
Medical History / Physical Examination		(X)	(X)	(X)	(X)	(X)	(X)	(X)	(X)	(X)
Biochemistry, Haematology (mL)								5	(X) Only if clinically necessary	5

Attendance Number (boost)	Screenin g	1 (prime)	2 (booster)	3	4	5	6	COVID-19 Testing (S0)	COVID-19 Testing +3-5 days (S3-5) – Only if PCR at S0 is negative	COVD-19 Positive PCR + 7 days (S7)
Exploratory immunology (mL)		Up to 50mL	Up to 50mL	up to 50	up to 50	up to 50	up to 50	up to 10		up to 10
Nose andThroat Swab								X	X [a]	
Urinary bHCG (women of childbearing potential only)	X	X	X							
Blood volume per visit		50	50	50	50	50	50	up to 15		up to 15
Cumulative blood volume (post boost)[%]		50	100	150	200	250	300	315		330

(X) = if deemed necessary ^ = Vital signs at screening or pre-enrolment assessment on D0 include pulse, blood pressure, temperature, respiratory rate +/- oxygen saturation. Only temperature will be routinely measured at subsequent follow-up visits. At COVID-19 testing visits a full set of observations will be taken, including respiratory rate and oxygen saturation; ** Chronology is approximate only. The exact times of the visits refer to the day of recruitment, that is, each visit must take place within the indicated interval of days after recruitment ±

time window. % Accumulated blood volume for participants if blood is drawn according to schedule, and excluding any repeat safety blood tests that may be required. [a] A second PCR at 3-5 days post symptoms onset will be done if the first sample is negative. [$] Vaccine reactogenicity diaries are applicable to a subset of participants only, all volunteers in the trial who present COVID-19 symptoms will be asked to fill out a COVID-19 symptoms diary. [b] New PIS/ICF only for participants enrolled before protocol 4.0 from groups 1c and 1d

NB Participants who accept to take part in booster dose subgroup will have their schedule of attendances replaced by above schedule. Participants who already attended their D28 visit post prime will be asked to attend a separate visit for their booster.

7.4.4 Symptomatic participants

Participants who become symptomatic during follow-up will be instructed to call the study team, who will then advise on how to proceed with clinical trials for COVID-19, if necessary, according to the trial work instructions. If a participant is symptomatic, COVID-19 testing should take place from enrolment onwards, regardless of time elapsed from vaccination to symptom onset. An isolated fever ≥37.8C is an indication for COVID-19 testing, unless this isolated fever has occurred within 48 hours of vaccination. If fever persists beyond 48 hours post-vaccination, the patient will then be eligible for COVID-19 testing. Participants will receive weekly reminders (for example, text messages - SMS or Whatsapp, surveillance APPs notifications, emailor telephone contacts) to contact the study team if they experience fever or cough or shortness of breath or anosmia/ageusia and if they are hospitalized for any reason. During the test visit for COVID-19, examination with nose/throat swab, collection of blood samples for safety will be performed (complete blood count, biochemistry, PCR and others, if considered clinically relevant) and immunology (serum and others), vital signs and other clinical data. Symptomatic participants can be regularly monitored by telephone, if appropriate. Participants who test positive for COVID-19 will continue to be followed throughout the duration of the trial, including repeated COVID-19 testing visits if symptomatic again during the course of the study and until the end of the trial. New episodes will be considered if they have a minimum 28 days interval between the previous PCR positive result.

If PCR is negative at S0 (first swab), participants will be asked to attend a follow-up visit at 3-5 days post symptoms onset (+2 days) for clinical review and further testing. Participants will be asked to record information on an electronic diary COVID-19 or a printed diary (for use in case the electronic diary presents problems), related symptoms for safety monitoring until symptom resolution or for at least 14 days if symptoms do not resolve before then. Volunteers who have 2 consecutive negative swabs may stop filling out the diaries before symptom resolution or 14 days. Participants who have a positive PCR at S0, will not be required to attend a S3-5 visit, but will be reviewed for safety at 7 days post positive swab. Clinical data, and additional blood samples for safety and immunology purposes will be taken at the S7 visit. Participants who have a positive swab at S3-5 will be reviewed for safety at 7 days post

positive swab where clinical data, and additional blood samples for safety and immunology purposes will be taken. Participants who have 2 negative PCR results from S0 and either a S3-5 visit will not be required to attend for an S7 visit. Closer follow-up and safety monitoring may be carried out by local trial teams if felt this is clinically indicated.

Participants who develop COVID-19 symptoms and have a positive PCR test after the first vaccination can only receive a booster dose after a minimum 4 weeks interval from their PCR positive test, provided their symptoms have significantly improved. The decision to proceed with booster vaccinations in those cases will be at clinical discretion of investigators. Booster vaccinations of participants are allowed to take place beyond the pre-specified 12 weeks window limit if they are required to be delayed due to PCR positivity within the preceding 4 weeks of the scheduled booster dose.

Further details and instructions on the symptomatic pathway can be found on the clinical study plan.

7.4.5 Review of the medical record

With the consent of the participant, the study team will request access to the medical records or send a data collection form to be filled out by the clinical team, in any episodes of medically attended COVID-19 . Investigators will aim to collect clinical data from medical records where participants with suspected COVID-19 have been admitted to Relevant data will be collected to verify the efficacy endpoints and disease enhancement. There is no internationally accepted definition of disease enhancement. Severity between groups will be described and compared. In addition, a proportion of serious illness/all illnesses will be constructed for recipients of the candidate and control vaccine. In case the vaccine induces increased disease, this proportion would be higher in the vaccine than in the control group. These probably include, but are not limited to information about ICU admissions, clinical parameters such as oxygen saturation, respiratory rates and vital signs, need for oxygen therapy, need for ventilatory support, blood test results and images, among others.

7.4.6 Randomization, blinding, and unblinding

Participants will be randomized to investigational vaccine or MenACWY vaccine in a 1:1 allocation ratio, using block randomization of 4 participants. The blinding scheme will be applied to the participants in relation to the arm in which they were allocated. The blinding scheme will not apply to the study team administering the vaccine. Vaccines will be prepared out of the participant's reach and the syringes will be covered with an opaque label that will guarantee the unilateral blinding of the participant.

If a participant's clinical condition requires unblinding, this will be done according to a specific study work instruction and the group allocation will be sent to the attending physician if unblinding is considered relevant and possibly changes treatment clinical.

7.5 Description of ChAdOx1 nCoV-19

ChAdOx1 nCoV-19 vaccine consists of the ChAdOx1 deficient replication monkey adenovirus vector, containing the SARS-CoV-2 structural surface glycoprotein antigen.

7.6 Storage

The vaccine manufactured by Advent is stored at -80 ^{o}C in a safe freezer at the clinics. The vaccine manufactured by Cobra Biologics Ltd is stored at 2-8°C in a secure fridge, at the clinical site. The traceability of the study vaccines will be documented according to the existing standard operating procedure (SOP). Accounting, storage, shipping, and handling of vaccines will be in accordance with relevant SOPs and forms.

7.7 Administration

On the day of vaccination, ChAdOx1 nCoV-19 will be thawed at room temperature and will be administered according to specific assay instructions. The vaccine manufactured by Cobra Biologics is a multi-dose vial which is stored at 2-8 degrees and does not require thawing. If the vaccine is stored outside of 2-8 it must be used within 6 hours. The vaccine will be administered intramuscularly in the deltoid of the non-dominant arm (preferably). All participants will be observed in the unit for a minimum of 15 minutes after vaccination. During the administration of research products, Advanced Life Support medications and

resuscitation equipment will be immediately available for the treatment of anaphylaxis. Vaccination will be performed and IMPs handled according to the relevant SOPs.

7.8 Rationale for selected dose

The dose to be administered in this study was selected based on clinical experience with the adenovirus vector ChAdOx1 expressing different inserts and other similar vectorized adenovirus vaccines (for example, ChAd63).

A first dose escalation study in humans using the ChAdOx1 vector encoding an influenza antigen (FLU004) administered ChAdOx1 NP + M1 safely at doses ranging from 5×10^{8} to 5×10^{10} pv. The subsequent review of the data identified an optimal dose of 2.5×10^{10} pv, balancing immunogenicity and reactogenicity. This dose was later administered to hundreds of volunteers in numerous larger phase 1 studies at the Jenner Institute. ChAdOx1 vectorized vaccines have so far shown to be very well tolerated. The vast majority of AEs have been mild to moderate and there have been no SARs to date.

Another monkey adenovirus vector (ChAd63) was safely administered in doses of up to 2×10^{11} pv, with an optimal dose of 5×10^{10} pv, balancing immunogenicity and reactogenicity.

MERS001 was the first clinical trial of a vector ChAdOx1 expressing the total Spike protein from a separate, but related beta-coronavirus. ChAdOx1 MERS has been administered to 31 participants so far in doses ranging from $5x10^{9}$ pv to $5x10^{10}$ pv. Despite the greater reactogenicity observed with the dose of $5x10^{10}$ pv, this dose was safe, with self-limiting AE and without registered SARs. The dose of $5x10^{10}$ pv was the most immunogenic, in terms of inducing neutralizing antibodies against MERS-CoV using a live virus assay (Folegatti et al. Lancet Infect Dis, 2020, https://doi.org /10.1016/S1473-3099(20)30160-2). Due to the immunology findings and the safety profile observed with a ChAdOx1 vector vaccine against MERS-CoV, the dose of $5x10^{10}$ pv was chosen for ChAdOx1 nCoV-19.

An analytical comparability assessment of ChAdOx1 nCoV-19 (AZD1222) manufactured by CBF, Advent and Cobra Biologics was conducted using a comprehensive set of physiochemical and biological release and characterization tests. In order to support the analytical comparability assessment, A260 testing of Advent's process (K.0007, K.0008, and K.0009 lots)

was performed, where corrections to the absorbance due to excess polysorbate 80 were made to compensate for polysorbate 80 concentrations above the formulation target of 0.1% (w/v).

Differences in strength related attributes (ie, virus particle concentration, virus genome concentration, and infectious virus concentration) are noted. These differences in strength is further examined for potential impact on clinical dosing. The target clinical dosage of CBF's product is 5×10^{10} viral particles per dose based on vp/mL concentration determined by UV spectroscopy (A260), whereas that of Advent's product is 5×10^{10} viral genome copies per dose based on vg/mL concentration determined by qPCR. The target clinical dosage of Symbiosis' product is $3.5 – 6.5 \times 10^{10}$ viral particles per dose based on the vp/mL concentration determined by A260, with a 0.5 mL dosing volume. This dosing range is based on a target 5×10^{10} viral particles per dose and a ± 30% range to take into account process and method variabilities. The planned clinical dosage of Symbiosis' product is compared to that of CBF and Advent products, the resulting Symbiosis' product dosage at 0.5 mL for lot 20481A is somewhat lower in total viral particle per dose (20% from the lower range limit), slightly higher in total viral genome copies per dose (12% from the higher range limit), and slightly lower in total infectious particle per dose (8% from the lower range limit). These differences are considered to be comparable to or within the variabilities from the analytical methods used in concentration determination (A260, qPCR, and infectivity) and the dosing volumes during clinical administration. In summary, with a 0.5 mL dosing volume for Symbiosis' product, strength difference from CBF and Advent products is not expected to have significant clinical impact in terms of reactogenicity and immunogenicity/efficacy

Table 12 Clinical Strengths of ChAdOx1 nCoV-19 (AZD1222) Drug Product

Strength Attribute	CBF		Advent			Cobra
	Lot 02P20-01	Lot 02P20-02	Lot K.0007	Lot K.0008	Lot K.0009	Lot 20481A
Concentration						
Virus particle concentration (A_{260}) (vp/mL)	1.49×10^{11}	1.22×10^{11}	3.12×10^{11}	3.16×10^{11}	2.45×10^{11}	0.8×10^{11}

Table 12 Clinical Strengths of ChAdOx1 nCoV-19 (AZD1222) Drug Product

Strength Attribute	CBF		Advent			Cobra
	Lot 02P20-01	Lot 02P20-02	Lot K.0007	Lot K.0008	Lot K.0009	Lot 20481A
Virus genome concentration (qPCR) (vg/mL)	1.7×10^{11}	Not tested	1.7×10^{11}	2.1×10^{11}	1.4×10^{11}	1.3×10^{11}
Infectious particle concentration (ifu/mL) [a]	2.6×10^{9}	Not tested	2.9×10^{9}	3.0×10^{9}	2.4×10^{9}	1.3×10^{9}
Target Clinical Dosage						
Equivalent DP volume per dose (mL)	0.34	0.41	0.294	0.235	0.356	0.50
Dosing of virus particle (vp/dose)	5.1×10^{10}	5.0×10^{10}	9.2×10^{10}	7.4×10^{10}	8.7×10^{10}	4.0×10^{10}
Dosing of viral genome (vg/dose)	5.8×10^{10}	NA	5.0×10^{10}	4.9×10^{10}	5.0×10^{10}	6.5×10^{10}
Dosing of infectious particle (ifu/dose)	8.8×10^{8}	NA	8.5×10^{8}	7.1×10^{8}	8.5×10^{8}	6.5×10^{8}

ifu = infectious units; NA = not applicable; vp = virus particle; vg = virus genome

[a] Testing performed using the Advent infectivity assay.

7.9 Environmental contamination control (GMO)

The possibility of environmental contamination with genetically modified organisms (GMOs) will be appropriately controlled. The study will be performed in accordance with the relevant local regulations regarding GMO products, following the recommendations of CTNBio. The approved SOPs will be followed to minimize the spread of the recombinant vector vaccine virus in the environment. GMO residues will be inactivated according to the approved SOPs. All material used during vaccination and by vaccination personnel will be autoclaved and incinerated later.

7.10 Control vaccine

Participants who are allocated to control groups will receive an injection of the MenACWY vaccine instead of ChAdOx1 nCoV-19. Either of the two MenACWY vaccines of authorized quadrivalent protein-polysaccharide conjugate – will be used, i.e.:

- Nimenrix (Pfizer). The authorized dosage of this vaccine for those over 6 months of age is a single intramuscular dose (0.5 mL), containing 5 mcg each of a polysaccharide from group A, C, W and Y of *Neisseria meningitidis*, each conjugated with 44 mcg of tetanus toxoid carrier protein.
- Menveo (Glaxo Smith Kline). The authorized dosage of this vaccine for those aged 2 years or older is a single intramuscular dose (0.5 mL), containing
  - 10 mcg of group A meningococcal polysaccharide, conjugated with 16.7 to 33.3 mcg of CRM $protein_{197}$ of *Corynebacterium diphtheriae*.
  - 5 mcg of group C meningococcal polysaccharide, conjugated with 7.1 to 12.5 mcg of CRM $protein_{197}$ of *Corynebacterium diphtheriae*.
  - 5 mcg of group W meningococcal polysaccharide, conjugated with 3.3 to 8.3 mcg of CRM $protein_{197}$ of *Corynebacterium diphtheriae*.
  - 5 mcg of group Y meningococcal polysaccharide, conjugated with 5.6 to 10.0 mcg of CRM $protein_{197}$ of *Corynebacterium diphtheriae.*

The summary of product characteristics for both vaccines allows the administration of a booster dose, if indicated by ongoing risk. Prior administration of a vaccine (or a quadrivalent simple meningococcal polysaccharide vaccine in groups A, C, W and Y) is not a contraindication to receiving another vaccine in this study.
The masking of the participants regarding the injection they are receiving will be maintained. A vaccination accounting record from MenACWY will be maintained at each study site.

MenACWY will be stored in a locked (or controlled access) refrigerator (2 °C to 8 °C) at the research site, according to the package insert.

7.11 Placebo

Participants who were allocated to the control group will receive a placebo injection of 0.9% saline instead of MenACWY at the time of boosting. The volume and site of injection will be the same as for the intervention arm and participants will be blinded as to which injection they are receiving. A vaccine accountability log of the saline will be maintained at each trial site, similar to what is done for the study intervention (ChAdOx1 nCoV-19) and the comparator used as the prime dose (MenACWY).

7.12 Compliance with the investigational treatment

All vaccines will be administered by the research team and registered with the CRF. The study medication will not be in the participant's possession at any time and, therefore, compliance will not be a problem.

7.13 Investigational treatment accounting

IMP accounting and control vaccines will be performed in accordance with the relevant SOPs.

7.14 Concomitant medication

As established by the exclusion criteria, participants cannot be enrolled in the study if they have received: any vaccine within 30 days prior to enrollment or if any other vaccine is expected to be administered within 30 days after each vaccination, any research product within 30 days prior to recruitment or if administration is planned during the study period, or if there is any chronic use (> 14 days) of any immunosuppressive medication in the 6 months prior to enrollment or if administration is planned at any time during the study period (topical steroids are allowed).

Participants who make continuous use of oral anticoagulants, such as coumarins and related anticoagulants (i.e. warfarin) or new oral anticoagulants (i.e. apixaban, rivaroxaban, dabigatran and edoxaban), and/or who received immunoglobulins and/or any blood products in the three months prior to the planned administration of the candidate vaccine, will be excluded from this study, according to the exclusion criteria.

Participants will be advised to take paracetamol, unless contraindicated (in which case they will be excluded from the study), for 24 hours after vaccination. Paracetamol will be stored according to the package insert. There will be no additional labeling beyond its authorized packaging.

7.15 Provision of treatment for controls

If the efficacy of the candidate vaccine is proven, after analysis of the primary endpoint and approval by the DSMB (Data and Safety Monitoring Committee), as established in the study protocol, the sponsor will make the candidate vaccine available to participants in the research allocated to the comparator group (MenACWY vaccine).

8 SAFETY ASSESSMENT

Safety will be assessed by the frequency, incidence and nature of the Aes and SAE emerging during the study.

8.1 Definitions

8.1.1 Adverse Event (AE)

An AE is any unexpected medical occurrence in a participant, which can occur during or after the administration of an IMP and does not necessarily have a causal relationship to treatment. An AE can therefore be any unfavorable and unintended sign (including any clinically significant abnormal finding or change from baseline), symptom or disease temporally associated with study treatment, even if it is considered to be related to study treatment or not.

8.1.2 Adverse Reaction (AR)

An AR is any unexpected or unintended response to an IMP. This means that the causal relationship between the IMP and the AE is at least reasonable, that is, the relationship cannot be ruled out. All cases judged by the medical investigator to have a reasonable causal relationship with an IMP (that is, possibly, probably, or definitely related to an IMP) will qualify as AR.

Adverse events that may be related to the IMP are listed in the Investigator's Brochure for each product.

8.1.3 Serious Adverse Event (SAE)

An SAE is an AE that results in any of the following endpoints, considered or unrelated to the study treatment.

- Death;
- Life-threatening event (i.e., the participant was, in the Investigator's view, at immediate risk of death from the event);
- Persistent disability or disability or significant disability (i.e., substantial disruption of the ability to perform normal life functions);
- Hospitalization or extension of existing hospitalization, regardless of length of stay, even if it is a precautionary measure for continuous observation;
    - Note: Hospitalization (including hospitalization or outpatient hospitalization for an elective procedure) for a pre-existing condition that has not unexpectedly worsened does not constitute a serious AE.
- An important clinical event (which cannot cause death, be life-threatening or require hospitalization) that may, based on appropriate clinical criteria, harm the participant and/or require medical or surgical treatment to avoid one of the outcomes listed above. Examples of such clinical events include an allergic reaction that requires intensive treatment in an emergency room or clinic, blood dyscrasias or seizures that do not result in hospitalization;
- Congenital anomaly or birth defect.

8.1.4 Serious Adverse Reaction (SAR)

A serious AE that, in the opinion of the investigator or sponsor, is believed to be possibly, probably, or definitely related to IMP or any other treatment of the study, based on the information provided.

8.1.5 Suspected Unexpected Serious Adverse Reaction (SUSAR)

A SAR, whose nature and severity are not consistent with the information on the drug in question set out in the IB.

8.2 Expectation

IMP-related SAE is not expected in this study. All SARs will therefore be reported as SUSAR.

8.3 Predicted/expected adverse reactions:

Predictable/expected AR after vaccination with ChAdOx1 nCoV-19 include pain at the injection site, tenderness, erythema, heat, swelling, induration, itching, myalgia, arthralgia, headache, fatigue, fever, feverish feeling, chills, malaise, and nausea.

8.4 Adverse Events of Special Interest

The potentiation of the disease after vaccination with ChAdOx1 nCoV-19 will be monitored. Serious COVID-19 disease will be defined by clinical criteria. Detailed clinical parameters will be collected from medical records and aligned with agreed definitions as they emerge. These probably include, but are not limited to oxygen saturation, need for oxygen therapy, respiratory rate, need for ventilatory support, results of blood tests and images, among other clinically relevant parameters. As there is no internationally accepted definition of ADE, differences in the severity of the disease between the groups will be described. If the proportion of serious illness is similar between the two groups, this would support the lack of effectiveness and not the improvement of the illness.

Acute respiratory failure, pneumonitis, acute cardiac injury, arrhythmia, septic shock syndrome and acute kidney injury related to COVID-19 disease will be monitored based on the review of medical records of hospitalized participants.

Eosinophilia as a marker of deviated Th2 responses will be monitored routinely in participants who attend their study visits and follow-up of COVID-19. Marked eosinophilia of ≥ 1.5 $x10^9$/L will be reported as SAE.

AESI relevant to vaccination in general will also be monitored, such as: generalized seizure, Guillain-Barre Syndrome (GBS), Acute Disseminated Encephalomyelitis (ADEM), Thrombocytopenia, Anaphylaxis, Vasculitides, in addition to the requested serious Aes.

8.5 Causality

For each AE, an assessment of the relationship between the event and the administration of the vaccine will be performed by the clinician delegated by the IC. An interpretation of the causal relationship of the treatment with the AE in question will be made, based on the type of event; the relationship of the event to the time of vaccine administration; and the known biology of vaccine therapy. Alternative causes of AE will be considered and investigated, such as the natural history of pre-existing medical conditions, concomitant therapy, other risk factors and the temporal relationship of the event to vaccination. The causality assessment will take place during planned safety reviews and in the final safety analysis, except for SAEs, which must be designated immediately by the investigator reporting the events.

0	Not related	No temporal relationship with the product under study ***and***<br>alternative etiology (clinical, environmental, or other treatments) ***and***<br>Does not follow known pattern of response to the product under study
1	Unlikely	Unlikely temporal relationship with the product under study ***and***<br>alternative etiology (clinical, environmental, or other treatments) ***and***<br>It does not follow the typical or plausible pattern of response to the product under study.
2	Possible	Reasonable temporal relationship with the product under study; ***or***<br>Event not produced immediately by clinical, environmental, or other treatments; ***or***<br>Response pattern similar to that seen with other vaccines
3	Probable	Reasonable temporal relationship with the product under study; ***and***<br>Event not produced immediately by clinical, environmental, or other treatments ***or***<br>Known response pattern seen with other vaccines

4	Definitive	Reasonable temporal relationship with the product under study; ***and***<br>Event not produced immediately by clinical, environmental, or other treatments; ***and***<br>Known response pattern seen with other vaccines

Table 5. Guidelines for assessing the relationship between vaccine administration and an AE.

8.6 Reporting procedures for all adverse events

All local and systemic AE that occur within 28 days after vaccination observed by the Investigator or reported by the participant, whether or not attributed to the study medication, will be recorded by the participants in the Diary of Symptoms and by the Investigators in the study CRF. All Aes that result in the withdrawal of a participant from the study will be followed up until a satisfactory resolution occurs, or until a causality unrelated to the study is assigned (if the participant agrees to do so). SAE and Adverse Events of Special Interest will be collected throughout the study period.

8.7 Evaluation of severity

The severity of adverse events will be assessed according to toxicity rating scales adapted from the FDA for healthy volunteers recruited in preventive vaccine clinical trials, listed in the specific study work instructions and tables 6-8 below.

Adverse Event	Grade	Intensity
Injection site pain	1	Pain that is easily tolerated
	2	Pain that interferes with daily activity
	3	Pain that impairs daily activity
	4	Hospitalization or A/E visit
Sensitivity	1	Mild discomfort to touch

	2	Discomfort with movement
	3	Significant discomfort at rest
	4	Hospitalization or A/E visit
Injection site erythema	1	2.5 – 5 cm
	2	5.1 – 10 cm
	3	>10 cm
	4	Exfoliating dermatitis or necrosis
Injection site induration/swelling	1	2.5 – 5 cm and does not interfere with activity
	2	5.1 – 10 cm or interferes with activity
	3	> 10 cm or impairs daily activity
	4	Necrosis

Table 6. Severity criteria for local adverse events

*erythema ≤2.5cm is an expected consequence of skin puncture and therefore will not be considered an adverse event

Vital Signs	Grade 1 (mild)	Grade 2 (moderate)	Grade 3 (serious)	Grade 4 Potentially fatal
Fever (oral)	38, 0°C- 38, 4°C	38.5°C – 38.9°C	39.0°C – 40°C	> 40°C
Tachycardia (bpm)*	101 – 115	116-130	>130	A/E visit or hospitalization for arrhythmia
Bradycardia (bpm)**	50 – 54	45 – 49	<45	A/E visit or hospitalization for arrhythmia
Systolic hypertension (mmHg)	141 -150	151 – 155	≥155	A/E visit or hospitalization for malignant hypertension
Diastolic hypertension (mmHg)	91 – 95	96 – 100	>1100	A/E visit or hospitalization for malignant hypertension
Systolic hypotension (mmHg)***	85 – 89	80 – 84	<80	A/E visit or hospitalization for hypotensive shock
Respiratory Rate – breaths per minute	17 – 20	21-25	>25	Intubation

Table 7. Severity rating criteria for physical observations (applies to adults only).

*Measured after ≥10 minutes at rest ** When the resting heart rate is between 60 to 100 beats per minute. Use the clinical criterion when characterizing bradycardia among some populations of healthy subjects, for example, conditioned athletes. *** Only if symptomatic (for example, dizziness/vertigo)

GRADE 0	None
GRADE 1	Mild: Transient or mild discomfort (<48 hours); There was no interference with routine activity; no medical treatment/therapy was needed
GRADE 2	Moderate: Mild to moderate limitation in routine activity – some assistance may be required; minimal medical treatment/therapy was required or none
GRADE 3	Serious: Marked limitation in routine activity, some assistance is usually required; medical treatment/therapy was required.
GRADE 4	Potentially fatal: requires assessment in A/E or hospitalization

Table 8. Severity classification criteria for local and systemic AE.

8.8 Serious AE reporting procedures

To comply with the rules in force on reporting SAE to regulatory authorities, the event will be accurately documented and the notification deadlines respected. Serious adverse events will be reported on the SAE forms to members of the study team as soon as the Investigators become aware of their occurrence. Copies of all reports will be forwarded for review by the Principal Investigator (as Sponsor's representative) within 24 hours after the Investigator becomes aware of the alleged SAE. The DSMB will be notified of SAE that are considered to be possibly, probably or definitely related to the study treatments; the DSMB president will be notified immediately (within 24 hours) as soon as the Sponsor become aware of the occurrence. Normally, SAE will not be reported immediately to the Ethics Committee, unless there is a clinically significant increase in the rate of occurrence, an unexpected endpoint, or a new event that may affect the safety of study participants, at the discretion of the Principal Investigator and/or DSMB. In addition to the expedited report above, the Investigator will include all SAE in the annual Development Safety Update (DSUR) report. In addition, all local reporting requirements apply.

Cases falling under the Hy's Law should be reported as SAEs. A Hy's Law Case is defined by FDA Guidance for Industry "Drug-Induced Liver Injury: Premarketing Clinical Evaluation" (2009). Any study participant with an increase in Aspartate Aminotransferase (AST) or Alanine

Aminotransferase (ALT) ≥ 3x Upper Limit of Normal (ULN) together with Total Bilirubin ≥2xULN, where no other reason can be found to explain the combination of these abnormal results, e.g., elevated serum alkaline phosphatase (ALP) indicating cholestasis, viral hepatitis A, B or C, another drug capable of causing the observed injury, amongst others.

8.9 Procedures for reporting SUSARs

All SUSARs will be communicated by the sponsor's delegate to the Competent Authority and REC and other parties, as applicable. For fatal and life-threatening SUSARs, this will be done within 7 calendar days after the Sponsor or delegate becomes aware of the reaction. Any additional relevant information will be reported within 8 calendar days of the initial report. All other SUSARs will be informed within 15 calendar days.

The principal investigators will be informed of all SUSARs for the relevant IMP for all studies with the same Sponsor, even if the event occurred or not in the present study.

8.10 Safety assessments

The safety profile will be continuously assessed by the Investigators. The CI and relevant investigators (according to the study delegation record) will also review safety and SAE issues as they arise.

The DSMB will assess the frequency of events, safety, and efficacy data every 4-8 weeks and/or as needed. The DSMB will make recommendations regarding the conduct, continuation or modification of the study.

The Sponsor may put the study on hold and pause recruitment if SUSARs reported in other international trials within the ChAdOx1 nCoV-19 programme are considered to pose a significant safety concern to all participants in the programme. The DSMB will review such events and will make a recommendation as to whether or not recruitment can continue. Study procedures other than vaccinations (e.g. safety follow-ups, immunogenicity assessments, and COVID-19 testing procedures) will continue as normal, regardless of length of study pause.

8.11 Data Safety Monitoring Committee

The Data Safety Monitoring Committee that is in force for the British studies COV001 and COV002 will also oversee this study and review the safety data for this study.

At least one properly qualified clinician/scientist from each international study site will be invited to attend meetings of the existing trial's DSMB.

The DSMB president can be contacted for independent advice and review by the Researcher or study sponsor in the following situations:

- Follow-up on any SAE considered possibly, probably or definitely related to a study treatment;
- Any other situation in which the Investigator or Study Sponsor feels that independent advice or review is important.

The DSMB will review the SAE considered to be possibly, probably, or definitely related to the study treatments. The DSMB will be notified within 24 hours after the Sponsor become aware of the occurrence. The DSMB can recommend stopping recruitment into the study, if necessary, to follow up on an SAE related to a study treatment. It will also recommend restarting the study, when appropriate, following review of such safety events (i.e. SUSARs associated with ChAdOx1 nCoV-19).

The DSMB will review safety data from volunteers aged 56 and above recruited as part of the COV002 UK study. Recruitment of older adults will only be allowed following advice from the DSMB.

9 STATISTICS

9.1 Description of statistical methods

Both a fully detailed study level statistical analysis plan (SAP) as well as a separate Statistical Analysis Plan for the Marketing Authorisation Application (MAA SAP) will be written and signed off before any interim data analyses are conducted.

The data from this study will be included in prospective pooled analyses of studies for efficacy and safety of ChAdOx1 nCoV-19 to provide greater precision of both efficacy and safety outcomes

9.2 Efficacy Outcomes

The primary efficacy endpoint is PCR positive symptomatic COVID-19.

This is defined as a participant with a PCR+ swab and at least one of the following symptoms: cough, fever > 37.8, shortness of breath, anosmia, or ageusia.

Where possible, sensitivity analyses will be conducted using common alternative definitions of virologically-confirmed COVID-19 disease, including those in use in other phase 3 protocols (including but not limited to: USA AstraZeneca phase 3 trial, South Africa COV005 trial, WHO solidarity trial, CEPI definition). This will aid in comparisons between various studies and meta-analyses. These alternative definitions will be detailed in the statistical analysis plan as exploratory analyses.

Due to the vaccine-induced disease mitigation potential, the inclusion of all positive PCR infections as a primary result may lower the vaccine's estimated effectiveness and reduce its accuracy. COVID-19 disease, positive for PCR and symptomatic, is a more specific primary outcome and may lead to an earlier demonstration of vaccine efficacy, although it includes fewer cases.

Regarding the differentiation of ADE and the lack of effectiveness of the vaccine: there is no internationally accepted definition of ADE. Differences in disease severity between groups will be described. If the proportion of serious illness is similar between the two groups, this would support the lack of effectiveness and not the improvement of the illness.

9.2.1 Efficacy

The primary and secondary analysis will be conducted on participants who are seronegative at baseline. A sensitivity analysis will be conducted including all participants regardless of baseline serostatus.

The screening of the participants will be based on the serological exam with IgG antibodies negative for SARs-CoV-2. However, for the analysis of the primary outcome, a validated assay detecting antibodies against SARS-CoV-2 nucleoprotein will be used to exclude any remaining participants who were seropositive at baseline.

Analysis of the primary endpoint will be computed as follows:

1. **Efficacy of two doses of ChAdOx1 nCoV-19**. Participants will be included who received two doses of ChAdOx1 nCoV-19. Events will be included if they occurred more than 14 days after the booster dose.
   Participants who are symptomatic up to 14 days after the second dose of vaccine will be excluded from the analysis. In addition, those with less than 14 days follow up post-second dose will also be excluded.

Secondary analyses of the primary outcome:

2. **Efficacy of at least one dose of vaccine**. Cases occurring more than 21 days after the first vaccination will be included.
   Participants who are symptomatic up to 21 days from vaccination will still attend site for PCR testing and blood samples but will be excluded from the analysis as these participants may have been exposed to SARS-CoV-2 prior to vaccination or before the immune system has had time to mount a response to the vaccine. In addition, those with less than 21 days follow up post-vaccination will also be excluded.

The proportions of participants meeting the primary outcome definition will be compared between groups of recipients of ChAdOx1 nCoV-19 and MenACWY using a Poisson regression model with robust variance (Zou 2004). The model will contain terms including treatment group, and age group at randomization if there is a sufficient sample size within each age category. The logarithm of the period at risk for primary endpoint will be used as an offset variable in the model to adjust for volunteers having different follow up times during which the events occur. Vaccine efficacy (VE) will be calculated as (1 – RR) x 100%, where RR is the relative risk of symptomatic infection (ChADOx1 nCoV-19: Control) and 95% confidence intervals will be presented.

If the Poisson regression model with robust variance fails to converge, the exact conditional method for stratified poisson regression will be used.

The cumulative incidence of symptomatic infections will be presented using the Kaplan-Meier method.

Secondary efficacy endpoints will be analysed in the same way as the primary efficacy endpoint.

Analyses will conducted for all adults combined as well as conducting analyses stratified by age cohorts.

All data from participants with PCR-positive swabs will be assessed for inclusion in the efficacy analyses by two blinded assessors who will independently review each case according to pre-specified criteria as detailed in the statistical analysis plan, to classify each for inclusion in the primary and secondary outcomes. A separate CRF will be designed for this purpose.

All PCR-positive results will be assessed for the primary outcome, including those with symptoms who were swabbed by trial staff and other potential sources of information such as health-care workers who are tested at their workplace as either a routine test procedure or due to developing symptoms.

9.2.2 Safety and Reactogenicity

For each group, the counts and percentages of each local and systemic adverse reaction requested from the daily cards, and all unsolicited AEs and SAEs will be presented..

9.2.3 Immunogenicity

Highly deviated antibody data will be transformed logarithmically before analysis. The geometric mean of the concentration and the associated 95% confidence interval will be summarized for each group at each timepoint, calculating the anti-log of the average difference of the logarithmically transformed data.

The geometric means of concentration on day 28 and the proportion of participants with serum conversion to S-spike protein from day 0 to day 28 will be computed. Comparisons between the ChAdOx1 nCoV-19 vaccine and control groups will be made using a Mann Whitney U test due to the low titers expected in the control group that will cause non-normal distribution.

9.3 Subgroup analyses

Subgroup comparisons of efficacy, and safety will be conducted by incorporating vaccine-group by subgroup interaction terms into appropriate regression models. Subgroup comparisons will only be conducted if there are at least 5 cases in all subgroups.

Comparisons will include:

1. Males vs females
2. Age (18 to 55 years vs 56-<70 years vs 70+ years)
3. Seropositive to S-spike or non-spike proteins at baseline vs not seropositive
4. Health care workers and highly-exposed participants versus others
5. Ethnicity
6. BMI (< 30 / >= 30 kg/m2)

9.4 Number of Participants

The research sites will include up to 10,300 participants (with a margin of 1%).

9.5 Interim and primary analyses of the primary outcome

It is planned that the primary evidence of efficacy and safety for the ChADOx1 nCoV-19 vaccine will be based on global analyses utilizing studies COV001 (the UK P1/2 study), COV002 (the UK P2/3 study), COV003 (the Brazil P3 study) and COV005 (the South Africa P1/2 study) including a pooled analysis across the studies. As such the interim and primary analyses for the primary outcome will be based on cases accumulated across multiple studies, details of which will be specified within the MAA SAP rather than for each individual study. Interim and primary data cuts from this study will therefore be carried out to support the pooled analysis.

The global MAA SAP allows for interim and primary analyses to be conducted once sufficient eligible cases have accumulated, where the overall type 1 error is controlled at the 5% level using a flexible alpha–spending approach that accounts for the incorporation of data from this study into pooled interim analyses under the global MAA SAP.

Evidence of efficacy will be determined if the lower bound of the multiplicity adjusted confidence interval is greater than a 20% threshold. The primary analysis will have approximately 90% power assuming a vaccine efficacy of 60%. A flexible alpha spending approach will be implemented to allow an earlier primary analysis in the situation where accumulation of eligible cases were lower than expected.

Evidence of efficacy at an interim or primary analysis of pooled data will not be considered a reason to stop the trial, but instead will be interpreted as early evidence of efficacy. However if an interim analysis demonstrates evidence of efficacy then a study level analysis according to the study SAP may be used to support study level evidence of efficacy.

9.6 Final Analysis

A final analysis will be conducted at the end of the study. The final study-specific analysis will incorporate all data from the study, including data that has previously contributed to global efficacy estimates under the pooled analysis strategy. The final analysis will be considered a supportive analysis to the global efficacy analysis.

9.7 Inclusion in the analysis

All vaccinated participants will be included in the analysis, unless otherwise specified

9.8 Data and Safety Monitoring Committee

The independent DSMB will meet regularly to review safety data from all available studies of ChAdOx1 nCoV-19. Additionally the independent DSMB will make recommendations based on the interim analyses to assess evidence of efficacy. The DSMB works according to the DSMB statute and/or follows the trigger points of the different protocols of the global clinical development plan, allowing the different steps to be achieved with respect to safety.

10 DATA MANAGEMENT

10.1 Data processing

The Principal Investigator will be responsible for all data that accumulates in the study.

All study data, including the participant's diary, will be recorded directly in an Electronic Data Capture (EDC) system (for example, OpenClinica, REDCap or similar) or in a paper source document for later insertion in EDC if the direct entry is not available. This includes safety data, laboratory data and endpoints data. All documents will be stored securely and in confidential conditions.

Participants will be identified by a unique number and code specific to the study in any database. The name and any other identifying details will NOT be included in any electronic study data file.

The EDC system (CRF data) uses a relational database (MySQL/PostgreSQL) through a secure web interface with data checks applied during data entry to ensure data quality. The database includes a full set of features that comply with GCP, EU and UK regulations and Sponsor security policies, including a full audit trail, user-based privileges and integration with the institutional LDAP server. The MySQL and PostgreSQL databases and the web server will be hosted on servers that are kept secure. The backups will be stored according to the IT department's schedule on a daily, weekly, and monthly basis and retained for one month, three months and six months, respectively. IT servers provide a stable, secure, well-maintained, high-capacity data storage environment. RedCap and OpenClinica are widely used, powerful, reliable and well supported systems. Access to the study database will be restricted to members of the study team with a username and password.

10.2 Record keeping

Investigators will maintain adequate medical and research records for this trial, in accordance with the GCP and regulatory and institutional requirements to protect the confidentiality of participants. The principal investigator, sub investigators and clinical research nurses will have access to the records. Investigators will allow Sponsor's authorized representatives, as well as ethical and regulatory agencies, to examine (and when required by applicable law, copy)

clinical records for the purposes of quality assurance reviews, audits and evaluation of the safety and progress of the study.

10.3 Source data and technical data sheets (CRFs)

All information required by the protocol will be collected in CRFs designed by the Sponsor. All source documents will be archived. The source documents are documents, data and original records from which the participant's CRF data is obtained.

For this study, these will include, but are not limited to, the Informed Consent Form, blood test results, and response letters from the general practitioner, laboratory records, diaries, medical records and correspondence. In this study, this will include, but is not limited to, medical history, medication records, vital signs, physical exam records, urine tests, blood test results, adverse event data and vaccine details. All source data and CRFs of the participants will be safely stored.

Where data are recorded directly onto the electronic data system these will be considered source documents. However, if local regulations require these electronic case report forms to be printed, they will be printed and filed in the participants

10.4 Data protection

The study protocol, documentation, data, and all other information generated will be kept strictly confidential. No information about the study or its data will be disclosed to unauthorized third parties without prior written approval from the sponsor.

Identifiable details, such as contact details, will be stored for a minimum of 5 years. Unidentified search data may be stored indefinitely. If participants agree to be contacted for future research, information about their Informed Consent Form will be recorded, retained and stored securely and separately from the research data.

10.5 Data quality

The data collection tools will undergo proper validation to ensure that the data is collected accurately and completely. The datasets provided for analysis will be subject to quality control processes to ensure that the data analyzed is a true reflection of the source data.

Study data will be managed in accordance with local data management SOPs. If an additional study of specific processes is necessary, an approved Data Management Plan will be implemented.

10.6 Archiving

Study data can be stored electronically on a secure server, and paper notes will be kept in a filing cabinet locked with a key in the site. All essential documents will be retained for a minimum of 5 years after the end of the study. The need to store study data for a longer time in relation to vaccine authorization will be subject to continuous review. For effective vaccines that can be authorized, we can safely store research data in the sites at least 15 years after the study ends, subject to adjustments to clinical trial regulations. Where participants' relevant bank details will be stored for 7 years, in accordance with the sites's financial policy. Unidentified search data may be stored indefinitely.

11 QUALITY CONTROL PROCEDURES AND QUALITY ASSURANCE

11.1 Investigator's Procedures

The approved standard operating procedures (SOPs) will be used in the research sites and in all laboratory centers.

11.2 Monitoring

Regular monitoring will be performed in accordance with the GCP by the monitor. When proceeding in accordance with the written SOPs, the monitor will verify that the clinical trial is performed and the data are generated, documented and reported in accordance with the protocol, GCP and applicable regulatory requirements. The sites will provide direct access to all data/documents and source reports related to the study for the purpose of monitoring and auditing by the Sponsor and to inspect by local and regulatory authorities.

11.3 Deviation from the protocol

Any deviations from the protocol will be documented on a protocol deviation form and filed in the study's master file.

Each deviation will be assessed for its impact on the safety of the participants and the conduct of the study. Significant deviations from the protocol will be listed at the end of the study report.

11.4 Audit and inspection

The QA manager conducts internal systems-based audits to verify that trials are being conducted in accordance with local procedures and in accordance with study protocols, departmental SOPs, GCP and applicable regulations.

The Sponsor, the study sites, the Research Ethics Committee, and the Regulatory Agencies may conduct audits to ensure compliance with the appropriate protocol, GCP, and regulations.

12 ETHICAL AND REGULATORY CONSIDERATIONS

12.1 Declaration of Helsinki

Investigators will ensure that this study is being conducted in accordance with the principles of the current revision of the Declaration of Helsinki.

12.2 Guidelines for good clinical practices

The Investigator will ensure that this trial is being conducted in accordance with the relevant standards and good clinical practices.

12.3 Ethical and regulatory approvals

After the Sponsor's approval, the Protocol, the Informed Consent Form, the participant's information sheet and any proposed advertising material will be submitted to an appropriate Research Ethics Committee (REC), regulatory authorities and host institutions for written approval. No changes to this protocol will be made without consulting the Sponsor and without its consent.

The Investigator is responsible for ensuring that changes in an approved study, during the period for which the approval of the Research Ethics Committee and Regulatory Agency has

already been given, are not initiated without their review and approval, except to eliminate immediate risks apparent to the subject (i.e., as an urgent safety measure).

12.4 Volunteers confidentiality

The study will comply with the EU General Data Protection Regulation (GDPR) and the UK Data Protection Act of 2018, as well as local data protection regulations, which require data not to be identified, whenever and when practical to do so. The processing of participants' personal data will be minimized by using only a single study number of the participant in all study documents and in any electronic database, with the exception of informed consent forms and participant identification records. All documents will be stored securely, and accessible only by study staff and authorized personnel. The study team will protect the privacy of participants' personal data. A separate confidential file containing personally identifiable information will be stored in a secure location in accordance with current data protection legislation. The photographs taken at the vaccination sites (if necessary, with the written and informed consent of the participant) will not include the face of the participant and will be identified by the date, study code and the subject's unique identifier. Once developed, the photographs will be stored as confidential records, as above. This material can be shown to other professionals, used for educational purposes, or included in a scientific publication.

If participants are diagnosed with COVID-19 during the course of the study, the study team will pass on their details to the local health protection team, if necessary, in accordance with the relevant notifiable disease legislation. Samples collected for the purpose of diagnosing COVID-19 can be sent to reference laboratories together with your personal data. This would be in line with national guidance and the policy of sending samples for testing in reference laboratories.

13 FUNDING AND INSURANCE

13.1 Funding

University of Oxford and external donors (Fundação Lemann, Fundacao Brava, Fundacao Telles, Instituto D'or de Ensino e Pesquisa and AstraZeneca Brasil).

13.2 Insurance

Global Insurance

Insured Party: University of Oxford

Exclusive market reference B 1526 CSHLC1900662

Research participants who suffer direct damage as a result of their participation in the study are entitled to claim compensation from the sponsor and the institutions involved in this study, covered by global and local insurance for research protocols.

13.3 Publication Policy

Researchers will be involved in reviewing draft manuscripts, abstracts, press releases and any other publications resulting from the study. The study data can also be used as part of a doctoral or master's thesis.

14 DEVELOPMENT OF A NEW PRODUCT/PROCESS OR INTELLECTUAL PROPERTY GENERATION

The IP title generated by University employees belongs to the University. The protection and exploitation of any new IP is managed by the University's technology transfer office, Oxford University Innovations. Researchers in this study can benefit from the University's royalty-sharing policy if new intellectual property is generated from the study. Several investigators are applicants or co-inventors of past patent registrations or patents related to ChAdOx1 vaccines. University of Oxford, which is a partner of Oxford University Hospitals NHS Foundation Trust at the NIHR Oxford Biomedical Research Centre, is committed to translational progress and the commercial development of healthcare products potentially

serving medical and global healthcare needs, and works and will work with business partners for these purposes.

References

1 Zhu, N. *et al.* A Novel Coronavirus from Patients with Pneumonia in China, 2019. *New England Journal of Medicine* **382**, 727-733, doi:10.1056/NEJMoa2001017 (2020).

2 Lu, R. *et al.* Genomic characterisation and epidemiology of 2019 novel coronavirus: implications for virus origins and receptor binding. *Lancet (London, England)* **395**, 565-574, doi:10.1016/S0140-6736(20)30251-8 (2020).

3 Li, F. Structure, Function, and Evolution of Coronavirus Spike Proteins. *Annual review of virology* **3**, 237-261, doi:10.1146/annurev-virology-110615-042301 (2016).

4 Zhou, P. *et al.* A pneumonia outbreak associated with a new coronavirus of probable bat origin. *Nature*, doi:10.1038/s41586-020-2012-7 (2020).

5 Alharbi, N. K. *et al.* ChAdOx1 and MVA based vaccine candidates against MERS-CoV elicit neutralising antibodies and cellular immune responses in mice. *Vaccine* **35**, 3780-3788, doi:10.1016/j.vaccine.2017.05.032 (2017).

6 Tseng, C. T. *et al.* Immunization with SARS coronavirus vaccines leads to pulmonary immunopathology on challenge with the SARS virus. *PloS one* **7**, e35421, doi:10.1371/journal.pone.0035421 (2012).

7 Weingartl, H. *et al.* Immunization with modified vaccinia virus Ankara-based recombinant vaccine against severe acute respiratory syndrome is associated with enhanced hepatitis in ferrets. *Journal of virology* **78**, 12672-12676, doi:10.1128/jvi.78.22.12672-12676.2004 (2004).

8 Liu, L. *et al.* Anti-spike IgG causes severe acute lung injury by skewing macrophage responses during acute SARS-CoV infection. *JCI insight* **4**, e123158, doi:10.1172/jci.insight.123158 (2019).

9 Agrawal, A. S. *et al.* Immunization with inactivated Middle East Respiratory Syndrome coronavirus vaccine leads to lung immunopathology on challenge with live virus. *Human vaccines & immunotherapeutics* **12**, 2351-2356, doi:10.1080/21645515.2016.1177688 (2016).

10 Munster, V. J. *et al.* Protective efficacy of a novel simian adenovirus vaccine against lethal MERS-CoV challenge in a transgenic human DPP4 mouse model. *NPJ vaccines* **2**, 28, doi:10.1038/s41541-017-0029-1 (2017).

11 Alharbi, N. K. *et al.* Humoral Immunogenicity and Efficacy of a Single Dose of ChAdOx1 MERS Vaccine Candidate in Dromedary Camels. *Scientific reports* **9**, 16292, doi:10.1038/s41598-019-52730-4 (2019).

12 Antrobus, R. D. *et al.* Clinical assessment of a novel recombinant simian adenovirus ChAdOx1 as a vectored vaccine expressing conserved Influenza A antigens. *Mol Ther* **22**, 668-674, doi:10.1038/mt.2013.284 (2014).

13 Coughlan, L. *et al.* Heterologous Two-Dose Vaccination with Simian Adenovirus and Poxvirus Vectors Elicits Long-Lasting Cellular Immunity to Influenza Virus A in Healthy Adults. *EbioMedicine*, doi:https://doi.org/10.1016/j.ebiom.2018.02.011 (2018).

14 Wilkie, M. *et al.* A phase I trial evaluating the safety and immunogenicity of a candidate tuberculosis vaccination regimen, ChAdOx1 85A prime – MVA85A boost in healthy UK adults. *Vaccine* **38**, 779-789, doi:10.1016/j.vaccine.2019.10.102 (2020).

15 Modjarrad, K. MERS-CoV vaccine candidates in development: The current landscape. *Vaccine* **34**, 2982-2987, doi:10.1016/j.vaccine.2016.03.104 (2016).

16 She, J., Liu, L. & Liu, W. COVID-19 epidemic: Disease characteristics in children. *J Med Virol*, doi:10.1002/jmv.25807 (2020).

17 Dong, Y. *et al.* Epidemiology of COVID-19 Among Children in China. *Pediatrics*, doi:10.1542/peds.2020-0702 (2020).

18 Ludvigsson, J. F. Systematic review of COVID-19 in children shows milder cases and a better prognosis than adults. *Acta Paediatr*, doi:10.1111/apa.15270 (2020).

19 Zimmermann, P. & Curtis, N. Coronavirus Infections in Children Including COVID-19: An Overview of the Epidemiology, Clinical Features, Diagnosis, Treatment and Prevention Options in Children. The Pediatric Infectious Disease Journal 39, 355-368, doi:10.1097/inf.0000000000002660 (2020).

APPENDIX A: AMENDMENTS HISTORY

Amendment No.	Version of the protocol No.	Date issued	Amendment author(s)	Details of Amendments made
N/A	1.0	May 27th, 2020	N/A	First version
1	1.0	May 31st, 2020	Sue Ann & Lily	Ethical requirements of the Brazilian Ethics Committee/CONEP system
2	2.0	June 10th, 2020	Pedro Folegatti	Ethical requirements of the English Ethics Committee (OxTREC) – updated data according to IB 6.0
3	3.0	June 14th, 2020	Sue Ann & Lily, Peter O'Reilly	ANVISA requests, updating of participating centers, electronic ICF, correction of sample size and exclusion of assistance to pregnant partners of research participants.
4	4.0	July 28th, 2020	Pedro Folegatti, Peter O'Reilly, Susan Tonks<br>Merryn Voysey	Added booster groups. Removed requirement for negative COVID-19 serology prior to enrollment; Added details on COBRA batch; clarified process around data entry/source data; updated statistical analysis section to reflect

				changes in trial procedures (e.g. addition of booster doses); added cellular immune responses in a subset of individuals as exploratory objective; added clarifications to swabbing procedures; added Hy's law cases as part of the requirement for SAE reporting; added clarifications to DSMB composition and their role in advising stopping recruitment.
5	4.1	August 11th, 2020	Pedro Folegatti	Clarify boosting dose windows; include placebo as comparator; update abbreviations; clarify the cellular immune response volunteers selection; clarify study groups; clarify non-hospitalized volunteers shall not take part in covid-19 treatment clinical trials; clarify serology criteria before/after protocol 4.0; update table 4
6	5.0	August 16th, 2020	Pedro Folegatti, Merryn Voysey	Increased sample size to up to 10,000; Changes to statistical analysis section, including changes to how the primary endpoint will be analysed; changes to the symptomatic pathway;

				clarifications to the inclusion and exclusion criteria
7	6.0	September 30th 2020	Pedro Folegatti, Sue Ann Clemens, Lily	Increased time from vial piercing to vaccine administration from 4 to 6 hours; clarifications to inclusion and exclusion criteria and removal of one of the exclusion criteria (participation in serological surveys); clarification and minor changes to symptomatic pathway in line with clinical study plan (requirements on visit S3-5, diaries, COVID-19 hospitalisation data collection); clarifications to vital signs collected at different timepoints; additional funders;
8	7.0	October 29th 2020	Pedro Folegatti, Sue Ann Clemens	Increase in sample size to up to 10.300 people to account for the competitive and simultaneous recruitment strategy at multiple sites.
9	8.0	12 Nov 2020	Pedro Folegatti, Sue Ann Clemens, Merryn Voysey	Clarifications to vital signs and physical measurements required at different visits; Updated Statistical Analysis section in line with global programme and with references to study level SAP and a separate SAP

				for Marketing Authorisation Application<br>Increased number of subset of individuals for CMI assessment from 50 to 60.

List details of all protocol amendments here whenever a new protocol version is produced.

Trial Title: An adaptive phase I/II randomized placebo-controlled trial to determine safety, immunogenicity and efficacy of non-replicating ChAdOx1 SARS-CoV-2 vaccine in South African adults living without HIV; and safety and immunogenicity in adults living with HIV.

Study Reference: ChAdOx1 nCoV-19_ZA_phI/II

Protocol Version: South Africa version 4.1

Date: ZA_30th September 2020

Trial registration: Clinicaltrials.gov: NCT04444674;
Pan African Clinical Trial Registry: PACTR202006922165132.

South African National Principal Investigator and Protocol Co-Chair:
Prof Shabir A. Madhi
Respiratory and Meningeal Pathogens Research Unit,
Wits Health Consortium (Pty) Ltd.

Protocol Co-Chair (UK): Prof Andrew Pollard
University of Oxford

Sponsor: University of Oxford

Funder: UK Research and Innovation (For Vaccine supply only)
Funding: The Bill and Melinda Gates Foundation
and South African Medical Research Council

**South African National Principal Investigator And Protocol Co-chair**	Professor Shabir A. Madhi<br>E-mail: madhis@rmpru.co.za<br>**Respiratory and Meningeal Pathogens Research Unit**<br>11th Floor, Nurses residence, Chris Hani Baragwanath Academic Hospital<br>Chris Hani Road, Soweto, Johannesburg, South Africa.<br>E-mail: madhis@rmpru.co.za
**South African clinical trial sites**	**Respiratory and Meningeal Pathogens Research Unit**<br>1st Floor, Nurses residence, Chris Hani Baragwanath Academic Hospital<br>Chris Hani Road, Soweto, Johannesburg, South Africa.<br>E-mail: koena@rmpru.co.za<br>**Site PI: Dr Anthonet Koen**

Setshaba Research Centre (SRC)
2088 Block H,
Soshanguve
0152, Gauteng, South Africa
Tel: +27 12 799 2422
Fax: +27 12 799 2685
Email: spadayachee@setshaba.org.za
Site PI: Dr Sherman Dominic Padayachee

Wits RHI Shandukani Research Centre
Premises 1: 2nd Floor, Hillbrow Health Precinct,
Corner Esselen Street and Klein Street, Hillbrow
Johannesburg, South Africa, 2001
Premises 2: 7 Esselen Street, Hillbrow,
Johannesburg, Gauteng, 2001
Phone: +27 11 358 5502
Fax: +27 86 548 4889
Email: LFairlie@wrhi.ac.za
Site PI: Dr Lee Fairlie

Perinatal HIV Research Unit, Kliptown.
Office no. 7, Walter Sisulu Square, Corner Union and Klipspruit Valley Roads, Kliptown, Soweto, 1809
Tel: +27 11 342 4075
Email: brinerc@phru.co.za
Site PI: Dr Carmen Briner, Anusha Nana

Main site:
Family Centre for Research with Ubuntu (FAMCRU)
FAMCRU
Ward J8, Tygerberg Hospital
Department of Paediatrics
Stellenbosch University
Francie van Zijl Ave
Parow Valley, 7505
Western Cape, South Africa
Tel: 021 938 4290 / 021 938 4157
Email: BARNABAS@sun.ac.za

Satellite sites:
Michael Mapongwana Community Health Centre (MMCHC)
Steve Biko Rd
Khayelitsha
Western Cape, South Africa

Kraaifontein Community Health Centre (MMCHC)
6th Avenue
Kraaifontein, 7570
Western Cape, South Africa

Worcester Community Health Centre (MMCHC)
1 Sugget Street
Worcester, 6850
Western Cape, South Africa
Site PI: Dr Shaun Barnabas

University of Cape Town Lung Institute and Centre for Lung Infection and Immunity (CLII)
H46.41 Old Main Building and E16 (Pulmonology Division)
Groote Schuur Hospital
Observatory
Tel: +21 406 6119
Email: keertan.dheda@uct.ac.za

Site PI: Prof Keertan Dheda

Soweto Clinical Trials Centre (SCTC)
House 1900, Sycamore Street,
Diamini Extension 2 Soweto, Johannesburg, 1818, RSA
Tel: +2711 984 9438
Direct Fax: +27 11 984 4417
E-mail: qeb@sowetoctc.co.za
Site PI: Dr Q. Bhorat

UK Protocol Co-Chair Prof Andrew Pollard

Address Centre for Clinical Vaccinology and Tropical Medicine
University of Oxford,
Churchill Hospital, Old Road, Headington
Oxford, OX3 7LE
Email: andrew.pollard@paediatrics.ox.ac.uk

South African Collaborators

Prof Lynn Morris
Interim Director
National Institute for Communicable Diseases,
Johannesburg, South Africa
E-mail: lynnm@nicd.ac.za

Prof. Penny Moore
Reader, University of the Witwatersrand
DST/NRF South African Research Chair of Virus-Host Dynamics Acting Head, Virology Section,
Centre for HIV and STIs,
National Institute for Communicable Diseases,
Johannesburg, South Africa
E-mail: pennym@nicd.ac.za

Dr Gaurav Kwatra
Scientist,
Respiratory and Meningeal Pathogens Research Unit
1st Floor, Nurses residence, Chris Hani Baragwanath Academic Hospital
Chris Hani Road, Soweto, Johannesburg, South Africa.
E-mail: kwatrag@rmpru.co.za

Dr Vicky Baillie
Scientist,
Respiratory and Meningeal Pathogens Research Unit
1st Floor, Nurses residence, Chris Hani Baragwanath Academic Hospital
Chris Hani Road, Soweto, Johannesburg, South Africa.
E-mail: bailliev@rmpru.co.za

Prof Marta Nunes
Scientist,
Respiratory and Meningeal Pathogens Research Unit
1st Floor, Nurses residence, Chris Hani Baragwanath Academic Hospital
Chris Hani Road, Soweto, Johannesburg, South Africa.
E-mail: nunesm@rmpru.co.za

Dr Clare Cutland
Scientific coordinator
African Leadership in Vaccinology Expertise, Alive
Room 10M11, Faculty of Health Sciences, University of the Witwatersrand,
York Road, Parktown, Johannesburg, South Africa
E-mail: cutlandc@rmpru.co.za

Dr Alane Izu
Statistician,
Respiratory and Meningeal Pathogens Research Unit
1st Floor, Nurses residence, Chris Hani Baragwanath Academic Hospital
Chris Hani Road, Soweto, Johannesburg, South Africa.
E-mail: izua@rmpru.co.za

UK Collaborators

Prof Brian Angus
Centre for Clinical Vaccinology and Tropical Medicine
Churchill Hospital, Old Road, Headington
Oxford, OX3 7LE
Email: brian.angus@ndm.ox.ac.uk

Prof Adrian Hill
The Jenner Institute, University of Oxford
Old Road Campus Research Building (ORCRB) Roosevelt Drive
Oxford OX3 7DQ
Email: adrian.hill@ndm.ox.ac.uk

Prof Sarah Gilbert
The Jenner Institute, University of Oxford
Old Road Campus Research Building (ORCRB) Roosevelt Drive
Oxford OX3 7DQ
Email: sarah.gilbert@ndm.ox.ac.uk

Dr Pedro Folegatti
Centre for Clinical Vaccinology and Tropical Medicine
Churchill Hospital, Old Road, Headington
Oxford, OX3 7LE
Email: pedro.folegatti@ndm.ox.ac.uk

Dr Alexander Douglas
The Jenner Institute
Wellcome Centre for Human Genetics
Roosevelt Drive
Oxford, OX3 7BN
Email: sandy.douglas@ndm.ox.ac.uk

Dr Maheshi Ramasamy
Oxford Vaccine Group
Centre for Clinical Vaccinology and Tropical Medicine
University of Oxford,
Churchill Hospital, Old Road, Headington
Oxford, OX3 7LE
Email : maheshi.ramasamy@paediatrics.ox.ac.uk

Prof Matthew Snape
Oxford Vaccine Group
Centre for Clinical Vaccinology and Tropical Medicine
University of Oxford,
Churchill Hospital, Old Road, Headington
Oxford, OX3 7LE
E-mail: matthew.snape@paediatrics.ox.ac.uk

**Funders**	UK Research and Innovation (Vaccine supply)<br><br>Clinical Trial: The Bill and Melinda Gates Foundation and South African Medical Research Council
**External Monitor, SA**	PPD SA (PTY) Ltd (blinded)<br><br>The Woodlands Office Park, Building 15,<br><br>Cnr Kelvin and Woodlands Drive, Woodmead, 2191, PO Box 37,<br><br>Woodlands, 2080<br><br>Phone: 011 612 8600<br><br>Fax: 011 612 8700<br><br>Contacts: Nicolette Stott; Nicolette.stott@ppdi.com<br><br>Savi Chetty-Tulsee (Unblinded, regulatory)<br>SCT consulting<br>Bryanston Homesteads, Ben Road, Bryanston, 2191<br>Email: savi.sct@mweb.co.za

Contents

Table of tables 12
Investigator agreement 13
1. SYNOPSIS 14
2. ABBREVIATIONS 29
3. BACKGROUND AND RATIONALE 31
3.1. Background 31
3.2. Pre-Clinical Studies 32
3.2.1. Immunogenicity (Jenner Institute, unpublished) 32
3.2.2. Efficacy 33
3.3. Antibody Dependent Enhancement 34
3.4. Previous clinical experience 34
3.5. Rationale 39
4. OBJECTIVES AND ENDPOINTS 40
5. TRIAL DESIGN 47
5.1. Trial participants 49
5.2. Definition of End of Trial 49
5.3. Duration of study 49
5.4. Potential Risks for participants 49
5.4.1. Venipuncture 49
5.4.2. Allergic reactions 49
5.4.3. Vaccination 49
5.4.4. Disease Enhancement 50
5.5. Known Potential Benefits 50
6. RECRUITMENT AND WITHDRAWAL OF TRIAL PARTICIPANTS 50
6.1. Identification of Trial Participants 50
6.2. Informed consent 51
6.3. Inclusion and exclusion criteria 52
6.3.1. Inclusion Criteria for all participants 52
6.3.2. Exclusion Criteria 53
6.3.3. Re-vaccination exclusion criteria 55
6.3.4. Effective contraception for female participants 55
6.3.5. Prevention of 'Over Participating' 56

6.3.6. Withdrawal of Participants 56
6.4. Pregnancy 57
7. TRIAL PROCEDURES 57
7.1. Schedule of Attendance 57
7.2. Observations 58
7.3. Blood tests, Nasal swab/saliva and urinalysis 58
7.4. Study visits 62
7.4.1. Screening visit 62
7.4.2. Day 0: Enrolment and vaccination visit 63
7.4.3. Vaccination 63
7.4.4. Subsequent visits: 64
7.4.5. Participants under quarantine 64
7.4.6. Symptomatic participants 71
7.4.7. Medical notes review 71
7.4.8. Randomisation, blinding and code-breaking 72
8. INVESTIGATIONAL PRODUCT 72
8.1. Manufacturing and presentation 72
8.1.1. Description of ChAdOx1 nCoV-19 72
8.2. Supply 72
8.3. Storage 73
8.4. Administration 74
8.5. Rationale for selected dose 74
8.6. Minimizing environmental contamination with genetically modified organisms (GMO) 77
8.7. Control Vaccine 78
8.8. Compliance with Trial Treatment 78
8.9. Accountability of the Trial Treatment 78
8.10. Concomitant Medication 78
8.11. Provision of Treatment for Controls 78
9. ASSESSMENT OF SAFETY 78
9.1. Definitions 78
9.1.1. Adverse Event (AE) 78
9.1.2. Adverse Reaction (AR) 79
9.1.3. Serious Adverse Event (SAE) 79
9.1.4. Serious Adverse Reaction (SAR) 80
9.1.5. Suspected Unexpected Serious Adverse Reaction (SUSAR) 80

9.2. Expectedness ... 80
9.3. Foreseeable Adverse Reactions: ... 80
9.4. Adverse Events of Special Interest ... 80
9.5. Causality ... 81
9.6. Reporting Procedures for All Adverse Events ... 82
9.7. Assessment of severity ... 82
9.8. Reporting Procedures for Serious AEs ... 85
9.9. Reporting Procedures for SUSARS ... 86
9.10. Development Safety Update Report ... 86
9.11. Procedures to be followed in the event of abnormal findings ... 86
9.12. Interim Reviews ... 87
9.13. Data Safety Monitoring Committee ... 87
9.14. Safety Group Holding Rules ... 88
9.15. Holding rules ... 88
9.15.1. Individual stopping rules ... 90
10. STATISTICS ... 92
10.1. Description of Statistical Methods ... 92
10.1.1 Efficacy endpoints ... 92
10.2. Primary efficacy [objective] and endpoint in COVID-19-naive persons ... 92
10.3. Secondary efficacy [objectives], endpoints and analyses, for overall population and based on COVID-19 sero-status at time of randomization ... 93
10.4. Exploratory efficacy endpoints ... 93
10.4.1. Safety & Reactogenicity ... 94
10.4.2. Immunogenicity ... 94
10.5. The Number of Participants ... 96
10.5.1. Sample size ... 96
10.6. Procedure for Accounting for Missing, Unused, and Spurious Data. ... 100
10.7. Inclusion in Analysis ... 100
10.8. Interim analysis ... 100
11. DATA MANAGEMENT ... 100
11.1. Data Handling ... 100
11.2. Record Keeping ... 101
11.3. Source Data and Case Report Forms (CRFs) ... 101
11.4. Data Protection ... 102
11.5. Data Quality ... 102

11.6. Archiving 102
12. QUALITY CONTROL AND QUALITY ASSURANCE PROCEDURES 102
12.1. Investigator procedures 102
12.2. Monitoring 102
12.3. Protocol deviation 103
12.4. Audit & inspection 103
13. ETHICS AND REGULATORY CONSIDERATIONS 103
13.1. Declaration of Helsinki 103
13.2. Guidelines for Good Clinical Practice 103
13.3. Ethical and Regulatory Approvals 103
13.4. Participant Confidentiality 104
14. FINANCING AND INSURANCE 104
14.1. Financing 104
14.2. Insurance 104
14.3. Compensation 105
15. Publication Policy 105
16. DEVELOPMENT OF A NEW PRODUCT/PROCESS OR THE GENERATION OF INTELLECTUAL PROPERTY 105
17. References 106
Appendix 1: Amendment history 107

Table of tables

Table 1: Clinical experience with ChAdOx1 viral vector vaccines. 35
Table 2: Clinical experience with ChAdOx1 MERS vaccine 38
Table 3: Details of objectives Groups 1 & 2 (HIV-uninfected): 41
Table 4: Symptoms of Suspected COVID-19 43
Table 5: Efficacy Endpoint Definitions of COVID-19 Severity 44
Table 6: Details of objectives Groups 3 (HIV-infected): 45
Table 7: Trial groups 48
Table 8: Schedule of visits: Groups 1 & 3 65
Table 9: Visit schedule for group 2a 67
Table 10: Visit schedule, group 2b (extended efficacy cohort; remaining 1650 participants) 69
Table 11: Guidelines for assessing the relationship of vaccine administration to an AE 81
Table 12: Severity grading criteria for local adverse events 82
Table 13: Severity grading criteria for select physical observations (Based on DAIDS Grading Table; Version 2.1 –July 2017 84
Table 15: Calculated probability of observing zero, at least one or at least two participants with and event among groups of size 25 or 50 for a range of true event probabilities: 96
Table 16: Exact Clopper-Pearson 95% confidence intervals (CI) when 0,1, or 2 participants observe a serious event for a group size of 25 or 50 97
Table 17: Minimum detectable difference in response rates between 2 groups calculated for various true response rates in the placebo group for groups size of 25 and statistical power of 80% and 90%. 97
Table 18: Sample size for group 2 required to conclude with 80% power the lower limit of a two-sided 95% confidence interval for vaccine efficacy (VE) is greater than 0% and 10%. 98
Table 19: Calculated power to conclude the lower limit of a 95% confidence interval for VE is greater than 0% or 10% for a total sample size of 1900 (950 per arm). 99

Investigator agreement

The principal investigator is responsible for ensuring that all study site personnel, including sub-investigators and other staff members, conduct this trial according to this protocol, Good Clinical Practice (GCP) and International Conference on Harmonization (ICF) guidelines, the Declaration of Helsinki and the pertinent country laws and regulations and to comply with its obligations, subject to ethical and safety considerations during and after the trial completion. The principal investigator also agrees not to disclose the information contained in this protocol or any results obtained from this trial without written authorization.

I have read and approve the protocol specified above and agree in its content:

Role: National Principal Investigator, Protocol co-chair	Name: Shabir A. Madhi
Signature:	Date:

Role: Protocol co-chair, University of Oxford	Name: Andrew Pollard
Signature:	Date:

Role: VIDA/ RMPRU site Principal Investigator	Name: Anthonet Koen
Signature:	Date:

Role: Wits RHI Shandukani Research Centre Principal Investigator	Name: Lee Fairlie
Signature:	Date:

Role: Setshaba Research Centre Principal Investigator	Name: Sherman D. Padayachee
Signature:	Date:

Role: PHRU Kliptown Principal Investigator	Name: Carmen Briner
Signature:	Date:

Role: FAMCRU Principal Investigator	Name: Shaun Barnabas
Signature:	Date:

Role: UCT CLII Principal Investigator	Name: Prof Keertan Dheda
Signature:	Date:

Role: SCTC Principal Investigator	Name: Dr Qasim Bhorat
Signature:	Date:

1. SYNOPSIS

**Trial Title**	An adaptive phase I/II randomized placebo-controlled trial to determine safety, immunogenicity and efficacy of non-replicating ChAdOx1 SARS-CoV-2 vaccine in South African adults living without HIV; and safety and immunogenicity in adults living with HIV
**Trial Identifier**	ChAdOx1 nCoV-19_ZA_phI/II
**Trial Registration**	Clinicaltrials.gov: NCT04444674;<br>Pan African Clinical Trial Registry: PACTR202006922165132
**National Principal investigator and Protocol Co-chair, South Africa**	Professor Shabir A. Madhi
**UK Protocol Co-chair**	Professor Andrew Pollard

**Trial Centers**	**Respiratory and Meningeal Pathogens Research Unit**<br>1st Floor, Nurses residence, Chris Hani Baragwanath Academic Hospital<br>Chris Hani Road, Soweto, Johannesburg, South Africa.<br>E-mail: koena@rmpru.co.za<br>Site PI: Dr Anthonet Koen<br><br>**Setshaba Research Centre (SRC)**<br>2088 Block H, Soshanguve, 0152, Gauteng, South Africa<br>Tel: +27 12 799 2422, Fax: +27 12 799 2685<br>Email: spadayachee@setshaba.org.za<br>Site PI: Dr Sherman Dominic Padayachee<br><br>**Wits RHI Shandukani Research Centre**<br>2nd Floor, Hillbrow Health Precinct, Corner Esselen Street and Klein Street, Hillbrow, Johannesburg, South Africa, 2001<br>Phone: +27 11 358 5502, Fax: +27 86 548 4889<br>Email: LFairlie@wrhi.ac.za<br>Site PI: Dr Lee Fairlie<br><br>**Perinatal HIV Research Unit, Kliptown**.<br>Office no. 7, Walter Sisulu Square, Corner Union and Klipspruit Valley Roads, Kliptown, Soweto, 1809<br>Tel: +27 11 342 4075<br>Email: brinerc@phru.co.za<br>**Site PI: Dr Carmen Briner**<br><br>**University of Cape Town Lung Institute and Centre for Lung Infection and Immunity (CLII)**<br>H46.41 Old Main Building and E16 (Pulmonology Division)<br>Groote Schuur Hospital<br>Observatory<br>Tel: +21 406 6119<br>Email: keertan.dheda@uct.ac.za<br><br>**Site PI: Prof Keertan Dheda**

	**<u>Main site:</u>**<br>**Family Centre for Research with Ubuntu (FAMCRU)**<br>FAMCRU<br>Ward J8, Tygerberg Hospital, Department of Paediatrics, Stellenbosch University<br>Francie van Zijl Ave, Parow Valley, 7505, Western Cape, South Africa<br>Tel: 021 938 4290 / 021 938 4157<br>Email: BARNABAS@sun.ac.za<br><br>**<u>Satellite sites:</u>**<br>Michael Mapongwana Community Health Centre (MMCHC)<br>Steve Biko Rd, Khayelitsha, Western Cape, South Africa<br><br>Kraaifontein Community Health Centre (MMCHC)<br>6th Avenue, Kraaifontein, 7570, Western Cape, South Africa<br><br>Worcester Community Health Centre (MMCHC)<br>1 Sugget Street, Worcester, 6850, Western Cape, South Africa<br>**Site PI: Dr Shaun Barnabas**<br><br>**Soweto Clinical Trials Centre (SCTC)**<br>House 1900, Sycamore Street,<br>Diamini Extension 2 Soweto, Johannesburg, 1818, RSA<br>Tel: +2711 984 9438<br>Direct Fax: +27 11 984 4417<br>E-mail: qeb@sowetoctc.co.za<br>**Site PI: Dr Q. Bhorat**
**Clinical Phase**	I/II
**Design**	Double -blinded, randomised, placebo controlled, multi-centre

Population Healthy adults aged 18-65 years, living with and without HIV

Planned Sample Size **2070** (possible upward adjustment for efficacy endpoint)

Planned Trial Duration: Regular visits from enrolment through to at least 12 months later.

Summary table of groups

Group #	Group description	Objective	Follow up	Treatment	Vaccination schedule
1 (n=70)	People without HIV (HIV-uninfected)	Intensive Safety and immunogenicity	Intensive	ChAdOx1 nCoV-19 5-7.5x1010 vp; OR Normal saline (0.9% NaCl)	2* doses, 4 weeks (21-35 days) apart
2a (n=250) §	People without HIV (HIV-uninfected)	Safety, intensive immunogenicity and vaccine efficacy	Extended	ChAdOx1 nCoV-19 5-7.5x10^{10} vp; OR Normal saline (0.9% NaCl)	2* doses, 4 weeks (21-35 days) apart
2b (n=1650) §	People without HIV (HIV-uninfected)	Safety, immunogenicity and vaccine efficacy	Extended	ChAdOx1 nCoV-19 5-7.5x10^{10} vp; OR Normal saline (0.9% NaCl)	2* doses, 4 weeks (21-35 days) apart
3 (n=100)	People living with HIV<br><br>(HIV-infected)	Intensive Safety and immunogenicity	Intensive	ChAdOx1 nCoV-19 5-7.5x10^{10} vp; OR Normal saline (0.9% NaCl)	Prime-boost<br><br>2* doses, 4 weeks (21-35 days) apart

*Participants will receive 2 doses of the same injection (EITHER IP or placebo) as assigned at randomization.

§Numbers will be increased to supplement for corresponding number of individuals randomized prior to implementation of Version 3.0 of the protocol that tested positive for SARS-CoV-2 on PCR at time of randomization.

# Following review of the initial safety/immunogenicity phase I trial conducted in the UK; COV0001 trial, and after review of the initial safety/ immunogenicity trial COV0001 by the DSMC; it was confirmed that Group 2 participants will receive 2 doses. SAHPRA and WHREC had been informed of this decision based on the earlier protocol requirements. Also, considering the unpredictability of the force of SARS-CoV-2 infection and the lower than anticipated attack rate for the primary-endpoint cases in the study being undertaken in the UK, the sample size for Group 2 (efficacy cohort) was expanded from the 550 included in protocol version 1.0, dated 24th April 2020. This will involve enrolling up to a total of 1900 people in Group-2, which will provide 80% power to detect at least a 60% vaccine efficacy (lower bound of 95%CI >0) with an attack rate of 3.5% in the placebo arm. Ongoing review of the number of COVID-19 endpoint cases accrued during the course of the study, may lend itself to enrolling smaller number of participants should the attack rate be higher than 3.5%. The sample size for Group-1 has been increased to 70 to accommodate for the higher than anticipated infection rate with SARS-CoV-2 (6 of initial 24 randomized subjects in Group-1). Similarly, in anticipation of approximately one-third of Group-3 participants possibly being already infected with SARS-CoV-2, the sample size will be increased to 100 to have approximately 30 sero-negative vaccinees and placebo recipients enrolled into the study.

Visit schedule, group 1 and 3

Visit number	Screening	V1	V2	V3	V4	V5	V6*	V7*	V8*	V9*	V10	V11	COVID-19
Day #	-14 to -1	0 (Vax1)	3	7	14	28 (Vax2)	31	35	42	56	182	364	Illness
	Screening	D0	V1+ 3 days ±1; (day 2-4)	V1 +7 days ±2 (day 5-9)	V1+ 14 days ±3 (day 11-17)	Visit 1 + 28 days ±7 (day 21-35)	V5+3 days ±1	V5+7 days ±2	V5+14 days ±3	V5 +28 (±7)	D182 (±14)	D364 (±14 days)	As required [$]
Eligibility	X	X											
Consenting	X[§]	X[¥]											
Inclusion/ exclusion	X	X				X							
Contraindications	X	X				X							
Vital signs [#]	X	X	X	X	X	X	X	X	X	X	X	X	X
Medical history	X												X
Physical examination	X (full)	X	X	X	X	X (full)	X	X	X	X	X	X	X (full)
Vaccination		X				X							
Post-vaccination obs		X	X (deltoid)	X (deltoid)		X	X (deltoid)	X (deltoid)					
Diary cards provided		X				X							X (illness DC)
DC collected				X				X					
Safety bloods (FBC, U&E, LFT)	X		X	X		X		X		X			
Screening bloods (HBsAg, HIV, HbA1C)	X											X (HIV Gr 1)	
HIV Viral load and CD4 (Grp 3 only)	VL and CD4												
Immunology bloods***		E, PAX (15.0-20.0ml)	Cyt, PAX (15.0 -20.0l)		E & CMI (20-25ml)	E, N, PAX (20.0-25.0ml)		Cyt (10-15mls)	E & N & CMI (25-30ml)	E (10-15ml)	E (10-15ml)	E & N (15-20ml)	E (10-20ml)
Urinalysis	X												
Urinalysis bHCG (women only)	X	(X)				X							
Nasal swab/ saliva	X (V1-96 hours)	X		X	X	X		X	X	X	X	X	X

• Visit 5 to Visit 9 are scheduled relative to when the 2nd dose of vaccine/placebo (Visit 4) has been administered.
§ Screening informed consent form (ICF).
¥Full study participation informed consent form, if remain eligible after completion of screening procedures.
Vital signs includes pulse, respiratory rate, oxygen saturation, blood pressure and temperature;
** Timeline is approximate only. Exact timings of visits relate to the day on enrolment, i.e., each visit must occur at indicated number of days after enrolment ± time window
***Abbreviations for laboratory tests: E =Elisa; Cyt= Th1 and Th2 cytokine profile; N= neutralization and/or pseudo-neutralisation assay; CMI= cell-mediated immunity assay, PAX= PAXgenes.
Blood test summary:

- Screening: Safety bloods (Full Blood Count, FBC; Urea and Electrolytes, U&E; Liver Function tests, LFT); Screening bloods (HBsAg, HIV, Glycosylated hemoglobin; HbA1c), In group 3 only- CD4+ -lymphocyte count, CD4+ & VHIV-1 viral load, VL)
- Visit 1: Immunogenicity- Elisa
- Visit 2 Safety bloods (FBC, U&E, LFT), Immunogenicity- Th1 and Th2 cytokine profile
- Visit 3 Safety bloods (FBC, U&E, LFT)
- Visit 4 Immunogenicity- Elisa & cell-mediated immunity
- Visit 5 Safety bloods (FBC, U&E, LFT), Immunogenicity- Elisa & neutralization and/or pseudo-neutralisation assay
- Visit 6 NIL
- Visit 7 Safety bloods (FBC, U&E, LFT), Immunogenicity- Th1 and Th2 cytokine profile
- Visit 8 Immunogenicity- Elisa, neutralization and/or pseudo-neutralisation assay & cell-mediated immunity
- Visit 9 Safety bloods (FBC, U&E, LFT), Immunogenicity- Elisa
- Visit 10 Immunogenicity- Elisa
- Visit 11 Immunogenicity- Elisa & neutralization and/or pseudo-neutralisation assays
- Illness visit Immunogenicity- Elisa

$ Nasal swabs/ saliva and Elisa (illness) will be repeated at Days 5-8, 12-15 and 28-35 days.

Visit schedule, group 2a (first 250 participants)

Visit number	Screening	V1	V2	V3	V4	V5*	V6*	V7*	V8	V9	COVID-19
Day #	-14 to -1	0 (Vax1)	7	14	28 (Vax2)	35	42	56	182	364	Illness
	Screening	D0	V1 +7 days ±2 (day 5-9)	V1+ 14 days ±3 (day 11-17)	Visit 1 + 28 days ±7	V4+7 days ±2	V4+14 days ±3	V4 +28 (±7)	D182 (±14)	D364 (±14 days)	As required $
Eligibility	X	X									
Consenting	X§	X¥									
Inclusion/ exclusion	X	X			X						
Contraindications	X	X			X						
Vital signs #	X	X	X	X	X	X	X	X	X	X	X
Medical history	X										X
Physical examination	X (full)	X	X	X	X (full)	X	X	X	X	X	X (full)
Vaccination		X			X						
Post-vaccination obs		X	X (deltoid)		X	X (deltoid)					
Diary cards provided		X			X						X (illness DC)
DC collected			X			X					
Screening bloods (HBsAg, , HIV, HbA1C)	X									X (HIV)	
Immunology bloods***		E, PAX (15.0-20.0)	Cyt, PAX (15.0 -20.0ml)	E & CMI (20-25ml)	E, N, PAX (20.0-25.0ml)		E & N & CMI (25-30ml)	E (10-15ml)	E (10-15ml)	E & N (15-20ml)	E (10-20ml)
Urinalysis	X										
Urinalysis bHCG (women only)	X	(X)			X						
Nasal swab/ saliva	X (V1-96 hours)	X	X	X	X	X	X	X	X	X	X

• Visit 5 to Visit 7 are scheduled relative to when the 2nd dose of vaccine/placebo (Visit 4) has been administered.

§ Screening informed consent form (ICF).

¥Full study participation informed consent form, if remain eligible after completion of screening procedures.

).

Vital signs includes pulse, respiratory rate, oxygen saturation, blood pressure and temperature;

** Timeline is approximate only. Exact timings of visits relate to the day on enrolment, i.e., each visit must occur at indicated number of days after enrolment ± time window
***Abbreviations for laboratory tests: E=Elisa; Cyt=Th1 and Th2 cytokine profile; N=neutralization and/or pseudo-neutralisation assay; CMI= cell-mediated immunity assay.

Blood test summary:

- Screening: Screening bloods (HBsAg, HIV, HBA1C)
- Visit 1: Immunogenicity- Elisa
- Visit 2 Immunogenicity- Th1 and Th2 cytokine profile
- Visit 3 Immunogenicity- Elisa & cell-mediated immunity assay
- Visit 4 Immunogenicity- Elisa & neutralization and/or pseudo-neutralisation assay, PAX- HLA
- Visit 5 Nil
- Visit 6 Immunogenicity- Elisa, neutralization and/or pseudo-neutralisation assay & cell-mediated immunity assay
- Visit 7 Immunogenicity- Elisa
- Visit 8 Immunogenicity- Elisa
- Visit 9 Immunogenicity- Elisa & neutralization and/or pseudo-neutralisation assay
- Illness visit Immunogenicity- Elisa

$ Nasal swabs/ saliva and Elisa (illness) will be repeated at Days 5-8, 12-15 and 28-35 days

Visit schedule, group 2b (extended efficacy cohort; remaining 1650 participants)

Visit number	Screening	V1	V2	V3	V4	V5	V6	COVID-19
Day #	-14 to -1	0 (Vax1)	28 (Vax2)	42	56	182	364	Illness
	Screening	D0	Visit 1 + 28 days ±7	V2+14 days ±3	V2 +28 (±7)	D182 (±14)	D364 (±14 days)	As required $
Eligibility	X	X						
Consenting	X§	X¥						
Inclusion/ exclusion	X	X	X					
Contraindications	X	X	X					
Vital signs #	X	X	X	X	X	X	X	X
Medical history	X							X
Physical examination	X(full)	X	X (full)	X	X	X	X	X (full)
Vaccination		X	X					
Post vaccination Obs		X	X					
Diary cards provided		X	X					X (illness DC)
DC collected			X					
Screening bloods (HBsAg, HIV, HbA1C)	X						X (HIV)	
Immunology bloods***		E (15-20 ml)	E, N, HLA (20-25 ml)	E & N (15-20ml)	E (10-15ml)	E (10-15ml)	E & N (15-20ml)	E (10-20ml)
Urinalysis	X							
Urinalysis bHCG (women only)	X	(X)	X					
Nasal swab/ saliva	X (V1-96 hours)	X	X	X	X	X	X	X

§ Screening informed consent form (ICF).
¥Full study participation informed consent form, if remain eligible after completion of screening procedures.
*
Vital signs includes pulse, respiratory rate, oxygen saturation, blood pressure and temperature;

** Timeline is approximate only. Exact timings of visits relate to the day on enrolment, i.e., each visit must occur at indicated number of days after enrolment ± time window

***Abbreviations for laboratory tests: E=Elisa; Cyt=Th1 and Th2 cytokine profile; N=neutralization and/or pseudo-neutralisation assay; CMI=cell-mediated immunity assay.

Blood test summary:

- Screening: Screening bloods (HBsAg, HIV, HbA1C)
- Visit 1: Immunogenicity- Elisa
- Visit 2 Immunogenicity- Elisa & neutralization and/or pseudo-neutralisation assay, HLA
- Visit 3 Immunogenicity- Elisa, neutralization and/or pseudo-neutralisation assay
- Visit 4 Immunogenicity- Elisa,
- Visit 5 Immunogenicity- Elisa
- Visit 6 Immunogenicity- Elisa & neutralization and/or pseudo-neutralisation assay
- Illness visit Immunogenicity- Elisa

$ Nasal swabs/ saliva and Elisa (illness) will be repeated at Days 5-8, 12-15 and 28-35 days.

Objectives:

In adults without HIV (HIV-uninfected)

Primary objective:

To assess the safety of the candidate vaccine ChAdOx1 nCoV-19 in healthy HIV-uninfected adults.

Co-primary objective:

To assess efficacy of the candidate ChAdOx1 nCoV-19 against COVID-19, defined as virologically confirmed (PCR positive) COVID-19 disease, in participants that were COVID-19 naïve at time of randomization and who received two doses of ChAdOx1 nCoV-19 or placebo. Events will be included if they occurred more than 14 days after the booster dose. "COVID-19 naïve" will be defined as sero-negative and tested negative for SARS-CoV-2 infection based on a high sensitivity serology antibody test and molecular detection testing of nasal swab, respectively

Secondary objective

To assess the immunogenicity of ChAdOx1 nCoV-19 in healthy HIV-uninfected adults

Details of objectives Groups 1 & 2 (HIV-uninfected):

**Objective**	**Objective details**	**Endpoint measures**
Primary Objective (Group 1 and Group 2)	To assess the safety, tolerability and reactogenicity profile of the candidate vaccine ChAdOx1 nCoV-19	a) occurrence of solicited local reactogenicity signs and symptoms for 7 days following vaccination;<br>b) occurrence of solicited systemic reactogenicity signs and symptoms for 7 days following vaccination;<br>c) occurrence of unsolicited adverse events (AEs) for 28 days following vaccination;<br>d) change from baseline for safety laboratory measures and;<br>e) occurrence of serious adverse events<br>e) occurrence of disease enhancement episodes

<table>
<tr>
<td>Co- Primary objective

(Group 2a and 2b; efficacy cohort)</td>
<td>To assess efficacy of the candidate ChAdOx1 nCoV-19 against mild to severe COVID-19</td>
<td>The primary efficacy [objective] and endpoint include PCR positive COVID-19 disease cases occurring in participants that were COVID-19 naïve at the time of randomization and who received at least two doses of ChAdOx1 nCoV-19 or placebo. Events will be included if they occurred more than 14 days after the booster dose. Virologically-confirmed COVID-19 clinical disease will be defined as an acute respiratory illness that is clinically consistent with COVID-19 based on presence of:
<table>
<tr><th>New onset systemic</th><th>Endpoint Definitions</th></tr>
<tr><td>Mild</td><td>Any one of:
• Fever (defined by subjective or objective measure, regardless of use of anti-pyretic medications)
• New onset cough
• ≥ 2 COVID-19 respiratory/non-respiratory symptoms, AND</td></tr>
<tr><td>Moderate</td><td>≥ 1 of:
• Fever (≥ 37.8°C) + any 2 COVID-19 symptoms for ≥ 3 days (need not be contiguous days)
• High fever (≥ 38.4°C) for ≥ 3 days (need not be contiguous days)
• Any evidence of significant LRTI:
Shortness of breath (or breathlessness or difficulty breathing) with or without exertion (beyond baseline)
Tachypnea: 20 to 29 breaths per minute at rest
SpO_2: < 94% on room air
Abnormal chest x-ray/CT consistent with pneumonia or LRTI</td></tr>
<tr><td>Severe</td><td>≥ 1 of:
• Tachypnea: ≥ 30 breaths per minute at rest
• SpO_2: < 92% on room air or PAO_2/FiO_2 < 300
• High flow oxygen therapy, CPAP, or NIV (eg, CPAP/BiPAP)
• Mechanical ventilation or ECMO
• One or more major organ system failure[a] (eg, cardiac/circulatory, pulmonary, renal, hepatic to be</td></tr>
</table>
</td>
</tr>
</table>

Secondary objectives (Group 2)	To assess the efficacy of the candidate ChAdOx1 nCoV-19 against COVID-19 of differing severity	All secondary VE analyses will be done for the overall population and stratified by COVID-19 serological status at baseline.<br>a. VE in preventing virologically-confirmed COVID-19 clinical disease including all cases occurring onward from 21 days after a single dose.<br>b. VE in preventing virologically-confirmed COVID-19 clinical disease occurring more than 14 days after a second dose for the overall population and those that were sero-positive at baseline..<br>c. VE in preventing PCR positive COVID-19 disease cases.<br>d. VE in preventing moderate-severe virologically confirmed COVID-19 disease.<br>e. VE in preventing severe confirmed COVID-19 disease.<br>f. VE in preventing LRTI associated with virologically-confirmed COVID-19 clinical disease<br>g. VE in preventing hospitalization due to virologically confirmed COVID-19 disease<br>h. VE in preventing all-cause LRTI (overall and stratified by hospitalization or not) irrespective of test result for SARS-COV-2.<br>i. h. VE using Oxford Primary Outcome definition (PCR+ at least one symptom of fever > 37.8°C, cough, shortness of breath, anosmia, aguesia).
Secondary objective (Group 1 and Group 2)	To assess cellular and humoral immunogenicity of ChAdOx1 nCoV-19	a) Enzyme-linked immunosorbent assay (ELISA) or fluorescence based micro-bead immunosorbent assay on luminex platform to quantify antibodies against SARS-CoV-2 spike protein (sero-conversion rates)<br>b) Interferon-gamma (IFN-γ) enzyme- linked immunospot (ELISpot) responses to SARS-CoV-2 spike protein<br>c) Virus neutralising antibody (NAb) assays against live and/or pseudotyped SARS-CoV-2 virus<br>d) Th1 and Th2 cytokine response profile at 3-4 days after vaccination.
Exploratory immunology:	To assess B cell responses to SARS-CoV-2 spike trimer and/or the receptor binding domain	a.Cellular Fc effector functionality assays to measure the ability of vaccine elicited antibodies to mediate cellular cytotoxicity, complement deposition, and phagocytosis.<br>b. Flow cytometric sorting of plasmablasts and memory B cells to using spike and receptor binding domain "baits" to isolate SARS-CoV-2 specific B cells, sequence their immunoglobulin genes and define their epitope specificity.

In adults living with HIV (HIV-infected)

Primary co-objectives:

- To assess the safety of the candidate vaccine ChAdOx1 nCoV in adults living with HIV.
- To evaluate the immunogenicity of ChAdOx1 nCoV-19 after first and second doses of vaccine.

Details of objectives Group 3 (HIV-infected):

	**Objective details**	**Endpoint measures**
Primary objective	To assess the safety, tolerability and reactogenicity profile of the candidate vaccine ChAdOx1 nCoV-19 in people living with HIV	a) occurrence of solicited local reactogenicity signs and symptoms for 7 days following vaccination;<br>b) occurrence of solicited systemic reactogenicity signs and symptoms for 7 days following vaccination;<br>c) occurrence of unsolicited adverse events (AEs) for 28 days following vaccination;<br>d) change from baseline for safety laboratory measures and;<br>e) occurrence of serious adverse events<br>e) occurrence of disease enhancement episodes
Co-primary objective	To assess cellular and humoral immunogenicity of ChAdOx1 nCoV-19 in people living with HIV after one and two doses of vaccine	a) Enzyme-linked immunosorbent assay (ELISA) or fluorescence based micro-bead immunosorbent assay on luminex platform to quantify antibodies against SARS-CoV-2 spike protein (seroconversion rates)<br>b) Interferon-gamma (IFN-γ) enzyme-linked immunospot (ELISpot) responses to SARS-CoV-2 spike protein<br>c) Virus neutralising antibody (NAb) assays against live and/or pseudotyped SARS-CoV-2 virus<br>d) Th1 and Th2 cytokine response profile at 3-4 days after vaccination.

Secondary objective	To descriptively compare immune responses to ChAdOx1 nCoV-19 in people living with HIV to HIV-uninfected individuals, overall and stratified by COVID-19 sero-status at enrolment.	a) Enzyme-linked immunosorbent assay (ELISA) or fluorescence based micro-bead immunosorbent assay on luminex platform to quantify antibodies against SARS-CoV-2 spike protein (seroconversion rates)<br>b) Interferon-gamma (IFN-γ) enzyme-linked immunospot (ELISpot) responses to SARS-CoV-2 spike protein<br>c) Virus neutralising antibody (NAb) assays against live and/or pseudotyped SARS-CoV-2 virus<br>d) Th1 and Th2 cytokine response profile at 3-4 days after vaccination.
Exploratory immunology	To assess B cell responses to SARS-CoV-2 spike trimer and/or the receptor binding domain	a. Cellular Fc effector functionality assays to measure the ability of vaccine elicited antibodies to mediate cellular cytotoxicity, complement deposition, and phagocytosis.<br><br>b. Flow cytometric sorting of plasmablasts and memory B cells to using spike and receptor binding domain "baits" to isolate SARS-CoV-2 specific B cells, sequence their immunoglobulin genes and define their epitope specificity.

Formulation Liquid

Investigational products

- ChAdOx1 nCoV-19, a non-replicating simian adenoviral vector expressing the spike (S) protein of SARS-CoV-2 (investigational product, IP)
- Normal saline, NaCl 0.9% as placebo

Route of Administration Intramuscularly (IM) into the deltoid region of the non-dominant arm

Dose per Administration ChAdOx1 nCoV-19 5-7.5x10^{10} vp

2. ABBREVIATIONS

**AdHu**	Human adenovirus
**AdHu5**	Human adenovirus serotype 5
**AE**	Adverse event
**AID**	Autoimmune Disease
**CCVTM**	Centre for Clinical Vaccinology and Tropical Medicine, Oxford
**CBF**	Clinical Bio manufacturing Facility
**CEF**	Chick embryo fibroblast
**ChAd63**	Chimpanzee adenovirus 63
**CI**	Confidence interval
**COP**	Code of Practice
**CRF**	Case Report Form or Clinical Research Facility
**CS or CSP**	Circumsporozoite protein
**CTRG**	Clinical Trials & Research Governance Office, Oxford University
**CTL**	Cytotoxic T Lymphocyte
**DSUR**	Development Safety Update Report
**ELISPOT**	Enzyme-linked immunospot
**GCP**	Good Clinical Practice
**GMO**	Genetically modified organism
**GMT**	Geometric Mean Titre
**GP**	General Practitioner
**GSK**	GlaxoSmithKline
**HCG**	Human Chorionic Gonadotrophin
**HBV**	Hepatitis B virus
**HEK**	Human embryonic kidney
**HCV**	Hepatitis C virus
**HIV**	Human immunodeficiency virus
**HLA**	Human leukocyte antigen
**HRA**	Health Research Authority
**HREC**	Human Research Ethics Committee
**HTLV**	Human T-Lymphotrophic Virus
**IB**	Investigator Brochure
**ICH**	*International Conference on Harmonisation*
ICMJE	*International Committee of Medical Journal Editors*
ICS	*Intracellular Cytokine Staining*
**IDT**	Impfstoffwerk Dessau-Tornau Biologika GmbH
**ID**	Intradermal
**IFNγ**	Interferon gamma
**IM**	Intramuscular
**IMP**	Investigational Medicinal Product
**IMP-D**	Investigational Medicinal Product Dossier
**IP**	Investigational Product
**IV**	Intravenous
**LSOC**	Local safety oversight clinician
**ME-TRAP**	Multiple epitopes and thrombospondin related adhesion protein
**MVA**	Modified vaccinia virus Ankara

**NANP**	N-acetylneuraminic acid phosphatase
**NHLS**	National Health Laboratory Service
**NICD**	National Institute for Communicable Diseases
**PBMC**	*Peripheral blood mononuclear cell*
Pb	*Plasmodium Berghei*
**PCR**	Polymerase chain reaction
**PI**	Principal Investigator
**pfu**	Plaque forming unit
**QP**	Qualified Person
**qPCR**	Quantitative polymerase chain reaction
**QS21**	*Quillaja saponaria* saponin molecule
**REC**	Research Ethics Committee
**SAE**	Serious adverse event
**SAHPRA**	South African Health Products Regulatory Authority
**SC**	Subcutaneous
**SmPc**	Summary of Product characteristics
**SOP**	Standard Operating Procedure
**SUSAR**	Suspected unexpected serious adverse reaction
**µg**	microgram
**vp**	viral particle
**VV**	viral vector
**WHO**	World Health Organization

3. BACKGROUND AND RATIONALE

3.1. Background

In December 2019, a cluster of patients with pneumonia of unknown cause was linked to a seafood wholesale market in Wuhan, China and were later confirmed to be infected with a novel coronavirus, known as 2019-nCoV [1]. The virus was subsequently renamed to SARS-CoV-2 because it is similar to the coronavirus responsible for severe acute respiratory syndrome (SARS-CoV), a lineage B betacoronavirus. SARS-CoV-2 shares more than 79% of its sequence with SARS-CoV, and 50% with the coronavirus responsible for Middle East respiratory syndrome (MERS-CoV), a member of the lineage C betacoronavirus [2]. COVID-19 is the illness caused by SARS-CoV-2. By January 2020 there was increasing evidence of human to human transmission as the number of cases rapidly began to increase in China. Despite unprecedented containment measures adopted by the Chinese government, SARS-CoV-2 rapidly spread across the world. The WHO declared the COVID-19 outbreak a public health emergency of international concern on 30th January 2020. As of 22nd April 2020, over 2.5 million cases have been reported with more than 177 000 deaths globally (Worldometers.info).

Coronaviruses (CoVs) are spherical, enveloped, large positive-sense single-stranded RNA genomes. One-fourth of their genome is responsible for coding structural proteins, such as the spike (S) glycoprotein, envelope (E), membrane (M) and nucleocapsid (N) proteins. E, M, and N are mainly responsible for virion assembly whilst the S protein is involved in receptor binding, mediating virus entry into host cells during CoVs infection via different receptors [3]. SARS-CoV-2 belongs to the phylogenetic lineage B of the genus *Betacoronavirus* and it uses the angiotensin-converting enzyme 2 (ACE2) as the entry receptor [4]. It is the seventh CoV known to cause human infections and the third known to cause severe disease after SARS-CoV and MERS-CoV.

The spike protein is a type I, trimeric, transmembrane glycoprotein located at the surface of the viral envelope of CoVs, which can be divided into two functional subunits: the N-terminal S1 and the C-terminal S2. S1 and S2 are responsible for cellular receptor binding via the receptor binding domain (RBD) and fusion of virus and cell membranes respectively, thereby mediating the entry of SARS-CoV-2 into target cells.[3] Neutralizing antibodies to SARS-CoV-2 are widely assumed to be correlated with recovery from infection, and the use of passively infused convalescent sera is being assessed for treatment of COVID-19. Such antibodies may protect from infection, as in vitro studies showed that cross-reactive SARS-CoV-1 antibodies prevented SARS-CoV-2 infection. The roles of S in receptor binding and membrane fusion, and the fact that it is the main target for neutralising antibodies, makes it an ideal target for vaccine and antiviral development. Furthermore, the potential of spike antibodies to mediate Fc effector functions has not been examined in SARS-CoV-2 vaccines,

nor extensively in any

related coronaviruses including SARS-CoV-1. Fc effector function is protective against Ebola and HIV as well as against respiratory diseases such as tuberculosis and Influenza (Saphire, et al., 2018; Lu, et al., 2016; Su, et al., 2019; Vanderven and Kent, 2020).

ChAdOx1 nCoV-19 vaccine consists of the replication-deficient simian adenovirus vector ChAdOx1, containing the structural surface glycoprotein (Spike protein) antigen of the SARS CoV-2 (nCoV-19), with a leading tissue plasminogen activator (tPA) signal sequence. ChAdOx1 nCoV-19 expresses a codon-optimised coding sequence for the Spike protein from genome sequence accession GenBank: MN908947. The tPA leader sequence has been shown to be beneficial in enhancing immunogenicity of another ChAdOx1 vectored CoV vaccine (ChAdOx1 MERS) [5].

3.2. Pre-Clinical Studies

3.2.1. Immunogenicity (Jenner Institute, unpublished)

Mice (balb/c and CD-1) were immunised with ChAdOx1 expressing SARS-CoV-2 Spike protein or green fluorescent protein (GFP). Spleens were harvested for assessment of IFY ELISpot responses and serum samples were taken for assessments of S1 and S2 antibody responses on ELISA at 9 or 10 days post vaccination. The results of this study show that a single dose of ChAdOx1 nCoV was immunogenic in mice.

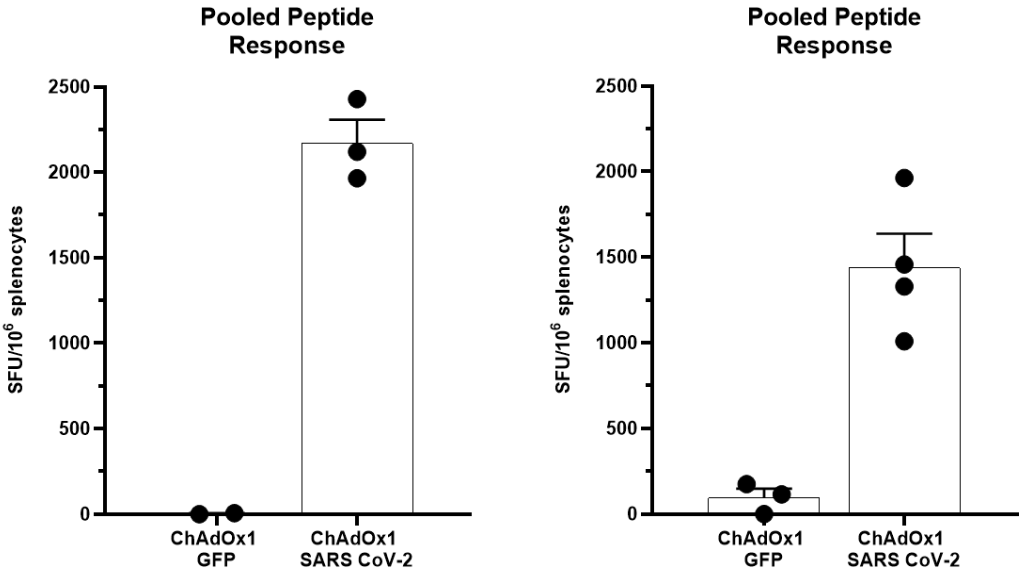

Figure 1. Summed splenic IFN-γ ELISpot responses of BALB/c (left panel) and CD-1 (right panel) mice, in response to peptides spanning the spike protein from SARS-CoV-2, nine or ten days post vaccination, with 1.7 × 10^{10} viral particles (vp) ChAdOx1 nCoV-19 or 8 × 10^{9} vp ChAdOx1 GFP.

Mean with SEM are depicted.

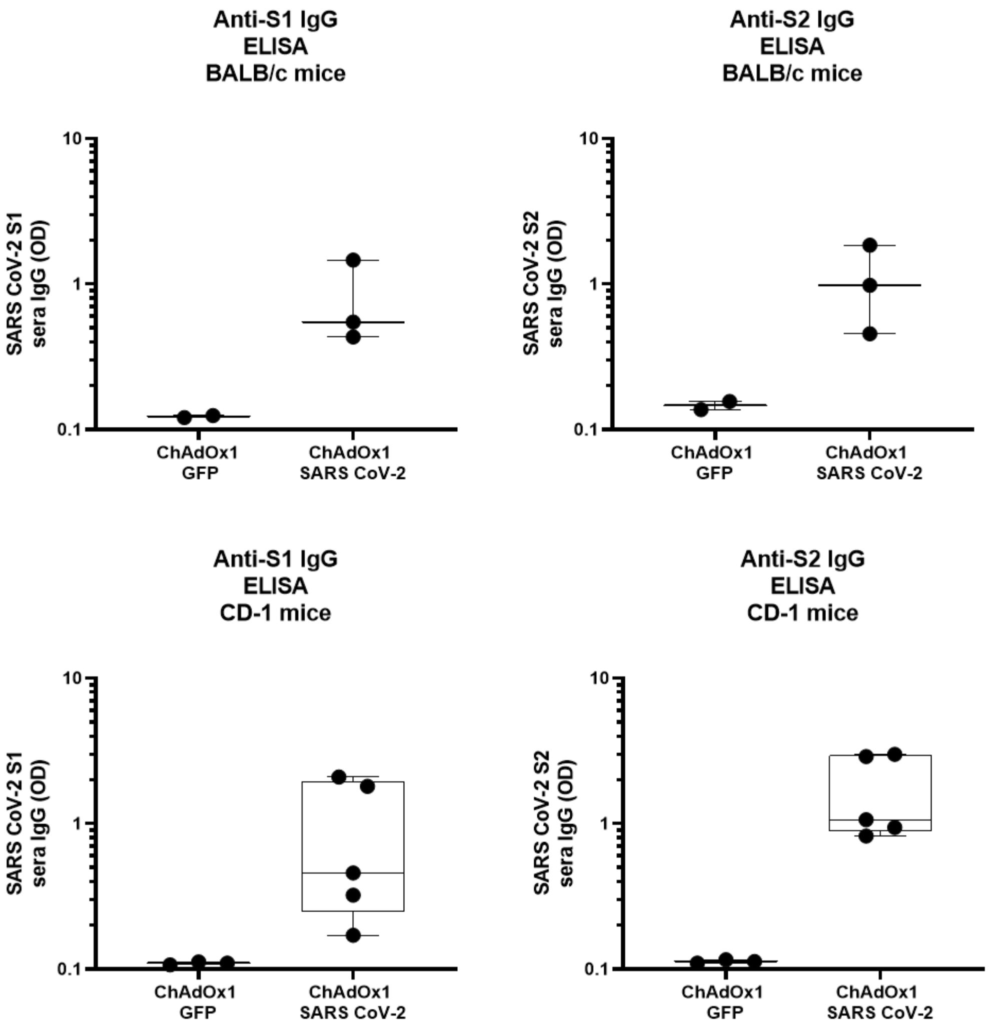

Figure 2. Box and whisker plot of the optical densities following ELISA analysis of BALB/C mouse sera (Top panel) incubated with purified protein spanning the S1 domain (left) or purified protein spanning the S2 domain (right) of the SARS-CoV-2 spike nine or ten days post vaccination, with 1.7×10^{10} vp ChAdOx1 nCoV-19 or 8×10^{9} vp ChAdOx1 GFP. Box and whisker plots of the optical densities following ELISA analysis of CD-1 mouse sera (Bottom panel) incubated with purified protein spanning the S1 domain (left) or purified protein spanning the S2 domain (right) of the SARS-CoV-2 spike.

3.2.2. Efficacy

Pre-clinical efficacy studies of ChAdOx1 nCoV-19 in ferrets and non-human primates are underway. Results will be included in an updated Investigator's Brochure when available.

3.3. Antibody Dependent Enhancement

Safety concerns around the use of full length coronavirus Spike glycoproteins and other viral antigens(nucleoprotein) as a vaccine antigen have been raised following historical and limited reports of immunopathology and antibody dependent enhancement (ADE) reported in vitro and post SARS-CoV challenge in mice, ferrets and non-human primates immunised with whole SARS-CoV inactivated or full-length S protein based vaccines, including a study using Modified Vaccinia Ankara as a vector [6-8]. To date, there has been one report of lung immunopathology following MERS-CoV challenge in mice immunised with an inactivated MERS-CoV candidate vaccine [9]. However, in preclinical studies of ChAdOx1 immunisation and MERS-CoV challenge, no ADE was observed in hDPP4 transgenic mice, dromedary camels or non-human primates (van Doremalen et al, manuscript submitted) [10, 11].

The risks of inducing lung immunopathology in the event of COVID-19 following ChAdOx1 nCoV-19 vaccination are unknown. Challenge studies on ferrets and NHPs are underway and these pre-clinical studies will report on presence or absence of lung pathology. Results will be reviewed as soon as they emerge and will inform discussions on risk/benefit to participants receiving the Investigational Medical Product (IMP). All pathology data arising from challenge studies of other SARS-CoV-2 vaccine candidates will also be taken into account.

3.4. Previous clinical experience

The phase I/II study in health adults in the UK, initiated in late April 2020 is the first-in-human study employing ChAdOx1 nCoV-19, and as of mid-June 2020 had enrolled more than 7000 participants. Furthermore, ChAdOx1 vectored vaccines expressing different inserts have previously been used in over 320 healthy participants taking part in clinical trials conducted by or in partnership with the University of Oxford in the UK, Switzerland Uganda and Saudi Arabia (Table 1, Table 2). Most importantly, a ChAdOx1 vectored vaccine expressing the full-length Spike protein from another Betacoronavirus, MERS-CoV, has been given to 31 participants to date as part of MERS001 and MERS002 trials. ChAdOx1 MERS was given at doses ranging from $5x10^9$ vp to $5x10^{10}$ vp (table 2) with no serious adverse reactions reported. Further safety and immunogenicity results on ChAdOx1 MERS can be found on the Investigator's Brochure for ChAdOx1 nCoV-19 for reference.

Clinical trials of ChAdOx1 vectored vaccines encoding antigens for Influenza (fusion protein NP+M1), Tuberculosis (85A), Prostate Cancer (5T4), Malaria (LS2), Chikungunya (structural polyprotein), Zika (prM and E), MERS-CoV (full-length Spike protein) and Meningitis B are listed below.

None of the below mentioned clinical trials reported serious adverse events associated with the administration of ChAdOx1, which was shown to have a good safety profile.

Table 1: Clinical experience with ChAdOx1 viral vector vaccines.

Country	Trial	Vaccine	Age	Route	Dose	Number of Participants (Received ChAdOx1)	Publication / Registration Number
UK	FLU004	ChAdOx1 NP+M1	18-50	IM	$5x10^{8}$ vp	3	Antrobus et al, 2014. Molecular Therapy. DOI: 10.1038/mt.2013.284 [12]
					$5x10^{9}$ vp	3	
					$2.5x10^{10}$ vp	3	
					$5x10^{10}$ vp	6	
UK	FLU005	ChAdOx1 NP+M1<br>MVA NP+M1 (week 8)	18-50	IM	$2.5x10^{10}$ vp	12	Coughlan et al, 2018. EBioMedicine DOI: 10.1016/j.ebiom.2018.02.011 DOI: 10.1016/j.ebiom.2018.05.001 [13]
		ChAdOx1 NP+M1<br>MVA NP+M1 (week 52)	18-50	IM	$2.5x10^{10}$ vp	12	
		MVA NP+M1<br>ChAdOx1 NP+M1 (week 8)	18-50	IM	$2.5x10^{10}$ vp	12	
		MVA NP+M1<br>ChAdOx1 NP+M1 (week 52)	18-50	IM	$2.5x10^{10}$ vp	9	
		ChAdOx1 NP+M1	>50	IM	$2.5x10^{10}$ vp	12	
		ChAdOx1 NP+M1<br>MVA NP+M1 (week 8)	>50	IM	$2.5x10^{10}$ vp	12	

Country	Trial	Vaccine	Age	Route	Dose	Number of Participants (Received ChAdOx1)	Publication / Registration Number
UK	TB034	ChAdOx1 85A	18-50	IM	$5x10^{9}$ vp	6	Wilkie et al, 2020 Vaccine DOI: 10.1016/j.vaccine.2019.10.102 [14]
					$2.5x10^{10}$ vp	12	
		ChAdOx1 85A MVA85A (week 8)	18-50	IM	$2.5x10^{10}$ vp	12	
		ChAdOx1 85A (x2, 4weeks apart) MVA85A (at 4 months)	18-50	IM	$2.5x10^{10}$ vp	12	
Switzerland	TB039 (ongoing)	ChAdOx1 85A	18-55	Aerosol	$1x10^{9}$ vp	3	Clinicaltrials.gov: NCT04121494
				Aerosol	$5x10^{9}$ vp	3	
				Aerosol	$1x10^{10}$ vp	11	
				Aerosol/IM	$1x10^{10}$ vp	15	
Uganda	TB042 (ongoing)	ChAdOx1 85A	18-49	IM	$5x10^{9}$ vp	6	Clinicaltrials.gov: NCT03681860
					$2.5 x10^{10}$	6	
UK	VANCE01	ChAdOx1.5T4 MVA.5T4	18 – 75	IM	$2.5x10^{10}$ vp	34	Clinicaltrials.gov: NCT02390063

Country	Trial	Vaccine	Age	Route	Dose	Number of Participants (Received ChAdOx1)	Publication / Registration Number
UK	ADVANCE (ongoing)	ChAdOx1.5T4<br>MVA.5T4	≥18	IM	$2.5x10^{10}$ vp	23 (as of Feb 20)	Clinicaltrials.gov:<br>NCT03815942
UK	VAC067	ChAdOx1 LS2	18-45	IM	$5x10^{9}$ vp	3	Clinicaltrials.gov:<br>NCT03203421
					$2.5x10^{10}$ vp	10	
UK	VAMBOX	ChAdOx1 MenB.1	18-50	IM	$2.5x10^{10}$ vp	3	ISRCTN46336916
					$5x10^{10}$ vp	26	
UK	CHIK001	ChAdOx1 Chik	18-50	IM	$5x10^{9}$ vp	6	Clinicaltrials.gov:<br>NCT03590392<br>DOI: https://doi.org/10.4269/ajtmh.abstract2019<br>Abstract #59, page 19.
					$2.5x10^{10}$ vp	9	
					$5x10^{10}$ vp	9	
UK	ZIKA001 (ongoing)	ChAdOx1 Zika	18-50	IM	$5x10^{9}$ vp	6	Clinicaltrials.gov:<br>NCT04015648
					$2.5x10^{10}$ vp	3 (as of Feb 20)	
					$5x10^{10}$ vp	-	

Table 2: Clinical experience with ChAdOx1 MERS vaccine

Country	Trial	Vaccine	Age	Route	Dose	Number of Participant s (Received ChAdOx1)	Publication / Registration Number
UK	MERS001 (ongoing)	ChAdOx1 MERS	18-50	IM	$5x10^{9}$ vp	6	Clinicaltrials.gov: NCT03399578<br>DOI: https://doi.org/10.4269/ajtmh.abstract2018<br>Abstract#973, page 305.<br>Folegatti et.al. 2020, Lancet Infect.Dis, *In press*.
					$2.5x10^{10}$ vp	9	
					$5x10^{10}$ vp	9	
					$2.5x10^{10}$ vp (homologous prime-boost)	3	
Saudi Arabia	MERS002 (ongoing)	ChAdOx1 MERS	18-50	IM	$5x10^{9}$ vp	4	Clinicaltrials.gov: NCT04170829
					$2.5x10^{10}$ vp	3	
					$5x10^{10}$ vp	-	

3.5. Rationale

The COVID-19 pandemic has caused major disruption to healthcare systems with significant socioeconomic impacts. Containment measures have failed to stop the global spread of virus. There are currently no specific treatments available against COVID-19 and accelerated vaccine development is urgently needed. South Africa is still at an early stage of its COVID-19 outbreak, which is expected to start peaking toward the end of July 2020, but has already documented 3,500 cases and 58 deaths as of 22 April 2020 (Wordometer.info). Recent modelling data indicates that globally there are likely to be 3-4 waves of COVID-19 outbreaks, possibly extending through to 2022.

Live attenuated viruses have historically been among the most immunogenic platforms available, as they have the capacity to present multiple antigens across the viral life cycle in their native conformations. However, manufacturing live-attenuated viruses requires complex containment and biosafety measures. Furthermore, live-attenuated viruses carry the risks of inadequate attenuation causing disseminated disease, particularly in immunocompromised hosts. Given that severe disease and fatal COVID-19 disproportionally affect older adults with co-morbidities, making a live-attenuated virus vaccine is a less viable option. Replication competent viral vectors could pose a similar threat for disseminated disease in the immuno-suppressed. Replication deficient vectors, however, avoid that risk while maintaining the advantages of native antigen presentation, elicitation of T cell immunity and the ability to express multiple antigens [15]. Subunit vaccines usually require the use of adjuvants and whilst DNA and RNA vaccines can offer manufacturing advantages, they are often poorly immunogenic requiring multiple doses, which is highly undesirable in the context of a pandemic.

Chimpanzee adenovirus vaccine vectors have been safely administered to thousands of people using a wide range of infectious disease targets. ChAdOx1 vectored vaccines have been given to over 320 participants with no safety concerns and have been shown to be highly immunogenic at single dose administration. Of relevance, a single dose of a ChAdOx1 vectored vaccine expressing full-length spike protein from another betacoronavirus (MERS-CoV) has shown to induce neutralising antibodies in recent clinical trials (Folegatti et. Al. 2020. Lancet Infect Dis, In press).

A Phase I single-blind, randomised controlled trial in the UK of ChAdOx1 nCoV-19 enrolled healthy adults aged 18–55 years with no history of laboratory confirmed SARS-CoV-2 infection or of COVID-19-like symptoms, who were randomly assigned (1:1) to receive ChAdOx1 nCoV-19 at a dose of 5×10^{10} viral particles or MenACWY as a single intramuscular injection. Local and systemic reactions were more common in the ChAdOx1 nCoV-19 group and many were reduced by use of prophylactic paracetamol, including pain, feeling feverish, chills, muscle ache, headache, and malaise. There were no serious adverse events related to ChAdOx1 nCoV-19. In the ChAdOx1 nCoV-19 group, spike-specific T-cell responses peaked on day 14 (median 856 spot-forming cells per million peripheral blood mononuclear

cells, IQR 493–1802; n=43). Anti-spike IgG responses rose by day 28 (median 157 ELISA units [EU], 96–317; n=127), and were boosted following a second dose (639 EU, 360–792; n=10). Neutralising antibody responses against SARS-CoV-2 were detected in 32 (91%) of 35 participants after a single dose when measured in MNA80 and in 35 (100%) participants when measured in PRNT50. After a booster dose, all participants had neutralising activity (nine of nine in MNA80 at day 42 and ten of ten in Marburg VN on day 56). Neutralising antibody responses correlated strongly with antibody levels measured by ELISA ($R^2=0{\cdot}67$ by Marburg VN; $p<0{\cdot}001$).[16] These data support the decision to pursue a two dose schedule for evaluation of efficacy of the vaccine candidate. Hence, this protocol has been adapted as such, with all Group now assigned to receive either two doses of ChAdOx1 nCoV-19 or placebo (Folegatti et al, Lancet 2020).

The trial to be conducted in South Africa will enroll adults living without and with HIV to assess safety, immunogenicity and efficacy two doses of ChAdOx1-nCoV-19. The South Africa study on ChAdOx1-nCoV-19 (Group 1 enrolment) was initiated following review by the Data and Safety Monitoring Committee (which oversees multiple ChAdOx1 nCoV-19 including the UK, South African and a planned study in Kenya) of the initial safety cohort (n=50) that will be enrolled in the UK. Enrolment into Group-1 of the study in South Africa occurred in tandem with opening of enrolment of the expanded immunogenicity and "efficacy-cohort" in the UK.

Recent guidelines from the Food and Drug Administration on conduct of COVID-19 vaccine trials recommend that "although establishing vaccine safety and efficacy in SARS-CoV-2 naïve individuals is critical, vaccine safety and COVID-19 outcomes in individuals with prior SARS-CoV-2 infection, which might have been asymptomatic, is also important to examine because re-vaccination screening for prior infection is unlikely to occur in practice with the deployment of licensed COVID-19 vaccines. Therefore, COVID-19 vaccine trials need not screen for or exclude participants with history or laboratory evidence of prior SARS-CoV-2 infection. However, individuals with acute COVID-19 (or other acute infectious illness) should be excluded from COVID-19 vaccine trials".[17]

4. OBJECTIVES AND ENDPOINTS

In adults without HIV (HIV-uninfected)

Primary objective:

To assess the safety of the candidate vaccine ChAdOx1 nCoV-19 in healthy HIV-uninfected adults.

Co-primary objective:

To assess efficacy of the candidate ChAdOx1 nCoV-19 against COVID-19, defined as virologically confirmed (PCR positive) COVID-19 disease, in participants that were COVID-19 naïve at the time of randomization and who received two doses of ChAdOx1 nCoV-19 or placebo. Events will be included if they occurred more than 14 days after the booster dose. "COVID-19 naïve" will be defined as sero-negative and tested negative for SARS-CoV-2 infection, based on a high sensitivity serology antibody

test and molecular detection testing of nasal swab, respectively.

Secondary objective

To assess the immunogenicity of ChAdOx1 nCoV-19 in healthy HIV-uninfected adults

Table 3: Details of objectives Groups 1 & 2 (HIV-uninfected):

**Objective**	**Objective details**	**Endpoint measures**
Primary Objective (Group 1 and Group 2 a and b)	To assess the safety, tolerability and reactogenicity profile of the candidate vaccine ChAdOx1 nCoV-19	a) occurrence of solicited local reactogenicity signs and symptoms for 7 days following vaccination;<br>b) occurrence of solicited systemic reactogenicity signs and symptoms for 7 days following vaccination;<br>c) occurrence of unsolicited adverse events (AEs) for 28 days following vaccination;<br>d) change from baseline for safety laboratory measures and;<br>e) occurrence of serious adverse events<br>e) occurrence of disease enhancement episodes
Co- Primary objective<br>(Group 2a and 2b; efficacy cohort)	To assess efficacy of the candidate ChAdOx1 nCoV-19 against all-severity COVID-19	The **primary efficacy [objective] and endpoint** include PCR positive COVID-19 disease cases occurring in participants that were COVID-19 naïve at randomization and who received two doses of ChAdOx1 nCoV-19 or placebo. Events will be included if they occurred more than 14 days after the booster dose.<br>Virologically-confirmed COVID-19 clinical disease will be defined as an acute respiratory illness that is clinically consistent with COVID-19 based on presence of criteria indicated in Table 4 and<br>Table 5 **AND** a positive SARS-CoV-2 specific reverse transcriptase polymerase chain reaction (RT-PCR)

**Secondary efficacy [objectives], endpoints**	To assess efficacy of the candidate ChAdOx1 nCoV-19 against COVID-19 of differing severity	**Secondary efficacy [objectives], endpoints** in for the overall population and stratified by COVID-19 serological status at randomisation include:<br>a. VE in preventing virologically-confirmed COVID-19 clinical disease including all cases occurring onward from 21 days after a single dose.<br>b. VE in preventing virologically-confirmed COVID-19 clinical disease occurring more than 14 days after a second dose for the overall population and those that were sero-positive at baseline.<br>c. VE in preventing PCR positive COVID-19 disease cases.<br>d. VE in preventing severe confirmed COVID-19 disease.<br>e. VE in preventing virologically-confirmed moderate-severe COVID-19 clinical disease.<br>f. VE in preventing hospitalization due to virologically confirmed COVID-19 disease<br>g. VE in preventing death associated with virologically-confirmed COVID-19 clinical disease<br>h. VE in preventing] all-cause LRTI (overall and stratified by hospitalization or not, irrespective of test result for SARS-COV-2.<br>i. VE using Oxford Primary Outcome definition (PCR+ at least one symptom of fever > 37.8oC, cough, shortness of breath, anosmia, aguesia.)
Secondary objective (Group 1 and Group 2)	To assess cellular and humoral immunogenicity of ChAdOx1 nCoV-19	a) Enzyme-linked immunosorbent assay (ELISA) or fluorescence based micro-bead immunosorbent assay on luminex platform to quantify antibodies against SARS-CoV-2 spike protein (seroconversion rates)<br>b) Interferon-gamma (IFN-γ) enzyme- linked immunospot (ELISpot) responses to SARS-CoV-2 spike protein<br>c) Virus neutralising antibody (NAb) assays against live and/or pseudotyped SARS-CoV-2 virus<br>d) Th1 and Th2 cytokine response profile at 3-4 days after vaccination.
Exploratory immunology:	To assess B cell responses to SARS-CoV-2 spike trimer and/or the receptor binding domain	a.Cellular Fc effector functionality assays to measure the ability of vaccine elicited antibodies to mediate cellular cytotoxicity, complement deposition, and phagocytosis.<br>b. Flow cytometric sorting of plasmablasts and memory B cells to using spike and receptor binding domain “baits” to isolate SARS-CoV-2 specific B cells, sequence their immunoglobulin genes and define their epitope specificity.

Table 4: Symptoms of Suspected COVID-19

**Respiratory**	**Non-Respiratory**
New onset cough	Fever or feverishness (defined subjectively, or objective fever ≥ 37.8°C, regardless of use of anti-pyretic medications)
New onset rapid breathing	Myalgia (or muscle ache)
New onset shortness of breath (or breathlessness or difficulty breathing)	Chills
Sore throat	Loss of taste (or taste disturbance)
Loss of smell (or smell disturbance)	Headache
Nasal congestion	Diarrhea
Runny nose	Tiredness (or fatigue or weakness)
	Nausea or vomiting
	Loss of appetite

Abbreviations: COVID-19 = coronavirus disease 2019.

Table 5: Efficacy Endpoint Definitions of COVID-19 Severity

COVID-19 Severity	Endpoint Definitions
**Mild**	Any one of:<br>• Fever (defined by subjective or objective measure, regardless of use of anti-pyretic medications)<br>• New onset cough<br>• ≥ 2 COVID-19 respiratory/non-respiratory symptoms in Table 4<br>**AND**<br>• Does not meet criteria for moderate or severe
**Moderate**	≥ 1 of:<br>• Fever (≥ 37.8°C) + any 2 COVID-19 symptoms in table 4 for ≥ 3 days (need not be contiguous days)<br>• High fever (≥ 38.4°C) for ≥ 3 days (need not be contiguous days)<br>• Any evidence of significant LRTI:<br>– Shortness of breath (or breathlessness or difficulty breathing) with or without exertion (beyond baseline)<br>– Tachypnea: 20 to 29 breaths per minute at rest<br>– SpO_2: < 94% on room air<br>– Abnormal chest x-ray/CT consistent with pneumonia or LRTI<br>– Adventitious sounds on lung auscultation
**Severe**	≥ 1 of:<br>• Tachypnea: ≥ 30 breaths per minute at rest<br>• SpO_2: < 92% on room air or PAO_2/FiO_2 < 300<br>• High flow oxygen therapy, CPAP, or NIV (eg, CPAP/BiPAP)<br>• Mechanical ventilation or ECMO<br>• One or more major organ system failure[a] (eg, cardiac/circulatory, pulmonary, renal, hepatic to be defined by diagnostic testing/clinical syndrome/interventions)

Abbreviations: BiPAP = bi-level positive airway pressure; CPAP = continuous positive air pressure; CT = computed tomography; ECMO = extracorporeal membrane oxygenation; FiO_2 = fraction of inspired oxygen; LRTI = lower respiratory tract infection; NIV = non-invasive ventilation; PAO_2 = partial pressure of oxygen in the alveolus; SpO_2 = oxygen saturation.

Evidence of major organ dysfunction or failure includes but is not limited to any of acute respiratory distress syndrome (ARDS), acute renal failure, acute hepatic failure, acute right or left heart failure, septic or cardiogenic shock, or requirement for vasopressors, systemic corticosteroids, or hemodialysis.

In adults living with HIV (HIV-infected)

Primary co-objectives:

- To assess the safety of the candidate vaccine ChAdOx1 nCoV-19 in adults living with HIV.
- To evaluate the immunogenicity of ChAdOx1 nCoV-19 after first and second doses of vaccine in adults living with HIV.

Table 6: Details of objectives Groups 3 (HIV-infected):

**Objective**	**Objective details**	**Endpoint measures**
Primary objective	To assess the safety, tolerability and reactogenicity profile of the candidate vaccine ChAdOx1 nCoV-19 in people living with HIV	a) occurrence of solicited local reactogenicity signs and symptoms for 7 days following vaccination;<br>b) occurrence of solicited systemic reactogenicity signs and symptoms for 7 days following vaccination;<br>c) occurrence of unsolicited adverse events (AEs) for 28 days following vaccination;<br>d) change from baseline for safety laboratory measures and;<br>e) occurrence of serious adverse events;<br>f) occurrence of disease enhancement episodes
Co-primary objective	To assess cellular and humoral immunogenicity of ChAdOx1 nCoV-19 in people living with HIV after one and two doses of vaccine	a) Enzyme-linked immunosorbent assay (ELISA) or fluorescence based micro-bead immunosorbent assay on luminex platform to quantify antibodies against SARS-CoV-2 spike protein (seroconversion rates)<br>b) Interferon-gamma (IFN-γ) enzyme-linked immunospot (ELISpot) responses to SARS-CoV-2 spike protein<br>c) Virus neutralising antibody (NAb) assays against live and/or pseudotyped SARS-CoV-2 virus<br>d) Th1 and Th2 cytokine response profile at 3-4 days after vaccination.
Secondary objective	To descriptively compare immune responses to ChAdOx1 nCoV-19 in people living with HIV to HIV-uninfected individuals, overall and stratified by COVID-19 sero-status at enrolment.	a) Enzyme-linked immunosorbent assay (ELISA) or fluorescence based micro-bead immunosorbent assay on luminex platform to quantify antibodies against SARS-CoV-2 spike protein (seroconversion rates)<br>b) Interferon-gamma (IFN-γ) enzyme-linked immunospot (ELISpot) responses to SARS-CoV-2 spike protein<br>c) Virus neutralising antibody (NAb)

		assays against live and/or pseudotyped SARS-CoV-2 virus<br>d) Th1 and Th2 cytokine response profile at 3-4 days after vaccination.
Exploratory immunology:	To assess B cell responses to SARS-CoV-2 spike trimer and/or the receptor binding domain	a. Cellular Fc effector functionality assays to measure the ability of vaccine elicited antibodies to mediate cellular cytotoxicity, complement deposition, and phagocytosis.<br>b. Flow cytometric sorting of plasmablasts and memory B cells to using spike and receptor binding domain “baits” to isolate SARS-CoV-2 specific B cells, sequence their immunoglobulin genes and define their epitope specificity.

5. TRIAL DESIGN

This is a Phase I/II, double-blinded, placebo-controlled, individually randomized study in adults aged 18-65 years living with and without HIV in South Africa. ChAdOx1 nCoV-19 or placebo will be administered via an intramuscular injection into the deltoid. The protocol has been adapted to confirm that the study will assess safety, immunogenicity and efficacy of two doses of ChAdOx1 nCoV-19 based on the phase I study results from the UK immunogenicity cohort.[16] For Group-1 (HIV-uninfected adults; n=70) and Group-3 (HIV-infected adults; n=100), a two dose schedule spaced 21-35 days apart will be evaluated for safety and immunogenicity. In Group II (phase II; immunogenicity and efficacy cohort), we will target enrolling 1900 participants to accrue sufficient number of endpoints to analyze for efficacy of at least 60% (and a lower bound of >0%) and 80% power assuming an attack rate of 3.5% for COVID-19 in the placebo arm (see sample size section). Based on the endpoint case accrual and the trajectory of the epidemic in South Africa, the sample size may be adjusted in relation to number of endpoints being accrued. Participants already enrolled prior to implementation of Version 3.0 of the protocol, that tested positive for SARS-CoV-2 by PCR at randomization will continue on the study, including all further scheduled visits and procedures. However, an equal number of additional participants that test negative for SARS-CoV-2 on PCR testing will be enrolled into the study.

The three trial groups are detailed in Table 7, with an overall sample size of ~2070. Randomisation will take place at an intervention to placebo ratio of 1:1 in blocks of 8 and all participants and clinical study staff will be blinded to IP or placebo. Site pharmacists and the person administering the allocated IP/placebo will be unblinded. Once group 1 is fully recruited, safety data will be reviewed by DSMC. Group 3 enrolment will follow on from group 1 enrolment. This decision will be guided by DSMC review of COV001 trial in the UK. Initiation of enrolment into Group 2 will be contingent upon review by the joint DSMC of the ongoing study in the UK, which will also ultimately inform whether to pursue a single or two-dose schedule for the efficacy-cohort in South Africa.

Participants will be followed over the duration of the study (through to 365 days post-randomization) to record adverse events and episodes of virologically confirmed symptomatic COVID-19. Participants will be tested for SARS-CoV-2 infection if they present with a new onset of symptoms suggestive of COVID-19 (Table 4) throughout the duration of their participation.

Detailed clinical parameters will be collected from medical records (or examination by study-staff) and aligned with the COVID-19 score; Table 5. These include measuring severity based on oxygen saturation, need for oxygen therapy, respiratory rate and other vital signs, need for ventilatory support, X-ray imaging and blood test results, amongst other clinically relevant parameters; Table 5.

Safety will be assessed in real time and at least monthly interim reviews by the DSMC will be scheduled after Group 1 (70 HIV-uninfected) participants received the IP (dose 1 and dose 2 if given), after enrolment of 100 HIV-infected adults (Group-3) and once all Group-2 participants are enrolled. The DSMC will periodically assess safety and efficacy data every 4-8 weeks and/or its discretion. All deaths and any serious adverse event considered to be study-related will be reviewed by the DSMC within 72 hours of site reporting of such cases to the DSMC (which will occur within 24 hours of site identification on any such cases).

Table 7: Trial groups

Group #	Group description	Objective	Follow up	Treatment	Vaccination schedule
1 (n=70)	People without HIV (HIV-uninfected)	Intensive Safety and immunogenicity	Intensive	ChAdOx1 nCoV-19 5-7.5x1010 vp; OR Normal saline (0.9% NaCl)	2* doses, 4 weeks (21-35 days) apart
2a (n=250)[§]	People without HIV (HIV-uninfected)	Safety, intensive immunogenicity and vaccine efficacy	Extended	ChAdOx1 nCoV-19 5-7.5x10^{10} vp; OR Normal saline (0.9% NaCl)	2* doses, 4 weeks (21-35 days) apart
2b (n=1650)[§]	People without HIV (HIV-uninfected)	Safety, immunogenicity and vaccine efficacy	Extended	ChAdOx1 nCoV-19 5-7.5x10^{10} vp; OR Normal saline (0.9% NaCl)	2* doses, 4 weeks (21-35 days) apart
3 (n=100)	People living with HIV (HIV-infected),	Intensive Safety and immunogenicity	Intensive	ChAdOx1 nCoV-19 5-7.5x10^{10} vp; OR Normal saline (0.9% NaCl)	Prime-boost<br>2* doses, 4 weeks (21-35 days) apart

[¥]Sample size increased from 50 to 70, following higher than anticipated positivity for SARS-CoV-2 infection (six of initial 24 randomized into the study), to accommodate for non-evaluable (i.e. not COVID-19 naive) participants.

*Participants will receive 2 doses of the same injection (EITHER IP or placebo) as assigned at randomization.

[§]Numbers will be increased to supplement for corresponding number of individuals randomized prior to implementation of Version 3.0 of the protocol that tested positive for SARS-CoV-2 on PCR at time of randomization.

[#] Following a review of the initial safety/immunogenicity trial being conducted in the UK; COV0001 trial, and review of the initial safety/ immunogenicity trial COV0001 by the DSMC, it was decided that Group 2 in this trial will receive 2 doses of assigned study intervention. SAHPRA and WHREC have already been informed of Group 2 receiving a two-dose schedule based on the earlier version of the protocol.

Also, considering the unpredictability of the force of SARS-CoV-2 infection and the lower than anticipated attack rate for the primary-endpoint cases in the study being undertaken in the UK, the sample size for Group 2 (efficacy cohort) has been expanded. This will involve enrolling up to a total of 1900 people in Group-2, which will provide 80% power to detect at least a 60% vaccine efficacy (lower bound of 95%CI >0) with an attack rate of 3.5% in the placebo arm. Ongoing review of the number of COVID-19 endpoint cases accrued during the course of the study, may lend itself to enrolling smaller number of participants should the attack rate be higher than 3.5%. The sample size for Group 1 has been increased to 70 to accommodate for the higher than anticipated infection rate with SARS-

CoV-2 (6 of the initial 24 participants randomized in group 1). Similarly, in anticipation of approximately one-third of Group-3 participants possibly being already infected with SARS-CoV-2, the sample size will be increased to 100 to have approximately 30 sero-negative vaccinees and placebo recipients enrolled into the study.

5.1. Trial participants

Adult participants (healthy HIV-uninfected; and generally well people living with HIV [Group 3]) aged 18-65 years will be enrolled. Participants will be considered enrolled immediately following randomization to receive first vaccination.

5.2. Definition of End of Trial

The end of the trial is the date of the last assay conducted on the last sample collected.

5.3. Duration of study

The total duration of the study will be 12 months from the day of enrolment for all participants.

5.4. Potential Risks for participants

The potential risks are those associated with phlebotomy, vaccination and disease enhancement

5.4.1. Venipuncture

Localised bruising and discomfort can occur at the site of venipuncture. Infrequently fainting may occur. These will not be documented as AEs if they occur. The total volume of blood drawn over a six-month period will be 160- 315mL (blood volumes may vary slightly for participants at different investigator sites due to use of different volume vacutainers, following local SOPs). This should not compromise these participants, as they would donate 470mL during a single blood donation for the National Blood transfusion Service over a 3-4-month period. Participants will be asked to refrain from blood donation for the duration of their involvement in the trial.

5.4.2. Allergic reactions

Allergic reactions from mild to severe may occur in response to any constituent of a medicinal product's preparation. Anaphylaxis is extremely rare (about 1 in 1,000,000 vaccine doses) but can occur in response to any vaccine or medication.

5.4.3. Vaccination

Local reaction from IM vaccination

The typical local reaction as a result of IM injection is temporary pain, tenderness, redness, and swelling at the site of the injection.

Systemic reactions

Constitutional influenza-like symptoms such as fatigue, headache, malaise, feverishness, and muscle aches can occur with any vaccination and last for 2-3 days. Presyncopal and syncopal episodes may occur at the time of vaccination which rapidly resolve. As with any other vaccine, temporary ascending paralysis (Guillain-Barré syndrome, GBS) or immune mediated reactions that can lead to organ damage may occur, but this should be extremely rare (1 in 100,000-1,000,000 vaccine doses).

Control participants will receive a placebo injection containing sterile normal saline (0.9% NaCl). The volume of the IP and placebo injections will be equal.

5.4.4. Disease Enhancement

The risks of inducing disease enhancement and lung immunopathology in the event of COVID-19 disease following ChAdOx1 nCoV-19 vaccination are unknown. Challenge studies on ferrets and Non-human primates (NHPs) are underway and results will be reviewed as they emerge. All pre-clinical data from challenge studies using ChAdOx1 nCoV-19 and other vaccine candidates (when available) will inform decisions on risks and benefits to participants receiving the IP.

5.5. Known Potential Benefits

Recipients of ChAdOx1 nCoV-19 do not have any guaranteed benefit. However, the information gained from this study could contribute to the development of a safe and effective vaccine against COVID-19. IP recipients may benefit from receipt of the ChAdOx1 nCoV-19 vaccine if the vaccine is found to be effective against reducing COVID-19. Placebo recipients will not benefit from receipt of placebo, however, may benefit from regular follow-up during the SARS-CoV-2 pandemic as they will be tested for infection if they are symptomatic.

6. RECRUITMENT AND WITHDRAWAL OF TRIAL PARTICIPANTS

6.1. Identification of Trial Participants

Adults in South Africa will be recruited by the following methods:

- Research sites will utilize databases available in the research units which contain contact details of participants or parents of participants in previous (completed) vaccine trials.
- Adverts, approved by local ethics committee, may be utilized and places in health care clinics and other public places.
- Radio announcements
- Community engagement via the community action boards affiliated to the research sites

6.2. Informed consent

All participants will have the opportunity to read the information sheet prior to or during screening visit. An 'assessment of understanding' (AOU) will be completed by participant to assess their understanding of participant information sheet. Participants will have the opportunity to discuss the trial information with investigators and family members. Informed consent will be signed and dated before any study specific procedures are performed. The informed consent process will be undertaken in two stages. In the first instance, following brief introduction about the study at the screening visit, including inclusion and exclusion criteria, volunteers will be asked to consent to procedures to determine their eligibility for possible randomization (full-study participation). This will include collection of key demographic information, a brief clinical history, testing inter alia for HIV-1 infection (except for Group 3), Hepatitis B surface antigen (HBsAG) positivity, evidence for current (by molecular detection) infection with SARS-CoV-2, pregnancy test (for women of reproductive age group), HbA1C (glycosylated hemoglobin) as well as general physical well-being (including blood pressure check). This approach has been adopted to accommodate for the higher than anticipated number of participants (six of 24) that tested positive for SARS-CoV-2 infection by PCR after having started enrolment of Group-1 participants.

Following fulfilment of inclusion and exclusion criteria based on findings from the screening visit, those who remain eligible and agree to undergo randomization into the full study, will be consented further for study participation. Screened participants will be encouraged to take complete informed (enrolment) consent forms home from the screening visit, and read and discuss the trial and their possible involvement in the trial with family. At the randomization visit, the participant will be fully informed of all aspects of the trial, the potential risks and their obligations. The following general principles will be emphasized:

- Participation in the study is entirely voluntary
- Refusal to participate involves no penalty or loss of medical benefits
- The participant may withdraw from the study at any time.
- The participant is free to ask questions at any time to allow him or her to understand the purpose of the study and the procedures involved
- The study involves research of an investigational vaccine
- There is no direct benefit to the participant from participating
- Participants will be asked to provide detailed medical and surgical history to investigator

verbally and if possibly, patient-held medical records (outpatient cards) will be reviewed.

- Blood samples taken as part of the study may be sent to laboratories outside South Africa (e.g. University of Oxford and InNexus BioPharma Inc in Canada) for immunogenicity testing. These will be anonymised samples. Participants who agree to full study participation will be asked if they consent to storage of any leftover samples for use in other ethically approved research for up to 25-year period, which will be optional.

The aims of the study and all tests to be carried out will be explained. The participant will be given the opportunity to ask about details of the trial, and will then have time to consider whether or not to participate. If they do decide to participate, the participant and the investigator will sign and date the relevant screening consent form, and full-study participation consent form (if eligible). However, in the current crisis, there may be occasions when it is necessary for the consent form to be signed by an appropriately trained and delegated research nurse instead of the investigator. The participant would always have the opportunity to discuss the study with a medically qualified investigator if they wish. The participant will then be provided with a copy of the consent forms to take away and keep, with the original being stored in the case report form (CRF).

6.3. Inclusion and exclusion criteria

This trial will be conducted in generally healthy adults without HIV, except for Group-3 (i.e. safety and immunogenicity in people with HIV).

6.3.1. Inclusion Criteria for all participants

The participants must satisfy all the following criteria to be eligible for the trial:

- Healthy adults aged 18-65 years.
- Documented result of not being infected with HIV (including screening by a rapid HIV antibody test) within two weeks of randomization into the study for Group-1 and Group-2 participants only.
- Able and willing (in the Investigator’s opinion) to comply with all study requirements.
- Willing to allow investigators review available medical records, and review all medical and laboratory records if participant is admitted to hospital with respiratory tract infection suspected or confirmed to be COVID-19.
- For females only, willingness to practice continuous effective contraception (see below) during the study and a negative pregnancy test on the day(s) of screening (within 14 days of randomization) or vaccination.
- For Group-3 only (i.e. HIV-infected), need to have been on anti-retroviral treatment for at least three months and HIV-1 viral load is <1,000 copies/ml within two weeks of randomization.

- Agreement to refrain from blood donation during the course of the study.
- Provide written informed consent.

6.3.2. Exclusion Criteria

The participant may not enter the study if any of the following apply:

- Planned receipt of any vaccine other (licensed or investigational) than the study intervention within 30 days before and after each study vaccination.
- Use of any unproven registered and unregistered treatments for COVID-19.
- Evidence of current SARS-CoV-2 infection detected by molecular assay detection of SARS-CoV-2 done within 96 hours prior to randomization.
- Acute respiratory and/or non-respiratory illness consistent with potential COVID-19 (see Table 4 for list of symptoms) concurrent or within 14 days prior to first study vaccination (medical history and/or physical examination) or documented temperature of > 38°C during this period. NOTE: This is a temporary exclusion for which the subject may be re-evaluated if they remain free from acute respiratory and/or non-respiratory illness consistent with potential COVID-19 after 14 days. Should a subject have a SARS-CoV-2 positive test, they may NOT be randomized.
- Prior receipt of an investigational or licensed vaccine likely to impact on interpretation of the trial data (e.g. Adenovirus vectored vaccines, any coronavirus vaccines).
- Administration of immunoglobulins and/or any blood products within the three months preceding the planned administration of the vaccine candidate.
- HbSAg positivity on the screening sample, or any sample obtained within three months of randomization.
- Grade 2 or higher level of abnormality for FBC, U&E or LFT based on DAIDS Grading Criteria (Version 2.1, July 2017)
- History of allergic disease or reactions likely to be exacerbated by any component of the ChAdOx1 nCoV-19 vaccine.
- Any history of hereditary angioedema or idiopathic angioedema.
- Any history of anaphylaxis in relation to vaccination.
- Pregnancy, lactation or willingness/intention to become pregnant during the study.
- History of cancer (except basal cell carcinoma of the skin and cervical carcinoma in situ).
- History of serious psychiatric condition likely to affect participation in the study.
- Bleeding disorder (e.g. factor deficiency, coagulopathy or platelet disorder), or prior history of

significant bleeding or bruising following IM injections or venipuncture.

- Any other serious chronic illness requiring hospital specialist supervision.
- Chronic respiratory diseases, including poorly controlled/ unstable asthma
- Chronic disease inclusive of:

  a) hypertension if ≥Grade 2 based on DAIDS AE Grading Version 2.1-July 2017

  b) congestive heart failure;

  c) chronic obstructive pulmonary disease by Global Initiative for Chronic Obstructive Lung Disease (GOLD) classification of ≥ 2;

  d) evidence of coronary artery disease as manifested by cardiac interventions or cardiac medications for control of symptoms;

  e) chronic type 2 diabetes (adult onset) requiring insulin;

  f) chronic kidney disease/renal insufficiency;

  g) chronic gastrointestinal and hepatic diseases; or

  h) chronic neurological diseases.

- Seriously overweight (BMI ≥ 40 Kg/m^2)
- Suspected or known current alcohol abuse as defined by an alcohol intake of greater than 42 units every week (% alcohol x volume (ml)/1000= number of units; e.g. Normal beer= 2 units, Glass of wine =3 units).
- Suspected or known injecting drug abuse in the 5 years preceding enrolment.
- Any clinically significant abnormal finding on screening urinalysis.
- Any other significant disease, disorder or finding which may significantly increase the risk to the participant because of participation in the study, affect the ability of the participant to participate in the study or impair interpretation of the study data.
- History of laboratory confirmed COVID-19 illness or known close contact with a person that was infected with SARS-COV-2. Close contact refers to being in contact with someone in the same household, or for at least 15 minutes and in close proximity with an infected person in the absence of wearing of a face masks.
- New onset of fever or a cough or shortness of breath in the 30 days preceding screening and/or enrolment

- In addition to above, Group 1 & 2 participants need to fulfil the following exclusion criteria: Any confirmed or suspected immunosuppressive or immunodeficient state, including HIV infection; asplenia; recurrent severe infections and chronic use (more than 14 days) immunosuppressant medication within the past 6 months (topical steroids are allowed).

- Any confirmed or suspected immunosuppressive or immunodeficient state (except HIV infection for Group-3), asplenia, recurrent severe infections and chronic use (more than 14 days) immunosuppressant medication within the past 6 months (topical steroids are allowed).

Note: Stable endocrine disorders that have a confirmed autoimmune etiology (eg, thyroid, pancreatic), including stable diabetes not requiring insulin are allowed.
Should participants develop COVID-19 prior to the second dose of vaccine, or test positive for SARS-CoV-2 infection and be asymptomatic, the participants will remain eligible to receive a second dose of assigned study-intervention. The second dose of assigned study-drug will however be delayed for at least: i. 14 days in individuals that had asymptomatic SARS-CoV-2 infection, ii. 14 days after symptom resolution if mild illness; iii. 28 days after illness onset following moderate or severe illness, and is clinically stable based on the discretion of the investigator; iv. For cases requiring hospitalization for COVID-19, the 2nd dose should be delayed for at least 14 days post-discharge and participant needs to be clinically stable.

6.3.3. Re-vaccination exclusion criteria

The following AEs associated with any vaccine, or identified on or before the day of vaccination constitute absolute contraindications to further administration of an IP to the participant in question. If any of these events occur during the study, the participant will continue follow-up in the study but will not receive any further study investigational vaccine:

- o Anaphylactic reaction following administration of vaccine
- o Pregnancy

6.3.4. Effective contraception for female participants

Female participants of childbearing potential (any woman or adolescent who has begun menstruation) are required to use an effective form of contraception during the course of the study (i.e. until their last

follow-up visit).

Acceptable forms of contraception for female participants include:

- Established use of oral, injected or implanted hormonal methods of contraception.
- Placement of an intrauterine device (IUD) or intrauterine system (IUS).
- Total abdominal hysterectomy.
- Bilateral tubal Occlusion
- Barrier methods of contraception (condom or occlusive cap with spermicide).
- Post-menopausal women, defined as a woman over the age of 45 who has not had a menstrual period for at least 12 months.

Female participants will be requested to continue obtaining their contraceptives from their nearest clinic, which is provided at no-cost in the public-sector. Should this not be feasible, the study will provide female participants with the contraceptives.

Female participants in a same-sex relationship and women that are post-menopausal will not be required to be on contraception.

6.3.5. Prevention of 'Over Participating'

Participants will be excluded from the study if they are concurrently involved in another trial where an IP has been administered within 30 days prior to enrolment, or will be administered during the trial period. They will not be enrolled they are actively registered on another investigational vaccine or medication trial.

6.3.6. Withdrawal of Participants

In accordance with the principles of the current revision of the Declaration of Helsinki and any other applicable regulations, a participant has the right to withdraw from the study at any time and for any reason, and is not obliged to give his or her reasons for doing so. The Investigator may withdraw the participant at any time in the interests of the participant's health and well-being. In addition, the participant may withdraw/be withdrawn for any of the following reasons:

- Administrative decision by the Investigator.
- Ineligibility (either arising during the study or retrospectively, having been overlooked at screening).
- Significant protocol deviation.
- Participant non-compliance with study requirements.

- An AE, which requires discontinuation of the study involvement or results in inability to continue to comply with study procedures.

The reason for withdrawal will be recorded in the CRF. If withdrawal is due to an AE, appropriate follow-up visits or medical care will be arranged, with the agreement of the participant, until the AE has resolved, stabilized or a non-trial related causality has been assigned. The DSMC or DSMC chair may recommend withdrawal of participants.

Any participant who is withdrawn from the study may be replaced, if that is possible within the specified time frame.

If a participant withdraws from the study, data and blood samples collected before their withdrawal will still be used on the analysis. Storage of blood samples will continue unless the participant specifically requests otherwise.

In all cases of participant withdrawal, long-term safety data collection, including some procedures such as safety bloods, will continue as appropriate if participants have received one or more vaccine doses, unless they decline any further follow-up.

6.4. Pregnancy

Should a participant become pregnant during the trial, no further study IP will be administered. She will be followed up for clinical safety assessment with her ongoing consent and in addition will be followed until pregnancy outcome is determined. We would not routinely perform venipuncture in a pregnant participant unless there is clinical need. Women falling pregnant during the study will also continue in follow-up for COVID-19 and be retained in the efficacy analyses. A 'Pregnancy reporting' form must be completed within 7 days of site staff becoming aware of the pregnancy, and a pregnancy outcome form must be completed within 7 days of delivery, or as soon as possible (within 7 days of site awareness) if site is notified more than 7 days post-delivery.

7. TRIAL PROCEDURES

This section describes the trial procedures for evaluating study participants and follow-up after administration of study vaccine.

7.1. Schedule of Attendance

All participants in groups 1 and 3 will have the same schedule of clinic attendances and procedures as indicated in the schedules of attendance (Table 8). Participants will receive either the ChAdOx1 nCoV-19

vaccine or NaCl (0.9%) placebo injection, and undergo follow-up for a total of 12 months' post enrolment. The total volume of blood donated during the study will be 160-315mL depending on which group they are allocated to. Additional visits or procedures may be performed at the discretion of the investigators, e.g., further medical history and physical examination, urine microscopy in the event of positive urinalysis or additional blood tests if clinically relevant.

7.2. Observations

Pulse, respiratory rate, oxygen saturation, blood pressure and temperature will be measured at the time-points indicated in the schedule of procedures and may also be measured as part of a physical examination if indicated at other time-points.

7.3. Blood tests, Nasal swab/saliva and urinalysis

Blood will be drawn for the following laboratory tests and processed at an accredited Laboratory for:

- **Haematology;** Full Blood Count and differential count (Groups 1 and 3)
- **Biochemistry; U&E (**Sodium, Potassium, Urea, Creatinine), Liver Function Tests (Albumin, ALT, ALP, Bilirubin) (Groups 1 and 3)
- **Diagnostic serology;** HBsAg, HIV antibodies (specific consent will be gained prior to testing blood for these blood-borne viruses). HIV Elisa will be repeated on HIV-negative participants at trial conclusion visit (day 364). HbA1C will be done on all participants, and those with an abnormal result will be referred for further medical care, but remain eligible for study enrolment. (All Groups)
- **Genetics;** Human Leukocyte Antigen (HLA) typing (All Groups)

COVID-19 PCR processing (nasal swab and/or saliva)

A nasal swab and/or saliva will be collected for testing of SARS-COV-2. Sample processing will be done at the RMPRU using molecular detection methods, with confirmatory testing at another accredited laboratory. In the case of discordant results between RMPRU and the second laboratory, a further aliquot of the sample will be submitted to a third accredited laboratory for testing, and the result from the third laboratory will be assumed to be the final result.

Additional safety blood tests may be performed if clinically relevant at the discretion of the medically qualified investigators, including potential prognostic indicators or markers of severe COVID-19 disease.

Urinalysis; Urine will be tested for protein, blood and glucose at screening. For female participants only, urine will be tested for beta-human chorionic gonadotrophin (β-HCG) at screening and immediately prior to vaccination.

Immunology; Immunogenicity will be assessed by a variety of immunological assays.

Serum IgG and IgM: The serum samples will be analysed by ELISA or on other appropriate immuno-assays platforms such as Luminex) for titres of IgG and IgM to two different versions of the spike protein (full length spike protein and receptor binding domain (RBD). These proteins are the major targets for neutralizing antibodies for SARS-CoV-2. Plasmids for these proteins were procured from the laboratory of Prof Florian Krammer, Mount Sinai USA and proteins were successfully expressed and purified in the laboratory of Prof Penny Moore at NICD, South Africa. Fluorescence based micro-bead immunosorbent assay for IgG against SARS-CoV-2 whole length spike protein and the RBD domain on luminex platform has been developed at RMPRU and assay harmonization will be done in collaboration with Prof. Andrew Pollard lab, Oxford University, United Kingdom.

This will be a two-step analysis in which first step includes screening of serum samples against the RBD and second step in which positive samples from the first step undergo a confirmatory testing against the full length spike protein. COVID-19 IgG assay against RBD and Spike protein has been set up at RMPRU and checked for sensitivity (compared to PCR positivity) and specificity (using serum samples from pre COVID months, Sep, Oct, Nov, Dec 2019). Covid 19 IgM assay set up underway. In house reference serum for both IgG and IgM will be developed by pooling convalescent serum from COVID-19 positive participants, and will be calibrated against standard reference reagent from National Institute for Biological Standards and Control (NIBSC), which is providing references sera to laboratories as part of a WHO COVID-19 serology working groups. COVID-19 assay will be further harmonized using NIBSC serum panel which includes high, medium and negative control serum panel. Each luminex run will include in-house high, medium and negative serums for quality control. The assay will further expand to IgA for breast milk analysis.

In addition, samples may be sent to the Oxford collaborators group, and possibly another reference group for further testing.

Ex vIvo- Elispot Assay:

The *ex vivo* IFN-gamma ELISpot assay, will be used to quantify the frequency of antigen-specific effector T cells in response to vaccination. The assay will be performed on standardized procedure from Oxford University. Pools of peptides needed for the assay will be supplied from Oxford University. For Elispot assay, peptides are designed to cover the length of the SARS-CoV2 spike construct and comprise 15mer peptides overlapping by 10 aa, giving a total of 258 peptides.

<u>Cytokine analysis</u>: Serum samples will be analysed at RMPRU for a panel of 25 cytokines which includes

TH1 cytokines (IFN-γ and interleukin (IL)-2), TH2 Cytokines (IL-4, IL-5, IL-6, IL-10, and IL-13) and other pro-inflammatory markers such as TNF-α on multiplex bead-based immunoassay using commercial kits as per manufacturer instructions (Novex, Human Cytokine Magnetic 25-Plex PanelCatalog #: LHC0009M).

Neutralisation measurements will use an assay adapted from well-validated existing HIV-based pseudovirus neutralization assays using the pNL4–3.luc.R-E HIV construct with a SARS-CoV-2 spike protein. This assay is being developed and validated in collaboration with Dr David Montefiori, Duke University.

Pseudotyped neutralization assay:
The SARS-CoV-2 neutralization assay (optimized in collaboration with Dr Nicole-Doria-Rose, VRC) is an adaptation of a highly validated HIV neutralization assay routinely in use at the NICD. The SARS-CoV-2 assay measures neutralization of pseudotyped virus in ACE2-over-expressing 293T target cells (developed by Dr Michael Farzan, The Scripps Research Institute) as a function of a reduction in Luc reporter gene expression. The pseudotyped virus consists of lentiviral particles that are deficient for lentiviral env, but have surface SARS-CoV-2 spike protein and package the gene for firefly luciferase. Infected cells express luciferase, and luciferase activity is quantified by relative light units (RLU) of luminescence. Virus is applied to cells with or without pre-incubation with antibodies; neutralizing antibodies reduce infection, resulting in lower RLUs. Serial dilution of antibodies can be used to produce a dose-response curve to quantify potency. The assay is performed in 96-well flat-bottom black culture plates for high throughput capacity and enhanced luciferase signal Use of a clonal cell line provides enhanced precision and uniformity. Controls will be neutralizing monoclonal antibodies expressed in-house as well as COVID-19 HIV positive and negative serum samples.

Live virus neutralization assays:
This assay, being developed at the NICD by Prof Janusz Paweska utilizes live SARS-CoV-2 coronavirus cultured for one week in Vero cells. After visualization of microscopic cytopathic effects by microscopy, cultures are confirmed positive by PCR and viral stocks cryopreserved. Microneutralization assays will be performed by incubation of SARS-CoV-2 virus with Vero cells with or without pre-incubation with antibodies. Neutralizing antibodies reduce infection, resulting in reduced cytopathic effect. Cross-validation of the live and pseudotyped neutralization assays will be performed using shared SARS-CoV-2 convalescent sera and neutralizing monoclonal antibodies expressed in-house.

Fc effector functionality, including antibody dependent cellular cytotoxicity, complement deposition, and phagocytosis will assess responses to spike trimer or the receptor binding domain. ADCC will use spike trimer or receptor binding domain (RBD)-coated Huh7 cells that express the SARS-CoV-2 receptor ACE-2. Targets for ADCD and ADCP will be neutravidin fluorescent beads coated with spike or RBD proteins.

Targets will be incubated with SARS-CoV-2 convalescent sera. Effector cells for ADCC will be PBMCS from uninfected donors, and will measure granzyme B. ADCD will be measured as the amount of C3b deposition on the surface of antigen-coated beads. ADCP will be measured as the percentage of antigen-coated beads taken up by THP-1 cells in an antibody dependent manner. In addition, samples may be sent to the Oxford collaborators group, and possibly another reference group for further testing. A detailed update of the specific immunology assays to be used in this study will be provided prior to enrolling the first subject in the study.

Other exploratory immunological assays including antibody subtype assays, DNA analysis of genetic polymorphisms potentially relevant to vaccine immunogenicity, monoclonal antibody isolation and gene expression studies amongst others may be performed at the discretion of the Investigators.

Collaboration with other specialist laboratories in South Africa, the UK, Europe, Canada and elsewhere for further exploratory tests may occur. This would involve the transfer of serum or plasma and, PBMC and/or other study samples to these laboratories, but these would remain anonymised. Informed consent for this will be gained from participants. Samples collected for the purposes of COVID-19 diagnosis will be sent to reference laboratories in South Africa for confirmatory testing.

All participants testing positive for SARS-COV2 will be notified through the Notifiable Medical Conditions, per regulatory requirements in South Africa, and includes providing personal data to implement isolation measures of infected individuals and tracing of their contacts. Participants will be informed the obligation on the part of the investigators to submit this level of information to the Notifiable Medical Conditions registry, and local authorities that are responsible for monitoring of infected cases and their contacts.

Participants will be informed that there may be leftover samples of their blood (after testing for this study is completed), and that such samples may be stored up to 25 years for possible future research (exploratory immunology), including genotypic testing of genetic polymorphisms potentially relevant to vaccine immunogenicity. Participants will be able to decide if they will permit such future use of any leftover samples. With the participants' informed consent, any leftover cells, urine and serum/plasma will be frozen for future analysis of COVID-19 and other coronaviruses related diseases or vaccine-related responses. If a participant elect not to permit this, all of that participant's leftover samples will be discarded after the required period of storage to meet Good Clinical Practice (GCP) and regulatory requirements.

Samples that are to be stored for future research will be stored at RMPRU.

7.4. Study visits

The study visits and procedures will be undertaken by one of the clinical trials team. The procedures to be included in each visit are documented in the schedule of attendances. Each visit is assigned a time-point and a window period, within which the visit will be conducted.

7.4.1. Screening visit

Participants will be required to share past medical and past surgical history, and medication at screening visit as an initial confirmation of eligibility. All potential participants will have a screening visit, which may take place up to 14 days prior to vaccination, although some results such as molecular testing for SARS-CoV-2 need to be done within 96 hours of randomization. The screening informed consent will be taken before screening. If consent is obtained, the procedures indicated in the schedule of attendances will be undertaken including a medical history, physical examination, blood tests and height and weight. Individually each participant will have the opportunity to question an appropriately trained and delegated researcher before signing the full-study participation consent at enrollment visit.

Abnormal clinical findings from the urinalysis or blood tests at screening will be assessed by a medically qualified study member. Abnormal clinical and blood tests following screening will be assessed according to specific laboratory adverse event grading tables (DAIDS Laboratory Grading of Abnormal Results; Version 2.1, July 2017). Any abnormal test result deemed clinically significant may be repeated to ensure it is not a single occurrence. If an abnormal finding is deemed to be clinically significant (Grade 2 or higher abnormality), the participant will be informed and appropriate medical care arranged with the permission of the participant.

The eligibility of the participant will be reviewed at the end of the screening visit and again when all results from the screening visit have been considered. Decisions to exclude the participant from enrolling in the trial or to withdraw a participant from the trial will be based on fulfilling the inclusion and exclusion criteria, as well as at the discretion of the Investigator. If eligible, a day 0 visit will be scheduled for the participant to receive the vaccine and subsequent follow-up. Participants will be consented for full study participation prior to randomization at the day-0 visit.

If more than 14 days elapse between screening and an eligible and willing participant presents for enrolment, re-screening will be required. Informed consent should be verified and discussion recorded in source document. All applicable screening procedures, except for HIV if negative in the past three months should be repeated at re-screening visit. Also, if applicable, safety bloods should be repeated, to ensure that they are done ≤14 days prior to vaccination, and SARS-CoV-2 swab needs to be done in

96 hours prior to randomisation.

7.4.2. Day 0: Enrolment and vaccination visit

Participants will be considered enrolled into the trial at the point of written, signed consent for full-study participation, i.e. following confirmation of eligibly through the screening visit. The initiation of the consenting process for full-study participation may precede the date on which the consent form is signed, to allow adequate time for potential participants to consider their willingness to participate. Before randomization, the eligibility of the participant will be reviewed. Pulse, respiratory rate, oxygen saturation, blood pressure and temperature will be observed and if necessary, a medical history and physical examination may be undertaken. Vaccinations will be administered as described below.

7.4.3. Vaccination

All vaccines will be administered intramuscularly according to specific SOPs. The injection site will be covered with a sterile dressing and the participant will stay in the trial site for observation, in case of immediate adverse events. Observations will be taken 60 minutes after vaccination (+/- 30 minutes). Post-vaccination observations include pulse rate, respiratory rate, oxygen saturation, blood pressure, temperature and vaccination site review.

In all groups, participants will be given an oral thermometer, measurement device and diary card (paper or electronic), with instructions on use, along with the emergency 24-hour telephone number to contact the on-call study doctor if needed. Participants will be instructed on how to self-assess the severity of these AEs. There will also be space on the diary card to self-document unsolicited AEs, and whether medication was taken to relieve the symptoms. Diary cards will collect information on the timing and severity of the following solicited AEs:

Table 8: Solicited AEs as collected on post vaccination diary cards

Local solicited AEs	Systemic solicited AEs
Pain	Fever
Tenderness	Feverishness
Redness	Chills
Warmth	Joint pains
Itch	Muscle pains
Swelling	Fatigue
Induration	Headache
	Malaise
	Nausea

7.4.3.1. Sequence of Enrolment and Vaccination of Participants

Prior to initiation of the study, any newly available safety data will be reviewed from animal studies (including non-human primate studies being conducted in UK) and clinical trials of coronavirus vaccines (including data from first 50 participants being enrolled in similar trial in UK, COV001) being tested elsewhere, and discussed with the DSMC and/or regulatory and ethics committees as necessary. Participants in group 1 (HIV-uninfected adults, prime-boost 2-dose, intensive follow up) and Group-3 may be enrolled concurrently, contingent upon approval by the DSMC.

Based on the immunogenicity data from the initial safety/immunogenicity cohort enrolled in the UK study (COV001);[1] and following review by the DSMC it was decided that all participants will receive two doses of the assigned study-intervention. A notification on the dosing schedule for Group-2 was submitted the Ethics committee and SAPHRA.

7.4.4. Subsequent visits:

Follow-up visits will take place as per the schedule of attendances described in Table 8, Table 9 and Table 10 with their respective windows. Participants will be assessed for local and systemic adverse events, interim history, physical examination, review of diary cards (paper or electronic) and blood tests at these time points as detailed in the schedule of attendances. Blood will also be taken for immunology purposes.

If participants experience adverse events (laboratory or clinical), which the investigator (physician), CI and/or DSMC chair determine necessary for further close observation, the participant may be admitted to a hospital for observation and further medical management under the care of the attending-physicians.

7.4.5. Participants under quarantine

Given the evolving epidemiological situation both globally and in South Africa, should a participant be under isolation or quarantine and unable to attend any of the scheduled visits, a telephone consultation will be arranged in order to obtain core study data where possible. Any study samples from participants under quarantine or isolation will be collected at the place of residence at the time of the participant (or in hospital if hospitalized), by trained study staff with appropriate precautionary measures being implemented (including use of protective personal equipment).

CONFIDENTIAL

Table 8: Schedule of visits: Groups 1 & 3

Visit number	Screening	V1	V2	V3	V4	V5	V6*	V7*	V8*	V9*	V10	V11	COVID-19
Day #	-14 to -1	0 (Vax1)	3	7	14	28 (Vax2)	31	35	42	56	182	364	Illness
	Screening	D0	V1+ 3 days ±1; (day 2-4)	V1 +7 days ±2 (day 5-9)	V1+ 14 days ±3 (day 11-17)	Visit 1 + 28 days ±7 day 21-35)	V5+3 days ±1	V5+7 days ±2	V5+14 days ±3	V5 +28 (±7)	D182 (±14)	D364 (±14 days)	As required $
Eligibility	X	X											
Consenting	X§	X¥											
Inclusion/ exclusion	X	X				X							
Contraindications	X	X				X							
Vital signs #	X	X	X	X	X	X	X	X	X	X	X	X	X
Medical history	X												X
Physical examination	X (full)	X	X	X	X	X (full)	X	X	X	X	X	X	X (full)
Vaccination		X				X							
Post vaccination obs		X	X (deltoid)	X (deltoid)		X	X (deltoid)	X (deltoid)					
Diary cards provided		X				X							X (illness DC)
DC collected				X				X					
Safety bloods (FBC, U&E, LFT)	X		X	X		X		X		X			
Screening bloods (HBsAg, , HIV, HbA1C)	X											X (HIV Gr 1)	
HIV Viral load and CD4 (Grp 3 only)	VL and CD4												
Immunology bloods***		E, PAX (12.5-17.5ml)	Cyt, PAX (12.5 - 17.5ml)		E & CMI (20-25ml)	E, N, PAX (17.5-22.5ml)		Cyt (10-15mls)	E & N & CMI (25-30ml)	E (10-15ml)	E (10-15ml)	E & N (15-20ml)	E (10-20ml)
Urinalysis	X												

Urinalysis bHCG (women only)	X	(X)				X							
Nasal swab/ saliva	X (V1-96 hours)	X		X	X	X		X	X	X	X	X	X

* Visit 5 to Visit 9 are scheduled relative to when the 2nd dose of vaccine/placebo (Visit 4) has been administered.

§ Screening informed consent form (ICF).

¥Full study participation informed consent form, if remain eligible after completion of screening procedures.

Vital signs includes pulse, respiratory rate, oxygen saturation, blood pressure and temperature;

** Timeline is approximate only. Exact timings of visits relate to the day on enrolment, i.e., each visit must occur at indicated number of days after enrolment ± time window

***Abbreviations for laboratory tests: E =Elisa; Cyt= Th1 and Th2 cytokine profile; N= neutralization and/or pseudo-neutralisation assay; CMI= cell-mediated immunity assay, PAX= PAXgenes.

Blood test summary:

- Screening: Safety bloods (Full Blood Count, FBC; Urea and Electrolytes, U&E; Liver Function tests, LFT); Screening bloods (HBsAg, HIV, Glycosylated hemoglobin; HbA1c), In group 3 only- CD4+ -lymphocyte count, CD4+ & VHIV-1 viral load, VL)
- Visit 1: Immunogenicity- Elisa
- Visit 2 Safety bloods (FBC, U&E, LFT), Immunogenicity- Th1 and Th2 cytokine profile
- Visit 3 Safety bloods (FBC, U&E, LFT)
- Visit 4 Immunogenicity- Elisa & cell-mediated immunity
- Visit 5 Safety bloods (FBC, U&E, LFT), Immunogenicity- Elisa & neutralization and/or pseudo-neutralisation assay
- Visit 6 NIL
- Visit 7 Safety bloods (FBC, U&E, LFT), Immunogenicity- Th1 and Th2 cytokine profile
- Visit 8 Immunogenicity- Elisa, neutralization and/or pseudo-neutralisation assay & cell-mediated immunity
- Visit 9 Safety bloods (FBC, U&E, LFT), Immunogenicity- Elisa
- Visit 10 Immunogenicity- Elisa
- Visit 11 Immunogenicity- Elisa & neutralization and/or pseudo-neutralisation assays
- Illness visit Immunogenicity- Elisa

$ Nasal swabs/ saliva and Elisa (illness) will be repeated at Days 5-8, 12-15 and 28-35 days.

Table 9: Visit schedule for group 2a

Visit number	Screening	V1	V2	V3	V4	V5*	V6*	V7*	V8	V9	COVID-19
Day #	-14 to -1	0 (Vax1)	7	14	28 (Vax2)	35	42	56	182	364	Illness
	Screening	D0	V1 +7 days ±2 (day 5-9)	V1+ 14 days ±3 (day 11-17)	Visit 1 + 28 days ±7	V4+7 days ±2	V4+14 days ±3	V4 +28 (±7)	D182 (±14)	D364 (±14 days)	As required $
Eligibility	X	X									
Consenting	X§	X¥									
Inclusion/ exclusion	X	X			X						
Contraindications	X	X			X						
Vital signs #	X	X	X	X	X	X	X	X	X	X	X
Medical history	X										X
Physical examination	X (full)	X	X	X	X (full)	X	X	X	X	X	X (full)
Vaccination		X			X						
Post vaccination obs		X	X (deltoid)		X	X (deltoid)					
Diary cards provided		X			X						X (illness DC)
DC collected			X			X					
Screening bloods (HBsAg, , HIV, HBA1C)	X									X (HIV)	
Immunology bloods***		E, PAX (12.5-17.5ml)	Cyt, PAX (12.5 -17.5ml)	E & CMI (20-25ml)	E, N, PAX (17.5-22.5ml)		E & N & CMI (25-30ml)	E (10-15ml)	E (10-15ml)	E & N (15-20ml)	E (10-20ml)
Urinalysis	X										
Urinalysis bHCG (women only)	X	(X)			X						
Nasal swab/ saliva	X (V1-96 hours)	X	X	X	X	X	X	X	X	X	X

- Visit 5 to Visit 7 are scheduled relative to when the 2nd dose of vaccine/placebo (Visit 4) has been administered.

§ Screening informed consent form (ICF).
¥Full study participation informed consent form, if remain eligible after completion of screening procedures.

Vital signs includes pulse, blood pressure and temperature;

** Timeline is approximate only. Exact timings of visits relate to the day on enrolment, i.e., each visit must occur at indicated number of days after enrolment ± time window

***Abbreviations for laboratory tests: E=Elisa; Cyt=Th1 and Th2 cytokine profile; N=neutralization and/or pseudo-neutralisation assay; CMI= cell-mediated immunity assay, PAX= PAXgenes.

Vital signs includes pulse, respiratory rate, oxygen saturation, blood pressure and temperature;

** Timeline is approximate only. Exact timings of visits relate to the day on enrolment, i.e., each visit must occur at indicated number of days after enrolment ± time window

***Abbreviations for laboratory tests: E =Elisa; Cyt= Th1 and Th2 cytokine profile; N= neutralization and/or pseudo-neutralisation assay; CMI= cell-mediated immunity assay, PAX= PAXgenes.

Blood test summary:

- Screening: Safety bloods (Full Blood Count, FBC; Urea and Electrolytes, U&E; Liver Function tests, LFT); Screening bloods (HBsAg, HIV, Glycosylated hemoglobin; HbA1c), In group 3 only- CD4+ -lymphocyte count, CD4+ & VHIV-1 viral load, VL)
- Visit 1: Immunogenicity- Elisa
- Visit 2 Safety bloods (FBC, U&E, LFT), Immunogenicity- Th1 and Th2 cytokine profile
- Visit 3 Safety bloods (FBC, U&E, LFT)
- Visit 4 Immunogenicity- Elisa & cell-mediated immunity
- Visit 5 Safety bloods (FBC, U&E, LFT), Immunogenicity- Elisa & neutralization and/or pseudo-neutralisation assay
- Visit 6 NIL
- Visit 7 Safety bloods (FBC, U&E, LFT), Immunogenicity- Th1 and Th2 cytokine profile
- Visit 8 Immunogenicity- Elisa, neutralization and/or pseudo-neutralisation assay & cell-mediated immunity
- Visit 9 Safety bloods (FBC, U&E, LFT), Immunogenicity- Elisa
- Visit 10 Immunogenicity- Elisa
- Visit 11 Immunogenicity- Elisa & neutralization and/or pseudo-neutralisation assays
- Illness visit Immunogenicity- Elisa

$ Nasal swabs/ saliva and Elisa (illness) will be repeated at Days 5-8, 12-15 and 28-35 days.

Table 10: Visit schedule, group 2b (extended efficacy cohort; remaining 1650 participants)

Visit number	Screening	V1	V2	V3	V4	V5	V6	COVID-19
Day #	-14 to -1	0 (Vax1)	28 (Vax2)	42	56	182	364	Illness
	Screening	D0	Visit 1 + 28 days ±7	V2+14 days ±3	V2 +28 (±7)	D182 (±14)	D364 (±14 days)	As required $
Eligibility	X	X						
Consenting	X§	X¥						
Inclusion/ exclusion	X	X	X					
Contraindications	X	X	X					
Vital signs #	X	X	X	X	X	X	X	X
Medical history	X							X
Physical examination	X (full)	X	X (full)	X	X	X	X	X (full)
Vaccination		X	X					
Post vaccination observation		X	X					
Diary cards provided		X	X					X (illness DC)
DC collected			X					
Screening bloods (HBsAg, HIV, HbBA1C)	X						X (HIV)	
Immunology bloods***		E (10-15ml)	E, N, HLA (17.5-22.5ml)	E & N (15-20ml)	E (10-15ml)	E (10-15ml)	E & N (15-20ml)	E (10-20ml)
Urinalysis	X							
Urinalysis bHCG (women only)	X	(X)	X					
Nasal swab/ saliva	X (V1-96 hours)	X	X	X	X	X	X	X

§ Screening informed consent form (ICF).
¥Full study participation informed consent form, if remain eligible after completion of screening procedures.

* IF participants receive two doses of vaccine, then dose 2 will be administered at Visit 2, and follow up visits will be completed 14 days post dose 2 (6- visit schedule). IF participants only receive ONE dose of IP, then no vaccine will be administered at visit 2, and day 42 visit will not be included in visit schedule (5-visit schedule)
# Vital signs includes pulse, respiratory rate, oxygen saturation, blood pressure and temperature;

** Timeline is approximate only. Exact timings of visits relate to the day on enrolment, i.e., each visit must occur at indicated number of days after enrolment ± time window

***Abbreviations for laboratory tests: E=Elisa; Cyt=Th1 and Th2 cytokine profile; N=neutralization and/or pseudo-neutralisation assay; CMI=cell-mediated immunity assay.

Blood test summary:

- Screening: Screening bloods (HBsAg, HIV, HBA1C)
- Visit 1: Immunogenicity- Elisa
- Visit 2 Immunogenicity- Elisa & neutralization and/or pseudo-neutralisation assay and HLA
- Visit 3 Immunogenicity- Elisa, neutralization and/or pseudo-neutralisation assay
- Visit 4 Immunogenicity- Elisa
- Visit 5 Immunogenicity- Elisa
- Visit 6 Immunogenicity- Elisa & neutralization and/or pseudo-neutralisation assay
- Illness visit Immunogenicity- Elisa

$ Nasal swabs/ saliva and Elisa (illness) will be repeated at Days 5-8, 12-15 and 28-35 days

7.4.6. Symptomatic participants

Participants who become symptomatic during follow-up will be instructed to call the study team who will then advise on how to proceed with clinical testing for SARS-COV-2 infection if necessary, as per the trial working instructions. Participants will get weekly reminders (text messages) to get in touch with the study team if they manifesting any of the symptoms indicated in Table 4; or if they are admitted to hospital for any reason. At the COVID-19 testing visit, a nasal swab and/or saliva, blood samples for safety (FBC, Biochemistry, CRP, others if deemed clinically relevant) and immunology, vital signs and other clinical data will be taken. Symptomatic participants may be regularly reviewed over the phone, or in-person if required. Participants will be asked to attend a follow-up visit or have a telephonic call 5 days (±2 days) post SARS-CoV-2 testing for clinical review and further testing if applicable (i.e. worsening or non-resolution of clinical symptoms) if the initial test result was negative for SARS-CoV-2. For participants that initially tested negative and who test positive for SARS-CoV-2 on a repeat swab, the participant will be followed-up as detailed below.
For participants that are confirmed to be infected with SARS-COV-2, repeat nasal swab or saliva sampling (preferably self-administered) and blood samples (for immunology assays) will be obtained at Days 5-8, 12-15 and 28-35 days.

All participants investigated for SARS-CoV-2 on an ambulatory basis, will be required to complete a diary card reporting on daily signs and symptoms for at least seven days from day on which sampled, and recording of the resolution date of the signs and symptoms if the illness duration exceeds 7 days. For hospitalized participants, clinical information will be sourced from the participant or medical records, through to hospital discharge and/or resolution of the illness. Any documented molecular test result confirming SARS-CoV-2 infection of study participants done as part of standard of care will be used as confirmatory evidence of confirmed COVID-19 illness.

7.4.7. Medical notes review

With the participant's consent, the study team will request access to medical notes or submit a data collection form for completion by attending clinical staff on any medically attended COVID-19 episodes. Any data which are relevant to ascertainment of efficacy endpoints and disease enhancement (AESI) will be collected. These are likely to include,

but not limited to, information on ICU admissions, clinical parameters such as oxygen saturation, respiratory rates and vital signs, need for oxygen therapy, need for ventilatory support, imaging and blood tests results, amongst others.

7.4.8. Randomisation, blinding and code-breaking

Participants will be randomised to investigational vaccine or placebo (0.9% NaCl) in a 1:1 allocation, using block randomisation. Block sizes of 8 will be used for all groups (4 IP and 4 placebo).

All participants and clinical study staff, except unblinded pharmacist and vaccine dispenser will be blinded to the trial arm that participants have been allocated to, whether investigational vaccine or placebo. The trial staff administering the vaccine will not be blinded. Vaccines will be prepared out of sight of the participant and syringes will be covered with an opaque object/material until ready for administration to ensure blinding.

If the clinical condition of a participant necessitates breaking the code, this will be undertaken according to a trial specific working instruction and group allocation sent to the attending physician, if unblinding is thought to be relevant and likely to change clinical management.

8. INVESTIGATIONAL PRODUCT

8.1. Manufacturing and presentation

8.1.1. Description of ChAdOx1 nCoV-19

ChAdOx1 nCoV-19 vaccine consists of the replication-deficient simian adenovirus vector ChAdOx1, containing the structural surface glycoprotein (Spike protein) antigens of SARS-CoV-2 of 5-7.5x10^{10} vp dose.

8.2. Supply

ChAdOx1 nCoV-19 utilised in the COV001 trial was formulated and vialed at the Clinical Biomanufacturing Facility (CBF), University of Oxford. The vaccine manufacturing, packaging and labelling have been relocated to the following GMP

manufacturing facilities:

Name of Facility	Responsibility
Cobra Biologics Limited<br><br>Stephenson Building<br><br>Newcastle, ST5 5SP, United Kingdom	Manufacture of Drug Substance
Symbiosis Pharmaceutical Services Limited<br><br>Unit 10 Scion House<br><br>Stirling University Innovation Park<br><br>Stirling, Scotland FK9 4NF<br><br>United Kingdom	Drug Substance Lot Release Testing
Advent Societa' A Responsabilita Limitata,<br><br>via Pontina KM 30,600,<br><br>Pomezia (RM), 00040, Italy	Manufacture of Drug Substance<br><br>Drug Substance Lot Release Testing and Stability Testing
Thermofisher Scientific, Fisher BioServices division, Unit 1,<br>Woodside, Dunmow road,<br>Birchanger, Bishop's Soortford, CM23 5RG,<br>United Kingdom	Packaging, Labelling and Distribution

ChAdOx1 nCoV-19 (AZD1222) has been formulated at Cobra Biologics Ltd, vialed at Symbiosis Pharmaceutical Services, and labelled and packaged at Thermo Fisher Scientific (Hertfordshire, United Kingdom). It will be certified by a Qualified Person (QP) at the MedImmune Pharma, BV (Nijmegen, The Netherlands) or MedImmune Ltd (Cambridge, United Kingdom) before release and transfer to the clinical site. Investigational product will be managed and distributed to South African sites by a qualified IP logistics company in South Africa.

8.3. Storage

The vaccines will be stored in a restricted access refrigerator and / or freezer according to the vial batch storage conditions requirement at the clinical site. The vaccine manufactured by Advent is stored at nominal -80oC (+/- 20 oC) in a secure freezer, at the clinical site. The vaccine manufactured by Cobra Biologics Ltd is stored at 2-8°C in a

secure fridge, at the clinical site.

Vaccine Batch	Storage Conditions
Batch K.0008<br><br>Batch K.0011	-80˚C
Batch 20482B	2- 8 ˚C

All movements of the study vaccines will be documented in accordance with existing standard operating procedure (SOP). Vaccine accountability, storage, shipment and handling will be in accordance with relevant SOPs and forms.

8.4. Administration

On vaccination day, ChAdOx1 nCoV-19 will be allowed to thaw to room temperature and will be administered in accordance with trial specific instructions or stored at 2-8˚C for a maximum of 6 hours, where multiple doses are required from a single vial. The vaccine manufactured by Cobra Biologics is a multi-dose vial which is stored at 2-8 ˚C and does not require thawing. If the vaccine is stored outside of 2-8˚C it must be used within 6 hours. The vaccine will be administered intramuscularly into the deltoid of the non-dominant arm (preferably). All volunteers will be observed in the unit for a minimum of 15 minutes (+15 minutes) after vaccination. During administration of the investigational products, Advanced Life Support drugs and resuscitation equipment will be immediately available for the management of anaphylaxis. Vaccination will be performed and the IMPs handled according to the relevant SOPs.

8.5. Rationale for selected dose

The dose to be administered in this trial have been selected on the basis of clinical experience with the ChAdOx1 adenovirus vector expressing different inserts and other similar adenovirus vectored vaccines (e.g. ChAd63).

A first-in-man dose escalation study using the ChAdOx1 vector encoding an influenza antigen (FLU004), safely administered ChAdOx1 NP+M1 at doses ranging from 5 x 10^8 to

5 x 10^{10} vp. Subsequent review of the data identified an optimal dose of 2.5 x 10^{10} vp balancing immunogenicity and reactogenicity. This dose has subsequently been given to hundreds of participants in numerous larger phase 1 studies at the Jenner Institute. ChAdOx1 vectored vaccines have thus far demonstrated to be very well tolerated. The vast majority of AEs have been mild-moderate and there have been no SARs until this date.

Another simian adenovirus vector (ChAd63) has been safely administered at doses up to 2 x 10^{11} vp with an optimal dose of 5 x 10^{10} vp, balancing immunogenicity and reactogenicity.

MERS001 was the first clinical trial of a ChAdOx1 vectored expressing the full-length Spike protein from a separate, but related betacoronavirus. ChAdOx1 MERS has been given to 31 participants to date at doses ranging from $5x10^9$ vp to $5x10^{10}$ vp. Despite higher reactogeniticy observed at the $5x10^{10}$ vp, this dose was safe, with self-limiting AEs and no SARs recorded. The $5x10^{10}$ vp was the most immunogenic, in terms of inducing neutralising antibodies against MERS-CoV using a live virus assay (Folegatti et al. Lancet Infect Dis,2020, in press). Given the immunology findings and safety profile observed with a ChAdOx1 vectored vaccine against MERS-CoV, the $5x10^{10}$ vp dose was chosen for ChAdOx1 nCoV-19.

The trial conducted in the UK is the first in human assessment of the SARS-CoV 2 S antigenic insert.[16] As other batches of ChAdOx1 nCoV-19 become available, including for this ChAdOx1 nCoV-19_ZA_PhI/II trial, a staggered approach will be used for use of the first 5 vaccines of each new batch. Safety of ChAdOx1 nCoV-19 will be monitored in real time and should unacceptable adverse events or safety concerns arise, doses will be decreased via an amendment. As of 19th August 2020, a total of 9981 participants have been enrolled in the COV001/COV002 studies in the UK and 3688 in Cov003 in Brazil.

Several different batches of vaccine have been produced for the clinical trials: at Oxford University in the UK, Advent in Italy and at COBRA in the UK. Dosing of the vaccine has been based on Abs260 (Oxford and COBRA) or qPCR (Advent) depending on the manufacturers release specifications. Emerging data from 6 different assays, provides more information on the dosing and provides insight into consistency across different batches. For batch K.0008, used in South Africa, dosing was based on the qPCR data from Advent to obtain approximately 5 x 10^{10} vp as the preferred dose level. For batch K.0011 from Advent, the dose has been adjusted to an equivalent of 7.5 x 10^{10} vp on the

Advent qPCR to ensure consistency across batches with the extended panel of assays. In future Astra Zeneca will be developing the vaccine and responsible for subsequent batches, and their assays are included in the table below.

An analytical comparability assessment of ChAdOx1 nCoV-19 (AZD1222) manufactured by CBF, Advent and Cobra Biologics was conducted using a comprehensive set of physiochemical and biological release and characterization tests. In order to support the analytical comparability assessment, A260 testing of Advent's process (K.0007, K.0008, K.0009 and K.0011 lots) was performed, where corrections to the absorbance due to excess polysorbate 80 were made to compensate for polysorbate 80 concentrations above the formulation target of 0.1% (w/v). Differences in strength related attributes (ie, virus particle concentration, virus genome concentration, and infectious virus concentration) are noted. These differences in strength is further examined for potential impact on clinical dosing. The target clinical dosage of CBF's product is 5×10^{10} viral particles per dose based on vp/mL concentration determined by UV spectroscopy (A260), whereas that of Advent's product is 5×10^{10} viral genome copies per dose based on vg/mL concentration determined by qPCR. The target clinical dosage of Symbiosis' product is $3.5 – 6.5 \times 10^{10}$ viral particles per dose based on the vp/mL concentration determined by A260, with a 0.5 mL dosing volume. This dosing range is based on a target 5×10^{10} viral particles per dose and a ± 30% range to take into account process and method variabilities. The planned clinical dosage of Symbiosis' product is compared to that of CBF and Advent products, the resulting Symbiosis' product dosage at 0.5 mL for lot 20481A is somewhat lower in total viral particle per dose (20% from the lower range limit), slightly higher in total viral genome copies per dose (12% from the higher range limit), and slightly lower in total infectious particle per dose (8% from the lower range limit). These differences are considered to be comparable to or within the variabilities from the analytical methods used in concentration determination (A260, qPCR, and infectivity) and the dosing volumes during clinical administration. In summary, with a 0.5 mL dosing volume for Symbiosis' product, strength difference from CBF and Advent products is not expected to have significant clinical impact in terms of reactogenicity and immunogenicity/efficacy.

Table Clinical Strengths of AZD1222 Drug Product

Strength Attribute	Process 1		Process 2				Process 3
	02P20-01	02P20-02	K.0007	K.0008	K.0009	K.0011	20481A
**Concentration**							
Virus particle concentration (A_{260}) (vp/mL)	1.49×10^{11}	1.22×10^{11}	3.12×10^{11}	3.16×10^{11}	2.45×10^{11}	1.4×10^{11}	0.8×10^{11}
Virus genome concentration (qPCR) (vg/mL)	1.7×10^{11}	Not tested	1.7×10^{11}	2.1×10^{11}	1.4×10^{11}	1.5×10^{11}	1.3×10^{11}
AZ qPCR (vg/mL)	1.37×10^{11}	Not tested	1.38×10^{11}	1.42×10^{11}	1.12×10^{11}	0.67×10^{11}	0.77×10^{11}
AZ ddPCR (vg/mL)	1.17×10^{11}	Not tested	1.29×10^{11}	1.27×10^{11}	1.01×10^{11}	0.59×10^{11}	0.71×10^{11}
Infectious particle concentration (ifu/mL) [a]	2.6×10^{9}	Not tested	2.9×10^{9}	3.0×10^{9}	2.4×10^{9}	1.3×10^{9}	1.3×10^{9}
AZ Infectivity (ifu/mL)	2.13×10^{9}	Not tested	1.89×10^{9}	2.04×10^{9}	2.06×10^{9}	1.09×10^{9}	1.28×10^{9}
**Target Clinical Dosage**							
Equivalent DP volume per dose (mL)	0.34	0.41	0.294	0.235	0.356	0.5	0.50
Dosing of virus particle (vp/dose)	5.1×10^{10}	5.0×10^{10}	9.2×10^{10}	7.4×10^{10}	8.7×10^{10}	7×10^{10}	4.0×10^{10}
Dosing of viral genome (vg/dose)	5.8×10^{10}	NA	5.0×10^{10}	4.9×10^{10}	5.0×10^{10}	7.5×10^{10}	6.5×10^{10}
AZ qPCR (vg/dose)	4.7×10^{10}	NA	4.1×10^{10}	3.3×10^{10}	4.0×10^{10}	3.35×10^{10}	3.9×10^{10}
AZ ddPCR (vg/dose)	4.0×10^{10}	NA	3.8×10^{10}	3.0×10^{10}	3.6×10^{10}	2.95×10^{10}	3.5×10^{10}
Dosing of infectious particle (ifu/dose)	8.8×10^{8}	NA	8.5×10^{8}	7.1×10^{8}	8.5×10^{8}	6.5×10^{8}	6.5×10^{8}
AZ Infectivity (ifu/dose)	7.2×10^{8}	NA	5.6×10^{8}	4.8×10^{8}	7.3×10^{8}	5.45×10^{8}	6.4×10^{8}

ifu = infectious units; NA = not applicable; vp = virus particle; vg = virus genome

[a] Testing performed using the Advent infectivity assay.

8.6. Minimizing environmental contamination with genetically modified organisms (GMO)

The trial will be performed in accordance with the South African Genetically Modified Organisms Act 15 of 1997 (as amended). Approved SOPs will be followed to minimise dissemination of the recombinant vectored vaccine virus into the environment. GMO waste will be inactivated according to approved SOPs.

8.7. Control Vaccine

Participants who are allocated to the control groups will receive two injections (all Groups) of 0.9% Normal saline (0.9% NaCl) instead of ChAdOx1 nCoV-19.

Participants will be blinded as to which intervention they are receiving. A vaccine accountability log of IP and placebo (NaCl) will be maintained at each trial site.

8.8. Compliance with Trial Treatment

All vaccinations will be administered by the research team and recorded in the CRF. The study medication will be at no time in the possession of the participant and compliance will not, therefore, be an issue.

8.9. Accountability of the Trial Treatment

Accountability of the IP and placebo will be conducted in accordance with the relevant SOPs.

8.10. Concomitant Medication

As set out by the exclusion criteria, participants may not enter the study if they have received: any vaccine in the 30 days prior to enrolment or there is planned receipt of any other vaccine within 30 days of each vaccination, any investigational product within 30 days prior to enrolment or if receipt is planned during the study period, or if there is any chronic use (>14 days) of any immunosuppressant medication (except ARVs in group 3 participants) within 6 months prior to enrolment or if receipt is planned at any time during the study period (inhaled and topical steroids are permitted).

8.11. Provision of Treatment for Controls

If this vaccine is proven to be efficacious following analysis of the primary endpoint and if the DSMC agrees, participants allocated to placebo group may be offered the IP.

9. ASSESSMENT OF SAFETY

Safety will be assessed by the frequency, incidence and nature of AEs and SAEs arising during the study, from the time of randomization (Day 0 visit) onward.

9.1. Definitions

9.1.1. Adverse Event (AE)

An AE is any untoward medical occurrence in a participant, which may occur during or after administration of an IP and does not necessarily have a causal relationship with the intervention. An AE can therefore be any unfavourable and unintended sign (including any clinically significant abnormal laboratory finding or change from baseline), symptom or disease temporally associated with the study intervention, whether or not considered related to the study intervention.

9.1.2. Adverse Reaction (AR)

An AR is any untoward or unintended response to an IP. This means that a causal relationship between the IP and an AE is at least a reasonable possibility, i.e., the relationship cannot be ruled out. All cases judged by the reporting medical Investigator as having a reasonable suspected causal relationship to an IP (i.e. possibly, probably or definitely related to an IP) will qualify as AR.

Adverse events that may be related to the IP are listed in the Investigator's Brochure for each product.

9.1.3. Serious Adverse Event (SAE)

An SAE is an AE that results in any of the following outcomes, whether or not considered related to the study intervention.

- Death
- Life-threatening event (i.e., the participant was, in the view of the Investigator, at immediate risk of death from the event that occurred). This does not include an AE that, if it occurred in a more severe form, might have caused death.
- Persistent or significant disability or incapacity (i.e., substantial disruption of one's ability to carry out normal life functions).
- Hospitalisation or prolongation of existing hospitalisation, regardless of length of stay, even if it is a precautionary measure for continued observation. Hospitalisation (including inpatient or outpatient hospitalisation for an elective procedure) for a pre-existing condition that has not worsened unexpectedly does not constitute a serious AE.
- An important medical event (that may not cause death, be life threatening, or require hospitalisation) that may, based upon appropriate medical judgment, jeopardise the participant and/or require medical or surgical intervention to prevent one of the outcomes listed above. Examples of such medical events

include allergic reaction requiring intensive treatment in an emergency room or clinic, blood dyscrasias, or convulsions that do not result in inpatient hospitalisation.

- Congenital anomaly or birth defect.

9.1.4. Serious Adverse Reaction (SAR)

An AE that is both serious and, in the opinion of the reporting Investigator or Sponsors, believed to be possibly, probably or definitely due to an IP or any other study treatments, based on the information provided.

9.1.5. Suspected Unexpected Serious Adverse Reaction (SUSAR)

A SUSAR, the nature and severity of which is not consistent with the information about the medicinal product in question set out in the IB.

9.2. Expectedness

No IP related SAEs are expected in this study. All SARs will therefore be reported as SUSARs.

9.3. Foreseeable Adverse Reactions:

The foreseeable ARs following vaccination with ChAdOx1 nCoV-19 include injection site pain, tenderness, erythema, warmth, swelling, induration, pruritus, myalgia, arthralgia, headache, fatigue, fever, feverishness, chills, malaise and nausea. Participants will be advised to make immediate contact with the site for any solicited adverse that is Grade 3 or 4 that occurred within 7 days of vaccination, to ensure timeliness of it being reported as an SAE and to determine necessary management.

9.4. Adverse Events of Special Interest

Disease enhancement following vaccination with ChAdOx1 nCoV-19, as defined by international working groups, will be monitored. Severe COVID-19 disease will be defined using clinical criteria. Detailed clinical parameters will be collected from medical records and aligned with agreed definitions as they emerge. These are likely to include, but are not limited to, oxygen saturation, need for oxygen therapy, respiratory rate, need for ventilatory support, imaging and blood test results, amongst other clinically relevant parameters.

9.5. Causality

For every AE, an assessment of the relationship of the event to the administration of the vaccine will be undertaken by the PI-delegated clinician. An interpretation of the causal relationship of the intervention to the AE in question will be made, based on the type of event; the relationship of the event to the time of vaccine administration; and the known biology of the vaccine therapy. Alternative causes of the AE, such as the natural history of pre-existing medical conditions, concomitant therapy, other risk factors and the temporal relationship of the event to vaccination will be considered and investigated. Causality assessment will take place during planned safety reviews, interim analyses (e.g. if a holding or stopping rule is activated) and at the final safety analysis, except for SAEs, which should be assigned by the reporting investigator, immediately, as described in SOP for Safety Reporting for CTIMPs.

Table 11: Guidelines for assessing the relationship of vaccine administration to an AE

0	**No Relationship**	No temporal relationship to study product ***and***<br>Alternate aetiology (clinical state, environmental or other interventions); ***and*** Does not follow known pattern of response to study product
1	**Unlikely**	Unlikely temporal relationship to study product ***and*** Alternate aetiology likely (clinical state, environmental or other interventions) ***and*** Does not follow known typical or plausible pattern of response to study product.
2	**Possible**	Reasonable temporal relationship to study product; ***or***<br>Event not readily produced by clinical state, environmental or other interventions; ***or***<br>Similar pattern of response to that seen with other vaccines
3	**Probable**	Reasonable temporal relationship to study product; ***and***<br>Event not readily produced by clinical state, environment, or other interventions ***or***<br>Known pattern of response seen with other vaccines
4	**Definite**	Reasonable temporal relationship to study product; ***and***<br>Event not readily produced by clinical state, environment, or other interventions; ***and***<br>Known pattern of response seen with other vaccines

9.6. Reporting Procedures for All Adverse Events

All local and systemic AEs occurring in the 28 days following each vaccination observed by the Investigator or reported by the participant, whether or not attributed to study medication, will be recorded in paper or electronic diaries and entered onto the study database. All AEs that result in a participant's withdrawal from the study will be followed up until a satisfactory resolution occurs, or until a non-study related causality is assigned (if the participant consents to this). SAEs and Adverse Events of Special Interest will be collected throughout the entire trial period. All SAE reports will be submitted to HREC and SAHPRA regularly, as per current guidelines. A line list of all AEs will be submitted to HREC & SAHPRA as an appendix to annual progress report.

9.7. Assessment of severity

The severity laboratory adverse events will be assessed according to scales based on DAIDS AE Grading Version 2.1-July 2017 (Table 13) for adolescent adult study participants. Grading for local adverse events will be based on severity grading criteria indicated in Table 12.

Table 12: Severity grading criteria for local adverse events

Adverse Event	Grade	Intensity
Pain at injection site	1	Pain that is easily tolerated
	2	Pain that interferes with daily activity
	3	Pain that prevents daily activity
	4	A&E visit or hospitalization
Tenderness	1	Mild discomfort to touch
	2	Discomfort with movement
	3	Significant discomfort at rest
	4	A&E visit or hospitalization
Erythema at injection site*	1	2.5 - 5 cm
	2	5.1 - 10 cm
	3	>10 cm
	4	Necrosis or exfoliative dermatitis

Induration/Swelling at injection site	1	2.5 – 5 cm and does not interfere with activity
	2	5.1 - 10 cm or interferes with activity
	3	>10 cm or prevents daily activity
	4	Necrosis

*erythema ≤2.5cm is an expected consequence of skin puncture and will therefore not be considered an adverse event

Table 13: Severity grading criteria for select physical observations (Based on DAIDS Grading Table; Version 2.1 –July 2017

Vital signs	Grade 1 (mild)	Grade 2 (moderate)	Grade 3 (severe)	Grade 4 Potentially life threatening
	Mild symptoms causing no or minimal interference with usual social & functional activities with intervention not indicated	Moderate symptoms causing greater than minimal interference with usual social & functional activities with intervention indicated	Severe symptoms causing inability to perform usual social & functional activities with intervention or hospitalization indicated	Potentially life-threatening symptoms causing inability to perform basic self-care functions with intervention indicated to prevent permanent impairment, persistent disability, or death
**Arthralgia**	Joint pain causing no or minimal interference with usual social & functional activities	Joint pain causing greater than minimal interference with usual social & functional activities	Joint pain causing inability to perform usual social & functional activities	Disabling joint pain causing inability to perform basic self-care functions
**Arthritis**	Stiffness or joint swelling causing no or minimal interference with usual social & functional activities	Stiffness or joint swelling causing greater than minimal interference with usual social & functional activities	Stiffness or joint swelling causing inability to perform usual social & functional activities	Disabling joint stiffness or swelling causing inability to perform basic self-care functions
**Chills**	Symptoms causing no or minimal interference with usual social & functional activities	Symptoms causing greater than minimal interference with usual social & functional activities	Symptoms causing inability to perform usual social & functional activities	NA
**Fatigue or Malaise** *Report only one*	Symptoms causing no or minimal interference with usual social & functional activities	Symptoms causing greater than minimal interference with usual social & functional activities	Symptoms causing inability to perform usual social & functional activities	Incapacitating symptoms of fatigue or malaise causing inability to perform basic self-care functions
**Fever** (non-axillary temperatures only)	38.0 to < 38.6°C	≥ 38.6 to < 39.3°C	≥ 39.3 to < 40.0°C	≥ 40.0°C or ≥ 104.0°F
**Headache**	Symptoms causing no or minimal interference with usual social & functional activities	Symptoms causing greater than minimal interference with usual social & functional activities	Symptoms causing inability to perform usual social & functional activities	Symptoms causing inability to perform basic self-care functions OR Hospitalization indicated OR Headache with significant impairment of alertness or other neurologic function
**Myalgia** (generalized)	Muscle pain causing no or minimal interference with usual social &	Muscle pain causing greater than minimal interference with usual social &	Muscle pain causing inability to perform usual social & functional activities	Disabling muscle pain causing inability to perform basic self-care functions

	functional activities	functional activities		
**Pain** (not associated with study agent injections and not specified elsewhere)<br>*Specify location*	Pain causing no or minimal interference with usual social & functional activities	Pain causing greater than minimal interference with usual social & functional activities	Pain causing inability to perform usual social & functional activities	Disabling pain causing inability to perform basic self-care functions OR Hospitalization indicated
**Acute Allergic Reaction**	Localized urticaria (wheals) with no medical intervention indicated	Localized urticaria with intervention indicated OR Mild angioedema with no intervention indicated	Generalized urticaria OR Angioedema with intervention indicated OR Symptoms of mild bronchospasm	Acute anaphylaxis OR Life-threatening bronchospasm OR Laryngeal edema
**Blood Pressure Abnormalities**[1]<br>***Hypertension*** *(with the lowest reading taken after repeat testing during a visit)*<br>*≥ 18 years of age*	140 to < 160 mmHg systolic OR 90 to < 100 mmHg diastolic	≥ 160 to < 180 mmHg systolic OR ≥ 100 to < 110 mmHg diastolic	≥ 180 mmHg systolic OR ≥ 110 mmHg diastolic	Life-threatening consequences in a participant not previously diagnosed with hypertension (e.g., malignant hypertension) OR Hospitalization indicated
Hypotension	No symptoms	Symptoms corrected with oral fluid replacement	Symptoms AND IV fluids indicated	Shock requiring use of vasopressors or mechanical assistance to maintain blood pressure
**Dyspnea or Respiratory Distress**<br>*Report only one*	Dyspnea on exertion with no or minimal interference with usual social & functional activities OR Wheezing OR Minimal increase in respiratory rate for age	Dyspnea on exertion causing greater than minimal interference with usual social & functional activities OR Nasal flaring OR Intercostal retractions OR Pulse oximetry 90 to < 95%	Dyspnea at rest causing inability to perform usual social & functional activities OR Pulse oximetry < 90%	Respiratory failure with ventilator support indicated (e.g., CPAP, BPAP, intubation)

9.8. Reporting Procedures for Serious AEs

In order to comply with current regulations on SAE reporting to regulatory authorities, the event will be documented accurately and notification deadlines respected. SAEs will be reported on the SAE forms to members of the study team within 24 hours of the Investigators becoming aware of their occurrence, as described in SOP Safety Reporting. Copies of all reports will be forwarded for review to the Principal Investigator in South Africa and the UK Chief Investigator (as the Sponsor's representative) within 24 hours of the Investigator being aware of the suspected SAE. The DSMC will

be notified of SAEs that are deemed possibly, probably or definitely related to study interventions; the chair of DSMC will be notified immediately (within 24 hours) of the Investigators' being aware of their occurrence. SAEs assessed to be possibly, probably or definitely related to trial, or involving hospitalization or death of participant will be reported to the ethical committee(s), regulatory authority (SAHPRA) and UK chief investigator within 24 hours of investigator being aware of SAE. In addition to the expedited reporting above, the Investigator shall include all SAEs in the annual Development Safety Update Report (DSUR) report.

Grade 4 laboratory AEs should be reported as SAEs and under the category of outcome of an important medical event. A&E attendances should not routinely be reported as SAEs unless they meet the SAE definition described above.

Cases falling under the Hy's Law should be reported as SAEs. A Hy's Law Case is defined by FDA Guidance for Industry "Drug-Induced Liver Injury: Premarketing Clinical Evaluation" (2009). Any study participant with an increase in Aspartate Aminotransferase (AST) or Alanine Aminotransferase (ALT) ≥ 3x Upper Limit of Normal (ULN) together with Total Bilirubin ≥2xULN, where no other reason can be found to explain the combination of these abnormal results, e.g., elevated serum alkaline phosphatase (ALP) indicating cholestasis, viral hepatitis A, B or C, another drug capable of causing the observed injury, amongst others.

9.9. Reporting Procedures for SUSARS

All other SUSARs will be reported by the investigator to the sponsor delegate (UK Chief investigator) and to the relevant Competent Authority and to the REC and other parties as applicable. Any additional relevant information for related SAEs and deaths will be reported within 8 calendar days of the initial report. All other SUSARs will be reported within 15 calendar days.

Principal Investigators will be informed of all SUSARs for the relevant IP for all studies with the same Sponsor, whether or not the event occurred in the current trial.

9.10. Development Safety Update Report

A Development Safety Update Report (DSUR) will be prepared annually, within 60 days of the anniversary of the first approval date from the regulatory authority for each IMP. The DSUR will be submitted by the national PI to the Competent Authority, Ethics Committee, and Sponsor.

9.11. Procedures to be followed in the event of abnormal findings

Eligibility for enrolment in the trial in terms of laboratory findings will be assessed by clinically

qualified staff. Abnormal clinical findings from medical history, examination or blood tests will be assessed as to their clinical significance throughout the trial. Laboratory AEs will be assessed using specific toxicity grading scales adapted from the DAIDS AE Grading Table Version 2.1 –July 2017 for Healthy Adult and Adolescent Participants. If a test is deemed clinically significant, it may be repeated, to ensure it is not a single occurrence. If a test remains clinically significant, the participant will be informed and appropriate medical care arranged as appropriate and with the permission of the participant. Decisions to exclude the participant from enrolling in the trial or to withdraw a participant from the trial will be at the discretion of the Investigator.

9.12. Interim Reviews

The safety profile will be assessed on an on-going basis by the Investigators. The national PI and relevant site Investigators (as per the trial delegation logs) will also review safety issues and SAEs as they arise.

Interim safety reviews are planned monthly, and will include safety reviews (i) after group 1 participants have completed 14 day post dose 1 (i) and dose 2 (ii) visits, (iii) after group 3 participants have completed 14-days post dose 1, and once all participants in groups 1,2 and 3 have been enrolled.

Immunopathology data from pre-clinical studies will be assessed by the UK- CI, national PI and relevant investigators and the DSMC.

The DSMC will evaluate frequency of events, safety and efficacy data every 4-8 weeks and/or as required. The DSMC will make recommendations concerning the conduct, continuation or modification of the study.

9.13. Data Safety Monitoring Committee

A Data Safety Monitoring Committee (DSMC) has been appointed to oversee the UK trial, and have agreed to oversee the South African study as well. A South African senior scientist has been co-opted onto this international DSMC. The DSMC will:

a) periodically review and evaluate the accumulated study data for participant safety, study conduct, progress, and efficacy.

b) make recommendations concerning the continuation, modification, or termination of the trial.

There will be a minimum of three appropriately qualified committee members of whom one will be the designated chair. The DSMC will operate in accordance with the trial specific charter, which will be established before recruitment starts. In order to maintain continuity, the members of the DSMC overseeing the UK trial of the ChAdOx1-nCoV-19 vaccine (CoV001) will also be members of the DSMC for this trial. At least one African scientist will be added to the existing trial DSMC.

The chair of the DSMC may be contacted for advice and independent review by the Investigator or trial Sponsor in the following situations:

- Following any SAE deemed to be possibly, probably or definitively related to a study intervention.
- Any other situation where the Investigator or trial Sponsor feels independent advice or review is important.

The DSMC will review SAEs deemed possibly, probably or definitively related to study interventions. The DSMC will be notified within 24 hours of the Investigators' being aware of their occurrence. The DSMC has the power to place the study on hold if deemed necessary following a study intervention-related SAE.

9.14. Safety Group Holding Rules

Safety holding rules have been developed considering the fact that this trial will enroll people living with HIV, who have not previously been enrolled in a trial utilizing this IP.

Solicited AEs are those listed as foreseeable ARs, occurring within the first 7 days after vaccination (day of vaccination and six subsequent days). 'Unsolicited adverse events' are adverse events other than the foreseeable ARs occurring within the first 7 days, or any AEs occurring after the first 7 days after vaccination.

9.15. Holding rules

Group holding rules mentioned below will apply to all study Groups

- **Solicited local adverse events:**
  - If more than 25% of doses of the vaccine at a given time point (e.g. Day 0, Day 28) in a study group are followed by the same Grade 3 solicited local adverse event beginning within 2 days after vaccination (day of vaccination and one subsequent day) and persisting at Grade 3 for >72 hrs.

- **Solicited systemic adverse events:**
  - If more than 25% of doses of the vaccine at a given time point (e.g. Day 0, Day 28) in a study group are followed by the same Grade 3 solicited systemic adverse event beginning within 2 days after vaccination (day of vaccination and one subsequent day) and persisting at Grade 3 for >72 hrs.
- **Unsolicited adverse events:**
  - If more than 25% of doses of the vaccine at a given time point (e.g. Day 0, Day 28) in a study group are followed by the same Grade 3 unsolicited adverse event beginning within 2 days after vaccination (day of vaccination and one subsequent day) and persisting at Grade 3 for >72 hrs.
- **Laboratory adverse event:**
  - If more than 25% of doses of the vaccine at a given time point (e.g. Day 0, Day 28) in a study group are followed by the same Grade 3 laboratory adverse event beginning within 2 days after vaccination (day of vaccination and one subsequent day) and persisting at Grade 3 for >72 hrs.
- **Any serious adverse event considered possibly, probably or definitely related to vaccination.**
  - If an SAE occurs in any one individual, which is possibly, probably or definitely related to vaccination this would trigger a holding rule. There are two exemptions from this rule, which would not activate a holding rule. These include:
    - COVID-19 related hospital admissions considered to be at least possibly related to ChAdOx1 nCoV-19 (e.g. if considered to be a clinical presentation of a disease enhancement episode). COVID-19 related SAEs will be regularly reviewed by the DSMB, and a single event will not trigger a holding rule.
    - SAEs reported under the Hy's Law requirement will not necessarily trigger a holding rule. These cases will also be reviewed by the DSMC

If any of the above holding rules are activated, then further vaccinations in any group will not occur until a safety review by the DSMC, study sponsor and the protocol Co-chairs has been conducted and it is deemed appropriate to restart dosing. The Regulatory Authority will be informed and a request to restart dosing with pertinent data will be submitted. The safety review will consider:

- The relationship of the AE or SAE to the vaccine.

- The relationship of the AE or SAE to the vaccine dose, or other possible causes of the event.
- If appropriate, additional screening or laboratory testing for other participants to identify those who may develop similar symptoms and alterations to the current Participant Information Sheet (PIS) are discussed.
- New, relevant safety information from ongoing research programs on the various components of the vaccine.

The local ethics committee and vaccine manufacturers will also be notified if a holding rule is activated or released.

All vaccinated participants will be followed for safety until resolution or stabilisation (if determined to be chronic sequelae) of their AEs.

9.15.1. Individual stopping rules

In addition to the above stated holding rules, stopping rules for individual participants will apply (i.e., indications to withdraw individuals from further vaccinations). Study participants who present with at least one of the following stopping rules will be withdrawn from further vaccination in the study:

- **Local reactions:** Injection site ulceration, abscess or necrosis
- **Laboratory AEs:** the participant develops a Grade 3 laboratory AE considered possibly, probably or definitely related within 7 days after vaccination and persisting continuously at Grade 3 for 72hrs.
- **Systemic solicited adverse events:** the participant develops a Grade 3 systemic solicited AE considered possibly, probably or definitely related within 2 days after vaccination (day of vaccination and one subsequent day) and persisting continuously at Grade 3 for > 72hrs.
- **Unsolicited adverse events:**
    - the participant has a Grade 3 adverse event, considered possibly, probably or definitely related to vaccination, persisting continuously at Grade 3 for >72hrs.
    - the participant has a SAE considered possibly, probably or definitely related to vaccination.
    - the participant has an acute allergic reaction or anaphylactic shock following the administration of vaccine investigational product.
- **Any serious adverse event considered possibly, probably or definitely related to vaccination.**

If a participant has an acute respiratory illness (moderate or severe illness with or without fever) or a fever (oral temperature greater than 37.8°C) at the scheduled time of administration of investigational product/placebo, the participant will not be enrolled and will be withdrawn from the study.

All vaccinated participants will be followed for safety until the end of their planned participation in the study or until resolution or stabilisation (if determined to be chronic sequelae) of their AEs, providing they consent to this.

In addition to these pre-defined criteria, the study can be put on hold upon advice of the DSMC, South African and UK Co-Chairs, Study Sponsor, regulatory authority, Ethical Committee(s), for any single event or combination of multiple events which, in their professional opinion, jeopardise the safety of the participants or the reliability of the data.

10. STATISTICS

10.1. Description of Statistical Methods

A fully detailed statistical analysis plan will be developed and signed by the Co-chairs prior to any data analysis being conducted. For the efficacy endpoints, VE will be calculated as 1-RR and 95% confidence intervals estimated using the Clopper-Pearson exact method. In brief, the analysis will incorporate the following:

10.1.1 Efficacy endpoints:

Criteria for clinical diagnosis of incident COVID-19 *disease* in adults (Adapted from CEPI recommendations for standardisation COVID-19 vaccine efficacy trials).

Virologically confirmed COVID-19 clinical disease will be defined as an acute respiratory illness that is clinically consistent with COVID-19 based on the presence of criteria indicated in Table 5 and a positive SARS-CoV-2 specific reverse transcriptase polymerase chain reaction (RT-PCR). An expert external committee of at least two physicians will be convened to adjudicate on inclusion of clinical endpoints of incident COVID-19 cases for inclusion in the VE analyses.

10.2. Primary efficacy [objective] and endpoint in COVID-19-naive persons

The primary efficacy [objective] and endpoint include PCR positive symptomatic COVID-19 occurring in participants that were COVID-19 naïve at randomization who received two-doses of the planned study-allocated intervention, and where the first episode of COVID-19 occurred more than 14 days after the second dose of study-drug. COVID naïve will be defined as sero-negative at time of randomization based on a high sensitivity serology antibody targeted at the whole-length spike protein and receptor binding domain protein, and tested negative on nasal swab for SARS-CoV-2 by molecular detection. This analysis will include participants randomised to Group-1 being analysed together with Group-2 participants, all of whom will have received a two-dose schedule of study-intervention.

A sensitivity analysis will be conducted using a modified intention-to-treat (mITT) approach. This analysis will include COVID-19 naïve participants who received two doses of either the investigational product or placebo, regardless of whether it was the planned study-allocation intervention.

Only events that occur more than 14 days after vaccination will be included in mITT efficacy evaluations, to allow for exclusion of SARS-CoV-2 infections that may have occurred within 7 days of the 2nd dose and may have been asymptomatic prior to the anticipated optimal immune response after the second dose of vaccine. Vaccine efficacy (VE) will be calculated as (1 – RR) x 100%, where RR is the relative risk of symptomatic infection (ChADOx1 nCOV-19: placebo) and 95% confidence intervals will be presented.

Cumulative incidence of COVID-19 disease will be presented using the Kaplan-Meier method.Depending on the rate of accrual of endpoint cases meeting the primary-endpoint criteria in this study and phase II/III efficacy studies ChAdOx1 nCoV-19 that are currently underway in Brazil (ISRCTN89951424) and the United Kingdom (NCT: 04400838), it may be necessary to undertake a pooled analysis for the primary endpoint across the studies to provide an early readout of the efficacy of the ChAdOx1 nCoV-19. The study design and endpoint definitions across the studies are similar, and the categorisation of COVID-19 cases would be aligned. Should this be pursued, SAHPRA and the Ethics committees will be engaged to discuss the merits thereof. It is anticipated that blinding will be maintained on the part of the study-staff and the study-participants throughout this process on an interim pooled-analysis.

10.3. Secondary efficacy [objectives], endpoints and analyses, for overall population and based on COVID-19 sero-status at time of randomization

VE in preventing other virologically-confirmed COVID-19 clinical disease endpoints will include all cases occurring onward 14 days after a second dose; and from 21 days after a single dose, for the following endpoints:.

a. VE in preventing virologically-confirmed COVID-19 clinical disease irrespective of COVID-19 sero-status at randomization, and in those who were sero-positive at randomization.
b. VE in preventing PCR positive COVID-19 disease cases
c. VE in preventing moderate-severe confirmed COVID-19 disease.
d. VE in preventing severe confirmed COVID-19 disease.
e. VE in preventing LRTI associated with virologically-confirmed COVID-19 clinical disease
f. VE in preventing hospitalization due to virologically confirmed COVID-19 disease
g. VE in preventing all-cause LRTI (overall and stratified by hospitalization or not) irrespective of test result for SARS-COV-2.
h. VE using the Oxford Primary Outcome definition

10.4. Exploratory efficacy endpoints could include

a. VE in preventing death associated with virologically-confirmed COVID-19 clinical disease
b. VE in preventing virologically-confirmed COVID-19 disease or all-cause LRTI requiring supplemental oxygenation
c. VE in preventing virologically-confirmed COVID-19 disease or all-cause LRTI mechanical ventilation
d. VE in preventing virologically-confirmed COVID-19 disease or all-cause LRTI multi-organ dysfunction syndrome (MODS)
e. VE in preventing virologically-confirmed COVID-19 disease or all-cause LRTI all-cause mortality
f. VE in preventing asymptomatic SARS-CoV-2 infection (samples collected at scheduled study visits); i.e. no presence of any of the symptoms contributing to COVID-19 disease outcome, but virologically confirmed infection.
g. VE against sero-conversion suggestive of SARS-CoV-2 infection tested using a N-protein IgG assay.

10.4.1. Safety & Reactogenicity

Counts and percentages of each local and systemic solicited adverse reaction from diary cards, and all unsolicited AEs, and SAEs of special interest will be presented for each group.

10.4.2. Immunogenicity

Immune responses to be evaluated as per Table 8, Table 9, Table 10 include:

1. ELISA or Luminex assay (to be finalized based on current laboratory investigations) for whole spike protein and receptor binding domain.
2. ELISA or Luminex assay for N-protein IgG (to discriminate sero-conversion that is independent of SARS-CoV-2 proteins included in the vaccine. This assay is currently being developed and addresses an exploratory objective of the study.
3. Cell mediated immune response using an ELISPOT assay.
4. Th1 and TH2 cytokine profile using a Luminex assay.
5. Neutralization assays and Fc effector assays using pseudotyped and/or live virus assays

Currently a WHO COVID-19 serology working group (Solidarity II – COVID-19 Seroepidemiology) has established standard research sera (NIBSC code 20/130) and serum controls panel (NIBSC code 20/118) for harmonization of assays to be used across vaccine studies, and the detail of the proposed assays will be adapted per the latest development and recommendation by the WHO

serology working group.

Highly skewed ELISA data will be log-transformed prior to analysis. The geometric mean concentration and associated 95% confidence interval will be summarised for each group at each time point, by computing the anti-log of the mean difference of the log-transformed data. Neutralisation measurements will use an assay adapted from well-validated existing HIV-based pseudovirus neutralization assays using the pNL4–3.luc.R-E HIV construct with a SARS-CoV-2 spike protein. This assay is being developed and validated in collaboration with Dr David Montefiori, Duke University. Fc effector functionality, including antibody dependent cellular cytotoxicity, complement deposition, and phagocytosis will assess responses to spike trimer or the receptor binding domain

10.5. The Number of Participants

10.5.1. Sample size

Primary safety objective

Table 14 shows the probability of observing zero, at least one or at least two participants with an event among groups of size 25 and 50 for a range of true event probabilities. For example, if the true rate of a serious event is 0.01, there is a 77.8% chance that there will be no participants that experience this event in a group of 25 participants and a 22.2% chance of at least one participant who experiences the event.

Table 14: Calculated probability of observing zero, at least one or at least two participants with and event among groups of size 25 or 50 for a range of true event probabilities:

	Group size = 25			Group size = 50		
True event rate (%)	Zero participants with an event (%)	At least one participant with an event (%)	At least two participants with an event	Zero participants with an event	At least one participant with an event	At least two participants with an event
1	77.8	22.2	2.6	60.5	39.5	8.9
5	27.7	72.3	35.8	7.7	92.3	72.1
10	7.2	92.8	72.9	0.5	99.5	96.6
20	0.4	99.6	97.3	0	100	100
30	0	100	99.8	0	100	100

To estimate the true rate of a serious event, Exact Clopper-Pearson two-sided 95% confidence intervals (CIs) will be calculated. Table 15 lists calculated 95% CIs for the true rate of a serious event when 0, 1 or 2 participants observe events for a group of size 25 or 50

Table 15: Exact Clopper-Pearson 95% confidence intervals (CI) when 0,1, or 2 participants observe a serious event for a group size of 25 or 50

Observed number of participants with a serious event	95% CI for the true rate (%) of a serious event (group size = 25)	95% CI for the true rate (%) of a serious event (group size = 50)
0	(0, 13.7)	(0, 7.1)
1	(0.1, 20.4)	(0.1, 10.6)
2	(1, 26)	(0.5, 13.7)

Primary immunogenicity

The minimum detectable difference in response rates between 2 groups (group size =25) for 80% and 90% power is listed in Table 16.

Table 16: Minimum detectable difference in response rates between 2 groups calculated for various true response rates in the placebo group for groups size of 25 and statistical power of 80% and 90%.

True response rate in unvaccinated group (%)	True response rate in vaccinated group (%)	
	80% power	90% power
10	48.4	54.2
20	60.5	66.0
30	70.8	76.2
40	80.5	85.5
50	88.9	92.5

*Based on Fisher's exact test

Primary efficacy objective

Sample size calculations based on the total number of cases required to conclude with 80% power the lower limit of a two-sided 95% confidence interval for vaccine efficacy (VE, success criteria) is greater than 0% and 10% are shown in Table 17 for VE ranging from 60%-90% and attack rate in the unvaccinated population ranging from 5%-20%. Sample sizes are adjusted for a 10% loss to follow-up.

Table 17: Sample size for group 2 required to conclude with 80% power the lower limit of a two-sided 95% confidence interval for vaccine efficacy (VE) is greater than 0% and 10%.

Attack rate in unvaccinated participants (%)			1.5	2	2.5	3	3.5	4	5	10	15	20
Total cases (total cases in vaccinated group)	Success criteria	VE										
42 (12)	0%	60%	4447	3336	2669	2225	**1907**	1669	1336	669	445	336
28 (6)	0%	70%	3192	2394	1916	1596	1369	1198	958	480	320	240
17 (3)	0%	80%	2100	1576	1260	1052	900	789	632	316	212	158
12 (1)	0%	90%	1618	1214	972	809	694	607	487	245	163	123
57 (16)	10%	60%	6034	4525	3620	3018	2587	2263	1812	907	605	454
32 (7)	10%	70%	3649	2736	2189	1825	1565	1369	1096	549	367	276
19 (3)	10%	80%	2347	1760	1409	1174	1007	880	705	354	236	178
13 (1)	10%	90%	1752	1314	1052	876	752	658	527	265	176	134

Assuming a final total sample size in Group 2 of 1900 (950 per arm), the power to conclude the lower limit of a 95% confidence interval for VE is greater than 10% is listed below in for various assumed true VE and attack rates in the unvaccinated population.

Table 18: Calculated power to conclude the lower limit of a 95% confidence interval for VE is greater than 0% or 10% for a total sample size of 1900 (950 per arm).

**Power (Exact method)**			
**VE (%)**	Attack rate in unvaccinated (%)	Success Criteria	
		0%	10%
**60**	2	72.78	60.06
**60**	2.5	81.46	72.09
**60**	3.5	93.52	83.97
**60**	3	90.62	77.3
**60**	4	97.35	92.67
**60**	5	98.91	96.88
**60**	10	100	99.96
**60**	20	100	100
**70**	2	91.14	83.54
**70**	2.5	96.77	88.24
**70**	3.5	99.28	97.07
**70**	3	98.02	96.06
**70**	4	99.74	98.86
**70**	5	99.97	99.83
**70**	10	100	100
**70**	20	100	100

10.6. Procedure for Accounting for Missing, Unused, and Spurious Data.

All available data will be included in the analysis

10.7. Inclusion in Analysis

All vaccinated participants will be included in the analysis and will be analysed according to vaccine received.

10.8. Interim analysis

The independent DSMC will meet regularly to review safety data and will assess whether the assumptions underlying the sample size calculation are in line with the observed cases.

11. DATA MANAGEMENT

11.1. Data Handling

The national principal investigator will be responsible for all data that accrue from the trial.

All trial data including participant diary will be recorded directly into an Electronic Data Capture (EDC) system (REDCap) or onto a paper source document for later entry into EDC if direct entry is not available. This includes safety data, laboratory data and outcome data. Any additional information that needs recording but is not relevant for the CRF (such as signed consent forms etc.) will be recorded on a separate paper source document. All documents will be stored safely and securely in confidential conditions.

All adverse event data (both solicited and unsolicited) reported by the participant will be entered onto a participant's paper diary card for a maximum of 28 days following administration of the IP. The Diary provides a full audit trial of edits and will be reviewed at each review time-points indicated in the schedule of events. Any adverse event continuing beyond the period of the diary will be copied into the eCRF and followed to resolution, if there is a causal relationship to the IP, or to the end of the study if there is no causal relationship.

The participants will be identified by a unique trial specific number and code in any database. The name and any other identifying detail will only be included in password-protected trial electronic logs, which will be used for tracing and medical records and laboratory results and conducting surveillance calls as required. Personal identifiers will not be accessible by any person/ institution outside of immediate study team.

The EDC system (CRF data) uses a relational database (MySQL/ PostgreSQL/ REDCap) via a secure web

interface with data checks applied during data entry to ensure data quality. The database includes a complete suite of features which are compliant with GCP, EU and UK regulations and Sponsor security policies, including a full audit trail, user-based privileges, and integration with the institutional LDAP server. The REDCap, MySQL and PostgreSQL database and the webserver will both be housed on secure servers maintained by the University of the Witwatersrand and RMPRU IT personal. Backups will be stored in accordance with the IT department schedule of daily, weekly, and monthly retained for one month, three months, and six months, respectively. The IT servers provide a stable, secure, well-maintained, and high capacity data storage environment. REDCap and OpenClinica are widely-used, powerful, reliable, well- supported systems. Access to the study's database will be restricted to the members of the study team by username and password.

11.2. Record Keeping

The Investigators will maintain appropriate medical and research records for this trial, in compliance with GCP and regulatory and institutional requirements for the protection of confidentiality of participants. The South African national principal investigator, co-Investigators and clinical research nurses will have access to records. The Investigators will permit authorised representatives of the Sponsor(s), as well as ethical and regulatory agencies to examine (and when required by applicable law, to copy) clinical records for the purposes of quality assurance reviews, audits and evaluation of the study safety and progress.

All trial records will be stored for a minimum of 15 years after the end of the trial at a secure archiving facility. If participants consent to be contacted for future research, information about their consent form will be recorded, retained and stored securely and separately from the research data. If participants consent to have their samples stored and used in future research, information about their consent form will be recorded, retained and stored securely as per sample storage procedures and SOP.

11.3. Source Data and Case Report Forms (CRFs)

All protocol-required information will be collected in CRFs designed by the Investigator. All source documents will be filed in the CRF. Source documents are original documents, data, and records from which the participant's CRF data are obtained. For this study, these will include, but are not limited to, participant consent form, blood results, community clinic and private general practitioner notes held by participant, laboratory records, diaries, medical records and correspondence. In the majority of cases, CRF entries will be considered source data as the CRF is the site of the original recording (i.e. there is no other written or electronic record of data). In this study this will include, but is not limited to medical history, medication records, vital signs, physical examination records, urine assessments, blood results, adverse event data and details of vaccinations. All source data and participant CRFs will be stored

securely.

11.4. Data Protection

The study protocol, documentation, data and all other information generated will be held in strict confidence. No information concerning the study or the data will be released to any unauthorised third party, without prior written approval of the sponsor.

11.5. Data Quality

Data collection tools will undergo appropriate validation to ensure that data are collected accurately and completely. Datasets provided for analysis will be subject to quality control processes to ensure analysed data is a true reflection of the source data.

Trial data will be managed in compliance with local data management SOPs. If additional, study specific processes are required, an approved Data Management Plan will be implemented.

11.6. Archiving

Trial data may be stored electronically on a secure server, and paper notes will be kept in a key-locked filing cabinet at the site. All essential documents will be retained for a minimum of 15 years after the trial has finished. The need to store study data for longer in relation to licensing of the vaccine will be subject to ongoing review.

General archiving procedures will be conducted in compliance to local SOP for Archiving.

12. QUALITY CONTROL AND QUALITY ASSURANCE PROCEDURES

12.1. Investigator procedures

Approved site-specific standard operating procedures (SOPs) will be used at all clinical and laboratory sites.

12.2. Monitoring

Regular monitoring will be performed according to GCP by the monitor. Following written SOPs, the monitor will verify that the clinical trial is conducted and data are generated, documented and reported in compliance with the protocol, GCP and the applicable regulatory requirements. The site will provide direct access to all trial related source data/documents and reports for the purpose of monitoring and auditing by the Sponsor and inspection by local and regulatory authorities.

12.3. Protocol deviation

Any deviations from the protocol will be documented in a protocol deviation form and filed in the trial master file. Each deviation will be assessed as to its impact on participant safety and study conduct. Significant protocol deviations will be listed in the end of study report.

12.4. Audit & inspection

The QA manager conducts systems based internal audits to check that trials are being conducted according to local procedures and in compliance with study protocols, departmental SOPs, GCP and applicable regulations.

The Sponsor, trial sites, and ethical committee(s) may carry out audit to ensure compliance with the protocol, GCP and appropriate regulations.

GCP inspections may also be undertaken by the HREC or SAHPRA to ensure compliance with protocol and the National Health Act No 61 (as amended) and Guidelines in Good Clinical Practice for the conduct of trials with human participants in South Africa 2006, as amended. The Sponsor will assist in any inspections and will support the response to the HREC/ SAHPRA as part of the inspection procedure.

13. ETHICS AND REGULATORY CONSIDERATIONS

13.1. Declaration of Helsinki

The Investigators will ensure that this study is conducted according to the principles of the current revision of the Declaration of Helsinki.

13.2. Guidelines for Good Clinical Practice

The Investigator will ensure that this trial is conducted in accordance with relevant regulations and with Good Clinical Practice.

13.3. Ethical and Regulatory Approvals

Following Sponsor approval, the protocol, informed consent form, participant information sheet and any proposed advertising material will be submitted to an appropriate Research Ethics Committee (REC: University of the Witwatersrand, OxTREC, University of Cape Town, University of Stellenbosch), regulatory authorities (SAHPRA in South Africa, MHRA in the UK), and host institution(s) for written approval. No amendments to this protocol will be made without consultation with, and agreement of, the Sponsor and national principal investigator.

The Investigator is responsible for ensuring that changes to an approved trial, during the period for

which regulatory and ethical committee(s) approval has already been given, are not initiated without regulatory and ethical committee(s)' review and approval except to eliminate apparent immediate hazards to the participant (i.e. as an Urgent Safety Measure).

13.4. Participant Confidentiality

The study will comply with the Protection of Personal Information **Act**, No 4 of and relevant Data Protection Act, which require data to be de-identified as soon as it is practical to do so. The processing of personal data of participants will be minimised by making use of a unique participant study number only on all study documents and any electronic database(s), with the exception of informed consent forms and participant ID logs. All documents will be stored securely and only accessible by study staff and authorised personnel. The study staff will safeguard the privacy of participants' personal data. A separate confidential file containing identifiable information will be stored in a secured location in accordance with the current data protection legislation. Photographs taken of vaccination sites (if required, with the participant's written, informed consent) will not include the participant's face and will be identified by the date, trial code and participant's unique identifier. Once developed, photographs will be stored as confidential records, as above. This material may be shown to other professional staff, used for educational purposes, or included in a scientific publication.

If participants are diagnosed with COVID-19 during the course of the study then the study team will pass on their details to the local health protection team, if required, in line with the relevant notifiable disease legislation. Samples collected for the purposes of COVID-19 diagnosis might be sent to reference labs in South Africa alongside their personal data. This would be in line with the national guidance and policy for submitting samples for testing at reference labs.

14. FINANCING AND INSURANCE

14.1. Financing

The vaccine development and manufacture study is funded through UK Research and Innovations. The vaccine will be supplied free of charge to South African sites by UK chief collaborator.
Funding for the trial conduct will be finalized prior to trial initiation. National PI is in discussion with several stakeholders who may contribute to trial funding, including The Bill & Melinda Gates Foundation and South African Medical Research Council.

14.2. Insurance

The investigators have medical malpractice insurance. Trial-specific insurance has been obtained and

insurance certificates will be shared with regulatory and ethics committees and will be available at all sites prior to trial initiation. Clinical management of COVID-19 will be undertaken by public or private health care providers (participant's choice/ insurance dependent), and will be under relevant institution indemnity.

14.3. Compensation

Participants will be compensated for their time, the inconvenience of having blood tests and procedures, and their travel expenses. Compensation rates will be aligned to those recommended by SAHPRA.

15. Publication Policy

South African investigators/collaborators and UK collaborators will be involved in reviewing drafts of the manuscripts, abstracts, press releases and any other publications arising from the study.

16. DEVELOPMENT OF A NEW PRODUCT/PROCESS OR THE GENERATION OF INTELLECTUAL PROPERTY

The IP has been developed by the University of Oxford and ownership of IP vests in the University of Oxford. Several UK investigators are applicants or co-inventors on previous patent filings or patents related to ChAdOx1 vaccines. The University of Oxford, which is partnered with the Oxford University Hospitals NHS Foundation Trust in the NIHR Oxford Biomedical Research Centre, is committed to the translational progress and commercial development of healthcare products potentially meeting medical and global health needs, and does and will work with commercial partners towards these goals.

17. References

1. Zhu, N., et al., A Novel Coronavirus from Patients with Pneumonia in China, 2019. New England Journal of Medicine, 2020. **382**(8): p. 727-733.
2. Lu, R., et al., Genomic characterisation and epidemiology of 2019 novel coronavirus: implications for virus origins and receptor binding. Lancet (London, England), 2020. **395**(10224): p. 565-574.
3. Li, F., Structure, Function, and Evolution of Coronavirus Spike Proteins. Annual review of virology, 2016. **3**(1): p. 237-261.
4. Zhou, P., et al., A pneumonia outbreak associated with a new coronavirus of probable bat origin. Nature, 2020.
5. Alharbi, N.K., et al., ChAdOx1 and MVA based vaccine candidates against MERS-CoV elicit neutralising antibodies and cellular immune responses in mice. Vaccine, 2017. **35**(30): p. 3780-3788.
6. Liu, L., et al., Anti–spike IgG causes severe acute lung injury by skewing macrophage responses during acute SARS-CoV infection. JCI insight, 2019. **4**(4).
7. Tseng, C.T., et al., Immunization with SARS coronavirus vaccines leads to pulmonary immunopathology on challenge with the SARS virus. PLoS One, 2012. **7**(4): p. e35421.
8. Weingartl, H., et al., Immunization with modified vaccinia virus Ankara-based recombinant vaccine against severe acute respiratory syndrome is associated with enhanced hepatitis in ferrets. J Virol, 2004. **78**(22): p. 12672-6.
9. Agrawal, A.S., et al., Immunization with inactivated Middle East Respiratory Syndrome coronavirus vaccine leads to lung immunopathology on challenge with live virus. Hum Vaccin Immunother, 2016. **12**(9): p. 2351-6.
10. Alharbi, N.K., et al., Humoral Immunogenicity and Efficacy of a Single Dose of ChAdOx1 MERS Vaccine Candidate in Dromedary Camels. Scientific reports, 2019. **9**(1): p. 1-11.
11. Munster, V.J., et al., Protective efficacy of a novel simian adenovirus vaccine against lethal MERS-CoV challenge in a transgenic human DPP4 mouse model. npj Vaccines, 2017. **2**(1): p. 1-4.
12. Antrobus, R.D., et al., Clinical assessment of a novel recombinant simian adenovirus ChAdOx1 as a vectored vaccine expressing conserved Influenza A antigens. Mol Ther, 2014. **22**(3): p. 668-74.
13. Coughlan, L., et al., Heterologous Two-Dose Vaccination with Simian Adenovirus and Poxvirus Vectors Elicits Long-Lasting Cellular Immunity to Influenza Virus A in Healthy Adults. EBioMedicine, 2018.
14. Wilkie, M., et al., A phase I trial evaluating the safety and immunogenicity of a candidate tuberculosis vaccination regimen, ChAdOx1 85A prime - MVA85A boost in healthy UK adults. Vaccine, 2020. **38**(4): p. 779-789.
15. Modjarrad, K., MERS-CoV vaccine candidates in development: The current landscape. Vaccine, 2016. **34**(26): p. 2982-7.
16. Folegatti PM, Ewer KJ, Aley PK, et al. Safety and immunogenicity of the ChAdOx1 nCoV-19 vaccine against SARS-CoV-2: a preliminary report of a phase 1/2, single-blind, randomised controlled trial. *Lancet* 2020; **396**(10249): 467-78.
17. US Department of Health and Human Services. Food and Drug Administration. Centre for Biological Evaluation and research. June 2020: https://www.fda.gov/regulatory-information/search-fda-guidance-documents/development-and-licensure-vaccines-prevent-covid-19.

Appendix 1: Amendment history

Summary of protocol amendments: Version 1.0 to version 2.0 **11th May 2020**

Protocol Title: An adaptive phase I/IIa randomised placebo- controlled study to determine safety, immunogenicity and efficacy of non-replicating ChAdOx1 SARS-CoV-2 vaccine in South African adults living without HIV; and safety and immunogenicity in adults living with HIV

Protocol Number: ChAdOx1 nCoV-19_ZA_phI/II

Protocol version, date: Revised Protocol version 2, 8th May 2020

Section	Amendment made	Justification
SA collaborators	Added Dr Alane Izu	Statistician at RMPRU. Provided sample size calculations for protocol, will oversee database development, data analysis
Inclusion criteria, trial population	Increased upper age limit to 65 years	The upper age limit of participants has been increased from 55 years to 65 years in line with HREC recommendation. Although co-morbid disease prevalence increases with increasing age, not all adults over 55 years are vulnerable and should be given the opportunity to partake in this trial, as long as inclusion and exclusion criteria are fulfilled.
Sample size	Group 2 has increased by 2150, from 550 to 2700.<br><br>Group 2a= 550 (original group 2)<br>**Group 2b= 2150 (additional)**<br><br>Group 1 and 3 sample size remains 50.<br><br>**TOTAL sample size = 2800**	Considering the unpredictability of the force of SARS-CoV-2 infection and the lower than anticipated attack rate for the primary-endpoint cases in the study being undertaken in the UK, the sample size for Group 2 (efficacy cohort) has been expanded from the 550 included in protocol version 1.0, dated 24th April 2020 to 2700 in protocol version 2.0, 8th May 2020.<br><br>Enrolling up to a total of 2,700 people without HIV in Group-2, will provide 80% power to detect at least a 60% vaccine efficacy (lower bound of 95%CI >0) with an attack rate of 2.5% in the placebo arm. Ongoing review of the number of COVID-19 cases accrued during the course of the study, may lend itself to enrolling smaller number of participants should the attack rate be higher than 2.5%.

Section	Amendment made	Justification
Table of groups, visit schedule table	Protocol amendment will not be required if group 2 participants receive 2 doses	Safety and immunogenicity data from the UK trial, COV001, and group 1 of this trial will be reviewed by the DSMC at least monthly. The DSMC will be tasked to make a decision, based on these results, regarding whether participants in Group 2 will receive one or two doses of IP. This decision will be communicated as a formal DSMC resolution communication to investigators, SAHPRA and WHREC. The option of the 2nd dose in group 2 has been built into the study design and events schedule. A protocol amendment would therefore not be necessary.
Table of groups, visit schedule table	Blood collection in PAXgene® Blood RNA tube added	Blood collection in PAXgene® Blood RNA tube has been added in line with COV001 protocol and at the advice of funders, BMGF.<br><br>The PAXgene® Blood RNA tube assists in stabilisation of intracellular RNA, thereby improving accuracy and reproducibility of gene expression data.
Table of groups, visit schedule table	HbA1C added to screening blood	The HbA1C is a test done to identify glycated haemoglobin. Measurement of HbA1C gives a clear indication of the average blood glucose levels over the duration of the life of the red blood cell, which is 8-12 weeks. High HbA1C levels indicate poor blood glucose level over time, either in known diabetics or undiagnosed diabetics/ pre-diabetic conditions.<br>Participants with high HbA1C levels will be referred to relevant medical teams for further assessment and management of diabetes or pre-diabetic conditions.
Visit schedule tables, synopsis, main protocol body	Added visit schedule table for group 2b	Group 2b is an extended efficacy cohort. Participants in Group 2b (HIV-uninfected adults) will have fewer scheduled visits and sample collections than participants in group 2a.
Exclusion criteria	Added: Use of any unproven registered and unregistered treatments for COVID-19	SAHPRA request.

Section	Amendment made	Justification
7.1 Schedule of attendance	Blood volume updated to include amended testing schedule for all groups	Group 1 (305ml) and Group 3 (315ml) and Group 2a (205ml) volumes increased by the addition of HbA1C and PAXgene tests. Group 2b participants will have 160ml collected.
7.3 Blood tests, nasal swabs/ saliva & urinaysis	Immunology blood test details added	SAHPRA request; details of immunology testing included.
8.6	GMO section updated	Amended in accordance with South African regulations.
9.6. Reporting procedures for all AEs	Added ‘All SAE reports will be submitted to HREC and SAHPRA regularly, as per current guidelines. A line list of all AEs will be submitted to HREC and SAHRA as an appendix to annual progress report’	SAHPRA request.<br>The applicant confirms its commitment to adhering to the South African safety reporting guidelines.
10.1 Description of Statistical methods	Expanded	SAHPRA request<br>A complete statistical analysis plan will be developed for the trial.

<u>Summary of protocol amendments: Version 2.0 to version 2.1</u> 29th May 2020

Protocol Title: An adaptive phase I/IIa randomised placebo- controlled study to determine safety, immunogenicity and efficacy of non-replicating ChAdOx1 SARS-CoV-2 vaccine in South African adults living without HIV; and safety and immunogenicity in adults living with HIV

Protocol Number: ChAdOx1 nCoV-19_ZA_phI/II

Protocol version, date: Revised Protocol version 2.1, 29th May 2020

Section	Amendment made	Justification
Title	Removed 'a' from Phase IIa	
Sample size adjustment	Reduced from 2800 to 2000 overall	The sample size has been calculated using 3.5% attack rate, instead of 2.5% attack rate. Other parameters used in sample size calculation remain unchanged (60% VE, success criteria 0%, 80% power)
Adjustment of group 2a and 2b	Group 2a reduced from 550 to 250<br>Group 2b reduced from 2150 to 1650	The UK COV001 trial (>1000 enrolled) and group 1 of this trial will contribute to the intensive immunogenicity analyses. Group 2a sample size has been reduced to 250
Schedule of events tables clarified	Full physical examinations at screening, vaccination 2 and illness visits. Targeted physical examinations at other visits.<br>Pulse oximetry added to observation	Protocol schedule of events tables and visit details have been clarified, to include full physical examination at screening, vaccination and illness visits. Targeted examinations can be done at other visits<br><br>Pulse oximetry added to physical observation to allow for adequate monitoring and clear classification of respiratory symptoms
Schedule of events table	HIV testing of HIV-negative participants at trial conclusion added	To assess possible differences in immunological responses in participants who sero-convert to HIV-positive during the trial participation
Timing of group 3 enrolment	Group 3 enrolment will either be in parallel with or will follow on from group 1 enrolment. Section 5 updated	More than 1000 participants have been enrolled into The UK's COV001 trial and will have had at least 6 weeks follow up prior to trial initiation of trial in South Africa. As of 28th May 2020, no significant vaccine-relates AEs or SAEs have been recorded in HIV-negative participants in the UK.
Screening window clarified	Screening window confirmed to be 14 days prior to vaccination.	Text portions of protocol (6.3.2 & 7.4.1) updated to ensure consistency (previously had 7 day window, not 14 days)
7.3. Blood tests, nasal swab/ saliva & urinalysis	Details of immunological assays have been added to the protocol	At reviewers' request
Clinical COVID-19 disease:	Added arthralgia, fatigue, nasal	As new research emerges, the clinical diagnostic criteria for COVID-19 is being

objectives, analysis	congestion, nausea, vomiting to clinical symptoms	amended. Protocol amended in line with symptoms being observed in COVID-19 patients globally.
Analysis according to accepted clinical risk/ ordinal scale added	WHO ordinal scale have been added to secondary objective analysis	Several organisations, including BMGF and WHO have developed a mortality risk index or ordinal scale for COVID-19 disease severity. Assessment of trial participant's potential disease will be assessed according to the WHO ordinal scale.
Intent to treat analysis modified	Amended to a modified ITT analysis. Participants will be randomised according to the treatment that they actually received, rather than what they were randomised to receive.	A modified intent-to treat analysis is currently the more accepted form of analysis of randomised controlled trials. A modified ITT analysis incorporates the benefits of improved external validity obtained in ITT analyses with improved internal validity obtained in PP analyses. It allows for analysis according to participant's actual experience/ vaccine received, rather than planned experience.

<u>Summary of protocol amendments: Version 2.1 to version 3.0</u> 30th June 2020

Protocol Title: An adaptive phase I/IIa randomised placebo- controlled study to determine safety, immunogenicity and efficacy of non-replicating ChAdOx1 SARS-CoV-2 vaccine in South African adults living without HIV; and safety and immunogenicity in adults living with HIV

Protocol Number: ChAdOx1 nCoV-19_ZA_phI/II

Protocol version, date: Revised Protocol version 3.0, 30th June 2020

Section	Amendment made	Justification
Cover pages	Trial registration added	Registration with Clinicaltrials.gov and Pan African Clinical Trial Registry finalised and numbers added to protocol
	Sponsor updated	University of Oxford has confirmed role of overall sponsor for trial
	Sites added	Additional sites in Gauteng (PHRU Kliptown; SCTC) and W. Cape (CLII, FAMCRU) have been added to assist with rapid enrolment of participants.
	External monitor changed from SCT consulting to PPD	Added PPD, who will be doing the blinded monitoring, as per requirements outlined in BMGF grant agreement and monitoring capacity for increased sample size<br>SCT will perform unblinded monitoring and provide regulatory support
Synopsis, group details tables	Sample size increased. Group 1 increased from 50 to 70 participants, and overall sample size increased from 2000 to 2020 participants	Enrolment was initiated on 24th June 2020, and 8 participants were enrolled daily on 24th, 25th & 26th June 2020. Six of the first 24 (25%) participants tested positive for SARS-CoV-2 on nasal swab at enrolment visit, which has led to higher than anticipated non-evaluable participants.<br>An additional 20 participants will be enrolled into Group1 to ensure adequate evaluable participants in safety cohort.
	Nasal swab for SARS-CoV-2 PCR testing will be collected at screening visit in 96 hours prior to randomisation	Twenty-five percent of first 24 participants enrolled tested positive for SARS-CoV-2 at enrolment. In order to ensure that this asymptomatic/ pre-symptomatic COVID-19 disease is identified prior to vaccination visit, a nasal swab for SARS-CoV-2 PCR will be collected in the 96 hours prior to vaccination visit.
	Serological (IgG) testing at screening	Participants need to be seronegative at vaccination visit to fulfil efficacy endpoint. Addition of immunology blood sample to screening visit to assess prior infection with SARS-CoV-2 (already screening/ safety bloods collected at screening)
	Amended Screening process	A new, reasonably abridged screening informed consent form is being implemented, which will allow for all screening procedures, including data collection (demographics, medical &

		surgical history) and safety and screening bloods. Additionally, a nasal swab for SARS-COV-2 testing collected at screening visit has been added to reduce the possibility of enrolling SARS-CoV-2 infected participant.<br>The previously-approved main ICF will be modified and signed at the enrolment (vaccination) visit.<br><br>Implementation of screening ICF will avoid interested volunteers, who become screening failures based on SARS-CoV-2 positivity (currently 25%) having to read detailed ICF at screening visit.
Objectives	Disease severity grading amended	DSMC advised not to use the NEWS65 grading scale, but rather to utilise grading scale based on CEPI criteria
Inclusion & Exclusion criteria	Amended	Previous and current COVID-19 disease included as exclusion criteria.<br>Chronic diseases clarified
Schedule of events	Vaccination window amended from day 28±3 days to day 28±7	Amended in line with UK trials of ChAdOx1 nCoV-19
	Adverse events grading scale	Protocol updated to utilise DAIDS table throughout.
Informed consent forms	New screening ICF implemented	New screening ICF implemented to cover screening procedures, including SARS-CoV-2 blood and nasal swab testing
	ICFs amended	HIV&Hep B ICF amended: will be signed at screening visit<br>'Main' ICF amended; removed screening procedures and will be signed at enrolment as 'Enrolment' ICF<br>Sample storage ICF amended to reflect 25 years sample storage

<u>Summary of protocol amendments: Version 3.0 to version 3.1</u> 13th July 2020

Protocol Title: An adaptive phase I/IIa randomised placebo- controlled study to determine safety, immunogenicity and efficacy of non-replicating ChAdOx1 SARS-CoV-2 vaccine in South African adults living without HIV; and safety and immunogenicity in adults living with HIV

Protocol Number: ChAdOx1 nCoV-19_ZA_phI/II

Protocol version, date: Revised Protocol version 3.1, 13th July 2020

Section	Amendment made	Justification
Protocol signature page	Added	Added at request of sponsor
SoE, exclusion criteria	COVID-19 serological testing at screening visit to exclude volunteers who have had previous infection has been removed	Recent FDA guidelines suggest not screening for past infection, as future vaccines for the following reasons:<br><br>'although establishing vaccine safety and efficacy in SARS-CoV-2 naïve individuals is critical, vaccine safety and COVID-19 outcomes in individuals with prior SARS-CoV-2 infection, which might have been asymptomatic, is also important to examine because re-vaccination screening for prior infection is unlikely to occur in practice with the deployment of licensed COVID-19 vaccines'<br><br>Additionally, logistical constraints in the laboratories have hampered timely release of serology results.
Holding rules	Clarified	Protocol not clear on holding rules. DSMB suggested amendment.
Objectives	Objectives amended to reflect participants will be stratified by SARS-CoV-2 serological status	Amended in line with removal of screening serological testing and trial exclusion if seropositive at screening
Amendment history	Protocol amendment history added as appendix	Added at request of sponsor
Screening ICF	Screening procedure (blood for SARS-CoV-2 serology) and exclusion criteria amended.	Amended to reflect changes made to protocol.

Summary of protocol amendments: Version 3.1 to version 4.0 19th August 2020

Protocol Title: An adaptive phase I/II randomised placebo- controlled study to determine safety, immunogenicity and efficacy of non-replicating ChAdOx1 SARS-CoV-2 vaccine in South African adults living without HIV; and safety and immunogenicity in adults living with HIV

Protocol Number: ChAdOx1 nCoV-19_ZA_phI/II

Protocol version, date: Revised Protocol version 4.0, 19th August 2020

Section	Amendment made	Justification
Cover page	Version update	Version update
Synopsis summary table of groups	Updated that Group 2 to receive two doses of study-intervention.	Phase I UK data indicate enhanced immunogenicity after two dose schedule.
Synopsis summary table of groups	Corrected window period for 2nd dose (28 days +-7 days)	Corrected to align to text.
Synopsis schedule 2b table	Corrected blood volume at V3 and clarified HLA done at V2. Also, edited to indicate only two dose schedule visits<br>Visit windows corrected to align with visit numbers	Corrections to align to text and also to confirm two dose schedule visits.
Synopsis (and main text) Visit schedule 2a table	Edited to indicate only two dose schedule visits<br>Visit windows corrected to align with visit numbers	Confirm two dose schedule visits
Objectives (Synopsis and main text Section 4.0)	Primary objective changed for endpoints occurring more than 14 days after 2nd dose	Decision to use a two-dose schedule based on enhanced immunogenicity.
Objectives (Synopsis and main text Section 4.0)	Endpoints occurring more than 21 days after first dose relegated to secondary objective	Decision to use a two-dose schedule based on enhanced immunogenicity.
Section 3.5	Data from Phase I UK study added.	Data informed dosing schedule and decision to pursue 2 dose schedule in the study.
Section 5.0 (Trial design)	Changes to confirm that two dose schedule being used	2-dsoe schedule was an option earlier on, and now implemented for Group 2 based on the Phase I data from the UK. SAPHRA and Ethics committee already notified.
Table 7	Updated that Group 2 to receive two doses of study-intervention.	Phase I UK data indicate enhanced immunogenicity after two dose schedule and DSMC concur with two dose schedule.
6.3.2	Clarification on timing on 2nd scheduled dose if participant develops COVID-19 prior to 2nd dose	Ensure 2nd dose only given when clinically stable and have shown adequate recovery from Covid-19.
7.3	Change “Immunology” to “genetics”	Correction

7.4.3.1	Updated to indicate to dose schedule to be used in Group 2	Phase I UK data indicate enhanced immunogenicity after two dose schedule and DSMC concur with two dose schedule.
8.4	Duration between vaccine removal from freezer and use amended from 1 hour to 6 hours	In line with recommendations from manufacturer
8.5	Update of most recent data from other studies	More than 9,000 now vaccinated in UK study
8.5	Detail on clinical strengths of different vaccine doses	Analysis of lot-lot clinical strengths and rational for change in dose range from 5.0 to 7.5 x10^9 vp on Advent qPCR assay.
8.5	Addition of information about batch consistency and amended dose range to account for maintaining consistency for K0011 batch	Batch K0011 was originally dosed on qPCR and from the CMO in Italy, However, additional assays suggest a higher dose is appropriate to maintain consistency with previous batches of vaccine and so this has been amended in the protocol and IB.
9.8	Update on reporting of SAEs.<br>Addition of Grade 4 laboratory AEs and Hy's law SAEs	Clarification of reporting of expected AE from vaccine and SAEs in line with changes to sponsor protocol
10.2	Primary endpoint/objective changed to endpoints occurring more than 14 days after 2nd dose	Align endpoint with new 2 dose schedule for Group 2, based on UK Phase I data.
10.2	Clarified only participants receiving the planned dose of vaccine are eligible for primary endpoint analysis.	Appears that the latest batch of vaccine (Lot K0011) might have lower concentration per milliliter than initially analyzed for. Some participants (N=XX) have received vaccine from this lot, and may have been under-dosed. These participants will be informed of them having been possibly been under-dosed (without unblinding). For purpose of analyses, these participants remain eligible for the sensitivity and secondary objectives, but are excluded from the primary endpoint analysis.
10.2	Inclusion the possibility of being involved in a pooled analysis for the primary endpoint that will include data from the studies underway in Brazil and UK.	To get an early readout of the efficacy of the study, which will be of global benefit, it is proposed that should it be observed there is significant decline in Covid-19 endpoint cases across the three studies (SA, UK and Brazil), that results for the primary endpoint may be pooled. This will be done without unblinding of study staff or participants, so that the study can reach their individual powered endpoints.
10.3	Revision of secondary endpoints	Aligned with change to a two dose schedule, and efficacy endpoints following a single dose now being secondary objective.
17	Updated reference	Added reference of UK Phase I study

Summary of protocol amendments: Version 4.0 to version 4.1 18th September 2020

Protocol Title: An adaptive phase I/II randomised placebo- controlled study to determine safety, immunogenicity and efficacy of non-replicating ChAdOx1 SARS-CoV-2 vaccine in South African adults living without HIV; and safety and immunogenicity in adults living with HIV

Protocol Number: ChAdOx1 nCoV-19_ZA_phI/II

Protocol version, date: Revised Protocol version 4.1, 30th September 2020

Section	Amendment made	Justification
Investigators	Carmen Briner replaced Erica Lazarus as PHRU Kliptown site principal investigator	Erica Lazarus on sabbatical. Carmen Briner approved by SAHPRA as site PI
Schedule of events tables	PAX gene testing removed from group 2b	PAX-gene testing only being done for Group 1, 2a and 3 participants
Schedule of events tables	Group 2b HLA test added to day 28 visit	Inadvertently omitted in previous SoE tables
Schedule of events tables	Amendment of day 56 visit: amended to be 28 days ±7 after dose 2	The aim of the day 56 visit is to collect immunology samples 28 days after receipt of both doses of vaccine. The timing of the day 56 visit should therefore be calculated in relation to the date of receipt of the 2nd dose of study vaccine. The window period for the day 56 visit is date of dose 2 + 28 days (±7).<br>Visits conducted prior to 9th September 2020 which are in alignment with previous protocol will not be regarded as protocol deviations. Clarification to protocol sent to HREC & investigators on 9th Sept 2020
Sample size- group 3	Increased from 50 to 100 participants	Expect ~ one-third to be sero-positive for SARS-CoV-2, hence having 100 will allow for approx. 30 vaccinees being sero-negative
Secondary objective added: group 3 (HIV-infected) participants	To descriptively compare immune responses to ChAdOx1 nCoV-19 in people living with HIV to HIV-uninfected individuals, overall and stratified by COVID-19 sero-status at enrolment.	This trial is the first ChAdOx1 nCoV1-9 vaccine trial which includes people living with HIV. Comparison of immune response in HIV-negative and HIV-positive participants will support planning for future trials and programmatic vaccine implementation
8.2	Revision to the manufacture, packaging, and labelling	To update the manufacture, packaging and labelling relocation from Clinical Biomanufacturing Facility (CBF), University of Oxford to the following GMP facilities - Cobra

		Biologics Limited, Symbiosis Pharmaceutical Services Limited, Advent Societa' A Responsabilita Limitata, and Thermofisher Scientific
8.3	Revision to the storage conditions of the vaccine	To update the storage condition requirements per vial batch
8.4	Revision to administration of the vaccine	To update the vaccine administration section to include information on the vials (Batch 20482B)
8.5	Rationale for dose	To update the analytical comparability assessment of ChAdOx1 nCoV-19 (AZD1222) details since the previous version of the protocol.
